# A systematic revision of *Operclipygus* Marseul (Coleoptera, Histeridae, Exosternini)

**DOI:** 10.3897/zookeys.271.4062

**Published:** 2013-02-20

**Authors:** Michael S. Caterino, Alexey K. Tishechkin

**Affiliations:** 1 Department of Invertebrate Zoology, Santa Barbara Museum of Natural History, 2559 Puesta del Sol, Santa Barbara, CA 93105 USA

**Keywords:** Histeridae, Histerinae, Exosternini, *Operclipygus*, myrmecophily, Neotropical region

## Abstract

We revise the large Neotropical genus *Operclipygus* Marseul, in the histerid tribe Exosternini (Histeridae: Histerinae). We synonymize 3 species, move 14 species from other genera, sink the genus *Tribalister* Horn into *Operclipygus*, and describe 138 species as new, bringing the total to 177 species of *Operclipygus*. Keys are provided for the identification of all species, and the majority of the species are illustrated by habitus and male genitalia illustrations. The species are diverse throughout tropical South and Central America, with only a few species extending into the temperate parts of North America. The majority of species can be recognized by the presence of a distinct stria or sulcus along the apical margin of the pygidium, though it is not exclusive to the genus. Natural history details for species of *Operclipygus* are scant, as most specimens have been collected through the use of passive flight interception traps. Many are probably generally associated with decaying vegetation and leaf litter, where they prey on small arthropods. But a small proportion are known inquilines, with social insects such as ants and termites, and also with some burrowing mammals, such as *Ctenomys* Blainville. The genus now includes the following species groups and species: *Operclipygus sulcistrius* group [*Operclipygus lucanoides* **sp. n.**, *Operclipygus schmidti* **sp. n.**, *Operclipygus simplistrius* **sp. n.**, *Operclipygus sulcistrius* Marseul, 1870], *Operclipygus mirabilis* group [*Operclipygus mirabilis* (Wenzel & Dybas, 1941) comb. n., *Operclipygus pustulifer* **sp. n.**, *Operclipygus plaumanni* **sp. n.**, *Operclipygus sinuatus* **sp. n.**, *Operclipygus mutuca* **sp. n.**, *Operclipygus carinistrius* (Lewis, 1908) comb. n., *Operclipygus parensis* **sp. n.**, *Operclipygus schlingeri* **sp. n.**], *Operclipygus kerga* group [*Operclipygus kerga* (Marseul, 1870), *Operclipygus planifrons* **sp. n.**, *Operclipygus punctistrius* **sp. n.**], *Operclipygus conquisitus* group [*Operclipygus bicolor* **sp. n.**, *Operclipygus conquisitus* (Lewis, 1902), *Operclipygus friburgius* (Marseul, 1864)], *Operclipygus impuncticollis* group [*Operclipygus bickhardti* **sp. n.**, *Operclipygus britannicus* **sp. n.**, *Operclipygus impuncticollis* (Hinton, 1935)], *Operclipygus panamensis* group [*Operclipygus crenatus* (Lewis, 1888), *Operclipygus panamensis* (Wenzel & Dybas, 1941)], *Operclipygus sejunctus* group [*Operclipygus depressus* (Hinton, 1935), *Operclipygus itoupe* **sp. n.**, *Operclipygus juninensis* **sp. n.**, *Operclipygus pecki* **sp. n.**, *Operclipygus punctiventer* **sp. n.**, *Operclipygus sejunctus* (Schmidt, 1896) comb. n., *Operclipygus setiventris***sp. n.**], *Operclipygus mortavis* group [*Operclipygus ecitonis* **sp. n.**, *Operclipygus mortavis* **sp. n.**, *Operclipygus paraguensis* **sp. n.**], *Operclipygus dytiscoides* group [*Operclipygus carinisternus* **sp. n.**, *Operclipygus crenulatus* **sp. n.**, *Operclipygus dytiscoides* **sp. n.**, *Operclipygus quadratus* **sp. n.**], *Operclipygus dubitabilis* group [*Operclipygus dubitabilis* (Marseul, 1889), *Operclipygus yasuni* **sp. n.**], *Operclipygus angulifer* group [*Operclipygus angulifer* **sp. n.**, *Operclipygus impressifrons* **sp. n.**], *Operclipygus dubius* group [*Operclipygus andinus* **sp. n.**, *Operclipygus dubius* (Lewis, 1888), *Operclipygus extraneus* **sp. n.**, *Operclipygus intermissus* **sp. n.**, *Operclipygus lunulus* **sp. n.**, *Operclipygus occultus* **sp. n.**, *Operclipygus perplexus* **sp. n.**, *Operclipygus remotus* **sp. n.**, *Operclipygus validus* **sp. n.**, *Operclipygus variabilis* **sp. n.**], *Operclipygus hospes* group [*Operclipygus assimilis* **sp. n.**, *Operclipygus belemensis* **sp. n.**, *Operclipygus bulbistoma* **sp. n.**, *Operclipygus callifrons* **sp. n.**, *Operclipygus colombicus* **sp. n.**, *Operclipygus communis* **sp. n.**, *Operclipygus confertus* **sp. n.**, *Operclipygus confluens* **sp. n.**, *Operclipygus curtistrius* **sp. n.**, *Operclipygus diffluens* **sp. n.**, *Operclipygus fusistrius* **sp. n.**, *Operclipygus gratus* **sp. n.**, *Operclipygus hospes* (Lewis, 1902), *Operclipygus ibiscus* **sp. n.**, *Operclipygus ignifer* **sp. n.**, *Operclipygus impositus* **sp. n.**, *Operclipygus incisus* **sp. n.**, *Operclipygus innocuus* **sp. n.**, *Operclipygus inquilinus* **sp. n.**, *Operclipygus minutus* **sp. n.**, *Operclipygus novateutoniae* **sp. n.**, *Operclipygus praecinctus* **sp. n.**, *Operclipygus prominens* **sp. n.**, *Operclipygus rileyi* **sp. n.**, *Operclipygus subterraneus* **sp. n.**, *Operclipygus tenuis* **sp. n.**, *Operclipygus tiputinus* **sp. n.**], *Operclipygus farctus* group [*Operclipygus atlanticus* **sp. n.**, *Operclipygus bidessois* (Marseul, 1889), *Operclipygus distinctus* (Hinton, 1935), *Operclipygus distractus* (Schmidt, 1896) comb. n., *Operclipygus farctissimus* **sp. n.**, *Operclipygus farctus* (Marseul, 1864), *Operclipygus gilli* **sp. n.**, *Operclipygus impressistrius* **sp. n.**, *Operclipygus inflatus* **sp. n.**, *Operclipygus latemarginatus* (Bickhardt, 1920) comb. n., *Operclipygus petrovi* **sp. n.**, *Operclipygus plicatus* (Hinton, 1935) comb. n., *Operclipygus prolixus* **sp. n.**, *Operclipygus punctifrons* **sp. n.**, *Operclipygus proximus* **sp. n.**, *Operclipygus subrufus* **sp. n.**], *Operclipygus hirsutipes* group [*Operclipygus guianensis* **sp. n.**, *Operclipygus hirsutipes* **sp. n.**], *Operclipygus hamistrius* group [*Operclipygus arquus* **sp. n.**, *Operclipygus campbelli* **sp. n.**, *Operclipygus chiapensis* **sp. n.**, *Operclipygus dybasi* **sp. n.**, *Operclipygus geometricus* (Casey, 1893) comb. n., *Operclipygus hamistrius* (Schmidt, 1893) comb. n., *Operclipygus impressicollis* **sp. n.**, *Operclipygus intersectus* **sp. n.**, *Operclipygus montanus* **sp. n.**, *Operclipygus nubosus* **sp. n.**, *Operclipygus pichinchensis* **sp. n.**, *Operclipygus propinquus* **sp. n.**, *Operclipygus quinquestriatus* **sp. n.**, *Operclipygus rubidus* (Hinton, 1935) comb. n., *Operclipygus rufescens* **sp. n.**, *Operclipygus troglodytes* **sp. n.**], *Operclipygus plicicollis* group [*Operclipygus cephalicus* **sp. n.**, *Operclipygus longidens* **sp. n.**, *Operclipygus plicicollis* (Schmidt, 1893)], *Operclipygus fossipygus* group [*Operclipygus disconnectus* **sp. n.**, *Operclipygus fossipygus* (Wenzel, 1944), *Operclipygus foveipygus* (Bickhardt, 1918), *Operclipygus fungicolus* (Wenzel & Dybas, 1941), *Operclipygus gibbulus* (Schmidt, 1889) comb. n., *Operclipygus olivensis* **sp. n.**, *Operclipygus simplicipygus* **sp. n.**, *Operclipygus subdepressus* (Schmidt, 1889), *Operclipygus therondi* (Wenzel, 1976)], *Operclipygus impunctipennis* group [*Operclipygus chamelensis* **sp. n.**, *Operclipygus foveiventris* **sp. n.**, *Operclipygus granulipectus* **sp. n.**, *Operclipygus impunctipennis* (Hinton, 1935) comb. n., *Operclipygus latifoveatus* **sp. n.**, *Operclipygus lissipygus* **sp. n.**, *Operclipygus maesi* **sp. n.**, *Operclipygus mangiferus* **sp. n.**, *Operclipygus marginipennis* **sp. n.**, *Operclipygus nicodemus* **sp. n.**, *Operclipygus nitidus* **sp. n.**, *Operclipygus pacificus* **sp. n.**, *Operclipygus pauperculus* **sp. n.**, *Operclipygus punctissipygus* **sp. n.**, *Operclipygus subviridis* **sp. n.**, *Operclipygus tripartitus* **sp. n.**, *Operclipygus vorax* **sp. n.**], *Operclipygus marginellus* group [*Operclipygus ashei* **sp. n.**, *Operclipygus baylessae* **sp. n.**, *Operclipygus dentatus* **sp. n.**, *Operclipygus formicatus* **sp. n.**, *Operclipygus hintoni* **sp. n.**, *Operclipygus marginellus* (J.E. LeConte, 1860) comb. n., *Operclipygus orchidophilus* **sp. n.**, *Operclipygus selvorum* **sp. n.**, *Operclipygus striatellus* (Fall, 1917) comb. n.], *incertae sedis: O. teapensis* (Marseul, 1853) comb. n., *Operclipygus punctulatus* **sp. n.**, *Operclipygus lama* Mazur, 1988, *Operclipygus florifaunensis* **sp. n.**, *Operclipygus bosquesecus* **sp. n.**, *Operclipygus arnaudi* Dégallier, 1982, *Operclipygus subsphaericus* **sp. n.**, *Operclipygus latipygus* **sp. n.**, *Operclipygus elongatus* **sp. n.**, *Operclipygus rupicolus* **sp. n.**, *Operclipygus punctipleurus* **sp. n.**, *Operclipygus falini* **sp. n.**, *Operclipygus peregrinus* **sp. n.**, *Operclipygus brooksi* **sp. n.**, *Operclipygus profundipygus* **sp. n.**, *Operclipygus punctatissimus* **sp. n.**, *Operclipygus cavisternus* **sp. n.**, *Operclipygus siluriformis* **sp. n.**, *Operclipygus parallelus* **sp. n.**, *Operclipygus abbreviatus* **sp. n.**, *Operclipygus pygidialis* (Lewis, 1908), *Operclipygus faltistrius* **sp. n.**, *Operclipygus limonensis* **sp. n.**, *Operclipygus wenzeli* **sp. n.**, *Operclipygus iheringi* (Bickhardt, 1917), *Operclipygus angustisternus* (Wenzel, 1944), *Operclipygus shorti* **sp. n.** We establish the following synonymies: *Phelisteroides miladae* Wenzel & Dybas, 1941 and *Pseudister propygidialis* Hinton, 1935e = *Operclipygus crenatus* (Lewis, 1888); *Phelister subplicatus* Schmidt, 1893b = *Operclipygus bidessois* (Marseul, 1889). We designate lectotypes for *Operclipygus sulcistrius* Marseul, 1870, *Phelister carinistrius* Lewis, 1908, *Phelister kerga* Marseul, 1870, *Phelister friburgius* Marseul, 1864, *Phelister impuncticollis* Hinton, 1935, *Phelister crenatus* Lewis, 1888, *Phelister sejunctus* Schmidt, 1896, *Pseudister depressus* Hinton, 1935, *Epierus dubius* Lewis, 1888, *Phelister hospes* Lewis, 1902, *Phelister farctus* Marseul, 1864, *Phelister bidessois* Marseul, 1889, *Phelister subplicatus* Schmidt, 1893, *Phelister plicatus* Hinton, 1935, *Phelister distinctus* Hinton, 1935, *Phelister distractus* Schmidt, 1896, *Pseudister latemarginatus* Bickhardt, 1920, *Phelister hamistrius* Schmidt, 1893, *Phelister plicicollis* Schmidt, 1893, *Phelister gibbulus* Schmidt, 1889, *Phelister subdepressus* Schmidt, 1889, *Phelister teapensis* Marseul, 1853, *Phelister pygidialis* Lewis, 1908, *Phelister iheringi* Bickhardt, 1917, and *Phelister marginellus* J.E. LeConte 1860. We designate a neotype for *Operclipygus conquisitus* Lewis, replacing its lost type specimen.

## Introduction

The Exosternini, a tribe in the subfamily Histerinae, represents one of the largest groups of histerid beetles in the New World. With nearly 200 described species, its diversity is rivalled only by Haeteriinae. Exosternini is represented in the New World by 11 described genera: *Phelister* Marseul, 1853, *Pseudister* Bickhardt, 1917, *Tribalister* Horn, 1873, *Baconia* Lewis, 1885, *Hypobletus* Schmidt, 1896, *Conchita* Mazur, 1994, *Nunbergia* Mazur, 1978, *Kaszabister* Mazur, 1972, *Mecistostethus* Marseul, 1870, *Yarmister* Wenzel, 1939 and *Operclipygus* Marseul, 1870 (Mazur 2011). With only a few recent exceptions (*Kaszabister*: Dégallier et al. 2012; *Mecistostethus*: Caterino et al. 2012) they are all poorly known, with undescribed species outnumbering described ones. None of the larger genera have ever been revised, or even adequately defined. In this paper we present a comprehensive revision of one of the largest of these genera, *Operclipygus*.

*Operclipygus* was first established by Marseul for a single Brazilian species, *Operclipygus sulcistrius* Marseul (1870). Both the genus name and that of the type species refer to one of the key characters of the group, a sulcus along the apical margin of the pygidium (abdominal tergite 7), which emphasized its ‘operculate’ nature. Marseul recognized similarities to *Phelister*, though for reasons unexplained placed the genus ‘entre les *Platysoma* et les *Cylistix*’ (tribe Platysomatini). Apparently the unusual cylindrical habitus of the type species prevented subsequent authors (or even Marseul himself) from placing additional species in *Operclipygus*, though many species described in subsequent years had a variably sulcate pygidium. *Operclipygus* remained in Platysomatini through the revisions of Bickhardt (1917, 1921). The majority of species we include here were originally described in *Phelister*, and though several authors recognized the close relationship of many of these (most notably Bickhardt 1917, Hinton 1935c, and Wenzel and Dybas 1941, under other generic names) it was not until Wenzel (1976) studied the type of *Operclipygus sulcistrius* that it was recognized that *Operclipygus* should properly be applied to a significantly larger group. Several authors have been further misled by a near complete (though frequently grudging) dependence on the form of the prosternal/mesoventral junction, first established by Bickhardt (1917) as a primary tribal-level character separating Exosternini, where *Operclipygus* properly belongs, from Histerini, where under other generic names many of these species have been placed. Thus, Bickhardt (1917) established the genus *Pseudister* for several species formerly included in *Phelister* that had the mesoventrite emarginate, and placed it, along with many of the species now included in *Operclipygus*, in Histerini. Hinton (1935) followed him, adding some new species to *Pseudister*, and reassigning some additional species from *Phelister*. Wenzel and Dybas (1941) disagreed with the assertion of a close relationship among all these species, establishing the genus *Phelisteroides* for several of them, and describing many new ones. Finally, in 1976, Wenzel synonymized *Phelisteroides* with *Operclipygus*, recognizing it as Exosternini, and paving the way for the present concept of the genus.

Wenzel (1976) listed 24 species in *Operclipygus*. Dégallier (1982) described an additional species, *Operclipygus arnaudi*. Mazur (1984) moved one additional species into the genus from *Phelister*, and subsequently described an additional one himself (Mazur 1988), and the genus now stands at 27 described species (Mazur 1997, 2011). In the present study we synonymize 3 species, move 14 species from other genera (mainly *Phelister* and *Pseudister*), synonymize the genus *Tribalister* (see *Operclipygus marginellus* group), and describe 138 new species, bringing the total to 177 species of *Operclipygus*.

As the original recognition of the genus rested on the pygidial sulcus, Wenzel's (1976) treatment continued to recognize this as the defining character, adding that the species also have ‘a single lateral pronotal stria, triangular prosternal keel formed by the union of the striae, anterior mesosternal margin broadly, shallowly emarginate, pygidium very densely, almost contiguously punctulate, […], tarsal grooves straight, only their inner margin well defined,’ although he acknowledged this was a tentative, and probably inadequate definition. While the present study expands the boundaries of the genus somewhat, mainly through attempting to define the genus in a more phylogenetically meaningful way, it has become clear that all of the characters listed by Wenzel as defining his concept of *Operclipygus* have multiple exceptions. Through phylogenetic analyses of higher-level relationships among Neotropical Exosternini, these exceptions have begun to be clarified. However, many of the character states found in what we consider unquestionably *Operclipygus* are found in species and lineages that are otherwise rather dissimilar. We largely adhere to the results of a comprehensive phylogenetic analysis of morphological and molecular characters including all named and unnamed species of Neotropical Exosternini (Caterino et al. in prep). However, the support for many critical branches is not great, and some results do not have unambiguous taxonomic implications. We present here what we consider to be the most clearly defensible delineation of the genus, only synonymizing other taxa where the results appear unambiguous. However, there is no question that future refinement and analysis will be necessary. Above all we are concerned with filling out the picture of the species diversity, and presenting sufficient information to allow them to be recognized and identified by other workers. We consider a solid higher taxonomy a much less critical goal in the face of the ongoing biodiversity crisis.

## Materials and methods

Type material of all named species was examined by one or both of the authors. Conventional imaging was done using a Visionary Digital's ‘Passport’ portable imaging system, which incorporates a Canon 7D with MP-E 65mm 1–5× macro zoom lens. Images were stacked using Helicon Focus software. SEM imaging was done on a Zeiss EVO 40 scope, with most specimens sputter-coated with gold. We present only selected images as necessary to identify the species in this paper. However, multiple photographs of all species have been archived in MorphBank (www.morphbank.net ), and are also available through the Encyclopedia of Life (www.eol.org ). Following histerid conventions, total body length is measured from the anterior margin of the pronotum to the posterior margin of the elytra (to exclude preservation variability in head and pygidial extension), while width is taken at the widest point, generally near the elytral humeri.

We present extensive descriptions for the majority of species. At the same time, we term these ‘diagnostic descriptions’, to emphasize the fact that they focus on those character systems in which differences among species are typically found. They are not intended to be exhaustive descriptions of each species’ morphology. We have attempted to make most of them consistent in character content and order, facilitating comparison as well as their reuse of descriptions in other contexts, such as in species pages and other media, which we would generally encourage. However, in a few cases, groups of species are so similar, separable mainly by one or two, often genitalic characters, that it seemed pointless to repeat extensive descriptions for all, in which the important differences might be easily missed. The ‘remarks’ sections attempt to highlight the few most important key characters of each species. Ordering of species within species groups is intended to reflect phylogeny to a certain degree, facilitating comparisons among closely related species and their diagnoses.

The material examined lists provide verbatim data only for holotypes and lectotypes, and summary data for all other material, whether paratypes or nontypes. Standardized (to the extent possible) type localities for each species are presented to facilitate verbatim data interpretation. Within verbatim records, data are enclosed in double quotes, with data on separate labels separated by a slash ‘/’. The summary data generally avoids excessive repetition. Each record begins with the number of specimens exhibiting identical data. Records separated by commas are largely identical, differing only in the datum presented, most frequently distinct dates or collectors. Distinct localities are separated by semicolons, and records from distinct countries are separated by periods (full-stops).

### Specimens were provided by the following institutions:

**AKTC** Alexey Tishechkin Collection, Santa Barbara, USA

**AMNH** American Museum of Natural History, New York, USA

**BDGC** Bruce Gill Collection, Ottawa, Canada

**BMNH** Natural History Museum, London, UK

**CASC** California Academy of Sciences Collection, San Francisco, USA

**CEMT** Coleção de Entomologia, Universidade Federal do Mato Grosso, Cuiabá, Brazil

**CHFP** Fabio Penati Collection, Genoa, Italy

**CHJG** Jeffrey P. Gruber Collection, Madison, USA

**CHND** Nicolas Dégallier Collection, Paris

**CHPK** Piet Kanaar Collection, Leiden, The Netherlands

**CHPWK** Peter Kovarik Collection, Columbus, USA

**CHSM** Slawomir Mazur Collection, Warsaw, Poland

**CMNC** Canadian Museum of Nature, Ottawa, Canada

**CMNH** Carnegie Museum of Natural History, Pittsburg, USA

**CNCI** Canadian National Collection of Insects, Ottawa, Canada

**EMEC** Essig Museum of Entomology, Berkeley, USA

**ECOSUR** El Colegio de la Frontera Sur, San Cristóbal de Las Casas, México

**FMNH** Field Museum, Chicago, USA

**FSCA** Florida State Collection of Arthropods, Gainesville, USA

**GBFM** Museo Fairchild, Universidad de Panama, Panama City, Panama

**IAVH** Instituto Alexander von Humboldt, Villa de Leyva, Colombia

**INBIO** Instituto Nacional de Biodiversidad, San Jose, Costa Rica

**IRSNB** Institut Royal des Sciences Naturelles de Belgique, Brussels, Belgium

**LSAM** Louisiana State Arthropod Museum, Baton Rouge, USA

**LUND** Entomological Museum of Lund University, Lund, Sweden

**MACN** Museo de Ciencias Naturales ‘Bernardino Rivadavia’, Buenos Aires, Argentina

**MCZC** Museum of Comparative Zoology, Harvard University, Cambridge, USA

**MEL** Museo Entomológico de León, Nicaragua

**MHNLS** Museo de Historia Natural La Salle, Caracas, Venezuela

**MHNG** Museum d’Histoire Naturelle, Geneva, Switzerland

**MNHN** Museum National d’Histoire Naturelle, Paris, France

**MSCC** Michael Caterino Collection, Santa Barbara, USA

**MUSM** Museo de Historia Natural, Universidad Nacional Mayor de San Marcos, Lima, Peru.

**OUMNH** Oxford University Museum of Natural History, Oxford, UK

**RMNH** National Natuurhistoisch Museum, Leiden, Netherlands

**SBMNH** Santa Barbara Museum of Natural History, Santa Barbara, USA

**SEMC** Snow Entomology Museum, University of Kansas, Lawrence, USA

**TAMU** Texas A&M University Collection, College Station, USA

**UCONN** University of Connecticut Insect Collection, Storrs, USA

**UDG** Universidad de Guadalajara, Guadalajara, Mexico

**UFPR** Universidade Federal do Paraná, Curitiba, Brazil

**UNESP-IS** Universdade Estadual Paulista, Faculdade de Engenharia de Ilha Solteira, Ilha Solteira, Brazil

**UNL** University of Nebraska State Museum, Lincoln, USA

**USFQ** Universidad San Francisco de Quito, Ecuador

**USNM** National Museum of Natural History, Washington, USA

**WBWC** William B. Warner collection, Phoenix, USA

**ZMHB** Zoological Museum of Humboldt University, Berlin, Germany

**ZISP** Zoological Institute, Russian Science Academy, Saint Petersburg, Russia

### Characters of *Operclipygus*, terminology and variability

**Figure 1.**
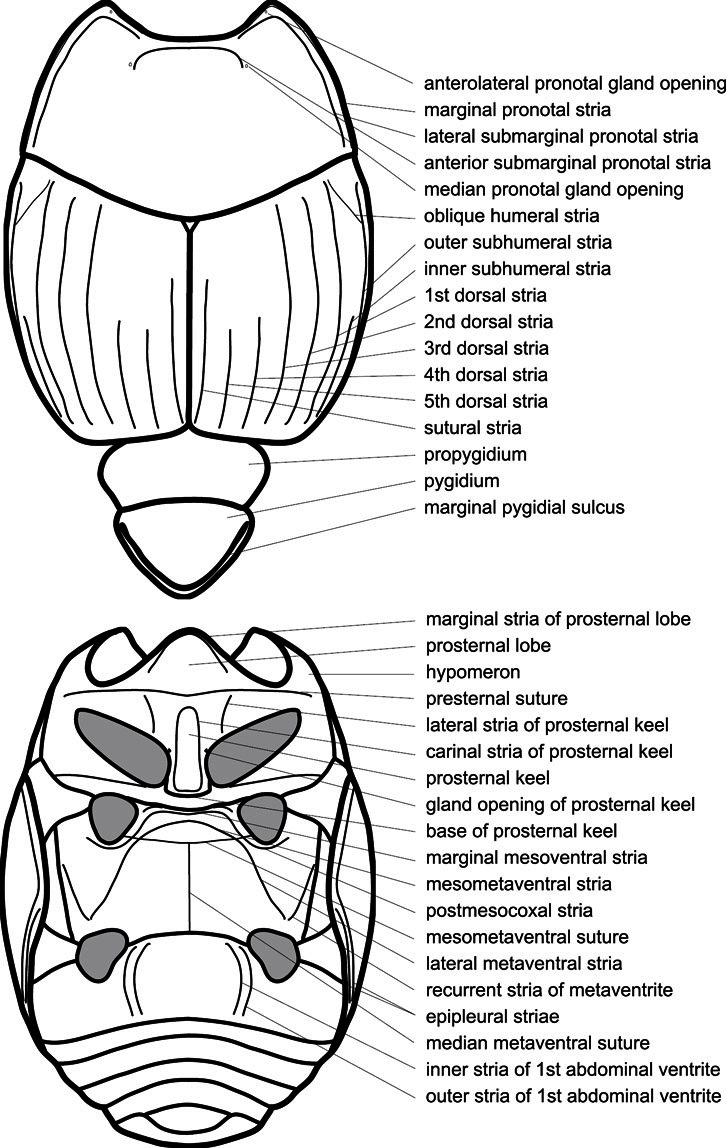
Dorsal (top) and ventral habitus of generalized *Operclipygus*, labeled to show terminology.

**Figure 2.**
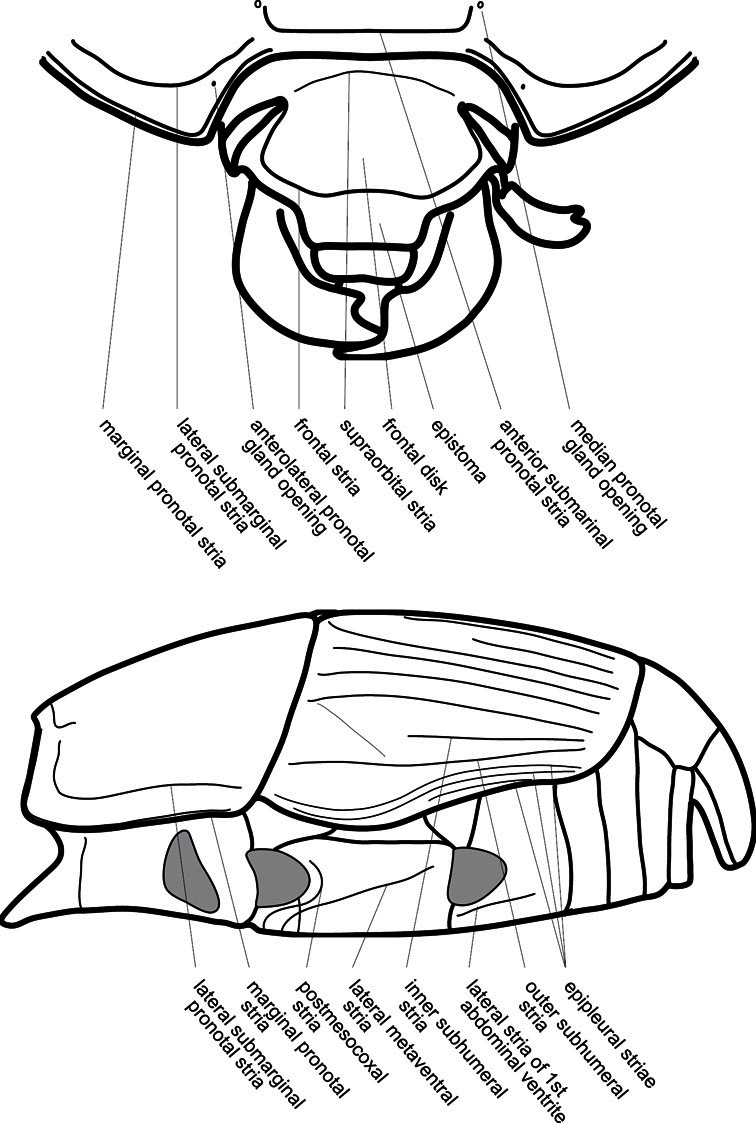
Frontal (top) and lateral habitus of generalized *Operclipygus*, labeled to show terminology.

**Figure 3.**
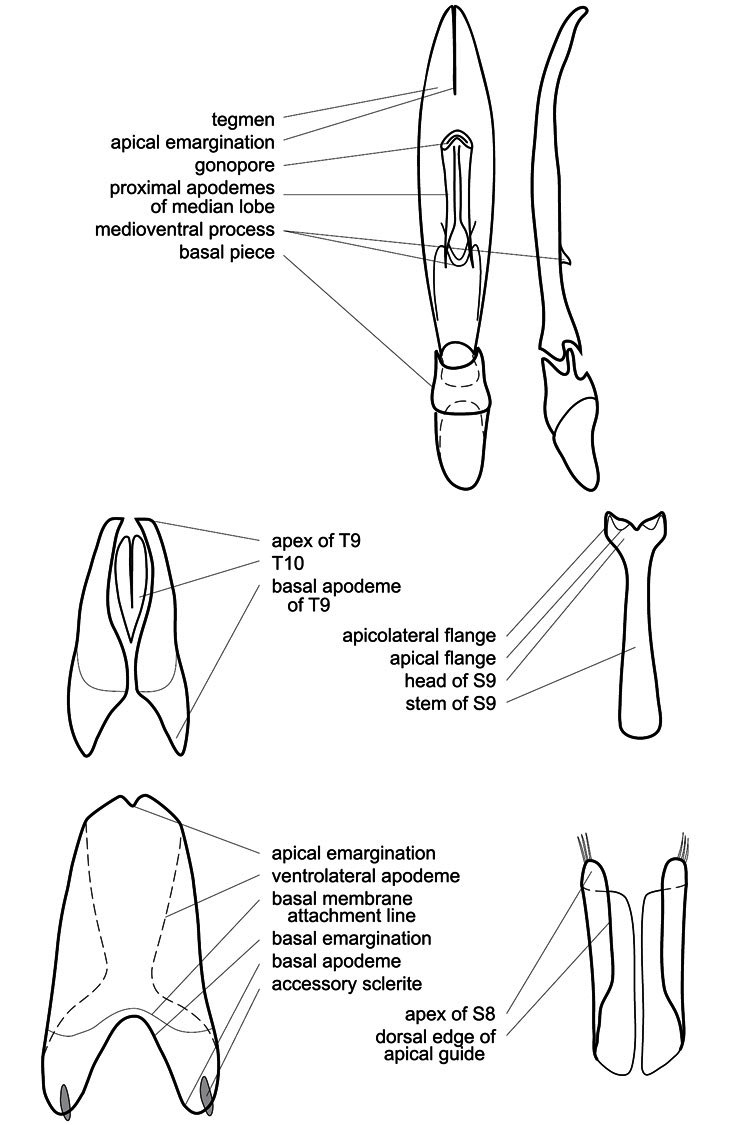
Dissociated male genital segments and aedeagus of *Operclipygus sulcistrius*, labeled to show terminology.

Much of the morphological terminology used is based on Wenzel and Dybas (1941), but modified to follow more recent treatments by Helava et al. (1985), Ôhara (1994), Kanaar (1997) and Lawrence et al. (2011). However, the species included here, and in other Exosternini have required the refinement of some existing terms, and introduction of a few new ones. The labeled illustrations provide an overview of our terminology for external (Figs 1–2) and male genitalic (Fig. 3) morphology.

**Overall body shape** – Body shapes vary considerably among the species of *Operclipygus*, varying in length/width ratio, roundness, whether narrowed more strongly toward the head or toward the pygidium, as well as in dorsoventral convexity. Body color and subtle texturing also show significant variation among species. All these characters of general appearance can be very useful in identification. At the same time they are difficult to describe, and best appreciated in photograph. We provide dorsal habitus photos for most species described in the paper, but we refer readers to online images for other perspectives.

**Figure 4.**
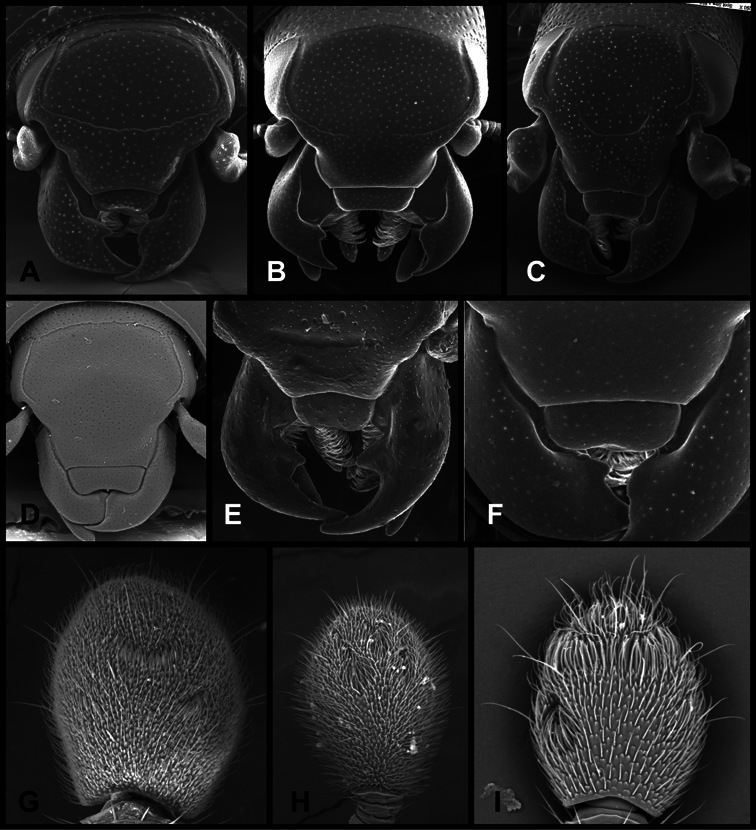
Head characters of *Operclipygus*. **A** Frons of *Operclipygus gilli* **B** Frons of *Operclipygus hamistrius* **C** Frons of *Operclipygus foveiventris* **D** Frons of *Operclipygus nubosus* **E** Mandibles of *Operclipygus schmidti* **F** Mandibles and labrum of *Operclipygus peregrinus* **G** Antennal club of *Operclipygus foveiventris* **H** Antennal club of *Operclipygus rufescens* **I** Antennal club of *Operclipygus confertus*.

**Head** – Informative characters of the head include varying completeness and shape of the frontal stria (Fig. 4). It is frequently interrupted at the middle (Fig. 4B) or doubly interrupted at the sides (Fig. 4C), occasionally both. It may be outwardly arcuate across the entire frons, sinuate over the antennal bases and straight in the middle, or rarely arched dorsad at the middle. Variation within species is usually minimal in these characters, although there are noted exceptions. The supraorbital stria may be present or absent, varies somewhat in degree of impression, and when present may be connected to the sides of the frontal stria or detached.

The convexity of the frons offers a number of useful characters, although these are difficult to adequately describe. Most often the frons and epistoma together are weakly depressed over most of the space between the eyes. There are a few species, or groups of species, like the *Operclipygus impressifrons* group, where this depression is markedly deeper (Fig. 28C). Alternatively, in a few groups, most notably the *Operclipygus kerga* group, the entire frontal area is flat to weakly convex (Fig. 11B).

We utilize a few characters of the labrum in the key and the descriptions, mainly the length/width ratio and the apical shape, which varies from weakly emarginate to weakly rounded. In many species the apical margin appears emarginate across the dorsal margin, but has a distinct process emerging from the ventral surface. This is frequently associated with a pronounced asymmetry in the emargination.

The only mouthpart characters we make regular use of are the presence and shape of basal teeth on the mandibles. The majority of species have a small subacute tooth at the base of the right mandible, but nothing on the left (Figs 4C, F). Where the left mandible bears a tooth it is generally larger and blunter than that on the right (Fig. 11B). It is rare, and taxonomically useful, to have both teeth present and more or less similar in appearance (Fig. 4E). There is relatively little variation in other mouthparts, and they are not detailed beyond the generic description, except in a couple of distinctive cases.

The antennal club exhibits some variation, although most of that is at higher levels in the tribe. Most *Operclipygus* have a similar club in which the basal annulus is weakly inwardly arcuate and interrupted, the middle annulus is weakly widened and densely setose in the middle of the dorsal and ventral surfaces, and the apical annulus is reduced to small setose patches on dorsal and ventral surfaces (Fig. 4G). The middle annulus is interrupted in numerous species, so that it appears to terminate in expanded setose patches (Fig. 4H). In a small number of species in the *Operclipygus hospes* group, there is a distinct round sensorium on the upper basolateral surface of the club (Fig. 4I) We have not made extensive use of these characters due to the difficulty of observing them clearly in the majority of specimens.

**Figure 5.**
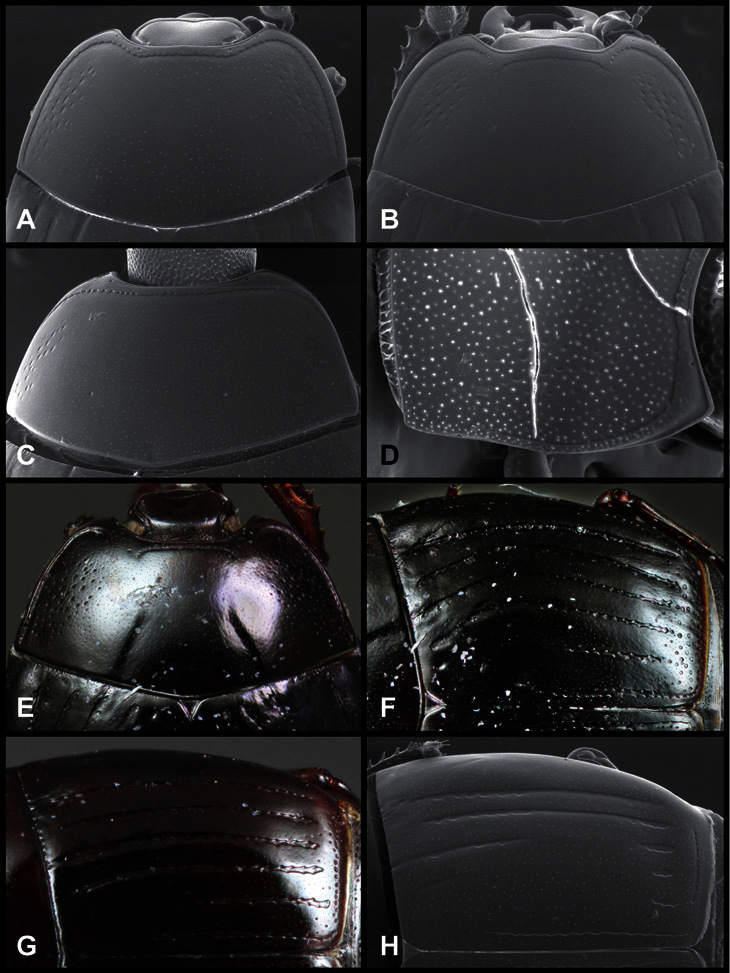
Pronotal and elytral characters of *Operclipygus*. **A** Pronotum of *Operclipygus farctus* **B** Pronotum of *Operclipygus punctiventer* **C** Pronotum of *Operclipygus foveiventris* **D** Pronotum of *Operclipygus hamistrius* **E** Pronotum of *Operclipygus mortavis* **F **Right elytron of *Operclipygus mortavis* **G** Right elytron of *Operclipygus maesi* **H** Right elytron of *Operclipygus foveiventris*.

**Pronotum** – The pronotum offers a large number of characters informative for phylogenetic relationships and for species recognition. Perhaps the most significant are newly documented gland openings on the pronotal dorsum. External gland openings, while numerous in Histeridae, have not been well documented. They are in many cases easily homologized among disparate taxa, though a more thorough accounting is needed. Most Neotropical Exosternini exhibit two pairs of gland openings along the anterior pronotal margin. In a few groups, particularly in *Operclipygus*, the medial pair of these openings varies greatly in position, ranging from the plesiomorphic position along the margin behind the eyes (Fig. 5A) to the middle or even further posterad on either side of the pronotal disk (Fig. 5B, C). Several lineages can be easily diagnosed through their position. While at first these openings are not obvious, in most clean specimens they are readily visible.

The various striae of the pronotum are also very useful in recognizing species and groups of species. The marginal stria generally runs continuously along the front and sides of the pronotum, usually immediately dorsad the actual edge. In many species it is broadly interrupted behind the head (Fig. 5A), or occasionally simply weakened. In some members of the *Operclipygus hamistrius* group the marginal stria crosses the lateral margin near the anterolateral corner, descending to run along the ventral margin (Fig. 5D). In a few such cases there is a distinct, fine groove where it crosses the margin. What we call the lateral submarginal stria is generally (e.g. Ôhara 1994, Kanaar 1997) referred to simply as the lateral stria. However, in many *Operclipygus* this stria is continuous with a stria behind the anterior pronotal margin, and we prefer to refer to this entire stria as submarginal stria, with lateral and anterior portions. It is frequently interrupted behind the eye, with the ends of the anterior portion recurved for a variable distance posterad (Fig. 5B). This nearly always occurs in concert with the median pronotal gland openings being displaced posterad, suggesting a developmental relationship between them. We refer to the narrow portion of the pronotal disk, between the lateral marginal and submarginal striae as the pronotal bead.

Many Neotropical Exosternini exhibit a distinct prescutellar impression, although this is relatively rare in *Operclipygus* species. Some groups, however, do consistently possess one. In many members of the *Operclipygus hospes* and *Operclipygus dubius* groups the prescutellar impression is distinctly elongate, frequently longer than the scutellum, occasionally flame-shaped, and a few species (*Operclipygus dubitabilis* group) have a small punctiform impression. However, *Operclipygus* almost never exhibit a prescutellar impression that is distinct and elongate oval (as in many *Phelister*) and never larger, approaching semicircular (as in several species related to *‘Phelister’ blairi* Hinton).

A number of species exhibit what are commonly termed plicae (Fig. 5E). These are a pair of more or less linear depressions extending forward from the base of the pronotum to about its midpoint, usually immediately in front of the bases of the 3^rd^ dorsal elytral striae. These are distinct from stria in that they generally delimit the outer edge of a broad medial depression. The pronotal disk is elevated laterad the plica.

**Elytra** – Characters of the elytra mostly comprise the traditional configuration of a highly conserved system of longitudinal striae (see Fig. 1). Common modifications include abbreviation from either the anterior or posterior (basal or apical, respectively) ends, some variation in depth of impression, and rarely connection of one or more striae at their basal or apical ends. In a small number of species, primarily those we place in the *Operclipygus marginellus* group, the outer subhumeral stria is sub- or fully carinate, defining a lateral elytral border separating the dorsum from the epipleuron. *Operclipygus* species exhibit variation in the number of epipleural striae, which lie very close and parallel to the lateral elytral margin along the side of the body. Many descriptions include the number of ‘complete’ epipleural striae (see Fig. 2). In such cases an additional abbreviated stria may be present. The epipleural striae are always well separated and distinguishable from the subhumeral stria(e).

Other important characters of the elytra include various sculpturing of the disk, particularly along the apical margin. Some species and groups, especially the *Operclipygus sejunctus* group, exhibit disorganized punctures in the apical half of the elytron, generally increasing in density apically (Fig. 5F). In other groups, especially the *Operclipygus impunctipennis* group, there may be a well organized series of punctures along the apical margin (Fig. 5G), and in some these coalesce into a distinct stria (Fig. 5H).

**Figure 6.**
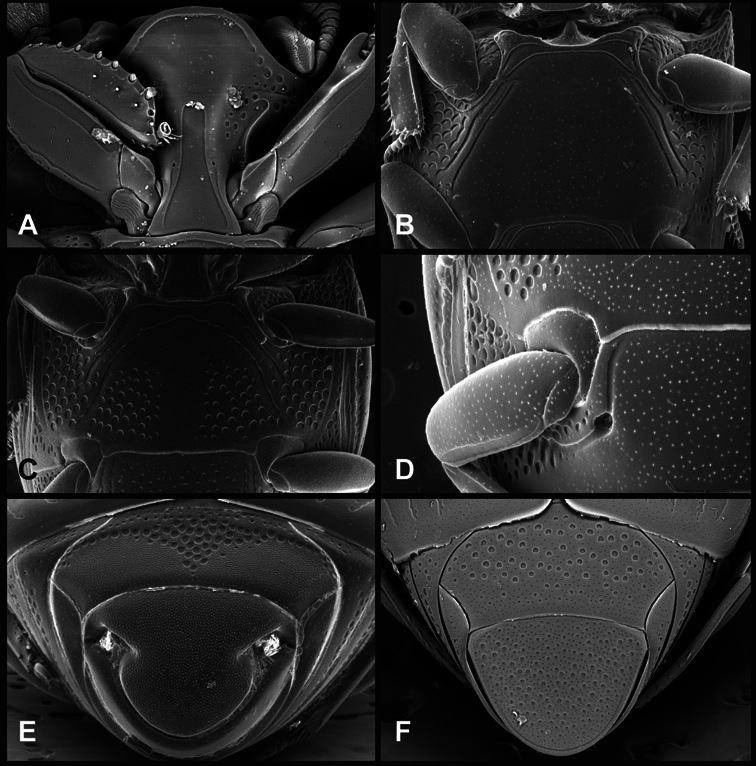
Ventral and abdominal characters of *Operclipygus*. **A** Prosternum of *Operclipygus lissipygus* **B** Meso- and metaventrites of *Operclipygus schmidti* **C** Meso- and metaventrites of *Operclipygus punctiventer* **D** First abdominal ventrite of *Operclipygus foveiventris* **E** Pygidium and propygidium of *Operclipygus fossipygus* **F** Right elytron of *Operclipygus hamistrius*.

**Venter** – The sterna and ventrites have numerous characters that vary among species and species groups. One of the most significant ventral character systems is the prosternal-mesoventral junction. Traditionally Exosternini as a whole have been defined by an emarginate prosternal base (e.g. Bickhardt 1917). This has been discussed and discredited (e.g. Wenzel 1976), primarily due to the full range of variation exhibited in *Operclipygus*, from a strongly produced prosternal keel to a strongly emarginate one. The breadth of the prosternum varies considerably in the genus, though this is difficult to describe. The carinal striae of the prosternum are almost invariably present and complete, but they vary somewhat in the angle of convergence and anterior or posterior connection. We refer in some descriptions to secondary striae, which are fine strioles occasionally present alongside the base of the primary carinal striae (Fig. 6A). The prosternal lobe varies in shape and development, as well as in the presence and completeness of its marginal stria, but these are relatively minor and only detailed in descriptions where particularly distinct.

Beyond the anterior shape of the mesoventrite, its major characters regard the presence of marginal and discal mesoventral striae. The marginal stria is usually present, and ranges from complete and distinct to broadly interrupted. Where interrupted it is often approached by what we term the mesometaventral stria. This latter stria has its apparently plesiomorphic position coincident with the mesometasternal suture. But in most *Operclipygus*, it is variably displaced anterad. The mesometaventral stria generally forms a continuous arch with the lateral metaventral stria (Fig. 6C). However, in a few taxa, particularly in the *Operclipygus marginellus* group, these are interrupted laterally, both terminating in a moderate to distinct depression at the inner edge of the mesocoxa.

The metaventrite exhibits a few useful characters of sculpture and striation. The most important and variable is completeness and position of the lateral metaventral stria. The commonest state is that it extends at a slight angle more or less straight and completely toward the inner anterior corner of the metacoxa, though it is commonly abbreviated apically. It may extend in a more lateral direction, toward the middle or outer corner of the metacoxa, in which case it is more often curved. Rarely it meets a recurrent stria, which recurves forward toward the metepisternum. A few species groups (especially *Operclipygus sulcistrius* and *Operclipygus sejunctus* groups) have a secondary lateral stria that parallels the primary metaventral stria along its outer edge for some or most of its length (Fig. 6C). A few species, primarily those in the *Operclipygus sejunctus* group, exhibit coarse punctures on the metaventrite (Fig. 6C). A small number of species show a dimorphism in which the meta- and mesoventrites are depressed and/or finely setose in the males (Fig. 19G).

The main characters of the abdominal ventrites are related to the lateral striae of the first visible ventrite (morphologically the 3^rd^ sternite), which run anteroposteriorly along the inner edge of the metacoxa. There may be one or two of these, termed the inner and outer, and they may be variably complete to obsolete. Frequently the outer stria is abruptly curved laterad behind the metacoxa. Essentially all *Operclipygus* exhibit a small gland opening near the posteromedial corner of the metacoxa (Fig. 34A), as do many other Exosternini. In some species, particularly in the *Operclipygus impunctipennis* group, this fovea is enlarged, sometimes greatly (Fig. 6D), and may also be displaced laterad, usually in association with a bent outer lateral abdominal stria. Otherwise few characters of the abdominal venter are used.

**Pygidia** – While technically part of the abdomen, the 6^th^ and 7^th^ abdominal tergites, termed the propygidium and pygidium, merit special discussion in any Histeridae. These are greatly varied in punctation in *Operclipygus*. Most importantly they frequently exhibit a very fine, dense ground punctation (Fig. 6E), against which background larger, coarser secondary punctures may be variously impressed. The propygidium rarely exhibits lateral striae, but is generally free of striae. The apical marginal stria/sulcus of the pygidium is, however, extremely varied and important for species recognition. It is completely absent in only very few species, particularly in the *Operclipygus hospes* group. In most species it is coarsely and completely impressed, with the depth, coarseness and completeness varied among species. The most remarkable modifications include foveate basal enlargements (Fig. 6E), which conceivably funtion as mycangia (Caterino and Tishechkin, pers. obs.). These are, however, found only in a few common species in the *Operclipygus fossipygus* group.

**Legs –** We utilize relatively few characters of the legs in the descriptions and keys in this paper. The tibiae are markedly expanded in a few species, providing useful characters, mainly within the *Operclipygus marginellus* group. The tarsi and their claws also provide valuable characters for distinguishing a couple species groups, especially in the arrangment of ventral setae, shape of the apical tarsomere, and shape of the claws. While there is considerable variation in marginal tibial dentation and spines, we have not found states that are sufficiently distinct to be useful for grouping or for species recognition, except in a few extreme cases.

**Genitalia, male** – Male genitalia, including the telescoping 8^th^-10^th^ abdominal segments, are extremely useful for recognizing species and species groups, and are described and illustrated extensively throughout the paper (see Fig. 3 for terminology). Accessory sclerites at the base of the 8^th^ tergite (T8) were first noted in Exosternini by Kanaar (1997), who observed muscle attachment to them. These small sclerites are present in most, but not all *Operclipygus*. In a few cases, we must acknowledge that their absence may be artifactual, as they may become dissociated during dissection. However, they are consistently absent in a few subgroups of species. A new character which we utilize in descriptions relates to the position of the attachment line of the basal membrane of T8. The basal apodemes of this segment lie within the tube of the genitalia, while the distal portion of the tergite forms an external surface. How deeply embedded the apodemes are in the genital tube appears characteristic in many species. This depth is described in relation of the distinct membrane attachment line to the tergite's basal emargination. The ventrolateral apodemes of T8 fall into two distinct categories with respect to symmetry. In many species they project most deeply beneath near their bases, tapering toward the apex, while in others the apodemes project most deeply at their middles, tapering evenly basally and apically. The latter state is generally associated with an interruption of the apicolateral margin of the tergite, where the apodemes end, and an abrupt bend to the apex. This is further often associated with a distinct desclerotization near this bend, indicating flexibility of the dorsal apices.

The male 8^th^ sternite (S8) comprises two halves, usually divided along the midline, though they may be fused for some of that length. The sides of the sternites curve upward, usually curving over dorsally, forming a internally open troughs referred to as apical guides. These are quite varied in degree of dorsal enclosure, as well as in shape of the upper inner edge, and are described for most species. The apices of S8 generally bear some setae, though these may be difficult to see, and are not comprehensively described (or illustrated). In a few species the apices are divergent, and may be broadly membraneous, forming a velum, presumably used in maintaining hold and position on the female.

The male T9 and T10 are intimately associated, with the apices of T9 nearly enclosing T10 dorsally. T9 itself shows little variation among groups, with a couple notable exceptions: in the *Operclipygus dubius* group its apices are apparently rotated and expanded to form peculiar and varied distal flanges. Like S8, T9 usually comprises separate lateral halves, approximate near their bases. However, they may be fused at this point (again in most species of the highly apomorphic *Operclipygus dubius* group). T10 also comprises separate lateral halves in most species, though partial or complete fusion along the midline is observed in several taxa. The 9^th^ sternite (S9 - often referred to as the spiculum gastrale) forms an elongate guide that articulates at its apex with the aedeagus. Its shape varies considerably, in basal and apical width, as well as in the depth of emarginations at either end. We generally refer to everything basad the apical expansion as the stem, and the apical portion as the head. The distal margin of the head generally bears an upturned apical flange, which may be complete or interrupted, depending on the depth of the apical emargination. The sides of the head are also variously upturned.

The aedeagus itself comprises the basal piece, the tegmen, and the (internal) median lobe. The proportion of total length accounted for by the basal piece and the tegmen varies among species, less so among groups. The basal piece exhibits some variation in its apical shape, related to tegmen articulation, but we have not made extensive reference to this. The shape of the tegmen is quite variable, though it is most frequently simply narrowly tubular, narrowed and weakly downturned at the apex. Its most important character is the position, shape, and strength of what we term the medioventral process This is a usually dentiform process that may or may not be articulated with the ventrolateral membrane of the tegmen, projecting beneath the tegmen at some distance from its base. In most cases the shape of this process is described as seen in a dorsal view, visible through the body of the tegmen, though the degree of projection is best appreciated in lateral view. The median lobe consists of a pair of proximal apodemes and an apical gonopore. The proximal apodemes may be simple thin rods extending toward the base of the aedeagus, may be uniformly thickened, or are frequently differentiated, with the gonopore ends thicker, and the more proximal portion of the rods thin. The gonopore itself varies primarily in width relative to the tegmen width. The position of the gonopore relative to the apex of the tegmen is probably uninformative due to its presumed mobility for extrusion.

**Genitalia, female –** Female genitalia, characters of the ovipositor, spermathecae, and associated abdominal segments do show some variation, but mostly at higher, intergeneric levels, so beyond a general characterization of the genus (see description, below) we do not detail female genitalia in this paper. Further documentation of variation within *Operclipygus* may help to further refine intrageneric relationships and groupings.

## Taxonomy

### Operclipygus

Marseul, 1870: 75

http://species-id.net/wiki/Operclipygus

Phelisteroides Wenzel & Dybas, 1941: 448; synonymized by Wenzel 1976: 248; Type species: *Phelisteroides fungicolus* Wenzel & Dybas, 1941: 452.
Tribalister Horn 1873: 299; **syn. n.** Type species: *Phelister marginellus* J.E. LeConte, 1860: 311.

#### Type species:

*Operclipygus sulcistrius* Marseul, 1870: 75

#### Description.

***Size range***: Length 1.1–5.6 mm; width 0.8–4.5 mm; ***Body*:** ovoid to elongate, sides broadly rounded to sub- or fully parallel, convex to subdepressed; color rufescent to rufo-brunneus, glabrous, rarely piceous or metallic. ***Head***: Frons usually weakly depressed at middle, rarely strongly depressed, flat, or weakly convex; frontal stria complete, interrupted at middle, or doubly interrupted at sides, usually outwardly arcuate, rarely recurved at middle; supraorbital stria generally present across top of frons, arcuate, connected to upper ends of frontal stria or ending free, rarely absent; epistoma flat to subdepressed, very rarely convex, lacking striae along anterior or lateral margins, apex truncate to weakly emarginate; labrum 1.5–3× as wide as long, flat to weakly convex, apical margin generally weakly emarginate, often slightly asymmetrical with weak tooth projecting ventrad, rarely outwardly arcuate; mandibles short, weakly dentate, with tooth more often on right mandible than left, rarely with both mandibles strongly toothed; antennal scape elongate, slightly expanded to apex; antennal club shape elongate oval, rarely circular, with three annuli, the basalmost two frequently interrupted. ***Pronotum***: Pronotal sides generally narrowed to front, anterior margin behind head straight to weakly, very rarely strongly produced; disk with prescutellar impression small or lacking, never as large or larger than scutellum; disk with marginal and anterior gland openings, the innermost pair of which is frequently displaced posteriorly onto pronotal disk; marginal stria usually present along lateral and anterior margins, often interrupted behind eyes, anterior portion rarely absent; lateral submarginal pronotal stria usually present along all or most of pronotal side, simple or rarely subcarinate, rarely abbreviated from posterior end, disconnected or continuous with anterior submarginal stria; anterior submarginal stria usually present in addition to anterior portion of marginal stria; lateral ends of anterior submarginal stria, if free, frequently recurved posterad, sometimes over considerable portion of pronotal length; pronotal disk variously punctate, usually only at sides, rarely with oblique basal plicae. ***Elytra***: one to three epipleural striae present, usually fine and close together; dorsal striae comprising 8 regular striae, highly variable in presence, completeness and degree of impression; elytral disk rarely with apical punctures, which may be diffuse or form a regular series. ***Prosternum***: Prosternal lobe weakly deflexed, usually rounded, rarely subtruncate apically or strongly shortened, usually with marginal stria present, variably abbreviated at sides; prosternal keel projecting, truncate, or emarginate at base, with two carinal striae usually present, complete, and connected anteriorly by narrow arch. ***Mesoventrite***: Anterior mesoventral margin varied in shape in correlation with prosternal keel; marginal mesoventral stria usually complete, rarely interrupted or absent; mesometaventral stria rarely present along mesometaventral suture, more often displaced anterad, often nearly to anterior mesoventral margin, may be angulate or arcuate, generally continuous at sides with lateral metaventral stria. ***Metaventrite***: Disk flat to weakly convex, generally glabrous, impunctate, rarely depressed and/or setose in males, or punctate in both sexes; postmesocoxal stria present, curving close to rear of mesocoxa toward mesepimeron; lateral metaventral stria oblique, extending from inner corner of mesocoxa toward metacoxa, rarely curving toward metepisternum or continued at side by recurrent stria. ***Abdomen***: Abdominal ventrite 1 with one or two lateral striae along inner edge of metacoxa, the outermost rarely curving laterad behind coxa; gland openings behind metacoxa usually small, inconspicuous, rarely developed into deep foveae; propygidium usually wide, short, with pair of gland openings in anterolateral corners, disk with highly varied punctation; pygidium subtriangular, variably punctate, usually with distinct marginal stria, this occasionally developed into deep sulcus, rarely with large foveae in basolateral corners. ***Legs***: protibial margin generally without strong marginal teeth, with 4-8 weak marginal teeth, each bearing a small spine; submarginal row of spines frequently present, along with one to three longitudinal striae on posterior face, two protibial spurs present, the inner one often weak; protarsal setae often sexually dimorphic, simple in females, broadly expanded in males; protarsal claws usually simple, rarely modifed; meso- and metatibiae lacking marginal teeth, with simple series of generally fine spines; meso- and metatarsi generally with two rows of ventral spines, rarely with spines not organized in rows. ***Male***: Accessory sclerites present or absent; 8^th^ tergite parallel-sided to weakly tapered, with conspicuous basal and apical emarginations, ventrolateral apodemes simple, not meeting along ventral midline; 8^th^ sternite usually divided along midline, rarely fused along part of its length, with sides upturned to form weak to moderately strong apical guides, apices usually bearing few fine setae, rarely with numerous conspicuous setae; 9^th^ tergite divided along midline, very rarely fused near base, apices usually simple, converging but separate, ventrolateral apodemes forming moderately to strongly hooked lateral structures; spiculum gastrale (S9) generally elongate, stem weakly expanded at base, head with apical flanges, apicolateral flanges rarely well developed; 10^th^ tergite elongate, usually divided along midline, rarely fused; aedeagus elongate, narrow, tubular, varied in shape, with apical division rather short, apices rarely convergent, usually with moderately strong ventromedial projection, easily visible from above, and often projecting beneath; median lobe usually simple, with small gonopore, elongate proximal apodemes, proximal apodemes rarely strongly differentiated into proximal and distal portions; basal piece usually one-fourth to one-third tegmen length, laterally articulated with tegmen. ***Female***: 8^th^ tergite forming a single plate, apically emarginate; 8^th^ sternite entire or divided along midline with basal baculi detached, articulated with sternites, basally convergent; 9^th^ sternite present, elongate; valviferae paddle-shaped; coxites elongate, apically bi- to tridentate, with distinct, articulated apical stylus; bursa copulatrx completely membraneous; generally with single weakly sclerotized spermatheca, although multiple spermathecae have been observed, inserted beneath apex of bursa copulatrix; spermathecal shape simple, saclike, or frequently forming complex spiral; spermathecal gland present, inserted at base of spermatheca.

### Recognizing members of *Operclipygus*

Collectively diagnosing the group of related species that we assign to *Operclipygus* is challenging. No single distinctive character remains constant through the group, synapomorphic or otherwise. The presence of a marginal pygidial stria/sulcus is almost sufficient to place a specimen into *Operclipygus*; only a very small number of excluded species exhibit this character. A marginal pygidial stria is seen in most *Mecistostethus*, which have the body strongly depressed and dorsally setose (see Caterino et al. 2012). A few species in a couple of undescribed genera also have a pygidial stria but in one case the pygidium itself strongly prolonged and apically pointed; in another there are deep lateral mesoventral foveae and no sutural elytral stria. Otherwise, if a New World histerine specimen has a marginal pygidial stria, it is *Operclipygus*.

Much harder is placing those true *Operclipygus* that lack a pygidial stria into the genus. Most of the *Operclipygus* that lack this stria are in the *Operclipygus impunctipennis* group, most of which have very fine, dense pygidial ground punctation, a character sufficient to place a specimen in the genus. Others in this group have an apical marginal stria or puncture series on the elytron, also sufficient for placement. Several members of the *Operclipygus hospes* group lack a pygidial stria, but these can easily be recognized by their elongate, subdepressed body form and their two lateral striae on the 1^st^ abdominal ventrite. Finally a few members of the *Operclipygus hamistrius* group lack the pygidial stria, but all show conspicuous transverse microsculpture on the pygidium, and generally will have a broken and recurved anterior submarginal pronotal stria.

Taking a different perspective, it is more or less possible to diagnose some other groups of New World Exosternini that might otherwise be confused with *Operclipygus*:

*Phelister sanguinipennis* group: This is a relatively small group of species that often occur abundantly in flight intercept trap and carrion and dung pitfall trap samples in the neotropics. They may be similar to some species of *Operclipygus*, mainly in that they may have an outwardly arcuate anterior pronotal margin, median pronotal gland openings that are displaced posterad onto the pronotal disk, and an anterior submarginal pronotal stria which may be broken and recurved posterad for some distance. But preliminary analyses do not support a close relationship between the groups. Members of the *Phelister sanguinipennis* group may generally be separated from *Operclipygus* by the following characters: prescutellar impression usually elongate oval, about as long as scutellum; propygidium strongly transverse, often depressed at sides; pygidium relatively short, lacking marginal sulcus; prosternal keel relatively broad, carinal striae generally at least partly effaced; base of prosternal keel emarginate to truncate, never outwardly arcuate; pronotum with marginal stria usually complete across front, anterior submarginal stria rarely present; central portion of anterior pronotal margin often projecting; outer subhumeral elytral stria never complete (may be absent); inner subhumeral stria never present.

*Phelister blairi* group: This is a large group of mostly undescribed species that is destined to be removed from *Phelister* and probably split into multiple genera. The feature by which they most often may be confused with *Operclipygus* is the posteriorly displaced median pronotal gland openings. They may usually be separated from *Operclipygus* by the following characters: median pronotal gland openings distinctly annulate; prescutellar impression larger than scutellum, often much larger and semicircular; frons and epistoma narrowly and deeply impressed, the epistoma usually with subcarinate lateral ridges; prosternal keel usually emarginate at base.

### Checklist of the species of *Operclipygus*

*Operclipygus sulcistrius* group

*Operclipygus sulcistrius* Marseul, 1870

*Operclipygus schmidti* sp. n.

*Operclipygus lucanoides* sp. n.

*Operclipygus simplistrius* sp. n.

*Operclipygus mirabilis* group

*Operclipygus mirabilis* (Wenzel & Dybas, 1941) comb. n.

*Operclipygus pustulifer* sp. n.

*Operclipygus plaumanni* sp. n.

*Operclipygus sinuatus* sp. n.

*Operclipygus mutuca* sp. n.

*Operclipygus carinistrius* (Lewis, 1908) comb. n.

*Operclipygus parensis* sp. n.

*Operclipygus schlingeri* sp. n.

*Operclipygus kerga* group

*Operclipygus kerga* (Marseul, 1870)

*Operclipygus punctistrius* sp. n.

*Operclipygus planifrons* sp. n.

*Operclipygus conquisitus* group

*Operclipygus conquisitus* (Lewis, 1902)

*Operclipygus bicolor* sp. n.

*Operclipygus friburgius* (Marseul, 1864)

*Operclipygus impuncticollis* group

*Operclipygus impuncticollis* (Hinton, 1935)

*Operclipygus britannicus* sp. n.

*Operclipygus bickhardti* sp. n.

*Operclipygus panamensis* group

*Operclipygus panamensis* (Wenzel & Dybas, 1941)

*Operclipygus crenatus* (Lewis, 1888)

*Operclipygus sejunctus* group

*Operclipygus sejunctus* (Schmidt, 1896) comb. n.

*Operclipygus pecki* sp. n.

*Operclipygus juninensis* sp. n.

*Operclipygus depressus* (Hinton, 1935)

*Operclipygus setiventris* sp. n.

*Operclipygus itoupe* sp. n.

*Operclipygus punctiventer* sp. n.

*Operclipygus mortavis* group

*Operclipygus mortavis* sp. n.

*Operclipygus ecitonis* sp. n.

*Operclipygus paraguensis* sp. n.

*Operclipygus dytiscoides* group

*Operclipygus dytiscoides* sp. n.

*Operclipygus carinisternus* sp. n.

*Operclipygus quadratus* sp. n.

*Operclipygus crenulatus* sp. n.

*Operclipygus dubitabilis* group

*Operclipygus dubitabilis* (Marseul, 1889)

*Operclipygus yasuni* sp. n.

*Operclipygus impressifrons* group

*Operclipygus angulifer* sp. n.

*Operclipygus impressifrons* sp. n.

*Operclipygus dubius* group

*Operclipygus dubius* (Lewis, 1888)

*Operclipygus andinus* sp. n.

*Operclipygus intermissus* sp. n.

*Operclipygus variabilis* sp. n.

*Operclipygus validus* sp. n.

*Operclipygus lunulus* sp. n.

*Operclipygus remotus* sp. n.

*Operclipygus occultus* sp. n.

*Operclipygus perplexus* sp. n.

*Operclipygus extraneus* sp. n.

*Operclipygus hospes* group

*Operclipygus hospes* (Lewis, 1902)

*Operclipygus ignifer* sp. n.

*Operclipygus subterraneus* sp. n.

*Operclipygus callifrons* sp. n.

*Operclipygus colombicus* sp. n.

*Operclipygus incisus* sp. n.

*Operclipygus prominens* sp. n.

*Operclipygus tiputinus* sp. n.

*Operclipygus minutus* sp. n.

*Operclipygus inquilinus* sp. n.

*Operclipygus tenuis* sp. n.

*Operclipygus communis* sp. n.

*Operclipygus belemensis* sp. n.

*Operclipygus innocuus* sp. n.

*Operclipygus diffluens* sp. n.

*Operclipygus confluens* sp. n.

*Operclipygus fusistrius* sp. n.

*Operclipygus ibiscus* sp. n.

*Operclipygus novateutoniae* sp. n.

*Operclipygus gratus* sp. n.

*Operclipygus confertus* sp. n.

*Operclipygus rileyi* sp. n.

*Operclipygus assimilis* sp. n.

*Operclipygus praecinctus* sp. n.

*Operclipygus impositus* sp. n.

*Operclipygus curtistrius* sp. n.

*Operclipygus bulbistoma* sp. n.

*Operclipygus farctus* group

*Operclipygus farctus* (Marseul, 1864)

*Operclipygus bidessois* (Marseul, 1889)

*Operclipygus subrufus* sp. n.

*Operclipygus plicatus* (Hinton, 1935) comb. n.

*Operclipygus petrovi* sp. n.

*Operclipygus distinctus* (Hinton, 1935)

*Operclipygus atlanticus* sp. n.

*Operclipygus gilli* sp. n.

*Operclipygus proximus* sp. n.

*Operclipygus prolixus* sp. n.

*Operclipygus impressistrius* sp. n.

*Operclipygus farctissimus* sp. n.

*Operclipygus inflatus* sp. n.

*Operclipygus distractus* (Schmidt, 1896) comb. n.

*Operclipygus punctifrons* sp. n.

*Operclipygus latemarginatus* (Bickhardt, 1920) comb. n.

*Operclipygus hirsutipes* group

*Operclipygus hirsutipes* sp. n.

*Operclipygus guianensis* sp. n.

*Operclipygus hamistrius* group

*Operclipygus hamistrius* (Schmidt, 1893) comb. n.

*Operclipygus geometricus* (Casey, 1893) comb. n.

*Operclipygus rubidus* (Hinton, 1935) comb. n.

*Operclipygus quinquestriatus* sp. n.

*Operclipygus campbelli* sp. n.

*Operclipygus dybasi* sp. n.

*Operclipygus pichinchensis* sp. n.

*Operclipygus rufescens* sp. n.

*Operclipygus impressicollis* sp. n.

*Operclipygus nubosus* sp. n.

*Operclipygus arquus* sp. n.

*Operclipygus propinquus* sp. n.

*Operclipygus intersectus* sp. n.

*Operclipygus troglodytes* sp. n.

*Operclipygus chiapensis* sp. n.

*Operclipygus montanus* sp. n.

*Operclipygus plicicollis* group

*Operclipygus plicicollis* (Schmidt, 1893)

*Operclipygus cephalicus* sp. n.

*Operclipygus longidens* sp. n.

*Operclipygus fossipygus* group

*Operclipygus fossipygus* (Wenzel, 1944)

*Operclipygus foveipygus* (Bickhardt, 1918)

*Operclipygus gibbulus* (Schmidt, 1889) comb. n.

*Operclipygus fungicolus* (Wenzel & Dybas, 1941)

*Operclipygus subdepressus* (Schmidt, 1889)

*Operclipygus simplicipygus* sp. n.

*Operclipygus therondi* Wenzel, 1976

*Operclipygus disconnectus* sp. n.

*Operclipygus olivensis* sp. n.

*Operclipygus impunctipennis* group

*Operclipygus impunctipennis* (Hinton, 1935) comb. n.

*Operclipygus punctissipygus* sp. n.

*Operclipygus granulipectus* sp. n.

*Operclipygus pauperculus* sp. n.

*Operclipygus nicodemus* sp. n.

*Operclipygus tripartitus* sp. n.

*Operclipygus latifoveatus* sp. n.

*Operclipygus lissipygus* sp. n.

*Operclipygus mangiferus* sp. n.

*Operclipygus foveiventris* sp. n.

*Operclipygus marginipennis* sp. n.

*Operclipygus maesi* sp. n.

*Operclipygus pacificus* sp. n.

*Operclipygus nitidus* sp. n.

*Operclipygus subviridis* sp. n.

*Operclipygus vorax* sp. n.

*Operclipygus chamelensis* sp. n.

*Operclipygus marginellus* group

*Operclipygus marginellus* (J.E. LeConte, 1860) comb. n.

*Operclipygus striatellus* (Fall, 1917) comb. n.

*Operclipygus formicatus* sp. n.

*Operclipygus hintoni* sp. n.

*Operclipygus orchidophilus* sp. n.

*Operclipygus baylessae* sp. n.

*Operclipygus selvorum* sp. n.

*Operclipygus dentatus* sp. n.

*Operclipygus ashei* sp. n.

*Operclipygus* incertae sedis

*Operclipygus teapensis* (Marseul, 1853) comb. n.

*Operclipygus punctulatus* sp. n.

*Operclipygus lama* Mazur, 1988

*Operclipygus florifaunensis* sp. n.

*Operclipygus bosquesecus* sp. n.

*Operclipygus arnaudi* Dégallier, 1982

*Operclipygus subsphaericus* sp. n.

*Operclipygus latipygus* sp. n.

*Operclipygus elongatus* sp. n.

*Operclipygus rupicolus* sp. n.

*Operclipygus punctipleurus* sp. n.

*Operclipygus falini* sp. n.

*Operclipygus peregrinus* sp. n.

*Operclipygus brooksi* sp. n.

*Operclipygus profundipygus* sp. n.

*Operclipygus punctatissimus* sp. n.

*Operclipygus cavisternus* sp. n.

*Operclipygus siluriformis* sp. n.

*Operclipygus parallelus* sp. n.

*Operclipygus abbreviatus* sp. n.

*Operclipygus pygidialis* (Lewis, 1908)

*Operclipygus faltistrius* sp. n.

*Operclipygus limonensis* sp. n.

*Operclipygus wenzeli* sp. n.

*Operclipygus iheringi* (Bickhardt, 1917)

*Operclipygus angustisternus* (Wenzel, 1944)

*Operclipygus shorti* sp. n.

### Key to species groups and unplaced species

**Table:** 

1	Outer subhumeral stria subcarinate to strongly carinate, forming a lateral elytral margin of varying strength (Fig. 80A) (where weak, in one species from Central Mexico (Fig. 80G), the epistoma has weak oblique lateral striae); lateral submarginal pronotal stria complete, carinate, very close to margin, pronotal disk depressed along its inner edge (Fig. 80C); body otherwise highly varied in appearance, often strongly carinate (Fig. 80A), or conversely with striae strongly reduced (Fig. 82E); never strongly and densely punctate	*Operclipygus marginellus* group (p. 315)
–	Outer subhumeral stria variously impressed, but rarely carinate; if elytra with lateral carina, then body is densely and uniformly punctate throughout; lateral submarginal pronotal stria, if close to margin, rarely carinate, and pronotal disk not depressed along its inner edge	2
2	Pronotum with a single pair of anterior gland openings, located along pronotal margin behind eye	3
–	Pronotum with two pairs of anterior gland openings, at least one along the anterior margin laterad eye, the second, median opening variable in position, may be close to anterolateral opening or displaced posterad on pronotal disk by as much as three-fourths the pronotal length	4
3	Body size <3 mm; pronotum with strong basal plicae; frontal striae abruptly bent dorsad near antennal bases, sinuate across middle (Fig. 8F); known only from a cave near Tingo Maria, Huanuco, Peru	*Operclipygus schlingeri* sp. n.
–	Body large, >5 mm, subquadrate (Fig. 103); pronotum lacking basal plicae; southeastern Brazil	*Operclipygus iheringi* (Bickhardt)
4	Median pronotal gland openings close to anterior pronotal margin, within approximately 10 diameters (Figs 5A, B)	5
–	Distance between median pronotal gland openings and anterior pronotal margin greater than, usually much greater than, 10 diameters (Fig. 5C); or, pronotal disk strongly and uniformly punctate, obscuring gland openings	41
5	Anterior submarginal pronotal stria continuous with lateral submarginal stria, joined smoothly, without postocular angulation; both pronotal gland openings very close to anterior margin (Fig. 5A), anterior to submarginal stria, median openings no more than two diameters from anterior margin	*Operclipygus farctus* group (p. 182)
–	Anterior submarginal pronotal stria detached from lateral submarginal stria, or meeting at distinct postocular angulation; median pronotal gland openings ranging from 4-10 diameters from anterior pronotal margin	6
6	Distinct pronotal plicae present (Fig. 5E)	7
–	Pronotal plicae absent, pronotum at most weakly flattened across base	8
7	Frons flat to convex, with frontal stria complete, transverse; body not unusually large	*Operclipygus mirabilis* group (females) (p. 39)
–	Frons strongly impressed at middle (Fig. 22A), frontal stria sinuate laterally or interrupted over antennal bases; body large, rounded, strongly convex	*Operclipygus mortavis* group (p. 91)
8	Metaventrite and first abdominal ventrite with coarse punctures (Figs 6C, 19B); second lateral metaventral stria present laterad primary stria; elytra usually with numerous poorly organized punctures toward apex (Fig. 5F)	*Operclipygus sejunctus* group (p. 77)
–	Metaventrite (and usually first abdominal ventrite) lacking coarse punctures; other characters variable	9
9	Body elongate, parallel-sided (Fig. 7A); prosternal lobe large, subtruncate apically (Fig. 7F); both mandibles with strong basal tooth (Fig. 4E); secondary lateral metaventral stria present	*Operclipygus sulcistrius* group (p. 34)
–	Body form varied; left mandible usually without, or with only small tooth; prosternal lobe rounded, never subtruncate apically; secondary lateral metaventral stria rarely present	10
10	Body small (usually <2mm), usually elongate, more or less parallel-sided; 1^st^ abdominal ventrite with two complete lateral striae, usually well separated (Figs 30C, 34A); prescutellar impression present, narrow, elongate	11
–	Body usually larger, rounded or elongate; 1^st^ abdominal ventrite with one or two lateral stria, but rarely with both complete – if two present then close together near metacoxa; prescutellar impression varied, but rarely thin and elongate	12
11	Left mandible with small but distinct basal tooth (Fig. 30A); antennal club large, circular; anterior mesoventral emargination deep, nearly meeting arch of mesometaventral stria (Fig. 30C); lateral metaventral stria approximately longitudinal, directed at inner half of metacoxa; pygidium short, wider than long (Fig. 30D); male genitalia generally short, with broad aedeagus bearing strong medioventral tooth (Figs 31–33)	*Operclipygus dubius* group (p. 113)
–	Left mandible usually lacking tooth, or with minute denticle; antennal club smaller, more elongate; mesoventral emargination varied; lateral metaventral stria more oblique, extending toward middle or outer third of metacoxa; pygidium generally longer; male genitalia varied, but never as above	*Operclipygus hospes* group (p. 129)
12	Body small (~2mm), rounded; marginal pygidial sulcus deep, strongly crenulate on inner edge (Figs 15B–D); outer subhumeral stria short, present only apically	*Operclipygus impuncticollis* group (p. 64)
–	Marginal pygidial sulcus rarely strongly crenulate on inner edge (may be absent); other characters varied	13
13	Frons convex, not at all depressed at middle (Fig. 11B); central portion of frontal stria detached from sides, not strongly impressed; inner and outer subhumeral striae complete; pygidium with dense, fine ground punctation (with or without coarser secondary punctures)	*Operclipygus kerga* group (p. 52)
–	Frons at least weakly depressed in middle; other characters variable, but very rarely with both subhumeral striae complete	14
14	Epistoma, prosternal lobe, prosternal keel, mesoventral disk with dense ground punctation (often other body surfaces as well, esp. pronotum, frons, metaventrite; Fig. 13); central portion of frontal stria absent; elytron with fine series of apical punctures	*Operclipygus conquisitus* group (p. 57)
–	Epistoma and prosternal lobe lacking dense ground punctation; central portion of frontal stria usually present (may be detached and/or interrupted); apex of elytra rarely with fine series of apical punctures	15
15	Central portion of anterior pronotal margin outwardly arcuate or angulate	16
–	Central portion of anterior pronotal margin not produced	20
16	Frons very strongly depressed at middle (Figs 28C, E); central portion of anterior pronotal margin strongly angulate at middle; base of prosternal keel weakly emarginate	*Operclipygus impressifrons* group (p. 106)
–	Frons not so strongly depressed at middle; central portion of anterior pronotal margin more weakly angulate or arcuate; base of prosternal keel varied, rarely weakly emarginate	17
17	Frontal stria complete, central portion connected to sides; pygidial sulcus finely impressed, may be abbreviated basally	18
–	Frontal stria interrupted at sides, central portion detached; pygidial sulcus strongly impressed, crenulate	*Operclipygus panamensis* (Wenzel & Dybas)
18	Outer subhumeral stria complete, inner absent; abdominal ventrites 3 and 4 with single series of large deep punctures (Fig. 85G); propygidium with dense medial grouping of small but deep punctures (Fig. 85H), with more or less impunctate band along basal and lateral margins; marginal pygidial sulcus complete	*Operclipygus lama* Mazur
–	Outer subhumeral stria not complete, interrupted at middle or present only in apical half; abdominal ventrites 3 and 4 with more numerous smaller punctures not forming a single series; marginal pygidial sulcus obsolete basally	19
19	Lateral submarginal pronotal stria absent from basal half; outer subhumeral elytral stria present only in apical half; inner subhumeral stria absent; body form strongly narrowed, sides subparallel (Fig. 95C)	*Operclipygus brooksi* sp. n.
–	Lateral submarginal pronotal stria complete along side; outer subhumeral stria reaching base and apex, but interrupted at middle; fragments of inner subhumeral stria generally present; body moderately narrowed, but sides weakly rounded (Fig. 95A)	*Operclipygus peregrinus* sp. n.
20	Coarse punctures of propygidium discretely limited to central part of disk (though fine series may also be present along extreme lateral margin; Figs 95E, 98E); sides of propygidium with only dense, fine ground punctation	21
–	Propygidium with coarser secondary punctures more or less uniformly distributed	23
21	Outer subhumeral stria present only in apical half; body shape distinctly rounded (Fig. 95D)	*Operclipygus profundipygus* sp. n.
–	Outer subhumeral stria complete; body shape subcylindrical, with sides narrow and subparallel (Fig. 98D)	22
22	5^th^ dorsal elytral stria present in apical half and represented by a distinct basal puncture (Fig. 98D); marginal pronotal bead weakly widening toward the front; apex of aedeagus lacking ventrolateral processes	*Operclipygus parallelus* sp. n.
–	5^th^ dorsal elytral stria present in apical half only, lacking basal puncture; marginal pronotal bead widening toward the front; apex of aedeagus with thin, membranous, ventrolateral processes (Fig. 99E)	*Operclipygus siluriformis* sp. n.
23	Elytra coarsely punctate, partially obliterating otherwise mostly complete elytral striae, particularly the apices of 4^th^ and 5^th^ striae (Fig. 85C); ground punctation of pronotum very conspicuous, but coarse secondary pronotal punctures limited to sides; frontal stria complete, transverse; pygidium with fine ground punctation and dense secondary punctation; marginal pygidial sulcus deeply impressed, complete (Fig. 85E)	*Operclipygus punctulatus* sp. n.
–	Elytra not coarsely punctate, except rarely along apical margin; other characters variable	24
24	Outer subhumeral stria complete	25
–	Outer subhumeral stria abbreviated, interrupted, or absent	32
25	Inner subhumeral stria complete	*Operclipygus cavisternus* sp. n.
–	Inner subhumeral stra abbreviated or absent	26
26	Marginal pygidial stria fragmented, abbreviated, or absent	27
–	Marginal pygidial stria complete, well impressed	28
27	4^th^ dorsal elytral stria abbreviated; pygidium with dense ground punctation, lacking coarse secondary punctures (Fig. 101D)	*Operclipygus faltistrius* sp. n.
–	4^th^ dorsal stria complete; pygidium with only sparse ground punctation, with or without secondary punctures	*Operclipygus teapensis* (Marseul)
28	Pronotum with numerous, conspicuous coarse punctures at sides (Fig. 88A); abdominal ventrites 3 and 4 with primary series of large, deep punctures, with few smaller punctures intermingled	*Operclipygus florifaunensis* sp. n.
–	Pronotum lacking coarse lateral punctures; abdominal ventrites 3 and 4 without single dominant series of large deep punctures	.29
29	Elytron with two complete and one partial epipleural striae; pronotal marginal bead wide, convex (Fig. 91A); body larger, ~3.5 mm	*Operclipygus arnaudi* Dégallier
–	Elytron with one complete epipleural stria (often a second, incomplete); pronotal marginal bead varied, weakly convex; body smaller, ~2–3mm	30
30	Elytron with single epipleural stria, with wide smooth epipleuron below; 4^th^ dorsal elytral stria abbreviated from base	31
–	Elytron with one complete upper epipleural stria, and an abbreviated lower stria; 4^th^ dorsal elytral stria complete	*Operclipygus rupicolus* sp. n.
31	Punctures of propygidium small, deep, and rather dense; marginal pygidial sulcus very deep and strongly crenulate (Fig. 101B); South America	*Operclipygus pygidialis* (Lewis)
–	Punctures of propygidium rather large, shallow, and sparse (Fig. 101F); marginal pygidial sulcus deep, but narrower and only weakly crenulate; known from Costa Rica	*Operclipygus limonensis* sp. n.
32	Marginal pygidial sulcus absent (rarely with few weak apical fragments present)	33
–	Marginal pygidial sulcus present, generally well impressed and complete	34
33	Pygidium with dense, fine ground punctation only, lacking any coarser punctures (Fig. 95G); lateral submarginal pronotal stria complete; pronotum lacking coarse lateral punctures; punctures of propygidium small, concentrated in basal two-thirds	*Operclipygus punctatissimus* sp. n.
–	Pygidium with dense, fine ground punctation and markedly coarser punctures uniformly interspersed (Fig. 101H); lateral submarginal pronotal stria obsolete in basal half; pronotum with numerous coarse lateral punctures very close to lateral margin (Fig. 101G)	*Operclipygus wenzeli* sp. n.
34	Lateral submarginal pronotal stria obsolete in basal half	35
–	Lateral submarginal pronotal stria complete along side	36
35	Elytral epipleuron elevated, flat below strong subhumeral swelling, with numerous coarse punctures on side in anterior half (Fig. 92F); outer subhumeral stria present in apical half only	*Operclipygus punctipleurus* sp. n.
–	Elytral epipleuron normally rounded, subhumeral region less strongly swollen, lacking coarse punctures; outer subhumeral stria present in apical half and as isolated basal fragment	*Operclipygus falini* sp. n.
36	Marginal pygidial stria strongly impressed, diverging from margin toward apex (Fig. 91G); pronotum with linear prescutellar impression about equal in length to scutellum	*Operclipygus latipygus* sp. n.
–	Marginal pygidial stria following margin throughout, marginal bead uniform in width; prescutellar impression varied (may be absent)	37
37	Body small, ~1.5 mm, broadly rounded (Fig. 91C); lateral metaventral stria curving laterad toward metepisternum	*Operclipygus subsphaericus* sp. n.
–	Body larger, >2 mm, more distinctly elongate oval; lateral metaventral stria extending posterad toward metacoxa	38
38	Elytron with 4 complete dorsal striae, with only one complete (and possibly additional abbreviated) epipleural stria	40
–	Elytron with 4^th^ dorsal stria abbreviated from base, present in apical half only	39
39	Base of prosternal keel truncate (Fig. 88A); pygidial sulcus fine, complete (Fig. 88D); vestige of inner subhumeral stria generally present; body rounded (Fig. 88C)	*Operclipygus bosquesecus* sp. n.
–	Base of prosternal keel outwardly produced; pygidial sulcus weak, obsolete at base (Fig. 98H); inner subhumeral stria absent; body more elongate (Fig. 98F)	*Operclipygus abbreviatus* sp. n.
40	Body large, ~4 mm, piceous; frontal stria distinctly interrupted above antennal bases (and often also at middle); pygidium with both dense ground punctation and very dense secondary punctation (Fig. 17D)	*Operclipygus crenatus* (Lewis)
–	Body smaller, rufescent; frontal stria complete or very narrowly interrupted over antennal base; pygidium with dense, fine ground punctation with coarser punctures very sparsely intermingled (Fig. 92B)	*Operclipygus elongatus* sp. n.
41	Median pronotal gland openings associated with elaborations of pronotal striae, including anterolateral tubercles and shallow, sinuate channels (Fig. 9); pronotum usually with basal plicae; elytral apices with disorganized punctures, 4^th^ and 5^th^ elytral striae often obscured apically; frons flat to convex, frontal stria complete, transverse; lateral submarginal striae rarely extending inward along anterior margin from corners	*Operclipygus mirabilis*group (males) (p. 39)
–	Median pronotal gland openings never associated with secondary sexual modifications, generally simple in both sexes; elytral apices with or without apical punctures, but 4^th^ and 5^th^ dorsal striae rarely both abbreviated apically	42
42	Pronotal plicae present; head disproportionately large (Fig. 62C); mandibles prolonged at apices (Figs 62E–G)	*Operclipygus plicicollis* group (p. 249)
–	Pronotal plicae absent	43
43	Body large (>7mm), strongly convex; legs broadly expanded, the meso- and metathoracic legs about two-thirds as wide as long (Fig. 80E); only known from Minas Gerais, Brazil	*Operclipygus formicatus* sp. n.
–	Body smaller, convex or not; legs elongate, no more than one-third as wide as long	44
44	Median pronotal gland openings obscured by dense pronotal punctation (Figs 24A, D, E); prosternal lobe elevated along midline (Fig. 24B); body generally conspicuously and completely punctate, depressed, elongate; lateral submarginal pronotal stria close to margin, subcarinate, continuous with anterior submarginal stria	*Operclipygus dytiscoides* group (p. 96)
–	Median pronotal gland openings distinct, situated one-third or more pronotal length behind anterior margin; prosternal lobe not elevated along midline; other characters variable	45
45	Median pronotal gland openings at or just in front of pronotal midline (Figs 54B, D); ventral surface of tarsomeres with dense brush of setae, not in organized lateral series (Fig. 54A); 9^th^ tergite of male genitalia with apices dorsolaterally flattened, divergent (Fig. 55C)	*Operclipygus hirsutipes* group (p. 213)
–	Median pronotal gland openings behind pronotal midline; ventral surface of tarsi with setae organized in distinct lateral rows; 9^th^ tergite of male never with apices flattened and divergent	46
46	Mandibles strongly dentate (Fig. 45G); epistoma strongly convex; body elongate, parallel sided, subdepressed (Fig. 45F); anterior submarginal pronotal stria detached, strongly recurved posterad; median pronotal gland openings about two-thirds behind pronotal margin	*Operclipygus bulbistoma* sp. n.
–	Left mandible with at most a very weak tooth; epistoma flat to subdepressed; other characters varied	47
47	Base of prosternal keel weakly emarginate; pygidial sulcus weak, never complete and rarely absent; microsculpture generally present on propygidium (Fig. 6F, 56A), often also on pygidium, metaventrite, and 1^st^ abdominal ventrite; anterior submarginal pronotal stria frequently detached from lateral stria (Fig. 56C); pygidium never with dense ground punctation; elytra always with 4 or more complete dorsal striae; body usually rufescent (Fig. 56D), rarely darker	*Operclipygus hamistrius* group (p. 218)
–	Base of prosternal keel subtruncate to posteriorly arcuate, rarely emarginate; pygidial sulcus varied from absent to strong, rarely weak and abbreviated; propygidium lacking microsculpture (microsculpture may be present on parts of the venter); anterior and lateral submarginal pronotal striae continuous across anterior margin, anterior portion rarely detached; pygidium frequently with dense, fine ground punctation; elytral striation frequently reduced; body color darker, generally rufopiceous	48
48	Anterior submarginal pronotal stria narrowly detached from lateral, barely recurved posterad at sides (Fig. 105A); anterior pronotal margin projecting at middle, with marginal stria complete along anterior edge; posterior half of lateral pronotal margin bent ventrad, nearly vertical; propygidium with dense ground punctation at sides, with coarse punctures more or less restricted to middle of propygidium; marginal pygidial sulcus complete, but not ending in basal foveae (Fig. 105C)	*Operclipygus angustisternus* (Wenzel & Dybas)
–	If anterior submarginal pronotal stria detached from lateral stria, then anterior pronotal margin not projecting; other characters varied	49
49	Outer subhumeral stria complete or interrupted at middle, present in basal and apical halves; pygidial marginal sulcus usually deep and coarse, often ending in basal foveae, though sulcus and/or foveae may be lacking	50
–	Outer subhumeral stria present in apical half only or absent; pygidial sulcus fine, without basal pygidial foveae	*Operclipygus impunctipennis* group (p. 277)
50	Pygidium with deep marginal sulcus widened in basal half, but not abruptly enlarged (Fig. 105E), basolateral foveae absent; 3^rd^ dorsal elytral stria interrupted; aedeagus narrow and elongate, not short and broad, with very weak medioventral process; known only from Venezuela	*Operclipygus shorti* sp. n.
–	Pygidium with or without marginal sulcus, but sulcus never gradually widened in basal half; basolateral pygidial foveae present or absent; 3^rd^ dorsal elytral stria complete; aedeagus short and broad (e.g., Fig. 65G), with strong medioventral process; median lobe with large gonopore frequently exposed beneath	*Operclipygus fossipygus* group (p. 256)

### *Operclipygus sulcistrius* group

**Figure 7.**
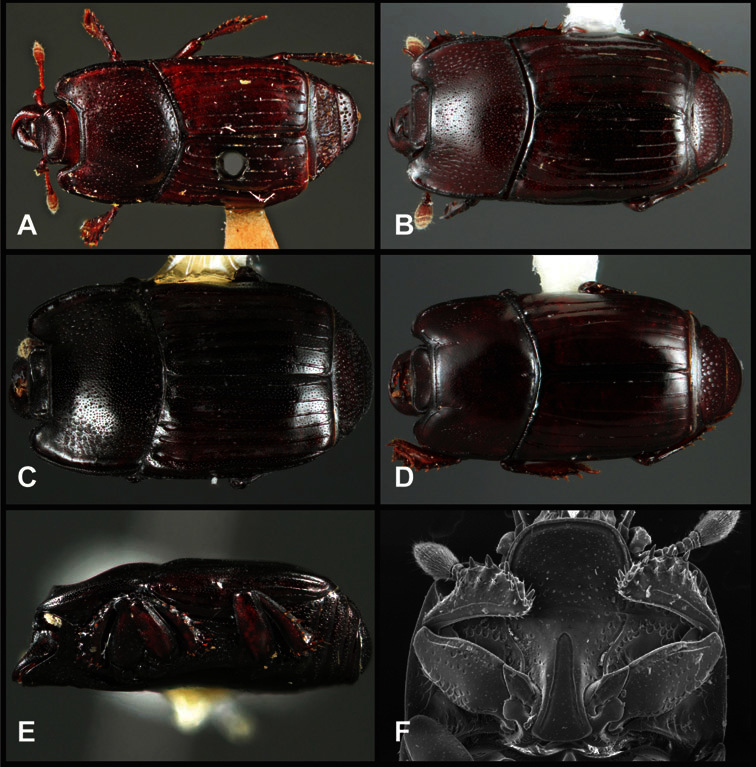
*Operclipygus sulcistrius* group. **A** Dorsal habitus of *Operclipygus sulcistrius* (lectotype) **B** Dorsal habitus of *Operclipygus schmidti* **C** Dorsal habitus of *Operclipygus lucanoides* **D** Dorsal habitus of *Operclipygus simplistrius* **E** Lateral habitus of *Operclipygus lucanoides* **F** Prosternum of *Operclipygus schmidti*.

The *Operclipygus sulcistrius* group includes 4 very similar species, which would be considered atypical if it weren’t for the somewhat unfortunate fact that *Operclipygus sulcistrius* is the type of the genus. These species may be recognized by the following shared characters: elongate, parallel-sided, and subdepressed body form (Fig. 7), with moderately or markedly exaggerated body sculpturing, particularly noticeable in the pronotal striae; anterior submarginal pronotal stria recurved onto the pronotal disk for varying distance; median pronotal glands about 5 puncture widths from the anterior margin; strong, unusually protracted mandibles, both of which bear a strong mesal tooth (Fig. 4E); enlarged, apically subtruncate prosternal lobe (Fig. 7F); prosternal keel projecting deeply into a mesoventral emargination; mesometaventral stria arched strongly forward, almost completely displacing marginal mesoventral stria; lateral metaventral stria doubled; abdominal ventrite 1 with two complete lateral striae; pygidium with complete, deep marginal sulcus. Male genitalia (Fig. 3) with accessory sclerites present; T8 elongate, with deep basal emargination and tangential basal membrane attachment line; ventrolateral apodemes of T8 most strongly developed at base, separated beneath; S8 with apical guides evenly developed from base to apex, ventral halves separate, approximate over entire length; T9 with apices broad, weakly subtruncate; T10 elongate, halves fused in basal one-fifth; S9 rather broad, truncate to subemarginate at base, lacking apical emargination, with thin, continuous apical flange; aedeagus broadly rounded at sides evenly tapered to apex, with moderately strong ‘U’-shaped medioventral process; median lobe about one-third tegmen length.

The four species of this group are known from a total of ten specimens, from localities scattered through much of tropical South and Central America, so are extremely rare. Nothing is known of their biology.

### Key to the species of the *Operclipygus sulcistrius* group

**Table:** 

1	5^th^ dorsal elytral stria complete	2
–	5^th^ dorsal stria abbreviated from base	*Operclipygus simplistrius* sp. n.
2	5^th^ dorsal and sutural elytral striae united in basal arch	3
–	5^th^ dorsal and sutural elytral striae free anteriorly	*Operclipygus schmidti* sp. n.
3	Anterior pronotal margin strongly elevated behind head; supraorbital stria interrupted at middle; inner subhumeral stria present in apical half	*Operclipygus lucanoides* sp. n.
–	Anterior pronotal margin weakly elevated behind head; supraorbital stria complete; inner subhumeral stria absent	*Operclipygus sulcistrius* Marseul

### Operclipygus sulcistrius

Marseul, 1870

http://species-id.net/wiki/Operclipygus_sulcistrius

Figs 3 7A

Operclipygus sulcistrius Marseul, 1870: 75.

#### Type locality.

Unspecified, somewhere along the Amazon in Brazil.

#### Type material.

**Lectotype**, here designated (MNHN): original handwritten green disk label barely legible: ?”*Operclipygus sulcistrius* Amazons Bates 69” [type locality as published is “Fleuve des Amazones”] / “TYPE” / MUSEUM PARIS COLL. DE MARSEUL 2842-90” / “LECTOTYPE *Operclipygus sulcistrius* Marseul 1870, M.S.Caterino & A.K.Tishechkin des. 2010”. This species was described from an unspecified number of specimens, and the lectotype designation fixes primary type status on the only known specimen.

#### Diagnostic description.

Length: 2.59 mm, width: 1.68 mm; body rufescent; frontal stria strongly impressed, complete to sides and continuous with supraorbital. Lateral pronotal stria complete, close to margin and carinate, continuous to front through strongly depressed anterior corners, meeting anterior portion of anterior submarginal stria, which is recurved about 1/4 the length of the pronotal disk; pronotal disk moderately convex, punctate throughout, with numerous larger punctures toward sides. Elytra with outer subhumeral and dorsal striae 1-5 complete, strongly impressed, stria 5 attached to sutural stria by basal arch, only the inner subhumeral stria absent. Prosternum with striae meeting in a narrow arch anteriorly, ending well behind presternal suture. Propygidium flat, with many close large, elongate punctures; pygidium with only a few large punctures along basal margin, finely but distinctly punctate elsewhere; apical sulcus well impressed and crenulate around apical margin. Male genitalia as described for the species group (Fig. 3).

#### Distribution.

This species is only known from the type specimen, from somewhere along the Amazon River (‘fleuve’).

#### Remarks.

This species can be separated from the others in this group by its basally connected 5^th^ and sutural striae (shared with *Operclipygus lucanoides*) in combination with the relatively weakly elevated anterior pronotal margin, complete absence of the inner subhumeral striae, and relatively simple frons bordered by a complete supraorbital stria.

### Operclipygus schmidti sp. n.

urn:lsid:zoobank.org:act:DD066ADA-63FB-49A8-AAD1-B1BC78F921E8

http://species-id.net/wiki/Operclipygus_schmidti

Figs 4E 7B Map 1

#### Type locality.

PANAMA: Colón: San Lorenzo Forest [9°17'N, 79°58'W].

#### Type material.

**Holotype male**: “**PANAMA**: **Colón** Pr., San Lorenzo Forest. 9°17'N, 79°58'W. Flight Intercept FIT-B2-16. 21–24 May 2004 A. Tishechkin. AT-490” / “LSAM 0111233” (FMNH). **Paratypes** (3): locality, collector and methods as for holotype, collected 13–14.v, 18–19.v, and 20–21.v, all 2004 (GBFM, AKTC).

#### Diagnostic description.

This species is very similar to *Operclipygus sulcistrius* as described above. It differs in the following characters: length: 2.25–2.31 mm, width: 1.47–1.56 mm; body smaller, rufo-brunneus; supraorbital stria absent; anterior submarginal stria recurved for a shorter distance along pronotal disk, about one-eighth total length; elytral striae less strongly impressed, the 5^th^ and sutural striae not joined by a basal arch; inner subhumeral stria vaguely traceable by a series of punctures in apical half; propygidial punctures smaller and sparser, only slightly elongate, separated by their widths or more; pygidium with numerous larger punctures evenly scattered among the fine, conspicuous ground punctation. Male genitalia indistinguishable from those of *Operclipygus sulcistrius* (Fig. 3).

#### Remarks.

Among members of the *Operclipygus sulcistrius* group, *Operclipygus schmidti* is distinct in having the 5^th^ and sutural elytral striae complete but not united by a basal arch. It is also unusual in lacking a supraorbital stria.

**Map 1.**
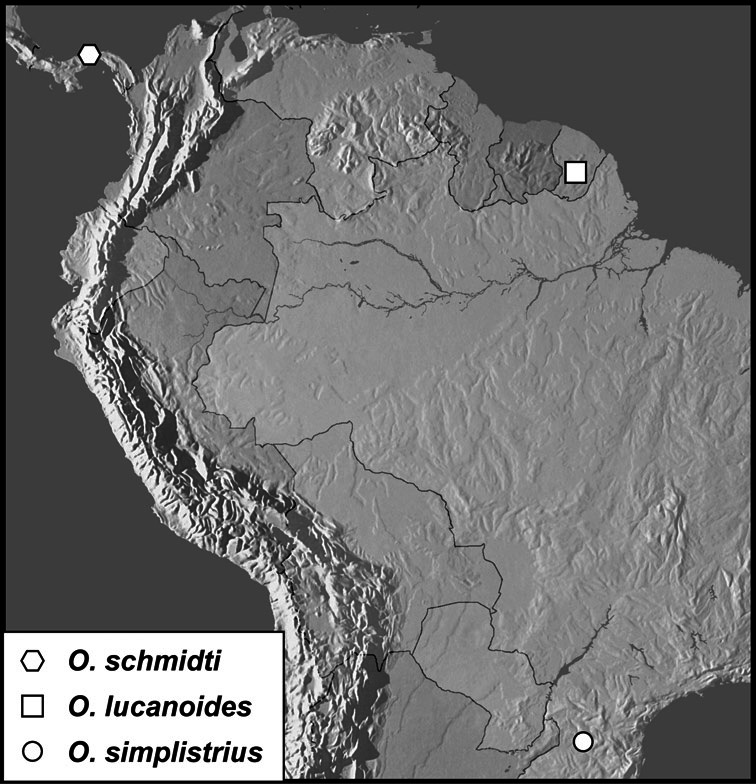
Records of the *Operclipygus sulcistrius* group.

#### Etymology.

We name this species in honor of Johannes Schmidt, the great 19^th^ century German histerid specialist, describer of numerous species of Neotropical Exosternini.

### Operclipygus lucanoides sp. n.

urn:lsid:zoobank.org:act:FE39681E-D89A-4516-B3B7-90CFFE00DC17

http://species-id.net/wiki/Operclipygus_lucanoides

Figs 7C 7E Map 1

#### Type locality.

FRENCH GUIANA:Itoupé Table Mountain [3°1.38'N, 53°5.73'W].

#### Type material.

**Holotype male**: “**GUYANE FR.,** Mont tabularie Itoupé. 3°1.38'N, 53°5.73'W. 570 m. Piège d’interception, 17 Mar 2010. SEAG leg.” / “Caterino/Tishechkin Exosternini Voucher EXO-00269” (MNHN). **Paratypes** (4): **FRENCH GUIANA:** 1: Rés. des Nouragues, Camp Inselberg, 20.viii.2010 (CHND), 1: 28.i.2010; 1: Rés. des Nouragues, Saut Pararé, 4°02'N, 52°41'W, 20.iv.2010 (MNHN); 1: Belvèdére de Saül, 3°1'22"N, 53°12'34"W, 30.ix.2010 (FMNH).

#### Other material.

**SURINAME: Sipaliwini**, W bank Suriname R., nr. Jungle Resort Pingpe, 4°02'30"N, 55°27'00"W, 1–11.ii.2012, FIT, A. Hielkema (RMNH).

#### Diagnostic description.

This species has the most strongly exaggerated sculputuring in the group, but otherwise is very similar to both of the above. It differs in the following characters: length: 2.65–2.68 mm, width: 1.81–1.83 mm; body piceous; frontal stria carinate, continuous with supraorbital stria, which is interrupted medially; pronotal disk strongly depressed along inner edges of lateral and anterior submarginal striae, marginal bead being strongly elevated and subcarinate; anterior submarginal pronotal stria recurved about one-fourth pronotal length (although varied in type material); elytra with inner subhumeral stria present in apical half or more, with the interval between it and dorsal stria 1 strongly elevated; dorsal striae 1-5 and sutural complete, 5^th^ and sutural striae often joined by basal arch; prosternal striae nearly reaching presternal suture anteriorly, joined in a broad arch; propygidium depressed on either side (and correspondingly elevated along lateral edges), punctures large, only slightly elongate, separated by less than half their widths; pygidium as in *Operclipygus schmidti*, with larger punctures sparsely scattered amongst fine, dense ground punctation. Male genitalia indistinguishable from those of *Operclipygus sulcistrius* (Fig. 3).

#### Remarks.

The strongly exaggerated dorsal sculpturing, especially the elevated anterior pronotal margin (Fig. 7E) distinguishes this species rather unambiguously. It is also unique in the group in having the strongly impressed supraorbital stria interrupted at the middle, and in having the apical half of the inner subhumeral stria present along the outer edge of the subhumeral elytral swelling.

#### Etymology.

This species’ name refers to its exaggerated, subporrect mandibles, reminiscent of a small stag beetle.

### Operclipygus simplistrius sp. n.

urn:lsid:zoobank.org:act:0D5A3C6B-9F61-4A69-8B91-F98090FBD8AE

http://species-id.net/wiki/Operclipygus_simplistrius

Fig. 7D Map 1

#### Type locality.

BRAZIL: Santa Catarina: Nova Teutonia [27°11'S, 52°23'W].

#### Type material.

**Holotype female**: “Nova Teutonia, Sta. Catharina, BRAZ. I:14:1958 Fritz Plaumann leg.” / “FMNH-INS 0000 069 297” / “♀” (FMNH).

#### Diagnostic description.

While this species shares most of the characters of the group, it is relatively unmodified in terms of sculpturing. Pronotal and elytral striae are all relatively shallowly depressed, with little exaggeration of interstrial intervals. This brief description will distinguish it from other species: length: 2.43 mm, width: 1.53 mm; body rufo-brunneus; anterior submarginal pronotal stria recurved slightly more than one-fourth pronotal disk length; lateral submarginal pronotal stria slightly abbreviated at base; pronotal disk with short, sublinear prescutellar impression, disk relatively impunctate with only small elongate group of punctures near sides, not at all depressed along inner edges of submarginal striae; elytra with outer subhumeral stria present only in apical half, inner subhumeral stria absent, dorsal striae 1-4 complete, 5^th^ present in apical third, sutural very slightly abbreviated at base; prosternal striae nearly reaching presternal suture anteriorly, joined in a broad arch; propygidium evenly convex with large punctures separated by about their widths at the base becoming smaller and more widely separated apically; pygidium with fine ground punctation throughout, with larger scattered punctures confined to basal third. Male: unknown.

#### Remarks.

This species is unique in the group in having rather simple pronotal striation, without any depression in the anterolateral corners, and in having the outer subhumeral and 5^th^ dorsal elytral striae abbreviated from base.

#### Etymology. 

This species is named for its relatively simple sculpturing and striation compared with other members of the group.

##### *Operclipygus mirabilis* group

**Figure 8.**
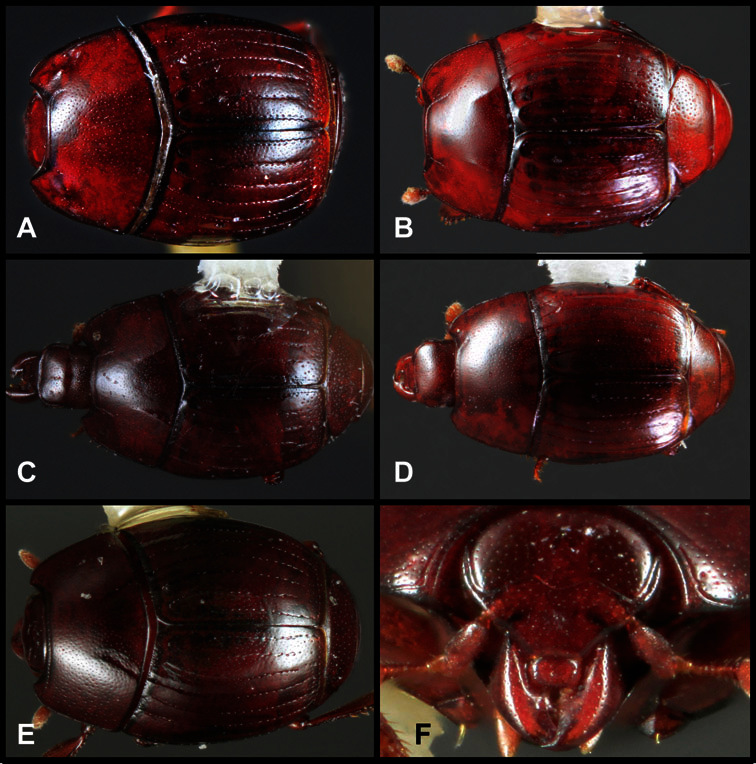
*Operclipygus mirabilis* group. **A** Dorsal habitus of *Operclipygus mirabilis* **B** Dorsal habitus of *Operclipygus mutuca* **C** Dorsal habitus of *Operclipygus carinistrius* (lectotype) **D** Dorsal habitus of *Operclipygus parensis* **E** Dorsal habitus of *Operclipygus schlingeri* **F** Frons of *Operclipygus schlingeri*.

**Figure 9.**
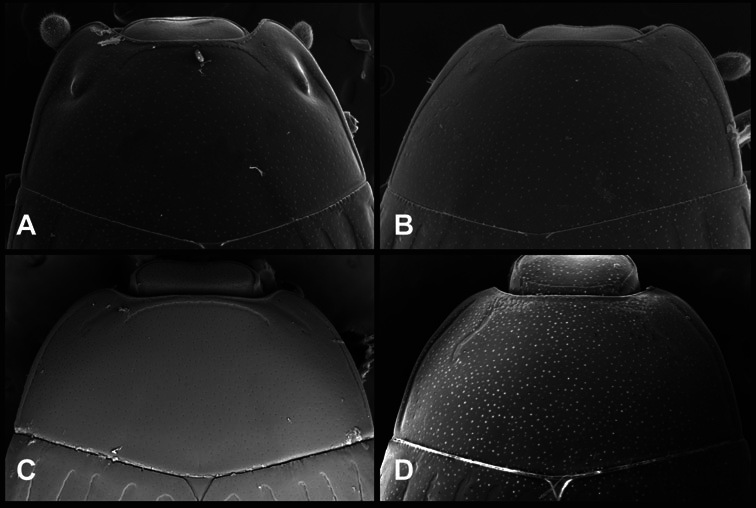
*Operclipygus mirabilis* group. **A** Pronotum of *Operclipygus mirabilis* **B** Pronotum of *Operclipygus pustulifer* **C **Pronotum of *Operclipygus plaumanni* **D** Pronotum of *Operclipygus sinuatus*.

**Figure 10.**
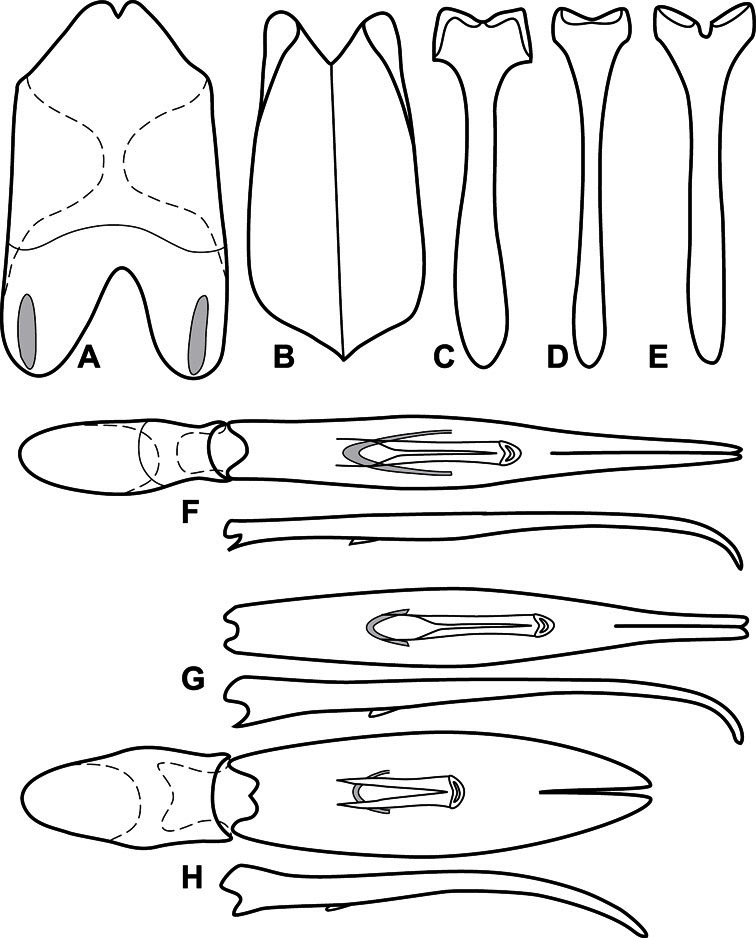
Male genitalia of *Operclipygus mirabilis* group. **A** T8 of *Operclipygus mirabilis* **B** S8 of *Operclipygus mirabilis* **C** S9 of *Operclipygus mirabilis* **D** S9 of *Operclipygus sinuatus* **E** S9 of *Operclipygus schlingeri* **F** Aedeagus, dorsal and lateral views, of *Operclipygus mirabilis* **G **Aedeagus, dorsal and lateral views, of *Operclipygus sinuatus* **H** Aedeagus, dorsal and lateral views, of *Operclipygus schlingeri*.

The *Operclipygus mirabilis* group includes eight species, most of which exhibit a unique sexual dimorphism in which the median pair of pronotal discal glands differs in placement and elaboration between the sexes. The name of the only described species is based on the male condition, which is quite remarkable; in males of this species the median gland opening is easily visible atop a pronounced tubercle, which itself sits within a moderately broad anterolateral pronotal depression (Fig. 9A). This is in fact the most pronounced of these dimorphisms present in known species, but most of the species in the group exhibit comparable conditions to varying degrees (Figs 9B–D). Other external characters shared by these species include: frons generally broad, flat to moderately convex; anterior part of frontal stria generally transverse, complete, not sinuate over antennal bases (with one notable exception, *Operclipygus schlingeri*, where the frontal stria is abruptly bent at the sides and strongly sinuate across the front); lateral submarginal pronotal striae not extending far inward along anterior margin, generally ending at anterolateral corner; pronotal plicae present; apical elytral punctures present, generally associated with apical disruption and abbreviation of the 4^th^ and 5^th^ elytral striae (e.g. Fig. 8C). The aedeagus of most of these species is distinctly ‘hooked’ apically, with the tip abruptly curved (Fig. 10F–H), moreso than in nearly any other *Operclipygus*. Two of the species we place here are known from females only, but other characters strongly support their assignments here. The real outlier is *Operclipygus schlingeri*, in which only a single pair of pronotal glands is apparent in the female, and those of the male are not visible dorsally. It appears that they have been displaced beneath the lateral pronotal margin, through a visible and unique anterolateral groove, onto the hypomeron, but it is very difficult to see a distinct opening at the terminus of this groove, and it is not easily visible in any of the available specimens. The aedeagus of *Operclipygus schlingeri* also does not conform well with the general diagnosis above (Fig. 10H). However, in most other characters it seems to fit well here.

### Key to the species of the *Operclipygus mirabilis* group

**Table:** 

1	Inner subhumeral stria present; pronotal plicae present, strong	2
–	Inner subhumeral stria absent; pronotal plicae present or absent	5
2	Secondary, outer lateral metaventral stria present in addition to inner	3
–	Only a single lateral metaventral stria present	4
3	Marginal pygidial stria present, distinct	*Operclipygus carinistrius* (Lewis)
–	Marginal pygidial stria absent	*Operclipygus mutuca* sp. n.
4	Male median pronotal gland opening borne on tubercle surrounded by shallow depression in anterolateral pronotal corner, depression displacing anterior half of lateral submarginal pronotal stria (Fig. 9A); female with numerous coarse punctures along sides of pronotum and along sides of pygidium	*Operclipygus mirabilis* (Wenzel & Dybas)
–	Male median pronotal gland opening borne on minute tubercle but without surrounding depression (Fig. 9B); lateral submarginal pronotal stria complete; female with lateral pronotal and pygidal punctures finer to inconspicuous	*Operclipygus pustulifer* sp. n.
5	Pygidial stria absent; propygidium with only very few punctures; male median pronotal gland opening borne on minute tubercle laterad apices of recurved anterior submarginal stria (Fig. 8D); northeastern Brazil	*Operclipygus parensis* sp. n.
–	Pygidial stria present;male median pronotal gland position varied	6
6	Frontal stria abruptly bent near antennal bases, strongly sinuate across front (Fig. 8F); marginal pygidial sulcus deep (though may be interrupted); male median pronotal gland openings not visible dorsally	*Operclipygus schlingeri* sp. n.
–	Frontal stria more or less transverse, at most weakly sinuate at sides; male median pronotal gland opening found at end of sinuate depression extending posterolaterad from anterior margin	7
7	Male median pronotal gland opening found at posterior end of shallow, sinuate impression extending posterad from granulate depression along anterior pronotal margin (Fig. 9D); anterior submarginal pronotal stria absent; marginal pygidial sulcus distinct, though fine and often obsolete basally; pronotum with numerous coarse punctures along lateral margin; Central America (Panama)	*Operclipygus sinuatus* sp. n.
–	Male median pronotal gland opening found at end of short impression extending obliquely from anterior pronotal margin, depression smooth, not granulate (Fig. 9C); anterior submarginal stria present, recurved parallel to depression; marginal pygidial sulcus very faint; pronotum lacking coarse lateral punctures; southeastern Brazil	*Operclipygus plaumanni* sp. n.

### Operclipygus mirabilis

(Wenzel & Dybas, 1941) comb. n.

http://species-id.net/wiki/Operclipygus_mirabilis

**Map 2.**
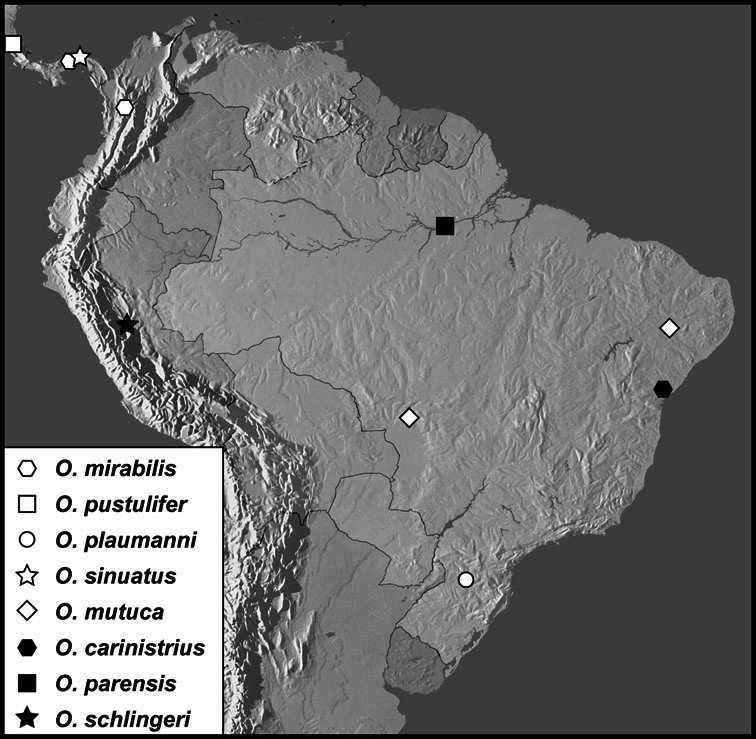
Records of the *Operclipygus mirabilis* group.

Figs 8A 9A 10A–C, F Map 2

Pseudister mirabilis Wenzel & Dybas, 1941: 454.

#### Type locality.

COLOMBIA: Cundinamarca: Puerto Salgar [5°28'N, 74°39'W].

#### Type material.

**Holotype male**: “P’to. Salgar, Cund Colomb, VII:31:38” / “Coll. by C.H.Seevers” / “Collection R. L. Wenzel” / “Type *Pseudister mirabilis* Wenzel & Dybas” (FMNH).

#### Other material.

**PANAMA, Panamá**: 1: Barro Colorado Isl., 09°11'N, 79°51'W, 8.vii.1994, D. Banks, FIT (SEMC), 1: external refuse deposit *Atta colombica* (Guérin-Méneville), 11.ii.1976 (FMNH); **Colón**: 1: Parque Nac. San Lorenzo, Achiote, 9°12'N, 79°59'W, 10m, 8–22.v.2007, FIT, A. Mercado (FMNH); 1: 50m [from forest edge, as for all A. Mercado specimens; pers. comm.], 9–23.v.2007(GBFM); 1: San Lorenzo Forest, STRI crane site, 9°17'N, 79°58'W, 25–26.v.2003, FIT, A.K. Tishechkin (GBFM), 1: ex. refuse pile *Atta colombica*, 25.x.2003 (AKTC), 1: FIT, 11–12.v.2004 (LSAM).

#### Diagnostic description.

Length: 1.90–1.97 mm, width: 1.50–1.68 mm; Body rufo-brunneous, sides rounded, moderately convex; lower part of frons and epistoma weakly depressed at middle, frontal stria outwardly arcuate, faintly sinuate at middle, rarely interrupted at sides; supraorbital stria absent; labrum rather narrow, about 1.5× as wide as long, shallowly emarginate at apex; left mandible untoothed, right mandible with very weak basal incisor tooth; pronotum with strong plicae in basal half, in front of 3^rd^ elytral stria, lateral submarginal pronotal stria complete in female, in male ending one-third behind anterior corner in anterolateral pronotal depression; anterior submarginal stria detached, with ends recurved about one-sixth pronotal length; anterior marginal pronotal stra narrowly interrupted behind head; male with depression in anterolateral corner with central tubercle bearing median pronotal gland opening at its apex; female with median pair of gland openings just laterad ends of recurved anterior submarginal stria, about 8 diameters from anterior margin; pronotal disk with few coarse punctures intermingled with fine ground punctures toward sides; elytra with two complete epipleural striae, outer subhumeral stria briefly interrupted at midpoint, otherwise complete, inner subhumeral stria present, obsolete at anterior and posterior ends, all other dorsal elytral striae complete, 5^th^ and sutural striae connected by an angulate basal arch; few coarse punctures present near elytral apex; prosternum with carinal striae complete, sinuate, close at midpoint, diverging slightly to front, connected by narrow anterior arch, keel with faint secondary carinal striae laterad primary striae; prosternal keel weakly produced posteriorly; anterior mesoventral margin broadly emarginate in continuous arc from corner to corner, marginal mesoventral stria complete, mesometaventral stria arched forward to middle of mesoventral disk, crenulate, extending posterad to middle of each metacoxa; 1^st^ abdominal ventrite with two lateral striae, outer stria abbreviated posteriorly; propygidium with sparse fine ground punctation and coarser punctures scattered, separated by about 2× their diameters; pygidium with similar ground punctation, and very few coarser punctures, particularly near anterolateral corners; pygidial marginal stria absent. Male genitalia (Figs 10A–C, F): accessory sclerites present; T8 with deep, rather narrow basal emargination with well sclerotized basal edge, basal membrane attachment line not intersecting basal emargination, apical emargination shallow, ventrolateral apodemes not meeting at midline; S8 narrowing weakly to apex, with apical guides developed only at apex, ventral halves meeting along most of midline but with median area markedly desclerotized; T9 with apices simple, pointed inward; T10 with halves fully separate; base of S9 widened gradually in basal two-thirds, lacking apical emargination, apical flanges separate, apicolateral flanges well developed; tegmen widest basad midpoint, more strongly narrowed to apex, apex strongly bent ventrad, medioventral process ‘U’-shaped, projecting beneath about one-fourth from base; median lobe about one-third tegmen length, proximal apodemes uniform; basal piece about one-third tegmen length.

#### Remarks.

The nominate species in this group is the most easily recognized, at least based on males. The distinctive depression bearing the median pronotal gland opening on a small tubercle (Fig. 9A) identifies them instantly. Males of the following species, *Operclipygus pustulifer*, have a smaller tubercle and no surrounding depression (Fig. 9B). Both of these species have the 5^th^ and sutural elytral striae complete and connected by a basal arch, and also have the inner subhumeral stria more or less complete. The females are more difficult to identify, and are extremely similar to those of *Operclipygus pustulifer*. Those of *Operclipygus mirabilis*, however,have more conspicuously coarse punctures along the sides of the pronotum and the pygidium.

The holotype was reportedly collected in a ‘rubbish heap of *Atta* sp.’ (Wenzel and Dybas 1941), and several recent records confirm this association, specifically with *Atta colombica*.

### Operclipygus pustulifer sp. n.

urn:lsid:zoobank.org:act:1423C21B-1A81-487B-97D4-EFA7A03E9863

http://species-id.net/wiki/Operclipygus_pustulifer

Fig. 9B Map 2

#### Type locality.

COSTA RICA: Guanacaste: 9 km S Santa Cecilia, Pitilla Research Station [10°59.3'N, 85°25.5'W].

**T**

#### ype material.

**Holotype male**: “Est. Pitilla, 9km S. Sta. Cecilia, P. N. Guanacaste, Prov. Guana, COSTA RICA. 700 m Ene [January] 1994, C. Moraga, L N 330200_380200 #2563” (INBIO). **Paratypes** (18): 17: same data as holotype (INBIO, FMNH, AKTC, MSCC); 1: same data as holotype, but iii.1994 (INBIO).

#### Diagnostic description.

This species is very similar in most characters to *Operclipygus mirabilis* and is only described to the extent that it differs from it. Length: 1.72–1.75 mm, width: 1.40–1.44 mm; male with lateral submarginal pronotal stria diverging from marginal in anterior two-thirds, ending behind anterior corner, median pair of pronotal gland openings borne on very weak tubercles; pronotal disk not depressed anteriorly around tubercles; lateral submarginal pronotal stria of female situated slightly further from margin, particularly anteriorly, than that of *Operclipygus mirabilis*. Male genitalia with apical guides of S8 slightly narrower apically than in *Operclipygus mirabilis*, S9 also slightly narrower and more elongate; aedeagus essentially indistinguishable.

#### Remarks.

This species and *Operclipygus mirabilis* are very similar, differing mainly in the exaggeration of the tubercle of the male pronotum, which in *Operclipygus pustulifer* lacks any surrounding depression (Fig. 9B).

#### Etymology.

This species’ name refers to the raised gland opening of the male pronotum.

### Operclipygus plaumanni sp. n.

urn:lsid:zoobank.org:act:22AEBC13-20AD-4AA3-94F9-9265C040C350

http://species-id.net/wiki/Operclipygus_plaumanni

Fig. 9C Map 2

#### Type locality.

BRAZIL: Santa Catarina: Nova Teutonia [27°11'S, 52°23'W].

#### Type material.

**Holotype male**: “Nova Teutonia, Sta. Catharina, BRAZ. X:16:52, Fritz Plaumann leg.” / “bei *Acromyrmex*” / “FMNH-INS0000069312” (FMNH). **Paratypes** (25): all from same locality, dates as follows: 3: 3.vi.1941, 13: 3.i.1949, 1: 4.x.1952, 1: 5.x.1952, 1: 9.x.1952, 1: 10.x.1952, 1: 13.x.1952, 2: 21.ix.1952, 1: 27.x.1952, 1: no date (FMNH, UFPR, MSCC, AKTC).

#### Diagnostic description.

This species is very similar in most characters to *Operclipygus mirabilis* and is only described to the extent that it differs from the above. Length: 1.62–1.93 mm, width: 1.37–1.65 mm; pronotum with well developed basal plicae; anterior submarginal pronotal stria present, recurved with ends divergent; anterior marginal stria complete or barely interrupted at middle; male with divergent, sublinear depression on each side laterad and parallel to divergent arm of anterior submarginal stria, these depressions bearing median pronotal gland openings near their posterior-most ends; lateral submarginal pronotal stria similarly complete in both sexes; elytron lacking inner subhumeral stria, sutural stria obsolete in basal third, not meeting basal arch of 5^th^ stria; pygidium with marginal stria faintly impressed along apical third of margin, rarely obsolete. Male with apical guides of S8 slightly narrower apically than in *Operclipygus mirabilis*; aedeagus more strongly hooked at apex.

#### Remarks.

The males of *Operclipygus plaumanni* and *Operclipygus sinuatus* have unique pronotal modifications, with the median pair of gland openings born at the end of a shallow, sinuate channel extending posterad or posterolaterad from anterior pronotal margin. In *Operclipygus plaumanni* this channel is shorter and extends more laterally than posteriorly (Fig. 9C). Its marginal pygidial sulcus is also very faint, barely detectable around the apex.

#### Etymology.

The species is named for famed German collector Fritz Plaumann (1902–1994), who made his home in Nova Teutonia, Santa Catarina.

### Operclipygus sinuatus sp. n.

urn:lsid:zoobank.org:act:72D6D852-F037-4958-B2B4-EC53419BB13B

http://species-id.net/wiki/Operclipygus_sinuatus

Figs 9D 10D, G Map 2

#### Type locality.

PANAMA: Colón: San Lorenzo Forest [9°17'N, 79°58'W].

#### Type material.

**Holotype male**: “**PANAMA**: Colón Pr., San Lorenzo Forest. 9°17'N, 79°58'W. Flight Intercept FIT-I3-13, 13–14 May 2004 A. Tishechkin. IBISCA ‘04”, FMNH. **Paratypes**: (9): 2: same data as type, except as noted: 1: 17–18.v.2004, 1: 21–24.v.2004 (AKTC, FMNH, GBFM, MSCC); **PANAMA**: **Colón:** 2: Parque Nac. San Lorenzo, Achiote, Cafetal B, 9°12'N, 79°59'W, 50m, 8–22.v.2007, FIT, A. Mercado (GBFM, LSAM); 2: Parque Nac. San Lorenzo, Achiote, Cafetal C, 9°11'N, 79°58'W, 100m, 9–23.v.2007, FIT, A. Mercado (AKTC); 1: Parque Nac. San Lorenzo, Achiote, Cafetal A, 9°12'N, 79°58'W, 100m, 26.vi–10.vii.2007, FIT, A. Mercado (AKTC), 1: 250m, 7–21.v.2007 (AKTC); 1: Barro Colorado Isl., iii.2001, host *Eciton burchelli* (Westwood), C. & M. Rettenmeyer (UCONN).

#### Diagnostic description.

This species is very similar in most characters to *Operclipygus mirabilis* and is only described to the extent that it differs. Length: 1.90–2.15 mm, width: 1.59–1.78 mm; head with supraorbital stria weakly impressed, barely detached from frontal stria at sides; pronotal disk depressed at base, but basal plicae weak to indistinct; anterior submarginal pronotal stria absent, anterior marginal stria complete behind head; male with granulate sculpturing along anterior margin of pronotum, with divergent, sinuate, elongate depressions extending posterad leading to the median pronotal gland openings halfway to posterior margin; coarse lateral discal punctures of pronotum few to absent; female with more distinct lateral coarse punctures, a few fragments of anterior submarginal pronotal stria frequntly visible at sides, and with median gland openings about one-fifth from anterior margin, with very shallow, indistinct impression anterior of them; elytra with inner subhumeral stria absent, sutural stria obsolete in basal third, not meeting basal arch of 5^th^ stria; apical punctures of elytral disk more numerous and diffuse; prosternal carinal striae variably shortened anteriorly, especially in males, meeting in a narrow arch just anterad midpoint of keel; base of keel truncate, not at all projecting posterad, anterior margin of mesoventrite correspondingly truncate, or even very weakly projecting at middle; mesometaventral stria more angulate than rounded at middle, reaching basal third of mesoventral disk; metaventral disk with secondary, outer lateral stria parallel to lateral stria in its basal half; pygidium with ground punctation very conspicuous, marginal pygidial stria present, obsolete in basal corners. Male genitalia (Figs 10D, G) with apical guides of S8 slightly narrower apically than in *Operclipygus mirabilis*, aedeagus more strongly hooked at apex.

#### Remarks.

Males of *Operclipygus sinuatus* are distinguished by the long, sinuate channel extending nearly half the length of the pronotum (Fig. 9D), and by the granulate impressed area along the anterior margin of the pronotum. The females are quite similar to most others in the *mirabilis* group, but have very weak pronotal plicae, lack an inner subhumeral elytral stria, and have a fine, distinct marginal pygidial stria.

#### Etymology.

This species’ name refers to the sinuous track of the male gland opening on the pronotal disk.

### Operclipygus mutuca sp. n.

urn:lsid:zoobank.org:act:01D5EC0F-2AAE-4B34-8DCC-0CD6CF220646

http://species-id.net/wiki/Operclipygus_mutuca

Fig. 8B Map 2

#### Type locality.

BRASIL: Mato Grosso:Fazenda Mutuca [15°18.9'S, 55°58.2'W]

#### Type material.

**Holotype female**: “**BRASIL: Mato Grosso,** Mpio. Cuiabá, Fazenda Mutuca, 15.3145°S, 55.9703°W. Flight intercept traps. 24 Jan 2009 F.H. Gava & J.R. Rocha”, CEMT. **Paratypes** (2): **Pernambuco**: 1: Tapéra, Reichensperger (BMNH); 1: same as paratype but also with host label *Acromyrmex octospinosa pallida* Crawley [now synonymous with *Acromyrmex octospinosus* (Reich) s. str.] (BMNH).

#### Diagnostic description.

Length: 1.72–1.87 mm, width: 1.44–1.62 mm; frons flat, with complete frontal stria; supraorbital stria absent; pronotum with strong plicae in basal third; lateral submarginal pronotal striae complete, curving inward anteriorly nearly to meet recurved anterior submarginal stria; anterior marginal stria interrupted for width of head; median pronotal gland openings situated alongside of recurved submarginal stria; elytra with all dorsal striae elevated, subcarinate, with three complete epipleural striae, outer subhumeral stria barely interrupted at middle, inner subhumeral stria more or less complete, just abbreviated at base and apex, striae 1-5 and sutural complete, 5^th^ and sutural connected by a basal arch; apices of elytra with numerous coarse punctures tending to obscure apices of striae; prosternal keel truncate at base, with complete carinal striae united in a narrow arch just short of presternal suture, secondary carinal striae absent; mesoventral margin very weakly projecting at middle, marginal stria complete, mesometaventral stria weakly arched onto mesoventrite, turned posterad well mediad mesocoxa, with secondary lateral metaventral stria present from inner edge of mesocoxa nearly to metacoxa; 1^st^ abdominal ventrite with two lateral striae, outer stria abbreviated at inner corner of metacoxa; propygidium with sparse fine ground punctation and coarser punctures scattered, separated by about 2× their diameters; pygidium with similar ground punctation, and very few coarser punctures, particularly near anterolateral corners; pygidial marginal stria barely traceable along sides as an incomplete marginal crease. Male not known.

#### Remarks.

Even lacking male specimens, *Operclipygus mutuca* is easily distinguished by the presence of a strong pronotal plica, complete sutural and inner subhumeral striae, and generally elevated, subcarinate elytral striae (Fig. 8B). It is very similar in these characters to *Operclipygus carinistrius*, but lacks the deeply impressed marginal pygidial sulcus of that species.

#### Etymology.

This species’ name refers to its type locality, Fazenda (farm) Mutuca, near Cuiabá, Mato Grosso, Brazil, which the authors had the good fortune to visit during a 2011 field trip. It is a noun in apposition.

### Operclipygus carinistrius

(Lewis, 1908) comb. n.

http://species-id.net/wiki/Operclipygus_carinistrius

Fig. 8C Map 2

Phelister carinistrius Lewis, 1908: 159.

#### Type locality.

Not specified beyond Bahia State, Brazil.

#### Type material.

**Lectotype female**, here designated: “Bahia, A.G.” [Lewis’ description says only Brazil] / “Type” / “*Phelister carinistrius* Lewis Type” / “G. Lewis Coll. B.M.1926-369” / “LECTOTYPE *Phelister carinistrius* Lewis, 1908 M.S. Caterino & A.K.Tishechkin des. 2010” (BMNH); **Paralectotype female**: same data as lectotype (BMNH). This species was described from an unspecified number of specimens, and the lectotype designation fixes primary type status on one of two known syntypes.

#### Diagnosis.

*Operclipygus carinistrius* is very similar to *Operclipygus mutuca*,differing only in the following characters: anterior submarginal pronotal stria recurved more distinctly straight posterad; prosternal keel very weakly emarginate at base, with carinal striae complete, united in narrow anterior arch; outer subhumeral stria complete, inner subhumeral stria present in apical two-thirds, dorsal striae 1-5 and sutural complete; 5^th^ and sutural striae connected by a basal arch; elytral disk with scattered punctures near apex; propygidial punctures small, separated by about their diameters; pygidium smooth, apical marginal sulcus well developed, especially at sides as series of close, subcontiguous punctures. Male not known.

#### Remarks.

*Operclipygus carinistrius* is only known from its type specimens, both females. But based on numerous characters it can be placed in the *Operclipygus mirabilis* group, very close to the preceding species, *Operclipygus mutuca*. They differ primarily in the presence in *Operclipygus carinistrius* of a distinct pygicial sulcus, which is absent or at most very faint in *Operclipygus mutuca*.

### Operclipygus parensis sp. n.

urn:lsid:zoobank.org:act:4E45F14D-4F97-42EE-9E67-A6A34829A721

http://species-id.net/wiki/Operclipygus_parensis

Fig. 8D Map 2

#### Type locality.

BRAZIL: Pará: Monte Alegre [1°43'S, 54°20'W].

#### Type material.

**Holotype** of undetermined sex (probably male but genitalia missing): “**BRASIL: Pará,** Monte Alegre 1°43'S, 54°20'W Piège d’interception 10–27.ix.1992” / “Caterino/Tishechkin Exosternini Voucher EXO-00351” (UFPR). **Paratype** (1): same data as type (CHND).

#### Diagnostic description.

Length: 1.37–1.40 mm, width: 1.09–1.10 mm; Very small species, frons markedly convex, frontal stria complete or barely interrupted, supraorbital stria absent; pronotal disk with short basal plicae, extending forward no more than one-fourth pronotal length; lateral submarginal pronotal stria close to marginal, ending just short of anterolateral corner; anterior submarginal pronotal stria close to margin, obliquely divergent at sides, displacing marginal stria for entire width of anterior emargination; elytra with two complete epipleural striae, outer subhumeral stria complete, not interrupted, inner subhumeral stria absent, striae 1-5 complete, 5^th^ stria subacutely hooked anteriorly, not meeting sutural stria, which is obsolete in basal third; prosternal keel truncate at base, striae extending entire length of keel, connected by anterior arch, secondary lateral strioles absent; anterior mesoventral margin shallowly arcuate, marginal stria complete; 1^st^ abdominal ventrite with only a single lateral stria; propygidium and pygidium with only very fine, sparse ground punctures, lacking coarser punctures; propygidium with conspicuous transverse waves of microsculpture; pygidium lacking apical marginal stria. Male not known.

#### Remarks.

Although males are most highly distinctive in this group, these specimens are adequately distinguishable to justify recognition as a distinct species. The specimens were poorly preserved, losing their genitalia prior to mounting. However, both have very weak tubercles on each side of the anterior submarginal pronotal stria, which would appear to represent the median pronotal gland openings of the male. The discovery of definite males would permit a more confident characterization of the species. In any case, *Operclipygus parensis* can be recognized by the combination of small size, weak pronotal plicae, absence of inner subhumeral stria, absence of a marginal pygidial sulcus, and presence of very weak tubercles on the anterolateral pronotal corners.

#### Etymology.

This species is named for the Brazilian state where it is found.

### Operclipygus schlingeri sp. n.

urn:lsid:zoobank.org:act:D51A041C-D038-4BB9-9CE1-FF388B84B6C3

http://species-id.net/wiki/Operclipygus_schlingeri

Figs 8E–F 10E, H Map 2

#### Type locality.

PERU: Huanuco: Tingo Maria, Monson Cave [9°18'S, 75°59'W].

#### Type material.

**Holotype male**: “PERU: Monson Cave Tingo Maria XII-15-1954” / “E.I.Schlinger, & E.S.Ross collectors” / “Caterino/Tishechkin Exosternini Voucher EXO-00188” (CASC). **Paratype** (1): same data as type (CASC).

#### Diagnostic description.

Length: 1.53–1.56 mm, width: 1.31–1.33 mm; body rufobrunneus, elongate oval, with conspicuous ground punctation throughout, especially on pronotum; frons flat, sides of frontal stria convergent anteriorly, abruptly bent dorsomediad above antennal bases, continuous but strongly bisinuate across middle of frons; epistoma weakly emarginate; labrum narrow, only slightly wider than long, apex rounded; mandibles markedly narrowed to apices, left untoothed, right with small, acute basal tooth; pronotum with sides relatively straight in basal two-thirds, convergent to front; pronotal disk with strong basal plicae extending forward from base of 3^rd^ dorsal stria, with only very faint prescutellar impression, ground punctation very conspicuous, with numerous coarser punctures toward sides; marginal pronotal stria broadly interrupted behind head; lateral submarginal pronotal stria close to margin, complete, joined with anterior submarginal stria (male) or free anteriorly (female); pronotum with only single pair of anterior gland openings, in female located along anterior margin close to anterior pronotal corners, in male apparenly displaced onto hypomeron in the vicinity of an anterolateral groove formed by marginal stria; elytron with two complete epipleural striae, outer subhumeral stria complete, inner subhumeral stria absent, dorsal striae 1-5 complete, 5^th^ stria weakly arched to sutural at base, 4^th^ and 5^th^ striae weakly fragmented toward apex, sutural stria present in apical three-fourths; elytral disk with conspicuous, disorganized punctures along apical margin; prosternal keel trunctate at base, carinal striae complete, free basally, united in rather broad anterior arch; prosternal lobe narrowed, subtruncate apically, with marginal stria obsolete at middle, but well impressed and diverging from margin to presternal suture at side in male, female prosternal lobe with fine marginal stria apically, not impressed at side; mesoventral margin shallowly emarginate, marginal stria interrupted; mesometaventral stria broadly arched forward on mesoventrite, reaching about one-third behind anterior margin, continued posterad by lateral metaventral stria to inner third of metacoxa; metaventral disk with ground punctation only; 1^st^ abdominal ventrite with one complete lateral stria; propygidium with sparse but conspicuous ground punctation, small coarse punctures evenly interspersed, separated by 1–2× their diameters; pygidium with conspicuous ground punctation and few slightly coarser punctures intermixed; marginal pygidial sulcus well impressed, complete to base, interrupted apically in holotype, complete in paratype. Male genitalia (Figs 10E, H): accessory sclerites absent; T8 rather short, with sides subparallel in basal two-thirds, abruptly narrowed to apex, narrowly emarginate apically, with broad, shallow basal emargination, basal membrane attachment line distad basal emargination, ventrolateral apodemes nearly meeting at midline; S8 narrowed strongly to apex, apical guides narrow, only barely widening to apex, ventral halves weakly divergent; T9 with apices simple, pointed inward; T10 with halves fully separate; stem of S9 narrow, rounded basally, apical emargination narrow, apical flanges separate; tegmen short, broad, sides rounded, rather evenly curved dorsoventrally, medioventral process ‘U’-shaped, projecting beneath about one-fourth from base; median lobe about one-third tegmen length, proximal apodemes uniform; basal piece nearly one-half tegmen length.

#### Remarks.

The unique frontal stria of *Operclipygus schlingeri* (Fig. 8F) is sufficient to recognize it among Neotropical Exosternini.

#### Etymology.

We name this species for one of its collectors, Dr. Evert Schlinger, also in recognition of his support of entomology at the Santa Barbara Museum of Natural History.

### *Operclipygus kerga* group

**Figure 11.**
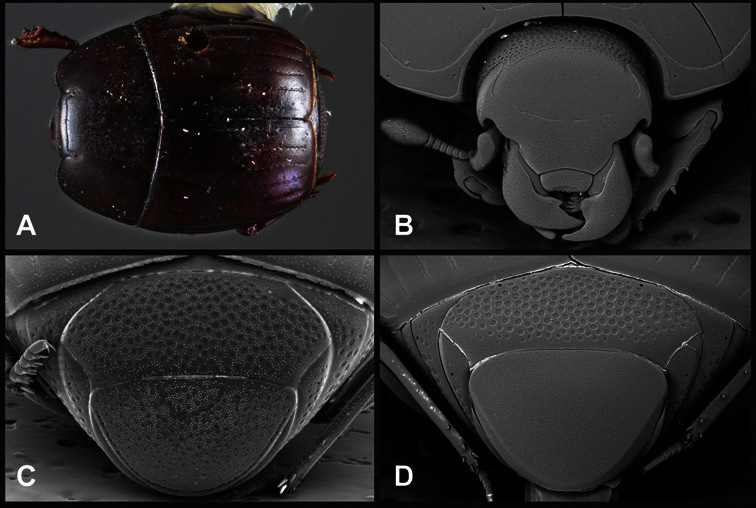
*Operclipygus kerga* group. **A** Dorsal habitus *Operclipygus kerga* (lectotype) **B** Frons *Operclipygus kerga* **C** Pygidia of *Operclipygus punctistrius* **D** Pygidia of *Operclipygus planifrons*.

This group contains three more or less cryptic species, most easily distinguished by aedeagus shape. The most distinctive external character they share is a flat to moderately convex frons (Fig. 11B), with the central part of the frontal stria detached from the sides and present as a short transverse line, or completely absent. Nearly all other *Operclipygus* have the frons at least slightly depressed in the middle. In addition, they are characterized by the following characters: body rounded, moderately and evenly convex; disk of pronotum with few or no coarse lateral punctures; inner subhumeral stria more or less complete; pygidium with dense, fine ground punctation and variably few and sparse coarser punctures; pygidial marginal stria relatively weak, abbreviated at sides to almost absent in some species/individuals.

### Key to the species of the *Operclipygus kerga* group

**Table:** 

1	Marginal pygidial sulcus absent (Fig. 11D); pronotum lacking coarse lateral punctures	*Operclipygus planifrons* sp. n.
–	Marginal pygidial sulcus present; pronotum with or without coarse lateral punctures	2
2	Marginal pygidial sulcus weak, often fragmented or abbreviated basally (Fig. 11C); aedeagus abruptly narrowed in apical third, its apices angulately truncate (Fig. 12G)	*Operclipygus punctistrius* sp. n.
–	Marginal pygidial sulcus complete; aedeagus with sides evenly rounded to base and apex (Fig. 12F)	*Operclipygus kerga* (Marseul)

### Operclipygus kerga

(Marseul, 1870)

http://species-id.net/wiki/Operclipygus_kerga

**Map 3.**
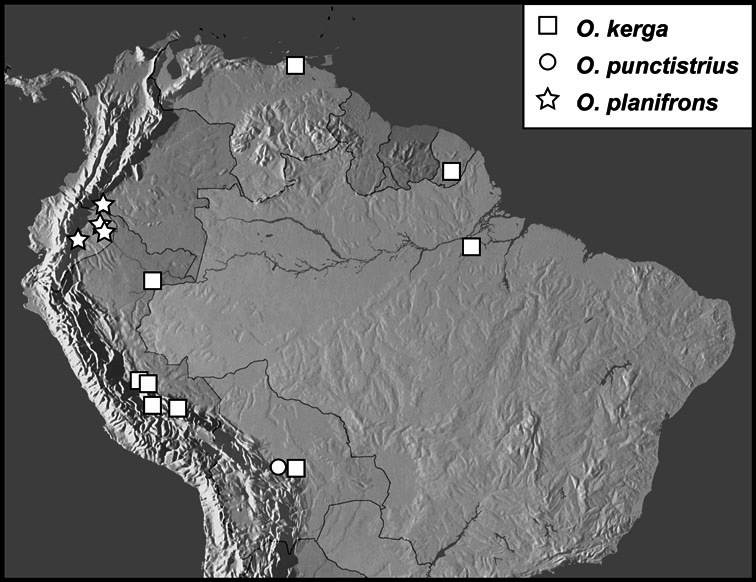
Records of the *Operclipygus kerga* group.

Figs 11A–B 12A–C, F Map 3

Phelister kerga Marseul, 1870: 77; *Pseudister kerga*: Bickhardt 1917: 165; *Operclipygus kerga*: Wenzel 1976: 259.

#### Type locality.

Unspecified, somewhere along the Amazon in Brazil.

#### Type material.

**Lectotype**, here designated, of undetermined sex: “*Phelister kerga*, Amazon” / “LECTOTYPE *Phelister kerga* Marseul 1870, M.S.Caterino & A.K.Tishechkin des. 2010” (MNHN). This species was described from an unspecified number of specimens, and the lectotype designation fixes primary type status on the only known original specimen.

#### Other material.

**BOLIVIA: Santa Cruz**: 6: 4–5km SSE Buena Vista, Hotel Flora y Fauna, 17°29.9'S, 63°39.1'W, 15–24.xii.2003, FIT, S. & J. Peck (AKTC); 2: [country record only] (ZMHB). **BRAZIL: Pará**: 1: Monte Alegre, 3°9'S, 52°3'W, 17.vi.1992, 3.vii.1992, FIT (CHND); 1: 10–27.vi.1992, FIT (CHND). 5: Tucuruí, 3°45'S, 49°40'W, 10–29.vii.1985, FIT (CHND); 1: 19.vi.1986, 7.vii.1986, FIT (CHND). **FRENCH GUIANA:** 1: Belvèdére de Saül, point de vue, 3°1'22"N, 53°12'34"W, 30.xi.2010, FIT, SEAG (CHND). **PERU: Cusco:** 1: Cock of the Rock Lodge, NE Paucartambo, 13°3.3'S, 71°32.7'W, 1120m, 4.xi.2007, 9.xi.2007, FIT, D. Brzoska (SEMC). 1: Manu rd. km 165, Consuelo, 10.x.1982, beating dead branches, L. Watrous & G. Mazurek (FMNH); 1: 12.x.1982, rotten palm, L. Watrous & G. Mazurek (FMNH). **Junín:** 4: Pampa Hermosa Lodge, 22km N San Ramon, 10°59.3'S, 75°25.5'W, 1220m, 24–27.xi.2007, FIT, D. Brzoska (SEMC). 1: 11km NE Puerto Ocopa, Los Olivos, 11°7.00'S, 74°15.52'W, 1200m, 26–28.iii.2009, Window trap, A.V. Petrov (AKTC); 1: 24–26.iii.2009, Window trap, A.V. Petrov (MUSM). 1: 7.5km NE Puerto Ocopa, Cananeden, 11°4.9'S, 74°16.1'W, 1180m, 11.i.2007, A.V. Petrov (AKTC); 1: 15km NW Satipo, Rio Venado, 11°11.709'S, 74°46.119'W, 1150m, 17–24.v.2012, A.V. Petrov, DNA Extract MSC-2322 (AKTC). **Loreto:** 2: km 63, rd. Iquitos - Nauta, Rio Itaya, 4°15.205'S, 73°26.074'W, 140m, 10–14.ii.2010, A.V. Petrov (AKTC). 1: 9–13.i.2011, A.V. Petrov (MUSM). **Madre de Dios:** 1: Los Amigos Field Sta., Luisa Bamboo forest, 12.5422°S, 74.1170°W, 274m, 2–11.i.2007, pitfall, bamboo forest, J. Jacobs (CASC). 1: Manu National Park, Zona res., Rio Manu, Cocha Juarez, trail nr. Manu Loge, 18–24.ix.1991, FIT, A. Hartman (FMNH). 16: Manu National Park, Pantiacolla Lodge, Alto Madre de Dios River, 12°39.3'S, 71°13.9'W, 420m, 14–19.xi.2007, FIT, D. Brzoska (SEMC). 1: Manu National Park, Pantiacolla Lodge, 5.5km NW El Mirador Trail, Alto Madre de Dios River, 12°39'10"S, 71°15'28"W, 500m, 23–26.x.2000, FIT, R. Brooks (SEMC). 6: Manu National Park, Cocha Cashu Bio. Sta., 11°53'45"S, 71°24'24"W, 350m, 17–19.x.2000, FIT, R. Brooks (SEMC). 2: Manu National Park, Cocha Salvador, 12°0'12"S, 71°31'36"W, 310m, 20–21.x.2000, FIT, R. Brooks (SEMC). 2: Tambopata, Reserva Cuzco Amazonico, 15km NE Puerto Maldonado, Quebrada Mariposa, 12°33'S, 69°03'W, 200m, 13.vi.1989, FIT, R. A. Leschen (SEMC). **VENEZUELA: Monagas:** 11: Caripe Cueva Guácharo, 700m, 20–30.vii.1987, malaise trap, forest over coffee, S. & J. Peck (FMNH).

#### Diagnostic description.

**Figure 12.**
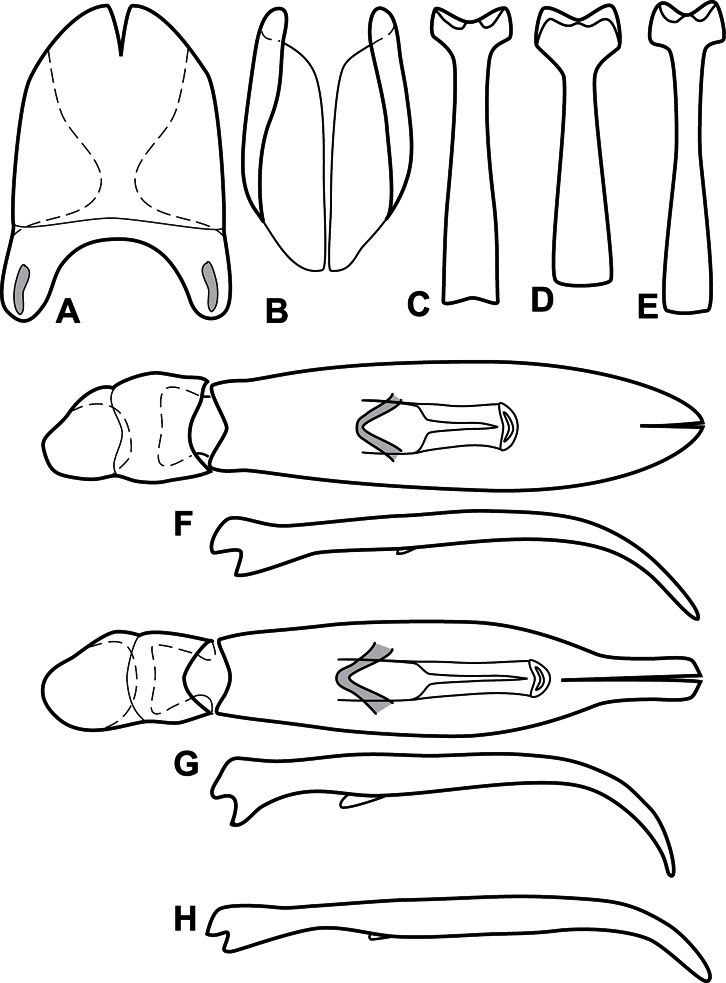
Male genitalia of *Operclipygus kerga* group. **A** T8 of *Operclipygus kerga* **B** S8 of *Operclipygus kerga* **C** S9 of *Operclipygus kerga* **D **S9 of *Operclipygus punctistrius* **E** S9 of *Operclipygus planifrons* **F** Aedeagus, dorsal and lateral views, of *Operclipygus kerga* **G** Aedeagus, dorsal and lateral views, of *Operclipygus punctistrius* **H** Aedeagus, lateral view, of *Operclipygus planifrons***.**

Length: 2.40–2.87 mm, width: 2.18–2.46 mm; body rounded at sides, moderately convex dorsally; frons slightly convex; frontal stria interrupted on each side; supraorbital stria complete across vertex, barely separated from portion of frontal stria in front of eyes; pronotum with lateral submarginal stria complete, free anteriorly; anterior submarginal pronotal stria present; median pronotal gland openings slightly behind anterior margin, near apices of recurved submarginal stria; pronotal disk with few or no coarse lateral punctures; antescutellar fovea present, small, ovoid; elytra with outer subhumeral, inner subhumeral, and striae 1-3 complete, striae 4 and 5 usually subequal, present in apical third to one-half, sutural stria present in apical two-thirds; prosternal keel striae convergent and connected anteriorly, keel projecting posteriorly; marginal mesoventral stria complete; mesometaventral stria outwardly arcuate, nearly in contact with marginal mesoventral stria at middle; central portion of metaventral disk impunctate, small fragment of secondary lateral metaventral stria generally present; propygidium uniformly covered with round punctures separated by one-fourth their diameters; pygidium with dense, fine ground punctation, with small but coarser punctures sparsely intermingled, apical marginal stria complete, finely crenulate. Male genitalia (Figs 12A–C, F): accessory sclerites present; T8 short, basal emargination broad, apical emargination narrow, basal membrane attachment line separated from basal emargination by one-third its depth, ventrolateral apodemes not meeting at midline, most strongly developed basally, narrowed toward apex; S8 with narrow apical guides gradually widened to apex, halves approximate in basal half, weakly diverging to apex; T9 with apices bluntly rounded, ventrolateral tooth well developed, just beyond midpoint; T10 halves separate; S9 narrowest near apex, expanded toward base, parallel sided in basal fourth, base truncate to weakly emarginate, lacking apical emargination, apical flanges separate; tegmen with sides evenly rounded to base and apex, medioventral process fine, narrowly rounded, weakly projecting beneath just basad midpoint; basal piece just less than one-third tegmen length; median lobe about one-half tegmen length.

#### Remarks.

Of two specimens in ZMHB, one bears a label *‘Phelister bimarginatus*’, apparently in Bickhardt's handwriting. However, this is a nomen nudum. Presumably, Bickhardt intended to describe this species before deciding it was *Operclipygus kerga*.

The type locality is merely the Amazon region. Beyond that it is widespread in much of the Amazonian basin, except where it is replaced by its close relatives, described below.

### Operclipygus punctistrius sp. n.

urn:lsid:zoobank.org:act:31C1A1E8-9ED3-4800-BA30-45A53114DC38

http://species-id.net/wiki/Operclipygus_punctistrius

Figs 11C 12D, G Map 3

#### Type locality.

BOLIVIA: Cochabamba: 67.5 km E Villa Tunari, Valle Sajta Biological Station [17°6.3'S, 64°46.9'W].

#### Type material.

**Holotype male**: “**BOLIVIA: COCHABAMBA** 67.5km E Villa Tunari, Est. Biol. Valle Sajta, Univ. San Simon 300m, 17°06'19"S, 64°46'57"W, 7–9.II.1999, F. Génier, lowland rain forest, ex. f.i.t. 1 99-041” / “Caterino/Tishechkin Exosternini Voucher EXO-00095” (CMNC). **Paratypes** (36): all same locality as type, 9: same date as type, 25: 9–13.II.1999 (CMNC, FMNH, MSCC, AKTC).

#### Diagnostic description.

This species is difficult to separate from *Operclipygus kerga* by external characters. It is on average slightly smaller (length: 2.31–2.65 mm, width: 2.03–2.40 mm) and frequently has the sides of the pygidial marginal stria abbreviated, or present only as a disconnected series of punctures (Fig. 11C). Specimens must be dissected for definite identification, and the aedeagus is highly distinctive, with the tegmen abruptly narrowed in the apical third, having the apices angulately truncate, and with the medioventral process more narrowly rounded, but more strongly projecting beneath. In addition, S9 is more robust, with a wider, truncate base and wider lateral flanges, and is more strongly sclerotized.

#### Remarks.

This species may only be separated from *Operclipygus kerga* by the characters in the description, particularly by the form of the aedeagus (Fig. 12G).

#### Etymology

**.** This species’ name refers to the frequent reduction of the pygidial sulcus to a series of marginal punctures.

### Operclipygus planifrons sp. n.

urn:lsid:zoobank.org:act:CDBFFFE3-133A-4054-A497-B2D3332FCBFF

http://species-id.net/wiki/Operclipygus_planifrons

Figs 11D 12E , H Map 3

#### Type locality.

ECUADOR: Orellana: Yasuní Research Station [0°40.5'S, 76°24'W].

#### Type material.

**Holotype male**: “**Ecuador:** Napo, mid.Rio Tiputini, Yasuní Res. Stn. 0°40.5'S, 76°24'W, FIT#3. 28Jun-5Jul 1999 AKT#049 C.Carlton & A.Tishechkin” / “LSAM 0013189” (FMNH). **Paratypes** (8): 1: same locality as type, 23.vii–4.viii.1999, FIT, A.K. Tishechkin (LSAM). **ECUADOR: Orellana:** 2:Yuturi Lodge, Rio Napo, 0°32'54"S, 76°2'18"W, 270m, 20–21.iii.1999, FIT, R. Brooks & D. Brzoska (SEMC). 1: Tiputini Biodiversity Station, 0.6376°S, 76.1499°W, 4–9.vi.2011, FIT, M.S. Caterino & A.K. Tishechkin, DNA Extract MSC-2181 (SBMNH). **Pastaza:** 1: Cusuimi, Rio Cusuimi 150km SE of Puyo, 320m, 15–31.v.1971, beating & sifting rotten foliage, B. Malkin (FMNH). **Sucumbíos:** 1:Sacha Lodge, 0.5°S, 76.5°W, 270m, 14–24.iii.1994, malaise trap, Hibbs (SEMC). **COLOMBIA: Putumayo:** 2: Santa Rosa (Kofan Indian village): headwaters of Rio San Miguel, 16–20.x.1970, on fermenting stump of cut palm, B. Malkin & P. Burchard (FMNH).

#### Diagnostic description.

As with the previous, this species is nearly identical to *Operclipygus kerga* in external characters. It is very similar in size (length: 2.40–2.53 mm, width: 2.12–2.22 mm). The only consistent difference is the near complete absence of a pygidial marginal stria (Fig. 11D). In a few individuals some fragments of this stria are visible near the apex, but in most it is completely absent. The genitalia are also very similar to *Operclipygus kerga*, with a slightly narrower, more elongate S9 (Fig. 12E), and a tegmen that is slightly thicker dorsoventrally (as viewed in lateral aspect; Fig. 12H.)

#### Remarks.

This species may only be separated from *Operclipygus kerga* by the characters in the description.

#### Etymology.

This species’ name refers to its flat frons, shared generally by members of the *Operclipygus kerga* group.

### *Operclipygus conquisitus* group

The three species in this group are united primarily by the presence of very dense ground punctation on atypical parts of the body (as opposed to its common presence on the pygidium in many *Operclipygus*). This punctation occurs on much of the body of *Operclipygus bicolor* and *Operclipygus conquisitus*, while it is much more restricted in *Operclipygus friburgius*. Support from other characters is hard to find, however, and the lack of a male of *Operclipygus friburgius* leaves some doubt as to its relationships.

### Key to the species of the *Operclipygus conquisitus* group

**Table:** 

1	Dense ground punctation limited to epistoma, prosternal lobe, prosternal keel, pygidium, and mesoventrite (Fig. 13E)	*Operclipygus friburgius* (Marseul)
–	Dense ground punctation present on most body surfaces, including on pronotum and metaventrite (but excluding elytra)	2
2	Body bicolored (Fig. 13B), elytra reddish with rest of body rufo-brunneus; mesometaventral stria doubled, with one arch on mesoventrite and a parallel arch posterad mesometaventral suture (Fig. 12C)	*Operclipygus bicolor* sp. n.
–	Body uniformly rufobrunneus; venter with single mesometaventral stria arched onto mesoventrite	*Operclipygus conquisitus* (Lewis)

### Operclipygus conquisitus

(Lewis, 1902)

http://species-id.net/wiki/Operclipygus_conquisitus

**Figure 13.**
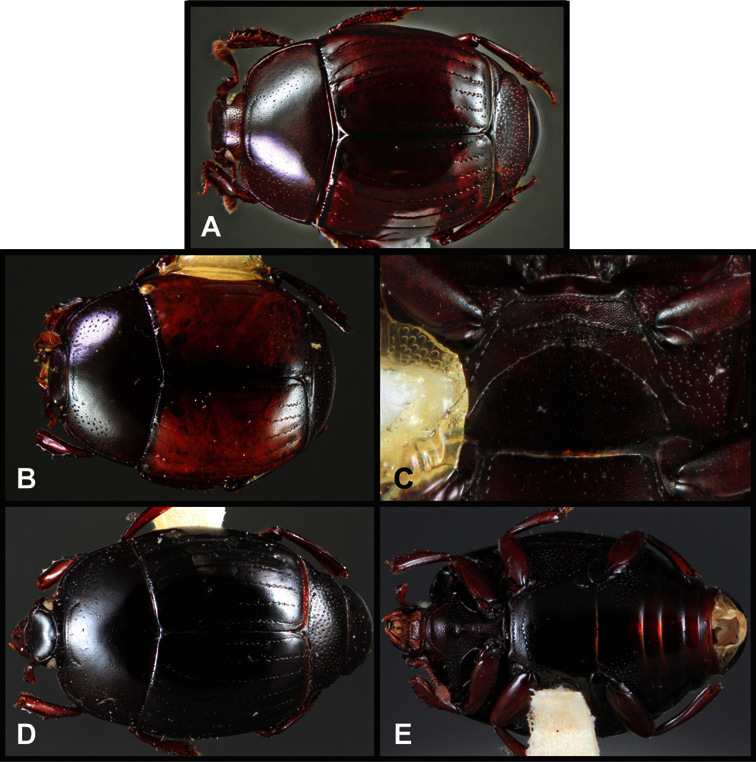
*Operclipygus conquisitus* group. **A** Dorsal habitus of *Operclipygus conquisitus* **B** Dorsal habitus of *Operclipygus bicolor* **C** Metaventrite of *Operclipygus bicolor* **D** Dorsal habitus of *Operclipygus friburgius* **E** Ventral habitus of *Operclipygus friburgius*.

**Map 4.**
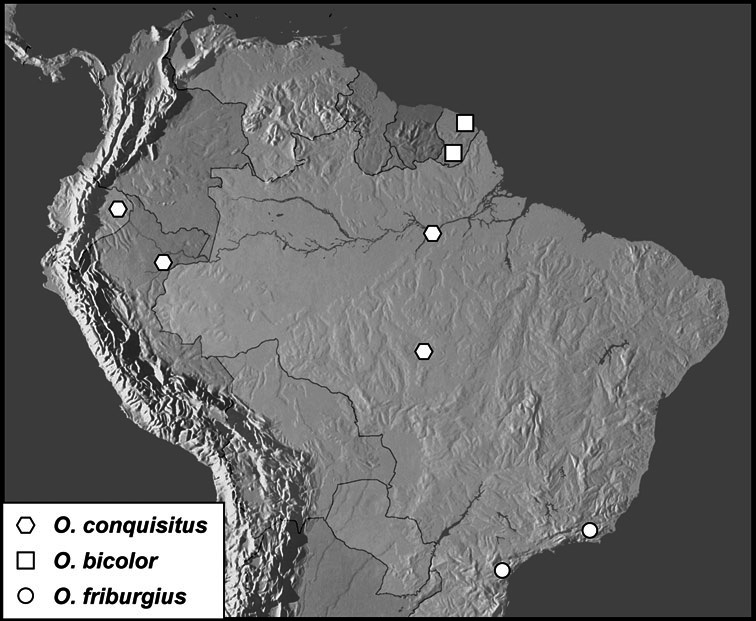
Records of the *Operclipygus conquisitus* group.

Figs 13A 14A–D Map 4

Phelister conquisitus Lewis, 1902: 235; *Operclipygus conquisitus*: Wenzel (1976: 257).
Phelisteroides ruptistrius Wenzel, 1944: 141; synonymized by Wenzel (1976: 257).

#### Type locality.

BRAZIL: Pará: Santarém [2°26'S, 54°42'W].

#### Type material.

***Phelister conquisitus***: Type missing. Published type locality: “Ulster Co. N.Y.” [in error!]; **Neotype male**, here designated: “Santarem” (BMNH). This specimen was determined by Hinton, himself having apparently studied Lewis's type before its loss (Hinton 1935a: 588). ***Phelisteroides ruptistrius***: **Holotype**: (CMNH), not examined. **Paratype**: “Santarem Brazil, Acc. No. 2966” / Dec. (FMNH), examined, 2010.

#### Other material.

**BRAZIL: Mato Grosso:** 1: Claudia, 11°24.5'S, 55°19.5'W, 17–27.x.2010, FIT, A.F. Oliveira (CEMT). **ECUADOR: Orellana:** 1:Res. Ethnica Waorani, 1km S Onkone Gare Camp, Trans. Ent., 0°39'10"S, 76°26'W, 220m, 6.vii.1995, fogging, T.L. Erwin (USNM). **PERU: Loreto:** 1: Iquitos - Nauta rd., km 58, Rio Itaya, 4°15.738'S, 73°28.052'W, 120m, 5–9.v.2009, Window trap, next to entrance, *Eciton burchelli* statary bivouac in hollow treee, A.V. Petrov (AKTC). 3: 68km SW Iquitos to Nauta, Rio Itaya, 4°11'S, 73°26'W, 110m, 1–3.iii.2008, A.V. Petrov (AKTC). 3: 26–30.ii.2008, A.V. Petrov (AKTC). 4: 18–19.i.2008, A.V. Petrov (AKTC, FMNH). 1: 9.ii.2007, A.V. Petrov (MUSM). 2: 1–5.iii.2008, A.V. Petrov (MUSM). 1: Left bank of Rio Amazonas 70km SSW Iquitos to Nauta, 140m, 25.ii.2008, A.V. Petrov (AKTC). 3: km 63, rd. Iquitos - Nauta, Rio Itaya, 4°15.205'S, 73°26.074'W, 140m, 9–13.i.2011, A.V. Petrov (AKTC). 4: 8–17.i.2010, A.V. Petrov (AKTC, MUSM). 1: 10–14.ii.2010, A.V. Petrov (AKTC).

#### Diagnostic description.

**Figure 14.**
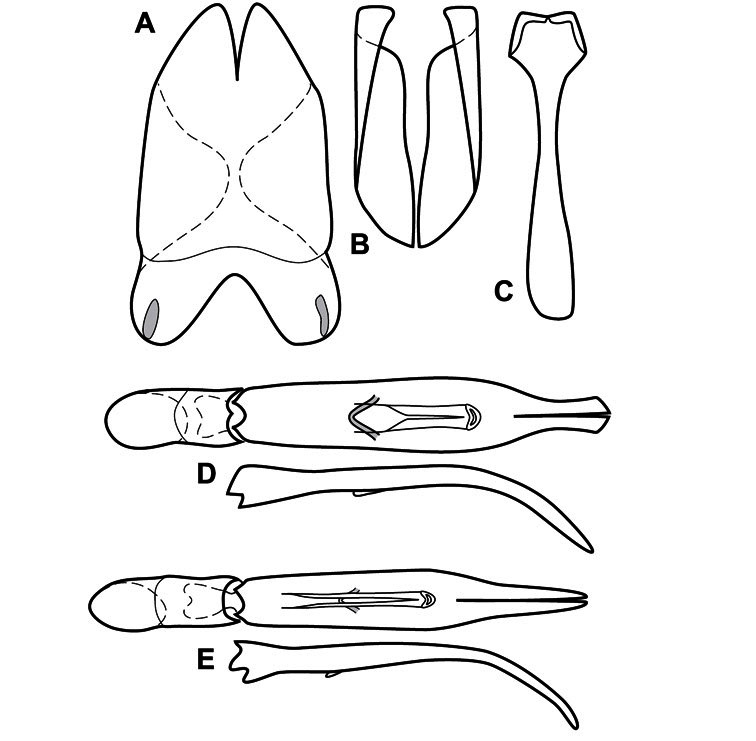
Male genitalia of *Operclipygus conquisitus* group. **A** T8 of *Operclipygus conquisitus* **B** S8 of *Operclipygus conquisitus* **C** S9 of *Operclipygus conquisitus* **D** Aedeagus, dorsal and lateral views, of *Operclipygus conquisitus* **E** Aedeagus, dorsal and lateral views, of *Operclipygus bicolor*.

Length: 2.00–2.34 mm, width: 1.68–2.00 mm; body ovoid, widest behind middle, rufobrunneus, with fine, dense ground punctation on frons, pronotum, and all sterna. Frons and epistoma convex; frontal stria present only along inner edge of eye, absent across front, not joining fine supraorbital; pronotum with marginal stria complete across anterior margin; lateral submarginal pronotal stria complete at side, curving inward at front but ending freely; anterior submarginal stria crenulate, weakly recurved at sides; pronotal disk with few larger punctures towards sides; median pronotal gland openings difficult to detect amidst ground punctation, but present about one-third of pronotal length behind anterior margin, directly behind ends of anterior submarginal stria; elytra with one complete epipleural stria, outer subhumeral stria present in apical half, inner subhumeral stria absent, striae 1-3 complete, 4^th^ present in basal half, arched inward at base but not reaching sutural stria, also represented by apical fragments, occasionally complete, 5^th^ stria only present in apical fourth, often fragmented, sutural stria in apical 2/3; elytral disk with narrow band of small punctures sparsely scattered along apical margin; prosternal keel broad, flat, truncate at base, carinal striae evenly convergent, joined by broad anterior arch and fine basal line; prosternal lobe with marginal stria complete, strongly impressed; anterior margin of mesoventrite shallowly, inwardly arcuate, not distinctly emarginate, marginal stria complete; mesometaventral stria narrowly arched to midpoint of mesoventral disk, crenulate, posteriorly extending toward inner third of metacoxa; 1^st^ abdominal ventrite with inner lateral stria complete, outer abbreviated posteriorly; propygidium with fine, rather sparse ground punctation, with small ocellate punctures irregularly separated by about their diameters, denser toward base; pygidial ground punctation fine, moderately dense, additional small punctures very sparsely interspersed; apical sulcus fine but nearly complete to base. Male genitalia (Fig. 14A–D): accessory sclerites present, small; T8 short, sides weakly convergent, rounded, angled in to apex, apical emargination narrow, basal emargination deep, broad, meeting basal membrane attachment line at apex, ventrolateral apodemes most strongly developed at base, narrowing to apex; S8 with sides divergent in apical third, apical guides strongly developed in apical half, apices broad and bluntly rounded, with several conspicuous apical setae, ventral halves separate throughout length, diverging slightly apicad; T9 with sides parallel in basal half, converging to narrow, subacute opposing apices; T10 with halves separate; S9 with stem narrowest near midpoint, rather abruptly widened to broadly subtruncate base, apical margin with very shallow median emargination, apical flanges short and separate; tegmen widest just beyond midpoint, abruptly narrowed toward apex, then widened, apices obliquely truncate, apical third of tegmen abruptly bend downward, medioventral process weak, very narrowly ‘U’-shaped, barely projecting beneath, about one-third from base; basal piece about one-third tegmen length; median lobe also about one-third tegmen length, with filamentous stems of proximal apodemes very short.

#### Remarks.

The presence of very conspicuous fine, dense ground punctation on the head, pronotum (Fig. 13A), and sterna is unique to this species.

The published type locality of Ulster Co., New York, USA has been convincingly discredited (Hinton 1935a), and the species is instead known to occur in Amazonian parts of South America. The type specimen was deposited at BMNH along with Lewis's collection, and the pin, point, and original labels are present in that collection. However, no specimens corresponding to the species distinctive characters have been found. One of us (MSC) was resident at BMNH during the conversion of the histerid collection from slats to unit trays, and a thorough search was conducted at that time (year 2000). Wenzel's synonymous *Operclipygus ruptistrius*, which agrees in all respects with *Operclipygus conquisitus*, was described from this area, as well.

### Operclipygus bicolor sp. n.

urn:lsid:zoobank.org:act:4315425F-4201-4F7A-AE50-0BC407B90FD6

http://species-id.net/wiki/Operclipygus_bicolor

Figs 13B–C 14E Map 4

#### Type locality.

FRENCH GUIANA: Itoupé Table Mountain [3°1.38'N, 53°5.73'W].

#### Type material.

**Holotype male**: “**GUYANE FR.,** Mont tabulaire Itoupé. 3°1.38'N, 53°5.73'W. 570 m. Piège d’interception, 17 Mar 2010. SEAG leg.” / “Caterino/Tishechkin Exosternini Voucher EXO-00548” (MNHN). **Paratypes** (14): **FRENCH GUIANA:** 8: same data as type (CHND, FMNH, MSCC, AKTC). 4: same locality as type, 24.iii.2010, FIT, SEAG (CHND), 1: 31.iii.2010, FIT, SEAG (CHND). 1: Route de l’est, après la riviere Comté, 4°39'N, 52°20'W, 7.i.1978, G. Nazaret (CHND).

#### Diagnostic description.

This species is very similar in many respects to *Operclipygus conquisitus*, especially in the presence over much of the body of dense ground punctation. It is described only to the extent that it differs here: length: 1.97–2.18 mm, width: 1.65–1.87 mm; frons with central part of frontal stria present; pronotum lacking dense ground punctation, median pronotal gland openings just beyond apices of weakly recurved anterior submarginal stria, only about 8 puncture widths from anterior margin; elytra distinctly reddish, darkened slightly along suture; base of prosternal keel weakly emarginate; mesoventrite weakly projecting; marginal mesoventral stria complete; metaventrite with secondary anterior stria, parallel to mesometaventral stria, but just reaching to mesometaventral suture; pygidium with coarse, secondary punctures slightly denser than in *Operclipygus conquisitus*. Male genitalia (Fig. 14E): accessory sclerites present, not unusually small; T8 with sides weakly convergent in basal two-thirds, angulate to narrow apex, apical emargination narrow, basal emargination broadly rounded, basal membrane attachment line distad by about one-half emargination depth from its apex, ventrolateral apodemes symmetrical anteriorly and posteriorly, nearly meeting at midline; S8 with sides parallel, apical guides narrow, widended to apex, apices subtruncate, ventral halves approximate in basal fourth, weakly diverging to near apex; T9 with apices subacute, slightly convergent; T10 with halves separate; S9 with stem narrowed at middle, widened to basal fourth, basal sides subparallel to truncate base, head with lateral flanges rather broad, apex lacking median emargination, apical flanges narrowed at middle, but continuous; tegmen narrow, parallel-sided in basal half, strongly narrowed to subacute apex, apical third bent ventrad, with thin ‘U’-shaped medioventral process projecting beneath about one-fourth from base; basal piece nearly one-half tegmen length; median lobe slightly over one-half tegmen length, proximal apodemes undifferentiated, thin and separate.

#### Remarks.

The red coloration of the elytra (Fig. 13B) of *Operclipygus bicolor* is practically sufficient for identification. There are very few bicolored histerids in the Neotropics, and even fewer Exosternini. This character, in addition to the dense ground punctation of the head and sterna allow confident identification of this species.

#### Etymology.

This species’ name refers to its bicolored appearance.

### Operclipygus friburgius

(Marseul, 1864)

http://species-id.net/wiki/Operclipygus_friburgius

Figs 13D–E Map 4

Phelister friburgius Marseul, 1864: 318.
Phelister friburgicus (misspelling): Gemminger and Harold 1868: 761.

#### Type locality.

BRAZIL: Rio de Janeiro:Nova Friburgo [22°16'S, 42°32'W].

#### Type material.

**Lectotype,** here designated, probably female: “*Phelister friburgius*, N. Friburg” / “LECTOTYPE *Phelister friburgius* Marseul 1864, M.S.Caterino & A.K.Tishechkin des. 2010”(MNHN). **Paralectotype**: “Bresil”/“*friburgicus* [sic] Mars.”/“Marseul 12.14.86” (BMNH). This species was described from an unspecified number of specimens, and the lectotype designation fixes primary type status on one of two known syntypes.

#### Other material.

**BRAZIL: Paraná:** 1: Piraquara, Mananciais da Serra, 2008, P. Grossi (UFPR). **Rio de Janeiro:** 1: Nova Friburgo, 22°16'S, 42°32'W, 26–31.x.2009, FIT (CHND).

#### Diagnostic description.

Length: 2.56–2.60 mm, width: 2.22–2.25 mm; body piceous, elongate oval, widest at humeri, convex; frons generally convex, but slightly depressed at middle, finely but densely punctate, increasingly so towards epistoma; lateral portions of frontal striae divergent anterad, slightly rounded, meeting complete supraorbital dorsally, frontal portion weak, both detached from lateral portions and interrupted at middle; labrum about 2.5× as wide as long, weakly emarginate at middle; pronotum faintly depressed in prescutellar region, with a few small punctures near depression, ground punctation overall fine, sparse, though becoming more conspicuous at sides, with ~15 coarser lateral punctures; anterior marginal pronotal stria complete; lateral submarginal pronotal stria complete at sides, incurved anteriorly but ending free; anterior submarginal stria present, with ends briefly recurved posterad; median pronotal gland openings laterad apices of recurved anterior stria, about 8 puncture widths from anterior margin; elytra with two complete epipleural striae, outer subhumeral stria present in apical half, inner subhumeral stria absent, striae 1-4 complete, 5^th^ present in apical third, sutural stria present in apical two-thirds; elytron with apical transverse row of small punctures; prosternal keel truncate at base, with carinal striae complete, convergent anteriorly, united by narrow anterior arch, enclosed area of keel with dense fine punctation, similar punctation on prosternal lobe and mesoventral disk; prosternal lobe with marginal stria strongly abbreviated at sides; mesoventrite broadly and shallowly emarginate, marginal stria complete; mesometaventral stria weakly arched forward onto basal third of mesoventral disk, continued by lateral metaventral stria to inner one-third of metacoxa; 1^st^ abdominal ventrite with one complete lateral stria, disk with few small punctures near this stria; propygidium with fine ground punctation sparse at base, denser apically, with small round punctures separated by about 1.5× their widths; pygidium with fine, dense ground punctation not quite reaching basal margin, coarser punctures fairly dense at base, sparser toward apex; apical marginal stria fine, ranging from a dense series of punctures to a complete stria. Male: not known.

#### Remarks.

The patterns of fine punctation on this species is unique, with the frontal punctation grading to denser toward the epistoma, the increasingly dense pygidial ground punctation toward the apex, and the fine punctures within the prosternal striae and on the prosternal lobe (Fig. 13E). This ground punctation is the main basis for grouping the species with *Operclipygus conquisitus*, and examination of a male *Operclipygus friburgius* would help substantiate this relationship.

### *Operclipygus impuncticollis* group

**Figure 15.**
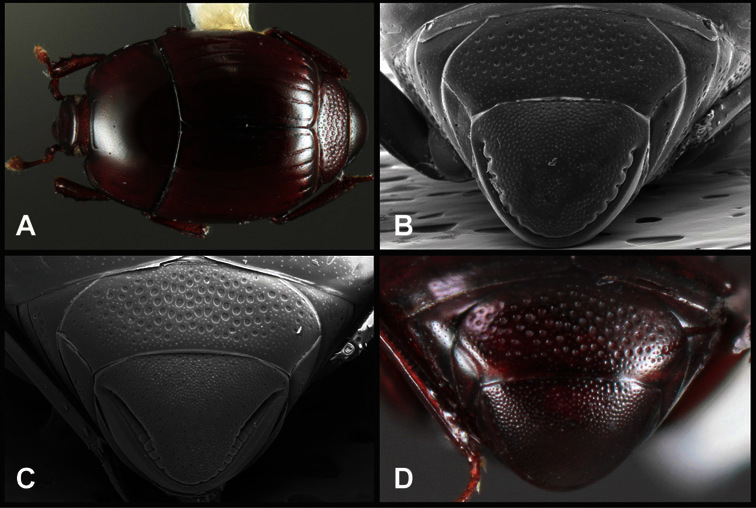
*Operclipygus impuncticollis* group. **A** Dorsal habitus of *Operclipygus britannicus* **B** Pygidia of *Operclipygus impuncticollis* **C** Pygidia of *Operclipygus britannicus* **D** Pygidia of *Operclipygus bickhardti*.

These three similar species are all rather small, with a generally coarsely sulcate marginal pygidial stria, which is particularly crenulate along the inner margin (Figs 15B–D). All have their anterior submarginal pronotal stria detached and rather weakly recurved at the sides, with the median pronotal gland openings located behind the ends of this stria, one-fifth to one-fourth the pronotal length from the anterior margin. They are only separable by minor external characters, principally in details of the pygidial sulcus.

### Key to the species of the *Operclipygus impuncticollis* group

**Table:** 

1	Frontal stria interrupted at sides, central portion detached; marginal pygidial sulcus very strongly crenulate on inner edge, all the way to base (Fig. 15B)	*Operclipygus impuncticollis* (Hinton)
–	Central portion of frontal stria not detached from sides; marginal pygidial sulcus varied, but more weakly crenulate along inner edge	2
2	Marginal pygidial sulcus very strongly incised at bases, inner edge not crenulate along this basal portion (Fig. 15C)	*Operclipygus britannicus* sp. n.
–	Marginal pygidial sulcus not strongly incised at bases, inner edge distinctly crenulate to base (Fig. 15D)	*Operclipygus bickhardti* sp. n.

### Operclipygus impuncticollis

(Hinton, 1935)

http://species-id.net/wiki/Operclipygus_impuncticollis

**Map 5.**
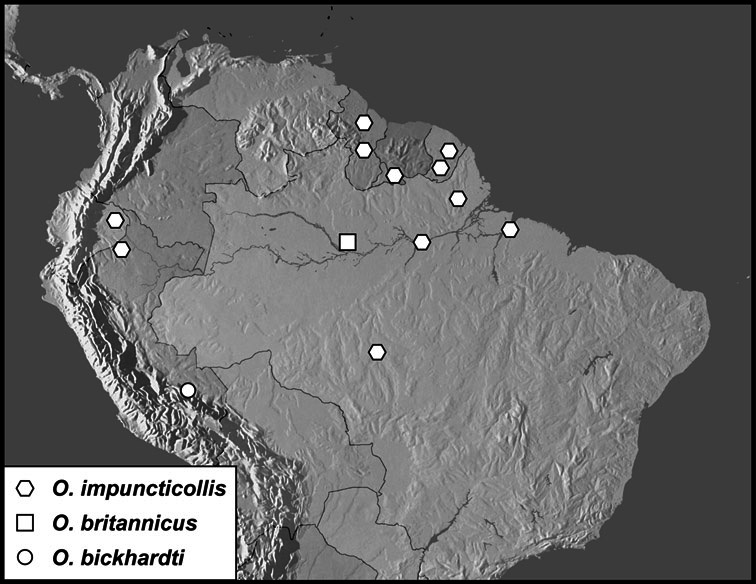
Records of the *Operclipygus impuncticollis* group.

Figs 15B 16A–D Map 5

Phelister impuncticollis Hinton, 1935a: 590; *Operclipygus impuncticollis*: Wenzel 1976: 259.

#### Type locality.

BRAZIL: Pará: Santarém [2°26'S, 54°42'W].

#### Type material.

**Lectotype**, here designated, probably male: “Santarem”/ “Type” / “*Phelister impuncticollis* type. Hntn.” / “G. Lewis Coll. B.M.1926-369” / “LECTOTYPE *Phelister impuncticollis* Hinton, 1935, M.S.Caterino & A.K.Tishechkin des. 2010” (BMNH). This species was described from an unspecified number of specimens, and the lectotype designation fixes primary type status on the only known original specimen.

#### Other material.

**BRAZIL: Amapá:** 1: Serra do Navio, 0°59'N, 52°00'W, 1.v.1991, 14.v.1991, FIT (CHND). **Mato Grosso:** 1: Cotriguaçu, Fazenda São Nicolau, 9°48.9'S, 58°17.15'W, 15.xii.2010, FIT, F.Z. Vaz-de-Mello & A.F. Oliveira (CEMT). **Pará:** 3: IPEAN, Utinga, Belém, 1°27'S, 48°26'W, ix.1984, FIT (CHND), 1: v.1985, FIT (CHND), 7: vii.1985, FIT (CHND), 2: viii.1985, FIT (CHND), 1: x.1985, FIT (CHND). 1: Tucuruí, 3°45'S, 49°40'W, v.1986, FIT (CHND), 1: 10–29.vii.1985, FIT (CHND), 1: 9–17.xii.1985, FIT (CHND). 1: Ilha Arapiuns, 2°24'S, 54°57'W, 30–31.xii.2008, FIT (CEMT). **ECUADOR: Orellana:** 1: Univ. Catolica Res. Sta., vii.1996, FIT, A. Cognato (MSCC). 1: Yasuní Res. Stn., mid.Rio Tiputini, 0°40.5'S, 76°24'W, 4.viii.1999, palm fruit/flower fall, A.K. Tishechkin (LSAM), same locality & collector except as noted: 4: 28.vi–5.vii.1999, FIT (LSAM), 1: 22–28.vi.1999, FIT (LSAM), 1: 12–20.vii.1999, FIT (LSAM), 2: 26.vii-4.viii.1999, FIT (LSAM), 1: 18–23.vi.1999, FIT (LSAM), 3: 20–26.vii.1999, FIT (LSAM, USFQ), 3: 23–30.vi.1999, FIT (LSAM), 4: 17–23.vi.1999, FIT (AKTC, LSAM), 1: 5–11.vii.1999, FIT (LSAM), 1: 11–18.vii.1999, FIT (LSAM), 1: 5–10.ix.1999, FIT, Primary forest, E.G. Riley (TAMU), 1: Berlese, 12.vii.2008, C.E. Carlton, DNA Extract MSC-1904 (LSAM); 4: Tiputini Biodiversity Station, 0.6376°S, 76.1499°W, 4–9.vi.2011, FIT, M.S. Caterino & A.K. Tishechkin (SBMNH, USFQ, MSCC), DNA Extract MSC-2117; 2: Tiputini Biodiversity Station, 0°38'0"S, 76°9'0"W, 220m, 5–25.ix.2000, D.J. Inward & K.A. Jackson (BMNH). 2: P.N. Yasuní, Via Maxus at Puente Piraña, 0°39.5'S, 76°26'W, 14–20.vii.2008, FIT, A.K. Tishechkin (AKTC). **FRENCH GUIANA:** 1: Régina, Réserve des Nouragues, 4°2.27'N, 52°40.35'W, 28.i.2010, FIT, SEAG (CHND). 1: Roura, 8.4km SSE, 4°40'41"N, 52°13'25"W, 200m, 29.v–10.vi.1997, FIT, J. Ashe & R. Brooks (CMNC). 1: Mont tabulaire Itoupé, 3°1.82'N, 53°6.40'W, 400m, 17.iii.2010, FIT, SEAG (CHND), 2: 17.iii.2010 (CHND), 1: 24.iii.2010 (CHND). **GUYANA: Mazaruni-Potaro**: 1, Takutu Mountains, 6°15'N, 58°55'W, 10–19.xii.1983, Window trap, montane rainforest near logging area, P.D. Perkins & W.E. Steiner (USNM), 2: same data except 8.xii.1983 (USNM). **Region 8**: 1: Iwokrama Forest, 26km SW Kurupukari, Iwokrama Mt., 4°20'2"N, 58°47'18"W, 300m, 23–25.v.2001, FIT, R. Brooks & Z. Falin (SEMC). **PERU: Loreto:** 1: Teniente Lopez, 2°35.66'S, 76°06.92'W, 210–240m, 25.vii.1993, flowerfall berlese, R. Leschen (SEMC), same locality & collector: 1: 21.vii.1993, palm fruit fall (SEMC), 1: 20.vii.1993, FIT (SEMC), 1: 26.vii.1993, FIT (SEMC), 1: 11.vii.1993, FIT (SEMC). **SURINAME: Sipaliwini:** 1: CI-RAP Surv. Camp 1 on Kutari River, 2°10.521'N, 56°47.244'W, 228m, 19–24.viii.2010, FIT, T. Larsen & A.E.Z. Short (SEMC).

#### Diagnostic description.

**Figure 16.**
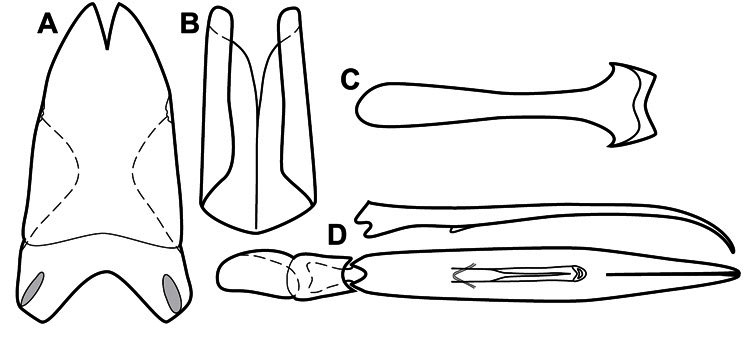
Male genitalia of *Operclipygus impuncticollis*. **A** T8 **B** S8 **C** S9 **D** Aedeagus, lateral and dorsal views, of *Operclipygus impuncticollis*.

Length: 1.62–2.15 mm, width: 1.44–1.84 mm; body rufescent, rounded, moderately convex; frons flat, very finely and sparsely punctate; frontal stria interrupted over antennal bases, rarely complete; epistoma notably convex near apex, weakly emarginate apically; labrum short, almost three times as wide as long; left mandible untoothed, right with acute basal tooth; pronotum with sides fairly straight, convergent in basal two-thirds, curved inward to anterior angles; pronotal disk (and elytral bases) flattened near scutellum, with faint, linear prescutellar impression, pronotal ground punctation very fine, sparse, some individuals with <5 coarser punctures at sides; lateral marginal pronotal stria broadly interrupted behind head; lateral submarginal stria complete, incurved anteriorly, ending freely behind eye; anterior submarginal stria close to anterior margin, crenulate, barely or not recurved behind eye; median pronotal gland openings situated beyond apices of recurved anterior stria, about one-fourth pronotal length from anterior margin; elytra with single complete, strongly sinuate epipleural stria, outer subhumeral stria present in apical third, inner subhumeral absent, striae 1-3 complete, 4^th^ present in apical half, 5th stria present in about apical third, sutural stria present in apical two-thirds, all elytral striae, especially sutural, markedly wider anterad; prosternal keel with base broad, weakly, arcuately produced, carinal striae slightly abbreviated in front, united anteriorly; prosternal lobe rather short, marginal stria abbreviated at sides; anterior mesoventral margin broadly, shallowly emarginate, marginal mesoventral stria complete; mesometaventral stria arched forward at middle, sinuate near mesocoxa, extending obliquely toward outer corner of metacoxa, slightly abbreviated at apex; metaventral disk finely punctate; 1^st^ abdominal ventrite with inner lateral stria displaced mediad, abbreviated, with broad gap between it and more nearly complete outer lateral stria; propygidium relatively long, about two-thirds pygidium length along midline, with medium punctures in middle part of disk, separated by about their diameters; pygidium with very dense, fine ground punctation and a few, slightly coarser punctures interspersed; apical sulcus deeply impressed, crenulate along inner margin, more strongly impressed towards sides, with narrow flat marginal bead.Male genitalia (Fig. 16A–D): accessory sclerites present; T8 with sides straight, weakly convergent in basal two-thirds, angled inward to narrow apex, desclerotized at angle, basal emargination shallowly triangular, basal apodemes obliquely truncate, basal membrane attachment line distad apex of basal emargination by almost its length, ventrolateral apodemes fairly narrowly rounded, not meeting beneath; S8 elongate, weakly narrowed to apex, apical guides evenly and narrowly developed throughout length, apices rounded, ventral halves approximate (possibly fused) at base, divergent and well separated in apical three-fourths; T9 with basal apodemes parallel-sided, sides slightly widened at basal third, convergent in apical two-thirds; apices narrow, subacute, convergent; T10 with halves separate; S9 with stem narrow, weakly widened to narrowly rounded base, head rather large, with lateral flanges strongly elevated, weakly convergent, apical margin narrowly arcuate, but lacking median emargination, apical flange well developed, continuous; tegmen narrow, elongate, with sides weakly rounded, widest in basal third, narrowing to apex, apex weakly hooked ventrad; medioventral process narrowly ‘U’-shaped, weakly projecting beneath about one-fourth from base; basal piece slightly over one-third tegmen length; median lobe about one-third tegmen length with basal apodemes barely separate, divergent at tips, filamentous part inconspicuous.

#### Remarks.

The nominate species of the *Operclipygus impuncticollis* group is distinguished from the other two by its usually interrupted frontal stria, its lack of coarse lateral pronotal punctures, and its marginal pygidial sulcus which appears as a series of very deep, coarse punctures all the way to the basal corners (Fig. 15B).

### Operclipygus britannicus sp. n.

urn:lsid:zoobank.org:act:9BD26FC5-F60D-4145-BBD2-04580DB61EE0

http://species-id.net/wiki/Operclipygus_britannicus

Figs 15A, C Map 5

#### Type locality.

BRAZIL: Amazonas: Manaus, INPA/Smithsonian Reserve [2°25'S, 59°50'W].

#### Type material.

**Holotype male**: “BRAZIL: Manaus, AM.INPA/Smithsonian Res. 2°25'S, 59°50'W R. Didham.v.1994” / “Leaf litter, Winkler method. Terra firme fst” / “889 | 1” / “Caterino/Tishechkin Exosternini Voucher EXO-00251” (BMNH). **Paratypes** (4): 1: same data as type (BMNH), same data as type, except as noted: 1: iii.1994 (FMNH), 1: v.1994 (MSCC). **BRAZIL: Amazonas:** 1: Reserva Ducke, 26km NE Manaus, iii.1995, leaf litter, M.G.V.Barbosa (BMNH).

#### Diagnostic description.

This species is extremely similar to *Operclipygus impuncticollis*, differing only in a few external characters: length: 1.72–2.15 mm, width: 1.47–1.84 mm; frontal stria complete; marginal pygidial sulcus much more strongly and broadly, but smoothly impressed in basal half, inner edge not obviously crenulate basally; male genitalia indistinguishable from those of *Operclipygus impuncticollis*.

#### Remarks.

The basally deep pygidial sulcus (Fig. 15C) is the best character for separating this species from *Operclipygus impuncticollis*.

#### Etymology.

This species is named to recognize the contributions of the Natural History Museum, London, to this study. All the specimens of the type series of this species were found among this museum's collections, and the museum hosted the senior author for a two year postdoc in 1999–2001 that continues to pay dividends.

### Operclipygus bickhardti sp. n.

urn:lsid:zoobank.org:act:5E35C303-0539-401C-B156-3508244C9D82

http://species-id.net/wiki/Operclipygus_bickhardti

Fig. 15D Map 5

#### Type locality.

PERU: Madre de Dios: Pantiacolla Lodge on Alto Madre de Dios River [12°39.3'S, 71°13.9'W].

#### Type material.

**Holotype male**: “PERU: Madre de Dios, Pantiacolla Lodge, Alto Madre de Dios River, 400m 12°39'22"S, 71°13'55"W 23–26 OCT 2000; R.Brooks PERU1B00 099 ex: flight intercept trap” / “SM0263092 KUNHM-ENT” (SEMC).

#### Diagnostic description.

This species is extremely similar to *Operclipygus impuncticollis* and *Operclipygus britannicus*, differing only in a few external characters: length: 1.68 mm, width: 1.40 mm; frontal stria complete; median pronotal gland openings nearer anterior pronotal margin, only about one-fifth pronotal length behind; lateral metaventral stria curved laterad toward metepisternum at apex; 1^st^ abdominal ventrite with inner lateral stria very short, straight, displaced far mediad; pygidial sulcus much less strongly impressed, its depth even around apical margin, rather finely crenulate along inner edge, not more strongly impressed towards basal corners; male genitalia indistinguishable from those of *Operclipygus impuncticollis*.

#### Remarks.

The short and displaced inner lateral stria of the 1^st^ abdominal ventrite, and the rather finely crenulate marginal pygidial sulcus (Fig. 15D) are adequate to distinguish this species.

#### Etymology.

We name this species in honor of Heinrich von Bickhardt (1873–1920), the German histerid specialist who established the tribe Exosternini, and many of its species.

### *Operclipygus panamensis* group

**Figure 17.**
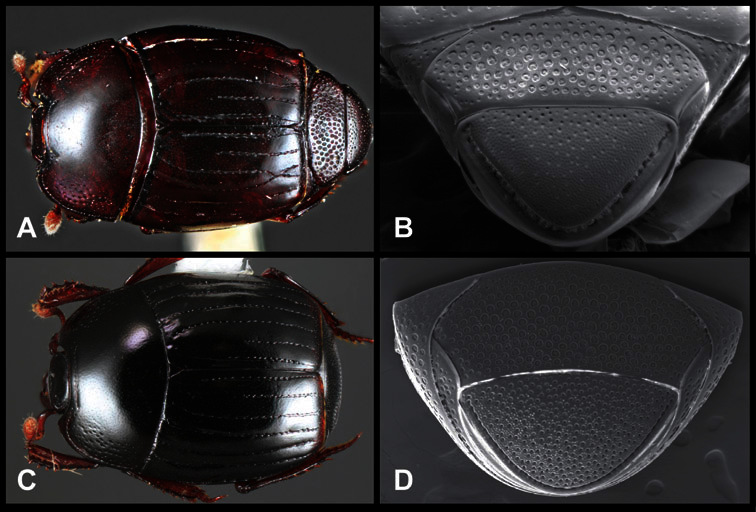
*Operclipygus panamensis* group. **A** Dorsal habitus *Operclipygus panamensis* (holotype) **B** Pygidia of *Operclipygus panamensis* **C** Dorsal habitus *Operclipygus crenatus* **D** Pygidia of *Operclipygus crenatus*.

**Figure 18.**
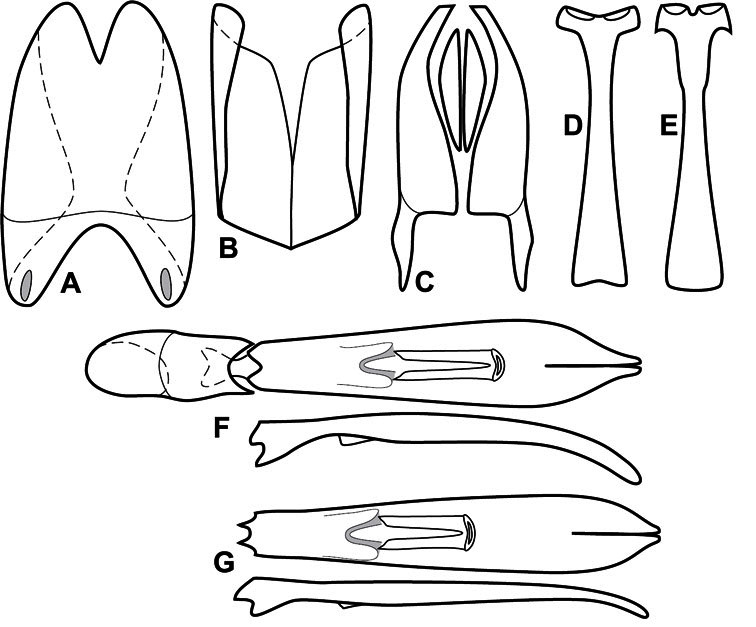
Male genitalia of *Operclipygus panamensis* group. **A** T8 of *Operclipygus panamensis* **B** S8 of *Operclipygus panamensis* **C** T9 & T10 of *Operclipygus panamensis* **D** S9 of *Operclipygus panamensis* **E** S9 of *Operclipygus crenatus* **F** Aedeagus, dorsal and lateral views, of *Operclipygus panamensis* **G** Aedeagus, dorsal and lateral views, of *Operclipygus crenatus*.

The *Operclipygus panamensis* group contains two common and closely related species, *Operclipygus panamensis* and *Operclipygus crenatus*. Externally they are very similar, with an elongate, subdepressed body, having the median portion of frontal stria detached from lateral portions (although varying in both species from complete to absent across front); anterior edge of the pronotum weakly to strongly projecting (Figs 17A, C); outer subhumeral elytral stria nearly complete, striae 1-4 complete; pygidium with dense, fine ground punctation and coarser punctures interspersed, and a complete marginal sulcus (differing in depth between the two) (Figs 17B, D). They are not keyed together in the main key to species groups due to rather significant variation in many external characters. Their male genitalia (Fig. 18) are characterized by: accessory sclerites present; T8 with deep basal and apical emarginations, with ventrolateral apodemes originating near proximal end, most strongly developed basad of midpoint, well separated along midline; S8 with apical guides gradually more developed to apex, lateral halves approximate basally, weakly divergent in apical half; T9 with apices narrow, truncate, with acute inner points; T10 elongate, divided; S9 relatively narrow and elongate, with shallow apical emargination and separate, moderately well developed apical flanges; aedeagus widest beyond midpoint, slightly to strongly attenuate near apex, medioventral process well developed, narrow but rounded situated about one-third from base, projecting strongly beneath; median lobe about one-third tegmen length. They are generally quite similar to members of the *Operclipygus sejunctus* and *Operclipygus kerga* groups, differing from the former by their lack of metaventral punctures, and from the latter by the depressed frons and more deeply impressed pygidial sulcus.

### Key to the species of the *Operclipygus panamensis* group

**Table:** 

1	Central portion of anterior pronotal margin distinctly outwardly arcuate behind head (Fig. 17A); anterior submarginal pronotal stria detached from lateral portions of stria; body rufo-brunneus	*Operclipygus panamensis* (Wenzel & Dybas)
–	Central portion of anterior pronotal margin not strongly arcuate behind head, at most very faintly projecting at middle (Fig. 17C); anterior submarginal pronotal stria connected to lateral submarginal striae at postocular angle; body generally darker, rufo-piceous	*Operclipygus crenatus* (Lewis)

### Operclipygus panamensis

(Wenzel & Dybas, 1941)

http://species-id.net/wiki/Operclipygus_panamensis

**Map 6.**
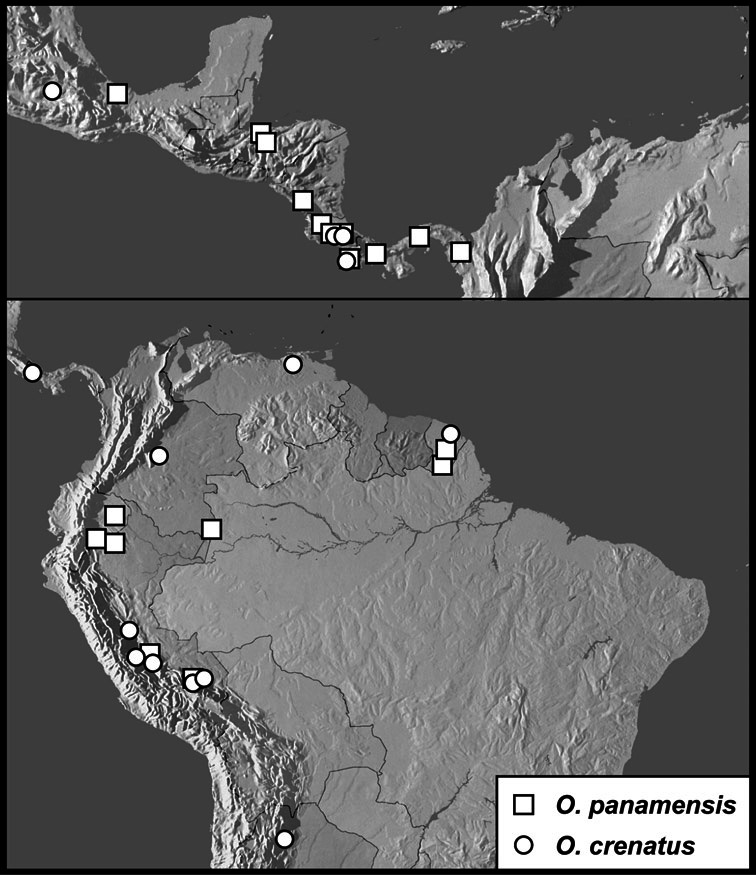
Records of the *Operclipygus panamensis* group.

Figs 17A–B 18A–D, F Map 6

Phelisteroides panamensis Wenzel & Dybas, 1941: 450; *Operclipygus panamensis*: Wenzel 1976: 259.

#### Type locality.

PANAMA: Panamá: Barro Colorado Island [9°11'N, 79°51'W].

#### Type material.

**Holotype male** (FMNH): “Barro Clrdo. Is. Panamá, C.Z., 9-14-38 1, 211” / ”Coll. by E.C. Williams”/”pres. by R.L. Wenzel”; examined 2006.

#### Other material.

**COLOMBIA: Vaupés:** 9: Parque Nac. Mosiro-Itajura (Caparú), Centro Ambiental, 1°04'S, 69°31'W, 60m, 20–30.i.2003, FIT, D. Arias & M. Sharkey (IAVH, FMNH, MSCC, AKTC). **COSTA RICA: Alajuela:** 22: Eladio's, Peñas Blancas Trail, 875m, 19.v.1989, FIT, J. Ashe, R. Brooks, R. Leschen (SEMC)**;** same data except as noted: 25: 800m, 19.v.1989, FIT (SEMC), 1: 1190m, 20.v.1989, FIT (SEMC), 1: 970m, 20.v.1989, FIT (SEMC). **Cartago:** 1: Parque Nac. Barbilla, R.F. Rio Pacuare. Turrialba, Send. Rio Danto, 500–600m, 28.viii–5.ix.2001, FIT, W. Arana (INBIO); same data except as noted: 1:800m despues del R. Dantas, 400m, 19.x–2.xi.2000, FIT, W. Arana (INBIO). **Guanacaste:** 1: Parque Nac. Guanacaste, 9km S Santa Cecilia, Est. Pitilla, 700m, ii.1990, C. Moraga & R. Blanco, (INBIO), 2: ix.1991, (INBIO), 2: Est. Pitilla, 10°59'22"N, 85°25'33"W, 610m, 13–15.vii.2000, FIT, J. Ashe, R. Brooks, Z. Falin (SEMC); 1: Rio San Lorenzo, Tierras Morenas, Z.P. Tenorio, 1050m, i.1993, G. Rodriguez, (INBIO). **Heredia:** 6: La Selva Biol. Stn., 10°26'N, 84°01'W, 19.vi.1998, FIT, C.E. Carlton & A.K. Tishechkin (LSAM); same data except as noted: 2: 20.vi.1998, FIT (LSAM), 1: 21.vi.1998, FIT (LSAM), 10: 22.vi.1998, FIT (LSAM), 2: 23.vi.1998, FIT (LSAM), 2: 24.vi.1998, FIT (LSAM), 1: 26.vi.1998, FIT (LSAM), 2: 29.vi.1998, FIT (LSAM), 1: 50–150m, 5–8.iii.2001, FIT, E.G. Riley (TAMU), 2: 6.vii.2005, FIT, M. Ferro (LSAM), 2: 23.vii.1976, H.A. Hespenheide, (INBIO), 1: 100m, 11.iii.1992, FIT, W. Bell (SEMC), 1: 10.vi.2012, OTS Beetle Course, DNA Extract MSC-2306 (AKTC). **Limón:** 2: Area Cons. Tortuguero, Sector Cerro Cocori, Fca. de E. Rojas, 150m, i.1993, E. Rojas, (INBIO), 4: ix.1991, E. Rojas, (INBIO), 3: viii.1991, E. Rojas, (INBIO), 2: Parque Nac. Tortuguero, Est. Cuatro Esquinas, 0m, v.1990, J. Solano, (INBIO), 2: vi.1990, J. Solano, (INBIO), 1: v.1990, (INBIO); 1: Res. Biol. Hitoy Cerere, send. Plano Espavel, 180m, 24.iii.2002, Red de Golpe, W. Arana (INBIO); 1: Sardinas, Barra del Colorado, 15m, 28.i-12.ii.1995, F. Araya, (INBIO). **Puntarenas:** 1: Est. Biol. Las Cruces, 8°47.14'N, 82°57.58'W, 1330m, 28–30.v.2004, FIT, J.S. Ashe, Z. Falin, I. Hinojosa (SEMC); 1: Wilson Botanical Garden, 1200m, 27.v.1993, FIT, J.S. & A.K. Ashe (SEMC)**;** 1: Est. Esquinas, Peninsula de Osa, 0m, i.1993, F. Quesada, (INBIO), 1: iv.1993, M. Segura, (INBIO), 1: 200m, iv.1993, J.F. Quesada, (INBIO); 1: Parque Nac. Corcovado, Est. Sirena, 0–100m, i.1990, G. Fonseca, (INBIO), 1: x.1990, C. Saborio, (INBIO); 1: Corcovado Trail, 8°29'7"N, 83°34'39"W, 150m, 28.vi-1.vii.2000, FIT, Z. Falin (SEMC); 2: upper Ollas Trail, 8°29'7"N, 83°34'39"W, 140m, 24–28.vi.2000, FIT, Z. Falin (SEMC); 1: Rancho Quemado, Peninsula de Osa, 200m, v.1991, F. Quesada, (INBIO), 3: v.1991, J.C. Saborio, (INBIO), 2: v.1992, F. Quesada y G. Varela, (INBIO); 1: Res. Biol. Carara, Est. Quebrada Bonita, 100m, i.1995, R. Guzman, (INBIO); 2: Laguna Meandrica, 100m, vi.1990, R. Zuniga, (INBIO); 1: Reserva Forestal Golfo Dulce, Est. Agujas, Golfito, 250–350m, 26.v.2000, FIT, A. Azofeifa (INBIO); 1: Rincon de Osa, 8°41.141'N, 83°31.117'W, 150m, 23–26.vi.2001, FIT, S. & J. Peck (SEMC), 2: 40m, 23–26.vi.2001, FIT, stream side, S. & J. Peck (SEMC). **ECUADOR: Orellana:** 1:Parque Nac. Yasuní, Via Maxus at Puente Piraña, 0°39.5'S, 76°26'W, 14–20.vii.2008, FIT, A.K. Tishechkin (AKTC), 1: 24.vii.2008, FIT, A.K. Tishechkin (AKTC); 2: Tiputini Biodiversity Station, 0.6376°S, 76.1499°W, 2–9.vi.2011, FIT, M.S. Caterino & A.K. Tishechkin (FMNH, USFQ). **Pastaza:** 1: Cushuime, on Rio Cushuime, 150km SE Puyo, 320m, 19.v.1971, polypore fungus, B. Malkin (FMNH). **FRENCH GUIANA:** 1: Mont tabulaire, Itoupé, 3°1.38'N, 53°5.73'W, 570m, 24.iii.2010, FIT, SEAG (CHND); 1: Montagne des Chevaux, 4°43'N, 52°24'W, 18.iv.2009, FIT, SEAG (CHND). **HONDURAS: Cortés:** 1: Lago Yojoa, 650m, 23–28.viii.1994, FIT, tropical, evergreen forest, S. & J. Peck (CMNC); 3: Parque Nac. Cerro Azul-Meambar, Los Pinos, 14°52.4'N, 87°54.7'W, 800m, 10–16.v.2001, FIT, S. Peck (SEMC); 4: Yojoa Lake, Deer Island, 14°55'N, 87°58'W, 670m, 19–21.vi.1994, FIT, J. Ashe, R. Brooks (SEMC), 13: 22–26.vi.1994, FIT, J. Ashe, R. Brooks (SEMC); **Santa Barbara**: 1: La Fe, Finca La Roca 5.3km S. Pena Blanca, 14°57'N, 88°02'W, 740m, 19–21.vi.1994, FIT, J.S. Ashe & R. Brooks (SEMC). **MEXICO: Veracruz**: 1: Cordoba, 4.viii.1969, tropical evergreen forest Berlese, S. Peck (FMNH). **NICARAGUA**: **Granada**: 3: Res. Nat. Volcan Mombacho, 11°50'N, 85°58.8'W, 1150m, 2–5.vi.2002, FIT, elfin montane forest, S. Peck (CMNC); 2: Volcan Mombacho, El Progreso #2, 30.vi.1998, malaise trap, J.M. Maes (MEL); **Rio San Juan**: 1: Refugio Bartola, 60km SE San Carlos, 10°58.40'N, 84°20.30'W, 100m, 28–30.v.2002, FIT, Z. Falin, S. Chatzimanolis (SEMC), 3: 30m, 23–31.v.2002, FIT, S. Peck (SEMC). **PANAMA: Chiriquí:** 1: La Fortuna, “Cont. Divide Trail”, 8°46'N, 82°12'W, 1150m, 23.v–9.vi.1995, FIT, J. Ashe, R. Brooks (SEMC); 1: La Fortuna, “Hydro. Trail”, 8°42'N, 82°14'W, 1150m, 21–23.v.1995, FIT, J. & A. Ashe (SEMC); 1: Reserva La Fortuna, 8°43'18"N, 82°14'17"W, 3900ft, 4–10.viii.1999, FIT, Schaffner & Woolley (TAMU); **Colón:** 40: San Lorenzo Forest, 9°17'N, 79°58'W, 11.v.2004, 12.v.2004, FIT, A.K. Tishechkin (LSAM)**,** 18: 12–13.v.2004 (LSAM)**,** 10: 13–14.v.2004 (GBFM, LSAM)**,** 10: 14–15.v.2004 (LSAM)**,** 2: 15–17.v.2004 (LSAM), 6: 18–19.v.2004 (GBFM, LSAM)**,** 5: 19–20.v.2004 (LSAM)**,** 1: 20–21.v.2004 (LSAM), 1: 28–30.ix.2003 (LSAM), 2: 21–24.v.2004 (LSAM)**,** 1: 24–25.v.2004 (LSAM), 2: 25–26.v.2004 (LSAM)**,** 1: 26–29.v.2004 (LSAM), 4: 29.v.2004 (LSAM), 1: 12–13.v.2004 (LSAM)**,** 5: 12–23.ix.2004, M. Rapp (LSAM)**,** 1: 12–19.v.2004, R. Didham (LSAM)**,** 5: 14–26.vii.2004, M. Rapp (LSAM)**,** 10: 15–25.v.2004, R. Didham (LSAM)**,** 2: 23.x–2.xi.2004, M. Rapp (LSAM)**,** 5: 24.vi-4.vii.2004, M. Rapp (GBFM, LSAM)**,** 3: 25.v–4.vi.2004, R. Didham (LSAM)**,** 2: 25.v–5.vi.2004, R. Didham (LSAM)**,** 2: 25.vi-5.vii.2004, M. Rapp (LSAM)**,** 1: 3–13.viii.2004, M. Rapp (LSAM)**,** 3: 3–13.x.2004, M. Rapp (LSAM)**,** 2: 4–17.vi.2004, M. Rapp (LSAM)**,** 1: 4–14.viii.2004, M. Rapp (LSAM)**,** 4: 5–18.vi.2004, M. Rapp (LSAM)**,** 1: 1.3m, 25.v–5.vi.2004, R. Didham (LSAM)**,** 1: 14m, 25.iv-5.v.2004, M. Gonzalez (LSAM)**,** 1: 7m, 12–23.ix.2004, M. Rapp (LSAM); **Darién:** 1: Cana Biological Station, 7°45'18"N, 77°41'6"W, 600m, 7–9.vi.1996, FIT, J. Ashe, R. Brooks (SEMC); **Panamá**: 3: Barro Colorado Isl., 9°11'N, 79°51'W, 1.viii.1994, FIT, D. Banks**,** 1: 16.viii.1994, FIT, D. Banks**,** 1: 21–22.vii.2000, FIT, S. Chatzimanolis (SEMC), 1: 24.viii.1994, D. Banks**,** 1: 29.vii.1994, D. Banks**,** 2: 6.viii.1994, D. Banks**,** 1: 7.vii.1994, D. Banks**,** 3: 8.vii.1994, D. Banks**,** 1: Cano Saddle Gatun Lake, Close's Pltn, 12.v.1923, RC Shannon (USNM); 1: Cerro Azul, ca., 2000ft, 21.ii.1976, flood debris, A.F. Newton (FMNH); 1: Pipeline Rd., 17–22.vi.1993, FIT, S. Lingafelter (SEMC); **PERU: Cusco:** 1: Consuelo, Manu rd. km 165, 6–7.x.1982, FIT, L. Watrous & G. Mazurek (FMNH); 1: 6.x.1982, rotten palm, L. Watrous & G. Mazurek (FMNH); 1: 7–8.x.1982, FIT, L. Watrous & G. Mazurek (FMNH); **Junín:** 2: 11km NE Puerto Ocopa, Los Olivos, 11°7.00'S, 74°15.52'W, 1200m, 26–28.iii.2009, Window trap, A.V. Petrov (AKTC, MUSM); **Loreto:** 4: 1.5km N Teniente Lopez, 2°35.66'S, 76°06.92'W, 210–240m, 18.vii.1993, FIT, R. Leschen (SEMC), 5: 20.vii.1993, FIT, R. Leschen (SEMC); 1: 68km SW Iquitos to Nauta, Rio Itaya, 4°11'S, 73°26'W, 110m, 18.ii.2008, A.V. Petrov (AKTC); 2: km 63, rd. Iquitos-Nauta, Rio Itaya, 4°15.205'S, 73°26.074'W, 140m, 10–14.ii.2010, A.V. Petrov (AKTC), 1: 9–13.i.2011, A.V. Petrov (MUSM); **Madre de Dios:** 1: Manu National Park, Pantiacolla Lodge, Alto Madre de Dios River, 12°39.3'S, 71°13.9'W, 420m, 14–19.xi.2007, FIT, D. Brzoska (SEMC).

#### Diagnosis.

Length: 2.18–2.84 mm, width: 1.75–2.37 mm; body rufo-brunneus; frons with median portion of frontal stria well impressed; supraorbital stria briefly interrupted at vertex; pronotum with anterior submarginal stria detached from lateral portion, slightly recurved posterad at apices, more or less parallel to outwardly arcuate anterior emargination, anterior marginal stria interrupted for short distance at middle; elytra generally lacking any fragments of inner subhumeral stria, sutural stria nearly or fully complete, frequently with the 4^th^ stria arched over at the base nearly meeting it; pygidium with punctation mainly fine, dense, with larger punctures interspersed only near the base, marginal sulcus complete, deep, crenulate. Male genitalia (Figs 18A–D, F): T8 with unique secondary sclerotization along edge of apical emargination; S9 narrow, evenly expanded to apex, with midline sclerotization distinct; tegmen abruptly narrowed toward apex, strongly narrowed and slightly bent near base in lateral aspect.

#### Remarks.

The best characters for separating *Operclipygus panamensis* and *Operclipygus crenatus* are the details of the anterior pronotal margin and submarginal stria, with the central portion of the pronotal margin outwardly arcuate in *Operclipygus panamensis* (Fig. 17A), and the anterior submarginal stria always detached from the lateral submarginal. In *Operclipygus crenatus*, the anterior margin is not outwardly arcuate (Fig. 17C), and the lateral and anterior portions of the submarginal pronotal stria are usually connected behind the median pronotal gland openings. The body color and pygidium also fairly readily distinguish the species, with *Operclipygus crenatus* consistently darker (Fig. 17C), and with a more coarsely punctate pygidium (Fig. 17D).

### Operclipygus crenatus

(Lewis, 1888)

http://species-id.net/wiki/Operclipygus_crenatus

Figs 17C–D 18E, G Map 6

Phelister crenatus Lewis, 1888: 192; Wenzel (1976: 257); *Operclipygus crenatus*: Wenzel (1976: 257).
Phelisteroides miladae Wenzel & Dybas, 1941: 450; *Operclipygus miladae*: Wenzel (1976: 257). **syn. n.**
Pseudister propygidialis Hinton, 1935e: 12; *Phelisteroides propygidialis*: Wenzel and Dybas (1941: 454). *Operclipygus propygidialis*: Wenzel (1976: 257). **syn. n.**

#### Type locality.

PANAMA: Chiriquí: Barú Volcano [8°48'N, 82°32.5'W].

#### Type material.

***Phelister crenatus***: **Lectotype**, here designated, probably male: “Sp. figured”/”V. de Chiriquí, 3–4000 ft. Champion” / “LECTOTYPE *Phelister crenatus* Lewis 1864, M.S.Caterino & A.K.Tishechkin des. 2010” (BMNH). This species was described from an unspecified number of specimens, and the lectotype designation fixes primary type status on the only known original specimen.***Phelisteroides miladae***: **Holotype** female (FMNH): “Villavicencio, Meta, Colomb. VII:25:38”/”under rotten bark”/”Coll. by H. Dybas”/”Type *Phelisteroides miladae* Wenz. & Dybas”; examined 2006.***Pseudister propygidialis***: Type repository unknown; published type locality: Morelos, Cuernevaca, Mexico.

#### Other material.

**ARGENTINA: Jujuy:** 1: Parque Nac. Calilegua, Aguas Negras, 440m, 18–28.xii.1987, malaise/FIT, S. & J. Peck (CHSM); 2: Parque Nac. Calilegua, El Cortaderal, km6, 800m, 18–28.xii.1987, malaise/FIT, forest, S. & J. Peck (CHSM). **COSTA RICA: Puntarenas:** 1: Parque Nac. Corcovado, Sirena Stn. Corcovado Trail, 8°29'7"N, 83°34'39"W, 150m, 28.vi–1.vii.2000, FIT, Z. Falin (SEMC); 1: Rancho Quemado, Peninsula de Osa, 200m, v.1992, F. Quesada y G. Varela, (INBIO), 1: xii.1991, F. Quesada, (INBIO); 1: Est. Agujas, Sendero Ajo, 300m, 24–26.ix.1996, in fruit, A. Azofeifa, (INBIO). **Cartago:** 1: Ref. Nac. Fauna Silv. Tapanti, Quebrada Segunda, 1250m, iii.1992, G. Mora, (INBIO); same data except as noted: 1: ix.1992, (INBIO); 5: v.1992 (INBIO), 1: v.1993, (INBIO), 1: 1250m, vi.1992, (INBIO), 20: 1250m, vii.1992, (INBIO), 2: 1250m, x.1992, (INBIO), 5: 1300m, vi.1992, (INBIO), 1: Grano de Oro, Chirripo, Turrialba, 1120m, iii.1993, P. Campos, (INBIO). **FRENCH GUIANA:** 1: Montagne des Chevaux, 4°43'N, 52°24'W, 11.x.2009, FIT, SEAG (CHND). **PERU: Cusco:** 1: Consuelo, Manu rd. km 165, 11.x.1982, palm litter, L. Watrous & G. Mazurek (FMNH); same data except as noted: 5: 3.x.1982 (FMNH), 1: 5.x.1982 (FMNH), 2: 8.x.1982 (FMNH). **Huanuco**: 1:Tingo Maria region, 6–14.vi.1987, F. Woytkowski (SEMC). **Junín:** 3: La Merced, 9.5km NE La Merced-Villa Rica Rd., 10°58'42"S, 75°18'18"W, 880m, 15–21.x.1999, FIT, R. Brooks (SEMC); 1: 7.5km NE Puerto Ocopa, Cananeden, 11°4.9'S, 74°16.1'W, 1180m, 5–9.i.2007, A.V. Petrov (AKTC). **Madre de Dios:** 1:Manu National Park, Rio Alto Madre de Dios, Pantiacolla Lodge, 12°39.3'S, 71°13.9'W, 14–19.xi.2007, FIT, D. Brzoska (SEMC). **VENEZUELA: Monagas:** 3: Caripe, Cueva Guácharo, 700m, 20–30.vii.1987, malaise/FIT, forest over coffee, S. & J. Peck (FMNH).

#### Diagnosis.

Length: 2.25–2.62 mm, width: 1.93–2.28 mm; body piceous; median portion of frontal stria weaker, occasionally absent across middle; supraorbital stria complete across vertex; anterior submarginal pronotal stria generally attached to lateral submarginal stria (angulate behind median pronotal gland opening); anterior emargination of pronotum barely or not outwardly angulate, with a complete marginal stria; elytra with a few fragments of inner subhumeral stria generally present, sutural stria from two-thirds to three-quarters complete; pygidium with fine, dense ground punctation and with coarser secondary punctures uniformly interspersed, apical sulcus complete, crenulate, shallower than in *Operclipygus panamensis*. Male genitalia (Figs 18E, G): T8 lacking unique secondary sclerotization along edge of apical emargination; S9 slightly broader, abruptly expanded near apex, with midline sclerotization vague; tegmen evenly rounded, narrowing toward apex, narrowed but more or less straight to near apex in lateral aspect.

#### Remarks.

See above for characters distinguishing *Operclipygus crenatus* from *Operclipygus panamensis*. While their distributions overlap, and *Operclipygus crenatus* is very widespread, it is curious that, in Panama, *Operclipygus crenatus* is only known from the type locality.

Wenzel and Dybas's (1941) *Operclipygus miladae* was not compared with other species in the original description, and at the time *Operclipygus crenatus* was still included in the genus *Phelister*. However, in Wenzel's (1976) key, the characters presented do not allow their consistent separation, and the variation seen in *Operclipygus crenatus* fully encompasses the states found in *Operclipygus miladae*. The proposed synonymy of *Pseudister propygidialis* here is admittedly more tentative, in the absence of a type specimen. However, Hinton's description and figure suggest a close match, and specimens identified as *Operclipygus propygidialis* in various collections are clearly *Operclipygus crenatus*. Hopefully the type specimen will eventually surface and this can be confirmed.

### *Operclipygus sejunctus* group

**Figure 19.**
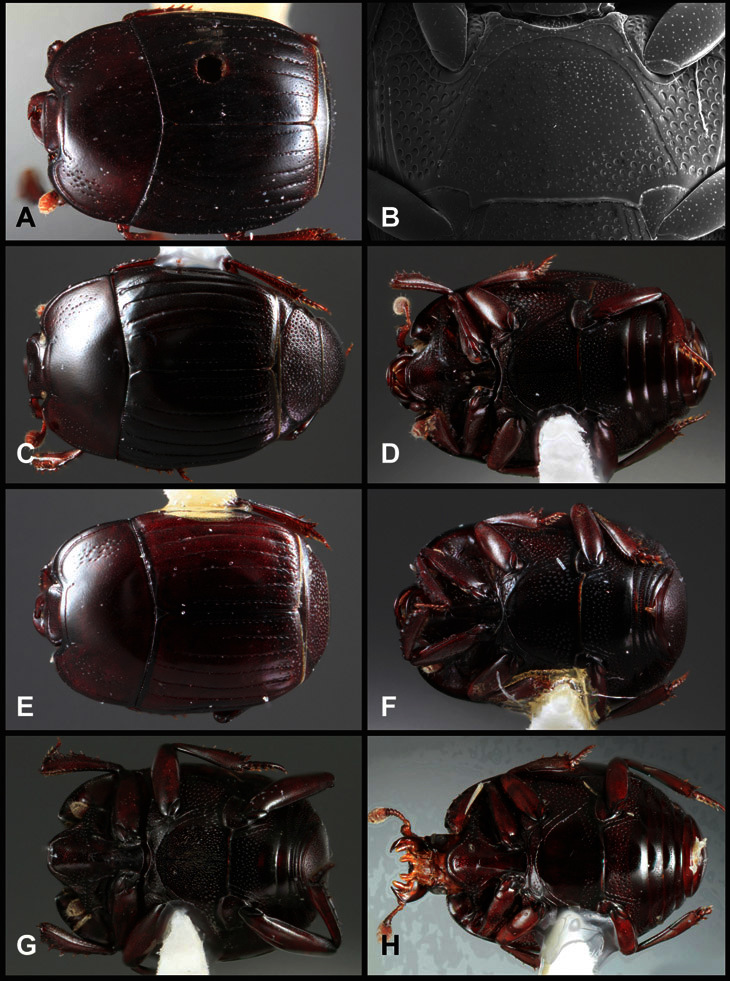
*Operclipygus sejunctus* group. **A** Dorsal habitus of *Operclipygus sejunctus* (lectotype) **B** Metaventrite of *Operclipygus sejunctus* **C** Dorsal habitus of *Operclipygus pecki* **D** Ventral habitus of *Operclipygus pecki* **E** Dorsal habitus of *Operclipygus depressus* **F **Ventral habitus of *Operclipygus depressus* **G** Ventral habitus of *Operclipygus setiventris* **H** Ventral habitus of *Operclipygus itoupe*.

The most distinctive character of this closely related group of seven species is the presence of numerous punctures on each side of the metaventrite and first abdominal ventrite (Figs 6C, 19B, F) though these vary somewhat in extent. They also all possess a secondary lateral metasterna stria, laterad and parallel to the primary metaventral stria (Figs 19B, D, F, H). All the species are somewhat broadly oval and subdepressed, and generally have conspicuous punctures near the elytral apices, which are otherwise infrequent in *Operclipygus*. As in the *Operclipygus panamensis* group, the central portion of the anterior pronotal emargination is usually outwardly arcuate, and this may partially support a close relationship between these groups. The species within the *Operclipygus sejunctus* group vary little amongst each other, and can in most cases best be distinguished by the shape of the aedeagus. Females are especially difficult or in a few cases impossible to distinguish, and some localities represented by little material have not been resolved, particularly in highland Peru and coastal Ecuador.

### Key to the species of the *Operclipygus sejunctus* group

**Table:** 

1	Apical elytral punctures absent; metaventral punctures discretely limited to posterior one-third of metaventral disk (Fig. 19H); 4^th^ dorsal elytral stria abbreviated; aedeagus rather strongly bent ventrad near apex (Fig. 20H); Guianas	*Operclipygus itoupe* sp. n.
–	Apical elytral punctures present; aedeagus more evenly rounded to straight near apex; other characters variable	2
2	Supraorbital stria complete; apical elytral punctures mostly restricted to apical margin	3
–	Supraorbital stria interrupted; apical elytral punctures extending forward between striae up to one-third from apical margin	4
3	Outer lateral metaventral stria well developed, extending from mesometaventral suture to at least metaventral midpoint (Fig. 6C); metaventral punctures present on most of metaventral disk; mesoventrite truncate, not distinctly emarginate; aedeagus parallel-sided throughout length, rather bluntly rounded apically (Fig. 20I); Central America to extreme northwestern South America	*Operclipygus punctiventer* sp. n.
–	Outer lateral metaventral stria weaker, frequently not reaching forward to mesometaventral suture (Fig. 19B); punctures of metaventrite discretely limited to posterior half of central part of disk; mesoventrite distinctly emarginate; aedeagus narrowed in most of apical half, apex subacute (Fig. 20D); South America	*Operclipygus sejunctus* (Schmidt)
4	Marginal pronotal bead convex, widening toward front	5
–	Marginal pronotal bead not distinctly convex, similar in width throughout length	6
5	Male metaventrite depressed and setose (Fig. 19G); body broad and strongly depressed, more in male than female, but notable in both sexes; outer metaventral stria paralleling inner lateral metaventral stria along at least half its length; aedeagus short, broad, with strong medioventral process (Fig. 20D)	*Operclipygus setiventris* sp. n.
–	Metaventrite not sexually dimorphic, convex and lacking setae in both sexes; outer metaventral stria shorter, usually present as only a short fragment near middle of inner metaventral stria; aedeagus elongate, narrow, medioventral process less strong (Fig. 20G)	*Operclipygus depressus* (Hinton)
6	Dorsum faintly bicolored, with pronotum rufescent (Fig. 19C); aedeagus with apicolateral corners minutely, acutely produced (Fig. 20E); only known from a single locality in Santa Cruz, Bolivia	*Operclipygus pecki* sp. n.
–	Dorsum unicolorous; aedeagus with apex simply subacute (Fig. 20F)	*Operclipygus juninensis* sp. n.

### Operclipygus sejunctus

(Schmidt, 1896) comb. n.

http://species-id.net/wiki/Operclipygus_sejunctus

**Map 7.**
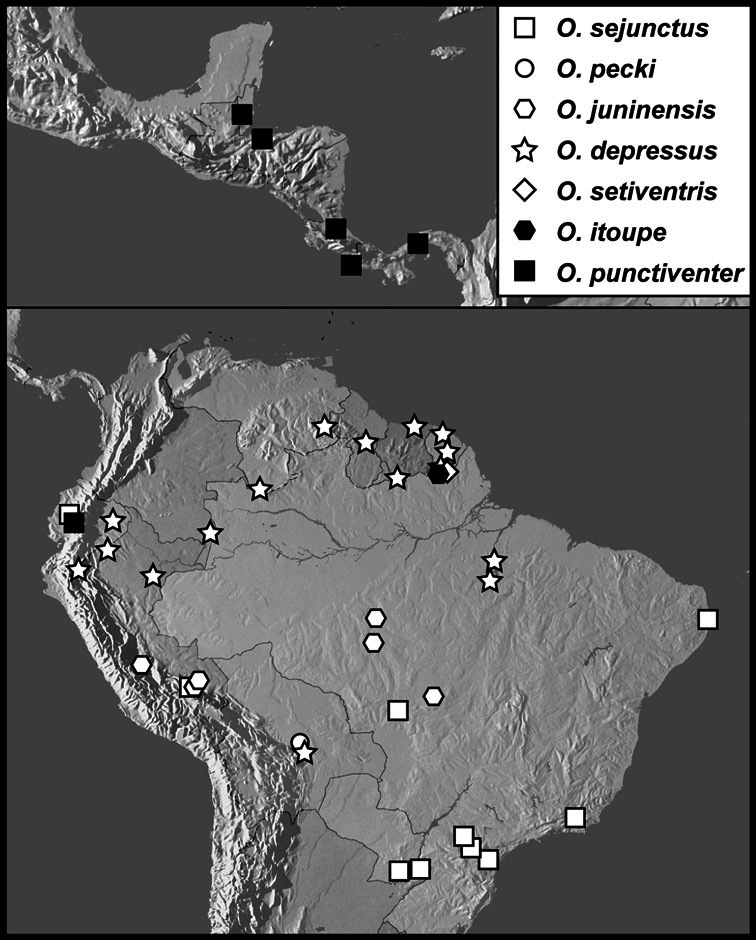
Records of the *Operclipygus sejunctus* group.

Figs 19A–B 20A–D Map 7

Phelister sejunctus Schmidt, 1896: 61; *Pseudister sejunctus*: Bickhardt (1917: 164).
Phelisteroides punctipennis Wenzel, 1944: 139; synonymized by Wenzel (1976: 257).

#### Type locality.

BRAZIL: Paraíba: Santa Rita [7°13'S, 35°2'W].

#### Type material.

***Phelister sejunctus***: **Lectotype** of undetermined sex, here designated: [Brazil: Paraíba:] “S. Rita” / “Type”[red printed] / “Type” [Schmidt's handwriting]” / “*Phelister sejunctus*” / “coll. J. Schmidt” / “LECTOTYPE *Phelister sejunctus* Schmidt, 1896, M.S.Caterino & A.K.Tishechkin des. 2010“ (ZMHB).This species was described from an unspecified number of specimens, and the lectotype designation fixes primary type status on the only known original specimen. ***Phelisteroides punctipennis*: Holotype:** “Chapada Brazil Acc. No 2966” / “Nov.” / “Type *Phelisteroides punctipennis* Wenzel” (CMNH), examined 2012.

#### Other material.

**ARGENTINA: Misiones:** 1: 15km SE Puerto Iguazú, 27.xii-6.i.1991, FIT, mature forest, roadside, S. & J. Peck (FMNH); 1: Parque Nac. Iguazú, Cantera, 200m, 8.xii–6.i.1991, FIT, old gravel pit at forest edge, S. & J. Peck (FMNH). **BRAZIL: Paraná:** 1: Piraquara, Mananciais da Serra, 25°29.77'S, 48°58.90'W, 1000m, 1–8.xi.2007, FIT, P. Grossi & D. Parizotto (UFPR), 1: 10–17.x.2007, FIT, P. Grossi & D. Parizotto (CHND), 1: 2008, P. Grossi (UFPR), 1: 3–10.ix.2007, P. Grossi (UFPR); 1: Mpio. Curitiba, nr. Campina Grande do Sul, 25.2965°S, 49.0381°W, 7–10.xii.2011, FIT, F.W.T. Leivas (UFPR): DNA Extract MSC-2257; 1: Ponta Grossa, V. Velha – IAPAR, 18.x.1999, Ganho & Marinoni (UFPR); 1: Telêmaco Borba, Klabin Papel e Celulose, Fazenda Monte Alegre, 21°13'18.4"S, 50°28'06.3"W, *Araucaria angustifolia* (Bertoloni) Kuntze forest, sulcatol baited FIT, 22.ix.2006, C.A.H. Flechtmann (UNESP-IS), Telêmaco Borba, Klabin Papel e Celulose, Fazenda Monte Alegre, *Pinus taeda* L. stand, a-pinene+ethanol+sulcatol baited FIT, 9.iii.2007 (UNESP-IS); **Rio de Janeiro:** 2: Nova Friburgo, 22°16'S, 42°32'W, 26–31.x.2009, FIT (CHND). **PARAGUAY: Itapúa:** 2: Karonay, 17km W. San Rafael Reserve, 26°45'53"S, 55°50'37"W, 90–110m, 18–20.xi.2000, FIT, Z. Falin (SEMC).

#### Diagnosis.

**Figure 20.**
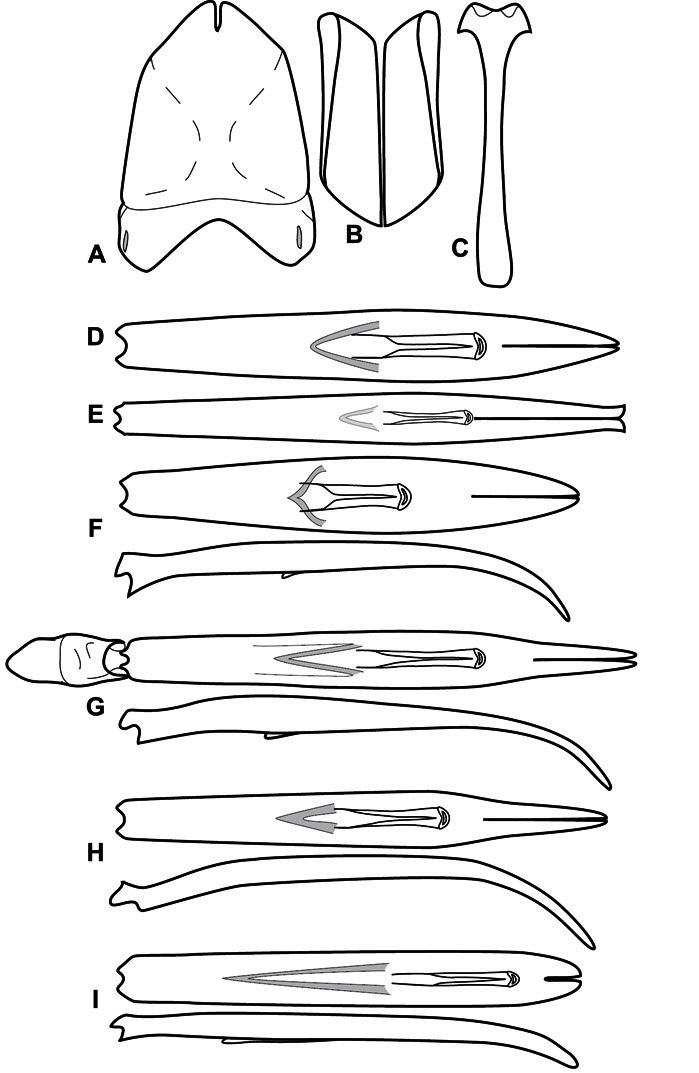
Male genitalia of *Operclipygus sejunctus* group. **A** T8 of *Operclipygus sejunctus* **B** S8 of *Operclipygus sejunctus* **C** S9 of *Operclipygus sejunctus* **D** Aedeagus, dorsal view, of *Operclipygus sejunctus* **E** Aedeagus, dorsal view, of *Operclipygus pecki* **F** Aedeagus, dorsal and lateral views, of *Operclipygus juninensis* **G** Aedeagus, dorsal and lateral views, of *Operclipygus depressus* **H** Aedeagus, dorsal and lateral views, of *Operclipygus itoupe* **I** Aedeagus, dorsal and lateral views, of *Operclipygus punctiventer*.

Length: 1.97–2.56 mm, width: 1.72–2.18 mm; body piceous; frons and epistoma very weakly depressed, with fine but conspicuous ground punctation; frontal stria variably impressed at middle, interrupted on each side, at sides continuous with complete supraorbital stria; right mandible with weak tooth, left with very weak basal tooth; pronotum broadly depressed across base, lacking discrete prescutellar impression; pronotal disk with discrete group of ~16 coarse punctures on each side; lateral submarginal pronotal stria complete, weakly crenulate; central portion of anterior pronotal emargination weakly produced; anterior marginal pronotal stria interrupted behind head; anterior submarginal pronotal stria detached, recurved slightly behind eyes, median pronotal glands near its apices, about 8 puncture widths from anterior margin. Elytra with complete inner epipleural stria, and apical half of outer epipleural stria; outer subhumeral stria present in apical half and with variable detached fragment in basal half; inner subhumeral stria nearly complete, just slightly abbreviated at both ends; dorsal striae 1-4 complete; 5^th^ present in apical half, sutural stria in apical two-thirds; elytral disk with coarser punctures confined to very narrow band near apex. Prosternal keel produced posterad into mesoventral emargination, carinal striae complete and connected at both ends, prosternal interspace with conspicuous microsculpture; prosternal lobe elongate, apically rounded, marginal stria well impressed at middle, variably obsolete laterally. Mesoventrite emarginate at front, marginal stria interrupted by broadly anteriorly arched mesometaventral stria; mesoventral disk with fine ground punctation only; postmesocoxal stria curved forward to mesepimeron; lateral metaventral stria extending to hind coxa, with a very short secondary lateral metaventral stria parallel to it just behind coxa; metaventral disk with inconspicuous ground punctation, with coarse punctures anteromediad metacoxae in a relatively discrete band in posterior one-fourth of disk; first abdominal ventrite with coarse punctures between metacoxae (mostly in anterior half of central portion of disk); all tibiae rather narrow and elongate, protibia with 5 weak marginal teeth, the meso- and metatibiae with 4-5 marginal spines, and well developed submarginal ridge. Propygidium with fine ground punctation and with uniformly placed coarser, crescent-shaped punctures, these set about half a puncture width apart; fine ground punctation of pygidium denser than propygidium, with slightly coarser punctures more or less uniformly spaced about 1–1.5 puncture widths apart, these denser toward basal margin; marginal pygidial stria present, complete, but very fine and close to margin. Male genitalia (Figs 20A–D): basal accessory sclerites present, but small and loosely associated; T8 with deep, broad basal emargination, apical emargination ‘V’-shaped, basal membrane attachment line complete, but very close to apex of basal emargination, ventrolateral apodemes strongly developed at base, gradually narrowing to apex; S8 with apical guides narrow, just slightly widening to apex, with a couple long apical spines, halves approximate along most of midline, fused in basal fourth to one-third; diverging slightly to apex; T9 with thin, rounded apices, ventrolateral apodemes forming blunt teeth near midpoint; T10 with halves separate, large, filling most of T9 space; S9 narrow and elongate, broadening in basal two-thirds, abruptly widened at extreme base, base bluntly rounded, with vague midline strengthening in apical half, apex with narrow emargination, apical flanges small and separate; aedeagus approximately parallel-sided in basal two-thirds, only very slightly widened to midpoint, evenly narrowed to apex, with moderately strong, ‘V’-shaped medioventral process with apex just basad midpoint; basal piece about one-fourth tegmen length; median lobe approximately one-third tegmen length.

#### Remarks.

The nominate species in the *Operclipygus sejunctus* group can be recognized by the metaventral punctures, which are largely and discretely limited to the posterior portion of the metaventral disk (Fig. 19B), in combination with the complete supraorbital stria.

### Operclipygus pecki sp. n.

urn:lsid:zoobank.org:act:52821ADB-5B4C-482F-A9CA-CC258A2846A9

http://species-id.net/wiki/Operclipygus_pecki

Figs 19C–D 20E Map 7

#### Type locality.

BOLIVIA: Santa Cruz: 5 km SSE Buena Vista, Flora y Fauna Hotel [17°29.925'S, 63°39.128'W].

#### Type material.

**Holotype male**: “**BOLIVIA: Dpto. Santa Cruz.** 5km SSE Buena Vista, Flora y Fauna Hotel 17°29.925'S, 63°39.128'W. 440m F.I.T. 15–24 Dec 2003. S. & J. Peck” / “Caterino/Tishechkin Exosternini Voucher EXO-01309” (CMNC)**. Paratypes (17):** 14: same data as type; same data as type except as noted: 2: 24–31.xii.2003 (CMNC, AKTC, FMNH, MSCC), 1: 7–12.v.2004, FIT, sandy forest, A. Cline & J. Wappes (AKTC)

#### Diagnostic description.

This species is extremely similar to *Operclipygus sejunctus*, differing only in a few characters as follows: length: 2.22–2.40 mm, width: 1.87–2.03 mm; body very faintly bicolored, with prothorax rufescent, posterior part of body rufo-piceous; frontal stria with secondary median interruption in some individuals; supraorbital stria interrupted at vertex; pronotal disk with lateral punctures shallower and fewer than *Operclipygus sejunctus*; anterior marginal pronotal stria complete behind head in most individuals; coarser elytral punctures densest at apex, but with few coarse punctures extending up the apical thirds of interstriae; mesoventral disk with few coarse punctures at sides; metaventral disk with strong ground punctation, with coarser punctures gradually becoming larger and denser toward metacoxae; secondary lateral metaventral stria longer, extending from mesometaventral suture about halfway to metacoxa; both ground punctation and coarse secondary punctation of propygidium and especially pygidium denser than in *Operclipygus sejunctus*. Male genitalia (Fig. 20E): as in *Operclipygus sejunctus* except S9 lacking apical emargination and with lateral flanges more strongly recurved at inner corners; aedeagus widest just beyond midpoint, narrowed toward base, then slightly widened at extreme base, gradually narrowed toward apex, but with apex appearing truncate, with distinct, membraneous apical pads that are widened beneath; medioventral process very weak, ‘V’-shaped, its apex at tegmen midpoint (not projecting in lateral view); median lobe about one-fourth tegmen length.

#### Remarks.

This species can be separated from others in the *Operclipygus sejunctus* group by its rufescent prothorax (Fig. 19B), diffuse metaventral punctation (Fig. 19C), and by the shape of the aedeagus (Fig. 20E).

#### Etymology.

We name this species in honor of Dr. Stewart Peck, collector of most of the type specimens of this species, as well as many others in this study.

### Operclipygus juninensis sp. n.

urn:lsid:zoobank.org:act:65BEDD1C-1A34-4AA3-8DC2-99D0B8B12BA9

http://species-id.net/wiki/Operclipygus_juninensis

Fig. 20F Map 7

#### Type locality.

PERU: Junín: ca. 1 km N Satipo, Sector San Isidro [11°14.51'S, 74°38.98'W].

#### Type material.

**Holotype male**: “**PERU: Depto. Junín**, ~1km N Satipo, Sector San Isidro. 11°14.51'S, 74°38.98'W 730m. Window trap at treefall, 11–12 April 2009. A.V.Petrov” / “Caterino/Tishechkin Exosternini Voucher EXO-01401” (FMNH). **Paratypes** (31): **BRAZIL: Mato Grosso:** 1: Querencia, Fazenda São Luiz, 12°38.819'S, 52°22.492'W, 560m, vii.2008, FIT, R.Andrade (CEMT); 2: Cotriguaçu, Fazenda São Nicolau, Mata Norte, 9°49.15'S, 58°15.6'W, 8–14.xii.2010, FIT, F.Z. Vaz-de-Mello (CEMT). **Pará:** 1:Jacareacanga, xii.1968, M. Alvarenga (UFPR). **PERU: Cusco:** 2: Consuelo, Manu rd. km 165, 9.x.1982, 10.x.1982, FIT, L. Watrous & G. Mazurek (FMNH); **Junín:** 1: ~1km N Satipo Sector San Isidro, 11°14.51'S, 74°38.98'W, 730m, 11–12.iv.2009, Window trap, at treefall, A.V. Petrov (AKTC); 2: 11km NE Puerto Ocopa, Los Olivos, 11°3.00'S, 74°15.52'W, 1200m, 30–31.iii.2009, FIT, A.K. Tishechkin (AKTC) DNA Extract MSC-2142; 1: 26–28.iii.2009, Window trap, A.V. Petrov (MUSM); 1: 8km NNE Puerto Ocopa, Rio Perene, Cananeden, 11°06'S, 73°50'W, 1100m, 6.ii.2008, A.V. Petrov (AKTC); 1: 7.5km NE Puerto Ocopa Cananeden, 11°4.9'S, 74°16.1'W, 1180m, 11.i.2007, A.V. Petrov (MUSM); 1: 25km SW Satipo, Kuviriaki, 1100m, 23.i.2007, A.V. Petrov (AKTC); **Loreto:** 2: Iquitos - Nauta rd., km 58, Rio Itaya, 4°15.738'S, 73°28.052'W, 120m, 5–9.v.2009, Window trap, next to entrance of *Eciton burchelli* statary bivouac in hollow treee, A.V. Petrov (AKTC); 4: 68km SW Iquitos to Nauta, Rio Itaya, 4°11'S, 73°26'W, 110m, 18–19.i.2008, A.V. Petrov (AKTC, MUSM), 2: 1–3.iii.2008, A.V. Petrov (AKTC), 1: 26–30.ii.2008, A.V. Petrov (AKTC); 2: km 63, rd. Iquitos - Nauta, Rio Itaya, 4°15.205'S, 73°26.074'W, 140m, 8–17.i.2010, A.V. Petrov (AKTC); **Madre de Dios:** 2: Manu National Park, Manu, 12.5624°S, 70.0930°W, 288m, 2–11.i.2007, pitfall, bamboo forest, J. Jacobs (CASC), 1: 20–29.vii.2006, pitfall, bamboo forest, J. Jacobs (CASC); 1: Manu National Park, Manu, 12.5421°S, 70.1435°W, 292m, 20–29.vii.2006, pitfall, bamboo forest, J. Jacobs (CASC); 1: Los Amigos Field Sta., 12.5421°S, 70.1435°W, 292m, 31.vii-9.viii.2006, pitfall, terra firma forest, J. Jacobs (CASC); 1: Manu National Park, Pantiacolla Lodge, 5.5km NW El Mirador Trail, Alto Madre de Dios River, 12°39'10"S, 71°15'28"W, 500m, 23–26.x.2000, FIT, R. Brooks (SEMC); 1: Manu National Park, Pakitza Bio. Stn., Reserved Zone, Castanal Trail, 11°56'41"S, 71°17'0"W, 317m, 15–16.x.2000, FIT, R. Brooks (SEMC).

#### Diagnostic description.

This species is extremely similar to *Operclipygus sejunctus*, differing only in a few characters as follows: length: 1.44–1.75 mm, width: 1.28–1.34 mm; body uniformly rufo-piceous; frons depressed, with coarse punctures intermingled with fine ground punctation; supraorbital stria interrupted; pronotal disk with slightly broader band of lateral punctures; elytra with sutural stria frequently longer, present on apical two-thirds to three-fourths; coarser elytral punctures densest at apex, but with few coarse punctures extending up the apical thirds of interstriae (as they do in *Operclipygus pecki*); sterna quite variable, but generally similar to *Operclipygus pecki*, the mesoventral disk with few coarse punctures at sides; metaventral disk with strong ground punctation, with coarser punctures gradually becoming larger and denser toward metacoxae; secondary lateral metaventral stria longer, extending from mesometaventral suture about halfway to metacoxa; pygidia also as in *Operclipygus pecki*, with very dense punctation. Male genitalia (Fig. 20F): as in *Operclipygus sejunctus* except S9 shorter, more markedly expanded to base, with basal margin varied from truncate to broadly rounded, lacking apical emargination, lateral flanges wide and strongly recurved at inner corners; tegmen with sides more or less evenly rounded from base to apex, apex subacute, medioventral process short, wide, slightly sinuate, with strong apical point located just basad tegmen midpoint, projecting beneath; median lobe about one-fourth tegmen length.

#### Remarks.

This species is very similar externally to *Operclipygus pecki* and *Operclipygus depressus*. It can be separated from the former by the lack of any hint of bicoloration (with the pronotum and elytra identical in coloration). From the latter it can only be confidently separated by the shape of the aedeagus (Fig. 20F). *Operclipygus juninensis* also generally has the outer lateral metaventral stria longer reaching the midpoint of the metaventrite, whereas it is generally very short in *Operclipygus pecki* and *Operclipygus depressus*.

#### Etymology.

We name this species for Junín department, Peru, where the type specimen was collected.

### Operclipygus depressus

(Hinton, 1935)

http://species-id.net/wiki/Operclipygus_depressus

Figs 19E–F 20G Map 7

Pseudister depressus Hinton, 1935: 584; *Operclipygus depressus*: Wenzel (1976: 257).

#### Type locality.

FRENCH GUIANA:Saint-Laurent du Maroni [5°30'N, 54°2'W].

#### Type material.

**Lectotype**, here designated (BMNH): “Guyane Francse., St-Laurent du Maroni, Collection Le Moult” / “G.Lewis Coll. B.M.1926-369” / “LECTOTYPE *Pseudister depressus* Hinton, 1935, M.S.Caterino & A.K.Tishechkin des. 2010”, somewhat damaged (BMNH). This species was described from an unspecified number of specimens, and the lectotype designation fixes primary type status on the only known original specimen.

#### Other material. 

1: **BOLIVIA: Santa Cruz:** Amboro National Park, Los Volcanes, 18°06'S, 63°36'W, 1000m, 20.xi–12.xii.2004, FIT, Mendel, H. & Barclay, M.V.L. (BMNH). **BRAZIL: Pará:** 1:Tucuruí, 3°45'S, 49°40'W, iv.1986, FIT (CHND), 3: v.1986, FIT (CHND), 2: 19.vi–7.vii.1986, FIT (CHND), 2: 20.v–15.vi.1987, FIT (CHND); Carajás (Serra Norte), 6°04'S, 50°12'W, xi.1984, FIT (CHND). **COLOMBIA: Vaupés:** 1: Parque Nac. Mosiro-Itajura (Caparú), Centro Ambiental, 1°04'S, 69°31'W, 60m, 20–30.i.2003, FIT, D. Arias & M. Sharkey (IAVH). **ECUADOR: Orellana:** 1: Tiputini Biodiversity Station, 0.6376°S, 76.1499°W, 4–9.vi.2011, FIT, M.S. Caterino & A.K. Tishechkin (SBMNH) DNA Extract MSC-2177; 1: Tiputini Biodiversity Station, 0°37'44"S, 76°08'39"W, 220–250m, 26.x.1998, fogging, T.L. Erwin (USNM); 1: Res. Ethnica Waorani, 1km S Onkone Gare Camp, Trans. Ent., 0°39'10"S, 76°26'W, 220m, 8.ii.1995, fogging, T.L. Erwin (USFQ); 2: Parque Nac. Yasuní, Via Maxus at Puente Piraña, 0°39.5'S, 76°26'W, 14–20.vii.2008, FIT, A.K. Tishechkin (AKTC); **Zamora Chinchipe:** 1: Yanzatza at Rio Yanzatza, 7.xi.1979, inflorescence of *Heliconia*, Jos. J. Anderson (USNM). **FRENCH GUIANA:** 13: Mont tabulaire Itoupé, 3°1.38'N, 53°5.73'W, 570m, 17.iii.2010, FIT, SEAG (CHND), 16: 570m, 24.iii.2010, FIT, SEAG (CHND), 1: 400m, 23.iii.2010, FIT, SEAG (CHND), 14: 570m, 31.iii.2010, FIT, SEAG (CHND); 1: Montagne des Chevaux, 4°43'N, 52°24'W, 16.xii.2008, FIT, SEAG (MNHN); 1: 65 (Cayenne-Kourou) lieu-dit Tibourou, 20.ix.2008, FIT, J. Touroult (CHND); 1: Rés. Natur. des Nouragues, Camp Inselberg, 4°05'N, 52°41'W, 25.i.2011, Window trap, SEAG (MNHN), 2: 31.iii.2011, Window trap, SEAG (MNHN). **GUYANA: Region 8**: 2: Iwokrama Forest, Pakatau hills, 4°44'54"N, 59°1'36"W, 70m, 25–29.v.2001, FIT, R. Brooks & Z. Falin (SEMC); 3: Kurupukari, 4°40'N, 58°40'W, ix-xi.1992, malaise/FIT (BMNH); 1: 26.x.1992, FIT (BMNH). **PERU: Loreto:** 1: km 63, rd. Iquitos - Nauta, Rio Itaya, 4°15.205'S, 73°26.074'W, 140m, 9–13.i.2011, A.V. Petrov (AKTC); 5: 1.5km N Teniente Lopez, 2°35.66'S, 76°06.92'W, 210–240m, 18.vii.1993, FIT, R. Leschen (SEMC); 3: 20.vii.1993, FIT, R. Leschen (SEMC). **SURINAME: Sipaliwini:** 1: CI-RAP Surv. Camp 2: Sipaliwini River, 2°10.521'N, 56°47.244'W, 210m, 27.viii-1.ix.2010, FIT, T. Larsen & A.E.Z. Short (SEMC); **Para**, 1:near Overbridge, 05°31'10"N, 55°04'10"W, 10–14.ii.2010, FIT, W.B. Warner (WBWC). **VENEZUELA: Amazonas**: 1: Cerro de la Neblina Basecamp, 0°50'N, 66°10'W, 140m, 26–31.i.1985, FIT, P.J. & P.M. Spangler, R.A. Faitoute, W.E. Steiner colrs. (USNM); **Bolívar**: 1:6km S San Iisidro, Km 88, 25.vi–11.vii.1987, FIT, lowland rain forest, S. & J. Peck (FMNH).

#### Diagnostic description.

This species is extremely similar to *Operclipygus sejunctus*, differing only in a few characters as follows: length: 1.90–2.40 mm, width: 1.59–2.09 mm; body uniformly rufo-piceous; frons with frontal stria nearly complete, only very narrowly interrupted above antennal bases; supraorbital stria interrupted across vertex; pronotum with lateral submarginal stria complete, distance from margin slightly increasing toward front; elytra somewhat variable, generally as in *Operclipygus juninensis*, sutural stria frequently longer than in *Operclipygus sejunctus*, present on apical two-thirds to three-fourths; coarser elytral punctures densest at apex, but with few coarse punctures extending up the apical third of interstriae; sterna as in *Operclipygus pecki* and *Operclipygus juninensis*, mesoventral disk rarely with few coarse punctures at sides; metaventral disk with strong ground punctation, coarser punctures gradually becoming larger and denser toward metacoxae; secondary lateral metaventral stria generally very short; propygidium and pygidium as in *Operclipygus sejunctus*. Male genitalia (Fig. 20G): T8 relatively short, basal membrane attachment line just intersecting basal emargination in some specimens; S8 with halves approximate but separate throughout length; S9 with apical emargination very small or lacking, lateral flanges narrow; tegmen with sides more or less straight, widest about two-thirds from base, evenly narrowed to base, somewhat abruptly narrowing in apical third, with apices narrowly rounded, medioventral process narrow, acute, about one-third from base, weakly projecting beneath; median lobe about one-fourth tegmen length.

#### Remarks.

*Operclipygus depressus* has the pronotal marginal bead rather convex (Fig. 19E), and widening toward the front, as is also distinct in the following two species. The male metaventrite is not depressed setose (as is the case in *Operclipygus setiventris*), and the 4^th^ dorsal elytral stria is complete (abbreviated in *Operclipygus itoupe*). Females of *Operclipygus depressus* and *Operclipygus setiventris* may not be consistently distinguishable.

### Operclipygus setiventris sp. n.

urn:lsid:zoobank.org:act:48B8BE56-A311-4FC7-9E48-2FF9C49198C5

http://species-id.net/wiki/Operclipygus_setiventris

Figs 19G 21 Map 7

#### Type locality.

FRENCH GUIANA: Itoupé Table Mountain [3°1.38'N, 53°5.73'W].

#### Type material.

**Holotype male**: “**GUYANE FR.,** Mont Tabulaire, Itoupé. 3°1.38'N, 53°5.73W. 570m. Piège d’interception 31 Mar 2010. SEAG leg.” / “Caterino/ Tishechkin Exosternini Voucher EXO-00272” (MNHN). **Paratypes** (2): same data as type, except as noted: 1: 17.iii.2010 (CHND); 1: **FRENCH GUIANA:** Montagne des Chevaux, 4°43'N, 52°24'W, 14.iv.2010, FIT, SEAG (FMNH).

#### Other material

(4 females): same data as type, except as noted: 1: 17.iii.2010 (CHND), 1: 24.iii.2010, FIT, SEAG (FMNH); **FRENCH GUIANA:** 1: Montagne des Chevaux, 4°43'N, 52°24'W, 21.ix.2008, FIT, SEAG (MSCC); 1: Rés. Natur. des Nouragues, Camp Inselberg, 4°05'N, 52°41'W, 25.i.2011, Window trap, SEAG (MNHN).

#### Diagnostic description.

**Figure 21.**
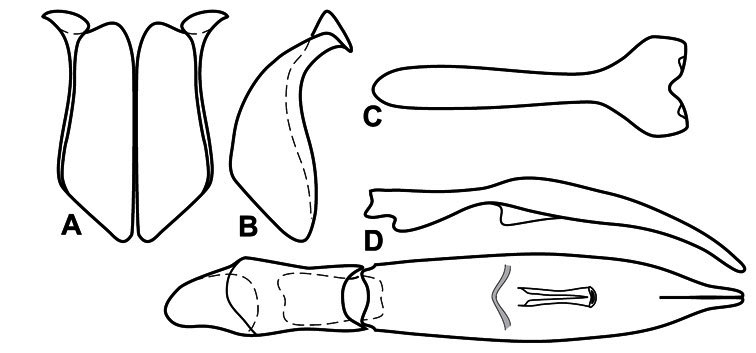
Male genitalia of *Operclipygus setiventris*. **A** T8 **B** S8 **C** S9 **D** Aedeagus, lateral and dorsal views.

This species is extremely similar to *Operclipygus depressus*, differing most significantly in showing quite pronounced sexual dimorphism; length: 2.37–2.71 mm, width: 1.97–2.31 mm; body rufescent, broadly rounded, more subdepressed than typical in this group, with relatively long legs and narrow tibiae; pronotal disk with relatively small but numerous (~22) lateral discal punctures; lateral submarginal pronotal stria increasingly distant from margin anterad (as in *Operclipygus depressus*); elytral striae as in *Operclipygus sejunctus* but with stria 4 finely impressed and fragmented anteriorly in some individuals; coarser elytral punctures densest at apex, but with few coarse punctures extending up the apical third of interstriae; sterna of female as in *Operclipygus depressus*, meso- and metaventral disks of male broadly depressed and setose, most of the punctures being strong ground punctures bearing setae, with only a few coarser punctures near metacoxae; outer lateral metaventral stria long, extending from mesometaventral suture along at least half of inner stria; propygidium and pygidium as in *Operclipygus sejunctus*.Male genitalia (Fig. 21) highly autapomorphic: accessory sclerites present; T8 elongate, parallel-sided in apical two-thirds, widened at base, with basal membrane attachment line distad basal emargination by about half its depth, apical emargination narrow, ventrolateral apodemes well-developed, meeting at midline about one-third from base, diverging to apex; S8 with halves separate, sides parallel and apical guides narrow over basal 3/4, then abruptly divergent and downturned at apex; S9 narrowest near apex, weakly widened then narrowed to base, apex more or less truncate, with shallow apical emargination, apical flanges small, midline sclerotized; tegmen short, only slightly longer than basal piece, widest just basad midpoint, weakly narrowed to base, more strongly narrowed to narrow apex, medioventral process wide, short, thin, with a blunt apex about one-third from base, strongly produced beneath; median lobe about one-half tegmen length.

#### Remarks.

The distinctly depressed and setose male metaventrite (Fig. 19G), as well as the unusual male genitalia (Fig. 21), are the best characters for recognizing this species. Females are difficult to separate from either *Operclipygus depressus* or from *Operclipygus itoupe*. The apparent females of *Operclipygus setiventris* are relatively broad bodied and long-legged, like the males, but there is variation in these characters even among the few available specimens. The well-developed lateral metaventral stria will generally separate them from *Operclipygus depressus*. The complete 4^th^ dorsal stria will separate them from most *Operclipygus itoupe*. We restrict the type series to known males.

#### Etymology.

The name of this species refers to the setose meso- and metaventrites of the males.

### Operclipygus itoupe sp. n.

urn:lsid:zoobank.org:act:30F18631-54EC-4BE7-AD21-64FD512889E0

http://species-id.net/wiki/Operclipygus_itoupe

Figs 19H 20H Map 7

#### Type locality.

FRENCH GUIANA:Itoupé Table Mountain [3°1.38'N, 53°5.73'W].

#### Type material.

**Holotype male**: “**GUYANE FR.,** Mont Tabulaire, Itoupé. 3°1.38'N, 53°5.73'W. 570m. Piège d’interception 17 Mar 2010. SEAG leg.” / “Caterino/Tishechkin Exosternini Voucher EXO-00273” (MNHN). **Paratypes** (12): 8: same data as type (FMNH, MSCC, AKTC, CHND); 1: Montagne des Chevaux, 4°43'N, 52°24'W, 23.ii.2009, FIT, SEAG (MNHN), 2: 16.xii.2008 (MNHN); 1: Rés. Natur. des Nouragues, Camp Inselberg, 4°05'N, 52°41'W, 25.i.2011, Window trap, SEAG (MNHN).

#### Diagnostic description.

This species is extremely similar to *Operclipygus sejunctus*, differing especially in a few characters as follows: length: 2.15–2.56 mm, width: 1.90–2.31 mm; body rufopiceous, diffusely rufescent toward elytral sides, more broadly rounded and more strongly convex than *Operclipygus sejunctus*, though distinctly flattened to subplicate across base of pronotum; sutural area of elytra depressed; elytral disk with few or no coarse apical punctures; elytral stria 4 fragmented to absent in basal half; venter lacking microsculpture; mesoventral marginal stria complete, mesoventral disk with a few small lateral punctures; metaventral punctures restricted to discrete grouping in posterior one-fourth of disk; secondary lateral metaventral stria present along basal half of inner metaventral stria; propygidium with coarse punctures becoming very sparse toward sides; pygidium with coarse punctures very small, about 2× the size of ground punctures, uniformly scattered about 4–5 puncture widths apart. Male genitalia (Fig. 20H): T8 relatively short, with basal membrane attachment line just intersecting basal emargination; S8 with halves separate throughout their lengths; S9 relatively broad, narrowest just distad middle, weakly widened to subtruncate base, apex broadly arcuate, without discrete median apical emargination, apical flanges more or less continuous across apex, with secondary sclerotizations along edges rather than along midline; tegmen widest about three-fourths from base, narrowing gradually to base, abruptly to apex, with apex narrowly rounded, medioventral process weakly sclerotized, narrowly ‘V’-shaped, not projecting ventrally; median lobe about one-third tegmen length.

#### Remarks.

The abbreviated 4^th^ dorsal elytral stria, lack of apical elytral punctures, and discrete posterolateral patch of metaventral punctures (Fig. 19H) will generally separate this species from all others of the *Operclipygus sejunctus* group.

#### Etymology.

This species is named for its type locality, the table mountain Itoupé, as a noun in apposition.

### Operclipygus punctiventer sp. n.

urn:lsid:zoobank.org:act:5C73ECB6-5DD4-429B-8090-39C8B8EE3E21

http://species-id.net/wiki/Operclipygus_punctiventer

Figs 6C 20I Map 7

#### Type locality.

COSTA RICA: Heredia: LaSelva Biological Station [10°26'N, 84°01'W].

#### Type material.

**Holotype male**: “**COSTA RICA**: Heredia, Est. Biol. LaSelva. 10.26’[sic]N 84.01'W. F.I.T.22June 1998 C.Carlton & A. Tishechkin” / “LSAM 0046242” (FMNH). **Paratypes** (21): 2: same data as type (FMNH, LSAM, MSCC, AKTC);same as type, except as noted: 1: 23.vi.1998 (LSAM), 2: 21.vi.1998 (LSAM), 1: 80m, 8.–11.vi.2001, FIT, S. Chatzimanolis (SEMC), 1: 10.vi.2012, OTS Beetle Course, DNA Extract MSC-2305 (SBMNH); **Limón:** 1: Area Cons. Tortuguero, Sector Cerro Cocori, Fca. de E. Rojas, 150m, v.1993, E. Rojas, (INBIO); **Puntarenas:** 1: Rancho Quemado, Peninsula de Osa, 200m, iv.1992, D. Brenes (INBIO), 1: xii.1992, F. Quesada, (INBIO); 1: Est. Biol. Las Cruces, San Vito, 1200m, viii.1982, B.D. Gill (CHSM); 1: Est. Agujas, Sendero Zamia, Rio Agujas, 300m, 2–15.i.1996, FIT, A. Azofeifa, (INBIO). **BELIZE: Cayo:** 2: Las Cuevas Research Station, 5.viii.1994, FIT (BMNH), 1: 16.ix.1994, FIT (BMNH). **ECUADOR: Pichincha:** 1: Rio Palenque Sta., 47km S Santo Domingo, 29.v.1975, fruit litter (FMNH). **HONDURAS: Cortés:** 2: Yojoa Lake, Deer Island, 14°55'N, 87°58'W, 670m, 22–26.vi.1994, FIT, J. Ashe, R. Brooks (SEMC); **PANAMA: Colón:** 1: San Lorenzo Forest, 9°17'N, 79°58'W, 4–6.x.2003, FIT, A.K. Tishechkin (GBFM), 1: 8–9.x.2003 (LSAM), 1: 21–22.x.2003 (GBFM), 1: 24–25.x.2003 (LSAM).

#### Diagnostic description.

This species is extremely similar to *Operclipygus sejunctus*, differing only in a few characters as follows: length: 1.93–2.40 mm, width: 1.75–2.06 mm; body rufo-piceous; generally smooth, ground punctation inconspicuous; central portion of anterior pronotal margin more subacute than simply outwardly arcuate; elytra with 4^th^ stria generally interrupted, occasionally absent from much of basal half; 5^th^ stria present in about apical fourth; coarse apical elytral punctures generally restricted to a single series along the margin; prosternal keel truncate to weakly emarginate posteriorly, with mesoventrite truncate or very weakly projecting; mesoventral marginal stria complete; mesometaventral stria subangulate rather than rounded anteriorly; ground punctation of meso- and metaventral disks relatively fine and inconspicuous, the coarser punctures of metaventral disk present in a discrete area in posterior two-thirds; secondary lateral metaventral stria long, paralleling metaventral stria for about two-thirds its length; first abdominal ventrite with few or no coarse punctures; propygidium and pygidium as in *Operclipygus sejunctus*.Male genitalia (Fig. 20I): S8 with halves approximate but separate throughout their length; S9 strongly narrowed at midpoint, weakly widened to base and apex, with entire apex broadly arcuate, but apical emargination very small, lateral flanges narrow, barely recurved at inner corners; tegmen approximately parallel-sided in basal three-fourths, apical division very short, visible in apical one-eighth or less, medioventral process narrow, acute, weakly projecting beneath; median lobe slightly about half tegmen length.

#### Remarks.

In addition to being the only species of the *Operclipygus sejunctus* group occurring principally outside of South America, this species can be separated from the others by its more or less truncate anterior mesoventral margin (and correspondingly weakly projecting prosternal keel; Fig. 19H). Also, the simple series of apical elytral punctures is distinct from others in this group (though not unique in the genus.) The range of this species extends from northern Central America through western Ecuador. Although the latter record appears somewhat surprising, a number of largely Central American Exosternini have shown up in western Ecuador.

#### Etymology.

The name of this species refers to the punctures on the metaventrite, unusual to members of the *Operclipygus sejunctus* group.

### *Operclipygus mortavis* group

**Figure 22.**
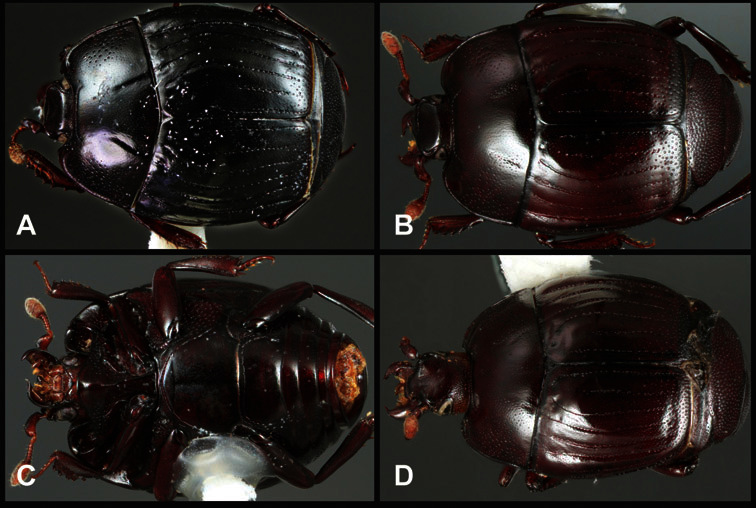
*Operclipygus mortavis* group. **A** Dorsal habitus of *Operclipygus mortavis* **B** Dorsal habitus of *Operclipygus ecitonis* **C** Ventral habitus of *Operclipygus ecitonis* **D** Dorsal habitus of *Operclipygus paraguensis*.

**Figure 23.**
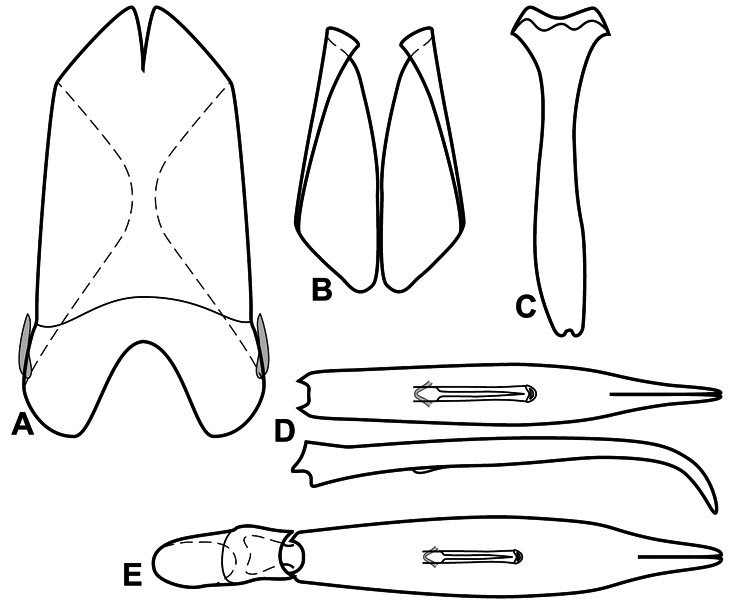
Male genitalia of *Operclipygus mortavis* group. **A** T8 of *Operclipygus ecitonis* **B** S8 of *Operclipygus ecitonis* **C** S9 of *Operclipygus ecitonis* **D** Aedeagus, dorsal and lateral views, of *Operclipygus ecitonis* **E** Aedeagus, dorsal view, of *Operclipygus paraguensis*.

This small group of three species is quite distinctive. They are larger in size than average, and more generally rounded and convex than many (Fig. 22). They have strong basal plicae on the pronotum, a strongly depressed frons, with the frontal stria interrupted on each side, and nearly complete elytral striation. The male genitalia (known for only two of the three species; Fig. 23) are quite similar to those of members of the *Operclipygus mirabilis* group, with an apically hooked aedeagus, narrow basal T8 emargination, and weakly emarginate S9. The pronotal plica is also shared by the two groups. Although they are not resolved as sister groups in any of our analyses to date, this possible relationship should be examined further.

### Key to the species of the *Operclipygus mortavis* group

**Table:** 

1	5^th^ dorsal stria complete to base (Fig. 22A); pronotal plical ridge impunctate; Central America	*Operclipygus mortavis* sp. n.
–	5^th^ dorsal stria obsolete in basal third; pronotal plical ridge with punctures concentrated along its length (Fig. 22B, D); South America	2
2	Inner subhumeral elytral stria well impressed	*Operclipygus paraguensis* sp. n.
–	Inner subhumeral elytral stria present only as an incomplete series of punctures	*Operclipygus ecitonis* sp. n.

### Operclipygus mortavis sp. n.

urn:lsid:zoobank.org:act:44BFCD78-6A52-4621-9D37-1714B1B7E1E3

http://species-id.net/wiki/Operclipygus_mortavis

Fig. 22A Map 8

#### Type locality.

COSTA RICA: Guanacaste: Miravalles Volcano [10.72°N, 85.17°W].

#### Type material.

**Holotype female**: “C.R.: Guan.; Vol. Miravalles; S. slope, 2800’ VII-2-1993; M.S. Caterino; pitfall w/dead bird and frog” / “Caterino/Tishechkin Exosternini Voucher EXO-00266” (FMNH).

#### Diagnostic description.

Length: 3.00 mm, width: 2.81 mm; body piceous, sides rounded, moderately convex above; head with frons depressed along midline, frontal stria present in middle, interrupted at sides; supraorbital stria absent; labrum about twice as wide as long, its apical margin strongly emarginate; left mandible untoothed, right with small tooth at base of incisor; pronotum with strong plicae opposite 3^rd^ elytral stria in basal half; marginal pronotal stria continuous, complete along anterior edge; lateral submarginal pronotal stria complete, just curved inward at front; anterior submarginal stria present, recurved posterad at sides; median pronotal gland openings laterad ends of submarginal stria; pronotal disk with numerous elongate, coarse punctures toward sides; elytra with three complete epipleural striae, outer subhumeral stria complete, although barely broken at sinuation at middle, inner more or less complete, barely abbreviated at base and apex, striae 1-5 complete, 2-5 noteably bent inward near base, sutural abbreviated one-fourth from base; elytral disk with numerous coarse punctures near apex; prosternal keel weakly emarginate at base, with carinal stria enclosing a triangular space about two-thirds length of keel (though not meeting basally); mesoventrite projecting weakly at middle, with complete marginal stria; mesometaventral stria arched forward in middle, continued by sinuate lateral metaventral stria to middle of metacoxa; postmesocoxal stria curving forward to mesepisternum; 1^st^ abdominal ventrite with two lateral striae, the outer abbreviated; propygidium with uniform coarse punctures separated about their diameters; pygidium with coarse punctures finer, diminishing to apex; marginal pygidial stria entire, finely but deeply impressed. Male not known.

#### Remarks.

In addition to the characters distinguishing this small species group, this species can be recognized by its simple (not punctate) pronotal plicae (Fig. 22A), and its complete 5^th^ dorsal elytral stria.

#### Etymology.

The name of this species means ‘dead bird’, to which the sole specimen was attracted, and is a noun in apposition.

### Operclipygus ecitonis sp. n.

urn:lsid:zoobank.org:act:F41E3C0B-AB0C-44B9-A76B-A3F932647E68

http://species-id.net/wiki/Operclipygus_ecitonis

**Map 8.**
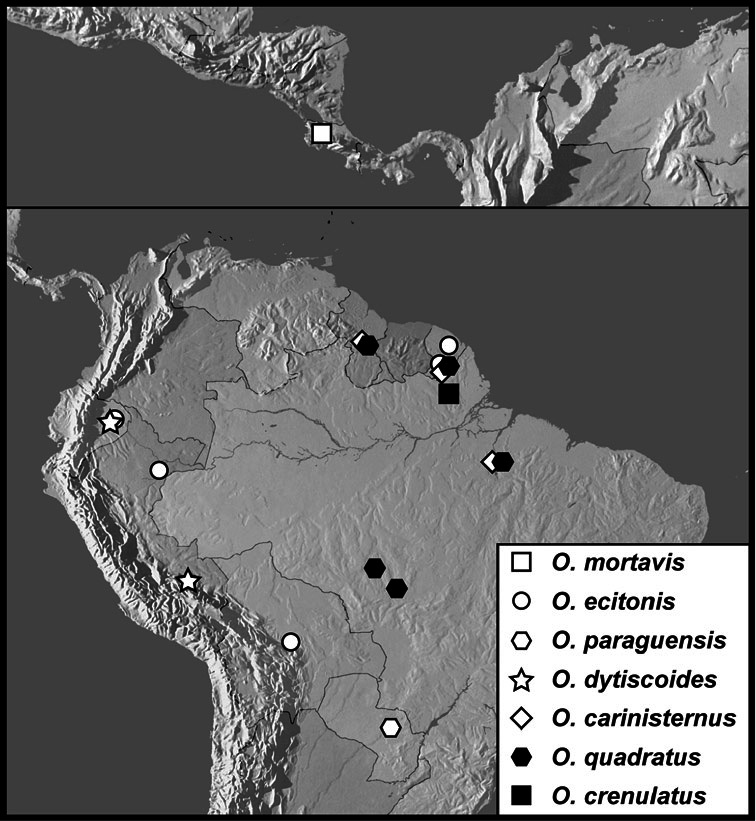
Records of the *Operclipygus mortavis* and *Operclipygus dytiscoides* groups.

Figs 22B–C 23A–D Map 8

#### Type locality.

ECUADOR: Orellana: Yasuní Research Station [0°40.5'S, 76°24'W].

#### Type material.

**Holotype male**: “**ECUADOR:** Napo, Yasuní Res. Stn. on mid.Rio Tiputini. 0°40.5'S, 76°24'W. 29 Jul 1999” / “*Eciton burchelli* Colony EC#27, refuse deposit statary phase. AKT#109, A. Tishechkin leg.” / “LSAM 0045442” (FMNH); **Paratypes** (3): **ECUADOR: Orellana:** 1:Yasuní Res. Stn., on mid.Rio Tiputini, 0°40.5'S, 76°24'W, 17–18.vii.1999, *Eciton burchelli* colony, at statary bivouac during emigration, A.K. Tishechkin (LSAM). **BOLIVIA: Cochabamba:** 1: Est. Biol. Valle Sajta, Univ. San Simon, 67.5km E Villa Tunari, 17°06'19"S, 64°46'57"W, 300m, 9–13.ii.1999, FIT, lowland rain forest, F. Genier (CMNC). **PERU: Loreto:** 1: Iquitos - Nauta rd., km 58, Rio Itaya, 4°15.738'S, 73°28.052'W, 120m, 5–9.v.2009, Window trap, next to entrance to *Eciton burchelli* statary bivouac in hollow treee, A.V. Petrov (AKTC).

#### Other material.

**FRENCH GUIANA:** 1: Montagne des Chevaux, 4°43'N, 52°24'W, 16.xi.2009, FIT, SEAG (CHND); 1: Mont Tabulaire, Itoupé, 3°1.32'N, 53°5.05'W, 800m, 17.iii.2010, FIT, SEAG (MSCC); 1: Rés. Natur. des Nouragues, Camp Inselberg, 4°05'N, 52°41'W, 30.ix.2010, Window trap, SEAG (MNHN).

#### Diagnostic description.

This species is very similar to the preceding, differing as follows: length: 2.40–2.65 mm, width: 2.25–2.50 mm; body rufopiceous; head with fragments of supraorbital stria present; pronotal plicae with concentration of coarse punctures along its length; lateral submarginal pronotal striae deeply impressed; coarse punctures of pronotal disk occurring in lateral one-third of each side; elytra with two or three complete epipleural striae, outer subhumeral stria interrupted or not, stria 5 fragmented to obsolete in basal half, usually with a distinct basal point; striae 2–5 obliterated apically in coarse apical punctures of elytral disk; mesometaventral stria arched far forward, nearly contacting marginal mesoventral (which remains complete); 1^st^ abdominal ventrite with complete anterior stria uniting inner lateral striae; punctures of propygidium and pygidium denser; marginal pygidial stria less deeply impressed, particularly near bases. Male genitalia (Figs 23A–D): accessory sclerites present; T8 elongate, parallel-sided, basal apodemes only slightly divergent, basal emargination narrow, deep, with basal membrane attachment line distad by about one-half basal emargination depth, apical emargination narrow, shallow; ventrolateral apodemes evenly rounded, nearly meeting beneath; S8 with apical guides narrow, evenly expanded to apex, halves approximate in basal half, diverging to apex; T9 with apices curving, subacute, nearly meeting; T10 with halves separate; S9 slightly curved to base, with base narrowly emarginate, apex shallowly arcuate, without distinct median emargination, but with apical flanges separated at middle; tegmen elongate, slender, slightly narrowing to base, narrowing in apical third to subacute apices, apex strongly curved ventrally; medioventral process small but well sclerotized, ‘U’-shaped, projecting weakly beneath; basal piece about one-third tegmen length; median lobe about one-third tegmen length, slender.

#### Remarks.

The complete stria across the anterior margin of abdominal ventrite 1 separates this species from the others in this group (and from many other *Operclipygus* as well; Fig. 22C.) We limit the type series to those specimens from western Amazonia. Specimens from the disjunct Guianan localities show very minor differences in sculpturing that may reflect significant differentiation.

#### Etymology.

The name of this species refers to its apparent association with army ants of the genus *Eciton*.

### Operclipygus paraguensis sp. n.

urn:lsid:zoobank.org:act:119B092A-EB96-44B1-96D6-3E55CE87EF18

http://species-id.net/wiki/Operclipygus_paraguensis

Figs 22D 23E Map 8

#### Type locality.

PARAGUAY: San Pedro: Cororô [23°28'S, 56°39'W]

#### Type material.

**Holotype male**: “**PARAGUAY: San Pedro**, Cororô. 23°28'N[sic, should be S], 56°39'W. 180m, 27–30 Oct 1999. J.Jensen” / “Caterino/Tishechkin Exosternini Voucher EXO-00267” (FMNH).

#### Diagnostic description.

As above, this species is very similar to *Operclipygus mortavis*, but with a number of minor differences: length: 2.56 mm, width: 2.37 mm; ground punctation of most of body coarser, more conspicuous, especially on sides of pronotum, with coarse punctures nearly continuously scattered from basal plicae to sides; pronotal disk more strongly, somewhat irregularly convex; elytra with outer subhumeral stria weakly impressed, interrupted at middle and strongly abbreviated in anterior half, inner subhumeral stria represented by a fine series of punctures, not impressed as a distinct stria; 1^st^ abdominal ventrite with anterior stria interrupted at middle for about width of one metacoxa. Male genitalia (Fig. 23E): as in preceding species, but with tegmen broader, sides more arcuate than parallel, narrowing more strongly to base.

#### Remarks.

The very weak inner subhumeral stria provides the most straightforward character for separating this species from the preceding.

#### Etymology.

This species is named for the country from which it is exclusively known.

### *Operclipygus dytiscoides* group

**Figure 24.**
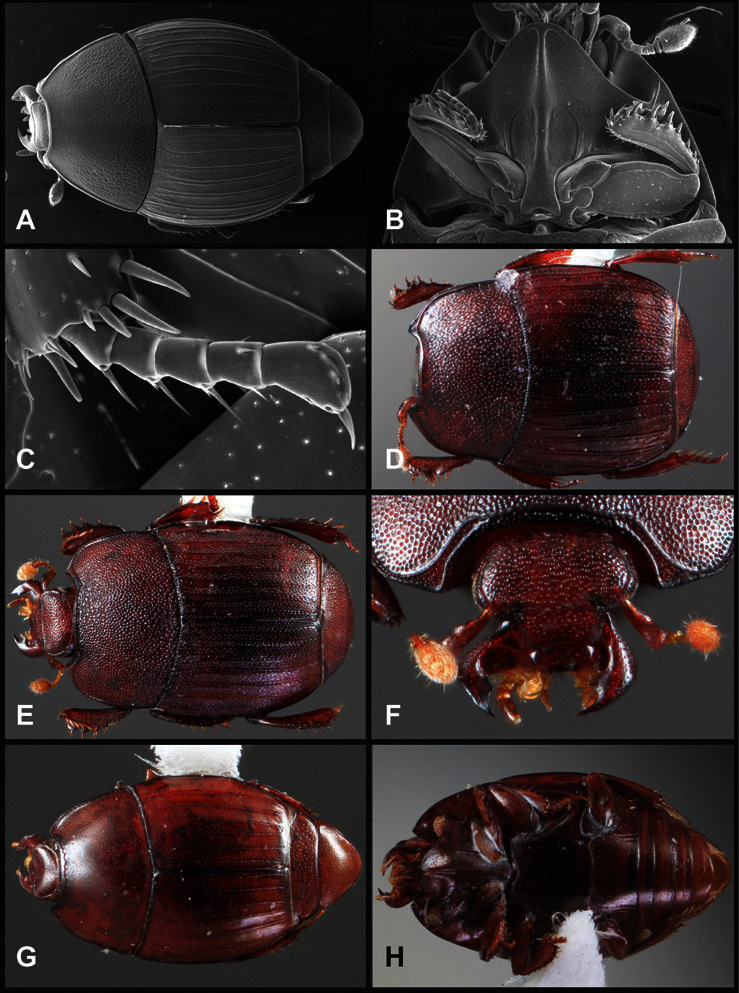
*Operclipygus dytiscoides* group. **A** Dorsal habitus of *Operclipygus dytiscoides* **B** Prosternum of *Operclipygus dytiscoides* **C** Tarsus of *Operclipygus dytiscoides* **D** Dorsal habitus of *Operclipygus carinisternus* **E** Dorsal habitus of *Operclipygus quadratus* **F **Mandibles of *Operclipygus quadratus* **G** Dorsal habitus of *Operclipygus crenulatus* **H** Ventral habitus of *Operclipygus crenulatus*.

The *Operclipygus dytiscoides* group is a highly distinctive and enigmatic group of four species. Their relationships to *Operclipygus* are supported by a reasonable number of external characters. However, the only species of the group for which a male is known, *Operclipygus quadratus*, is itself only assigned here with some uncertainty, and the position and monophyly of the species will need to be reevaluated when males of the other species are discovered. The name of the group refers to a flattened, elliptical body form that narrows rather strongly toward the front (especially in the nominate species – Fig. 24A). The strongest and most distinctive character uniting these species is the prosternal lobe with a strong median ridge (Fig. 24H), unique among Neotropical Exosternini. In addition, the form of the pronotal striae is distinctive, with the lateral submarginal stria running very close to the marginal, with the pronotal disk depressed along its inner edge, continuous with the anterior marginal stria, which departs conspicuously from the anterior margin behind the head, producing a broad, flat marginal bead (Figs 24A, F, G). All of the species have fairly distinctive elytral striae, with each stria consisting of a pair of finely elevated carinae, though such are seen in other genera as well, and the elytral striae are all more or less complete, with the outer subhumeral stria forming a distinct marginal ridge. Three of the four species below are coarsely punctate on all body surfaces, but one is relatively impunctate.

### Key to the species of the *Operclipygus dytiscoides* group

**Table:** 

1	Apical tarsomere swollen apically, subtriangular in lateral view (Fig. 24A)	2
–	Ultimate tarsomere simple, cylindrical to very slightly expanded to apex	3
2	Elytron with apical marginal stria uniting apices of dorsal striae (Fig. 24D); inner and outer subhumeral striae joined about one-third from elytral apex	*Operclipygus carinisternus* sp. n.
–	Elytron lacking apical marginal stria; inner and outer subhumeral striae free throughout their lengths (Fig. 24A)	*Operclipygus dytiscoides* sp. n.
3	Junction of anterior and lateral submarginal pronotal striae smooth (Fig. 24G); recurrent metaventral stria present (Fig. 24H)	*Operclipygus crenulatus* sp. n.
–	Anterior and lateral submarginal pronotal striae meeting at angulate postocular junction (Figs 24E, F); recurrent stria of metaventrite absent	*Operclipygus quadratus* sp. n.

### Operclipygus dytiscoides sp. n.

urn:lsid:zoobank.org:act:DEE36779-F5AC-46CF-90AA-B6F7ECFD21B5

http://species-id.net/wiki/Operclipygus_dytiscoides

Figs 24A–C Map 8

#### Type locality.

ECUADOR: Orellana: Tiputini Biodiversity Station [0°38'S, 76°9'W].

#### Type material.

**Holotype female**: “ECUADOR Napo Region 5–25.ix.00” / “Tiputini Research Station 220m 0°38'0"S, 76°9'0"W” / “BM2000:194 D.J.Inward K.A.Jackson” / “Caterino/Tishechkin Exosternini Voucher EXO-00017”, specimen gold coated for SEM (BMNH).

#### Other material.

**PERU: Cusco:** 1 female: Villa Carmen Field Station, 12.89250°S, 71.41917°W, 24–26.v.2011, FIT (SEMC).

#### Diagnostic description.

Length: 1.90 mm, width: 1.59 mm; body rufo-piceous, elongate oval, prolonged anteriorly, depressed, coarsely punctate over most of pronotum, less strongly so on elytra; head deeply recessed into prothorax at rest, frons broad, punctate, frontal striae straight, divergent between eyes, with central portion transverse, complete; epistoma weakly depressed, apical margin shallowly emarginate; labrum two-thirds as long as wide, apical margin outwardly rounded; mandibles strong, left with small basal tooth, right with stronger one; prothorax with sides convergent, straight in basal two-thirds, abruptly narrowed, sinuate in apical third; lateral marginal pronotal stria present along side, ending freely in a small inward hook in anterior corner; lateral submarginal stria close to marginal, strongly impressed along inner edge, bent inward and continuous with anterior marginal stria across front, marginal bead broad and slightly elevated; pronotal disk strongly punctate throughout, punctures becoming smaller and sparser anterad, lacking plicae or prescutellar impression; median pronotal gland openings difficult to discern among punctures but present, simple, about head-width apart, nearly one-fourth pronotal length behind anterior margin; elytra with two complete epipleural striae, with all dorsal striae elevated, sides of each stria subcarinate with relatively broad, flat inner depression; all striae complete except inner subhumeral, barely abbreviated at apex; elytral disk with numerous small punctures in elytral intervals; prosternal keel trunctate posteriorly, narrowing strongly toward presternal suture, carinal striae fragmented to absent; lateral prosternal striae curving inward toward front; prosternal lobe with strong median ridge continuous with prosternal keel, apical marginal stria interrupted at middle where ridge reaches edge; mesoventrite shallowly emarginate at middle, marginal stria interrupted at middle by strongly arched meso-metaventral stria; postmesocoxal stria arched to mesepimeron, lateral metaventral stria extending nearly to metacoxa, meeting recurrent stria, which extends anterad to meet anterior part of metepisternum; meso- and metaventral disks, as well as 1^st^ abdominal ventrite with numerous punctures separated by about their widths; 1^st^ abdominal ventrite with two complete lateral striae; protibiae very strongly toothed, each tooth with a long spine; all tarsi with apical tarsomere expanded, laterally flattened, pretarsal claws small, straight, parallel, approximate, extending from ventral corner of tarsomere (rather than apex); propygidium transverse, about half length of pygidium along midline, with intermixed punctures of various sizes, gland openings present in anterolateral corners, small; pygidium with uniform small punctures throughout, lacking apical marginal stria. Male not known.

#### Remarks.

This species is more elongate, especially markedly in pronotal shape, than other species of the group (Fig. 24A). It also lacks an apical marginal elytral stria, which the following two species have.

#### Etymology.

This species is named for its somewhat diving beetle-like body form.

### Operclipygus carinisternus sp. n.

urn:lsid:zoobank.org:act:C6CF0818-623D-4803-A768-9FF7D26CE76F

http://species-id.net/wiki/Operclipygus_carinisternus

Fig. 24D Map 8

#### Type locality.

GUYANA: Region 8: Iwokrama Forest Reserve [4°40.3'N, 58°41.1'W].

#### Type material.

**Holotype female**: “GUYANA: Region 8 Iwokrama Forest Res. 4°40'19"N, 58°41'04"W 100–200m V–VI.2001, R. Brooks & Z. Falin, FIT” / “Caterino/Tishechkin Exosternini Voucher EXO-00184” (SEMC). **Paratypes** (3): **FRENCH GUIANA:** 1: Belvèdére de Saül, point de vue, 3°1'22"N, 53°12'34"W, 20.xii.2010, Window trap, SEAG (MNHN), 2: 24.i.2011 (CHND, FMNH).

#### Other material.

**BRAZIL: Pará:** 1: Tucuruí, 3°45'S, 49°40'W, 27.x–9.xi.1985, FIT (CHND).

#### Diagnostic description.

This species is similar in most characters to the proceeding, differing as follows: length: 1.59–1.75 mm, width: 1.47–1.65 mm; body rufo-brunneus, more nearly rectangular, sides of elytra less strongly rounded, basal two-thirds of pronotal sides only barely convergent to front, abruptly narrowed but not sinuate to anterior corners; surfaces generally more densely punctate, especially pronotum, with large punctures becoming subconfluent posterolaterally; elytra similar to above, but with inner subhumeral stria merging with outer stria one-third from apex, each elytron with apical marginal stria running from apex of outer subhumeral stria to sutural; prosternal keel with carinal striae present, meeting anteriorly near midpoint of keel; prosternal lobe with marginal stria present or absent; mesoventral disk with mesometaventral stria composed of arched series of large punctures, barely connected to each other; metaventral disk with larger punctures, narrowly separated by half their diameters, lateral metaventral stria not or barely separated from recurrent stria in front of the metacoxa; pygidium with fine punctures more densely scattered, marginal stria complete, fine. Male not known.

#### Remarks.

*Operclipygus carinisternus* and *Operclipygus quadratus* are the most strongly punctate species in the group, and have a generally more quadrate body form. Together their complete apical elytral stria distinguishes them from the others in the group. *Operclipygus carinisternus* can be separated by the smoothly continuous anterior and lateral submarginal pronotal striae (Fig. 24D). In *Operclipygus quadratus* these meet at a postocular angulations (Figs 24E–F). One specimen from Brazil: Pará is excluded from the type series, differing in many fine details of puncture and sculpture. It may represent a distinct species, but given the limited material available at present we consider it to represent a distinct population of the present species.

#### Etymology.

This species’ name refers to the elevated ridge of the prosternal lobe.

### Operclipygus quadratus sp. n.

urn:lsid:zoobank.org:act:CEDAC319-6843-4944-96F5-7A7875B0D549

http://species-id.net/wiki/Operclipygus_quadratus

Figs 24E–F 25A–D Map 8

#### Type locality.

BRAZIL: Pará: Tucuruí [3°45'S, 49°40'W].

**Type material. Holotype male**: “**BRASIL: Pará:** Tucuruí, 3°45'S, 49°40'W. Piège d’interception. vi.1985”/ “Caterino/Tishechkin Exosternini Voucher EXO-00287” (UFPR). **Paratypes** (6): 1: same data as type (CHND); **FRENCH GUIANA:** 1: Belvèdére de Saül, point de vue, 3°1'22"N, 53°12'34"W, 20.xii.2010, FIT, SEAG (FMNH), 1: 4.i.2011, FIT, SEAG (MNHN); 1: Rés. Natur. des Nouragues, Camp Inselberg, 4°05'N, 52°41'W, 22.viii.2011, FIT, SEAG (CHND), 1: 25.viii.2011, FIT, SEAG (MSCC). 1: **GUYANA:** Kurupukari, 4°40'N, 58°40'W, ix–xi.1992, malaise/FIT (BMNH).

#### Other material.

**BRAZIL:** 2: **Mato Grosso:** Claudia, 11°24.5'S, 55°19.5'W, 17–27.x.2010, FIT, A.F. Oliveira (MSCC, FMNH); 1: Cotriguaçu, Fazenda São Nicolau, Mata Norte, 9°49.15'S, 58°15.6'W, 8–14.xii.2010, FIT, F.Z. Vaz-de-Mello (CEMT); **Pará:** 2: Tucuruí, 3°45'S, 49°40'W, vi.1985, FIT (CHND, AKTC).

#### Diagnostic description.

**Figure 25.**
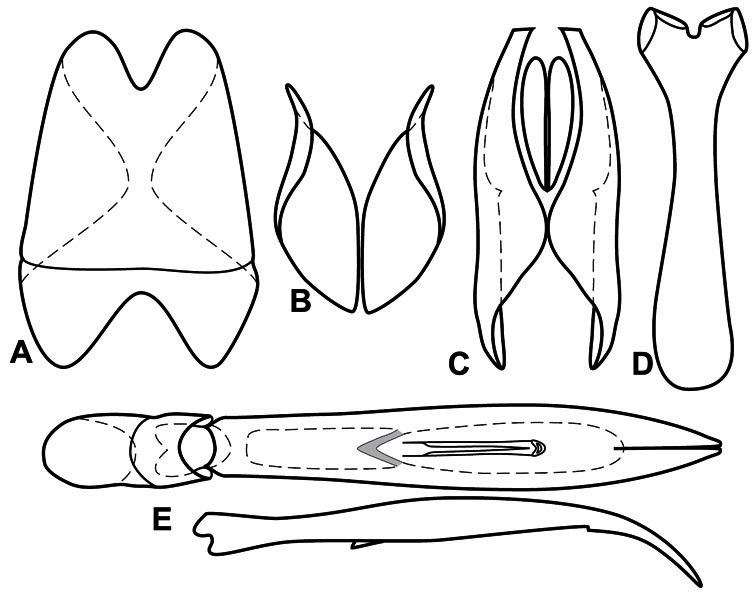
Male genitalia of *Operclipygus quadratus*. **A** T8 **B** S8 **C** T9 & T10 **D** S9 **E** Aedeagus, dorsal and lateral views.

Length: 1.93–2.00 mm, width: 1.34–1.56 mm; body rufobrunneus, broad, depressed, subquadrate, densely punctate on all surfaces; frons depressed at middle, frontal stria divergent, rounded between eyes, complete, transverse across front, continuous with complete supraorbital stria; epistoma broadly depressed, elevated at sides and along apical margin; labrum about half as long as wide, with sides rounded, apex shallowly emarginate; left mandible with prominent, acute basal tooth, right mandible untoothed, with apex frequently narrow, elongate; pronotal disk with indistinct prescutellar impression; marginal stria interrupted behind head; lateral submarginal pronotal stria complete, very close to margin, subcarinate, curving inward at front, meeting anterior submarginal stria at distinct postocular angulation; anterior submarginal stria transverse, ends not recurved posterad; median pronotal gland openings within postocular angulation of striae; elytra with all striae impressed as pair of dense rows of interconnected punctures; one complete epipleural stria present, outer subhumeral complete beneath strong lateral elytral margin (apparently representing complete inner subhumeral stria), dorsal striae 1-5 complete, sutural stria and marginal carina connected by a complete, fine apical marginal stria; prosternal keel produced posteriorly, truncate at base, carinal striae deeply impressed, meeting at acute angle in front; prosternal lobe with midline elevated, apex distinctly, narrowly emarginate, marginal stria interrupted by emargination; mesoventrite deeply emarginate anteriorly, marginal stria interrupted by anteriorly arched, deeply impressed, crenulate mesometaventral stria; lateral metaventral stria reaching middle of metacoxa; 1^st^ abdominal ventrite with two complete lateral striae; propygidium and pygidium similarly densely, uniformly covered with small punctures; marginal pygidial sulcus complete, fine. Male genitalia (Fig. 25): accessory sclerites absent; T8 rather short, sides moderately convergent to apex, basal apodemes rounded, basal emargination subangulate, basal membrane attachment line distad emargination by about one-third its depth, ventrolateral apodemes most strongly produced at middle, nearly meeting at midline, apical emargination simple; S8 very much like *Operclipygus hospes*, sides broadly rounded in basal two-thirds, apical guides narrow, only developed near apex, ventrally halves approximate for short distance near base, weakly diverging to apex; T9 with sides in contact for short distance along dorsal midline, apices narrow, truncate; T10 divided; S9 narrowest just basad middle, basal half strongly widened, broadly rounded, largely desclerotized, lateral flanges with prominent apical corners, apex with narrow, shallow median emargination, apical flanges small and separate; tegmen rather narrow, elongate, widest just distad midpoint, narrowed to base, apex subacute, with subapical, ventrolateral cleft, medioventral process long with very narrowly truncate apex projecting beneath about one-third from base; basal piece about one-third tegmen length; median lobe about one-third tegmen length, with filamentous portions of proximal apodemes not apparent.

#### Remarks.

This species is generally very similar to *Operclipygus carinisternus*, but can be separated by the angulate junction of the anterior and lateral submarginal pronotal striae behind the eye (Figs 24E–F). *Operclipygus quadratus* also lacks a recurrent stria on the lateral part of the metaventrite, which all the other species in the group have. While this species is highly distinctive, there is also a surprising amount of variation in several significant characters. In particular, a few individuals have mandibles with the apex of the incisor edge strongly prolonged and narrowed (Fig. 24F), and this might appear to represent sexual dimorphism. However, some males do not show this, even from the same locality as the holotype. So it seems to represent a more complex within-species polymorphism. Other variable characters include the degree of frontal depression, the depth of the prosternal lobe emargination, the size of elytral interstrial punctures, and presence of elytral microsculpture. We therefore delimit the type series very narrowly, in case this turns out to be a complex of closely related species.

*Operclipygus quadratus* shares significant characters with members of the *Operclipygus hospes* group, including the deeply emarginate mesoventrite with arched mesometaventral stria, the two complete lateral striae on the 1^st^ abdominal ventrite, the basally rounded S8 and by the shape of the tegmen and its ventral subapical cleft, and it may represent a transitional form linking the two groups, although phylogenetic analyses do not support this idea unambiguously.

#### Etymology.

The name of this species refers to its distinctly quadrate body form.

### Operclipygus crenulatus sp. n.

urn:lsid:zoobank.org:act:1050B57B-F2C1-4A63-9004-ECE843750F91

http://species-id.net/wiki/Operclipygus_crenulatus

Figs 24G–H Map 8

#### Type locality.

BRAZIL: Amapá:Serra do Navio [0°59'N, 52°00'W].

#### Type material.

**Holotype female**: “**BRASIL: Amapá:** Serra do Navio 0°59'N, 52°00'W Piège d’interception 17–30.ix.1991” / “Caterino/Tishechkin Exosternini Voucher EXO-00185” (UFPR).

#### Diagnostic description.

This species is similar in most characters to the preceding two, differing as follows: length: 1.72 mm, width: 1.44 mm; body rufescent, with sides rounded, not particularly elongate, finely punctate, lacking coarse punctures on pronotum and elytra; pronotal sides moderately convergent in basal two-thirds, curving inward to anterior corners; central portion of anterior marginal stria strongly crenulate, marginal bead not markedly elevated; elytral striae not so broadly impressed, with only a single epipleural stria, inner subhumeral stria completely absent, 5^th^ stria obsolete in basal third, sutural stria obsolete in basal fourth, other striae complete; apical marginal elytral stria absent; prosternal keel more distinctly rounded at base, with fine, complete carinal striae remaining separate to presternal suture; marginal stria of prosternal lobe present, interrupted at middle by median ridge; mesoventrite emarginate in front, with fine, complete marginal stria; mesometaventral stria weakly and narrowly arched forward at middle, central arch isolated from lateral metaventral striae, which are fine, continuous with recurrent stria; propygidium with sparse coarse punctures separated by slightly more than their diameters; pygidial punctures finer, uniform; apical marginal stria of pygidium complete; apical tarsomeres more or less cylindrical, not expanded or laterally flattened; pretarsal claws longer, divergent.

#### Remarks.

This species is quite distinct in the group, not so distinctly punctate (Fig. 24G) and with plesiomorphic, simple apical tarsomeres. However, the distinctive prosternal lobe (Fig. 24H) associates it with little doubt.

#### Etymology.

The name of this species refers to its crenulate anterior marginal pronotal stria.

### *Operclipygus dubitabilis* group

**Figure 26.**
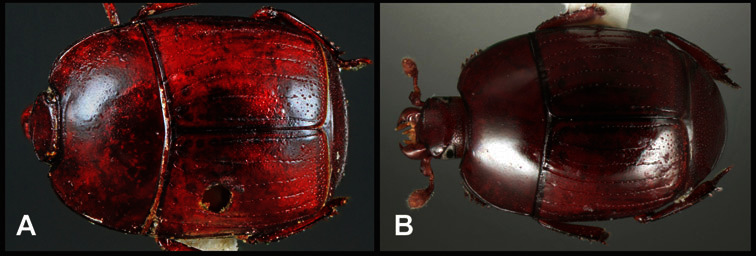
*Operclipygus dubitabilis* group. **A** Dorsal habitus of *Operclipygus dubitabilis* **B** Dorsal habitus of *Operclipygus yasuni*.

The two species in this group can be recognized fairly easily by overall body shape alone, being rather broadly oval and subdepressed (Figs 26A–B). A small, but distinct punctiform prescutellar impression is unique, as is the combination of a lateral submarginal pronotal stria close to the marginal stria, the nearly complete pattern of elytral striation (only the sutural stria may be basally abbreviated), and the presence of coarse apical elytral punctures.

### Key to the species of the *Operclipygus dubitabilis* group

**Table:** 

1	Epistoma depressed, margined at sides by oblique strioles continuous with frontal stria	*Operclipygus dubitabilis* (Marseul)
–	Epistoma convex, lacking lateral striae; frontal stria simple across front	*Operclipygus yasuni* sp. n.

### Operclipygus dubitabilis

(Marseul, 1889)

http://species-id.net/wiki/Operclipygus_dubitabilis

Fig. 26A Map 9

Phelister dubitabilis Marseul, 1889: 126; *Operclipygus dubitabilis*: Wenzel (1976: 258).

#### Type locality.

BRAZIL: Amazonas: Tefé [3°22'S, 64°42'W].

**Type material. Lectotype** hereby designated: “Ega” / “Marseul, 14.12.86” / “*dubitabilis*” / “*Phelister dubitabilis* Mars., Type.” / “G.Lewis Coll. B.M.1926-369” / “LECTOTYPE *Phelister dubitabilis* Marseul, M.S. Caterino & A.K. Tishechkin des. 2010” (BMNH). This species was described from an unspecified number of specimens, and the lectotype designation fixes primary type status on the only known original specimen.

#### Other material.

**BRAZIL: Minas Gerais:** 1: Ingaí, Res. Boqueirão, nr. Lavras, 21°21'S, 44°59'W, 5.xi.2002, FIT, gallery forest, F. Freiro-Costa & F.Z. Vaz-de-Mello (FMNH), 2: 27.xi.2002, FIT, R.J. Silva (AKTC, MSCC).

#### Diagnostic description.

Length: 2.46–2.53 mm, width: 2.03–2.15 mm; body rufo-brunneus, broadly elongate oval, subdepressed, weakly convex above; frons broad, with sides of frontal stria divergent anterad, frontal disk depressed behind carinate, arcuate, complete frontal stria; epistoma depressed between fragments of oblique lateral striae which vaguely meet frontal stria; labrum short, emarginate; mandibles without strong inner teeth; pronotal sides weakly convergent in basal two-thirds, rounded to frontal angles, with a shallow prescutellar depression bearing a single median puncture; lateral marginal pronotal stria complete at sides and continuous across front; lateral submarginal pronotal stria complete at sides, deeply depressed along its inner edge, ending freely after turning mediad at front; anterior submarginal stria present, with sides weakly divergent from anterior margin; median pronotal gland openings anterad ends of submarginal stria, about 5 puncture widths from anterior margin; pronotal disk shallowly punctato-rugose along sides; elytra with two complete epipleural striae; inner and outer subhumeral striae, as well as dorsal striae 1-5 complete to front (4 and 5 slightly fragmented at apices), 5^th^ arched toward suture at base, sutural stria absent from basal fourth; elytral disks with coarse apical punctures in apical fourth; prosternal keel truncate at base, with carinal striae in basal three-fourths meeting in narrow anterior arch, faint secondary striae present alongside carinal striae; prosternal lobe short, wide, extending to hypomera, its marginal stria present only at middle; mesoventrite very shallowly emarginate in front, with complete marginal stria; mesometaventral stria arched strongly forward, nearly meeting marginal mesoventral, continued by lateral metaventral stria nearly to metacoxa, then curved laterad, ending short of metepisternum; metaventral disk with fine ground punctures and very fine, transverse microsculpture; 1^st^ abdominal ventrite with single lateral stria; propygidium with conspicuous, more or less transverse waves of microsculpture, with fine ground punctures sparse, and coarser, round punctures uniformly separated by about their diameters; pygidium similar to propygidium with slightly higher density of coarse punctures, with marginal stria complete or slightly fractured near basal corners. Male not known.

#### Remarks.

This species may be easily distinguished from the following by its depressed epistoma with oblique lateral strioles.

### Operclipygus yasuni

sp. n.

urn:lsid:zoobank.org:act:7C4D8650-EC57-4954-83D7-9987EDE8F452

http://species-id.net/wiki/Operclipygus_yasuni

**Map 9.**
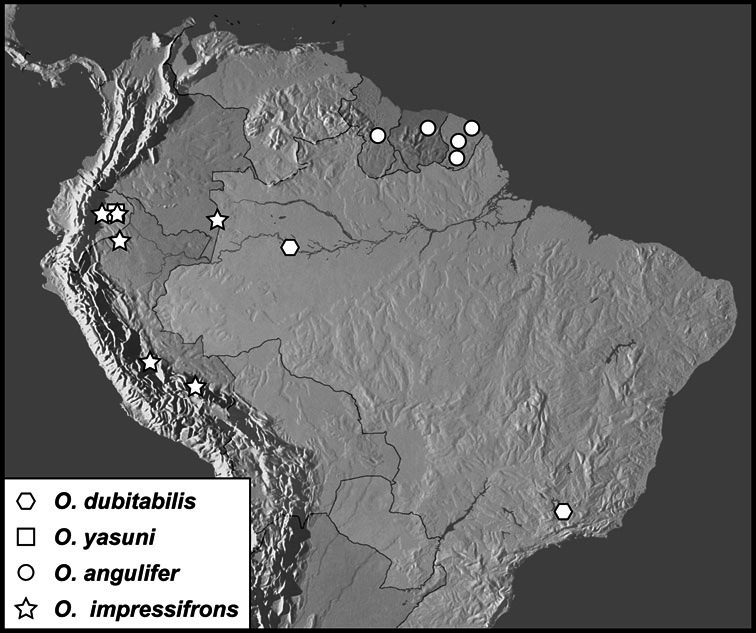
Records of the *Operclipygus dubitabilis* and *Operclipygus impressifrons* groups

Figs 26B 27A–E Map 9

#### Type locality.

ECUADOR: Orellana:Yasuní Research Station [0°40.5'S, 76°24'W].

#### Type material.

**Holotype male**: “**Ecuador:** Napo, mid.Rio Tiputini, Yasuní Res. Stn. 0°40.5'S, 76°24'W, Dead fish trap, late stage. 24–26 Jun 1999. CEC#024 C.Carlton” / “LSAM 0045445” / “*Phelister* sp. #21, Hist 161 Yasuní NP Inventory, A.K.Tishechkin det. 2010” (FMNH).

#### Diagnostic description.

**Figure 27.**
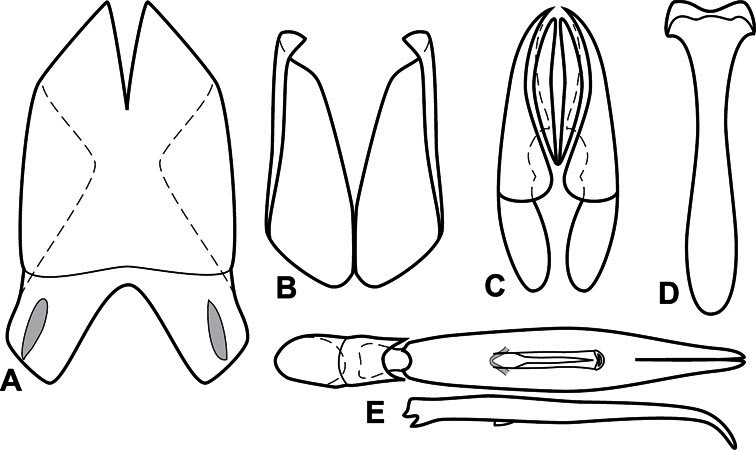
Male genitalia of *Operclipygus yasuni*. **A** T8 **B** S8 **C** T9 & T10 **D** S9 **E** Aedeagus, dorsal and lateral views.

This species is extremely similar to *Operclipygus dubitabilis*, differing as follows: length: 2.18 mm, width: 1.90 mm; frontal stria fragmented at middle; epistoma not at all depressed, lacking fragments of lateral striae; pronotum with lateral submarginal stria shallowly impressed, disk not depressed mediad; pronotal disk with only few small coarse lateral punctures; pygidium lacking marginal stria, with barely visible crease near apex. Male genitalia (Fig. 27): accessory sclerites present; T8 elongate, parallel-sided, basal apodemes only slightly divergent, basal emargination narrow, deep, with basal membrane attachment line distad by about one-fourth basal emargination depth, apical emargination narrow; ventrolateral apodemes narrow, well separated beneath; S8 with apical guides narrow for most of length, abruptly widened at apex, halves approximate in basal fourth, diverging to apex; T9 with apices curving, subacute; T10 with halves separate; S9 narrow at middle, evenly expanded to narrowly rounded base, apex shallowly arcuate, without distinct median emargination, with apical flange narrowed at middle; tegmen slender, with rounded sides slightly narrowing to base, narrowing in apical half to subacute apices, apex moderately curved ventrad; medioventral process small but well sclerotized, ‘U’-shaped, situated about one-fourth tegmen length from base, projecting beneath; basal piece about one-third tegmen length; median lobe about one-third tegmen length, slender.

#### Remarks.

This species can be distinguished from the former primarily by the convex epistoma and simple frontal stria.

#### Etymology.

This species is named, as a noun in apposition, for the Yasuní Research Station, the type locality for this species, and the source for numerous specimens of a great diversity of Exosternini.

### *Operclipygus impressifrons* group

**Figure 28.**
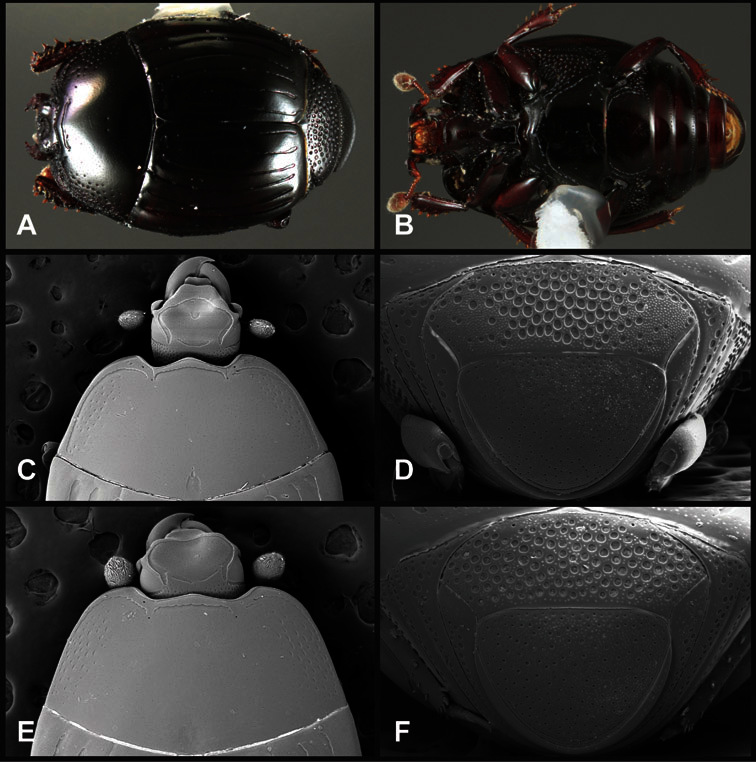
*Operclipygus impressifrons* group. **A** Dorsal habitus of *Operclipygus angulifer* **B** Ventral habitus of *Operclipygus impressifrons* **C** Frons & pronotum of *Operclipygus angulifer* **D** Pygidia of *Operclipygus angulifer* **E** Frons & pronotum of *Operclipygus impressifrons* **F** Pygidia of *Operclipygus impressifrons*.

The two species of this group are rather large and convex, with their frons very strongly depressed in the middle (Figs 28A, C, E). The central portion of the anterior pronotal margin is strongly angulate medially, and the prosternal keel is relatively broad, with its base weakly emarginate (Fig. 28B).

### Key to the species of the *Operclipygus impressifrons* group

**Table:** 

1	Dense ground punctation of propygidium especially evident at sides, where coarse punctures are few (Fig. 28D); lateral submarginal stria of pronotum entire along side (Fig. 28C); basal arch between 4^th^ dorsal and sutural elytral striae fragmented	*Operclipygus angulifer* sp. n.
–	Dense ground punctation of propygidium not especially evident at sides, coarse punctures uniformly dense throughout (Fig. 28F); lateral submarginal stria of pronotum generally interrupted at middle (Fig. 28E); basal arch of 4^th^ dorsal elytral stria usually complete to suture (though rarely connected to abbreviated sutural stria)	*Operclipygus impressifrons* sp. n.

### Operclipygus angulifer sp. n.

urn:lsid:zoobank.org:act:BBD7AF23-E960-405E-AF39-11186B9C6AD2

http://species-id.net/wiki/Operclipygus_angulifer

Figs 28A–D 29A–C, E Map 9

#### Type locality.

FRENCH GUIANA: 27.4 km SSE Roura [4°44.3'N, 52°13.4'W].

#### Type material.

**Holotype male**: “FRENCH GUIANA Roura, 27.4 km SSE, 280 m 4°44'20"N, 52°13'25"W 25–29 MAY 1997; J.Ashe,R.Brooks FG1AB97 079 ex:flight intercept trap” / “SM0096393 KUNHM-ENT” (SEMC). **Paratypes** (14): **FRENCH GUIANA:** 2: Roura, 27.4km SSE, 4°44'20"N, 52°13'25"W, 280m, 10.vi.1997, FIT, J. Ashe, R. Brooks (SEMC, FMNH); 1: Roura, 18.4km SSE, 4°36'38"N, 52°13'25"W, 240m, 29.v–10.vi.1997, FIT, J. Ashe, R. Brooks (SEMC); 1: Saül, 7km N, Les Eaux Claires, 3°39'46"N, 53°13'19"W, 220m, 31.v–3.vi.1997, FIT, J. Ashe, R. Brooks (SEMC); 1: Cayenne, 33.5km S and 8.4km NW of Hwy N2 on Hwy D5, 4°48'18"N, 52°28'41"W, 30m, 29.v–9.vi.1997, FIT, J. Ashe, R. Brooks (SEMC); 1: Mont tabulaire, Itoupé, 3°1.32'N, 53°5.05'W, 800m, 17.iii.2010, FIT, SEAG (MNHN), 1: 24.iii.2010, FIT, SEAG (CHND); 1: Matoury (41.5km SSW: 4°37'22"N, 52°22'35"W, 50m, 26–28.v.1997, FIT, J. Ashe & R. Brooks (SEMC); 2: Rés. Natur. des Nouragues, Camp Inselberg, 4°05'N, 52°41'W, 25.i.2011, FIT, SEAG (CHND, MSCC). **GUYANA: Region 8**: 1: Iwokrama Field Stn., Iwokrama Forest, 1km W Kurupukari, 4°40'19"N, 58°41'4"W, 60m, 26–29.v.2001, FIT, R. Brooks & Z. Falin (SEMC); 1: Kabocalli Field Stn., Iwokrama Forest, 4°17'4"N, 58°30'35"W, 60m, 3–5.vi.2001, FIT, R. Brooks & Z. Falin (SEMC); 1: Iwokrama Forest, Pakatau hills, 4°44'54"N, 59°1'36"W, 70m, 25–29.v.2001, FIT, R. Brooks & Z. Falin (AKTC). **SURINAME: Brokopondo:** 1: Brownsberg Nature Preserve, Witi Creek Trail, 4°56'55"N, 55°10'53"W, 480m, 23–25.vi.1999, FIT, Z. Falin, A. Gangadin, H. Hiwat (SEMC).

#### Diagnostic description.

**Figure 29.**
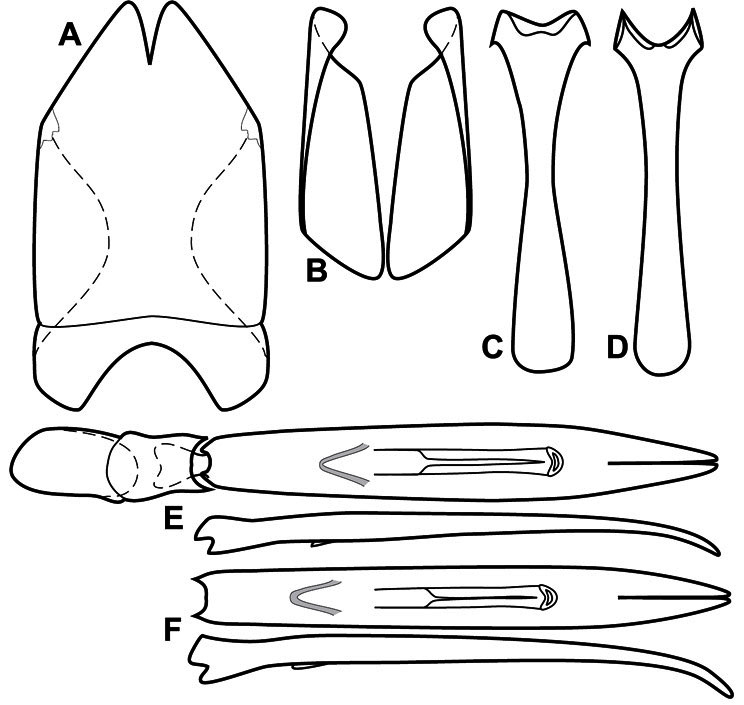
Male genitalia of *Operclipygus impressifrons* group. **A** T8 of *Operclipygus angulifer* **B** S8 of *Operclipygus angulifer* **C** S9 of *Operclipygus angulifer* **D** S9 of *Operclipygus impressifrons* **E** Aedeagus, dorsal and lateral views, of *Operclipygus angulifer* **F** Aedeagus, dorsal and lateral views, of *Operclipygus impressifrons*.

Length: 2.25–2.50 mm, width: 1.87–2.06 mm; body rufo-piceous, elongate oval, widest near middle of elytra; head with frons strongly and narrowly depressed at middle, especially behind frontal stria but also onto epistoma, with pronounced swellings above antennal bases; frontal stria diverging anterad at sides, sinuate over swellings, strongly arcuate at middle; supraorbital stria fragmented, not connected to frontal stria; epistoma elevated above labrum and mandibles, flat anteriorly becoming concave; labrum twice as wide as long, sides convergent, apex emarginate; left mandible untoothed, right mandible with acute basal tooth; sides of pronotum evenly narrowed in posterior two-thirds, rounded to apices; pronotum with shallow but distinct prescutellar impression, irregularly oval, about size of scutellum, disk with fine but conspicuous ground punctation, with ~15 coarse punctures close to sides; lateral marginal stria sinuate two-thirds from base, rising from side to dorsum of pronotum, continuous anteriorly along acutely projecting anterior pronotal margin; lateral submarginal stria complete at sides, curved inward at front, ending freely behind eye; anterior submarginal stria more or less straight across front, ends barely recurved posterad at sides, median pronotal gland openings laterad its ends; elytra with sides strongly swollen laterad 1^st^ dorsal stria, most intervals markedly convex, with one complete epipleural stria; outer subhumeral stria present in posterior half, interrupted at middle, with isolated fragment in basal half, rarely uninterrupted; inner subhumeral stria usually absent, rarely represented by short fragment near middle, striae 1- 4 complete, 4^th^ stria usually with small anterior ‘hook’, 5^th^ stria present in apical half to two-thirds, with isolated basal arch, sutural stria present in apical four-fifths, distinctly more broadly impressed toward front; prosternal keel broad at base, shallowly emarginate, with carinal striae sinuately convergent, united near presternal suture, with short, faint secondary striae present behind prosternal gland openings; prosternal lobe rather short, with complete marginal stria, and prominent marginal bead; anterior mesoventral margin sinuate, bluntly projecting at middle, with marginal stria interrupted for about width of prosternal keel; central part of mesometaventral stria detached from lateral metaventral, arched strongly forward at sides, arcuate just behind marginal mesoventral stria; postmesocoxal stria extended slightly inward along mesometaventral suture, lateral metaventral stria replacing it medially, median ends of lateral metaventral nearly meeting at midline, posterolaterally extending toward inner corner of metacoxa; central part of metaventral disk impunctate; 1^st^ abdominal ventrite with complete inner lateral stria, outer stria obsolete in posterior half; ventrites 2-4 with single, very regular series of small punctures along their posterior margins; propygidum about half length of pygidium along midline, with dense ground punctation, especially evident at sides, with large punctures separated by about one-fourth their diameters mainly concentrated in middle half and along anterior margin; pygidium with fine, dense ground punctation, and small punctures sparsely intermixed; marginal stria fine, present only at extreme apex of pygidium. Male genitalia (Figs 29A–C, E): accessory sclerites absent; T8 with sides straight, weakly convergent in basal three-fourths, angled to apex, basal emargination broad, shallow, nearly reaching basal membrane attachment line, apical emargination narrow, apices acute; S8 with sides sinuately convergent to apex, guides narrow, even in width throughout length, rounded apically, ventrally halves approximate just at base, diverging apically; T9 with sides weakly rounded, moderately converging toward apex, apices acute, not opposing; T10 with halves separate; S9 narrowest in basal half, sides evenly widening to base and apex, base rounded, narrowly desclerotized; apex inwardly angulate, but without distinct median emargination, apical flange continuous though narrowed at middle; tegmen narrow, elongate, more or less parallel-sided, narrowing in apical one-fifth to subacute apex, medioventral process lightly sclerotized, narrowly ‘U’-shaped, weakly projecting beneath about one-fourth from base; basal piece just over one-third tegmen length; median lobe about one-half tegmen length.

#### Remarks.

This distinctive species is easily distinguished by the very strongly projecting pronotal margin and the strongly depressed frons (Figs 28A, C). The following species is similar, and closely related, but is less extreme in both of these characters. *Operclipygus angulifer* also exhibits basal fragment of the outer subhumeral stria, and has the propygidial punctures sparse toward the lateral portions of the disk (Fig. 28D).

#### Etymology.

This species’ name refers to the distinctively angulate anterior pronotal margin.

### Operclipygus impressifrons sp. n.

urn:lsid:zoobank.org:act:A8053F96-4810-4A56-9562-B9F1B8D55C3F

http://species-id.net/wiki/Operclipygus_impressifrons

Figs 28E–F 29D, F Map 9

#### Type locality.

ECUADOR: Orellana:Yasuní Research Station [0°40.5'S, 76°24'W].

#### Type material.

**Holotype male**: “**Ecuador:** Napo, mid.Rio Tiputini, Yasuní Res. Stn. 0°40.5'S, 76°24'W, FIT#4. 5–14 July 1999. AKT#073 A.Tishechkin” / “LSAM 0013194” / “*Operclipygus* sp. #8, Hist 130 Yasuní NP Inventory, A.K.Tishechkin det. 2010” (FMNH). **Paratypes** (14): 8: same locality as type, differences as noted: 1: 28.vi–7.vii.1999 (LSAM), 1: 25–30.vii.1999 (FMNH), 1: 23–30.vi.1999 (MSCC), 1: 25.vii–4.viii.1999 (AKTC), 1: 28.vi–5.vii.1999 (LSAM), 1: 5–11.vii.1999 (LSAM), 2: 215m, 5–10.ix.1999, E.G. Riley (TAMU); 1: Tiputini Biodiversity Station, 0.6376°S, 76.1499°W, 4–9.vi.2011, FIT, M.S. Caterino & A.K. Tishechkin, DNA Extract MSC-2173 (SBMNH), 3: 2–9.vi.2011 (USFQ, CHND); 1: Payamino Research Station, 0°29'36.01"S, 77°17'29.15"W, 300m, 30.vii–12.viii.2007, Malaise trap, tropical rainforest, CPDT Gillett (BMNH); **Sucumbíos:** 1: Sacha Lodge, 0°28'14"S, 76°27'35"W, 270m, 21–24.iii.1999, FIT, R. Brooks (SEMC).

#### Other material.

**COLOMBIA: Vaupés:** 1: Est. Biol. Caparú, Rio Apoporis, 1.1°S, 69.5°W, 27.ix–1.xii.1995, FIT, Black-water terrace forest on sandy soils, B.D. Gill (BDGC); 7: Parque Nac. Mosiro-Itajura (Caparú), Centro Ambiental, 1°04'S, 69°31'W, 60m, 20–30.i.2003, FIT, D. Arias & M. Sharkey (MSCC, AKTC, IAVH). **PERU: Cusco:** 1: Cock of the Rock Lodge, NE Paucartambo, 13°3.3'S, 71°32.7'W, 1120m, 4–9.xi.2007, FIT, D. Brzoska (SEMC); **Junín:** 1: ~16km NW Satipo, Rio Venado, 11°11.677'S, 74°46.137'W, 1150m, 3–8.iii.2010, A.V. Petrov (MUSM); **Loreto:** 1: 1.5km N Teniente Lopez, 2°35.66'S, 76°6.92'W, 210–240m, 18.vii.1993, FIT, R. Leschen (SEMC).

#### Diagnostic description.

This species is very similar to *Operclipygus angulifer*, differing in the following characters: length: 2.25–2.50 mm, width: 1.87–2.06 mm; frons less deeply depressed at middle, frontal stria usually interrupted on each side, isolating central portion; pronotum with prescutellar impression slightly larger than scutellum; lateral submarginal pronotal stria frequently interrupted along side, present at base and apex; elytra with 4^th^ dorsal stria abruptly angled inward at base toward, rarely connected with, base of sutural stria; mesoventrite with marginal stria complete; mesometaventral stria usually more or less continuous with lateral metaventral stria, the latter never extending inward to near the midline; propygidium with larger punctures extending to sides, ground punctation not conspicuous; coarser punctures of pygidium larger and more numerous; marginal pygidial stria fine, restricted to apical half of pygidial margin or less. Male genitalia (Figs 29D, E) extremely similar to those of *Operclipygus angulifer*, but with S9 slightly narrower, and with the tegmen more nearly parallel-sided throughout its length.

#### Remarks.

This species can best be distinguished from the preceding by its less deeply impressed frons (Fig. 28E), its basally weak or obsolete outer subhumeral stria, and the basal arch between the 4^th^ dorsal elytral stria and the suture. There is considerable variation in several characters, such as the completeness of the lateral submarginal pronotal stria, and in the length of the 5^th^ elytral stria. While all populations share the typical characters of the species, we restrict the type series to specimens from the Napo region of Ecuador.

#### Etymology.

This species’ name refers to its impressed frons.

### *Operclipygus dubius* group

**Figure 30.**
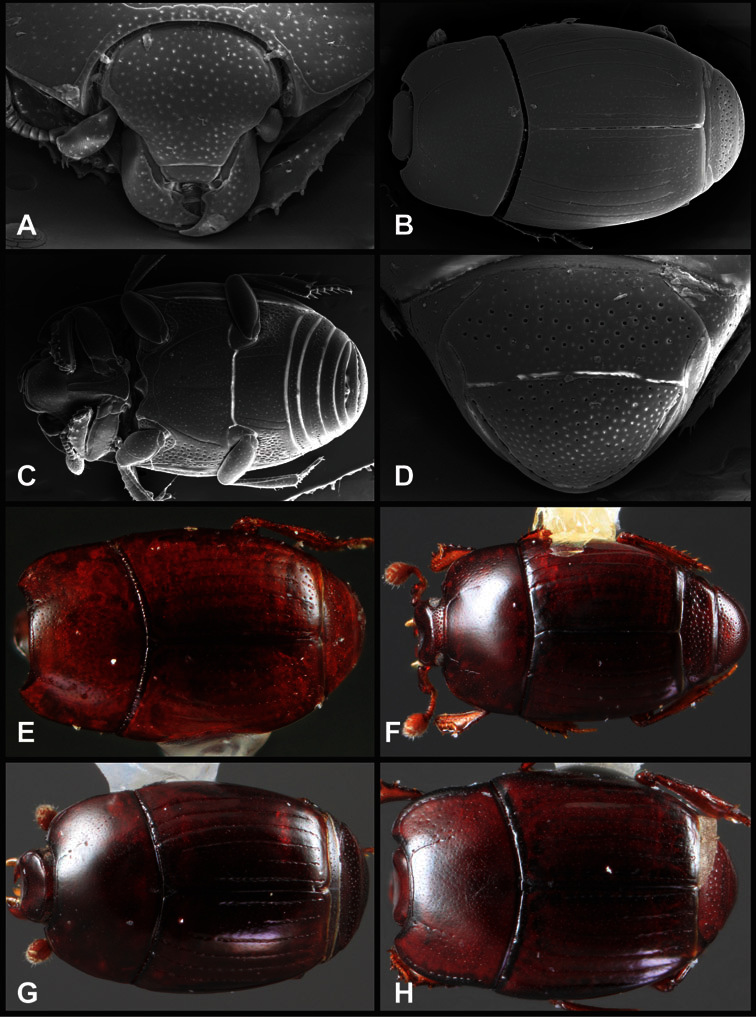
*Operclipygus dubius* group. **A** Frons of *Operclipygus dubius* **B** Dorsal habitus of *Operclipygus dubius* **C** Ventral habitus of *Operclipygus dubius* **D** Pygidia of *Operclipygus dubius* **E** Dorsal habitus of *Operclipygus dubius* (lectotype) **F** Dorsal habitus of *Operclipygus variabilis* **G** Dorsal habitus of *Operclipygus lunulus* **H** Dorsal habitus of *Operclipygus extraneus*.

**Figure 31.**
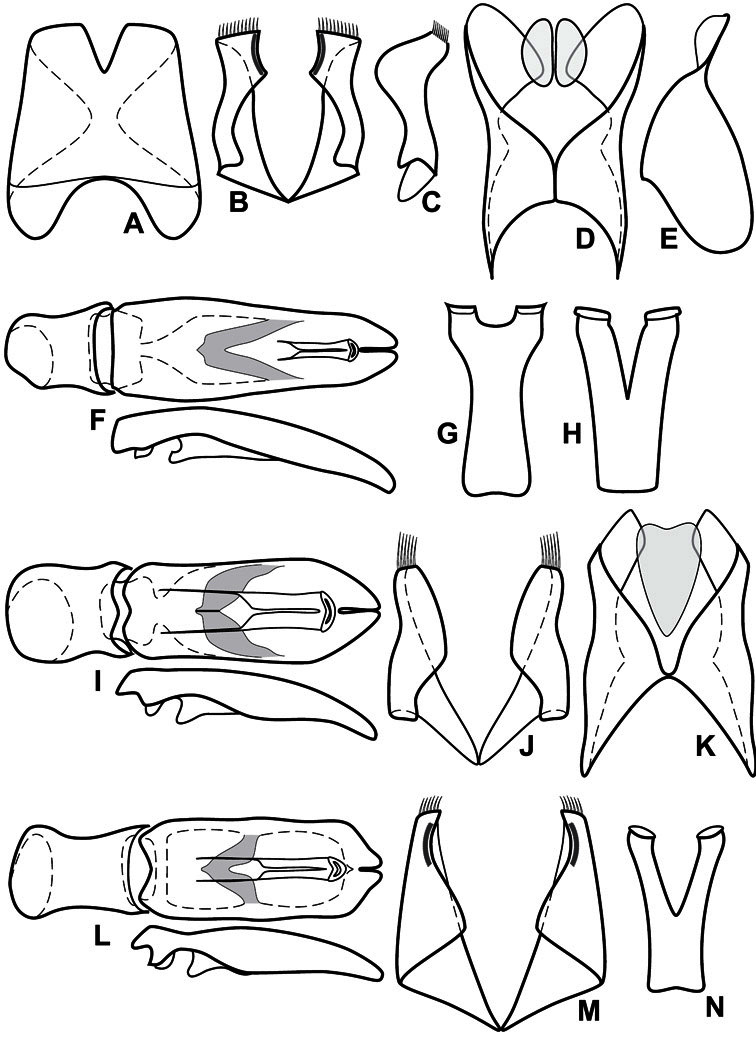
Male genitalia of *Operclipygus dubius* group. **A** T8 of *Operclipygus dubius* **B** S8 of *Operclipygus dubius*, dorsal view **C** S8 of *Operclipygus dubius*, lateral view, **D** T9 & T10 of *Operclipygus dubius*, dorsal view **E** T9 of *Operclipygus dubius*, lateral view **F** Aedeagus, dorsal and lateral views, of *Operclipygus dubius* **G** S9 of *Operclipygus dubius* **H** S9 of *Operclipygus andinus* **I** Aedeagus, dorsal and lateral views, of *Operclipygus andinus* **J** T9 & T10 of *Operclipygus andinus* **K** S8 of *Operclipygus andinus* **L** Aedeagus, dorsal and lateral views, of *Operclipygus intermissus* **M** S8 of *Operclipygus intermissus* **N** S9 of *Operclipygus intermissus*.

**Figure 32.**
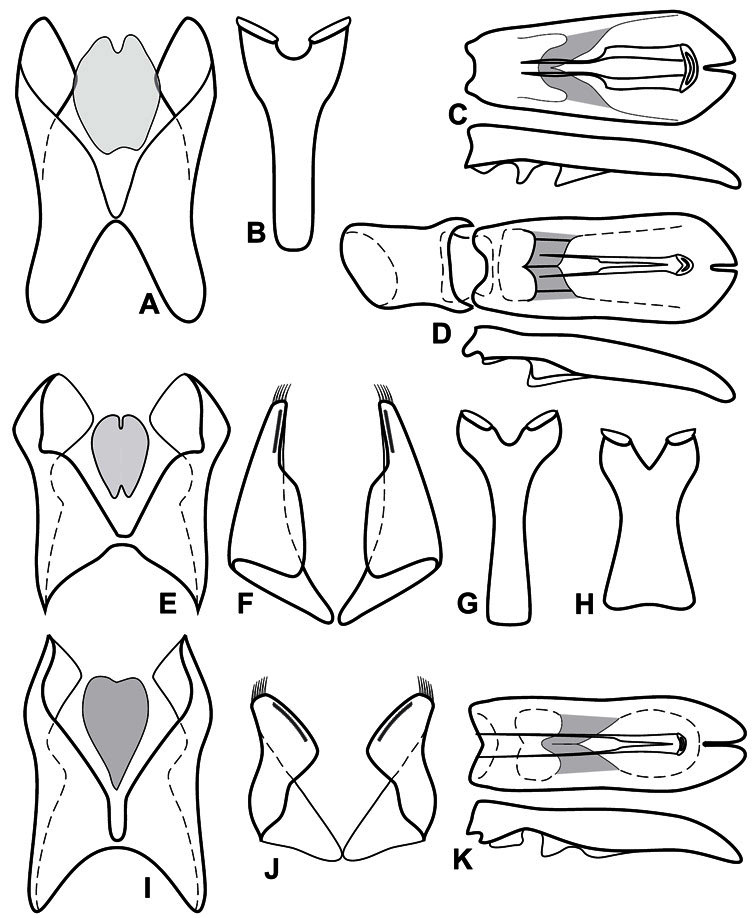
Male genitalia of *Operclipygus dubius* group. **A** T9 & T10 of *Operclipygus variabilis* **B** S9 of *Operclipygus variabilis* **C** Aedeagus, dorsal and lateral views, of *Operclipygus variabilis* **D** Aedeagus, dorsal and lateral views, of *Operclipygus validus* **E** T9 & T10 of *Operclipygus validus* **F** S8 of *Operclipygus validus* **G** S9 of *Operclipygus validus* **H** S9 of *Operclipygus lunulus* **I** T9 & T10 of *Operclipygus lunulus* **J** S8 of *Operclipygus lunulus* **K** Aedeagus, dorsal and lateral views, of *Operclipygus lunulus*.

**Figure 33.**
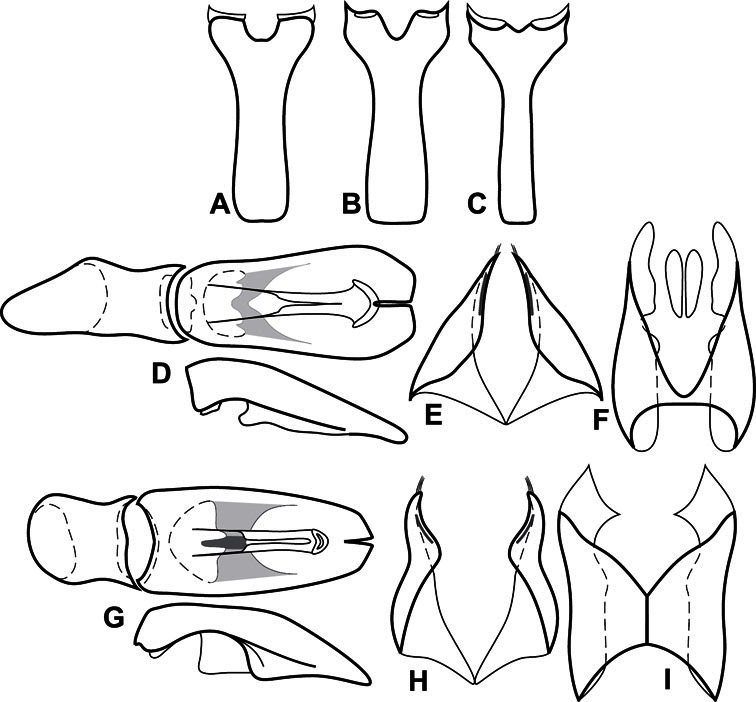
Male genitalia of *Operclipygus dubius* group. **A** S9 of *Operclipygus remotus* **B** S9 of *Operclipygus occultus* **C** S9 of *Operclipygus perplexus* **D** Aedeagus, dorsal and lateral views, of *Operclipygus occultus* **E** S8 of *Operclipygus occultus* **F** T9 & T10 of *Operclipygus occultus* **G **Aedeagus, dorsal and lateral views, of *Operclipygus perplexus* **H** S8 of *Operclipygus perplexus* **I** T9 of *Operclipygus perplexus*.

**Figure 34.**
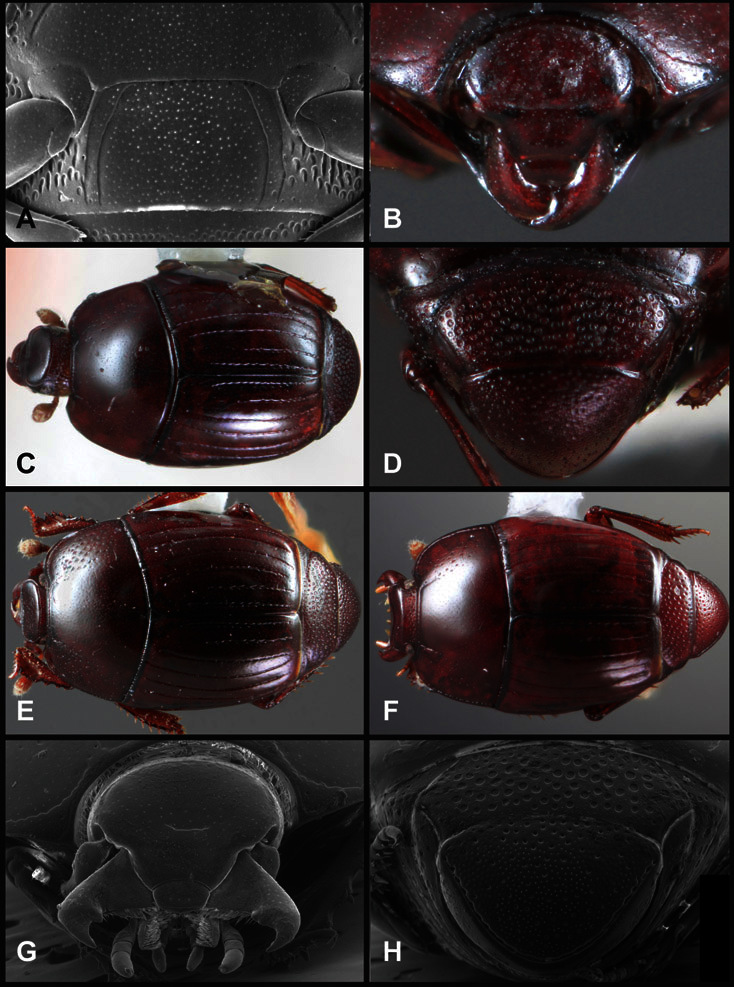
*Operclipygus hospes* group. **A** Metaventrite and 1^st^ abdominal ventrite of *Operclipygus fusistrius* **B** Frons of *Operclipygus hospes* (lectotype) **C** Dorsal habitus of *Operclipygus hospes* (lectotype) **D** Pygidia of *Operclipygus hospes* (lectotype) **E **Dorsal habitus of *Operclipygus ignifer* **F** Dorsal habitus *Operclipygus subterraneus* **G** Frons of *Operclipygus subterraneus* **H** Pygidia of *Operclipygus subterraneus*.

The *Operclipygus dubius* and *Operclipygus hospes* groups are together fairly distinctive within the genus. The first and most obvious feature they share is that most members of both groups are quite small, generally <2 mm body size. This isn’t entirely outside the range of other *Operclipygus*, but is markedly smaller than the majority. Species in both groups also tend to be slightly more elongate, with the sides of the body nearly or fully subparallel, and the entire body subdepressed (Figs 30, 34C, E). There are, however, exceptions to all these. Male genitalia provide the best characters for recognizing and delimiting these groups, and are highly distinctive within the *Operclipygus dubius* group, in many details (Figs 31–33). The entire genital capsule is relatively short and quadrate. T8 lacks basal accessory sclerites. S8 has its halves strongly divergent from the base. Its apices are highly varied in shape, but frequently have a peculiar subapical sclerotization paralleling the upper inside edge of the apical guides, and the apices usually have a dense brush of setae. T9 has the inner upper edges fused at the bases, then strongly divergent, with the ventrolateral apodemes apparently displaced apicad and bent inward, forming a sort of apicoventral flange on each side, the shape of which varies considerably among the species. T10 is frequently but not always fused along the midline. S9 is frequently but not always short and broad, with a deep medioapical emargination. The aedeagus itself is very short and wide, and has the medioventral process developed into a very strong ventral tooth or keel.

Externally, on the other hand, the *Operclipygus dubius* and *Operclipygus hospes* groups are remarkably similar, and no characters have been found to consistently distinguish them. In general the following help to distinguish species in the *Operclipygus dubius* group: left mandible with distinct basal tooth (Fig. 30A); antennal club tending to be large, circular; anterior mesoventral emargination almost meeting anterior arc of mesometaventral stria (Fig. 30C), the marginal mesoventral stria usually interrupted at middle; lateral metaventral stria more nearly longitudinal, extending toward inner third of metacoxa; propygidium strongly transverse; pygidium rather short, generally distinctly wider than long (Fig. 30D). In addition, most of the species have the lateral submarginal pronotal stria largely obsolete, although it is complete in more than one species. Finally, most have the recurved arms of the anterior submarginal pronotal stria long, thin, slightly oblique, and frequently disrupted by median pronotal gland openings (Figs 30F–G).

### Key to the species of the *Operclipygus dubius* group

**Table:** 

1	Lateral submarginal pronotal stria absent or represented by few fragments at anterior corner	3
–	Lateral submarginal pronotal stria complete	2
2	Central portion of frontal stria present (may be interrupted at sides); body narrowly elongate; weakly depressed	*Operclipygus perplexus* sp. n.
–	Central portion of frontal stria absent; body, especially pronotum, broad, subdepressed (Fig. 30H)	*Operclipygus extraneus* sp. n.
3	Recurved ends of anterior submarginal pronotal stria long, reaching around midpoint of pronotal length, may be interrupted by median pronotal gland openings	5
–	Recurved ends of anterior pronotal stria short, abbreviated in anterior fifth of pronotal length	4
4	No fragments of lateral submarginal pronotal stria present; pygidium with coarse punctures interspersed with ground punctation throughout; Ecuadorian Andes	*Operclipygus andinus* sp. n.
–	Few fragments of lateral submarginal pronotal stria present in anterior corners; pygidium lacking coarse punctures beyond basal third; Panama	*Operclipygus lunulus* sp. n.
5	Pronotal prescutellar mark represented by long striole occupying posterior fifth of pronotal length (Fig. 30B)	*Operclipygus dubius* (Lewis)
–	Pronotal prescutellar mark represented by short striole reaching at most posterior eighth of pronotal length	6
6	Lateral submarginal pronotal stria completely absent	*Operclipygus intermissus* sp. n.
–	At least a few fragments of lateral submarginal pronotal stria present near anterior corners	7
7	Frontal stria complete	*Operclipygus occultus* sp. n.
–	Central portion of frontal stria detached from sides	8
8	Aedeagus parallel-sided to near apex (as in Fig. 32K); apices of T9 obliquely rectangular, apically acute (as in Fig. 32I); Panama	*Operclipygus remotus* sp. n.
–	Aedeagus widened to near apex (Figs 32C–D); Amazonia and Andean foothills	9
9	4^th^ dorsal stria more distinctly (though variably) abbreviated at base; sides of pronotum with few or no coarse punctures; apices of T9 subquadrate, apices blunt (Fig. 32E)	*Operclipygus validus* sp. n.
–	4^th^ dorsal stria nearly complete to base; sides of pronotum generally with numerous coarse punctures; apices of T9 bluntly triangular (Fig. 32A)	*Operclipygus variabilis* sp. n.

### Operclipygus dubius

(Lewis, 1888)

http://species-id.net/wiki/Operclipygus_dubius

**Map 10.**
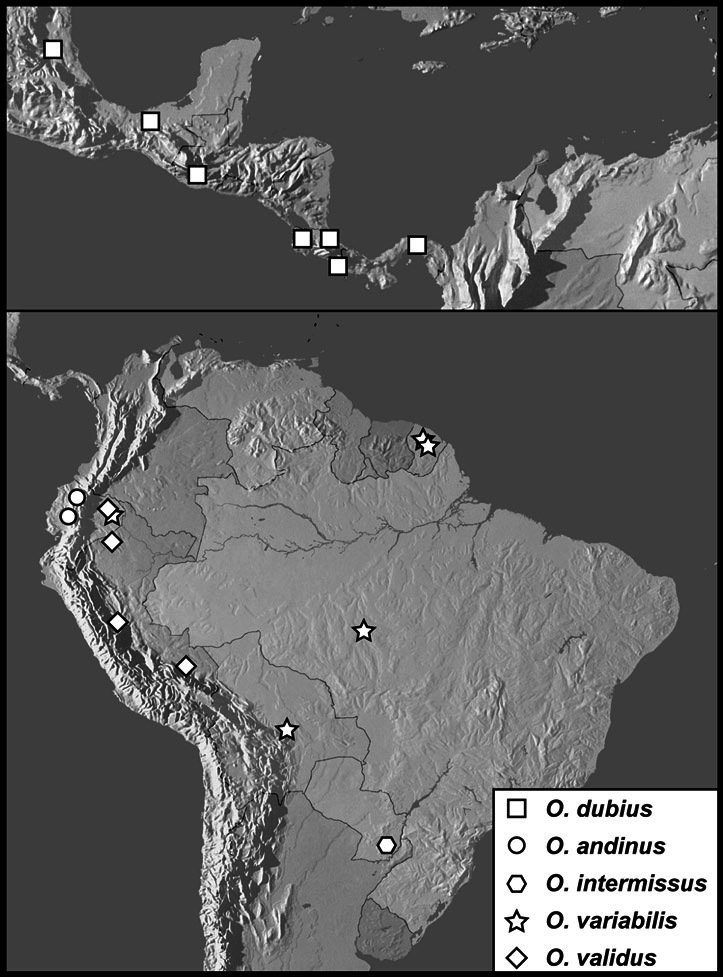
Records of the *Operclipygus dubius* group.

Figs 30A–E 31A–G Map 10

Epierus dubius Lewis, 1888: 208; *Pseudister dubius*: Bickhardt (1917: 165); *Operclipygus dubius*: Wenzel (1976: 258).

#### Type locality.

GUATEMALA: Guatemala: El Zapote Ranch [14°28'N, 91°54'W].

#### Type material.

**Lectotype**, here designated: “Zapote, Guatemala, G. Champion” / “*Epierus dubius* Lewis type” / “LECTOTYPE *Epierus dubius* Lewis, 1888 M.S. Caterino & A.K. Tishechkin des. 2010” (BMNH). This species was described from an unspecified number of specimens, and the lectotype designation fixes primary type status on the only known original specimen.

#### Other material.

**COSTA RICA: Guanacaste:** 1: Parque Nac. Guanacaste, Est. Pitilla [misspelled Patilla], 10°59'22"N, 85°25'33"W, 610m, 13–15.vii.2000, FIT, J. Ashe, R. Brooks, Z. Falin (SEMC); 1: Parque Nac. Guanacaste, Lado SO Vol. Cacao, 1000–1400m, 21–29.v.1992, F.A. Quesada, (INBIO); **Heredia:** 1: La Selva Biol. Stn., 3km S Puerto Viejo, 100m, 24.iii.1992, FIT, W. Bell (SEMC); 1: Finca Naranjo Valenciana 2km sur Pueblo Nuevo Sarapiqui, 90m, 24.vii–22.viii.1992, M. Ortiz (INBIO); **Puntarenas:** 1: Est. Biol. Las Cruces, Coto Brus, 8°47'N, 82°57'W, 1000m, 1–2.iv.2002, FIT, A. Cline & A. Tishechkin (LSAM), 2: 8–10.iv.2002, FIT, A. Cline & A. Tishechkin (LSAM, AKTC); 1: Est. Biol. Las Cruces, San Vito, 1200m, 1–30.vii.1982, FIT, B.D. Gill (MSCC); 2: Las Alturas Biol. Sta., 08°56.17'N, 82°50.01'W, 1660m, 31.v–3.vi.2004, FIT, J.S. Ashe, Z. Falin, I. Hinojosa (SEMC). **MEXICO: San Luis Potosí**: 1: 20km W. Xilitla, 1600m, 12.vi-6.viii.1983, FIT, cloud forest, S. & J. Peck (CMNC); **Tabasco**, 1: Teapa, i., H.H. S. (BMNH). 1: **PANAMA: Panamá**: Chepo-Carti Rd., 400m, vii.1982, FIT, B.D. Gill (BDGC).

#### Diagnostic description.

Length: 1.44–1.59 mm, width: 1.06–1.15 mm; body rufescent, nearly parallel sided and depressed; frons depressed and finely punctate, frontal stria absent across middle or represented by few rudimentary fragments, sides forming a continuous arch with complete supraorbital stria; epistoma weakly convex along apical margin; labrum 1.5× as wide as long; both mandibles with similar, subacute basal teeth; prescutellar impression present, elongate, almost linear, 2–3× length of scutellum; pronotum finely punctate throughout; without coarser punctures laterally; lateral submarginal pronotal stria absent; marginal stria interrupted behind head; anterior submarginal stria present, recurved obliquely posterad about one-third to one-half pronotal length; median pronotal glands about 6 puncture widths from anterior margin, alongside recurved anterior stria; elytra with one complete epipleural stria, with outer subhumeral stria fine, present in apical half, inner subhumeral stria absent, striae 1-4 complete to base, the apices of 1^st^ and 2^nd^ striae slightly abbreviated in some individuals, 5^th^ stria present in apical two-thirds, sutural stria present in apical three-fourths, widened anteriorly; prosternal keel produced and rather broadly rounded at base, carinal striae complete, connected anteriorly and basally, narrowly separated at middle; prosternal lobe subtruncate apically, with complete marginal stria; mesoventrite deeply emarginate; mesoventral marginal stria narrowly interrupted at middle; mesometaventral stria arched forward about halfway to mesoventral margin, sinuate, then angulate near mesocoxa, posteriorly nearly reaching middle of metacoxa; 1^st^ abdominal ventrite with 2 complete striae on each side; propygidial punctures small and sparse, major punctures separated by a little more than their diameters; pygidium with ground punctures not extremely dense, separated by a puncture width or more, with slightly coarser punctures intermingled, principally in basal third; marginal pygidial sulcus complete, ranging in appearance from a connected series of deep punctures to a deeply crenulate sulcus. Male genitalia (Figs 31A–G): as for group description above, distinguished by the broad truncate apices of S8 which bear a long series of setae, as well as by the subtriangular, hatchet-like apices of T9; S9 short, truncate at base, with broad shallow apical emargination, apical flanges widely separated; T10 poorly sclerotized; tegmen widest at base, narrowed in apical half.

#### Remarks.

The small size, lack of lateral submarginal pronotal stria, and long, oblique recurved anterior pronotal stria (Fig. 30B) will separate this species from any other *Operclipygus* known to occur in Central America.

### Operclipygus andinus sp. n.

urn:lsid:zoobank.org:act:0CF2FF21-D7F3-4A84-8F9C-96EF637311A8

http://species-id.net/wiki/Operclipygus_andinus

Figs 31H–K Map 10

#### Type locality.

ECUADOR: Pichincha: 7 km S Nanegalito [0°0.4'N, 78°40.6'W].

#### Type material.

**Holotype male**: “ECUADOR: Pichincha, Nanegalito, 7 km S on Nono Road, 1540m; 0°0'23"N,78°40'36"W 27–31 OCT 1999; Z. H. Falin, ECU1F99 070, ex: flight intercept trap” / “SM0353539 KUNHM-ENT” (SEMC). **Paratypes** (4): **ECUADOR: Los Ríos:** 1: CCRP [Centro Cientifico Rio Palenque], 21.xii.1980, S. Sandoval (CHSM); **Pichincha**: 1: Rio Palenque Sta., 47km S Santo Domingo, 250m, 5.v–25.vii.1985, malaise/FIT, S. & J. Peck (CMNC); 1: Maquipucuna Biological Station, River Trail, 0°7'34"N, 78°37'57"W, 1200m, 27–29.x.1999, FIT, Z. Falin (SEMC); 1: 16km E SantoDomingo, Tinalandia, 680m, 4.v–25.vii.1985, malaise/FIT, S. & J. Peck (CMNC).

#### Diagnostic description.

This species is extremely similar to *Operclipygus dubius*, differing mainly in the following characters: length: 1.50–1.62 mm, width: 1.15–1.22 mm; fragments of frontal stria more frequently evident; anterior submarginal pronotal stria barely recurved posterad, not reaching median pronotal gland openings which are about 8 puncture widths from anterior margin; prescutellar impression small, narrow, no longer than scutellum; lateral parts of pronotal disk with numerous very small punctures; prosternal striae more broadly separated, parallel in front; mesometaventral stria arched forward to middle of mesoventral disk, transverse in middle; propygidium sparsely covered with small deep punctures separated by about twice their diameters; pygidium with fine ground punctation very sparse, coarser punctures similarly spaced as on propygidium, sparser toward apex; pygidial marginal sulcus deep, crenulate (as in *Operclipygus dubius*). Male genitalia (Figs 31H–K) as for group description, with the apices of S8 obliquely subtruncate, with about 4 long apical setae; T9 with apices obliquely subquadrate; T10 not divided; S9 truncate at base, with deep narrow apical emargination; tegmen more or less parallel-sided, rounded to apex.

#### Remarks.

The very short recurved arms of the anterior submarginal pronotal stria and male genitalia will distinguish this species from *Operclipygus dubius*.

#### Etymology.

The name of this species refers to fact that all specimens are known from the Ecuadorian Andes.

### Operclipygus intermissus sp. n.

urn:lsid:zoobank.org:act:5E903190-4523-418A-93DD-EF2B418C85E0

http://species-id.net/wiki/Operclipygus_intermissus

Figs 31L–N Map 10

#### Type locality.

PARAGUAY: Itapúa: San Rafael Reserve [26°38.3'S, 55°39.8'W].

#### Type material.

**Holotype male**: “**PARAGUAY: Itapúa**, Yataí, San Rafael Reserve, 100m, 26°38'17"S,55°39'50"W, 26–30.IX.2000, Z.H.Falin, ex: flight intercept trap”/ “Caterino/Tishechkin Exosternini Voucher EXO-00277” (SEMC). **Paratypes** (1): same data as type (CMNC).

#### Diagnostic description.

This species is extremely similar to *Operclipygus dubius*, differing mainly in the following characters: length: 1.62–1.65 mm, width: 1.25–1.28 mm; frontal stria fragmented to absent at middle; anterior submarginal pronotal stria interrupted by median pronotal gland opening, continuing beyond it nearly to elytral midpoint; prescutellar impression narrow, not longer than scutellum; fragments of lateral submarginal pronotal stria may be present in anterior corners; pronotal disk with small, faint depressions alongside anterior submarginal stria; prosternal striae convergent to front, meeting in subacute arch; fine ground punctation of pygidium slightly denser than in *Operclipygus dubius*. Male genitalia (Figs 31L–N) as for group description, with the apices of S8 vertically oriented, curved to ventral apex, bearing 4 or 5 long apical setae, with distinct submarginal sclerotization along apical margins; T9 with apices obliquely subquadrate; T10 not divided; S9 strongly desclerotized along midline, weakly emarginate at base, apical emargination deep, narrow; tegmen more or less parallel sided, abruptly narrowed to apex.

#### Remarks.

The combination of an interrupted anterior submarginal pronotal striae (similar to that shown in Fig. 30F), lack of coarse lateral pronotal punctures, and prosternal striae that are joined in an acute anterior angle are the most distinctive external characters of *Operclipygus intermissus*. It is most similar externally to *Operclipygus variabilis*, which shares the interrupted anterior submarginal pronotal stria, but *Operclipygus variabilis* has the prosternal striae united in a distinct arch and has coarse lateral pronotal punctures.

#### Etymology.

This species’ name refers to the interruption of the anterior submarginal stria by the pronotal gland openings.

### Operclipygus variabilis sp. n.

urn:lsid:zoobank.org:act:4812A721-81FB-4B3D-A7D0-8DB7E52EB216

http://species-id.net/wiki/Operclipygus_variabilis

Figs 30F 32A–C Map 10

#### Type locality.

ECUADOR: Orellana: Yasuní National Park, Maxus Road at Piraña Bridge [0°39.5'S, 76°26'W].

#### Type material.

**Holotype male**: “**ECUADOR: Depto. Orellana** P.N. Yasuní, Via Maxus at Puente Piraña. 0°39.5'S, 76°26'W, Flight intercept FIT 1b-1. 14–20.vii.2008. A.K. Tishechkin. AT1052” / “Caterino/Tishechkin Exosternini Voucher EXO-02054” (FMNH). **Paratypes** (4): 1: Yasuní Res. Stn., mid.Rio Tiputini, 0°40.5'S, 76°24'W, 17–26.vii.1999, FIT, A.K. Tishechkin (LSAM), 1: 23–30.vi.1999, FIT, C.E. Carlton & A.K. Tishechkin (LSAM), 1: 4–17.vii.1999, FIT, C.E. Carlton & A.K. Tishechkin (AKTC); 1: Tiputini Biodiversity Station, 0.6376°S, 76.1499°W, 4–9.vi.2011, FIT, M.S. Caterino & A.K. Tishechkin, DNA Extract MSC-2192 (SBMNH).

#### Other material.

**BOLIVIA: Santa Cruz:** 1: 5km SSE Buena Vista, Flora y Fauna Hotel, 17°29.925'S, 63°39.128'W, 440m, 15–24.xii.2003, FIT, S. & J. Peck (AKTC). **BRAZIL: Mato Grosso:** 1: Cotriguaçu, Fazenda São Nicolau, 9°49.0'S, 58°16.6'W, 15–18.xii.2010, FIT, F.Z. Vaz-de-Mello & A.F. Oliveira (CEMT). **FRENCH GUIANA:** 1: Rés. Natur. de la Trinité, 4°40.11'N, 53°16.99'W, 7.ii.2011, Window trap, understory, SEAG (MNHN); 1: Rés. Natur. des Nouragues, Camp Inselberg, 4°05'N, 52°41'W, 30.ix.2010, Window trap, SEAG (CHND).

#### Diagnostic description.

This species is extremely similar to *Operclipygus andinus*, differing mainly in the following characters: length: 1.40–1.47 mm, width: 1.03–1.06 mm; frons always with central portion of frontal stria present, detached from sides; pronotal disk with fragments of lateral submarginal stria usually present in anterior corners; anterior submarginal stria recurved, interrupted at median pronotal gland openings, but continued posterolaterad beyond to near pronotal midpoint; pronotal disk finely punctulate throughout, with few coarser lateral punctures posterolaterad apices of recurved anterior submarginal stria; prosternal striae more or less complete, nearly reaching presternal suture, subparallel in anterior half, joined in anterior arch; propygidial punctures moderate in size, round, denser in middle two-thirds of disk than at sides; pygidium with ground punctation fine, dense, though rather shallow; with coarser punctures concentrated along the anterior margin, with few additional coarse punctures mostly restricted to basal third of disk; marginal pygidial sulcus appearing as a connected series of deep, coarse punctures. Male genitalia (Figs 32A–C) as for group description, with the apices of S8 narrowed, but broadly subtruncate, bearing 4 or 5 long apical setae, with linear submarginal sclerotization along upper, apical margins; T9 with apices narrowed, bluntly triangular; T10 not divided; S9 narrow, with base only slightly widened, weakly rounded, with shallow apical emargination; tegmen with sides widening strongly toward apex, narrowed to broadly rounded apex.

#### Remarks.

The aedeagus of this species is somewhat similar to that of *Operclipygus validus*, widening toward the apex (compare figs 32C and 32D). However, several other genitalic characters distinguish *Operclipygus variabilis*, including the narrower apices of T9 (Fig. 32A), the truncate apices of S8, and the basally narrower S9 (Fig. 32B). Due to variability in external characters, which may indicate significant geographic differentiation, we restrict the type series to those specimens from eastern Ecuador.

#### Etymology.

This species’ name refers to its notable variability in a few characters, especially the lateral pronotal punctation and length of the 4^th^ dorsal elytral stria.

### Operclipygus validus sp. n.

urn:lsid:zoobank.org:act:B184C5B8-2EEB-4D44-8B07-97100689F939

http://species-id.net/wiki/Operclipygus_validus

Figs 32D–G Map 10

#### Type locality.

PERU: Ucayali: km 205 of Tingo Maria-Pucallpa Road [9°8.2'S, 75°47.3'W].

#### Type material.

**Holotype male**: “PERU: Ucayali Dept. Tingo Maria-Pucallpa Rd. Puente Chino, km 205, 1300m, 9°8'12"S,75°47'20"W, 11–14 OCT 1999; R.Brooks, PERU1B99 007A, ex: flight intercept trap”/ “SM0143737 KUNHM-ENT” (SEMC). **Paratypes** (4): **PERU: Madre de Dios:** 2: Manu National Park, Pantiacolla Lodge, Alto Madre de Dios R., 12°39.3'S, 71°13.9'W, 420m, 14–19.xi.2007, FIT, D. Brzoska (SEMC, FMNH); 1: Reserva Cuzco Amazonico, 15km NE Pto. Maldonado, 12°33'S, 69°03'W, 200m, 22.vi.1989, FIT, J. Ashe & R. Leschen (SEMC), 1: 28.vi.1989, FIT, J. Ashe & R. Leschen (SEMC).

#### Other material.

**ECUADOR, Sucumbíos:** 1: Sacha Lodge, 0.5°S, 76.5°W, 270m, 14–24.v.1994, malaise trap, Hibbs (SEMC); **PERU: Loreto:** 1: 1.5km N Teniente Lopez, 2°35.66'S, 76°06.92'W, 210–240m, 20.vii.1993, FIT, R. Leschen (SEMC).

#### Diagnostic description.

This species is extremely similar to *Operclipygus intermissus* and *Operclipygus variabilis*, differing mainly in the following characters: length: 1.53–1.62 mm, width: 1.28–1.30 mm; frons always with central portion of frontal stria present, detached from sides; pronotal disk with fragments of lateral submarginal stria usually present in anterior corners; anterior stria recurved, usually complete, though rarely interrupted at median pronotal gland opening (as seen in holotype); elytra with 4^th^ stria abbreviated basally, present in anterior one-half to two-thirds; pygidium rather wide and short, with the marginal sulcus comprising large, deep punctures, ground punctation as in *Operclipygus variabilis*. Male genitalia (Figs 32D–G) as for group description, with the apices of S8 vertically oriented, curved to ventral apex, bearing 4 or 5 long apical setae, with very short submarginal sclerotization along upper, apical margins; T9 with apices obliquely subquadrate, widened to inner apical edges; T10 not divided; S9 narrow, with base only slightly widened, truncate, with shallow apical emargination; tegmen with sides straight, expanded toward broadly rounded apex.

#### Remarks.

This species is most confidently distinguished by its aedeagus, having a tegmen that broadens toward the apex (Fig. 32D), and which has secondary longitudinal keels on either side of the medioventral keel. Externally this species is generally separable from those others of the *dubius* group that lack most of the lateral submarginal pronotal stria by having a distinctly abbreviated 4^th^ elytral stria. One specimen from Loreto, Peru exhibits a generally similar aedeagus, which is, however, even more extremely expanded toward apex. It has a more nearly complete 4^th^ dorsal stria, and may constitute a distinct species. Unfortunately this specimen lacks a head and prothorax, and is tentatively considered to represent a variant of this species until additional material becomes available for study.

#### Etymology.

This species’ name means ‘strong’ or ‘active’, consistent with all specimens being caught in flight traps.

### Operclipygus lunulus sp. n.

urn:lsid:zoobank.org:act:12672154-F782-48B2-B02C-8288FB0B9417

http://species-id.net/wiki/Operclipygus_lunulus

**Map 11.**
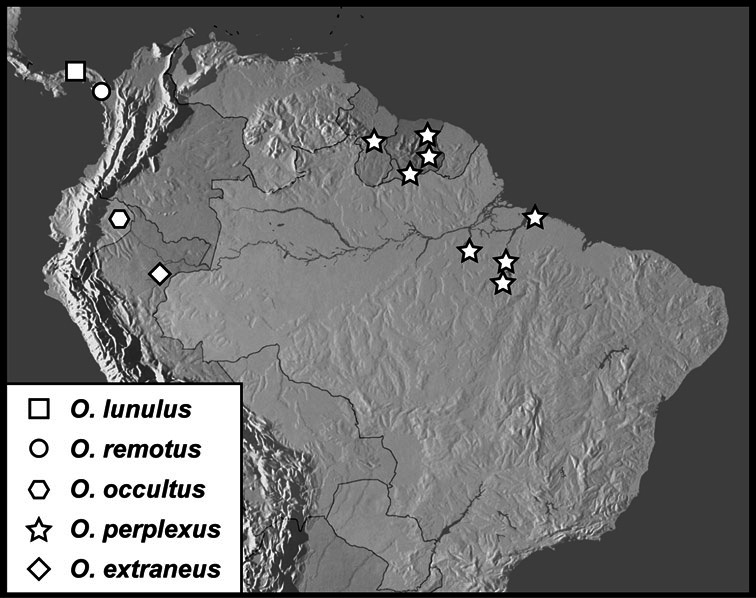
Records of the *Operclipygus dubius* group.

Figs 30G 32H–K Map 11

#### Type locality.

PANAMA: Colón: San Lorenzo National Park, Achiote [9°12'N, 79°59'W].

#### Type material.

**Holotype male**: “**PANAMA**: Prov. Colón, Achiote – P. N. San Lorenzo N09°12', W79°59' Cafetal B Dist. 50m Tr. Intercepción, A. Mercado 8–22.V.2007” / “*Operclipygus* sp. # 12 San Lorenzo Inventory A.K. Tishechkin det. 2010” / “Caterino/Tishechkin Exosternini Voucher EXO-00282” (FMNH). **Paratype** (1): same data as type (GBFM).

#### Diagnostic description.

This species is extremely similar to *Operclipygus dubius*, differing mainly in the following characters: length: 1.59–1.62 mm, width: 1.22–1.28 mm; central portion of frontal stria present, detached from sides; supraorbital and frontal striae united in a broad, continuous arch; recurved arms of anterior submarginal stria ending near median pronotal gland openings, about 8 puncture widths from anterior margin; short fragment of lateral submarginal pronotal stria present in anterior corner; prescutellar impression slightly shorter than scutellum; prosternal striae divergent posteriorly, not connected at base; mesometaventral stria arching narrowly forward at middle; propygidium with moderate, round, shallow punctures separated by about their widths or slightly less basally, less dense posterad; pygidium with coarser punctures sparsely scattered in basal third of disk; pygidial sulcus complete, deep, strongly crenulate along outer edge. Male genitalia (Figs 32H–K) as for group description, with the apices of S8 bluntly subtruncate, dorsal edge oblique to apex, bearing about 5 long apical setae, with distinct submarginal sclerotization along upper margins; T9 with apices obliquely subquadrate, expanded to inner edges; T10 not divided; S9 desclerotized along midline, strongly expanded to base, shallowly but acutely emarginate at apex; tegmen more or less parallel-sided, rounded to apex, very similar to that of *Operclipygus andinus*, but distinctly more elongate.

#### Remarks.

The short recurved arms of the anterior submarginal stria (Fig. 30G) combined with the presence of a frontal stria and strongly reduced lateral submarginal pronotal striae distinguish this species from several close relatives.

#### Etymology.

The name of this species refers to the continuous, crescent-like arch formed by the frontal and supraorbital striae.

### Operclipygus remotus sp. n.

urn:lsid:zoobank.org:act:0AF82D92-CAB5-4263-BA19-43AD7E6E3DED

http://species-id.net/wiki/Operclipygus_remotus

Fig. 33A Map 11

#### Type locality.

PANAMA: Darién: Cana, Pirre Camp [7°45.825'N, 77°43.325'W].

#### Type material.

**Holotype male**: “**PANAMA**: Darién Prov. Cana, Pirre Camp, 1320, 7°45.825'N, 77°43.325'W, 6-MAY-2008, A. R. Gillogly”/ “Caterino/Tishechkin Exosternini Voucher EXO-00281”(FMNH).

#### Diagnostic description.

This species is extremely similar to *Operclipygus lunulus*, differing mainly in the following characters: length: 1.65 mm, width: 1.34 mm; anterior submarginal pronotal stria fine, recurved obliquely to about middle of pronotum; lateral region of pronotal disk with few coarse punctures; mesometaventral stria more broadly arched forward on mesoventral disk; propygidial punctures small, round, sparse, separated by at least 2× their diameters throughout; pygidium with coarse punctures restricted to narrow basal marginal band. Male genitalia very similar to those of *Operclipygus lunulus* (see Figs 32I–K), the aedeagus indistinguishable; S9 only weakly widened toward base (Fig. 33A).

#### Remarks.

This species can only be recognized by the combination of characters given above.

#### Etymology.

This species’ name refers to the remoteness of its type locality.

### Operclipygus occultus sp. n.

urn:lsid:zoobank.org:act:98A28E0E-C6B2-4406-9DC2-34D549763C8F

http://species-id.net/wiki/Operclipygus_occultus

Figs 34B, D–F Map 11

#### Type locality.

ECUADOR: Orellana: Tiputini Biodiversity Station [0°38.2'S, 76°8.9'W].

#### Type material.

**Holotype male**: “**ECUADOR: Orellana**, Tiputini Biodiversity Station 0°38.2'S, 76°8.9'W. Flight intercept FIT7-1. 27–31 July 2008. A.K.Tishechkin” / “Caterino/Tishechkin Exosternini Voucher EXO-00283” (FMNH).

#### Diagnostic description.

This species is very similar to *Operclipygus dubius*, differing mainly in the following characters: length: 1.59 mm, width: 1.12 mm; frons weakly depressed, frontal stria complete; pronotal disk with prescutellar impression short and very narrow, few coarse punctures posterolaterad ends of anterior stria; lateral submarginal pronotal stria fine, present in anterior corners only; anterior submarginal pronotal stria fine, recurved posterolaterally to about one-third from anterior pronotal margin; elytra with dorsal stria 1 obsolete in apical third, striae 2-3 complete, 4^th^ stria present in only apical half; prosternal keel with carinal striae subparallel in apical three-fourths, only weakly divergent basally; marginal mesoventral stria complete; metaventrite with shallow longitudinal depression; propygidium with dense, small, rather deep punctures; pygidium with fine dense ground punctation, coarser punctures dense along basal margin, sparsely scattered in apical two-thirds; marginal pygidial sulcus deep, coarsely crenulate. Male genitalia (Figs 33B, D–F) as for group description, the apices of S8 strongly narrowed, with only a couple inconspicuous inner apical setae, curved to ventral apex, bearing 4 or 5 long apical setae, with distinct submarginal sclerotization along apical margins; T9 with apices elongate subtriangular, inner edges parallel; T10 not divided; S9 not desclerotized along midline, truncate at base, with shallow, equilateral apical emargination; tegmen widening to near apex, narrowed to truncate apex; median lobe narrowed proximad gonopore.

#### Remarks.

This species can only confidently be recognized by the male genitalia (Figs 33B, D–F). The elongate apical arms of T9 and the narrow, subacute apices of S9 are both highly distinctive in the group. A few other characters, such as the median longitudinal depression of the metaventrite and the complete frontal stria may be helpful, but with only a single available specimen it is impossible to ascertain their significance.

#### Etymology.

This species’ name refers to the fact that hidden characters must be observed for identification.

### Operclipygus perplexus sp. n.

urn:lsid:zoobank.org:act:BA9FBA28-C788-46F3-9591-C5308438E63A

http://species-id.net/wiki/Operclipygus_perplexus

Figs 33G–I Map 11

#### Type locality.

BRAZIL: Pará: Tucuruí [3°45'S, 49°40'W].

#### Type material.

**Holotype male**: “**BRASIL: Pará:** Tucuruí, 3°45'S, 49°40'W. Piège d’interception. iv.1986”/ “Caterino/Tishechkin Exosternini Voucher EXO-00595” (UFPR). **Paratypes** (8): **BRAZIL: Pará:** 4: Altamira - Marabá: km 18, 3°09'S, 52°03'W, v.1984, FIT (CHND, FMNH, MSCC, AKTC); 1: IPEAN, Belém, Utinga, 1°27'S, 48°26'W, viii.1985, FIT (CHND), x.1985, FIT (CHND); 1: Barcarena, 1°30'S, 48°37'W, 13–25.vi.1991, FIT (CHND); 1: Carajás, Serra Norte, 6°04'S, 50°12'W, 13.xi–2.xii.1987, FIT (CHND)

#### Other material.

**GUYANA:** 1: Kurupukari, 4°40'N, 58°40'W, ix-xi.1992, FIT/Malaise (BMNH). **SURINAME: Brokopondo:** 1: Brownsberg Nature Preserve, Witi Creek Trail, 4°56'55"N, 55°10'53"W, 340m, 23.vi.1999, 25.vi.1999, FIT, Z. Falin, A. Gangadin, H. Hiwat (SEMC); **Marowijne**: 1: Palumeu, 3°20'56"N, 55°26'18"W, 160m, 9.vii.1999, FIT, Z. Falin (CMNC); **Sipaliwini**: 1: CI-RAP Surv. Camp 2: Sipaliwini River, 2°10.521'N, 56°47.244'W, 210m, 27.viii.2010, 1.ix.2010, FIT, T. Larsen & A.E.Z. Short (SEMC).

#### Diagnostic description.

Length: 1.15–1.34 mm, width: 0.84–1.03 mm; body elongate oval, widest behind humeri; frons and upper two-thirds of epistoma weakly depressed, frontal stria complete; labrum about twice as wide as long, straight across apical margin; both mandibles with subacute marginal teeth; prescutellar impression about half as broad, slightly longer than scutellum; pronotal disk with fine sparse ground punctation only, lacking coarser lateral punctures; lateral submarginal pronotal stria complete; anterior submarginal pronotal stria recurved obliquely posterad slightly more than half length of pronotum; elytra with one complete epipleural stria, outer subhumeral stria present in apical half, inner subhumeral stria absent, striae 1-4 complete, 5^th^ stria present in half to two-thirds, sutural stria present in apical three-fourths; prosternal keel projecting at base, carinal striae complete, narrowed between coxae, slightly divergent posterad and anterad, connected by anterior arch; mesoventrite deeply, narrowly emarginate at front, marginal stria interrupted; mesometaventral stria arched forward just behind emargination, sinuate at sides, extended posterolaterally toward outer third of metacoxa; 1^st^ abdominal ventrite with two complete lateral striae; propygidium with narrow impunctate band along base, with small, round, rather deep discal punctures in posterior two-thirds; pygidium with fine dense ground punctation, coarser punctures densest along basal margin, sparsely scattered in apical two-thirds; marginal pygidial sulcus deep, coarsely crenulate. Male genitalia (Figs 33G–I) as for group description, apices of S8 narrowed, subacute, with few long apical setae, with distinct submarginal sclerotization along upper, apical margins; T9 with apices acute at apical and inner corners, halves broadly fused dorsally; T10 very weakly sclerotized, inconspicuous; S9 desclerotized along midline, narrow, parallel-sided to truncate base, apical emargination very shallow; tegmen widest near base, gradually narrowed to apex, medioventral process forming prominent ventral keel.

#### Remarks.

This species is unusual among species in the *dubius* group, having a complete frontal stria and complete lateral submarginal pronotal striae. Once dissected and confirmed to belong to the *Operclipygus dubius* group, these characters alone will separate it from all others. The type series is restricted to specimens from northeastern Brazil.

#### Etymology.

This species’ name refers to the difficulty of recognizing its true relationships.

### Operclipygus extraneus sp. n.

urn:lsid:zoobank.org:act:B2305025-50D8-4042-B03C-EFEAF9163106

http://species-id.net/wiki/Operclipygus_extraneus

Fig. 30H Map 11

#### Type locality.

PERU: Loreto: Iquitos [3°45'S, 73°15'W]

#### Type material.

**Holotype female**: “PERU: Loreto Prov., Iquitos, 90m, 5 May 1992, J. Danoff-Berg, ex:flight intercept trap” / “♀”/ “SEMC0903628” (SEMC).

#### Diagnostic description.

Length: 1.40 mm, width: 1.12 mm; body rufobrunneus, broad, subquadrate, subdepressed, with relatively conspicuous ground punctation, particularly on pronotal disk; frons depressed at middle; frontal stria rounded at sides, absent across front; labrum short, apex truncate; left mandible with subacute tooth, right mandible not visible in type; pronotal sides sinuate (appearing weakly pinched) at base; pronotal disk with elongate but faint prescutellar impression, almost 3× scutellar length; ground punctation sparse but well impressed, with numerous coarser lateral punctures; lateral submarginal pronotal stria complete; anterior marginal stria complete behind head; anterior submarginal stria also present behind anterior margin, obliquely recurved posterad about half pronotal length; elytra with one complete epipleural stria, outer subhumeral stria present in apical half, inner subhumeral stria absent, striae 1-4 complete, 5^th^ stria present in apical half, sutural stria nearly complete, barely abbreviated at base; prosternal keel projecting basally, short carinal striae united in narrow arch just anterad midpoint, joined at base; mesoventrite emarginate anteriorly, marginal stria complete; mesometaventral stria broadly arched to near mesoventral marginal stria, extending posterolaterally to near outer third of metacoxa; 1^st^ abdominal ventrite with two complete lateral striae which converge both anteriorly and posteriorly; propygidium with moderately large, very shallow punctures separated by slightly less than their diameters, sparser and smaller in apical half; pygidium with fine dense ground punctation, coarser punctures very inconspicuous, few evident near basal margin; marginal pygidial sulcus complete, very fine, even and shallow. Male not known.

#### Remarks.

This species is only known from a single female, but is quite distinct regardless. This species’ broad, depressed body form (Fig. 30H), along with the shape of the sides of the pronotum, subparallel with a basal sinuation, are nearly adequate to recognize it. The lack of frontal stria and fine marginal pygidial sulcus in addition to these will diagnose it unambiguously. A few external characters, particularly the depressed frons lacking a frontal stria, and the dentate left mandible, suggest placement in the *Operclipygus dubius* group. However, this will need reassessment if a male specimen is discovered.

#### Etymology.

This species’ name refers to its uncertain relationships.

### *Operclipygus hospes* group

As discussed above under the *Operclipygus dubius* group introduction, the *Operclipygus hospes* and *Operclipygus dubius* groups are highly similar externally. Both are small, generally elongate, somewhat parallel-sided, and subdepressed. Externally the species of the *Operclipygus hospes* group are harder to characterize, but they nearly all have 2 complete striae on the 1^st^ abdominal ventrite (Fig. 34A), have the antennal club distinctly elongate, rarely have both mandibles strongly toothed, and usually have the posterolateral end of the lateral metaventral stria extending toward the outer corner of the metacoxa, often with the extreme apex bent laterally.

### Key to the species of the *Operclipygus hospes* group

**Table:** 

1	Outer subhumeral stria complete	2
–	Outer subhumeral stria abbreviated, present only in posterior half	6
2	1^st^ abdominal ventrite with single lateral stria; propygidium with larger, sparse punctures, their diameters equal to about one-fifth of propygidial length; lateral submarginal pronotal stria distant from margin; prosternal carinal striae widely separated, weakly converging, almost parallel; Costa Rica	*Operclipygus limonensis* sp. n. [not belonging to the *Operclipygus hospes* group, but also keyed here as it is very similar]
–	1^st^ abdominal ventrite with two lateral striae; propygidium with much smaller punctures, their diameters less than one-eighth of propygidial length	3
3	Species from Central America	4
–	Species from South America	5
4	Outer lateral stria of 1^st^ abdominal ventrite complete, reaching posterior margin; pronotum with at least a few coarse lateral punctures; lateral metaventral stria extending toward outer corner of metacoxa; pygidial punctures larger, shallow, ocellate (Fig. 39F)	*Operclipygus fusistrius* sp. n.
–	Outer lateral stria of 1^st^ abdominal ventrite abbreviated, sinuate near midpoint of ventrite length around postcoxal fovea; pronotum lacking coarse lateral punctures; metaventral stria extending obliquely toward metepisternum; pygidial punctures small, deep (Fig. 41C)	*Operclipygus ibiscus* sp. n.
5	Propygidial punctures small, deep, and dense, separated by less than one-half puncture diameter (Fig. 39B); marginal pygidial sulcus relatively fine; apices of 4^th^ and 5^th^ elytral striae free (Fig. 39A); W Amazonia	*Operclipygus diffluens* sp. n.
–	Propygidial punctures sparser (Fig. 39D); marginal pygidial sulcus deep and coarse; apices of 4^th^ and 5^th^ dorsal striae confluent (Figs 39C–D); SE Andean slopes	*Operclipygus confluens* sp. n.
6	Marginal sulcus of pygidium absent	7
–	Marginal sulcus of pygidium present	8
7	Sternite 8 of male genitalia with apices narrow, parallel, only weakly curved downward (Fig. 43B); aedeagus narrow (Fig. 43I); Central America	*Operclipygus gratus* sp. n.
–	Sternite 8 of the male genitalia with apices broader, divergent, strongly downturned (Fig. 43F); aedeagus wider (Fig. 43K); W Amazonia	*Operclipygus rileyi* sp. n.
8	Pygidial sulcus normally developed, well-incised (may be inconspicuous in specimens with pygidium retracted), rarely abbreviated basally	10
–	Pygidial sulcus very thin, striiform, complete or not	9
9	Lateral submarginal pronotal stria complete, well impressed; numerous coarse lateral pronotal punctures present; sutural stria obsolete in anterior one-fourth	*Operclipygus* sp. [a probably distinct entity from French Guiana, represented only by females, which we have chosen not to describe at this time]
–	Lateral submarginal pronotal stria represented by short, thin fragments near anterior corners; sutural stria complete or only very slightly abbreviated at base (Fig. 37A)	*Operclipygus inquilinus* sp. n.
10	Pronotum without lateral submarginal stria, with at most minute anterior fragments	11
–	Pronotum with lateral stria present, at least as short anterior stria	12
11	Recurved arms of anterior submarginal pronotal stria nearly reaching pronotal midpoint (Fig. 45A); mesometaventral stria forming wide trapezoid on mesoventrite; ground punctation of body relatively faint; prosternal carinal striae united posteriorly	*Operclipygus impositus* sp. n.
–	Recurved arms of anterior submarginal pronotal stria shorter, not reaching beyond anterior third of pronotum (Fig. 35G); mesometaventral stria forming narrower arch; ground punctation of body conspicuous; prosternal carinal striae free posteriorly	*Operclipygus minutus* sp. n.
12	Lateral submarginal pronotal stria present as a short fragment in distinctly less than anterior half	13
–	Lateral pronotal stria longer, complete or posteriorly abbreviated behind midpoint	18
13	Dorsal elytral stria 4 complete, almost reaching anterior margin of elytron	14
–	Dorsal elytral stria 4 not extending anterad middle third of elytron	*Operclipygus colombicus* sp. n.
14	Frontal stria finely carinate, complete across middle, dividing separately concave epistoma and frons; upper surface of antennal club with round, basolateral sensory depression (Fig. 4I); lateral metaventral stria bent laterad at apex toward metepisternum	*Operclipygus confertus* sp. n.
–	Frontal stria not carinate, depression of frons and epistoma confluent; other characters varied15
15	Body very small, <1.5 mm; ground punctation of pronotum and elytra fine but relatively conspicuous (Fig. 35C); pygidium short, basal width distinctly greater than midline length (Fig. 35D)	*Operclipygus prominens* sp. n.
–	Body larger, >2 mm; body smoother, ground punctation of pronotum and elytra relatively inconspicuous; pygidium longer, midline length greater than basal width	16
16	Antennal bossae prominent, frontal stria slightly displaced dorsad, nearly straight across front (Fig. 35A); epistoma broadly concave; prosternal carinal striae free posteriorly; pygidium with numerous, conspicuous larger punctures over dense ground punctation (Fig. 35B)	*Operclipygus callifrons* sp. n.
–	Antennal bossae not prominent, frontal stria sinuate at sides; epistoma very shallowly concave; prosternal carinal striae united posteriorly; pygidium with secondary punctures finer	17
17	Secondary pygidial punctures fine (Fig. 37D); T8 of male genitalia not strongly sclerotized, with deep, narrow basal emargination (Fig. 38E); W Amazonia	*Operclipygus communis* sp. n.
–	Secondary pygidial punctures slightly coarser (Fig. 37F); T8 of male genitalia strongly sclerotized, with shallower basal emargination (Fig. 38F); E Amazonia…	*Operclipygus belemensis* sp. n.
18	Pronotum with few or no coarse lateral punctures (Fig. 35E)	*Operclipygus tiputinus* sp. n.
–	Pronotum with numerous coarse lateral punctures	19
19	Recurved ends of median portion of anterior pronotal stria short, restricted to anterior fourth of pronotal length	20
–	Recurved ends of median portion of anterior pronotal stria long, reaching middle third of pronotal length	28
20	Pronotal prescutellar impression long, flame-shaped (Figs 34C, E)	21
–	Pronotal prescutellar impression represented by thin, striiform impression, approximately as long as scutellum	24
21	Prosternal carinal striae extending forward nearly to presternal suture, prosternal keel not narrowed anterad; Central America	22
–	Prosternal carinal striae united well posterad presternal suture, keel distinctly narrowed anterad (Fig. 42D); Amazonia	23
22	Pronotum with <20 coarse lateral punctures (Fig. 42H); propygidial punctures small, ocellate, separated by nearly their diameters	*Operclipygus praecinctus* sp. n.
–	Pronotum with entire lateral third of disk coarsely punctate (Fig. 42F); propygidial punctures denser, separated by about half their diameters	*Operclipygus assimilis* sp. n.
23	Lateral pronotal punctures numerous (>20) and coarse (Fig. 34E); body larger (~2.5 mm); only known from Bolivia	*Operclipygus ignifer* sp. n.
–	Lateral pronotal punctures fewer (<20) and rather shallow (Fig. 34C); body smaller (~2 mm); distribution poorly documented, probably only northeastern Amazonia	*Operclipygus hospes* (Lewis)
24	Lateral submarginal pronotal stria complete	25
–	Lateral submarginal pronotal stria slightly abbreviated posteriorly; frontal stria weakly carinate, dividing depressed epistoma and frons	*Operclipygus confertus* sp. n.
25	Posterior ends of prosternal carinal striae united; anterior half of epistoma convex	26
–	Posterior ends of prosternal carinal striae free; epistoma longitudinally concave throughout	27
26	Fourth dorsal stria complete (Fig. 45C)	*Operclipygus curtistrius* sp. n.
–	Fourth dorsal stria abbreviated (Fig. 41D)	*Operclipygus novateutoniae* sp. n.
27	Central portion of frontal stria detached from sides (Fig. 34G)	*Operclipygus subterraneus* sp. n.
–	Frontal stria complete, not interrupted at sides	*Operclipygus tenuis* sp. n.
28	Posterior ends of prosternal carinal striae united; secondary punctures on pygidium tiny and sparse (Fig. 37G)	*Operclipygus innocuus* sp. n.
–	Posterior ends of prosternal carinal free; secondary punctures on pygidium larger and dense	*Operclipygus incisus* sp. n.

### Operclipygus hospes

(Lewis, 1902)

http://species-id.net/wiki/Operclipygus_hospes

**Map 12.**
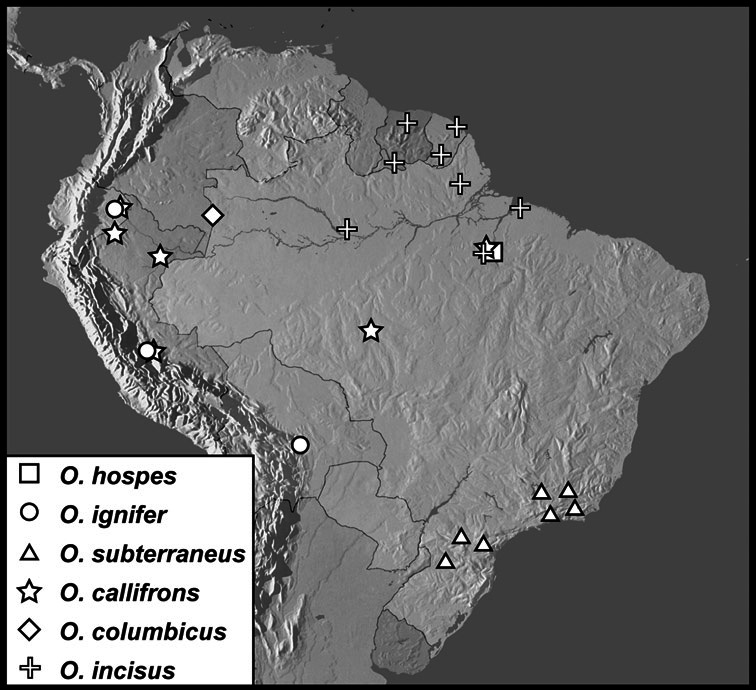
Records of the *Operclipygus hospes* group.

Figs 34B–D 36A–E Map 12

Phelister hospes Lewis, 1902: 236; *Pseudister hospes*: Hinton (1935e: 15); *Operclipygus hospes*: Wenzel (1976: 258).

#### Type locality.

Unknown, the original type locality in New York State, USA is wrong (see below).

#### Type material.

**Lectotype male**, here designated: “Ulster Co., N.Y.” [in error; Wenzel, 1976] / “LECTOTYPE *Phelister hospes* Lewis M.S.Caterino & A.K.Tishechkin des. 2010” (BMNH). This species was described from an unspecified number of specimens, and the lectotype designation fixes primary type status on the only known original specimen.

#### Other material.

**BRAZIL: Pará:** 1:Tucuruí, 3°45'S, 49°40'W, 9–17.xii.1985, FIT (CHND).

#### Diagnostic description.

Length: 1.75–1.78 mm, width: 1.37–1.40 mm; body rufobrunneus, elongate oval, sides subparallel at middle; frons and epistoma slightly depressed at middle, epistoma slightly elevated relative to frons; sides of frontal stria rounded, central portion complete, slightly outwardly arcuate at middle; supraorbital stria more or less complete, narrowly separated from sides of frontal; antennal club with upper basolateral sensory pit; pronotal disk with deeply impressed, elongate prescutellar impression, nearly twice as long as scutellum, acuminate anterad; pronotal disk with ~16 moderately coarse punctures posterolaterad recurved end of anterior submarginal stria, not extending to margin; lateral submarginal stria slightly abbreviated posterad, may be interrupted at middle; anterior submarginal stria crenulate, recurved straight posterad for ~1/6 pronotal length; median pronotal gland openings present just beyond apices of recurved anterior stria, nearly one-fourth pronotal length from anterior margin; elytra with outer subhumeral stria present in apical half, inner subhumeral stria absent, striae 1-3 complete, 4^th^ stria barely abbreviated at base, 5^th^ stria present in apical half; sutural stria present in apical three-fourths; prosternal keel weakly produced posteriorly, carinal striae short, close, weakly convergent to front, united posteriorly and anteriorly, by narrow arch about one-third keel length from presternal suture, keel distinctly narrowed anterad arch; mesoventrite emarginate, marginal stria complete; mesometaventral stria shallowly subangulate forward onto mesoventral disk, continued posterolaterad by lateral metaventral stria, extending obliquely toward outer corner of metacoxa, bent outward at apex; 1^st^ abdominal ventrite with two complete, subparallel lateral striae, inner tending to continue mediad along basal margin; propygidium with moderately large, shallow punctures separated by about half their diameters; pygidium with dense ground punctation, coarser punctures irregularly interspersed, slightly denser toward basal margin; marginal pygidial sulcus complete, deep but rather fine, inner edge crenulate, outer edge smooth. Male genitalia (Figs 36A–E): accessory sclerites present; T8 elongate, sides subparallel in middle half, basal apodemes slightly divergent, apices roundly narrowed, apical emargination deep, acute, basal emargination deep, broad, basal membrane attachment line distad emargination by about one-sixth its depth, ventrolateral apodemes most strongly developed before middle, narrowly separated at midline; S8 with sides broadly rounded in basal three-fourths, apical guides developed only in apical fourth, very narrow, rounded at apices, halves approximate at base, diverging to apex; T9 widest one-third from base, with subacute angle at this point, weakly narrowed to base, sinuately narrowed to apex, apices subtruncate, rather broad; T10 with halves separate along midline; S9 broad, narrowed at middle, markedly desclerotized along midline, base broadly rounded, lateral flanges wide, apex with narrow median emargination, apical flanges well developed, separate; tegmen widest about one-third from apex, weakly narrowed to base, rounded to subacute apex, apex rather strongly curved downward, with weak subapical cleft visible in lateral view, medioventral process well developed, long, its apex distinctly truncate, strongly projecting beneath about one-third from base; basal piece about one-fourth tegmen length; median lobe about two-thirds tegmen length, nearly half of this length made up of very long filamentous portions of proximal apodemes.

#### Remarks.

The only named species of the *Operclipygus hospes* group is also one of its most difficult to characterize. The type does not correspond perfectly to any other material that we have studied, and it is conceivable that it is a somewhat aberrant individual, noting that the lateral submarginal pronotal striae are not exactly bilaterally consistent (interrupted on one side, complete on the other). The true type locality of this species is unknown. ‘Ulster Co., N.Y.’ is clearly an error. Wenzel (1976) considered Santarém, Pará, Brazil a good possibility, for reasons unknown to us. This uncertainty in type locality is extremely unfortunate, as that would provide some clue as to whether its distinctness might result from being an outlying locality relative to most other material examined. The closest match we have is indeed a specimen from Pará (Tucuruí), but it is not identical.

*Operclipygus hospes* is very closely related to the following species, and they could almost be lumped in a single polymorphic entity, but the following exhibits a number of distinctive character states (detailed below), and is significantly larger, so we choose to recognize it separately. These two species are also enigmatic in the larger scheme of the *hospes* group. They both have a distinct basolateral sensory pit on the antennal club (Fig. 4I), which is otherwise restricted to species near *Operclipygus gratus* (below), but have genitalia with a shorter, apically truncate medioventral aedeagal process. The subapical cleft of the aedeagus that is found in *Operclipygus ignifer* and others is, however, very poorly developed in *Operclipygus hospes* (Fig. 36E).

The citation of *Operclipygus hospes* by Corréa et al. (2012) as a species of potential forensic importance in southern Brazil refers instead to the closely related *Operclipygus subterraneus* sp. n. described below.

In this species and the 13 that follow (through *Operclipygus innocuus*) all have the medioventral process of the aedeagus narrowed but distinctly truncate at the tip, rather than acute (e.g., Figs 36E–M), an apparently good synapomorphy. Generally in these the tegmen itself has a weak cleft, or dentate process near the apex. Many have the 8^th^ sternite broadly rounded, widened basally (Fig. 36B). However, while this helps to place many species in the larger *Operclipygus hospes* group, it is not exclusive to these species. In the last three species here (*Operclipygus communis*, *Operclipygus belemensis* and *Operclipygus innoccus*) the accessory sclerites are very small, the 8^th^ tergite exhibits distinctive apicodorsal sclerotizations (Figs 38E–F), and the 9^th^ sternite is strongly sclerotized.

### Operclipygus ignifer sp. n.

urn:lsid:zoobank.org:act:FF425190-B7FE-40E8-9DF0-98C9B887184B

http://species-id.net/wiki/Operclipygus_ignifer

Figs 34E 36 Map 12

#### Type locality.

BOLIVIA: Santa Cruz: 5 km SSE Buena Vista, Flora y Fauna Hotel [17°29.925'S, 63°39.128'W].

#### Type material.

**Holotype male**: “**BOLIVIA: Dpto. Santa Cruz.** 5km SSE Buena Vista, Flora y Fauna Hotel 17°29.925'S, 63°39.128'W. 440m F.I.T. 15–24 Dec 2003. S. & J. Peck” / “Caterino/Tishechkin Exosternini Voucher EXO-00291” (CMNC). **Paratypes:** 1: same data as type except: 6–15.xii.2003 (CMNC); 1: same data as type except: 7–12.v.2004, FIT, A.R. Cline (AKTC).

#### Other material.

**ECUADOR: Orellana:** 1:Yasuní Res. Stn., mid.Rio Tiputini, 0°40.5'S, 76°24'W, 23–30.vi.1999, FIT, C.E. Carlton & A.K. Tishechkin (LSAM); 1: Parque Nac. Yasuní, Via Maxus at Puente Piraña, 0°39.5'S, 76°26'W, 14–20.vii.2008, FIT, A.K. Tishechkin (AKTC). **PERU: Junín:** 1: ~16km NW Satipo, Rio Venado, 11°11.677'S, 74°46.137'W, 1150m, 3–8.iii.2010, A.V. Petrov (AKTC), 1: 8–14.iii.2010, A.V. Petrov (MUSM).

#### Diagnostic description.

This species is very closely related and similar to *Operclipygus hospes*, differing mainly in the following characters: length: 1.87–1.93 mm, width: 1.44–1.62 mm; sides of frontal stria rounded, central portion complete, sinuate, slightly recurved dorsad at middle; epistoma shallowly depressed at middle; antennal club with upper basolateral sensory pit; pronotal disk with numerous (>30) coarse punctures at sides; lateral submarginal stria complete or barely abbreviated at base; anterior submarginal stria distinctly crenulate, recurved posterad for very short distance (~1/8 pronotal length); median pronotal gland openings present beyond apices of recurved anterior stria, nearly one-fourth pronotal length from anterior margin; elytra with outer subhumeral stria present in apical half, inner subhumeral stria absent, striae 1-3 complete, 4^th^ stria barely abbreviated at base, 5^th^ stria present in apical half to two-thirds; sutural in apical three-fourths; prosternal keel produced posteriorly, carinal striae short, close, subparallel anteriorly, united in narrow arch about one-fourth from presternal suture; mesoventrite deeply emarginate; propygidium with uniform, shallow punctures separated by slightly less than their diameters; pygidium with dense, coarse ground punctation, with coarser punctures irregularly interspersed, slightly denser toward basal margin; marginal pygidial sulcus complete, deep, crenulate along inner edge. Male genitalia largely indistinguishable from that of *Operclipygus hospes*, but aedeagus with more distinct subapical cleft, basically identical to that of *Operclipygus subterraneus* (Fig. 36H).

#### Remarks.

As discussed above, *Operclipygus ignifer* is very closely related to *Operclipygus hospes*, sharing both genitalic characters and the presence of a basolateral sensory pit on the antennal club. They can be easily distinguished, however, by the numerous very coarse punctures on the sides of the pronotum of *Operclipygus ignifer* (Fig. 34E), as well as its large size and slightly longer prosternal carinal striae. The aedeagus of *Operclipygus ignifer* also bears a more distinct subapical cleft than *Operclipygus hospes*, more or less indistinguishable from that of the following species.

The type series is limited to those specimens from Santa Cruz, Bolivia, as some minor variation can be observed in superficial characters in specimens from Ecuador and Peru.

#### Etymology.

This species’ name refers to its flame-shaped prescutellar impression (Fig. 34E).

### Operclipygus subterraneus sp. n.

urn:lsid:zoobank.org:act:F43D0437-2D61-47B1-B189-0DE7B327B9DE

http://species-id.net/wiki/Operclipygus_subterraneus

Figs 34F–H 36F–H Map 12

#### Type locality.

BRAZIL: Rio De Janeiro: 17 km E Nova Friburgo [22°23.1'S, 42°33.5'W].

#### Type material.

**Holotype male**: “**BRASIL: RIO DE JANEIRO**, 17 km E Nova Friburgo, 22°23'04"S, 42°33'30"W, 750m, 29.I.2000, F. Génier & S. Ide, secondary mountain Atlantic for. ex. f.i.t., day 4–9, FG2000–58”/ “Caterino/Tishechkin Exosternini Voucher EXO-00290” (CMNC). **Paratypes** (94): **BRAZIL: Minas Gerais**: 7: Viçosa, 20°45'S, 42°53'W, ix.1999, primary Atlantic forest, E. Grossi (CHND); 2: Ingaí, Res. Boqueirão, nr. Lavras, 21°21'S, 44°59'W, 20.xi.2002, FIT, gallery forest, F. Frieiro-Costa & F.Z. Vaz-de-Mello (AKTC), 3: 13.xi.2002, FIT, gallery forest, F. Vaz-de-Mello & F. Frieiro-Costa (AKTC, CEMT), 1: 29.x.2002, FIT, gallery forest, F. Frieiro-Costa & F.Z. Vaz-de-Mello (AKTC); 1: Passa Quatro, Sul de Minas (BMNH); **Paraná:** 1: Piraquara, Mananciais da Serra, 25°29'77'W, 48°58.90'W, 1000m, i.2007, FIT, P .Grossi & D. Parizotto (CHND); 4: Guartelá, Campo São Paulo, 24°32'S, 50°17'W, 900m, xi.2007, pitfall, E. Grossi (CHND); 1: Campina Grande do Sul, xii.2004, pitfall, Armadilha, F.W.T. Leivas (UFPR); 2: Campina Grande do Sul, Estrada de Mandacaia, 26.xii.2008, FIT, F.W.T. Leivas (UFPR); 2: Curitiba, 25°26'44.5"S, 49°13'56.6"W, 18.iii.2009, in buried carrion, R.C. Correa (UFPR), 1: 10.xii.2008, Terra Acima, R.C. Correa (UFPR); 1: Curitiba, Campus UFPR, Mata Cap_ [?illegible] do Tigre, 5.iv.2003, P. Grossi (UFPR); 3 (on one card): Curitiba, Centro Politecnico, 16.xii.2009, in buried carrion, R. C. Correa (UFPR); 1: Tibagi, Parque Estad. Guartelá, 24.5663°S, 50.2570°W, 12.xii.2011, 15.xii.2011, FIT, forest, M.S. Caterino & A.K. Tishechkin, DNA Extract MSC-2272 (SBMNH); 1: Vila Velha - IAPAR, 18.x.1999, Ganho & Mannoni (UFPR); 1: [locality lost] 2011, DNA Extract MSC-2260; **Rio de Janeiro:** 12: 17km E Nova Friburgo, 22°23'04"S, 42°33'30"W, 750m, 29.i.2000, FIT, secondary montane Atlantic for., F. Genier & S. Ide (CMNC, FMNH, MSCC, AKTC, CHND), 3: 23.i.2000 (CMNC); 5: Nova Friburgo, Sans Souci, 9–15.xi.2009, FIT, E. Grossi (UFPR); 27: Nova Friburgo, 22°16'S, 42°32'W, 26–31.x.2009, FIT (CHND); 2: Corcovado, Guanabara, x.1968, Alvarenga & Seabra (UFPR); **Santa Catarina**: 1: Nova Teutonia, xii.1953, F. Plaumann (FMNH), 1: xii.1972, F. Plaumann (FMNH), 2: 18.xii.1953, F. Plaumann (FMNH), 1: 25.iii.1954, F. Plaumann (FMNH), 1: 18.xi.1954, F. Plaumann (FMNH), 1: 29.v.1954, F. Plaumann (FMNH), 1: 12.xi.1953, F. Plaumann (FMNH), 1: 16.viii.1953, F. Plaumann (FMNH); **São Paulo**: 1: Vila Mariana, 23°35'00"S, 046°38'00"W, vii.1935, B. Pohl (AKTC); 1: São Miguel, Turvo River, 24°01'S, 48°00'W, 800m, xii.1963, F. Plaumann (FMNH); 1: Ilha Santo Amaro nr. Santos, 26.iii.1916, G.E. Bryant (BMNH).

#### Diagnostic description.

Length: 1.59–1.65 mm, width: 1.31–1.35 mm; body rufescent, subquadrate, subdepressed; frons depressed at middle; frontal corners prominent above antennal bases, epistoma and labrum coplanar;frontal stria short, detached from sides; labrum flat, weakly emarginate at apex; pronotum with sides subparallel in basal two-thirds; prescutellar impression short, linear, subequal in length to scutellum, disk with ~20 coarse punctures clustered posterolaterad ends of recurved anterior submarginal stria; lateral submarginal pronotal stria complete;anterior submarginal pronotal stria recurved about one-fourth pronotal length; median pronotal gland openings near anterior corners of anterior submarginal stria, about 4 puncture widths from anterior margin; elytra with one complete epipleural stria, outer subhumeral stria present in apical half, inner subhumeral stria absent, striae 1-4 more or less complete, 5^th^ stria present in apical half, sutural stria present in apical two-thirds; prosternal keel weakly produced at base, carinal striae about four-fifths keel length, narrowly united anteriorly, divergent, faintly united at base; mesoventrite emarginate, marginal stria complete; mesometaventral stria broadly arched forward two-thirds from suture to anterior mesoventral margin; metaventral stria extended to near outer third of metacoxa, abruptly bent laterad at apex; 1^st^ abdominal ventrite with two complete lateral striae parallel; propygidium with moderate-sized shallow punctures separated by slightly less than their diameters; pygidium with ground punctation not extremely dense, with coarser punctures sparsely interspersed; marginal pygidial sulcus complete, fine but deep, weakly crenulate.Male genitalia very similar to that of *Operclipygus hospes* (Figs 36A–D), except T8 narrower, with deeper apical emargination, T9 with fine, acute basolateral processes, and tegmen with more distinct subapical cleft (Fig. 36H).

#### Remarks.

The form of the frons and the frontal stria is unique in this species, having the antennal bossae prominent with the frontal stria broadly interrupted at the sides (Fig. 34G). The head is relatively large compared to most relatives (Fig. 34F), and this in combination with the subquadrate body makes it relatively easy to recognize. This is the species cited as *Operclipygus hospes*, as having some forensic significance, by Corréa et al. (2012).

#### Etymology.

This species names refers to the discovery of numerous specimens in association with subterranean carrion by entomologists with UFPR.

### Operclipygus callifrons sp. n.

urn:lsid:zoobank.org:act:C68D1B5F-E849-4D1B-8600-86EF11561DED

http://species-id.net/wiki/Operclipygus_callifrons

Figs 35A–B 3 Map 12

#### Type locality.

ECUADOR: Orellana:Yasuní Research Station [0°40.5'S, 76°24'W].

#### Type material.

**Holotype male**: “**Ecuador:** Napo, mid.Rio Tiputini, Yasuní Res. Stn. 0°40.5'S, 76°24'W, FIT #1, 23–30 Jun 1999. AKT#032, C.Carlton & A.Tishechkin”/ “LSAM0013254” (FMNH). **Paratypes** (42): **ECUADOR: Orellana:** 5:Yasuní Res. Stn., mid.Rio Tiputini, 0°40.5'S, 76°24'W, 17–23.vi.1999, FIT, C.E. Carlton & A.K. Tishechkin (LSAM, MSCC, AKTC), 5: 18–23.vi.1999 (LSAM, CHND), 3: 20–29.vi.1999 (LSAM), 7: 23–28.vi.1999 (LSAM), 1: 28.vi–5.vii.1999 (LSAM), 2: 4–17.vii.1999 (LSAM), 5: 5–11.vii.1999 (LSAM), 1: 20–26.vii.1999 (LSAM), 1: 23.vii–4.viii.1999 (USFQ), 1: 25.vii–3.viii.1999 (LSAM), 1: 26.vii–4.viii.1999 (LSAM), 1: 215m, 5–10.ix.1999, FIT, primary forest, E.G. Riley (LSAM); 5: Parque Nac. Yasuní, Via Maxus at Puente Piraña, 0°39.5'S, 76°26'W, 14–20.vii.2008, FIT, A.K. Tishechkin (AKTC); 1: Tiputini Biodiversity Station, 0.6376°S, 76.1499°W, 4–9.vi.2011, FIT, M.S. Caterino & A.K. Tishechkin, DNA Extract MSC-2191 (SBMNH), 1: 220m, 5–25.ix.2000, D.J. Inward & K.A. Jackson (BMNH); **Sucumbíos:** 1: Sacha Lodge, 0°28'14"S, 76°27'35"W, 270m, 21–24.iii.1999, FIT, R. Brooks (SEMC).

#### Other material.

**BRAZIL: Pará:** 1:Tucuruí, 3°45'S, 49°40'W, iv.1986, FIT (CHND); **Mato Grosso:** 1: Cotriguaçu, Fazenda São Nicolau, Matinha, 9°50.3'S, 58°15.05'W, x.2009, FIT, F.Z. Vaz-de-Mello, 1: xii.2010, FIT, F.Z. Vaz-de-Mello (CEMT). **PERU: Junín:** 2: 11km NE Puerto Ocopa, Los Olivos, 11°7.00'S, 74°15.52'W, 1200m, 30–31.iii.2009, Window trap, A.V. Petrov (AKTC); 1: 4km NE Puerto Ocopa, Santa Cruz, 11°3.8'S, 74°17.0'W, 1150m, 19.ii.2006, A.V. Petrov (AKTC); 1: 7.5km NE Puerto Ocopa, Cananeden, 11°4.9'S, 74°16.1'W, 1180m, 6.ii.2008, A.V. Petrov (MUSM), 1: 11.i.2007, A.V. Petrov (MUSM); 2: **Loreto:** Campamento San Jacinto, 2°18.75'S, 75°51.77'W, 175–215m, 3.vii.1993, FIT, R. Leschen (SEMC), 2: 5.vii.1993, FIT, R. Leschen (SEMC); 1: 68km SW Iquitos to Nauta, Rio Itaya, 4°11'S, 73°26'W, 110m, 4.ii.2007, A.V. Petrov (AKTC), 1: 10.ii.2007, A.V. Petrov (MUSM).

#### Diagnostic description.

Length: 1.44–1.68 mm, width: 1.12–1.31 mm; frontal stria complete, rather high on the frons, with broad bead above antennal insertions; antennal bosses prominent; supraorbital stria complete, continuous with sides of frontal stria; epistoma weakly depressed, shallowly emarginate along anterior margin, coplanar with labrum; prescutellar impression linear, about 1.5× length of scutellum; lateral marginal pronotal stria present only present in anterior third of pronotum, subequal in length to recurved arms of anterior submarginal stria; pronotal disk with ~12 coarse lateral punctures; median pronotal gland openings about 6 puncture widths from anterior margin; elytra with outer subhumeral stria prsent in apical half, striae 1-4 complete (4^th^ occasionally slightly abbreviated at base), 5^th^ stria present in apical half, sutural stria present in apical three-fourths; prosternal keel produced posteriorly, carinal striae short anteriorly, tending to be faint to obsolete posteriorly; marginal mesoventral stria complete; mesometaventral stria narrowly arched forward at middle nearly to marginal mesoventral stria; lateral metaventral stria extended toward outer fourth of metacoxa, occasionally bent laterad at apex; propygidium uniformly covered with small round punctures separated by their diameters; pygidium with ground punctation moderately dense, coarser punctures irregularly but conspicuously interspersed; marginal pygidial sulcus fine, deep, crenulate along inner edge, relatively smooth on outer edge. Male genitalia more or less indistinguishable from those of *Operclipygus subterraneus* (see Figs 36F–H); T8 slightly shorter, with sides slightly more strongly convergent, basal emargination narrower.

#### Remarks.

**Figure 35.**
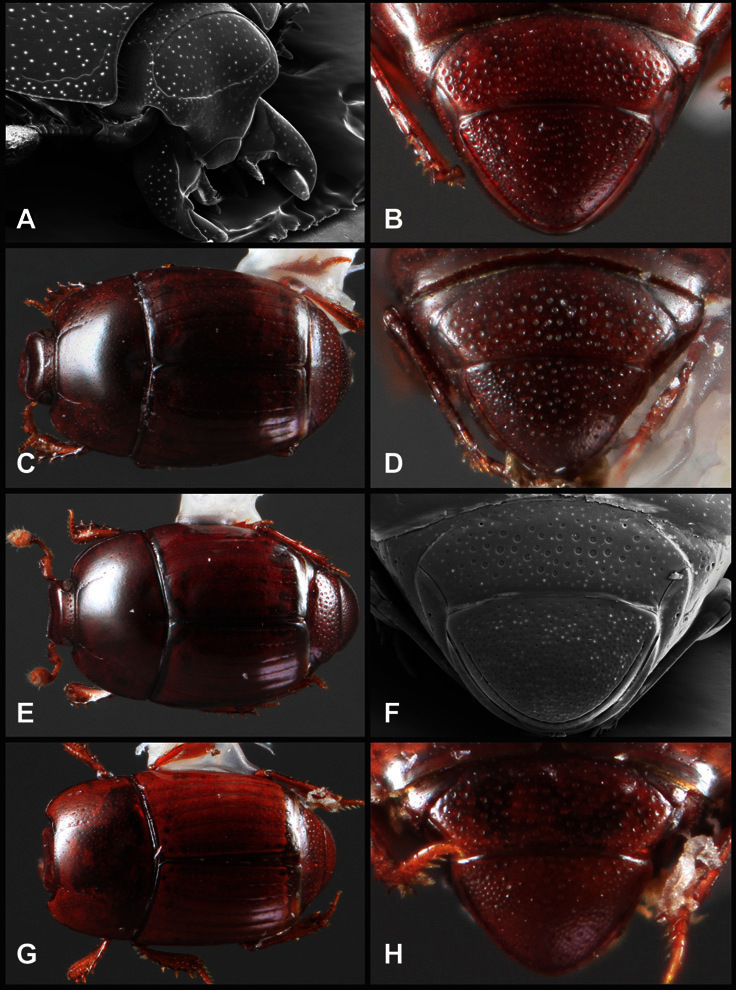
*Operclipygus hospes* group. **A** Frons of *Operclipygus callifrons* **B** Pygidia of *Operclipygus callifrons* **C** Dorsal habitus of *Operclipygus prominens* **D** Pygidia of *Operclipygus prominens* **E** Dorsal habitus of *Operclipygus tiputinus* **F** Pygidia of *Operclipygus tiputinus* **G **Dorsal habitus of *Operclipygus minutus* **H** Pygidia of *Operclipygus minutus*.

This species can usually be recognized by its shortened lateral submarginal pronotal stria, complete frontal stria situated relatively high on the frons (Fig. 35A), depressed epistoma coplanar with labrum, prosternal striae which tend to be obsolete basally, and pygidial sulcus which is narrowly rounded to subangulate around the apical margin (Fig. 35B). Due to the broad distribution and some intraspecific variation we restrict the type series to those specimens from eastern Ecuador.

**Figure 36.**
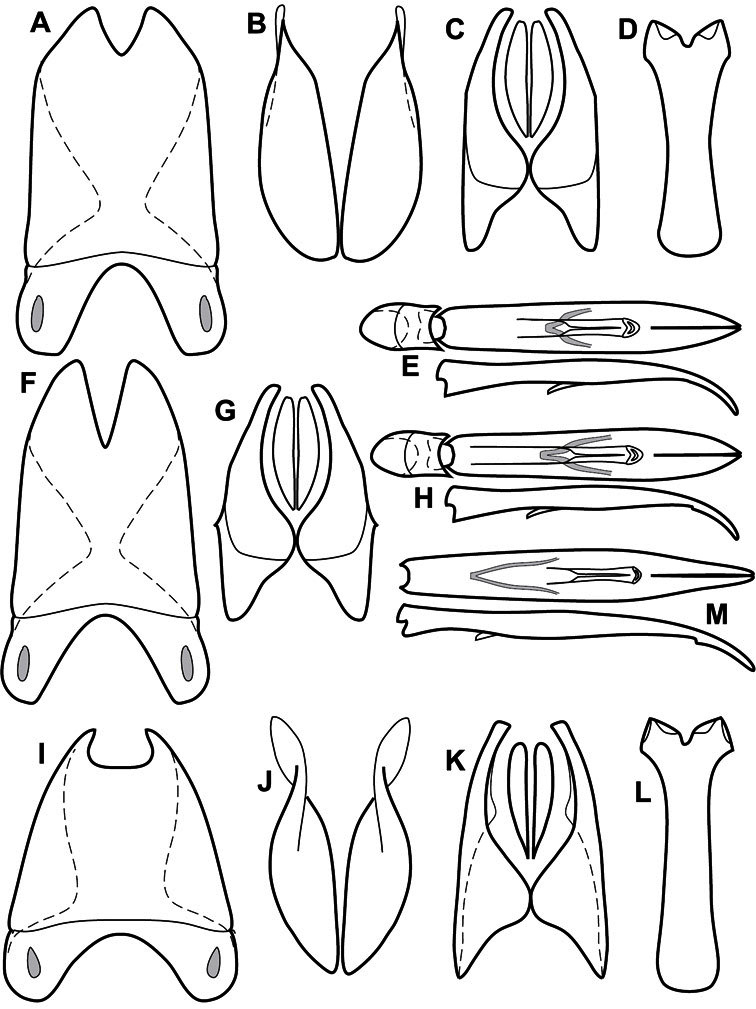
Male genitalia of *Operclipygus hospes* group. **A** T8 of *Operclipygus hospes* **B** S8 of *Operclipygus hospes* **C** T9 & T10 of *Operclipygus hospes* **D** S9 of *Operclipygus hospes* **E** Aedeagus, dorsal and lateral views, of *Operclipygus hospes* **F** T8 of *Operclipygus subterraneus* **G **T9 & T10 of *Operclipygus subterraneus* **H** Aedeagus, dorsal and lateral views, of *Operclipygus subterraneus* **I** T8 of *Operclipygus incisus* **J** S8 of *Operclipygus incisus* **K** T9 & T10 of *Operclipygus incisus* **L** S9 of *Operclipygus incisus* **M** Aedeagus, dorsal and lateral views, of *Operclipygus incisus*.

#### Etymology.

This species’ name refers to the frontal calli, or prominent bosses above the antennal bases.

### Operclipygus colombicus sp. n.

urn:lsid:zoobank.org:act:28114B97-4F68-49D0-BDC5-B531C83ABED5

http://species-id.net/wiki/Operclipygus_colombicus

Map 12

#### Type locality.

COLOMBIA: Vaupés: Caparú Biological Station [1°04'S, 69°31'W].

#### Type material.

**Holotype male**: “**COLOMBIA**: Vaupés, Rio Apoporis, Est. Biol. Caparú, 1.1°S, 69.5°W 27ix–1.xii.1995 Black-water terrace forest on sandy soils, F.I.T. B.D.Gill”/ “Caterino/Tishechkin Exosternini Voucher EXO-00289” (CNCI). **Paratypes** (14): 13, same data as type (BDGC, FMNH, MSCC, AKTC); 1: **COLOMBIA**: **Vaupés:** Parque Nac. Mosiro-Itajura (Caparú), Centro Ambiental. 1°04'S, 69°31'W. 60m. F.I.T. 20–30.i.2003. D.Arias & M.Sharkey (IAVH).

#### Diagnostic description.

This species is extremely similar to the preceding, differing as follows: length: 1.37–1.40 mm, width: 1.09–1.12 mm; pronotum with anterior submarginal stria with recurved ends slightly longer, between one-third and one-half pronotal length; elytra with 1^st^ dorsal stria frequently obsolete at apex, 4^th^ stria present in apical half, 5^th^ stria consistently very slightly shorter than 4^th^; prosternal keel with carinal striae not weak toward base, generally connected posteriorly, subparallel toward front, united in anterior arch about one-fourth keel length from presternal suture; propygidium with punctures smaller, denser, separated by about one-half their diameters; pygidial marginal sulcus deep, but simple with margins only very faintly crenulate. Male genitalia indistinguishable from those of *Operclipygus subterraneus* (see Figs 36F–H).

#### Remarks.

This species is generally similar in all respects to the preceding, but may be separated by the distinctly shortened 4^th^ dorsal stria.

#### Etymology.

This species is named for the country where it is found.

### Operclipygus incisus sp. n.

urn:lsid:zoobank.org:act:372569E6-48F5-42B5-887A-5D170D5436E7

http://species-id.net/wiki/Operclipygus_incisus

Figs 36I–M Map 12

#### Type locality.

FRENCH GUIANA: 33.5 km S and 8.4 km NW of Highway N2 on Highway D5 [4°48.3'N, 52°28.7'W].

#### Type material.

**Holotype male**: “FRENCH GUIANA, Cayenne, 33.5 km S and 8.4 km NW of Hwy N2 on Hwy D5, 30 m, 4°48'18"N, 52°28'41"W 29 MAY-9 JUN 1997; J.Ashe,R.Brooks FG1AB97 171, ex:flight intercept trap”/ “SM0102418 KUNHM-ENT” (SEMC). **Paratypes** (9): 5: same data as type (FMNH, MSCC, AKTC, MNHN); 3: same data as type except 26–28.v.1997, FIT, J. Ashe, R. Brooks (SEMC, CHND); 1: 8.4km SSE Roura, , 4°40'41"N, 52°13'25"W, 200m, 22–24.v.1997, FIT, J. Ashe, R. Brooks (SEMC).

#### Other material.

**BRAZIL: Amapá:** 6: Serra do Navio, 0°59'N, 52°00'W, 1–14.v.1991, FIT (CHND, MNHN); **Amazonas**: 1: INPA/Smithsonian Res., Manaus, 2°25'S, 59°50'W, ii.1994, Winkler method, leaf litter, terra firma forest, R. Didham (BMNH); 2: Reserva Ducke, 26km NE Manaus, ii.1995, FIT, M.G.V. Barbosa (BMNH); **Pará:** 1: Tucuruí, 3°45'S, 49°40'W, iv.1986, FIT (CHND); 2: IPEAN, Utinga, Belém, 1°27'S, 48°26'W, xii.1984, FIT (CHND). **FRENCH GUIANA:** 1: Saül, 7km N, 1km NW Les Eaux Claires, along Route de Bélizon trail, 3°39'46"N, 53°13'19"W, 280m, 4–8.vi.1997, FIT, J. Ashe, R. Brooks (SEMC); 1: Cayenne, Saül, 7km N, Les Eaux Claires, 3°39'46"N, 53°13'19"W, 220m, 31.v–3.vi.1997, FIT, J. Ashe, R. Brooks (SEMC); 1: Belvèdére de Saül, point de vue, 3°1'22"N, 53°12'34"W, 17.i.2011, FIT, SEAG (CHND); 1: Mont Tabulaire, Itoupé, 3°1.82'N, 53°6.40'W, 400m, 31.iii.2010, Window trap, SEAG (CHND), 1: 24.iii.2010, Window trap, SEAG (CHND), 3: 17.iii.2010, Window trap, SEAG (CHND). **SURINAME**: **Saramacca**: 1: West Suriname Road, 139km WSW Zanderji Airport, ca, 5°9'N, 56°4'W, 40–50m, 10–14.vi.1999, FIT, Z. Falin, B. DeDijn (SEMC); **Sipaliwini**: 1:CI-RAP Surv. Camp 3: Wehepai SE Kwamala, 2°21.776'N, 56°41.861'W, 237m, 3–7.ix.2010, FIT, T. Larsen & A.E.Z. Short (SEMC).

#### Diagnostic description.

This species is very similar to both of the preceding, differing as follows: length: 1.44–1.47 mm, width: 1.09–1.12 mm; frons less strongly impressed at middle, antennal bosses not as prominent; frontal stria usually complete across front, rarely narrowly interrupted at sides; epistoma depressed, very weakly convex along apical margin, but basically coplanar with flat labrum; prescutellar impression narrow, linear, almost twice as long as scutellum; lateral submarginal stria complete; lateral portion of pronotal disk with ~15 coarse punctures; anterior submarginal pronotal stria strongly and slightly obliquely recurved posterad, reaching nearly to the middle of the pronotal disk; propygidium densely covered with small round punctures separated by about half their diameters. Male genitalia (Fig. 36I–M): accessory sclerites present, rather small; T8 shorter, with sides more strongly convergent, rounded to apex, apical emargination broader, shallow, rounded; S8 less broadly rounded basally, with apical guide portion accounting for nearly half of overall length; T9 with sides more evenly rounded from base to apex, apices only weakly convergent, obliquely truncate; S9 desclerotized along midline, almost parallel sided, only weakly widened to base, lateral flanges wide but without prominent corners, apex with small median emargination, apical flanges short and separate; tegmen more or less parallel-sided in basal two-thirds, weakly narrowed to base, narrowed to apex, apical fourth curved downward, with subapical ventral cleft, medioventral process long, its apex narrowly truncate, projecting beneath about one-fourth from base; basal piece about one-fourth tegmen length (broken in type specimen); median lobe about one-third tegmen length, filamentous portions of proximal apodemes one-half the length of thick portions.

#### Remarks.

This species is best diagnosed by the complete frontal stria with the antennal bosses only weakly prominent, the complete lateral submarginal pronotal striae, long recurved arms of anterior submarginal pronotal stria, and the complete 4^th^ elytral stria. Specimens from Amapá and Manaus tend to have the lateral submarginal pronotal stria abbreviated posteriorly. We limit the type series to those specimens from localities in coastal French Guiana.

#### Etymology.

This species’ name refers to the rather elongate, narrowly incised prescutellar impression.

### Operclipygus prominens sp. n.

urn:lsid:zoobank.org:act:ABB39111-E411-43DB-9765-DF4CCFC16B41

http://species-id.net/wiki/Operclipygus_prominens

Figs 35C–D Map 13

#### Type locality.

BRAZIL: Amapá: Serra do Navio [0°59'N, 52°00'W].

#### Type material.

**Holotype male**: “**BRASIL: Amapá**, Serra do Navio 0°59'N, 52°00'W, Piège d’interception 1–14.v.1991”/ “Caterino/Tishechkin Exosternini Voucher EXO-00296” (UFPR). **Paratypes** (3): 2: same data as type (CHND, FMNH); **FRENCH GUIANA:** 1: Belvèdére de Saül, point de vue, 3°1'22"N, 53°12'34"W, 7.ii.2011, FIT, SEAG (CHND).

#### Other material.

**BRAZIL: Pará:** 1:IPEAN, Utinga, Belém, 1°27'S, 48°26'W, v.1985, FIT (CHND), 1: xii.1984, FIT (MSCC); 1: Tucuruí, 3°45'S, 49°40'W, 9–17.xii.1985, FIT (AKTC).

#### Diagnostic description.

Nearly indistinguishable from *Operclipygus callifrons*, except as follows; length: 1.25–1.37 mm, width: 0.97–1.06 mm; body distinctly smaller, slightly more elongate and dorsally convex; anterior submarginal pronotal striae with sides recurved posterad between one-third to one-half pronotal length; lateral submarginal striae strongly abbreviated posteriorly, nearly absent in one of the paratypes; elytra with ground punctation rather conspicuous; coarse punctures of propygidium very sparse, separated by 2× their diameters or more, absent from fairly broad basal band; pygidium with coarse punctures slightly smaller and sparser than in *Operclipygus callifrons*, marginal pygidial sulcus fine, deep. Male genitalia more or less indistinguishable from those of *Operclipygus subterraneus* (see Figs 36F–H), though aedeagus smaller in overall size, slightly more narrowed to base.

#### Remarks.

The small size, strongly reduced lateral submarginal pronotal stria (Fig. 35C), unusually coarse elytral ground punctation, and prominent antennal bossae will help distinguish this species from its relatives. The specimens from Pará (south side of the Amazon) differ slightly in frontal appearance and in propygidial punctation, and are excluded from the type series.

**Map 13.**
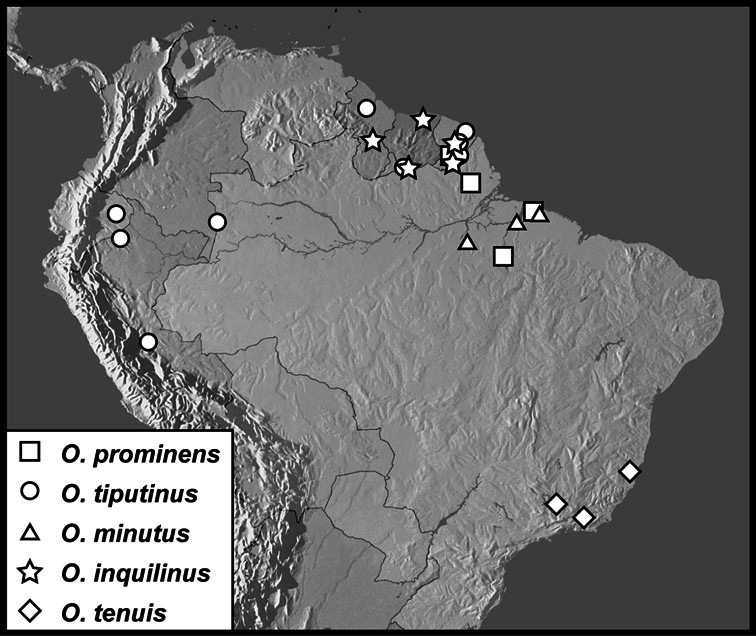
Records of the *Operclipygus hospes* group.

#### Etymology.

The name of this species refers to the relatively prominent frontal corners.

### Operclipygus tiputinus sp. n.

urn:lsid:zoobank.org:act:FAED1E56-DF00-4F27-9A3B-230C7C624F80

http://species-id.net/wiki/Operclipygus_tiputinus

Figs 35E–F Map 13

#### Type locality.

ECUADOR: Orellana:Yasuní Research Station [0°40.5'S, 76°24'W].

#### Type material.

**Holotype male**: “**Ecuador:** Napo, mid.Rio Tiputini, Yasuní Res. Stn. 0°40.5'S, 76°24'W, FIT#M2 20–29 Jun 1999. CEC#030 C.Carlton & V.Moseley” / “LSAM 0013234” / “*Operclipygus* sp #11, Hist 132, Yasuní NP Inventory A.K.Tishechkin det 2010” (FMNH). **Paratypes** (22): **ECUADOR: Orellana:** 1:Parque Nac. Yasuní, Via Maxus at Puente Piraña, 0°39.5'S, 76°26'W, 14–20.vii.2008, FIT, A.K. Tishechkin (AKTC); 2: Tiputini Biodiversity Station, 0.6376°S, 76.1499°W, 4–9.vi.2011, FIT, M.S. Caterino & A.K. Tishechkin, DNA Extracts MSC-2183 & MSC-2288 (SBMNH), 10: 2–9.vi.2011, FIT, M.S. Caterino & A.K. Tishechkin (SBMNH, FMNH, AKTC, MSCC, CHND, USFQ), 1: 5–25.ix.2000, D.J. Inward & K.A. Jackson (BMNH), 2: 27–31.vii.2008, FIT, A.K. Tishechkin (LSAM); **Napo**: 2: 20 km S Tena, 600m, 11.vii.1976, Berlese, forest litter, S. Peck (FMNH).

#### Other material.

**COLOMBIA: Vaupés:** 2: Est. Biol. Caparú, Rio Apoporis, 1.1°S, 69.5°W, 27.ix–1.xii.1995, FIT, Black-water terrace forest on sandy soils, B.D. Gill (BDGC); 2: Parque Nac. Mosiro-Itajura (Caparú), Centro Ambiental, 1°04'S, 69°31'W, 60m, 20–30.i.2003, FIT, D. Arias & M.Sharkey (IAVH). **FRENCH GUIANA:** 1: Cayenne, 33.5km S and 8.4km NW of Hwy N2 on Hwy D5, 4°48'18"N, 52°28'41"W, 30m, 29.v–9.vi.1997, FIT, J. Ashe, R. Brooks (SEMC); 4: Mont Tabulaire, Itoupé, 3°1.82'N, 53°6.40'W, 400m, 17.iii.2010, Window trap, SEAG (CHND), 1: 23.iii.2010, Window trap, SEAG (CHND); 2: Rés. Natur. des Nouragues, Camp Inselberg, 4°05'N, 52°41'W, 22.ix.2010, Window trap, SEAG (MNHN); 1: Belvèdére de Saül, point de vue, 3°1'22"N, 53°12'34"W, 10.xii.2010, Window trap, SEAG (CHND). **GUYANA: Mazaruni Potaro**: 3: Takutu Mountains, 6°15'N, 59°5'W, 11.xii.1983, Window trap, montane rainforest near logging area, P.J. Spangler, R.A. Faitoute & W.E. Steiner (USNM). **PERU: Junín:** 1: 11km NE Puerto Ocopa, Los Olivos, 11°3.00'S, 74°15.52'W, 1200m, 26–30.iii.2009, fig fruitfall, A.K. Tishechkin, DNA Extract MSC-2228 (AKTC); **Loreto:** 1: 1.5km N Teniente Lopez, 2°35.66'S, 76°06.92'W, 210–240m, 18.vii.1993, FIT, R. Leschen (SEMC), 3: 20.vii.1993, FIT, R. Leschen (SEMC), 2: 23.vii.1993, FIT, R. Leschen (SEMC), 1: 26.vii.1993, FIT, R. Leschen (SEMC), 1: 28.vii.1993, FIT, R. Leschen (SEMC). **SURINAME: Sipaliwini:** 2: CI-RAP Surv. Camp 3: Wehepai SE Kwamala, 2°21.776'N, 56°41.861'W, 237m, 3–7.ix.2010, FIT, T. Larsen & A.E.Z. Short (SEMC); 1: CI-RAP Surv. Camp 2: Sipaliwini River, 2°10.521'N, 56°47.244'W, 210m, 27.viii-1.ix.2010, FIT, T. Larsen & A.E.Z. Short (SEMC); 1: CI-RAP Surv. Camp 1: on Kutari River, 2°10.521'N, 56°47.244'W, 228m, 19–24.viii.2010, FIT, T. Larsen & A.E.Z. Short (SEMC).

#### Diagnostic description.

Length: 1.25–1.50 mm, width: 1.00–1.15 mm; frons flat to convex, frontal stria complete, more or less transverse across front, not displaced dorsad, antennal bases not particularly prominent; epistoma weakly depressed in middle, narrowly convex along anterior margin; labrum about twice as wide as long, straight across apical margin; pronotal disk with narrow prescutellar impression about 1.5× length of scutellum; ground punctation of pronotal disk fine and sparse, slightly more strongly impressed toward sides, with few (<8) small secondary punctures at sides; lateral submarginal pronotal stria complete, curving inward in front and ending freely; anterior submarginal pronotal stria recurved posterad at sides about one-third length of pronotal disk; median pronotal gland openings close, laterad anterior submarginal stria, sometimes disrupting its path, about 6 puncture widths from anterior margin; elytra with outer subhumeral stria present in apical half, inner subhumeral stria absent, 1^st^ stria complete to base, tending toward obsolete in apical fourth, striae 2-3 complete, 4^th^ stria varied, from present in only apical half to very nearly complete, 5^th^ stria present in apical half, sutural stria present in apical three-fourths; prosternal keel produced at base, carinal striae present in basal three-fourths of keel, slightly divergent and separate at base, united anteriorly; mesoventrite emarginate anteriorly, with complete marginal stria; mesometaventral stria broadly angulate forward to middle of mesoventral disk, continued by lateral metaventral stria extending posterolaterally toward posterior corner of metepisternum; 1^st^ abdominal ventrite with inner lateral stria weakly abbreviated apically, outer complete, parallel; propygidium with very fine, sparse ground punctures and small, round, shallow punctures separated by about 1.5× their diameters; pygidium with fine, dense ground punctures only, lacking coarser punctures, even along basal margin; marginal pygidial sulcus fine, shallow, frequently obsolete toward basal corners. Male genitalia indistinguishable from those of *Operclipygus subterraneus* (see Figs 36F–H).

#### Remarks.

The small size of this species, along with its pygidium (Fig. 35F), with only fine ground punctation, and having a fine, often basally abbreviated marginal sulcus, will adequately diagnose this species. The body outline (Fig. 35E) may appear interrupted at the humeri, which are generally more broadly rounded than the sides of the pronotum. The type series is limited to a relatively restricted area in eastern Ecuador, as some variation among localities is evident.

#### Etymology.

This species is named for the type locality's region, along the Rio Tiputini, in Amazonian Ecuador.

### Operclipygus minutus sp. n.

urn:lsid:zoobank.org:act:B7D20E38-5376-4CD7-933B-127318B88B1B

http://species-id.net/wiki/Operclipygus_minutus

Figs 35G–H 38A–D Map 13

#### Type locality.

BRASIL: Pará:Belém, Utinga [1°27'S, 48°26'W].

#### Type material.

**Holotype male**: “**BRASIL: Pará:** Belém, Utinga (IPEAN), 1°27'S, 48°26'W. Piège d’interception. v.1985” / “Caterino/Tishechkin Exosternini Voucher EXO-01776” (MNHN). **Paratypes** (5): **BRAZIL: Pará:** 1:IPEAN, Utinga, Belém, 1°27'S, 48°26'W, viii.1985, FIT (FMNH), 1: xi.1984, FIT (CHND); 2: Altamira - Marabá: km 18, 3°09'S, 52°03'W, v.1984, FIT (CHND, MSCC); 1: Barcarena, 1°30'S, 48°37'W, 13–25.vi.1991, FIT (AKTC).

#### Diagnostic description.

Length: 1.15–1.25 mm, width: 0.81–0.87 mm; body very small, rather elongate, parallel-sided; frons very shallowly depressed, frontal stria complete, transverse across front; supraorbital stria fine, complete, meeting sides of frontal stria; epistoma shallowly emarginate anteriorly, flat, coplanar with labrum; labrum with sides narrowed, apex weakly emarginate; left mandible with fairly prominent bifid tooth at base; right mandible with single acute tooth; pronotal disk with prescutellar impression linear, almost 2× as long as scutellum; lateral submarginal pronotal stria absent; anterior marginal pronotal stria interrupted for width of head, but meeting corner of anterior submarginal stria, which is close to margin, recurved posterad for approximately one-third pronotal length; median pronotal gland openings situated alongside anterior submarginal stria, about 4 puncture widths from anterior margin; elytra with outer subhumeral stria present in apical half, inner subhumeral stria absent, striae 1-4 complete, 5^th^ stria present in apical half, sutural stria present in apical three-fourths; ground punctation of elytral disks sparse but conspicuous; prosternal keel narrowly produced at base, carinal striae close, subparallel anteriorly, slightly diverging but connected basally, united in anterior arch about one-fourth from presternal suture; mesoventral margin emarginate, marginal stria abbreviated; mesometaventral stria narrowly arched forward to near mesoventral margin, displacing marginal stria; lateral metaventral stria extending toward middle of metacoxa, not abbreviated apically; 1^st^ abdominal ventrite with two complete lateral striae close and parallel; propygidium with ground punctation sparse, well impressed, conspicuous, with small, shallow, round punctures separated by about their diameters; pygidium with fine ground punctation very dense, with coarser punctures fairly sparsely interspersed; marginal pygidial sulcus complete, fine but well impressed. Male genitalia (Figs 38A–D): accessory sclerites present; T8 with sides subparallel, roundly converging to apex, apical emargination shallow, but with larger, distinctly desclerotized region of roughly the same shape surrounding it, basal emargination broad, shallow, basal membrane attachment line distad emargination by about one-third its depth; S8 widest at base, sides rounded, convergent to narrow subacute apices, apical guides narrow, evenly developed throughout length, halves separate, diverging from base; T9 wider, subparallel in middle half, narrowed to basal apodemes, with prominent angles where narrowed to apex, apices curved inward, weakly subacute; T10 with halves separate; S9 desclerotized along midline, broad, narrowest near apex, widened to broad, truncate base, lateral flanges wide, widened laterally to prominent basal and apical corners, apex with narrow median emargination, apical flanges low and separate; tegmen with sides rounded, widest at middle, narrowed to subacute apices, curved downward in apical fourth, with ventral subapical cleft, medioventral process long, with truncate apex, projecting strongly beneath about one-third from base; basal piece about one-third tegmen length; median lobe short, only about one-fourth tegmen length.

#### Remarks.

This species is unusual in its small and narrowly elongate body form (Fig. 35G), as well as its lack of lateral submarginal pronotal stria. It is very similar to *Operclipygus impositus*, but is generally slightly more convex, with slightly rounder sides. Male genitalia should be examined for unambiguous identification.

#### Etymology.

Even by *Operclipygus hospes* group standards this is a small species, and is named to reflect this fact.

### Operclipygus inquilinus sp. n.

urn:lsid:zoobank.org:act:3CCC345A-79A2-457D-81F2-28A7A4F13095

http://species-id.net/wiki/Operclipygus_inquilinus

Figs 37A–B Map 13

#### Type locality.

GUYANA: Region 8: 1 km W Kurupukari, Iwokrama Field Station [4°40.3'N, 58°41.1'W].

#### Type material.

**Holotype male**: “GUYANA: Region 8 Iwokrama Forest, 1 km W Kurupukari, Iwokrama Field Stn., 60 m 4°40'19"N, 58°41'04"W 21 MAY 2001, R. Brooks,Z. Falin, GUY1BF01 005 ex: *Acromyrmex hystrix* refuse pile” / “SM0568398 KUNHM-ENT” (SEMC). **Paratypes** (9): 1: same data as type (SEMC); **GUYANA: Region 8**: 2, Kabocalli Field Stn., Iwokrama Forest, 4°17'4"N, 58°30'35"W, 60m, 3–5.vi.2001, FIT, R. Brooks & Z. Falin (SEMC, MSCC); **FRENCH GUIANA:** 1: Régina, Réserve des Nouragues, 4°2.27'N, 52°40.35'W, 28.i.2010, FIT, SEAG (CHND); 1: Rés. Natur. des Nouragues, Camp Inselberg, 4°05'N, 52°41'W, 20.vii.2009, Window trap, SEAG (FMNH); 1: Mont tabulaire Itoupé, 3°1.82'N, 53°6.40'W, 400m, 17.iii.2010, FIT, SEAG (CHND); 1: Belvèdére de Saül, point de vue, 3°1'22"N, 53°12'34"W, 30.xi.2010, Window trap, SEAG (AKTC). **SURINAME: Para**: 1: nr. Overbridge River Resort, 5°31.8'N, 55°3.5'W, 15–18.ii.2010, FIT, C. Gillet, P. Skelley, W. Warner (FSCA); **Sipaliwini**: 1:CI-RAP Surv. Camp 1: on Kutari River, 2°10.521'N, 56°47.244'W, 228m, 19–24.viii.2010, FIT, T. Larsen & A.E.Z. Short (SEMC).

#### Diagnostic description.

Length: 1.28–1.59 mm, width: 0.97–1.15 mm; body rufescent, depressed, elongate, sides subparallel; frons and epistoma strongly depressed at middle, antennal bosses strongly prominent; frontal stria absent; supraorbital stria very fine, inconspicuous, or absent; epistoma weakly convex along anterior margin; labrum flat, slightly sinuate along anterior margin; both mandibles with strong, simple basal teeth; pronotal disk with thin, linear prescutellar impression about 1.5× scutellar length, ground punctation sparse, fine, with few coarser punctures at sides, particularly posterolaterad ends of anterior submarginal stria; lateral submarginal pronotal stria variably strongly reduced, present only in anterior corners to completely absent; anterior submarginal stria recurved somewhat obliquely posterad, reaching middle of pronotum; elytra with outer subhumeral stria present in apical half, inner subhumeral stria absent, striae 1-4 complete, 5^th^ stria present in apical half, sutural stria present in apical four-fifths; base of prosternal keel projecting, but rather bluntly rounded posteriorly, with carinal striae strongly shortened, meeting in narrow anterior arch just anterad midpoint of keel, faintly connected basally; mesoventral margin emarginate at middle, marginal stria complete; mesometaventral stria strongly arched forward at middle, reaching anterior third of mesoventral disk, continued posterolaterally by lateral metaventral, which extends toward middle of metacoxa; 1^st^ abdominal ventrite with two complete lateral striae, central portion of disk rather narrow, with a few small punctures near apical margin; propygidium with medium-sized round, shallow punctures concentrated in anterior two-thirds, separated by about their diameters; pygidium with fine, very dense ground punctation only, lacking coarser punctures; marginal pygidial sulcus complete, but very fine. Male genitalia more or less indistinguishable from those of *Operclipygus minutus* (see Figs 38A–D), showing the distinct desclerotization around the apical emargination of S8, but with S9 less distinctly desclerotized along midline.

#### Remarks.

**Figure 37.**
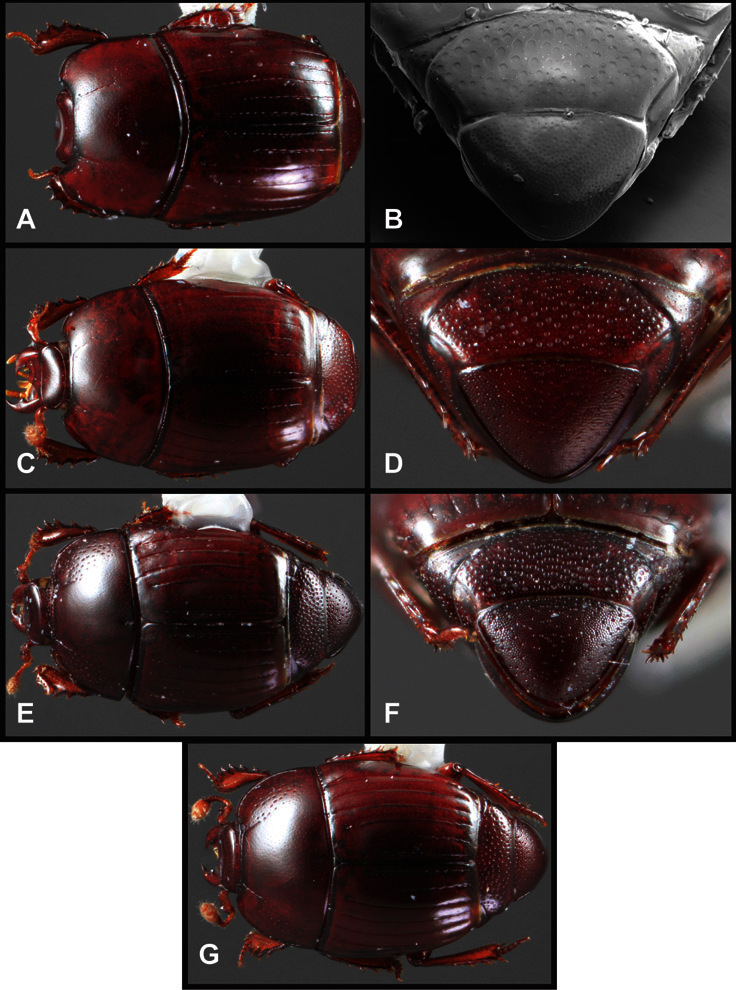
*Operclipygus hospes* group. **A** Dorsal habitus of *Operclipygus inquilinus* **B** Pygidia of *Operclipygus inquilinus* **C **Dorsal habitus of *Operclipygus communis* **D** Pygidia of *Operclipygus communis* **E** Dorsal habitus of *Operclipygus belemensis* **F** Pygidia of *Operclipygus belemensis* **G** Dorsal habitus of *Operclipygus innocuus*

Although in genitalic characters this species is very similar to *Operclipygus minutus*, and it may be related, it is highly distinctive in external characters. The complete lack of frontal stria, very fine or absent supraorbital stria, reduction (or loss) of lateral submarginal pronotal striae (Fig. 37A), very short prosternal striae, and the lack of coarser punctures on the densely granulose pygidium (Fig. 37B) diagnose this species easily.

#### Etymology.

This species’ name refers to its apparently myrmecophilus habits, which are infrequent in *Operclipygus*.

### Operclipygus tenuis sp. n.

urn:lsid:zoobank.org:act:5CC4E4A7-9F3C-4CBF-B9A5-55C432EBB5FA

http://species-id.net/wiki/Operclipygus_tenuis

Map 13

#### Type locality.

BRAZIL: Espirito Santo: Fazenda Lagoa do Macuco [19°3.833'S, 39°58.717'W].

#### Type material.

**Holotype male**: “**BRAZIL:** Espirito Santo: Mun. Linhares, Faz. Lagoa do Macuco, 19°03.833'S, 39°58.717'W 10m 27-I-2000 F. Genier & S. Ide ex. flight int. trap 3, day 1-3, primary lowland Atlantic forest, sandy soil BRA1G00 036”/ “SM0809949 KUNHM-ENT” (SEMC). **Paratypes** (2): **BRAZIL: Minas Gerais:** 1: Ingaí, Res. Boqueirão, nr. Lavras, 21°21'S, 44°59'W, 4–11.xii.2002, FIT, R.J. Silva (CEMT); **Rio de Janeiro:** 1: Nova Friburgo, Sans Souci, 9–15.xi.2009, FIT, E. Grossi (UFPR).

#### Diagnostic description.

**Figure 38.**
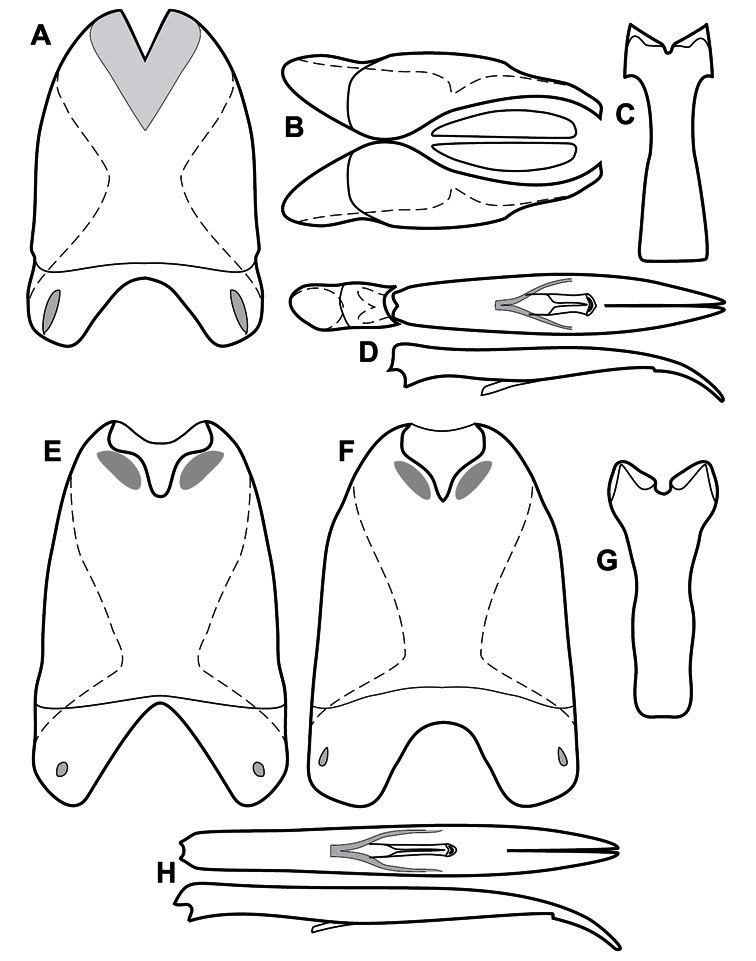
Male genitalia of *Operclipygus hospes* group. **A** T8 of *Operclipygus minutus* **B** T9 & T10 of *Operclipygus minutus* **C** S9 of *Operclipygus minutus* **D** Aedeagus, dorsal and lateral views, of *Operclipygus minutus* **E** T8 of *Operclipygus communis* **F** T8 of *Operclipygus belemensis* **G** S9 of *Operclipygus communis* **H** Aedeagus, dorsal and lateral views, of *Operclipygus communis*.

Length: 1.50–1.78 mm, width: 1.15–1.22 mm; body elongate, sides subparallel; frons depressed at middle; frontal stria with sides strongly divergent along inner edge of eye, arcuate at sides above antennal bases but slightly arched dorsad across middle; epistoma rather flat, coplanar with labrum, emarginate anteriorly; labrum flat, narrowed to apex, almost two-thirds as long as wide, with apical margin weakly rounded; left mandible with bluntly bifid basal tooth, right mandible with subacute tooth; pronotal disk with narrow, linear prescutellar impression slightly longer than scutellum, ground punctation very fine and sparse, with ~12 coarser punctures posterolaterad apices of anterior submarginal stria; lateral submarginal stria complete, close to side, bent inward rather abruptly in front, ending freely; anterior submarginal stria recurved obliquely posterad about one-fifth pronotal length; median pronotal gland openings next to anterior submarginal stria, about 6 puncture widths from anterior margin; elytra with outer subhumeral stria present in just over apical half, inner subhumeral stria absent, striae 1-4 complete, 5^th^ stria present in apical half, sutural stria present in apical three-fourths; prosternal keel narrowly rounded at base, carinal striae long, subparallel in anterior three-fourths, united in narrow arch, just divergent at base; stria of prosternal lobe interrupted at middle and shortened at sides; mesoventral margin narrowly emarginate, marginal stria complete; mesometaventral stria rather broadly arched forward to near mesoventral stria, mesoventral disk with conspicuous transverse microsculpture; lateral metaventral stria sinuate at sides, extending toward posterior corner of metacoxa; 1^st^ abdominal ventrite with two complete, parallel lateral striae, inner ones barely extending mediad along basal margin; propygidium with rather larger, shallow punctures separated by about two-thirds their diameters; pygidium with fine, dense ground punctation and coarser punctures fairly densely interspersed, particularly toward basal margin; marginal pygidial sulcus complete, narrow but deeply impressed, weakly crenulate along inner and outer edges. Male genitalia more or less indistinguishable from those of *Operclipygus minutus* (see Figs 38A–D), showing the distinct desclerotization around the apical emargination of T8; accessory sclerites of T8 somewhat smaller.

#### Remarks.

This species is very similar to *Operclipygus subterraneus*, but may be distinguished by its complete frontal stria, as well as its long, close and parallel prosternal striae.

#### Etymology.

This species’ name refers to its slender, elongate body form.

### Operclipygus communis sp. n.

urn:lsid:zoobank.org:act:BF339BE8-F045-4B8E-8CBC-613B3160FC24

http://species-id.net/wiki/Operclipygus_communis

**Map 14.**
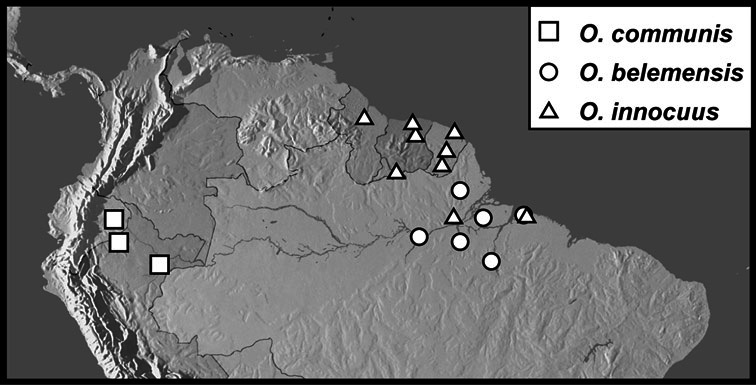
Records of the *Operclipygus hospes* group.

Figs 37C–D 38E, G–H Map 14

#### Type locality.

ECUADOR: Orellana: Yasuní Research Station [0°40.5'S, 76°24'W].

#### Type material.

**Holotype male**: “**Ecuador:** Napo, mid.Rio Tiputini, Yasuní Res. Stn. 0°40.5'S, 76°24'W, FIT#4. 20–29 Jun 1999. AKT#028 A.Tishechkin” / “LSAM 0013198” / “*Operclipygus* sp. #10, Hist 003 Yasuní NP Inventory, A.K.Tishechkin det. 2010” (FMNH). **Paratypes** (53): 4: same data as type (LSAM); same data as type, except as noted: 1: 22–28.vi.1999 (LSAM), 5: 23–30.vi.1999 (LSAM, MSCC, AKTCC, FMNH), 9: 28.vi–5.vii.1999 (LSAM, CHND), 1: 4–17.vii.1999 (LSAM), 1: 5–11.vii.1999 (LSAM), 2: 7–13.vii.1999 (LSAM), 2: 25.vii–4.viii.1999 (LSAM); 4: Yasuní Res. Stn., 00°40'28"S, 76°38'50"W, 215m, 5–10.ix.1999, FIT, primary forest, E.G. Riley (LSAM); 18: Parque Nac. Yasuní, Via Maxus at Puente Piraña, 0°39.5'S, 76°26'W, 14–20.vii.2008, FIT, A.K. Tishechkin (AKTC, MSCC, USFQ), 4: 20–24.vii.2008, FIT, A.K. Tishechkin (AKTC); 1: Res. Ethnica Waorani, 1km S Onkone Gare Camp, Trans. Ent., 0°39'10"S, 76°26'W, 220m, 23.i.1994, fogging, T.L. Erwin (USNM); 2: Tiputini Biodiversity Station, 0.6376°S, 76.1499°W, 2–9.vi.2011, FIT, M.S. Caterino & A.K. Tishechkin (USFQ, AKTC), 1: 5–25.ix.2000, D.J. Inward & K.A. Jackson (BMNH).

#### Other material.

**PERU: Loreto:** 1: Campamento San Jacinto, 2°18.75'S, 75°51.77'W, 175–215m, 7.vii.1993, FIT, R. Leschen (SEMC), 1: 5.vii.1993, FIT, R. Leschen (SEMC); 1: Teniente Lopez, 2°35.66'S, 76°06W, 210–240m, 24.vii.1993, FIT, R. Leschen (SEMC); 2: Iquitos, 90m, 8.v.1992, pitfall, J. Danoff-Berg (SEMC); 1: Iquitos, Jenaro Herrera, x-xi.1988, FIT, G. Couturier (CHND).

#### Diagnostic description.

Length: 1.68–1.81 mm, width: 1.37–1.47 mm; body rufescent, elongate oval; frontal stria complete, weakly displaced dorsad, divergent, rounded at sides, transverse across front, continuous with complete suborbital stria; epistoma not depressed in front of frontal stria, rather flat, weakly emarginate apically; labrum with upper edge of apical margin emarginate, but with small acute median tooth produced beneath; left mandible with bluntly bifid basal tooth, right mandible with subacute tooth; prescutellar impression linear, narrow, almost twice as long as scutellum; few (~12) coarser lateral pronotal punctures present; lateral submarginal pronotal stria abbreviated posteriorly; anterior submarginal pronotal stria recurved posterad about one-sixth pronotal length; median pronotal gland openings laterad base of anterior submarginal stria, about 4 puncture widths from anterior margin; elytra with outer subhumeral stria present in apical half, inner subhumeral stria absent, striae 1-4 complete (4^th^ sometimes barely abbreviated or fragmented at base), 5^th^ stria present in apical half or less, frequently fragmented, sutural stria present in apical three-fourths; prosternal keel weakly produced posteriorly, carinal striae united well short of presternal suture, united at base; mesoventral margin narrowly emarginate, marginal stria complete; mesometaventral stria narrowly arched forward to middle of mesoventral disk; lateral metaventral stria extending to near outer corner of metacoxa, frequently bent outward at apex; 1^st^ abdominal ventrite with two complete subparallel lateral striae; propygidium with very small, shallow punctures separated by slightly more than their diameters; pygidium with fine dense ground punctation, very slightly coarser punctures sparsely interspersed; marginal pygidial stria complete, fine but deep. Male genitalia (Figs 38E, G–H): accessory sclerites present, very small; T8 elongate, sides only slightly convergent in basal three-fourths, apex rounded, basal emargination deep, subangulate, nearly reaching basal membrane attachment line, apical emargination sinuate, with distinct bilateral sclerotizations on either side of narrow median part, ventrolateral apodemes most strongly developed near base, narrowing to apex; T9 (as in *Operclipygus hospes*) widest one-third from base, with subacute angle at this point, weakly narrowed to base, sinuately narrowed to apex, apices subtruncate, rather broad; T10 with halves separate along midline; S9 with thin sclerotization along midline, with sides sinuate, base rounded, lateral flanges wide, with distal corners prominent, apex with small median emargination and prominent, separate apical flanges; tegmen elongate, narrow, subcylindrical, parallel-sided in middle half, rather abruptly narrowed near base, thence parallel to base, apex subacute, curved downward in apical fourth, with subapical ventral cleft, medioventral process long, its apex narrowly truncate, projecting beneath about one-third from base; basal piece about one-fourth tegmen length; median lobe about one-fourth tegmen length, filamentous portions of proximal apodemes short and inconspicuous.

#### Remarks.

As mentioned above, this and the following two species form a distinctive subgroup based on male genitalia. While their aedeagi shows the truncate medioventral process and subapical cleft of the preceding species, the T8 has very small accessory sclerites, distinctive subapical sclerotizations (Figs 38E–F), and a well sclerotized spiculum gastrale. No obvious external characters unite them.

The strongly abbreviated lateral pronotal stria (Fig. 37C), subequal in length with the recurved anterior submarginal stria, along with the unusually small, sparse punctures of the propygidium (Fig. 37D), the complete frontal and supraorbital striae enclosing a rather evenly oval frons, and the very fine, complete marginal pygidial sulcus will allow recognition of *Operclipygus communis*. There is some variation in propygidial punctation among Peruvian specimens, so we exclude these from the type series.

#### Etymology.

This species’ name refers to its relative abundance at its type locality, among the most common *Operclipygus* in the Yasuní National Park area.

### Operclipygus belemensis sp. n.

urn:lsid:zoobank.org:act:46810614-6AC7-4E0C-B739-806CF46A4286

http://species-id.net/wiki/Operclipygus_belemensis

Figs 37E–F 38F Map 14

#### Type locality.

BRAZIL: Pará: Belém, Utinga [1°27'S, 48°26'W].

#### Type material.

**Holotype male**: “**BRAZIL: Pará:** Belém, Utinga (IPEAN), 1°27'S, 48°26'W. Piège d’interception. xi.1984” / “Caterino/Tishechkin Exosternini Voucher EXO-00295” (UFPR). **Paratypes** (7): 4: same data as type (FMNH, AKTC, MSCC); 3: same locality as type but xii.1984, FIT (MNHN, CHND).

#### Other material.

**BRAZIL: Amapá**: 1: Serra do Navio, 0°59'N, 52°00'W, 1–14.v.1991, FIT (CHND). **Pará:** 1:Tucuruí, 3°45'S, 49°40'W, iv.1986, FIT (CHND), 1: vi.1986, FIT (CHND), 1: 20.v–15.vi.1987, FIT (CHND), 1: 9–17.xii.1985, FIT (CHND); 1: Altamira - Marabá: km 18, 3°09'S, 52°03'W, v.1985, FIT (CHND); 6: Marajo-Breves, 0°53'S, 50°32'W, 18.xi–5.xii.1987, FIT (CHND); 2: Ilha Arapiuns, 2°24'S, 54°57'W, 30–31.xii.2008, FIT (CEMT).

#### Diagnostic description.

This species is very similar to the preceding, differing only in a few characters, as follows: length: 1.44–1.78 mm, width: 1.19–1.40 mm; prosternal carinal striae nearly reaching presternum (longer than in *Operclipygus communis*); 4^th^ dorsal stria more strongly impressed to base; 5^th^ stria longer, present in apical half, rarely fragmented; coarser pygidial punctures slightly larger and denser. Male: genitalia very similar to *Operclipygus communis* (Figs 38E, G–H), but with all parts more heavily sclerotized, especially T8; T8 (Fig. 38F) with greater distance between basal emargination and basal membrane attachment line, especially darkened in this area, basal apodemes of T8 broader, subtruncate apically, basal emargination shallower, more nearly rounded; all else as in preceding.

#### Remarks.

This species can be most easily separated from close relatives by the characters in the description, particularly the longer prosternal striae and coarser pygidial punctures (Fig. 37F), but should be dissected for unambiguous identification. We narrowly restrict the type series to the immediate type locality due to some variation among other specimens.

#### Etymology.

This species’ name refers to its primary occurrence in and near the city of Belém, Pará, Brazil.

### Operclipygus innocuus sp. n.

urn:lsid:zoobank.org:act:C99F16BE-1CAC-441A-B20B-3ABFFEE69331

http://species-id.net/wiki/Operclipygus_innocuus

Fig. 37G Map 14

#### Type locality.

SURINAME: Brokopondo:Ston Eiland Ecological Resort [4°59'N, 55°8'W].

#### Type material.

**Holotype male**: “**SURINAME: Brokopondo,** Ston Eiland Eco Resort nr. Brownsberg. 4°59.0'N, 55°8.0'W Flight intercepts. 10–13.ii.2010 C.Gillet, P.Skelley, W.Warner”/ “Caterino/Tishechkin Exosternini Voucher EXO-00297” (FSCA). **Paratypes** (3): **SURINAME: Para**: 2: nr. Overbridge River Resort, 5°31.8'N, 55°3.5'W, 15–18.ii.2010, FIT, C. Gillet, P. Skelley, W. Warner (FSCA, FMNH); 1: 05°31'10"N, 55°04'10"W, 10–14.ii.2010, FIT, W.B. Warner (WBWC).

#### Other material.

**BRAZIL: Pará:** 2: IPEAN, Belém, Utinga, 1°27'S, 48°26'W, ix.1985, FIT (CHND, AKTC), 1: viii.1985, FIT (CHND); 7: Monte Dourado, 1°11.35'S, 52°38.55'W, 220m, ix.2011, FIT, V. Korasaki (CEMT, UFPR, CHND). **FRENCH GUIANA:** 1: Matoury, 41.5km SSW on Hwy N2, 4°37'22"N, 52°22'35"W, 50m, 29.v–9.vi.1997, FIT, J. Ashe, R. Brooks (SEMC); 2: Régina, Réserve des Nouragues, 4°2.27'N, 52°40.35'W, 19.ii.2010, FIT, SEAG (CHND), 1: 3.xi.2009, FIT, SEAG (CHND), 5: 28.i.2010, FIT, SEAG (CHND, MNHN), 1: 20.vii.2009, Window trap, SEAG (CHND); 2: Roura, 27.4km SSE, 4°44'20"N, 52°13'25"W, 280m, 10.vi.1997, FIT, J. Ashe, R. Brooks (SEMC); 1: Belvèdére de Saül, point de vue, 3°1'22"N, 53°12'34"W, 10.xii.2010, FIT, SEAG (CHND), 1: 17.i.2011, FIT, SEAG (CHND), 1: 31.i.2011, FIT, SEAG (CHND); 2: Mont Tabulaire, Itoupé, 3°1.82'N, 53°6.40'W, 400m, 17.iii.2010, Window trap, SEAG (CHND), 1: 23.iii.2010, Window trap, SEAG (CHND). **GUYANA: Mazaruni Potaro**: 2: Takutu Mountains, 6°15'N, 58°58'W, 8.xii.1983, Window trap, montane rainforest near logging area, P.D. Perkins & W.E. Steiner (USNM); 7: 11.xii.1983, P.J. Spangler, R.A. Faitoute & W.E. Steiner (USNM). **SURINAME: Sipaliwini:** 8: CI-RAP Surv. Camp 3: Wehepai SE Kwamala, 2°21.776'N, 56°41.861'W, 237m, 3–7.ix.2010, FIT, T. Larsen & A.E.Z. Short (SEMC); 4: CI-RAP Surv. Camp2: Sipaliwini River, 2°10.521'N, 56°47.244'W, 210m, 27.viii–1.ix.2010, FIT, T. Larsen & A.E.Z. Short (SEMC, FMNH, MSCC).

#### Diagnostic description.

As with the preceding, this species is very similar to *Operclipygus communis*, differing only in a few characters, as follows: length: 1.53–1.68 mm, width: 1.25–1.34 mm; lateral submarginal pronotal stria present in basal two-thirds or more; 4^th^ and 5^th^ elytral striae more strongly impressed, not fragmented. Male: genitalia indistinguishable from those of *Operclipygus belemensis* (see Figs 38E, G–H).

#### Remarks.

This species has little to distinguish it from the preceding two beyond the longer lateral submarginal pronotal stria (Fig. 37G). This character is, however, very consistent over a wide geographic range. The type series is limited to those specimens from northern Suriname.

#### Etymology.

This species’ name means ‘harmless’.

### Operclipygus diffluens sp. n.

urn:lsid:zoobank.org:act:231649CD-EABC-40DE-847A-2C9BCFFB5E38

http://species-id.net/wiki/Operclipygus_diffluens

**Figure 39.**
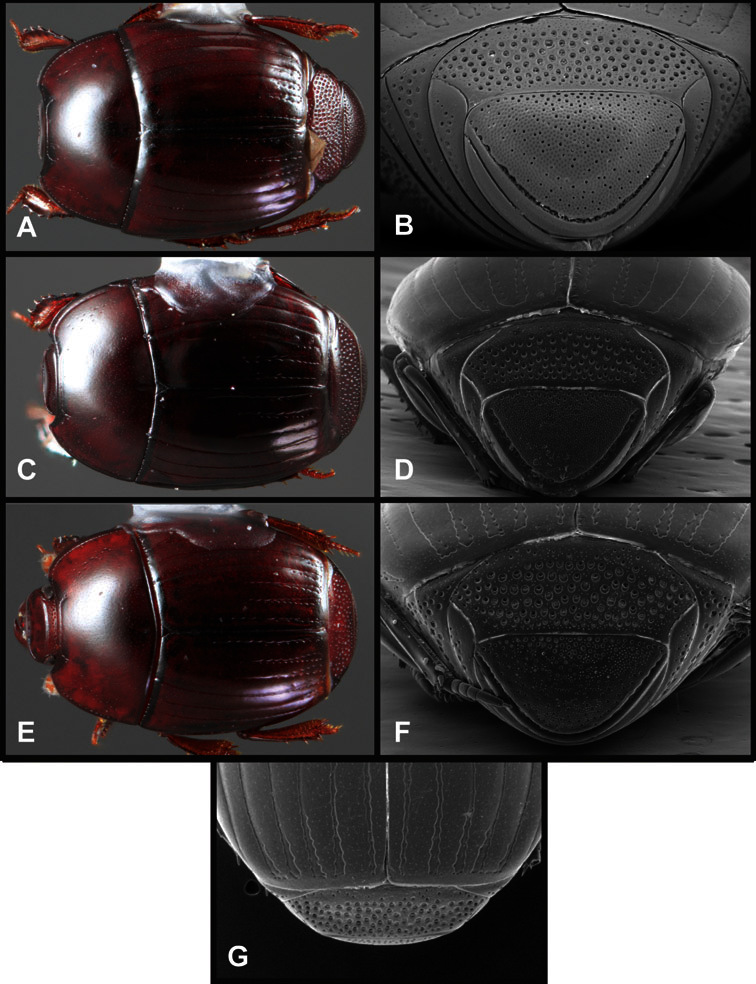
*Operclipygus hospes* group. **A** Dorsal habitus of *Operclipygus diffluens* **B** Pygidia of *Operclipygus diffluens* **C** Dorsal habitus of *Operclipygus confluens* **D** Pygidia of *Operclipygus confluens* **E** Dorsal habitus of *Operclipygus fusistrius* **F** Pygidia of *Operclipygus fusistrius* **G** Elytral striae of *Operclipygus fusistrius*.

**Map 15.**
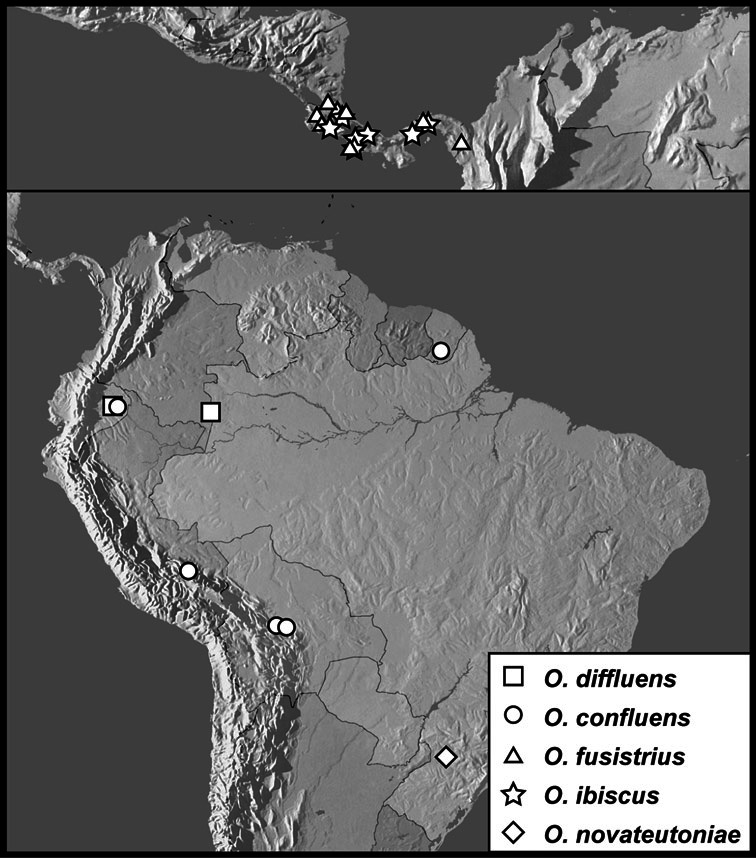
Records of the *Operclipygus hospes* group.

Figs 39A–B 40A–D, I Map 15

#### Type locality.

ECUADOR: Orellana: Yasuní Research Station [0°40.5'S, 76°24'W].

#### Type material.

**Holotype male**: “**Ecuador:** Napo, mid.Rio Tiputini, Yasuní Res. Stn. 0°40.5'S, 76°24'W, FIT#2. 25–30 July 1999. AKT#108 A.Tishechkin” / “LSAM 0013287” / “Operclipygus sp. #16, Hist 137 Yasuní NP Inventory, A.K.Tishechkin det. 2010” (FMNH). **Paratypes** (3): same data as type, except as noted: 1: 17–23.vi.1999, FIT, C.E. Carlton & A.K. Tishechkin (LSAM); 1: 18–25.vii.1999, FIT, A.K. Tishechkin (LSAM); 1: 23–30.vi.1999, FIT, C.E. Carlton & A.K. Tishechkin (MSCC).

#### Other material.

**COLOMBIA: Vaupés:** 1: Est. Biol. Caparú, Rio Apoporis, 1.1°S, 69.5°W, 27.ix–1.xii.1995, FIT, Black-water terrace forest on sandy soils, B.D. Gill (BDGC).

#### Diagnostic description.

**Figure 40.**
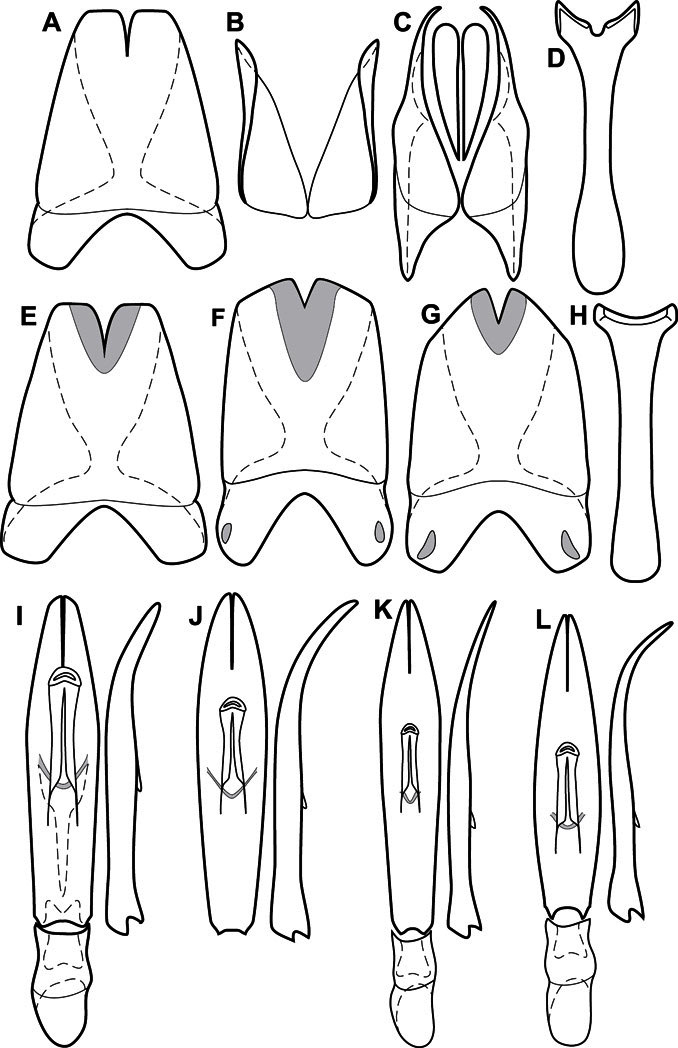
Male genitalia of *Operclipygus hospes* group. **A** T8 of *Operclipygus diffluens* **B** S8 of *Operclipygus diffluens* **C** T9 & T10 of *Operclipygus diffluens* **D** S9 of *Operclipygus diffluens* **E** T8 of *Operclipygus fusistrius* **F** T8 of *Operclipygus ibiscus* **G** T8 of *Operclipygus novateutoniae* **H** S9 of *Operclipygus novateutoniae* **I** Aedeagus, dorsal and lateral views, of *Operclipygus diffluens* **J** Aedeagus, dorsal and lateral views, of *Operclipygus confluens* **K** Aedeagus, dorsal and lateral views, of *Operclipygus ibiscus* **L** Aedeagus, dorsal and lateral views, of *Operclipygus novateutoniae*.

Length: 1.44–1.75 mm, width: 1.25–1.47 mm; body rufobrunneus, elongate oval, moderately convex, widest behind humeri; frontal stria complete, more or less evenly rounded from sides to front; epistoma convex, shallowly emarginate at apex; labrum about half as long as wide, sides rounded, apex slightly asymmetrical, with left side produced more than right; left mandible with blunt basal tooth, right mandible with small subacute basal tooth; pronotum depressed across base; prescutellar impression short, linear, length about equal to that of scutellum; lateral submarginal pronotal stria complete, marginal bead relatively wide; anterior submarginal pronotal stria barely recurved at apices; pronotal disk with few, ~6, very faint coarse lateral punctures; elytra with outer subhumeral stria complete, inner subhumeral stria absent, striae 1-3 complete, striae 3-5 abruptly more broadly impressed at apices; prosternal carinal striae united very close to presternum; lateral metaventral stria extending obliquely toward posterior third of metepisternum, abbreviated at apex; 1^st^ abdominal ventrite with two complete, subparallel lateral striae, disk lacking any median punctures; propygidium densely and uniformly convered with medium sized punctures; pygidium with fine dense ground punctation and slightly coarser punctures uniformly interspersed, separated by about 2× their diameters; marginal pygidial sulcus finely but deeply impressed, weakly crenulate on edges. Male genitalia (Figs 40A–D, I): accessory sclerites absent; T8 with sides convergent, basal apodemes rather short, basal emargination broad, shallow, nearly meeting basal membrane attachment line; S8 atypical for *hospes* group, sides subparallel, apical guides narrow, gradually widening to apex, ventral edges strongly divergent from base; 9T with sides subparallel in basal two-thirds, curving inward to narrow, subacute apices; T10 halves separate; S9 elongate, stem narrow for much of length, abruptly widened to rounded base; apex with narrow median emargination, apical flanges small, separate; tegmen widest about two-thirds from base, narrowed to rounded apex, medioventral process wide, rounded, projecting beneath near tegmen midpoint; basal piece about one-third tegmen length; median lobe about half tegmen length, filamentous portions of proximal apodemes short, inconspicuous.

#### Remarks.

This species and the four that follow are characterized by having the medioventral aedeagal process broadly rounded (Figs 40I–L), neither acute nor truncate, and the tegmen lacking a subapicoventral cleft. None of these species have their 8^th^ sternite broadly rounded at the base (Fig. 40B). Externally, most of the species have a complete outer subhumeral stria and expanded apices of elytral striae 2–4 (Fig. 39G). The last of these species, *Operclipygus novateutoniae* is an outlier in both of these respects, but its male genitalia associates it strongly with the other four.

The combination of apically expanded elytral striae (Fig. 39A), lack of coarse lateral pronotal punctures, dense propygidial punctation (Fig. 39B), and complete outer subhumeral elytral stria will distinguish *Operclipygus diffluens* from the following.

#### Etymology.

This species is named mainly as a counterpoint to the following, in which the 4^th^ and 5^th^ elytral striae are confluent apically. In *Operclipygus diffluens* they are separate. We restrict the type series to specimens from eastern Ecuador.

### Operclipygus confluens sp. n.

urn:lsid:zoobank.org:act:0F0B2842-AC37-407B-B6FA-33D79519FC59

http://species-id.net/wiki/Operclipygus_confluens

Figs 39C–D 40J Map 15

#### Type locality.

BOLIVIA: Cochabamba: 117 km E Yungas on Cochabamba – Villa Tunari Road [17°6.5'S, 65°41.2'W].

#### Type material.

**Holotype male**: “BOLIVIA: Cochabamba, Cochabamba, 117km E Yungas, (Cochabamba – Villa Tunari Rd.), 1040m 17°6'32"S, 65°41'12"W, 1–6 FEB 1999; R.Hanley BOL1H99 028, ex: flight intercept trap”/ “SM0160002 KUNHM-ENT” (SEMC). **Paratypes** (18): 3: same data as type (FMNH, SEMC); same data as type, except as noted: 3: 6–8.ii.1999, FIT, montane evergreen forest, F. Genier (CMNC, MSCC), 1: 8–12.ii.1999, FIT, montane evergreen forest, F. Genier (CMNC); **BOLIVIA: Cochabamba:** 2: Est. Biol. Valle Sajta, Univ. San Simon, 67.5km E Villa Tunari, 17°06'19"S, 64°46'57"W, 300m, 7–9.ii.1999, FIT, lowland rain forest, F. Genier (CMNC), 7: 9–13.ii.1999, FIT, lowland rain forest, F. Genier (CMNC, MSCC, AKTC); 2: 117km E Cochabamba, Yungas, Cochabamba-Villa Tunari Rd., 17°6'32"S, 65°41'12"W, 1040m, 1–6.ii.1999, FIT, yungas forest, R. Hanley (SEMC).

#### Other material.

**ECUADOR: Orellana:** 1:Tiputini Biodiversity Station, 0.6376°S, 76.1499°W, 4–9.vi.2011, FIT, M.S. Caterino & A.K. Tishechkin, DNA Extract MSC-2182 (SBMNH). **FRENCH GUIANA:** 1: Mont Tabulaire, Itoupé, 3°1.32'N, 53°5.05'W, 800m, 17.iii.2010, FIT, SEAG (CHND). 3: **PERU: Cusco:** Cock of the Rock Lodge, NE Paucartambo, 13°03.3'S, 71°32.7'W, 1120m, 4–9.xi.2007, FIT, D. Brzoska (SEMC).

#### Diagnostic description.

This species is very similar to the preceding, differing as follows: length: 1.68–1.97 mm, width: 1.37–1.62 mm; body larger, broader; 5^th^ and sutural elytral striae slightly longer and more broadly impressed; elytral striae 3-5 expanded at apex, striae 4-5 usually joined in apical arch; mesoventrite less deeply emarginate at middle; lateral metaventral stria extended more posteriorly; lateral striae of 1^st^ abdominal ventrite closer to each other, subparallel; pygidial disk with coarser punctures sparser though slightly larger at middle; marginal pygidial sulcus deeper and coarser. Male: basal genitalic segments identical to those of *Operclipygus diffluens* (see Figs 40A–D); tegmen (Fig. 40J) shorter, sides subparallel in basal three-fourths, rounded to wider apex; basal piece shorter, less than one-third tegmen length.

#### Remarks.

This species is very similar to several other *Operclipygus hospes* group species, especially *Operclipygus diffluens* and *Operclipygus ibiscus*. However, it may be distinguished by its much deeper and more strongly crenulate pygidial sulcus (Fig. 39D). In most individuals the expansions of the apices of elytral striae 4 and 5 merge (Figs 39C–D), forming a distinctive apical arch joining them. We restrict the type series to specimens from Cochabamba, as there is some variation in propygidial sculpturing among localities.

#### Etymology.

The name of this species refers to the unusual tendency for the apices of the 4^th^ and 5^th^ elytral striae to merge.

### Operclipygus fusistrius sp. n.

urn:lsid:zoobank.org:act:861C1389-BA76-4D4D-BE6B-5387E6479768

http://species-id.net/wiki/Operclipygus_fusistrius

Figs 39E–G 40E Map 15

#### Type locality.

COSTA RICA: Heredia: La Selva Biological Station 10°26'N, 84°1'W].

#### Type material.

**Holotype male**: “**COSTA RICA:** Heredia, Est.Biol. LaSelva. 10.26°N, 84.01°W. F.I.T. 23 June 1998 C.Carlton & A.Tishechkin, leg.”/ “LSAM0046456” (FMNH). **Paratypes**: (20): 1: same data as type; same data as type, except as noted: 1: 19.vi.1998, (LSAM), 2: 21.vi.1998 (LSAM, FMNH), 1: 22.vi.1998 (LSAM), 1: 25.vi.1998 (LSAM), 1: 27.vi.1998 (LSAM), 3: 29.vi.1998 (LSAM, MSCC, AKTC), 1: 5–8.vi.2001, FIT, S. Chatzimanolis (SEMC), 1: 2–5.vi.2001, FIT, S. Chatzimanolis (SEMC), 1: 11–14.vi.2001, FIT, S. Chatzimanolis (CMNC), 2: 28.ii.1992, FIT, W. Bell (SEMC), 1: 3.iii.1992, FIT, W. Bell (SEMC), 2: 5–8.iii.2001, FIT, E.G. Riley (TAMU), 1: 21–28.iii.1988, W.E. Steiner, J.M. Hill, J.M. Swearingen, J.M. Mitchell (USNM); 1: 10kmW. Puerto Viejo, 170m, 2–5.iii.1991, FIT, H. & A. Howden (CHSM).

#### Other material.

**COSTA RICA: Heredia:** 1: Parque Nac. Braulio Carrillo, Est. Magsasay, 264600, 531100, 200m, i.1991, M. Barrelier, (INBIO); **Cartago:** 1: Parque Nac. Barbilla, Turrialba, 2km despues del Rio Dantas, 400m, 17–28.xi.2000, FIT, W. Arana (INBIO); **Guanacaste:** 1: Parque Nac. Guanacaste, 9km S Santa Cecilia, Est. Pitilla, 700m, vii.1995, FIT, C. Moraga, P. Rios, (INBIO), 1: 27.vii-14.viii.1992, C. Moraga, (INBIO); **Limón:** 1: Area Cons. Tortuguero, Sector Cerro Cocori, Fca. de E. Rojas, 150m, v.1991, E. Rojas (INBIO), 1: vi.1991, E. Rojas (INBIO), 1: vii.1991, E. Rojas (INBIO), 1: xii.1992, E. Rojas (INBIO), 1: iv.1993, E. Rojas (INBIO), 1: v.1993, E. Rojas (INBIO), 1: vi.1993, E. Rojas (INBIO), 1: Oeste de R.B. Hitoy Cerere, Finca el Tucan, 100–200m, 14–21.viii.2001, FIT, W. Arana (INBIO); **Puntarenas:** 1: Rancho Quemado, Peninsula de Osa, 200m, xii.1991, F. Quesada (INBIO), 1: vi.1992, F. Quesada (INBIO), 2: ix.1992, F. Quesada (INBIO), 1: x.1992, F. Quesada (INBIO); 1: Res. Biol. Carara, Est. Quebrada Bonita, 50m, vi.1993, R. Guzman (INBIO), 1: v.1994, J. Saborio (INBIO), 1: xi.1994, R. Guzman (INBIO); 1: Golfito, Est. Agujas, Sendero Zamia, 300m, 21–30.vi.2000, A. Azofeifa (INBIO); 1: Est. Esquinas, Peninsula de Osa, x.1993, M. Segura (INBIO); 1: Parque Nac. Corcovado, Est Agujas, Golfito, C. Rincon, 745m, 12.ii.2000, FIT, A. Azofeifa (INBIO). **NICARAGUA: Rio San Juan**: 1: env. of Ref. Bartola, El Castillo, 22–25.ii.2000, FIT, secondary rainforest, Barbero E. & Penati F. (CHFP); 1: Refugio Bartola, 60km SE San Carlos, 10°58.40'N, 84°20.30'W, 100m, 28–30.v.2002, FIT, R. Brooks, Z. Falin, S. Chatzimanolis (SEMC). **PANAMA: Colón:** 1: San Lorenzo Forest, STRI crane site, 9°17'N, 79°58'W, 24–25.v.2004, FIT, A.K. Tishechkin (GBFM); 1: Parque. Nac. Soberania, Pipeline Rd., 09°07'N, 79°45'W, 27.v.1995, FIT, J. Jolly, C. Chaboo (SEMC); **Darién:** 1: Cana Biological Station, 7°45'18"N, 77°41'6"W, 550m, 3–7.vi.1996, FIT, J. Ashe, R. Brooks (SEMC); **Panamá**: 1: Old Plantation Rd. 6.9km S Gamboa, 09°05'N, 79°40'W, 80m, 7–22.vi.1995, FIT, J. Ashe, R. Brooks (SEMC); 1: Nusagandi Sta., Ina Igar Trail, 09°21'N, 78°59'W, 12.v.1994, FIT, D. Windsor (SEMC).

#### Diagnostic description.

This species is very similar to the preceding, differing in only a few characters, as follows: length: 1.44–1.68 mm, width: 1.19–1.34 mm; frontal stria with central portion straighter, more transverse; coarser lateral pronotal punctures more numerous and conspicuous; lateral metaventral stria directed more posteriorly; mesoventrite and 1^st^ abdominal ventrite with conspicuous transverse waves of microsculpture; propygidium with secondary punctures larger, shallower, slightly sparser, separated by almost half their diameters; pygidium appearing shorter, partly due to more deeply impressed apical marginal sulcus; secondary pygidial punctures similarly coarse, but tending to be more concentrated toward basal margin. Male genitalia: very similar to *Operclipygus diffluens* (see Figs 40A–D, I), but T8 (Fig. 40E) with faint submarginal desclerotization around apical emargination.

#### Remarks.

*Operclipygus fusistrius* and the following two species exhibit a broad, desclerotized margin around the apical emargination of T8 (Fig. 40E), an apparently strong synapomorphy, although no consistent external characters unite all of them. The combination of this type of 8^th^ tergite, the presence of a few lateral pronotal punctures (Fig. 39E), and uniformly convex frons will distinguish *Operclipygus fusistrius* from the other two.

#### Etymology.

The name of this species refers to its apically widened elytral striae.

### Operclipygus ibiscus sp. n.

urn:lsid:zoobank.org:act:BF51784E-E209-4038-B318-F88FEC9130B0

http://species-id.net/wiki/Operclipygus_ibiscus

Figs 40F, K 41A–C Map 15

#### Type locality.

PANAMA: Colón: San Lorenzo Forest [9°17'N, 79°58'W].

#### Type material.

**Holotype male**: “**PANAMA**: Colón Pr., San Lorenzo Forest. 9°17'N, 79°58'W. Flight Intercept FIT-C3-12 12-13 May 2004 A. Tishechkin. IBISCA ‘04” / “LSAM 0111298” (FMNH). **Paratypes**: (103): same data as type, except as noted: 1: 23–24.ix.2003 (LSAM), 3: 27–28.ix.2003 (LSAM, FMNH), 2: 30–1.x.2003 (LSAM), 1: 1–3.x.2003 (LSAM), 4: 4–6.x.2003 (LSAM, MSCC, AKTC), 2: 8–9.x.2003 (LSAM, CHND), 1: 9–11.x.2003 (LSAM), 3: 13–14.x.2003 (LSAM, CMNC), 4: 14–17.x.2003 (LSAM), 3: 17–18.x.2003 (LSAM), 3: 17.x.2003, R. Didham, L. Fagan (LSAM), 1: 18–21.x.2003 (LSAM), 6: 19–21.x.2003 (LSAM), 4: 21–22.x.2003 (LSAM), 1: 24.x.2003, in rotting fruit of Bombacaceae tree, L. Cizek (LSAM), 1: 25–26.x.2003 (LSAM), 1: 28–30.x.2003 (LSAM), 4: 29.x.2003, R. Didham, L. Fagan (GBFM, LSAM), 3: 11–12.v.2004 (LSAM), 2: 12–13.v.2004 (LSAM), 2: 14–15.v.2004 (LSAM), 6: 15–17.v.2004 (GBFM, LSAM), 1: 15–25.v.2004, R. Didham (LSAM), 6: 17–18.v.2004 (LSAM), 4: 18–19.v.2004 (LSAM), 3: 19–20.v.2004 (LSAM), 6: 20–21.v.2004 (LSAM), 9: 21–24.v.2004 (LSAM), 2: 24–25.v.2004 (LSAM), 1: 25.v–5.vi.2004, R. Didham (LSAM) , 8: 25–26.v.2004 (GBFM, LSAM), 1: 26–28.v.2004 (LSAM), 3: 26–29.v.2004 (LSAM), 1: 4–17.vi.2004, M. Rapp (LSAM).

#### Other material.

**PANAMA: Colón:** 1: Parque. Nac. Soberania, Pipeline Rd. Km 6.1, 09°07'N, 79°45'W, 40m, 7–21.vi.1995, FIT, J. Ashe, R. Brooks (SEMC); **Bocas del Toro**: 1: Almirante, 23.iii.1959, decayed fibrous palm trunk, H.S. Dybas (FMNH); 1: Almirante, 1.iv.1959, forest litter, H.S. Dybas (FMNH); **Chiriquí**: 2: 4km N Sta. Clara Hartmann's Finca, 1500m, 30.vi–13.vii.1982, FIT, B.D. Gill (BDGC); **Coclé**: 3: 7.2km NE. El Cope, 08°37'N, 80°35'W, 730m, 20.v–7.vi.1995, FIT, J. Ashe, R. Brooks (SEMC); **Panamá**: 1: Barro Colorado Isl., 09°11'N, 79°51'W, 8.vii.1994, FIT, D. Banks (SEMC), 1: 1.viii.1994, FIT, D. Banks (SEMC), 1: 24.viii.1994, FIT, D. Banks (SEMC), 1: 16.ii.1976, *Pseudobombax* flower fall, A.F. Newton (FMNH), 1: 1–5.vii.2000, FIT, S. Chatzimanolis (SEMC),1: 22–25.vi.2000, FIT, S. Chatzimanolis (SEMC), 1: i–iii.1944 (FMNH); 7: Chepo-Carti Rd., 400m, vi.1982, FIT, B.D. Gill (BDGC, AKTC), 1: vii.1982, FIT, B.D. Gill (CHSM); 8: Cerro Azul, ca., 2000ft, 21.ii.1976, flood debris, A.F. Newton (FMNH); 1: Nusagandi, Ina Igar trail, 18–21.v.1993, FIT/pitfall, primary forest, E.G. Riley (TAMU); 1: Old Plantation Rd. 6.9km S Gamboa, 09°05'N, 79°40'W, 80m, 7–22.vi.1995, FIT, J. Ashe, R. Brooks (SEMC). **COSTA RICA: Heredia:** 2: La Selva Biol. Stn., 10°26'N, 84°01'W, 21.vi.1998, FIT, C.E. Carlton & A.K. Tishechkin (LSAM) , 3: 22.vi.1998 (LSAM) , 3: 23.vi.1998 (LSAM) , 2: 24.vi.1998 (LSAM) , 2: 25.vi.1998 (LSAM) , 5: 26.vi.1998 (LSAM) , 5: 27.vi.1998 (LSAM) , 1: 28.vi.1998 (LSAM) , 4: 29.vi.1998 (LSAM) , 1: 24.ii.1992, FIT, W. Bell (SEMC), 1: 16.vi.2012, OTS Beetle Course, DNA Extract MSC-2307 (SBMNH), 1: 19.iii.1992, FIT, W. Bell (SEMC), 1: 6.vii.2005, FIT, M. Ferro (AKTC), 1: 8.vii.2005, FIT, M. Ferro (AKTC); 1: 10kmW. Puerto Viejo, 170m, 2–5.iii.1991, FIT, H. & A. Howden (CHSM); **Limón:** 3: Area Cons. Tortuguero, Sector Cerro Cocori, Fca. de E. Rojas, 150m, ii.1993, E. Rojas (INBIO), 1: v.1991, E. Rojas (INBIO), 6: vi.1991, E. Rojas (INBIO), 1: ix.1991, E. Rojas (INBIO), 2: x.1991, E. Rojas (INBIO), 1: iii.1992, E. Rojas (INBIO), 6: iv.1992, E. Rojas (INBIO), 3: iv.1993, E. Rojas (INBIO), 2: ix.1993, E. Rojas (INBIO), 1: v.1993, E. Rojas (INBIO), 2: xii.1992, E. Rojas (INBIO), 3: 26.vii-2.viii.1992, E. Rojas (INBIO), 1: 9–30.xi.1992, E. Rojas (INBIO); 1: Area Cons. Tortuguero, Rio Sardinas, R.N.F.S. Barra del Colorado, 50m, v.1994, F.V. Araya (INBIO), 1: 10m, 1–14.ii.1994, F.V. Araya (INBIO); 1: Sardinas, Barra del Colorado, 15m, 28.i–12.ii.1995, F. Araya, (INBIO), 2: 26.iv–3.v.1995, F. Araya, (INBIO), 2: 4–11.i.1995, F. Araya, (INBIO); 2: 3km N. del Puente Rio Suerte, Ruta Puerto Lindo, Cedrales de la Rita, 10m, viii.1996, FIT, E. Rojas (INBIO); 1: Amubri, 70m, 1–22.x.1994, G. Gallardo, (INBIO); **Puntarenas:** 1: Res. Biol. Carara, Est. Quebrada Bonita, 100m, ii.1995, R. Guzman, (INBIO), 1: 50m, iv.1994, R. Guzman, (INBIO); 1: Rancho Quemado, Peninsula de Osa, 200m, ix.1991, F. Quesada, (INBIO), 1: viii.1991, F. Quesada, (INBIO), 2: ix.1992, F. Quesada, (INBIO), 2: v.1992, F. Quesada y G. Varela, (INBIO), 2: vi.1992, F. Quesada, (INBIO), 1: vii.1992, M. Segura, (INBIO), 1: 12.v.1993, 24.v.1993, A. Gutierrez, (INBIO); 1: Est. Esquinas, Peninsula de Osa, x.1993, M. Segura, (INBIO); 1: Bosque Esquinas, Peninsula de Osa, 200m, vi.1994, M. Segura, (INBIO); 1: Reserva Forestal Golfo Dulce, Est. Agujas, Golfito, Sendero Zamia, 250–350m, 5–7.xi.1999, FIT, A. Azofeifa (INBIO); 1: Est. Biol. Las Cruces, Coto Brus, 8°47'N, 82°57'W, 1100m, 22–23.iii.2002, FIT, A.K. Tishechkin (LSAM), 1: 1100m, 2–8.iv.2002, FIT, A.K. Tishechkin (LSAM), 1: 1100m, 31.iii-1.iv.2002, FIT, A.K. Tishechkin (LSAM), 1: 1330m, 28–31.v.2004, FIT, J.S. Ashe, Z. Falin, I. Hinojosa (SEMC), 1: 1330m, 28–30.v.2004, FIT, J.S. Ashe, Z. Falin, I. Hinojosa (SEMC); 1: Est. Biol. Las Cruces, San Vito, 8°46'N, 82°58'W, 4000ft, 19.iii.1973, Berlese, stream bed litter, Virgin forest, J. Wagner, J. Kethley (FMNH), 7: 17.viii-12.ix.1982, FIT, B.D. Gill (AKTC), 2: 1200m, 1–30.vii.1982, B.D. Gill (BDGC); 2: Est. Biol. Las Alturas, Coto Brus, 8°57'N, 82°52'W, 1550m, 30.iii-4.iv.2002, FIT, A.K. Tishechkin (LSAM), 1: 25–30.iii.2002, FIT, A. Cline & A. Tishechkin (LSAM); 1: Est. F.N. Aguas Buenas, Rincon, 7km W. Osa Penn., 50m, 21–25.vi.1997, FIT, S. & J. Peck (SEMC); 1: Estrella Valley, Pandora, 1–16.iii.1984, FIT, H. Howden & G. Manley (CHSM).

#### Diagnostic description.

This species is very similar to the preceding, differing in the following characters: length: 1.53–1.78 mm, width: 1.25–1.44 mm; frontal stria complete, rather evenly rounded from sides to front; lateral pronotal complete; pronotum finely punctate, lacking any larger lateral punctures; pronotum depressed, but lacking distinct antescutellar impression; lateral metaventral stria extending obliquely laterad just behind postmesocoxal stria; 1^st^ abdominal ventrite with the two lateral striae fine and very close at base, diverging posteriorly, outer stria frequently abbreviated or fragmented; outer subhumeral stria complete, fine, occasionally fragmented basally, elytral striae 1-3 complete , 4^th^ and 5^th^ striae present in apical third, sutural stria present in apical two-thirds; elytral striae 3-5 weakly expanded at apices; marginal pygidial sulcus deep, well developed, somewhat crenulate; pygidium with fine, dense ground punctation interspersed with sparse, slightly coarser secondary punctures. Male genitalia similar in most respects to those of *Operclipygus fusistrius* and *Operclipygus diffluens* (see Figs 40B–D), but T8 (Fig. 40F) with accessory sclerites present, submarginal desclerotization of T8 extending to near midpoint of segment, and tegmen (Fig. 40K) narrower and more elongate.

#### Remarks.

**Figure 41.**
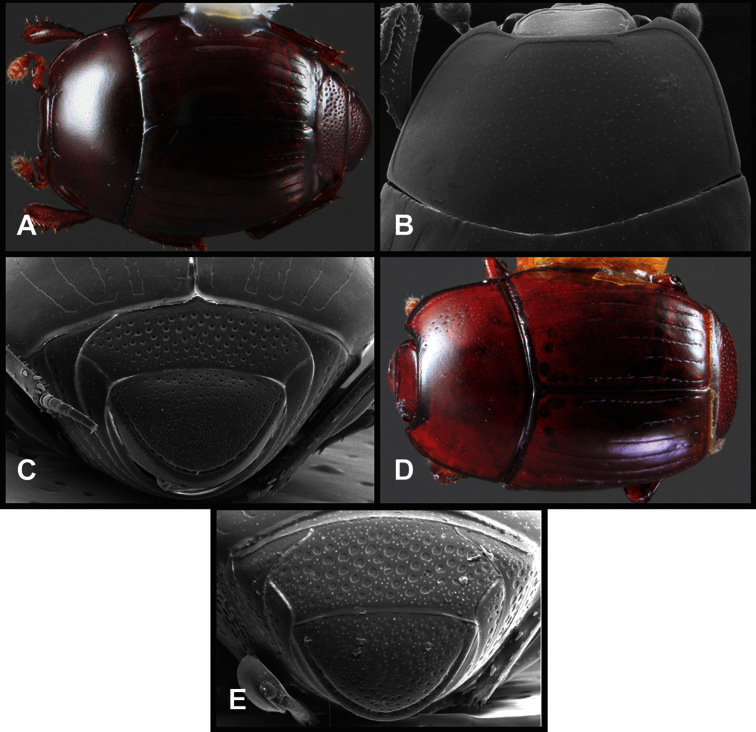
*Operclipygus hospes* group. **A** Dorsal habitus of *Operclipygus ibiscus* **B** Pronotum of *Operclipygus ibiscus* **C** Pygidia of *Operclipygus ibiscus* **D** Dorsal habitus of *Operclipygus novateutoniae* **E** Pygidia of *Operclipygus novateutoniae*.

Among the species that exhibit a subapical desclerotization along the apical emargination of the male 8^th^ tergite (Fig. 40F), *Operclipygus ibiscus* and *Operclipygus novateutoniae* are unusual in having a complete outer subhumeral stria. In addition, the absence of coarse punctures on the sides of the pronotum (Fig. 41B), weakly apically widened elytral striae (Fig. 41A), and very closely-set lateral striae of abdominal ventrite 1 will distinguish *Operclipygus ibiscus*. *Operclipygus fusistrius* is also very similar, and has a similar distribution, but has more distinctly expanded elytral striae and clearly separated lateral striae on ventrite 1. Because there is slight variation throughout the range of this species in propygidial punctation and degree of elytral stria widening, we restrict the type series to specimens from the type locality.

#### Etymology.

This species’ name recognizes the IBISCA (Investigating the Biodiversity of Soil and Canopy Arthropods) project, whose 2003–04 Panamanian survey yielded the type specimens of this species.

### Operclipygus novateutoniae sp. n.

urn:lsid:zoobank.org:act:1A775831-1639-4CB7-A6BD-EAD3A971F840

http://species-id.net/wiki/Operclipygus_novateutoniae

Figs 40 G–H, L 41D–E Map 15

#### Type locality.

BRAZIL: Santa Catarina: Nova Teutonia [27°11'S, 52°23'W].

#### Type material.

**Holotype male**: “Nova Teutonia, Sta. Catharina, BRAZ. X:20:1959 Fritz Plaumann leg.”/ “FMNH-INS 0000069171” (FMNH). **Paratypes** (3): same data as type, except as noted: 1: 13.v.1951, with *Acromyrmex* (FMNH), 1: xi.25.1953 (FMNH), 1: xi.20.1958 Berlese floor litter (UFPR).

#### Diagnostic description.

Length: 1.59–1.87 mm, width: 1.22–1.44 mm; body rufobrunneus, elongate oval; frontal stria with central portion connected to sides, barely arcuate, transverse; anterior submarginal pronotal stria barely recurved posterad; pronotal disk with ~20 fairly conspicuous coarse lateral punctures; elytra with outer subhumeral stria present only in apical half, inner subhumeral stria absent, striae 1-3 complete, 4^th^ stria present in apical half to two-thirds, 5^th^ stria present in apical half, sutural stria present in apical three-fourths; elytral striae 3-5 not expanded apically; mesoventral disk and 1^st^ abdominal ventrite lacking microsculpture; lateral metaventral stria extending posterolaterad toward outer corner of metacoxa, not appreciably curved; 1^st^ abdominal ventrite with two lateral striae moderately close, subparallel in basal half, diverging in posterior half, inner turned posterad, outer turned laterad, abbreviated, disk with fine series of small punctures along posterior margin; ventrites 2-4 similar but with larger punctures; propygidium with small round punctures sparsely but uniformly separated by nearly their diameters; pygidium with ground punctation less dense than that of propygidium, with shining impunctate pygidial surface visible between, slightly coarser punctures uniformly interspersed, not concentrated along basal margin; marginal sulcus of pygidium complete, deeply impressed, crenulate along outer margin, slightly removed from margin at middle. Male genitalia (Figs 40G–H, L): similar in many respects to those of *Operclipygus ibiscus*, with accessory sclerites present, T8 submarginal desclerotization shallow, distinct, sides of S8 straight, convergent, with narrow apical guides; differing in having S9 elongate, with more distinct median sclerotization, lacking apical emargination, apical flange uninterrupted; tegmen with sides rounded, widest near middle, narrowed evenly to base and apex, medioventral process short, rounded, projecting beneath about one-third from base, apical half of tegmen with strong ventral curvature.

#### Remarks.

The shortened 4^th^ dorsal stria (Fig. 41D), frons with evenly arcuate frontal stria, and deeply impressed pygidial sulcus (Fig. 41E) which is slightly removed from the margin at middle will distinguish this species from the preceding two genitalically similar species.

#### Etymology.

This species is named for its type (and sole known) locality, Nova Teutonia in Santa Catarina, Brazil.

### Operclipygus gratus sp. n.

urn:lsid:zoobank.org:act:59FC594F-1F13-4841-80E9-28D4800ED952

http://species-id.net/wiki/Operclipygus_gratus

**Figure 42.**
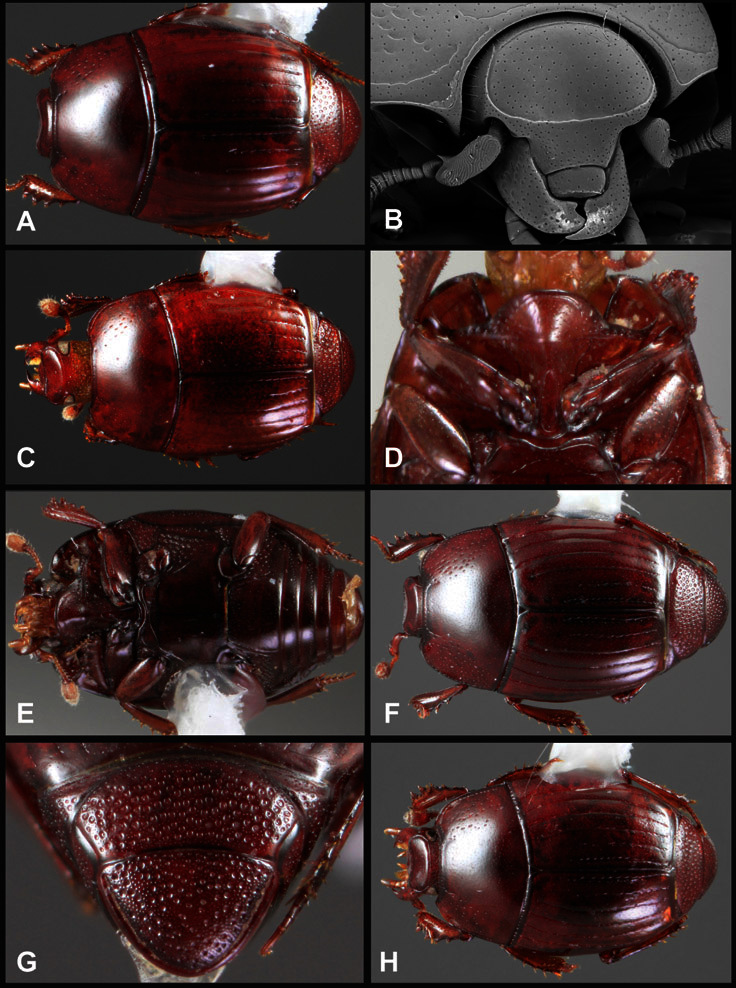
*Operclipygus hospes* group. **A** Dorsal habitus of *Operclipygus gratus* **B** Frons of *Operclipygus gratus* **C** Dorsal habitus of *Operclipygus confertus* **D** Prosternum of *Operclipygus confertus* **E** Ventral habitus of *Operclipygus rileyi* **F** Dorsal habitus of *Operclipygus assimilis* **G** Pygidia of *Operclipygus* assimilis **H** Dorsal habitus of *Operclipygus praecinctus*.

**Map 16.**
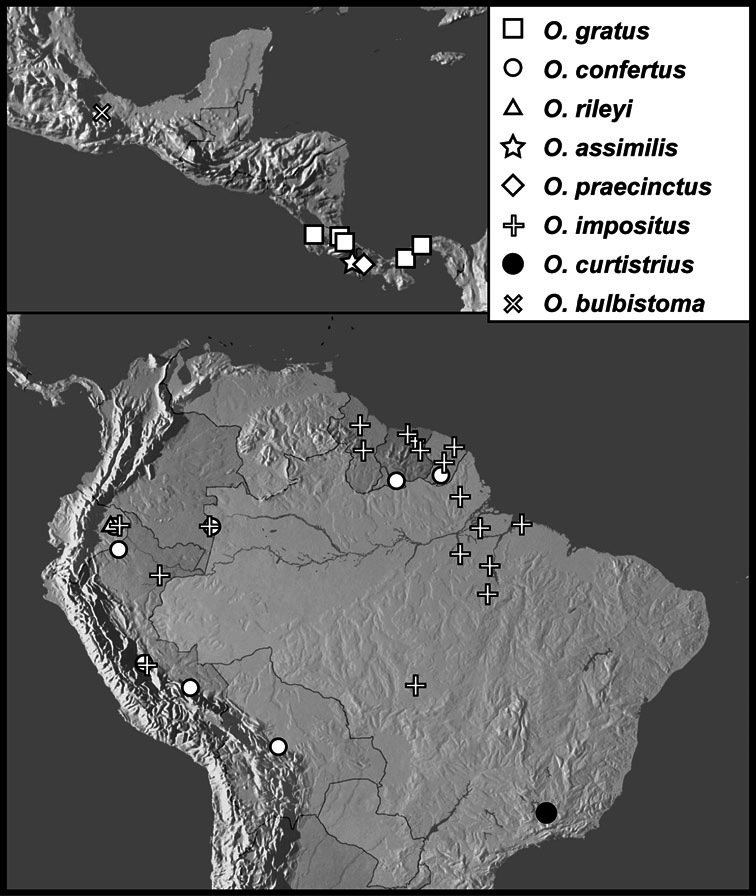
Records of the *Operclipygus hospes* group.

Figs 42A–B 43A–D, I Map 16

#### Type locality.

COSTA RICA: Heredia: La Selva Biological Station 10°26'N, 84°1'W].

#### Type material.

**Holotype male**: “COSTA RICA: Heredia, La Selva, 3.2 km SE Puerto Viejo, 100m, 28 Jan. 1992, W. Bell, ex: flight intercept trap”/ “SEMC0903624 KUNHM-ENT” (INBIO). **Paratypes** (10) same data as type, except as noted: 1: 14.ii.1992 (SEMC), 1: 17.ii.1992 (SEMC), 1: 28.ii.1992 (SEMC), 1: 22.vi.1998, FIT, C.E. Carlton & A.K. Tishechkin (LSAM); **COSTA RICA: Limón:** 1: Area Cons. Tortuguero, Sector Cerro Cocori, Fca. de E. Rojas, 150m, i.1993, E. Rojas, (INBIO), 2: v.1993, E. Rojas, (INBIO, FMNH), 1: vi.1993, E. Rojas, (INBIO); **Guanacaste:** 1: Parque Nac. Guanacaste, Est. Pitilla [misspelled Patilla], 10°59'22"N, 85°25'33"W, 610m, 13–15.vii.2000, FIT, J. Ashe, R. Brooks, Z. Falin (SEMC); **Cartago:** 1: Parque Nac. Barbilla, R.F. Rio Pacuare. Turrialba, Send. Principal, 200–300m, 14–23.vii.2001, FIT, W. Arana (INBIO).

#### Other material.

**COSTA RICA: Puntarenas:** 1: Las Cruces Biol. Sta., 08°47.14'N, 82°57.58'W, 1330m, 28–30.v.2004, FIT, J.S. Ashe, Z. Falin, I. Hinojosa (SEMC); 1: Est. Biol. Las Cruces, San Vito, 17.viii–12.ix.1982, FIT, B.D. Gill (BDGC); 1: Est. Biol. Las Cruces, Coto Brus, 8°47'N, 82°57'W, 1000m, 22–23.iii.2002, FIT, A. Cline & A. Tishechkin (LSAM). **PANAMA: Coclé**: 1: El Cope, Atlantic Slope, 08°37'N, 80°35'W, 730m, 19–20.xi.1994, FIT, D. Windsor, C. Edwards (SEMC); **Colón:** 1: San Lorenzo Forest, 9°17'N, 79°58'W, 4–6.x.2003, FIT, A.K. Tishechkin (LSAM), 1: 12–13.v.2004, FIT, A.K. Tishechkin (GBFM), 2: 21–24.v.2004, FIT, A.K. Tishechkin (AKTC, MSCC); **Panamá**: 1: Pipeline Rd., 17–22.vi.1993, FIT, S. Lingafelter (SEMC); 1: Old Gamboa Rd., 15.vii.1993, FIT, D. Windsor (SEMC); 1: Barro Colorado Isl., 09°11'N, 79°51'W, 1.viii.1994, FIT, D. Banks (SEMC), 1: 7.vii.1994, FIT, D. Banks (SEMC); 1: Old Plantation Rd. 6.9km S Gamboa, 09°05'N, 79°40'W, 80m, 4–7.vi.1995, FIT, J.S. Ashe & R. Brooks (SEMC), 2: 7–22.vi.1995, FIT, J. Ashe, R. Brooks (SEMC).

#### Diagnostic description.

**Figure 43.**
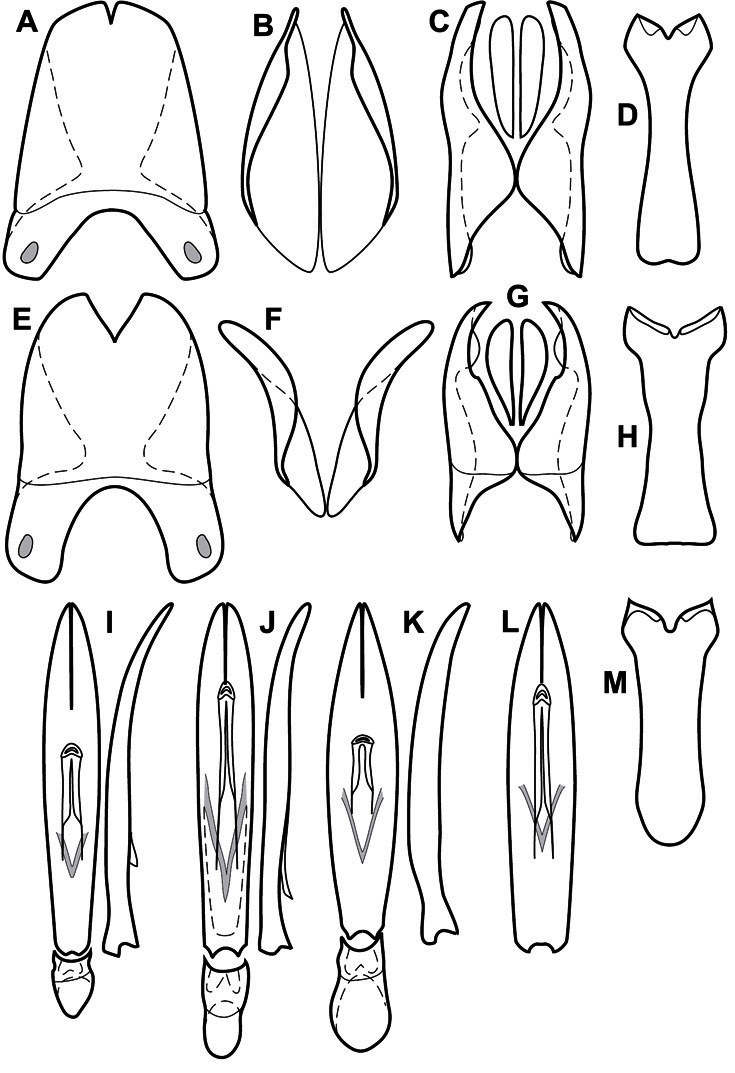
Male genitalia of *Operclipygus hospes* group. **A** T8 of *Operclipygus gratus* **B** S8 of *Operclipygus gratus* **C** T9 & T10 of *Operclipygus gratus* **D** S9 of *Operclipygus gratus* **E** T8 of *Operclipygus rileyi* **F** S8 of *Operclipygus rileyi* **G** T9 & T10 of *Operclipygus rileyi* **H** S9 of *Operclipygus rileyi* **I** Aedeagus, dorsal and lateral views, of *Operclipygus gratus* **J** Aedeagus, dorsal and lateral views, of *Operclipygus confertus* **K** Aedeagus, dorsal and lateral views, of *Operclipygus rileyi* **L** Aedeagus, dorsal and lateral views, of *Operclipygus praecinctus* **M** S9 of *Operclipygus assimilis*.

Length: 1.40–1.78 mm, width: 1.09–1.31 mm; body rufescent, elongate oval, widest near humeri; frontal stria complete, subcarinate at middle; epistoma depressed at middle, apical margin appearing subcarinate; antennal club with basolateral pit, with second annulus interrupted on dorsal surface; lateral submarginal pronotal stria present in apical third to half; anterior submarginal stria barely recurved, much shorter than lateral submarginal; pronotal disk with ~15 coarser punctures, most concentrated in anterolateral third; antescutellar region broadly depressed, with narrow prescutellar impression barely longer than scutellum; outer subhumeral stria present in apical half only, inner subhumeral stria absent, striae 1-3 complete, 4^th^ stria varied, from present in apical half to nearly complete, 5^th^ stria present in apical half to two-thirds, sutural stria present in apical three-fourths; prosternal keel distinctly produced at base, with carinal striae closely-set, subparallel, weakly convergent to front, united in anterior arch about three-fourths from base; mesoventrite distinctly and discretely emarginate at middle, marginal stria complete; mesometaventral stria subangulate to midpoint of mesoventral disk, extending posterlaterally toward outer corner of metacoxa; 1^st^ abdominal ventrite with two lateral striae complete, subparallel; propygidium with uniform round, shallow punctures separated by about their diameters; pygidium lacking apical marginal sulcus, ground punctation fine, dense, with coarser secondary punctures interspersed, denser along basal margin. Male genitalia (Fig. 43A–D, I): accessory sclerites present; T8 with sides weakly convergent apicad, with broad, shallow basal emargination, basal membrane attachment line about one-third distad basal emargination, apical emargination shallow, acute, with ventrolateral apodemes most well-developed basally, not meeting, narrowed to apex; S8 with sides moderately rounded in basal two-thirds, narrowing to apex, with apical guides narrow, not strongly upturned; S9 with sides subparallel in basal two-thirds, apices weakly convergent, bluntly acute at inner corners; T10 with halves separate; S9 rather short, narrowed at middle, expanded to broad, weakly emarginate base, with apical emargination narrow, apical flanges separate, desclerotized along midline; tegmen with sides weakly rounded, widest just distad midpoint, narrowed to subacute apex lacking subapical cleft, medioventral process well-sclerotized, acute, projecting beneath about one-fourth from base; median lobe about one-third tegmen length, proximal apodemes differentiated about two-thirds from gonopore; basal piece short, slightly less than one-fourth tegmen length.

#### Remarks.

In this species and the four that follow (through *Operclipygus assimilis*), the medioventral process of the aedeagus is acute (Figs 43I–L), and the tegmen lacks any subapicoventral cleft. Most of these species have the 9^th^ sternite of the male relatively short and broad (Figs 43D, H, M). All five of them exhibit a distinct sensory pit on the upper surface of the antennal club near the laterobasal edge (Fig. 4I). However, this is also found in *Operclipygus hospes* and in *Operclipygus ignifer*, a surprising conflict in otherwise apparently strong characters.

*Operclipygus gratus* can be distinguished from other species in the *Operclipygus hospes* group by the presence of a basolateral pit on the upper surface of the antennal club Fig. 4I), in combination with a depressed epistoma having a subcarinate anterior margin (Fig. 42B), frontal stria slightly carinate across the front, abbreviated lateral submarginal pronotal stria, and a complete lack of a marginal pygidial sulcus. Individuals from Costa Rica more typically have the 4^th^ elytral stria strongly abbreviated, whereas it is very nearly complete in Panamanian specimens. There are no other significant differences separating specimens from these regions that we could find so we keep them together as a single species, though the type series is limited to northeastern Costa Rican localities.

#### Etymology.

This species’ name hints at its pleasantly recognizeable morphology.

### Operclipygus confertus sp. n.

urn:lsid:zoobank.org:act:1B109F7A-59F8-4448-934E-C6A720713BA7

http://species-id.net/wiki/Operclipygus_confertus

Figs 42C–D 43J Map 16

#### Type locality.

ECUADOR: Orellana:Yasuní Research Station [0°40.5'S, 76°24'W].

#### Type material.

**Holotype male**: “**ECUADOR:** Napo, mid.Rio Tiputini, Yasuní Res. Stn. 0°40.5'S, 76°24'W, FIT #M1, 18–20 Jun 1999. AKT#008, C.Carlton & A.Tishechkin”/ “LSAM0013291” (FMNH). **Paratypes** (15): same data as type, except as noted: 1: 17–23.vi.1999 (LSAM), 1: 23–30.vi.1999 (LSAM), 1: 23–28.vi.1999 (FMNH), 1: 5–11.vii.1999 (LSAM), 1: 28.vi–5.vii.1999 (LSAM); 4: Parque Nac. Yasuní, Via Maxus at Puente Piraña, 0°39.5'S, 76°26'W, 14–20.vii.2008, FIT, A.K. Tishechkin (AKTC, MSCC, USFQ); 2: Tiputini Biodiversity Station, 0.6376°S, 76.1499°W, 4–9.vi.2011, FIT, M.S. Caterino & A.K. Tishechkin, DNA Extracts MSC-2193 & MSC-2184 (SBMNH); 1: 0°38.2'S, 76°8.9'W, 27–31.vii.2008, FIT, A.K. Tishechkin (AKTC), 1: 30.vii.2008, FIT, A.K. Tishechkin, DNA Extract MSC-1897 (AKTC); 2: 5–25.ix.2000, D.J. Inward & K.A. Jackson (BMNH).

#### Other material.

**BOLIVIA: Cochabamba:** 1: 67.5km NE Cochabamba, Est. Biol. Valle del Sajita Univ. de San Simon, 17°6'33"S, 64°47'52"W, 300m, 7–9.ii.1999, FIT, R. Hanley (SEMC). **COLOMBIA: Vaupés:** 1: Est. Biol. Caparú, Rio Apoporis, 1.1°S, 69.5°W, 27.ix–1.xii.1995, FIT, Black-water terrace forest on sandy soils, B.D. Gill (BDGC). **FRENCH GUIANA:** 1: Mont. Tabulaire, Itoupé, 3°1.82'N, 53°6.40'W, 400m, 23.iii.2010, FIT, SEAG (CHND). **PERU: Junín:** 1: ~16km NW Satipo, Rio Venado, 11°11.935'S, 74°46.227'W, 1110m, 19–20.ii.2010, A.V. Petrov (AKTC), 3: 3–8.iii.2010, A.V. Petrov (AKTC), 7: 8–14.iii.2010, A.V. Petrov (AKTC, MUSM); **Loreto:** 1: 1.5km N Teniente Lopez, 2°35.66'S, 76°06.92'W, 210–240m, 26.vii.1993, FIT, R. Leschen (SEMC); 2: km 63, rd. Iquitos - Nauta, Rio Itaya, 4°15.205'S, 73°26.074'W, 140m, 5–6.ii.2010, A.V. Petrov (AKTC, MUSM); **Madre de Dios:** 1: Manu National Park, Pantiacolla Lodge, 5.5km NW El Mirador Trail, Alto Madre de Dios River, 12°39'10"S, 71°15'28"W, 500m, 23–26.x.2000, FIT, R. Brooks (SEMC). **SURINAME: Sipaliwini:** 2: CI-RAP Surv. Camp 1: on Kutari River, 2°10.521'N, 56°47.244'W, 228m, 19–24.viii.2010, FIT, T. Larsen & A.E.Z. Short (SEMC); 1: CI-RAP Surv. Camp 3: Wehepai SE Kwamala, 2°21.776'N, 56°41.861'W, 237m, 3–7.ix.2010, FIT, T. Larsen & A.E.Z. Short (SEMC).

#### Diagnostic description.

This species is extremely similar to *Operclipygus gratus*, differing as follows: length: 1.56–1.84 mm, width: 1.19–1.40 mm; epistoma depressed, but not subcarinate along apical margin; pronotum with fewer coarse lateral punctures (<15); basal sides of pronotum weakly sinuate, appearing weakly constricted; lateral submarginal pronotal stria obsolete in basal fifth, nearly complete, slightly divergent from margin near base; anterior submarginal pronotal stria barely recurved at ends; 5^th^ elytral stria shorter, present in no more than apical half; prosternum with carinal striae of keel very short, united in narrow arch one-third from presternal suture; lateral metaventral stria long, extending to near outer corner of metacoxa, tending to bend laterad at apex; propygidium with coarse punctures shallow, slightly elongate, separated by about their widths; pygidium with ground punctation very coarse and dense, with slightly coarser punctures almost indistinguishable (but present and scattered throughout); marginal pygidial sulcus complete, deep, crenulate primarily on inner edge, outer edge relatively smooth. Male genitalia very similar to those of *Operclipygus gratus* (see Figs 43A–D), but with tegmen (Fig. 43J) more elongate, more nearly parallel-sided, less strongly narrowed to base, with medioventral process more elongate and distinctly projecting beneath about one-sixth from base; median lobe more elongate, about two-thirds length of tegmen, with narrow gonopore.

**Remarks.** This species can be distinguished by its rather convex shape, depressed epistoma, basally sinuate or constricted pronotal margin, abbreviated lateral submarginal pronotal stria (Fig. 42C), large prescutellar impression, and shortened prosternal striae (Fig. 42D). It is similar not only to *Operclipygus gratus*, but also to *Operclipygus hospes* and *Operclipygus ignifer*, sharing their large prescutellar impression especially. However, the medioventral process of the aedeagus is entirely different. Externally, neither of those species have the epistoma nearly as depressed as *Operclipygus confertus*.

Due to the extreme external similarity of this species with multiple others, and some evident variation in punctation and striation, we restrict the type series to those specimens from eastern Ecuador.

#### Etymology.

This species’ name refers to the weak but distinctive constriction near the base of the pronotal margin.

### Operclipygus rileyi sp. n.

urn:lsid:zoobank.org:act:03E00155-1CDB-4EE2-B449-5D593E33827F

http://species-id.net/wiki/Operclipygus_rileyi

Figs 42E 43E–H, K Map 16

#### Type locality.

ECUADOR: Orellana: Yasuní Research Station [0°40.5'S, 76°24'W].

#### Type material.

**Holotype male**: “ECUADOR: Napo Prov. Estacion Cientifica Yasuní, 00°40'28"S, 76°38'50"W, IX-5-10-1999. 215m”/ “Coll. E. G. Riley, flight intercept trap primary forest” / “LSAM 0013297 (TAMU). **Paratypes** (2): same data as type (TAMU, FMNH).

#### Diagnostic description.

Length: 1.56–1.65 mm, width: 1.22–1.28 mm; this species is externally nearly identical to *Operclipygus gratus*, lacking a pygidial sulcus and having subcarinate frontal and epistomal margins. It differs slightly in having the lateral striae of the 1^st^ abdominal ventrite both complete and closer together, as well as in male genitalic characters (Figs 43E–H, K): accessory sclerites present, small; T8 rather short, with sides evenly rounded to apex, basal emargination narrow, deep, rounded, nearly reaching basal membrane attachment line, ventrolateral apodemes most strongly developed near base, diverging to apex; S8 with sides parallel in basal half, strongly divergent and downturned apically, halves divergent from base; T9 with sides rounded to subacute apices, ventrolateral apodemes displaced to near apex; T10 halves separate; S9 short, broad, with wide truncate base; lateral flanges minimally differentiated from apical expansion, apex with very narrow median emargination, apical flanges short, separate; tegmen widest at midpoint, evenly narrowed to base and apex, medioventral process long, apically acute, weakly sclerotized, not projecting beneath; basal piece about one-fourth length of tegmen; median lobe about one-third length of tegmen, with proximal apodemes strongly differentiated, but with narrow portions still well sclerotized, not filamentous.

#### Remarks.

Aside from their allopatric distributions, this species can only be reliably separated from *Operclipygus gratus* by its male genitalia, which differs in several characters, most significantly the very divergent apices of S8 (Fig. 43F) and rounded sides of the tegmen (Fig. 43K).

#### Etymology.

We name this species for chrysomelid specialist Edward Riley, in recognition of his numerous contributions to this study.

### Operclipygus assimilis sp. n.

urn:lsid:zoobank.org:act:81FED8A5-DF50-42EC-8E35-CF373CFAAB83

http://species-id.net/wiki/Operclipygus_assimilis

Figs 42F–G 43M Map 16

#### Type locality.

COSTA RICA: Puntarenas: Las Cruces Biological Station [8°17'N, 82°57'W].

#### Type material. 

**Holotype male**: “**COSTA RICA: Puntarenas**, Coto Brus, Estac. Biol. Las Cruces. 8°17'N, 82°57'W 1100m. Flight intercept # 2, 18–20 June 2005. M.Ferro” / “Caterino/Tishechkin Exosternini Voucher EXO-00293” (FMNH).

#### Diagnostic description.

This species is extremely similar to *Operclipygus gratus*, differing as follows: length: 1.97 mm, width: 1.47 mm; anterior half of frons and epistoma depressed at middle; frontal stria fine, transverse; apical margin of epistoma convex, but not subcarinate; prescutellar impression elongate, about twice length of scutellum, acute anteriorly; pronotal disk with ground punctation conspicuous, with numerous (~40) punctures at sides; lateral submarginal pronotal stria complete, curved inward, nearly meeting anterior submarginal stria; anterior submarginal stria strongly recurved, extending posterad about one-fourth pronotal length; median pronotal gland openings laterad recurved arms of anterior submarginal stria, about 8 puncture widths from anterior margin; propygidium with uniform, shallow, round punctures separated by about half their diameters; pygidium with ground punctation moderately dense, with much coarser punctures irregularly but rather densely interspersed; marginal pygidial sulcus complete, deep, crenulate on inner and outer edges. Male genitalia: indistinguishable from those of *Operclipygus gratus* (see Figs 43A–C), except for S9 (Fig. 43M) rounded at base, and its lateral flanges less strongly differentiated from apical sides.

#### Remarks.

This species may be separated from similar species by the less strongly impressed epistoma, large prescutellar impression (Fig. 42F), numerous lateral pronotal punctures, complete lateral submarginal pronotal stria, and deep, complete marginal pygidial sulcus (Fig. 42G).

#### Etymology.

This species’ name underscores the general similarity of *Operclipygus hospes* group species to each other.

### Operclipygus praecinctus sp. n.

urn:lsid:zoobank.org:act:606BB831-9C72-424D-9233-EC8626E77DAF

http://species-id.net/wiki/Operclipygus_praecinctus

Figs 42H 43L Map 16

#### Type locality.

PANAMÁ: Chiriquí: La Fortuna [08°42'N, 82°14'W].

#### Type material.

**Holotype male**: “Panama: Chiriquí Prov. La Fortuna. “Cont. Div. Trail” 08°42'N, 82°14'W, 1150m, 9–12 VI 1995, J. Ashe & R. Brooks#186, ex: flight intercept trap”/ “SEMC0903667” (SEMC).

#### Diagnostic description.

This species is extremely similar to *Operclipygus assimilis*, except for the following characters: length: 1.65 mm, width: 1.28 mm; body slightly shorter, more broadly rounded; second antennal annulus not interrupted on dorsal surface; coarser lateral pronotal punctures not as numerous, <20; lateral submarginal pronotal stria slightly abbreviated at base; anterior submarginal pronotal stria recurved posterad only about one-sixth pronotal length; elytral stria 4 nearly complete, just barely abbreviated at base. Male: more or less indistinguishable from *Operclipygus gratus* (see Figs 43A–D) except in having a slightly larger overall, and relatively broader tegmen (Fig. 43L).

#### Remarks.

While the characters listed in the description will help to distinguish this species from *Operclipygus gratus* and *Operclipygus assimilis*, examination of the aedeagus is necessary for unambiguous identification.

#### Etymology.

This species’ name refers to its limited distribution, being known only from the type locality in Chiriquí, Panama.

### Operclipygus impositus sp. n.

urn:lsid:zoobank.org:act:DECF9B78-D51C-4B51-9CDC-1FDBDC5EBE1A

http://species-id.net/wiki/Operclipygus_impositus

**Figure 44.**
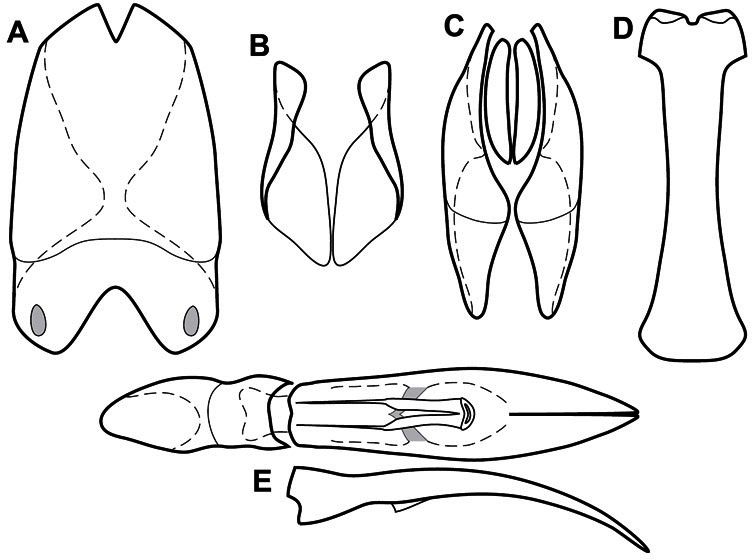
Male genitalia of *Operclipygus impositus* (*hospes* group). **A** T8 **B** S8 **C** T9 & T10 **D** S9 **E** Aedeagus, dorsal and lateral views.

Figs 44A–E 45A–B Map 16

#### Type locality.

BRAZIL: Pará: km 18 Altamira – Marabá road [3°09'S, 52°03'W[

#### Type material.

**Holotype male**: “**BRASIL: Pará:** Altamira – Marabá: km 18, 3°09'S, 52°03'W. Piège de’interception. v.1984” / “Caterino/Tishechkin Exosternini Voucher EXO-00307” (UFPR). **Paratypes** (23): 1: same data as type(CHND); **BRAZIL: Amapá:** 3: Serra do Navio, 0°59'N, 52°00'W, 1–14.v.1991, FIT (CHND, MNHN); **Mato Grosso:** 1: Sinop, 11°49'S, 55°29'W, 12–24.vi.1985, FIT (CHND); **Pará:** 3: Tucuruí, 3°45'S, 49°40'W, 20.v–15.vi.1987, FIT (CHND, FMNH), 1: iv.1986, FIT (CHND), 1: IPEAN, Utinga, Belém, 1°27'S, 48°26'W, v.1985, FIT (CHND), 2: viii.1985, FIT (CHND), 1: x.1985, FIT (CHND), 2: xi.1984, FIT (MSCC, CHND); 3: Carajás, Serra Norte, 6°04'S, 50°12'W, 13.xi-2.xii.1987, FIT (CHND, UFPR, AKTC); 5: Marajo-Breves, 0°53'S, 50°32'W, 18.xi-5.xii.1987, FIT (CHND, UFPR).

#### Other material

**. COLOMBIA: Vaupés:** 1: Parque Nac. Mosiro-Itajura (Caparú), Centro Ambiental, 1°04'S, 69°31'W, 60m, 20–30.i.2003, FIT, D. Arias & M. Sharkey (IAVH). **ECUADOR: Orellana:** 1:Res. Ethnica Waorani, 1km S Onkone Gare Camp, Trans. Ent., 0°39'10"S, 76°26'W, 220m, 16.i.1994, fogging, T.L. Erwin (USNM); 1: Yasuní Res. Stn., 215m, 5–10.ix.1999, FIT, primary forest, E.G. Riley (TAMU); 1: Tiputini Biodiversity Station, 0.6376°S, 76.1499°W, 4–9.vi.2011, FIT, M.S. Caterino & A.K. Tishechkin, DNA Extract MSC-2190 (SBMNH); 1: 5–25.ix.2000, D.J. Inward & K.A. Jackson (BMNH); **Sucumbíos:** 2: Sacha Lodge, 0°28'14"S, 76°27'35"W, 270m, 21–24.iii.1999, FIT, R. Brooks (SEMC). **FRENCH GUIANA:** 1: Roura, 18.4km SSE, 4°36'38"N, 52°13'25"W, 240m, 29.v–10.vi.1997, FIT, J. Ashe, R. Brooks (SEMC); 2: Saül, 7km N, Les Eaux Claires, 3°39'46"N, 53°13'19"W, 220m, 31.v–3.vi.1997, FIT, J. Ashe, R. Brooks (SEMC); 1: Belvèdére de Saül, point de vue, 3°1'22"N, 53°12'34"W, 20.xii.2010, Window trap, SEAG (MNHN); 1: Rés. Natur. des Nouragues, Camp Inselberg, 4°05'N, 52°41'W, 25.i.2011, Window trap, SEAG (MNHN), 1: 9.xi.2010, Window trap, SEAG (MNHN). **GUYANA: Mazaruni Potaro**: 1: Takutu Mountains, 6°15'N, 59°5'W, 11.xii.1983, Window trap, montane rainforest near logging area, P.J. Spangler, R.A. Faitoute & W.E. Steiner (USNM); **Region 8**: 1: Iwokrama Forest, Turtle Mt. summit, 4°43'57"N, 58°44'1"W, 290m, 30.v–1.vi.2001, FIT, R. Brooks & Z. Falin (SEMC). **PERU: Junín:** 1: 11km NE Puerto Ocopa, Los Olivos, 11°7.00'S, 74°15.52'W, 1200m, 24–26.iii.2009, Window trap, A.V. Petrov (AKTC), 1: 26–28.iii.2009, Window trap, A.V. Petrov (AKTC), 1: 30–31.iii.2009, Window trap, A.V. Petrov (MUSM); **Loreto:** 1: 68km SW Iquitos to Nauta, Rio Itaya, 4°11'S, 73°26'W, 110m, 28.ii.2008, A.V. Petrov (AKTC), 1: 1–3.iii.2008, A.V. Petrov (MUSM). **SURINAME: Brokopondo:** 1: Brownsberg Nature Preserve, Witi Creek Trail, 4°56'55"N, 55°10'53"W, 480m, 23–25.vi.1999, FIT, Z. Falin, A. Gangadin, H. Hiwat (SEMC); **Marowijne**: 1: Nassau Mountain, 4°48'36"N, 54°31'16"W, 500m, 2–4.vi.1999, FIT, Z. Falin, B. DeDijn (SEMC); **Para**: 1: nr. Overbridge River Resort, 5°31.8'N, 55°3.5'W, 15–18.ii.2010, FIT, C. Gillet, P. Skelley, W. Warner (FSCA).

#### Diagnostic description.

**Figure 45.**
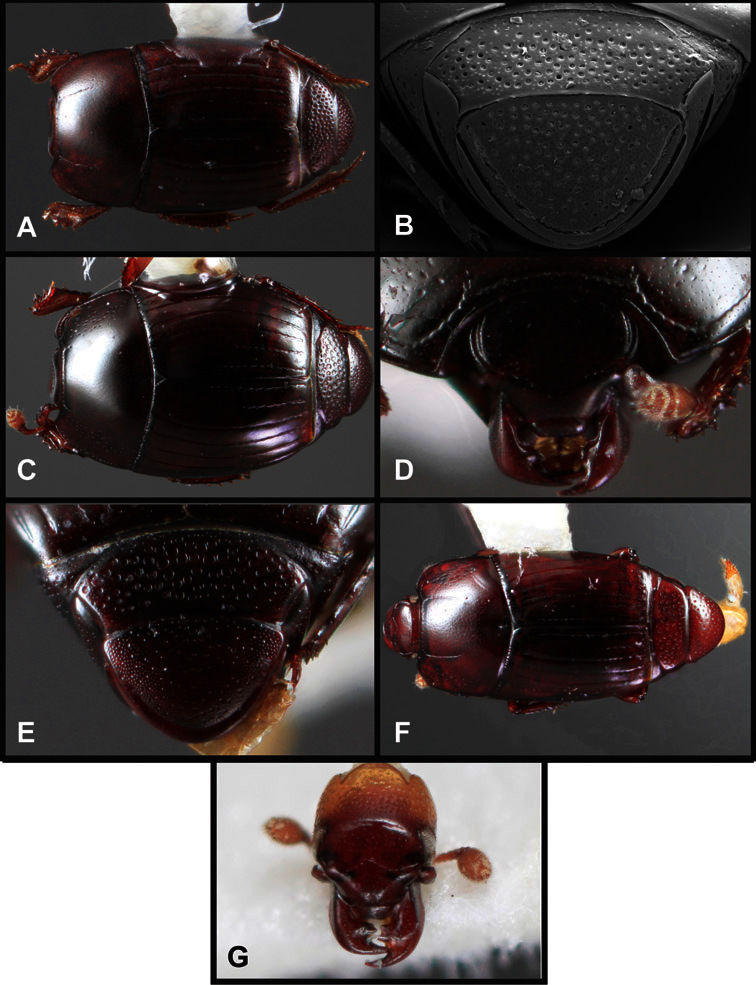
*Operclipygus hospes* group. **A** Dorsal habitus of *Operclipygus impositus* **B** Pygidia of *Operclipygus impositus* **C **Dorsal habitus of *Operclipygus curtistrius* **D** Frons of *Operclipygus curtistrius* **E** Pygidia of *Operclipygus curtistrius* **F** Dorsal habitus of *Operclipygus bulbistoma* **G** Frons of *Operclipygus bulbistoma*.

Length: 1.22–1.40 mm, width: 0.84–0.97 mm; body rufobrunneus, elongate, sides subparallel, depressed; frons weakly depressed at middle, frontal stria complete, sinuate, slightly recurved at middle; supraorbital stria complete, meeting sides of frontal stria; epistoma broad, sides rounded, emarginate across anterior margin; labrum half as long as wide, straight across apical margin; left and right mandibles both with acute basal tooth; pronotal disk with linear prescutellar impression about 1.5× as long as scutellum; lateral submarginal pronotal stria absent, anterior marginal stria interrupted behind head; anterior submarginal stria strongly recurved posterad, reaching pronotal midpoint; median pronotal gland openings along side base of recurved arms of anterior stria, about 4 puncture widths from anterior margin; elytra with 1 complete epipleural stria, outer subhumeral stria present in apical half, inner subhumeral stria absent, striae 1-4 complete, 5^th^ stria present in apical half, sutural stria present in apical three-fourths; prosternal keel with base produced, rounded, carinal striae long, close and subparallel in anterior three-fourths, united in narrow anterior arch, separate basally; mesoventrite emarginate anteriorly, marginal stria interrupted; mesometaventral stria narrowly arched forward to near margin, sinuate laterally, continued by lateral metaventral stria to near middle of metacoxa; 1^st^ abdominal ventrite with two complete lateral striae; propygidium (Fig. 45B) with sparse fine ground punctation, small round coarser punctures uniformly separated by about their diameters; pygidium with fine, dense ground punctation, with distinct coarser punctures densely interspersed, especially near basal margin; marginal pygidial sulcus complete, deep, strongly crenulate on inner and outer edges. Male genitalia (Figs 44A–E): accessory sclerites present; T8 elongate, sides slightly convergent to apex, basal emargination shallow, narrow, basal membrane attachment line distad emargination by nearly its depth, apical emargination narrow, shallow, ventrolateral apodemes most strongly developed at base, nearly meeting at midline, narrowed to apex; S8 short, widest at base, sides weakly rounded, apical guides developed throughout length, widened gradually to apex, apices subacute in outer corners, halves separate, strongly divergent in apical half; T9 with sides subparallel in basal two-thirds, convergent to narrow, obliquely truncate apices; T10 with halves separate; S9 broad, weakly desclerotized along midline, narrowest at middle, strongly widened to broadly rounded base, lateral flanges wide, apex with small median emargination, apical flanges short, separate; tegmen widest just beyond midpoint, narrowing to subacute base, becoming quite flat dorsoventrally, curving downward toward apex, with wide ‘V’-shaped medioventral process strongly projecting beneath about one-third from base; basal piece nearly half tegmen length; median lobe over half tegmen length, with proximal apodemes not strongly differentiated, proximal portions conspicuous.

#### Remarks.

In general body shape (Fig. 45A) this species strongly resembles members of the *Operclipygus dubius* group. However, its male genitalia absolutely preclude placement there. This species can be distinguished by its lack of lateral submarginal pronotal stria, and by its elongate, subcylindrical body shape. It is very similar to the sympatric *Operclipygus minutus*, but is more distinctly parallel-sided and subdepressed, and has entirely different genitalia, lacking the truncate medioventral process and subapical tegmenal cleft characteristic of that species and several close relatives. The type series is limited to those specimens from northeastern Brazil, as populations from other areas do exhibit some variation, some with anterior fragments of the lateral submarginal pronotal stria.

#### Etymology.

The Latin name *impositus* is related to the English impostor, as this species looks very much like members of the *Operclipygus dubius* species group.

### Operclipygus curtistrius sp. n.

urn:lsid:zoobank.org:act:9A436016-7425-420B-963F-862C7F9F1DC3

http://species-id.net/wiki/Operclipygus_curtistrius

Figs 45C–E Map 16

#### Type locality.

BRASIL: Minas Gerais,Boqueirão Reserve near Lavras [21°14'S, 45°0'W].

#### Type material.

**Holotype female**: “**BRASIL: Minas Gerais,** Municip. Ingaí, Res. Boqueirão nr. Lavras. Gallery forest, F.I.T., 13–20 Nov 2002, F.Freiro-Costa”/ “Caterino/Tishechkin Exosternini Voucher EXO-00312” (CEMT).

#### Diagnostic description.

Length: 1.93 mm, width: 1.56 mm; body rufopiceous, rather strongly elongate-oval, moderately convex, not depressed; frons weakly depressed at middle, with fine, inconspicuous ground punctation; sides of frontal stria rounded, evenly curving over antennal bases, finely impressed, complete across front; epistoma weakly convex across anterior edge, weakly emarginate; labrum about twice as wide as long, shallowly emarginate apically; left mandible untoothed, right with small, acute basal tooth; pronotum with short, sublinear, anteriorly bulbous prescutellar impression, pronotal disk slightly depressed across base, with fine, inconspicuous ground punctation and ~15 coarse lateral punctures in a band slightly removed from margin; marginal pronotal stria broadly interrupted behind head; lateral submarginal stria complete along side, curving inward at front, nearly meeting anterior submarginal stria, which is very briefly recurved at sides; median pronotal gland openings present just beyond recurved ends of anterior submarginal stria; elytra with one complete epipleural stria, outer subhumeral stria present in apical half, inner subhumeral stria absent, dorsal striae 1-4 complete, 5^th^ stria present in apical third, sutural stria present in apical two-thirds; prosternal keel produced at base, with complete carinal striae divergent posteriorly, connected though somewhat fragmented along basal margin, united in narrow anterior arch; prosternal lobe with complete marginal stria; anterior margin of mesoventrite broadly, rather deeply emarginate, with complete marginal stria; mesometaventral stria narrowly arched forward to about mesoventral midpoint, sinuate at sides, continued posterolaterad by lateral metaventral stria toward outer corner of metacoxa, abruptly bent laterad at apex; 1^st^ abdominal ventrite with inner lateral stria complete, outer lateral stria obsolete apically; propygidium with fine ground punctation mostly sparse, becoming slightly denser apically, with rather large, shallow secondary punctures irregularly separated by less than their widths; pygidium with fine ground punctation moderately dense, with small secondary punctures sparsely intermingled, denser along basal margin; marginal pygidial sulcus slightly separated from margin, with smooth, flat marginal bead, deeply impressed in just over apical half, obsolete basally. Male not known.

#### Remarks.

This species, although known from only a single female specimen, is quite distinctive in the *Operclipygus hospes* group, particularly in the fairly broadly rounded body form (Fig. 45C), deeply impressed but basally obsolete pygidial sulcus (Fig. 45E). The weakly depressed frons with fine, complete frontal stria (Fig. 45D) is also rather unusual in the group. Discovery of a male would help confirm placement within the *hospes* group.

#### Etymology.

This species’ name refers to the shortened marginal pygidial stria.

### Operclipygus bulbistoma sp. n.

urn:lsid:zoobank.org:act:00657B9B-A591-4789-B5A4-41A6B564B4EA

http://species-id.net/wiki/Operclipygus_bulbistoma

Figs 45F–G Map 16

#### Type locality.

MEXICO: Oaxaca: 26 km E Valle Nacional [18°2'N, 96°2'W].

#### Type material.

**Holotype female**: “MEX: Oax; 1220m, 26km E Valle Nacional, 25.VI–2.VIII.83,km71, S&J.Peck, FIT, mont. trop. forest”/ “♀”/ “Caterino/ Tishechkin Exosternini Voucher EXO-01296” (CMNC).

#### Diagnostic description.

Length: 1.78 mm, width: 1.12 mm; body rufobrunneus, very elongate, parallel-sided, subdepressed; frons flat, with sides of frontal stria more or less parallel between eyes, central part complete, arching dorsad at middle; supraorbital stria weak, fragmented at middle, detached from frontal stria; epistoma strongly convex, with conspicuous longitudinal waves of microsculpture on the sides; labrum rounded at sides, narrowing to apex, apex appearing narrowly emarginate, with an apical concavity encircled by a sharp carina; left mandible with prominent bifid tooth; right mandible with smaller but similar bifid tooth; pronotal disk with short, narrow, distinct prescutellar impression, but with longer vague depression extending forward in posterior half of disk; ground punctation fine, but relatively conspicuous, with ~15 coarser punctures at sides; lateral submarginal stria absent from sides, only present as short fragments in anterior corners that meet the anterior submarginal stria at a narrow angle; anterior marginal stria weak at front, interrupted behind head; anterior submarginal stria strongly recurved straight posterad reaching behind middle of pronotum; median pronotal gland openings beyond ends of anterior stria, about two-thirds pronotal length from anterior margin; elytra with one complete epipleural stria; outer subhumeral stria complete, inner subhumeral stria absent, striae 1-4 complete to base, striae 1-2 barely obsolete at apex, 5^th^ stria present in apical third, sutural stria present in apical four-fifths, the 2^nd^-5^th^ striae weakly expanded at apices; prosternal keel weakly produced at base, with very shallow median emargination, carinal striae faint, present in basal half only, not connected anteriorly or posteriorly; prosternal lobe lacking marginal stria; mesoventrite shallowly, broadly emarginate, with marginal stria mostly absent, only weakly present at sides; mesometaventral stria arched forward to near mesoventral margin, continued by lateral metaventral stria extending posterad to inner third of metacoxa; 1^st^ abdominal ventrite with inner lateral stria complete, outer abbreviated and divergent posteriorly; profemur strongly produced along midanterior margin, the protibia correspondingly bent at middle; propygidium with moderately large, shallow ocellate punctures separated by about their widths; pygidium with fine, sparse ground punctation, coarser punctures ocellate, sparse, separated by slightly more than their diameters; marginal pygidial sulcus absent, although vague fragments of a marginal depression can be seen near apex. Male not known.

#### Remarks.

This species can be very easily recognized by its elongate body form (Fig. 45F), long recurved anterior submarginal stria, and by the strongly convex epistoma (Fig. 45G). Its relationships are obscure, and it is placed in the *Operclipygus hospes* species group only tentatively. Some superficial characters (mainly pronotal striae and general body form) might also suggest placement in the *Operclipygus dubius* group, and the position of the openings of the median pronotal glands and the near absence of prosternal striae would be highly apomorphic in either group. Discovery of a male would help to clarify this question.

#### Etymology.

The name of this species refers to its prominently convex epistoma.

### *Operclipygusfarctus* group

**Figure 46.**
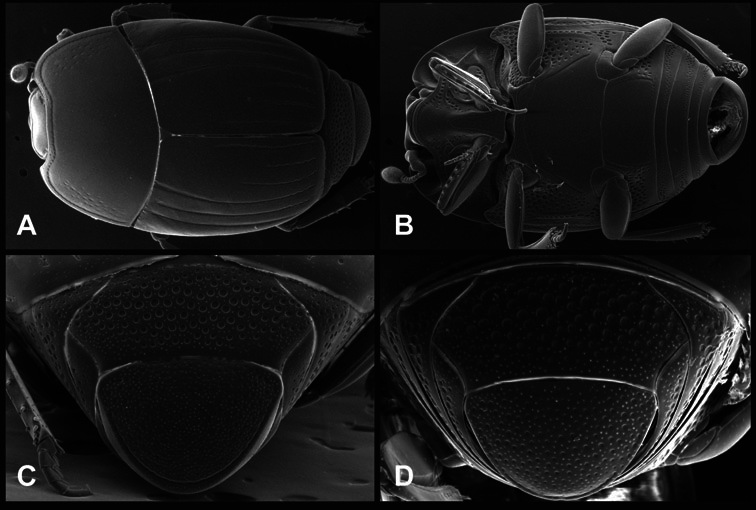
*Operclipygus farctus* group. **A** Dorsal habitus of *Operclipygus farctus* **B** Ventral habitus of *Operclipygus farctus* **C **Pygidia of *Operclipygus farctus* **D** Pygidia of *Operclipygus bidessois*.

The *Operclipygus farctus* group comprises sixteen closely related species, which span a broad range of morphological variation. The species are primarily united by the configuration of anterior pronotal gland openings, with both glands located along the anterior marginal bead (Fig. 5A), always in front of a submarginal stria that is continuous from the sides all the way across the front. The position of the outer gland opening varies from the anterolateral corner to quite close to the median opening behind the eye. In nearly all of the species the base of the prosternal keel is weakly emarginate (Fig. 46B), not completely truncate or outwardly arcuate, and the mesoventrite is correspondingly weakly projecting anteriorly, although *Operclipygus distractus* and *Operclipygus punctifrons* are exceptions, with a weakly produced prosternal keel. Most also have distinctly dual punctation on the pygidium, with sparse secondary punctures against a dense ground punctation (Fig. 46C), and have a simple, generally fine marginal pygidial sulcus, although such conditions are found widely in the genus. The male genitalia (Figs 48, 51) are generally very similar across the group, with accessory sclerites present, T8 usually desclerotized at apicolateral angle, where ventral apodemes end, S8 with apical guides mostly evenly developed from base to apex, apex of S9 entire, with uninterrupted apical flange, aedeagus strongly tapered to apex, apex bent abruptly at apical third; medioventral process narrowly ‘U’-shaped, distinctly but not strongly produced beneath. Beyond this similarity, however, the general body shape varies considerably, with a few species showing a rather elongate, subdepressed form, and others showing a quite broadly rounded, strongly convex shape.

### Key to the species of the *Operclipygus farctus* group

**Table:** 

1	1^st^ abdominal ventrite with two lateral striae (outer may be slightly abbreviated or curved behind metacoxa)	2
–	1^st^ abdominal ventrite with one lateral stria, rarely with fragments of an additional, outer stria	6
2	Outer lateral stria of 1^st^ abdominal ventrite curving behind metacoxa; inner subhumeral elytral stria absent	3
–	Lateral striae of 1^st^ abdominal ventrite parallel; inner subhumeral stria present or absent	4
3	Outer subhumeral stria not complete, either present only in apical half or with short basal fragment; Central America	*Operclipygus gilli* sp. n.
–	Outer subhumeral stria more or less complete, may be faintly abbreviated at apex, but never interrupted at middle; South America	*Operclipygus proximus* sp. n.
4	Central portion of frontal stria absent, or represented by few remnant fragments (Fig. 49H); inner subhumeral elytral stria present as distinct stria, varied in length	*Operclipygus prolixus* sp. n.
–	Frontal stria complete (Fig. 53B); inner subhumeral stria absent or represented by few apical fragments	5
5	Frontal stria complete; frontal disk with distinct, dense fine punctation; 4^th^ elytral stria complete or nearly so	*Operclipygus punctifrons* sp. n.
–	Frontal stria interrupted at sides; frontal disk with sparser ground punctation; 4^th^ dorsal stria present in apical half only	*Operclipygus distractus* (Schmidt)
6	Body large, >4 mm, subdepressed, subquadrate (Fig. 53C); dorsal elytral striae fine, not strongly impressed; labrum wide, emarginate apically (Fig. 53D); epistoma strongly convex, frons depressed behind frontal suture; southeastern Brazil	*Operclipygus latemarginatus* (Bickhardt)
–	Body smaller, convex to subdepressed; dorsal striae strongly impressed toward apices; labrum narrower, not distinctly emarginate apically; epistoma not much more strongly convex than frons	7
7	Pygidium completely lacking marginal sulcus	*Operclipygus plicatus* (Hinton)
–	Marginal sulcus of pygidium present, though it may be weak and/or fragmented toward base	8
8	Anterior marginal pronotal gland openings close together, outer about half as far from inner as outer is from anterior pronotal corner (Fig. 47C)	9
–	Anterior marginal pronotal gland openings further apart, outer nearly twice as far from inner as it is from anterior pronotal corner	13
9	Body strongly convex (Fig. 50C), elytra elevated above pronotum in lateral view, interval between subhumeral and 1^st^ elytral striae strongly convex relative to interval between 1^st^ and 2^nd^ dorsal striae; anterior pronotal angles turned downward, approximately vertical next to head	10
–	Body less strongly convex, elytral-pronotal profile more or less even; subhumeral elytral interval only slightly more strongly convex than 1^st^-2^nd^ strial interval; anterior pronotal angles at least slightly oblique next to head	12
10	Pronotal disk lacking coarse lateral punctures; anterior pronotal margin strongly projecting above head	*Operclipygus inflatus* sp. n.
–	Pronotal disk with distinct coarse lateral punctures; anterior pronotal margin no more than weakly projecting above head	11
11	Outer subhumeral stria present in apical half and generally with additional, detached basal fragment; 4^th^ elytral stria longer, generally present on apical two-thirds (Fig. 50A)	*Operclipygus impressistrius* sp. n.
–	Outer subhumeral stria present in apical half only, without basal fragment; 4^th^ elytral stria shorter, present on less than apical half	*Operclipygus farctissimus* sp. n.
12	Outer subhumeral stria most often complete to base, rarely interrupted or abbreviated; inner subhumeral stria usually represented by few apical fragments; pygidium depressed along basal margin, with deep, coarse punctures interspersed with dense, fine ground punctation	*Operclipygus distinctus* (Hinton)
–	Outer subhumeral stria present in apical half only; inner subhumeral stria not evident; pygidium usually flatter, with coarse punctures not as large or dense	*Operclipygus atlanticus* sp. n.
13	Marginal mesoventral stria complete along anterior margin	14
–	Marginal mesoventral stria interrupted for some distance at middle	15
14	Body frequently appearing faintly bicolored, with rufescent elytra and darker pronotum (Fig. 47A), rounded, moderately convex; frontal stria interrupted over antennal bases	*Operclipygus subrufus* sp. n.
–	Body unicolorous, rufescent to rufobrunneus, more elongate, subdepressed; frontal stria complete	*Operclipygus farctus* (Marseul)
15	Apical margin of elytron with series of small punctures; ground punctation of pygidium generally sparse (Fig. 46D); sides of pronotal disk with distinct coarse punctures	*Operclipygus bidessois* (Marseul)
–	Apical margin of elytron lacking punctures; pygidium with moderately dense ground punctation; frontal stria interrupted over antennal bases; sides of pronotum with only faint lateral punctures (Fig. 47F)	*Operclipygus petrovi* sp. n.

### Operclipygus farctus

(Marseul, 1864)

http://species-id.net/wiki/Operclipygus_farctus

**Map 17.**
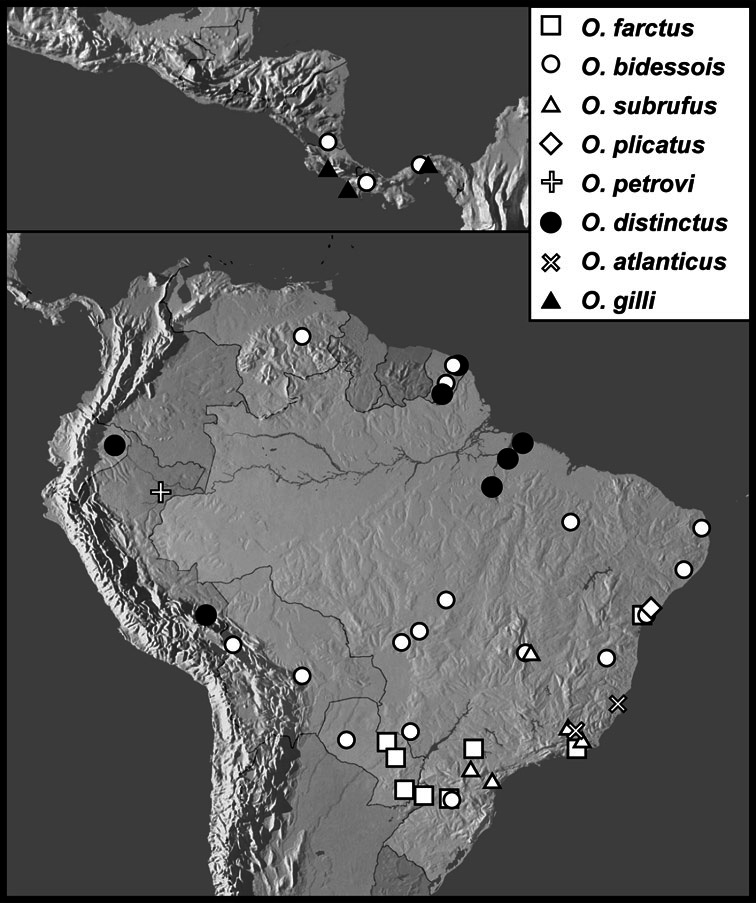
Records of the *Operclipygus farctus* group.

Figs 5A 46A–C 48A–D, K Map 17

Phelister farctus Marseul, 1864: 319; *Operclipygus farctus*: Wenzel (1976: 258).

#### Type locality.

Not specified beyond Brazil.

#### Type materal.

**Lectotype**, here designated: type “*Phelister farctus* M., Bresil” / “LECTOTYPE *Phelister farctus* Marseul, 1864 M.S.Caterino & A.K.Tishechkin des. 2010” (MNHN). **Paralectotype**: “Bresil” / “*farctus*” / “G.Lewis Coll. B.M.1926-369” / “PARALECTOTYPE *Phelister farctus* Marseul M.S.Caterino & A.K.Tishechkin des. 2010” (BMNH). This species was described from an unspecified number of specimens, and the lectotype designation fixes primary type status on one of two known syntypes.

#### Other material.

**ARGENTINA: Misiones:** 2: 15km SE Puerto Iguazú, 27.xii-6.i.1991, FIT, mature forest, roadside, S. & J. Peck (FMNH); 1: Parque Nac. Iguazú, Cantera, 200m, 8.xii-6.i.1991, FIT, old gravel pit at forest edge, S. & J. Peck (FMNH). **BRAZIL: Bahia**: 5: G. Lewis (BMNH); **Paraná:** 1: Ibipora, Faz. Doralice, 3.xi.2006, A.A. Santos (UFPR); **Rio de Janeiro:** 1: Boa Sorta, J. Schmidt (ZMHB); **Santa Catarina**: 1: Nova Teutonia, 10.x.1955, beneath leaves, F. Plaumann (FMNH), 2: 30.xi.1955, F. Plaumann (FMNH); 1: Rio Grande, tabacco, G. Lewis (BMNH). **PARAGUAY: Caazapá:** 1: San Rafael Reserve, Estancia Condesa/Toro Blanco, 26°19'11"S, 55°39'57"W, 8–10.xii.2000, FIT, Z. Falin (SEMC); 2: San Rafael Reserve, Hermosa, prop. Lopez family, 26°18'29"S, 55°45'3"W, 80m, 1–3.xii.2000, FIT, Z. Falin (CMNC); 3: San Rafael Reserve, Hermosa, Sosa family, 26°19'15"S, 55°44'55"W, 3–6.xii.2000, FIT, Z. Falin (SEMC, MSCC); **Concepción**: 1: Estancia Cororo, 26–30.xi.1999, trap with cow entrails, C. Aguilar; **Itapúa**: 1: San Rafael Reserve, Yataí, prop. Hostettler family, 26°38'17"S, 55°39'56"W, 100m, 25–26.xi.2000, FIT, Z. Falin (SEMC); 4: Karonay, 17mi W. San Rafael Reserve, 26°45'53"S, 55°50'37"W, 90–110m, 18–20.x.2000, FIT, Z. Falin (SEMC, AKTC, CMNC); 1: **San Pedro**, Cororo, 23°28'N, 56°39'W, 180m, 27–30.x.1999, J. Jensen (CHND).

#### Diagnostic description.

Length: 1.62–1.87 mm, width: 1.44–1.59 mm; body rufobrunneus, broadly oval, not too strongly depressed; frons and base of epistoma rather narrowly depressed at middle; frontal stria complete, sinuate above antennal bases and attached at sides; supraorbital stria present only as median fragment, detached from frontal; labrum about twice as wide as long, with slightly asymmetrical, weak median protuberance; left mandible untoothed, right mandible with small, subacute basal tooth; pronotal disk basally depressed but lacking prescutellar impression or plicae, with very fine sparse ground punctation, and ~15 coarser punctures in rather well-defined band near sides; marginal pronotal stria generally interrupted behind head, occasionally few fragments present; lateral submarginal stria continuous with anterior submarginal; pronotal gland openings both along anterior margin, median pair behind eyes, in front of postocular angle in submarginal stria, the other midway between median opening and anterior pronotal angle; elytra with one complete epipleural stria, outer subhumeral stria present in apical half, inner subhumeral stria absent, the interval between outer subhumeral and 1^st^ dorsal striae moderately strongly swollen, striae 1-3 complete, 4^th^ stria present in apical half, 5^th^ stria present in apical third, sutural stria present in apical two-thirds; prosternal keel broad, flat, truncate to very weakly emarginate at base, carinal striae complete, convergent, united in front; mesoventrite weakly projecting at middle, marginal stria complete, rarely narrowly interrupted; mesometaventral stria broadly arched forward to middle of mesoventral disk, continued at sides by lateral metaventral stria toward outer third of metacoxa; 1^st^ abdominal ventrite with complete inner lateral stria, outer stria often present, abbreviated between inner stria and metacoxa, central portion of ventrite with few small punctures posterolaterally; propygidium with conspicuous fine ground punctation along sides and posterior margin, with coarser round secondary punctures dense, separated by about half their diameters; pygidium with dense but rather shallow ground punctation, with small secondary punctures uniformly interspersed; marginal pygidial sulcus fine, weakly crenulate, nearly complete but usually obsolete in basal corners. Male genitalia (Figs 48A–D, K): accessory sclerites present; T8 elongate, sides weakly convergent in basal two-thirds, bent in to apex at weakly desclerotized corner (notched at this point in lateral view), apices acute, with narrow median emargination, with deep, rather narrow basal emargination that does not reach basal membrane attachment line, ventrolateral apodemes symmetrical, not more strongly developed basally, separated beneath by about one-fifth T8 width; S8 with sides subparallel to slightly convergent, with apical guides narrow, slightly enlarged along inner edge at apex, separate, weakly divergent along midline; T9 subparallel in basal third, sides evenly convergent to narrow, opposing apices; T10 with halves separate; S9 elongate, narrowest near apex, stem weakly widened to rounded base, with broad head lacking median emargination, apical flange entire; tegmen widest just basad middle, strongly narrowed to subacute apex, apex rather abruptly bent downward in apical third, with medioventral process narrowly ‘U’-shaped, projecting beneath about one-fourth from base; median lobe about one-third tegmen length, with filamentous portions of proximal apodemes inconspicuous; basal piece nearly half as long as tegmen.

#### Remarks.

This small species is fairly generalized, and exhibits a combination of mostly apparently plesiomorphic states. It can be recognized by the combination of a barely abbreviated, fine marginal pygidial sulcus (Fig. 46C), along with its complete marginal mesoventral stria (Fig. 46B), moderately large, dense punctures of the propygidium (Fig. 46C), and presence of several small punctures along the posterior margin of abdominal ventrite 1 (Fig. 46B). It lacks several distinctive characters of *Operclipygus bidessois*, including pronotal plicae and apical elytral punctures.

### Operclipygus bidessois

(Marseul, 1889)

http://species-id.net/wiki/Operclipygus_bidessois

Fig. 46D Map 17

Phelister bidessois Marseul, 1889: 147; *Operclipygus bidessois*: Wenzel (1976: 257).
Phelister subplicatus Schmidt, 1893b: 88; *Operclipygus subplicatus*: Wenzel (1976: 260); syn. n.

#### Type locality.

Not specified beyond Brazil.

#### Type material.

***Phelister bidessois*: Lectotype**, here designated: [with red ‘type’ disk] “Braz.”/ “*Phelister bidessois* Type. Mars”[in Lewis’ writing]/ “*bidessois*” / “Marseul 14.12.86” / “Brazil, coll. Saunders” / “LECTOTYPE *Phelister bidessois* Marseul M.S.Caterino & A.K.Tishechkin des. 2010” (BMNH). This species was described from an unspecified number of specimens, and the lectotype designation fixes primary type status on the only known original specimen.***Phelister subplicatus*: Lectotype**, here designated: “Bahia” / “Type” / “*Operclipygus subplicatus* (Schmidt) N. Degallier” / “LECTOTYPE *Phelister subplicatus* Schmidt M.S.Caterino & A.K.Tishechkin des. 2010” (ZMHB). This species was described from an unspecified number of specimens, and the lectotype designation fixes primary type status on the only known original specimen.

#### Other material.

**BOLIVIA: La Paz**: 1: Tumupasa, 1921–1922, W.M. Mann (USNM); **Santa Cruz:** 5: 3.7km SSE Buena Vista, Flora y Fauna Hotel, 17°29.9'S, 63°33.2'W, 400–440m, 2–4.xi.2002, FIT, R. Leschen (AKTC, MSCC), 3: 14–16.x.2000, R. Morris (AKTC). **BRAZIL: Distrito Federal**: 4: Brasilia, 15°47'S, 47°55'W, 15–30.iii.1997, cerrado, dead fish bait (CHND, UFPR); 4: Res. Ecol. de IBGE, Brasilia, 15°5.5'S, 47°53'W, 6.i.1997 (CHND), 3: 20.i.1998 (CHND), 5: 30.ix.1997 (CHND), 1: 22.xii.1997 (CHND); **Maranhão**: 1: Mirador, Caicarinha, 6°22'S, 44°22'W, 1.v.1993, FIT (CHND); **Mato Grosso:** 1: Querencia, Fazenda São Luiz, 12°35.819'S, 52°22.492'W, ii.2009, FIT, R. Andrade (CEMT); 1: Diamantino, Serra do Tombador, 14°36'47"S, 56°15'12"W, 450m, 14.i.2001, FIT, cerrado forest, F. Genier & F. Vaz-de-Mello (CMNC); 1: Diamantino, Vale da Solidao, 14°21.63'S, 56°7.4'W, 640m, 22.xii–26.i.2009, FIT, D.C.T. Oliveira (CEMT); 1: Diamantino, Faz. São Joao, 14°23'38"S, 56°08'50"W, 520m, 13.i.2001, cerrado, carrion, F. Genier & F. Vaz-de-Mello (CMNC); 2: Cuiabá, Fazenda Mutuca, 15.3145°S, 55.9703°W, 6.xii.2008, FIT, F.H. Gava & J.R. Rocha, 2: 20.xii.2008, 2: 11.x.2008, 1: 20.iii.2009, 2: 18.x.2008, 2: 25.x.2008, 1: 29.x.2008, 2: 1.xi.2008, 1: 22.xi.2008 (MSCC, AKTC, UFPR, CEMT); 1: Estac. Exper. da CEPLAC, Alta Floresta, 9°53.85'S, 56°17.1'W, 5.ii.2010, FIT, V. Goncalves (MSCC); **Mato Grosso do Sul**: 1: Itaum, 22°00'S, 55°20'W, iii.1973, malaise trap (CHND); **Minas Gerais**: 1: Pedra Azul, 700m, xi.1972, Seabra & Oliveira (UFPR); **Pará:** 1: Amazons, viii.1875 (BMNH); **Pernambuco**: 1: G. Lewis (FMNH); **Rio Grande do Norte**: 1: Natal, viii.1949, M. Alvarenga (UFPR); **Santa Catarina**: 1: Nova Teutonia, 2.iii.1951, F. Plaumann (FMNH). **FRENCH GUIANA:** 3: Le Gallion, 4°47'N, 52°26'W, 8.i.1978, dead snake bait (CHND, MNHN), 3: 12–18.xii.1977, dead snake bait (CHND); 2: Belvèdére de Saül, point de vue, 3°1'22"N, 53°12'34"W, 31.ix.2010, Window trap, SEAG (MNHN), 1: 10.xii.2010, Window trap, SEAG (MNHN). **NICARAGUA, Rio San Juan**: 1: Refugio Bartola, 60km SE San Carlos, 10°58.40'N, 84°20.30'W, 100m, 25–28.v.2002, FIT, R. Brooks, Z. Falin, S. Chatzimanolis (SEMC). **PANAMA: Chiriquí:** 1: Chorcha Abajo., 8°22'N, 82°17'W, 19–20.i.2001, pitfall, in *Orthogeomys* burrow, W. Godwin & A. Gillogly (AKTC); **Colón:** 3: Parque Nac. San Lorenzo, Achiote, Cafetal A Dist., 09°12'N, 79°58'W, 50m, 7–21.v.2007, FIT, A. Mercado (AKTC, GBFM). **PARAGUAY: Boquerón**: 1: Loma Plata, 22°23'S, 59°51'W, 15.i.1993, A. Sanchez (CHND), 3: 24.i–7.ii.1993, P. Gerlach (CHND). **VENEZUELA: Bolívar**: 2: Guri, 9.vii.1998, FIT, in dry forest, H. & A. Howden (CMNC).

#### Diagnostic description.

This species is extremely similar to *Operclipygus farctus*, differing mainly in the following characters: length: 1.68–1.87 mm, width: 1.37–1.53 mm; supraorbital stria complete and continuous with frontal stria; base of pronotum with short, distinct plicae in front of 3^rd^ elytral striae; sides of pronotal disk with more numerous punctures; elytra with 4^th^ dorsal stria varied, from present only in apical half to complete; elytral apex with numerous marginal setae, subserially arranged, often vaguely connecting apices of striae; mesoventral marginal stria interrupted by more strongly anteriorly arched mesometaventral stria; propygidium with coarse punctures shallower, subocellate, slightly sparser; pygidial ground punctation fine and sparse, with coarser punctures uniformly scattered; marginal pygidial sulcus impressed around apical margin only, obsolete from most of basal half. Male genitalia indistinguishable from those of *Operclipygus farctus* (see Figs 48A–D, K).

#### Remarks.

This species is generally very similar to *Operclipygus farctus*, above, but may be easily separated by its pronotal plicae, and very strongly abbreviated marginal pygidial sulcus (Fig. 46D).

The only characters that have been used to separate *Operclipygus subplicatus* from *Operclipygus bidessois* have been minor variations in elytral striation (particularly whether the 4^th^ dorsal stria was complete or not). This character is quite variable, and we can find no substantial differences between these types otherwise.

### Operclipygus subrufus sp. n.

urn:lsid:zoobank.org:act:7F7FED66-3AC7-4B25-A899-3FD73EB66661

http://species-id.net/wiki/Operclipygus_subrufus

Figs 47A–B Map 17

#### Type locality.

BRAZIL: Rio de Janeiro:Nova Friburgo [22°16'S, 42°32'W].

#### Type material.

**Holotype male**: “Brasil, Rio de Janeiro: Nova Friburgo, Sans Souci, 9–15/XI/2009, E. Grossi (leg.)” / “Interceptção de vôo (FIT)” / “DZUP272588”/ “*Operclipygus* Marseul, Det. F. W. T. Leivas 2010” (UFPR). **Paratypes** (4): 1: same data as type (UFPR); 3: same locality as type but 26–31.x.2009, FIT (CHND, FMNH).

#### Other material.

**BRAZIL: Distr. Federal**: 1: Reserva Ecol. de IBGE, Brasilia, 15°56.5'S, 47°53'W, x.1986, FIT, cerrado forest, I. Diniz (AKTC); **Minas Gerais**, 2: Viçosa, 20°45'S, 42°53'W, xi.2000, FIT, forest, F.Z. Vaz-de-Mello (AKTC, CEMT), x.2000 (MSCC); **Paraná:** 1: Tibagi, Parque Estad. Guartelá, 24.5663°S, 50.2570°W, 12–15.xii.2011, FIT, forest, M.S. Caterino & A.K. Tishechkin, DNA Extract MSC-2265 (SBMNH); 1: Piraquara, Mananciais da Serra, 25.4934°S, 48.9786°W, 9–11.xii.2011, FIT, F.W.T. Leivas, M.S. Caterino & A.K. Tishechkin, DNA Extract MSC-2263 (UFPR).

#### Diagnostic description.

This species is extremely similar to *Operclipygus farctus*, differing mainly in the following characters: length: 1.87–2.03 mm, width: 1.62–1.78 mm; body rufopiceous to faintly bicolored, with sides of elytra rufescent from dorsal stria 3 laterad, broad and subquadrate; base of pronotal disk strongly flattened to weakly plicate; anterior pronotal corners curved downward, nearly vertical at sides; elytra with outer subhumeral stria present in apical third, inner subhumeral stria absent, striae 1-3 complete, 4^th^ stria present in apical fourth to one-half, 5^th^ stria present in apical third, sutural stria present in apical two-thirds; meso- and metaventrites wide and short, mesoventral stria complete, mesometaventral stria broadly arched to middle of mesoventral disk; propygidium with punctures moderately small, round, separated by about their diameters; pygidium as in *Operclipygus farctus*. Male genitalia mostly similar to those of *Operclipygus farctus* (see Figs 48A–D, K), except with ventrolateral apodemes of T8 more distant ventrally, midlateral desclerotization not distinct (though it is still notched as seen in lateral view); S9 more strongly and somewhat asymmetrically expanded basally.

#### Remarks.

**Figure 47.**
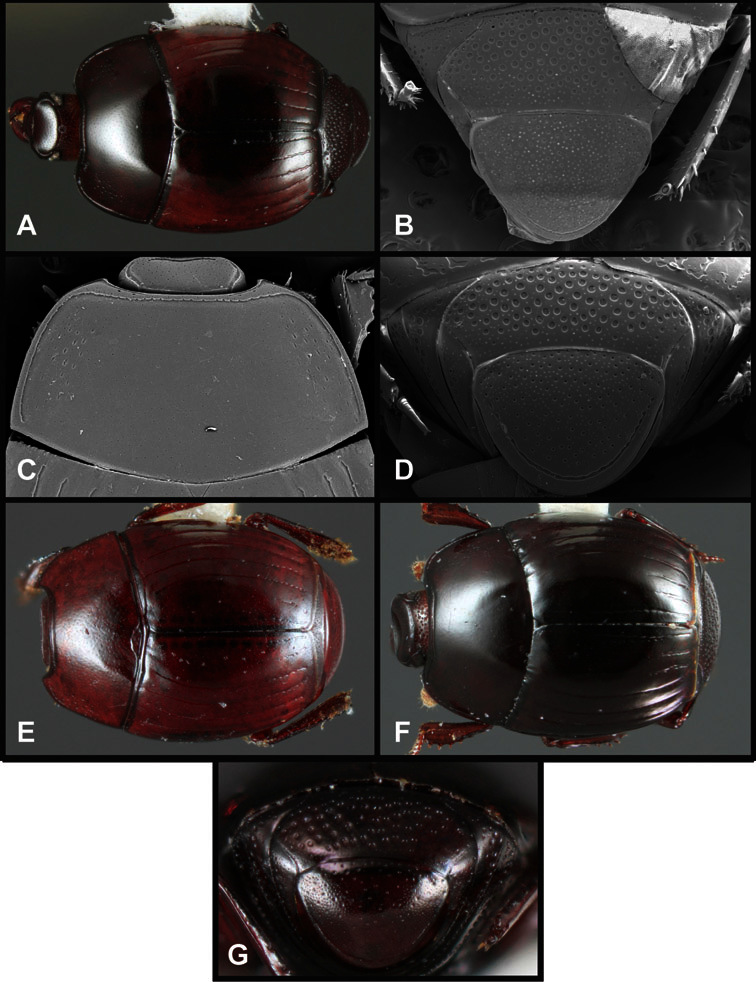
*Operclipygus farctus* group. **A** Dorsal habitus of *Operclipygus subrufus* **B** Pygidia of *Operclipygus subrufus* **C** Pronotum of *Operclipygus atlanticus* **D** Pygidia of *Operclipygus atlanticus* **E** Dorsal habitus of *Operclipygus plicatus* **F** Dorsal habitus of *Operclipygus petrovi* **G** Pygidia of *Operclipygus petrovi*.

Where bicolored (Fig. 47A), this species is easy to recognize, but nearly half of available specimens are uniformly colored. In those cases the body shape, in addition to the generally short 4^th^ dorsal stria, will distinguish it from *Operclipygus farctus*. We restrict the type series to specimens from the Nova Friburgo vicinity, as these are the only ones that consistently show the rufescent elytral coloration.

#### Etymology.

The name of this species refers to the reddish coloration of the elytra.

### Operclipygus plicatus

(Hinton, 1935) comb. n.

http://species-id.net/wiki/Operclipygus_plicatus

Fig. 47E Map 17

Phelister plicatus Hinton, 1935a: 591.

#### Type locality.

Not specified beyond Bahia State, Brazil.

#### Type material.

**Lectotype** of undetermined sex, here designated: “Bahia, A.G.” / “LECTOTYPE *Phelister plicatus* Hinton, 1935 M.S.Caterino & A.K.Tishechkin des. 2010” (BMNH). This species was described from an unspecified number of specimens, and the lectotype designation fixes primary type status on the only known original specimen.

#### Diagnostic description.

This species is extremely similar to *Operclipygus farctus* and *Operclipygus bidessois*, differing mainly in the following characters: frontal stria rather evenly, weakly rounded across front; pronotal disk with basal plicae; lateral submarginal pronotal stria present only in apical half to third, obsolete basally; anterolateral pronotal marginal gland opening nearer anterior pronotal angle; elytra with outer subhumeral stria complete, inner subhumeral represented by a fine basal scratch, striae 1-4 complete, 5^th^ stria present in apical fourth, sutural stria present in apical two-thirds; prosternum narrow, carinal striae converging strongly, parallel in apical two-thirds; connected by slightly bulbous anterior arch; posterior edge of prosternal keel emarginate but mesoventral margin not projecting; mesoventral marginal stria interrupted at middle by arched mesometaventral stria; propygidial punctures round, moderately dense basally, becoming both smaller and sparser posteriorly; pygidial punctures very fine and sparse, lacking coarser secondary punctures; marginal pygidial sulcus absent. Male not known.

#### Remarks.

This species is very similar to *Operclipygus bidessois*, particularly in the presence of plicae along the basal margin of the pronotal disk (Fig. 47E). *Operclipygus plicatus* differs primarily in the abbreviation of the lateral submarginal pronotal stria, the diminishing propygidal punctures posteriorly, and in the lack of a marginal pygidial sulcus. This species is only known from the type specimen.

### Operclipygus petrovi sp. n.

urn:lsid:zoobank.org:act:96B7B9E5-AFDF-4581-B634-CBA3725918E3

http://species-id.net/wiki/Operclipygus_petrovi

Figs 47F–G Map 17

#### Type locality.

PERU: Loreto: 20 km NNE Iquitos, Gen Gen [3°37'S, 73°18'W].

#### Type material.

**Holotype male**: “**PERU: Loreto**, 20 km NNE Iquitos, Rio Momón, Gen Gen, 120m, 5–6 Febr 2007, A.V. Petrov”/ “Caterino/Tishechkin Exosternini Voucher EXO-00319” (FMNH).

#### Diagnostic description.

**Figure 48.**
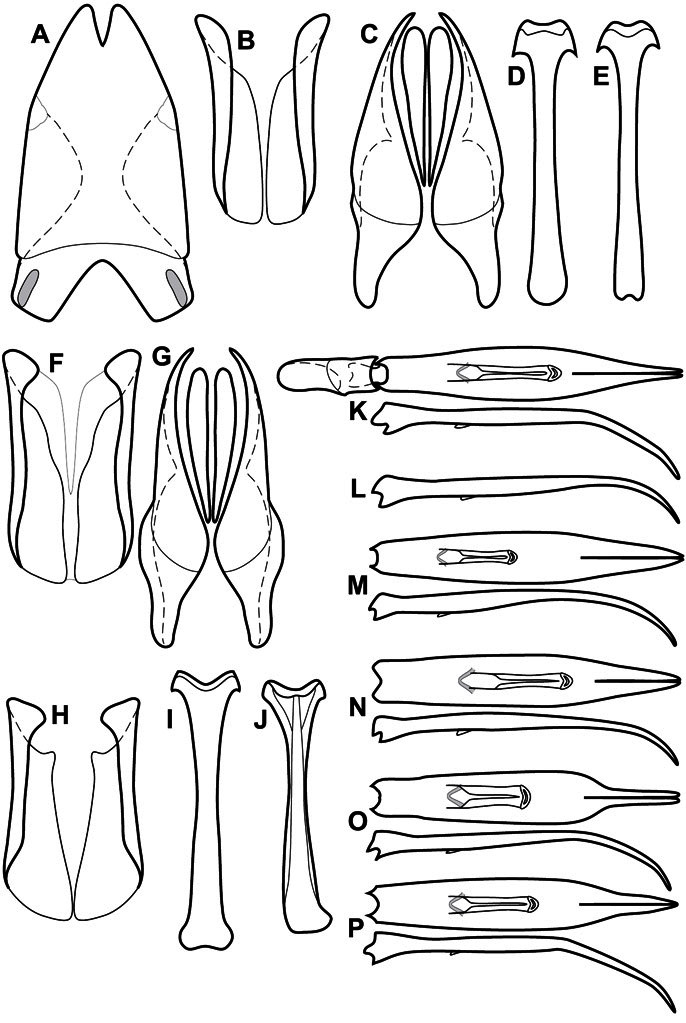
Male genitalia of *Operclipygus farctus* group. **A** T8 of *Operclipygus farctus* **B** S8 of *Operclipygus farctus* **C** T9 & T10 of *Operclipygus farctus* **D** S9 of *Operclipygus farctus* **E** S9 of *Operclipygus atlanticus* **F** S8 of *Operclipygus atlanticus* **G** T9 & T10 of *Operclipygus atlanticus* **H** S8 of *Operclipygus gilli* **I** S9 of *Operclipygus gilli* **J** S9 of *Operclipygus proximus* **K** Aedeagus, dorsal and lateral views, of *Operclipygus farctus* **L** Aedeagus, lateral view, of *Operclipygus distinctus* **M** Aedeagus, dorsal and lateral views, of *Operclipygus atlanticus* **N** Aedeagus, dorsal and lateral views, of *Operclipygus gilli* **O** Aedeagus, dorsal and lateral views, of *Operclipygus proximus* **P** Aedeagus, dorsal and lateral views, of *Operclipygus prolixus*.

Length: 1.97 mm, width: 1.65 mm; body rufo-piceous, smooth and shining, ovoid, rather strongly convex; frons depressed at middle, frontal stria divergent between eyes, central portion detached from sides; supraorbital stria fine, more or less complete, connected to frontal stria; epistoma convex, weakly emarginate at apex; pronotal disk strongly flattened across middle of base, but not plicate; disk with very fine ground punctation and few faint coarser punctures at sides; marginal pronotal stria absent behind head; lateral submarginal stria continuous with anterior submarginal stria; anterior pronotal gland openings present behind eye and midway between median opening and anterior pronotal corner; elytron with one complete epipleural stria, outer subhumeral stria present in apical half, inner subhumeral stria absent, the interval between outer subhumeral and 1^st^ dorsal stria strongly swollen, striae 1-3 complete, 4^th^ and 5^th^ striae short, present only in apical one-fifth, sutural striae present in apical two-thirds, more broadly impressed anteriorly; prosternal keel broad, flat, weakly emarginate at base, carinal striae complete, convergent, united in front; mesoventrite weakly projecting at middle, marginal stria interrupted; mesometaventral stria broadly arched forward beyond middle of mesoventral disk, continued at sides by lateral metaventral stria toward outer third of metacoxa; 1^st^ abdominal ventrite with single complete lateral stria, bent mediad at apex; propygidium with faint ground punctation, with large, shallow, elongate oval, punctures separated by about half their diameters; pygidium with dense but rather shallow ground punctation, small, coarser punctures uniformly interspersed; marginal pygidial sulcus fine, weakly crenulate, nearly complete but usually obsolete in basal corners. Male genitalia indistinguishable from those of *Operclipygus farctus* (see Figs 48A–D, K).

#### Remarks.

While this species is very similar to *Operclipygus farctus* in many characters, it is easily distinguished by its darker, more strongly convex, and rounder body form (Fig. 47F). The large, shallow punctures of the propygidium (Fig. 47G) and the absence of pronotal plicae distinguish it from some of the even more strongly convex species.

#### Etymology.

We name this species for scolytine specialist Alexander Petrov, in recognition of his numerous contributions of specimens to this study, principally from Peru.

### Operclipygus distinctus

(Hinton 1935)

http://species-id.net/wiki/Operclipygus_distinctus

Figs 48L 49A–B Map 17

Phelister distinctus Hinton, 1935a: 589; *Operclipygus distinctus*: Wenzel 1976: 257.

#### Type locality.

BRAZIL: Pará: Santarém [2°26'S, 54°42'W].

#### Type material.

**Lectotype**, here designated: “H.H. Smith” / "Santarem” / “*Phelister distinctus* Hntn.” / “LECTOTYPE *Phelister distinctus* Hinton M.S.Caterino & A.K.Tishechkin des. 2010” (BMNH). This species was described from an unspecified number of specimens, and the lectotype designation fixes primary type status on the only known original specimen.

#### Other material.

**BRAZIL: Pará:** 1:IPEAN, Utinga, Belém, 1°27'S, 48°26'W, v.1985, FIT (CHND), 1: viii.1985, FIT (CHND), 1: xi.1984, FIT (FMNH); 1: Barcarena, 1°30'S, 48°37'W, 13–25.vi.1991, FIT (MSCC); 1: Tucuruí, 3°45'S, 49°40'W, 23.vi–7.vii.1986, FIT (AKTC). **ECUADOR: Orellana:** 1:Yasuní Res. Stn., 00°40'28"S, 76°38'50"W, 215m, 5–10.ix.1999, FIT, primary forest, E.G. Riley (TAMU). **FRENCH GUIANA:** 1: Rés. Trésor, route de Kaw Pk18, 4°36.63'N, 52°16.74'W, 225m, 6.xi.2009, FIT, SEAG (MNHN); 1: Mont. Tabulaire, Itoupé, 3°1.32'N, 53°5.05'W, 800m, 17.iii.2010, FIT, SEAG (CHND); 1: Réserve des Nouragues, Régina, 4°2.27'N, 52°40.35'W, 3.xi.2009, FIT, SEAG (CHND). **PERU: Madre de Dios:** 1: Tambopata, 24.x.1982, rotten palm flowers, L. Watrous & G. Mazurek (FMNH).

#### Diagnostic description.

Length: 1.87–2.22 mm, width: 1.56–1.90 mm; body rufobrunneus to rufopiceous, elongate oval, moderately strongly convex; frons depressed, finely punctate, frontal portion of frontal stria detached from lateral; supraorbital stria complete, but narrowly detached from sides of frontal; epistoma slightly emarginate; labrum rectangular, about twice as wide as long; pronotum curved downward in anterior corners, nearly vertical on either side; pronotal disk lacking prescutellar impression, but strongly depressed across base; ground punctation of pronotal disk very fine and sparse, with ~15 shallowly impressed coarser punctures near sides; marginal pronotal stria obsolete for width of head; lateral submarginal stria complete, continuous with anterior submarginal stria; pronotal gland openings close together behind eye, between end of marginal stria and submarginal stria; elytra strongly swollen in subhumeral interval, with single complete epipleural stria, outer subhumeral stria complete, inner subhumeral stria present in apical half to three-fourths, frequently fragmented basally, rarely absent, striae 1-3 complete, 4^th^ stria present in apical half, 5^th^ stria present in apical third, sutural stria present in apical two-thirds, the sutural stria widening anteriorly; prosternal keel very weakly emarginate at base, carinal striae enclosing triangular area which is finely microsculptured, meeting anteriorly; mesoventrite faintly projecting, with complete marginal stria; mesometaventral arch nearly reaches mesoventral stria, sinuate near mesocoxae, continued by lateral metaventral stria posterolaterally toward middle of metacoxa, slightly curved outward at apex; central portion of metaventral disk weakly depressed on either side; 1^st^ abdominal ventrite with single lateral stria; propygidium with large, elongate punctures separated by about their diameters; pygidial disk strongly convex, with dense, fine ground punctation and numerous conspicuous coarser punctures interspersed; marginal pygidial sulcus distinct, complete, crenulate, with distinct series of punctures within. Male genitalia largely indistinguishable from trhose of *Operclipygus farctus* (see Figs 48A–D), except that S8 has its sides more strongly convergent, halves ventrally more strongly divergent along ventral midline, and apical guides narrower; tegmen curved ventrad in apical third(Fig. 48L), but not strongly bent as in *Operclipygus farctus*.

#### Remarks.

**Figure 49.**
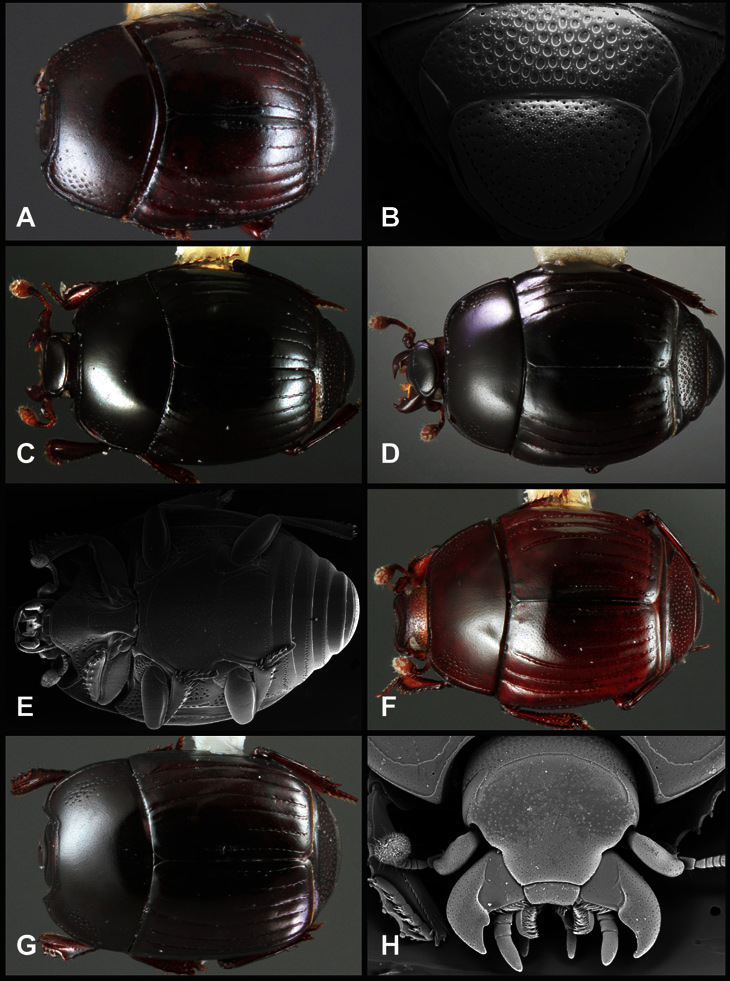
*Operclipygus farctus* group. **A** Dorsal habitus of *Operclipygus distinctus* **B** Pygidia of *Operclipygus distinctus* **C **Dorsal habitus of *Operclipygus atlanticus* **D** Dorsal habitus of *Operclipygus gilli* **E** Ventral habitus of *Operclipygus gilli* **F** Dorsal habitus of *Operclipygus proximus* **G** Dorsal habitus of *Operclipygus prolixus* **H** Frons of *Operclipygus prolixus*.

This species is the only previously described member of a small subgroup of four very similar species (including the following three), all of which have their pronotal gland openings very close together along the anterior margin. All are more or less piceous, and relatively strongly convex, with conspicuously dual punctation on the pygidium (Fig. 49B). *Operclipygus distinctus* has the most convex pygidium of the four, with the largest secondary punctures. It also lacks an outer lateral stria on the 1^st^ abdominal ventrite, which the last two species in this subgroup have.

### Operclipygus atlanticus sp. n.

urn:lsid:zoobank.org:act:866B8471-87D5-43EA-842A-25B98872F35C

http://species-id.net/wiki/Operclipygus_atlanticus

Figs 47C–D 48E–F, M 49C Map 17

#### Type locality.

BRAZIL: Minas Gerais: Viçosa [20°45'S, 42°53'W].

#### Type material.

**Holotype male**: “**BRAZIL: Minas Gerais**, Viçosa, 20°45'S, 42°53'W. October 2000, Flight intercept trap, F.Z.Vaz-de-Mello”/ “Caterino/Tishechkin Exosternini Voucher EXO-00318” (CEMT). **Paratypes** (3): same locality as type, IX.1999, E. Grossi, coll (UFPR, FMNH).

#### Other material.

1: **BRAZIL:** **Espirito Santo**: Mun. Linhares, Faz. Lagoa do Macuco, 19°03'50"S, 39°58'43"W, 10m, 27-i-2000 F. Genier & S. Ide, primary lowland Atlantic forest, FIT (CMNC).

#### Diagnostic description.

This species is very similar to the preceding, differing in a few characters as follows: length: 1.81–1.93 mm, width: 1.50–1.59 mm; body slightly less strongly convex; anterior submarginal pronotal stria more nearly straight across the front, not strongly arched into anterior corners; elytra with outer subhumeral stria present in apical half only, inner absent; propygidium with punctures more nearly round, sparser, separated by slightly more than their diameters; pygidium not strongly convex, with marginal sulcus obsolete in basal corners. Male: genitalia extremely similar to those of *Operclipygus farctus* (see Fig. 48A), except S8 (Fig. 48F) with apices of guides more strongly protuberant at inner angles; T9 (Fig. 48G) with bulbous basolateral angles; tegmen (Fig. 48M) slightly broader toward apex, more gently curved ventrad, not strongly bent.

#### Remarks.

Although very similar to *Operclipygus distinctus*, this species can be distinguished from it by its abbreviated outer subhumeral stria, less strongly convex pygidium, and submarginal pronotal stria (Fig. 49C) which is nearly transverse across entire front of pronotum. The single specimen from Espirito Santo differs slightly in having the male S8 widening slightly toward the apex, and the head of S9 more robust. Therefore we exclude it from the type series.

#### Etymology.

This species’ name refers to its occurrence only in the threatened Atlantic forests of southeastern Brazil.

### Operclipygus gilli sp. n.

urn:lsid:zoobank.org:act:A0FA8977-81C4-41A4-A7CC-4184DCB73A01

http://species-id.net/wiki/Operclipygus_gilli

Figs 4A 48H–I, N 49D–E Map 17

#### Type locality.

PANAMA: Panamá: Chepo-Carti Road [9.28°N, 79.1°W].

#### Type material.

**Holotype male**: “**PANAMA: Panamá** Chepo-Carti Rd. 400m. June 1982, FIT. B.D. Gill”/ “Caterino/Tishechkin Exosternini Voucher EXO-00301” (CNCI). **Paratypes** (28): 4: same data as type (FMNH, MSCC, AKTC); 2: same locality as type but: vii.1982 (CHSM, BDGC). **COSTA RICA: Puntarenas:** 1: Res. Biol. Carara, Est. Carara, 200m, iv.1990, R. Zuniga, (INBIO); 1: Res. Biol. Carara, Est. Quebrada Bonita, 50m, iv.1994, R. Guzman, (INBIO); 2: Rancho Quemado, Peninsula de Osa, 200m, ix.1992, F. Quesada, (INBIO), 1: ix.1992, M. Segura, (INBIO), 1: ix.1992, A. Marin, (INBIO), 1: v.1992, F. Quesada y G. Varela, (INBIO), 2: viii.1991, F. Quesada, (INBIO), 1: 12–24.v.1993, A. Gutierrez, (INBIO), 1: 7–27.i.1992, A.H. Gutierrez, (INBIO); 1: Reserva Forestal Golfo Dulce, Est. Agujas, Golfito, Sendero Zamia, 300m, 5.x.2000, primary forest, A. Azofeifa (INBIO), 3: 3.v.1998, FIT, A. Azofeifa (INBIO), 1: 2–15.i.1996, FIT, A. Azofeifa, (INBIO), 1: 250–350m, 15.iv.2000, FIT, A. Azofeifa (INBIO), 1: 250–350m, 19.v.2000, FIT, A. Azofeifa (INBIO), 1: 9.iv.2000, FIT, A. Azofeifa (INBIO), 1: 11–15.xi.1999, FIT, A. Azofeifa (INBIO); 1: Parque Nac. Corcovado, Sirena Stn. Rio Pavo Trail, 8°29'5"N, 83°35'33"W, 5m, 25–28.vi.2000, FIT, Z. Falin (SEMC); 1: Rincon de Osa, 8°41.141'N, 83°31.117'W, 150m, 23–26.vi.2001, FIT, S. & J. Peck (SEMC).

#### Diagnostic description.

This species is very similar to *Operclipygus distinctus*, differing in a few characters as follows: length: 1.84–2.25 mm, width: 1.56–1.90 mm; elytra with outer subhumeral stria present only in apical half, inner subhumeral stria absent; prosternal keel not microsculptured between carinal striae; marginal mesoventral stria interrupted at middle; metaventral disk not depressed on each side of midline; 1^st^ abdominal ventrite with inner lateral stria complete, outer lateral stria finely impressed along inner edge of metacoxa (sometimes obsolete there), curving behind it; coarse pygidial punctures slightly smaller. Male genitalia very similar to those of *Operclipygus farctus*, except S8 (Fig. 48H) with apical guides more strongly developed at inner edge; S9 (Fig. 48I) with strong, abrupt basal expansion, apex lacking midline sclerotization, but with strong lateral marginal sclerotizations forming a triangular ‘window’ of thin cuticle near apex; tegmen (Fig. 48N) with sides subparallel in basal two-thirds, rather abruptly narrowing about one-fourth from apex, apex not strongly bent ventrad.

#### Remarks.

Within the subgroup of species near *Operclipygus distinctus*, *Operclipygus gilli* can be identified on geography alone, being the only one occurring in Central America. Within the larger *Operclipygus farctus* group the only sympatric species is *Operclipygus bidessois*, which is easily distinguished by the smaller, subdepressed, elongate body form. *Operclipygus gilli* and *Operclipygus proximus* both possess an outer lateral stria on the 1^st^ abdominal ventrite that curves laterad behind the metacoxa (Fig. 49E), though in *Operclipygus gilli* its anterior end does not reach the basal margin of the ventrite. An additional difference is that *Operclipygus gilli* has the base of the pronotum flattened but not distinctly plicate (Fig. 49D).

#### Etymology.

This species is named for Bruce Gill, whose collections in the Neotropics have enriched this study greatly.

### Operclipygus proximus sp. n.

urn:lsid:zoobank.org:act:6537BFAC-1411-4DF0-95D2-D2B53ACA732F

http://species-id.net/wiki/Operclipygus_proximus

**Map 18.**
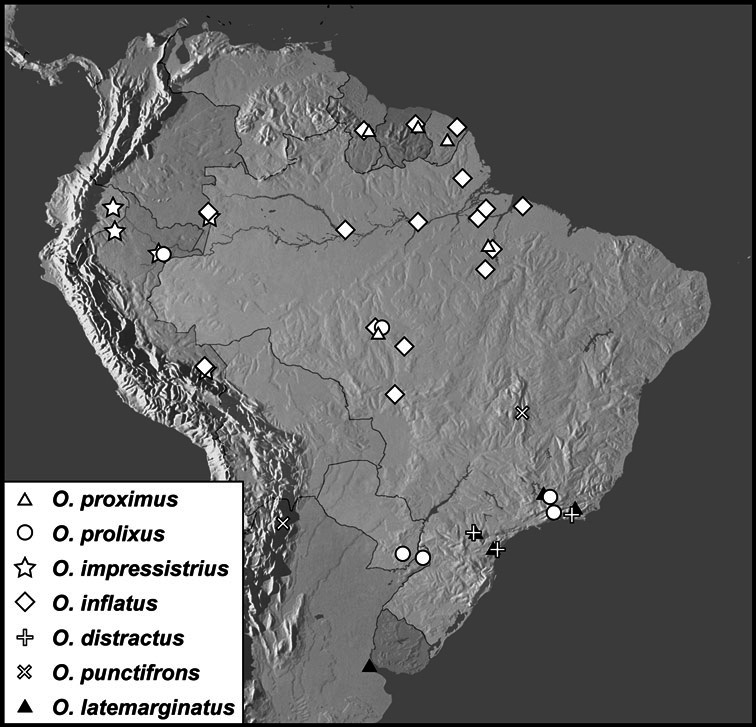
Records of the *Operclipygus farctus* group.

Figs 48J, O 49F Map 18

#### Type locality.

SURINAME: Brokopondo: Brownsberg Nature Preserve [4°56.9'N, 55°10.9'W].

#### Type material.

**Holotype male**: “SURINAME: Brokopondo, Brownsberg Nature Preserve, Witi Creek Trail, 340m, 4°56'55"N, 55°10'53"W, 23–25 JUN 1999; Z.H. Falin, A. Gangadin, H. Hiwat, SUR1F99 115; ex: flight intercept trap”/ “SM0178062 KUNHM-ENT” (SEMC). **Paratypes** (2): **FRENCH GUIANA:** 1: Belvèdére de Saül, point de vue, 3°1'22"N, 53°12'34"W, 13.v.2011, polytrap, SEAG (MNHN). **GUYANA: Region 8**: 1: Iwokrama Field Stn., Iwokrama Forest, 1km W Kurupukari, 4°40'19"N, 58°41'4"W, 60m, 20–25.v.2001, FIT, R. Brooks & Z. Falin (SEMC).

#### Other material.

**BRAZIL: Mato Grosso:** 1: Cotriguaçu, Fazenda São Nicolau, Matinha, 9°50.3'S, 58°15.05'W, xii.2009, FIT, F.Z. Vaz-de-Mello (CEMT), 1: xii.2010, FIT, F.Z. Vaz-de-Mello (AKTC); 2: Cotriguaçu, Fazenda São Nicolau, Mata Norte, 9°49.15'S, 58°15.6'W, 8–14.xii.2010, FIT, F.Z. Vaz-de-Mello (MSCC, FMNH); 2: **Pará:** Tucuruí, 3°45'S, 49°40'W, 24.iii–11.iv.1988, FIT (CHND).

#### Diagnostic description.

This species is very similar to *Operclipygus distinctus*, as well as *Operclipygus gilli*, differing in a few characters as follows: length: 2.15–2.50 mm, width: 1.81–2.15 mm; base of prothorax with distinct plicae; elytra with outer subhumeral stria complete, inner subhumeral stria absent; prosternal keel without microsculpture between carinal striae; mesoventrite with marginal stria complete; fragments of secondary lateral metaventral stria visible on sides of metaventrite; 1^st^ abdominal ventrite with inner lateral stria complete, outer lateral stria well-impressed, present along inner edge of metacoxa and curving behind it as a distinct stria; pygidium not so strongly convex, with secondary punctures relatively small. Male genitalia very similar to those of *Operclipygus gilli*, above (see Figs 48A, H), (which are in turn indistinguishable from those of *Operclipygus farctus* in many respects), except basal expansion of S9 (Fig. 48J) markedly asymmetrical, not emarginate, apical midline sclerotization well developed, elevated above sides; tegmen (Fig. 48O) with sides subparallel in middle, abruptly narrowed to base and much more strongly narrowed to apices.

#### Remarks.

This species and *Operclipygus gilli* are very similar and closely related. Aside from the marked differences in aedeagus shape, the best way to separate the two is by the presence of distinct pronotal plicae (Fig. 49F), and the complete outer subhumeral stria in *Operclipygus proximus*. We restrict the type series to Guianan localities, due to some variability, and a largely disjunct distribution overall.

#### Etymology.

This species’ name refers to the close proximity of the two median pronotal gland openings on the anterior pronotal margin (not, it should be emphasized, unique to this species).

### Operclipygus prolixus sp. n.

urn:lsid:zoobank.org:act:7F64729D-ECDB-4457-9BB9-7839C5C315A6

http://species-id.net/wiki/Operclipygus_prolixus

Figs 48P 49G–H Map 18

#### Type locality.

BRASIL: Minas Gerais:Boqueirão Reserve near Lavras [21°14'S, 45°0'W]

#### Type material.

**Holotype male**: “**BRASIL: Minas Gerais,** Municip. Ingaí, Reserva Boqueirão nr. Lavras. F.I.T., gallery forest. 29 Oct 2002, F.Freiro-Costa & F.Z.Vaz-de-Mello collrs.”/ “Caterino/Tishechkin Exosternini Voucher EXO-00796” (CEMT). **Paratypes** (5): 2: same data as type (FMNH, UFPR); same data as type, except as noted:1: 13.xi.2002 (CEMT), 2: 4–11.xii.2002, FIT, R.J. Silva (MSCC, AKTC).

#### Other material.

**ARGENTINA: Misiones:** 2: 15km SE Puerto Iguazú, 27.xii-6.i.1991, FIT, mature forest, roadside, S. & J. Peck (FMNH); 1: Parque Nac. Iguazú, Puerto Canaos, 180m, 8.xii–6.i.1991, FIT, riparian forest, S. & J. Peck (FMNH). **BRAZIL: Mato Grosso:** 1: Cotriguaçu, Fazenda São Nicolau, Mata Norte, 9°49.15'S, 58°15.6'W, 8–14.xii.2010, FIT, F.Z. Vaz-de-Mello (CEMT); **São Paulo**: 1: Cipo, 7.ix.1970, V.N. Alin (FMNH). **PARAGUAY: Caazapá:** 1: San Rafael Reserve, Hermosa, prop. Sosa family, 26°19'15"S, 55°44'55"W, 90m, 3–6.xii.2000, FIT, Z. Falin (SEMC); **Itapúa**: 1: San Rafael Reserve, San Pedro Mt., 26°31'24"S, 55°48'18"W, 90m, 29.xi.2000, beating treefall, Z. Falin (SEMC); 2: San Rafael Reserve, Yataí, prop. Hostettler family, 26°38'17"S, 55°39'50"W, 100m, 21–25.xi.2000, FIT, Z. Falin (SEMC), 2: 26–30.ix.2000, FIT, Z. Falin (CMNC); 3: San Rafael Reserve, Karonay, 17km W, 26°45'53"S, 55°50'37"W, 90–110m, 18–20.xi.2000, FIT, Z. Falin (SEMC). **PERU: Loreto:** 1: km 63, rd. Iquitos - Nauta, Rio Itaya, 4°15.205'S, 73°26.074'W, 140m, 9–13.i.2011, A.V. Petrov (AKTC).

#### Diagnostic description.

Length: 2.22–2.65 mm, width: 1.84–2.12 mm; body piceous, elongate oval, sides of elytra nearly parallel, sides of pronotum curving inward in front, depressed; frons depressed in middle, sides of frontal striae rounded, ending above antennal cavity, absent across front; supraorbital stria complete, narrowly detached from sides of frontal stria; labrum about 2.5× as wide as long, weakly asymmetrical at apex, left side more strongly produced; left mandible untoothed, right mandible with small, subacute basal tooth; pronotal disk faintly and narrowly impressed in prescutellar region, not broadly flattened or plicate, ground punctation fine and sparse, with numerous punctures in lateral band; marginal stria absent behind head; lateral submarginal stria continuous with anterior submarginal stria, diverging slightly from margin behind median pronotal gland opening; anterolateral gland opening closer to pronotal corner than to median opening; elytron with single complete epipleural stria, outer subhumeral stria interrupted at middle, basal fragment variably abbreviated, inner subhumeral stria nearly complete, barely abbreviated at both ends, striae 1-3 complete, 4^th^ stria present in apical half to two-thirds, 5^th^ stria present in apical half, sutural stria present in apical two-thirds; elytral disk usually with distinct series of very small apical marginal punctures; prosternal keel evenly but weakly produced at base, carinal stria complete, connected anteriorly and posteriorly; mesoventral margin broadly and shallowly emarginate, with complete marginal stria; mesometaventral stria subangulately arched forward at middle, reaching middle of mesoventral disk, continued posterad by lateral metaventral stria, extending to middle of metacoxa; 1^st^ abdominal ventrite with inner lateral stria complete, outer abbreviated; propygidium with rather sparse ground punctation, coarser punctures nearly round, irregularly separated by about their diameters or slightly less; pygidium with fine, dense ground punctation, slightly coarser punctures interspersed mainly near base, frequently inconspicuous; marginal pygidial sulcus quite varied, generally fine, obsolete toward base, frequently deep apically, obsolete in basal half. Male: genitalia largely similar to those of *Operclipygus farctus* (see Figs 48A–D), except T8 with midlateral desclerotization not distinct; S9 narrower at base, not obviously asymmetrical; tegmen (Fig. 48P) not as strongly narrowed to base, apices not as strongly narrowed, apical third more strongly bend ventrad.

#### Remarks.

This species is easily recognized in the *farctus* group by the absence of the central part of the frontal stria. It is also a bit larger, more elongate, and subdepressed than most of the species in this group. It is, however, quite variable in some other characters, particularly in the extent of the subhumeral striae, the density of coarse pygidial punctures, and of length of the marginal pygidial sulcus, and for this reason we limit the type series to a small region.

#### Etymology.

This species’ name means ‘lengthy’, though generally more in the sense of verbose than elongate, as we imply here.

### Operclipygus impressistrius sp. n.

urn:lsid:zoobank.org:act:99DF8C57-860E-488B-BD01-4B0DBC749A57

http://species-id.net/wiki/Operclipygus_impressistrius

Fig. 50A Map 18

#### Type locality.

ECUADOR: Orellana:Yasuní Research Station [0°40.5'S, 76°24'W].

**Type material. Holotype male**: “**ECUADOR:** Napo, mid.Rio Tiputini, Yasuní Res. Stn. 0°40.5'S, 76°24'W, FIT #M1, 18–20 Jun 1999. AKT#008, C.Carlton & A.Tishechkin”/ “LSAM0013188” / “*Operclipygus* sp #6, Hist 128, Yasuní NP Inventory A.K.Tishechkin det 2010” (FMNH). **Paratypes** (61): 1: same data as type; same data as type, except as noted: 4: 20–26.vii.1999 (LSAM), 6: 12–20.vii.1999 (LSAM), 4: 5–12.vii.1999 (LSAM), 5: 22–28.v.1999 (LSAM), 6: 26.vii-4.viii.1999 (LSAM, CHND), 6: 28.vi-5.vii.1999 (LSAM, MSCC, AKTC), 1: 20–29.v.1999, C.E. Carlton & V. Moseley (LSAM), 3: 26.vii.1999, *Couratari guianensis* Aublet flowerfall (LSAM), 1: 22.vii.1999, *Sterculia apetala* (Jaquin) Karsten flowerfall (LSAM); 6: Parque Nac. Yasuní, Via Maxus at Puente Piraña, 0°39.5'S, 76°26'W, 14–20.vii.2008, FIT, A.K. Tishechkin (AKTC), 1: 20–24.vii.2008, FIT, A.K. Tishechkin (AKTC); 1: Yasuní Res. Stn., 00°40'28"S, 76°38'50"W, 215m, 5–10.ix.1999, FIT, primary forest, E.G. Riley (TAMU); 1: Tiputini Biodiversity Station, 0.6376°S, 76.1499°W, 4–9.vi.2011, FIT, M.S. Caterino & A.K. Tishechkin, DNA Extract MSC-2176 (SBMNH), 3: 2–9.vi.2011, FIT, M.S. Caterino & A.K. Tishechkin (USFQ, SBMNH); 1: 0°38'0"S, 76°9'0"W, 220m, 5–25.ix.2000, D.J. Inward & K.A. Jackson (BMNH). **COLOMBIA: Vaupés:** 3: Parque Nac. Mosiro-Itajura (Caparú), Centro Ambiental, 1°04'S, 69°31'W, 60m, 20–30.i.2003, FIT, D. Arias & M. Sharkey (IAVH). **PERU: Loreto:** 1: 1.5km N Teniente Lopez, 2°35.66'S, 76°06.92'W, 210–240m, 23.vii.1993, flowerfall, R. Leschen (SEMC).,2: 18.vii.1993, FIT, R. Leschen (SEMC); 2: Teniente Lopez, 2°35.66'S, 76°06.92'W, 210–240m, 25.vii.1993, flowerfall berlese, R. Leschen (SEMC); 1: 68km SW Iquitos to Nauta, Rio Itaya, 4°11'S, 73°26'W, 110m, 1–3.iii.2008, A.V. Petrov (MUSM); 1: km 63, rd. Iquitos - Nauta, Rio Itaya, 4°15.205'S, 73°26.074'W, 140m, 10–14.ii.2010, A.V. Petrov (AKTC); **Madre de Dios:** 1: Manu National Park, Pantiacolla Lodge, Alto Madre de Dios R., 12°39.3'S, 71°13.9'W, 420m, 14–19.xi.2007, FIT, D. Brzoska (SEMC).

#### Diagnostic description.

This species is generally similar to *Operclipygus distinctus*, being piceous, convex, broadly rounded, and is only described to the extent that they differ: length: 1.81–2.31 mm, width: 1.53–1.97 mm; pronotal disk with base strongly flattened and weakly plicate, with ~12 faintly impressed lateral punctures; submarginal pronotal stria not markedly displaced behind median pronotal gland openings; elytral striae 1-5 deeply and more broadly impressed at apices, with intervals strongly convex; outer subhumeral stria interrupted at middle, with basal fragment short, not reaching base, striae 1-3 complete, somewhat crowded to side, 4^th^ stria present in apical two-thirds, 5^th^ stria present in apical third, sutural stria present in apical two-thirds; prosternal keel broad, flat, weakly emarginate at base, carinal striae well separated, weakly convergent to broad anterior arch; mesometaventral stria strongly arched forward to near mesoventral margin, frequently but not always interrupting marginal mesoventral stria; 1^st^ abdominal ventrite with inner lateral stria only, curving mediad toward apex; propygidium with shallow, elongate, ovoid punctures separated by about half their diameters; pygidium with fine, dense but shallow ground punctation, very faint coarser punctures interspersed; marginal pygidial sulcus fine, complete. Male genitalia more or less indistinguishable from those of *Operclipygus distinctus* (see Figs 48A–D, L), except S8 longer, sides more parallel, apical guides more evenly and strongly developed along length; tegmen identical.

#### Remarks. 

**Figure 50.**
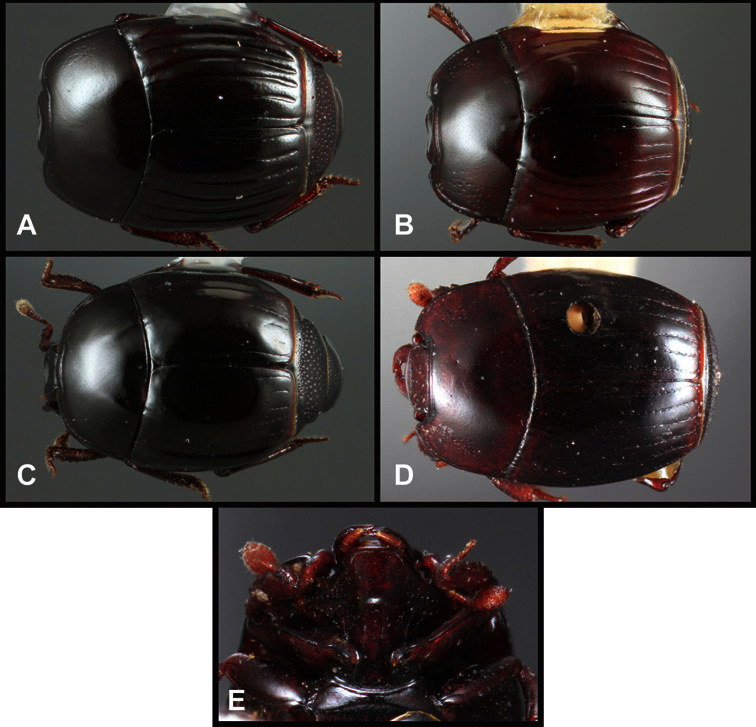
*Operclipygus farctus* group. **A** Dorsal habitus of *Operclipygus impressistrius* **B** Dorsal habitus of *Operclipygus farctissimus* **C** Dorsal habitus of *Operclipygus inflatus* **D** Dorsal habitus of *Operclipygus distractus* (lectotype) **E** Prosternum of *Operclipygus distractus*

In addition to its strongly rounded, convex body form(Fig. 50A), common to several species in this group, this species is distinguished by the elytral striae which are strongly impressed apically, the weakly plicate pronotal base, and the interrupted outer subhumeral stria.

#### Etymology.

The name of this species refers to its strongly impressed elytral striae.

### Operclipygus farctissimus sp. n.

urn:lsid:zoobank.org:act:3F8D76FD-A16B-4768-B100-FA6474269DC5

http://species-id.net/wiki/Operclipygus_farctissimus

Fig. 50B

#### Type locality.

Not specified beyond Brazil.

#### Type material.

**Holotype male**: “Braz.” [handwritten]/ “Caterino/Tishechkin Exosternini Voucher EXO-00303” (BMNH).

#### Diagnostic description.

As with the preceding, this species is very similar to *Operclipygus distinctus*, except as follows: length: 2.03 mm, width: 1.87 mm; body piceous with elytra faintly rufopiceous; frontal stria very nearly complete, only narrowly interrupted above antennal bases; antennal bossae weakly produced; pronotal disk with strong basal plicae, extending forward about one-third pronotal length; central part of anterior pronotal margin very weakly projecting at middle; elytral striae not very strongly impressed, but striae 1-5 widened slightly to apex, outer subhumeral stria present in apical half only, inner subhumeral stria absent, striae 1-3 complete, 4^th^ stria present in apical third, 5^th^ stria present in apical fourth, sutural stria present in apical two-thirds; prosternal keel very broad, emarginate at base, carinal striae strongly divergent basally; mesoventrite weakly projecting, marginal stria interrupted; mesometaventral stria arched forward at middle, sinuate near mesocoxa, extending posterad toward inner third of metacoxa; propygidium with rather small, round punctures separated by about their diameters, finer ground punctures most evident toward sides and posterior margin; pygidium with fine, dense ground punctation, and few slightly coarser punctures near basal margin; marginal pygidial sulcus fine, obsolete in basal half. Male genitalia mostly similar to those of *Operclipygus farctus* (see Figs 48A–D, K), except that the ventrolateral apodemes of T8 are more distant ventrally, with midlateral desclerotization not distinct (though it is still notched as seen in lateral view); S9 more strongly and somewhat asymmetrically expanded basally, indistinguishable from that of *Operclipygus subrufus*.

#### Remarks.

This species is relatively easy to distinguish in this group, with a broadly rounded, convex, faintly bicolored body(Fig. 50B), strong pronotal plicae, and very weakly projecting anterior pronotal margin. It is only known from the single type specimen, with an unfortunately imprecise type locality.

#### Etymology.

This species’ name refers to its relationship with *Operclipygus farctus*, but also to its more broadly rounded, convex (‘farctate’) form.

### Operclipygus inflatus sp. n.

urn:lsid:zoobank.org:act:AA7558AF-4E96-4598-87F0-578403D60237

http://species-id.net/wiki/Operclipygus_inflatus

Figs 50C 51A–C Map 18

#### Type locality.

BRASIL: Pará:Belém, Utinga [1°27'S, 48°26'W].

#### Type material.

**Holotype male**: “**BRASIL: Pará:** Belem, Utinga (IPEAN) 1°27'S, 48°26'W Piège d'interception. ix.1985” / “EXO-01628 (UFPR). **Paratypes** (48): **BRAZIL: Amapá**: 1: Serra do Navio, 0°59'N, 52°00'W, 1–14.v.1991, FIT (CHND), 5: 28.i-2.ii.1990, FIT (CHND); **Amazonas**: 2: INPA/Smithsonian Res., Manaus, 2°25'S, 59°50'W, ii.1994, Winkler method, leaf litter, terra firma forest, R. Didham (BMNH); 1: Reserva Ducke, 26km NE Manaus, ii.1995, FIT, M.G.V. (BMNH); **Mato Grosso:** 1: Cotriguaçu, Fazenda São Nicolau, Prainha, 9°51.6'S, 58°12.9'W, x.2009, FIT, F.Z. Vaz-de-Mello (CEMT); 2: Cotriguaçu, Fazenda São Nicolau, Matinha, 9°50.3'S, 58°15.05'W, x.2009, FIT, F.Z. Vaz-de-Mello (CEMT, FMNH), 1: x.2009, FIT, M.S. Gigliotti (MSCC), 1: xii.2009, FIT, F.Z. Vaz-de-Mello (AKTC), 2: xii.2010, FIT, F.Z. Vaz-de-Mello (FMNH); 2: Cotriguaçu, Fazenda São Nicolau, 9°49.0'S, 58°16.6'W, 15–18.xii.2010, FIT, F.Z. Vaz-de-Mello & A.F. Oliveira (MSCC, AKTC); 1: Diamantino, 20.2km S Posto do Gil on BR-364, 14°40'58"S, 56°17'57"W, 180m, 11.i.2001, FIT, F. Genier & F. Vaz-de-Mello (CMNC), 1: 14.i.2001, FIT, F. Genier & F. Vaz-de-Mello (CMNC); 1: Claudia, 11°24.5'S, 55°19.5'W, 17–27.x.2010, FIT, A.F. Oliveira (CEMT). **Pará:** 1:IPEAN, Utinga, Belém, 1°27'S, 48°26'W, x.1985, FIT (CHND), 1: xi.1984, FIT (MNHN); 1: Tucuruí, 3°45'S, 49°40'W, iv.1986, FIT (CHND), 2: v.1986, FIT (CHND, FMNH), 2: vi.1985, FIT (CHND, UFPR), 1: 10–29.vii.1985, FIT (CHND); 1: Carajás, Serra Norte, 6°04'S, 50°12'W, v.1985, FIT (CHND); 1: Ilha Arapiuns, 2°24'S, 54°57'W, 30–31.xii.2008, FIT (CEMT); 3: Melgaço, Rio Marinau, 1°51'S, 51°20'W, 31.x–13.xi.1993, FIT (CHND, MSCC, AKTC); 2: Marajo-Breves, 0°53'S, 50°32'W, 18.xi–5.xii.1987, FIT (CHND); **FRENCH GUIANA:** 4: Cayenne, 33.5km S and 8.4km NW of Hwy N2 on Hwy D5, 4°48'18"N, 52°28'41"W, 30m, 29.v–9.vi.1997, FIT, J. Ashe, R. Brooks (SEMC); 1: Cayenne, 20km SW, 4°48'18"N, 52°28'41"W, 30m, 29.v-9.vi.1997, FIT, J. Ashe & R. Brooks (CMNC); 1: Montagne des Chevaux, 4°43'N, 52°24'W, 18.i.2009, FIT, SEAG (MNHN). **GUYANA: Region 8**: 1: Iwokrama Forest, 26km SW Kurupukari, Iwokrama Mt., 4°20'2"N, 58°47'18"W, 300m, 23–25.v.2001, FIT, R. Brooks & Z. Falin (SEMC). **SURINAME: Brokopondo:** 1: Brownsberg Nature Preserve, Witi Creek Trail, 4°56'55"N, 55°10'53"W, 340m, 23–25.vi.1999, FIT, Z. Falin, A. Gangadin, H. Hiwat (SEMC), 2: 440m, 22–25.vi.1999, FIT, Z. Falin (SEMC), 1: 480m, 23.vi.1999, FIT, Z. Falin (CMNC); 1: Ston Eiland Eco Resort, nr. Brownsberg, 4°59.0'N, 55°8.0'W, 10–13.ii.2010, FIT, C. Gillet, P. Skelley, W. Warner (FSCA).

#### Other material.

**PERU: Madre de Dios:** 2: CICRA, Los Amigos Field Station, 12.5624°S, 70.0930°W, 288m, 2–11.i.2007, pitfall, terra firma forest, J. Jacobs, (CASC); 1: Manu National Park, Pantiacolla Lodge, Alto Madre de Dios River, 12°39.3'S, 71°13.9'W, 420m, 14–19.xi.2007, FIT, D. Brzoska (SEMC), 1: 400m, 23–26.x.2000, FIT, R. Brooks (SEMC); 1: Manu National Park, Pantiacolla Lodge, 5.5km NW El Mirador Trail, Alto Madre de Dios River, 12°39'10"S, 71°15'28"W, 500m, 23–26.x.2000, FIT, R. Brooks (SEMC). **COLOMBIA: Vaupés:** 4: Parque Nac. Mosiro-Itajura (Caparú), Centro Ambiental, 1°04'S, 69°31'W, 60m, 20–30.i.2003, FIT, D. Arias & M. Sharkey (IAVH).

#### Diagnostic description.

**Figure 51.**
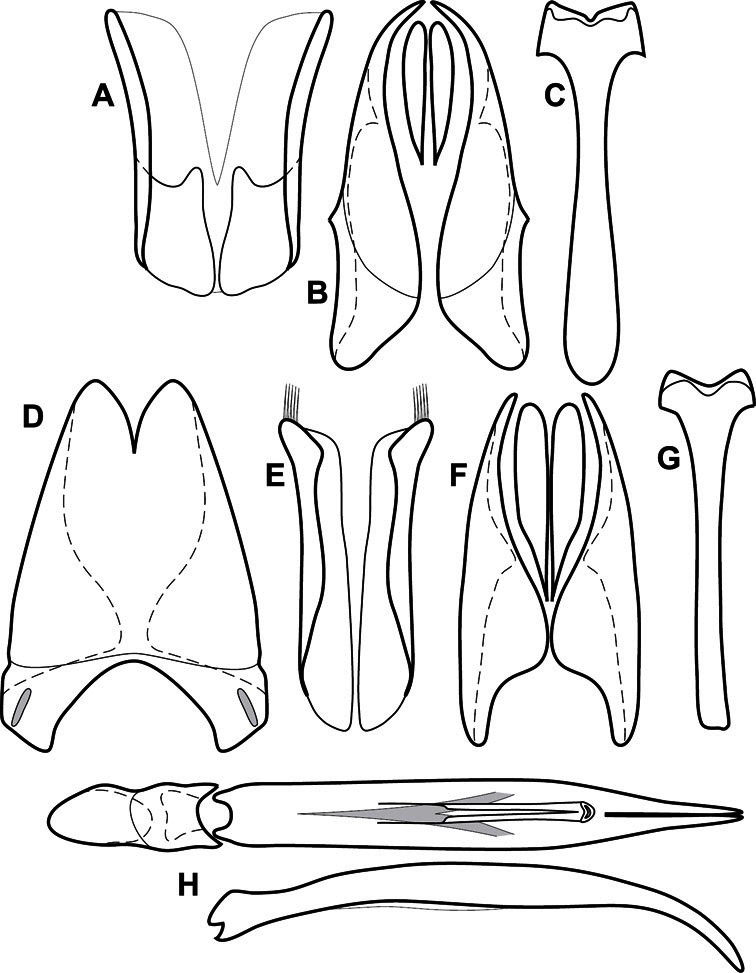
Male genitalia of *Operclipygus farctus* group. **A** S8 of *Operclipygus inflatus* **B** T9 & T10 of *Operclipygus inflatus* **C** S9 of *Operclipygus inflatus* **D** T8 of *Operclipygus punctifrons* **E** S8 of *Operclipygus punctifrons* **F** T9 & T10 of *Operclipygus punctifrons* **G** S9 of *Operclipygus punctifrons* **H** Aedeagus, dorsal and lateral views, of *Operclipygus punctifrons*.

This species is very similar to *Operclipygus farctissimus*, differing as follows: length: 2.00–2.40 mm, width: 1.75–2.03 mm; body rufopiceous, rounded and strongly convex; frons deeply impressed at middle, with central part of frontal stria strongly arcuate, detached from sides; antennal bossae strongly produced; epistoma convex; labrum narrow, short; pronotal disk with strong basal plicae in posterior third; anterior margin of pronotum angulately produced over head, anterior corners curved ventrad, forming vertical surface on either side of head; anterior pronotal gland openings close together behind eye; elytra with single complete epipleural stria, with epipleuron broad and smooth, outer subhumeral stria present in apical third, inner subhumeral stria absent, subhumeral interval strongly swollen, striae 1-3 complete, 4^th^ and 5^th^ striae represented only by short apical fragments, sutural stria present in apical two-thirds; prosternal keel with carinal striae meeting about one-fourth from presternal suture, keel coplanar with short prosternal lobe; mesoventral margin weakly projecting at middle, marginal stria interrupted; propygidium with rather small, round punctures separated by about their diameters at base, sparser toward apex; pygidium with fine, dense ground punctation only; marginal pygidial sulcus fine, obsolete in basal corners. Male genitalia generally similar to those of *Operclipygus farctus* (see Figs 48A–D, K), except T8(Fig. 51A) with deeper apical emargination; S8 with sides more strongly divergent, sclerotized inner edges of ventral halves approximate in basal fourth, with a small apical projection, then strongly divergent, broad and entirely membraneous in apical half; T9(Fig. 51B) with prominent basolateral angles; S9(Fig. 51C) slightly shorter, broader, with distinctive median and lateral longitudinal sclerotizations in apical half; tegmen identical to that of *Operclipygus farctus*.

**Remarks.** Within this group, this species is the most easily recognizeable due to its relatively extreme modifications of the head and prothorax(Fig. 50C), with the frons deeply impressed, the epistoma convex, the pronotal plicae well impressed, the anterior pronotal corners strongly deflexed, and the anterior margin of the pronotum angulate over the head. The preceding species, although very similar, has no appreciable pronotal projection, and has all these other features more modestly developed. While we limit the type series to localities in northeastern Brazil and nearby parts of the Guianas, the species is remarkably consistent in morphology, including that of male genitalia, throughout its broad range.

#### Etymology.

 The name of this species refers to the relatively strong convexity of the body as a whole, as well as that of the epistoma and the anterior half of the pronotum.

### Operclipygus distractus

(Schmidt, 1896) comb. n.

http://species-id.net/wiki/Operclipygus_distractus

Figs 50D–E Map 18

Phelister distractus Schmidt, 1896: 59; *Pseudister distractus*: Bickhardt 1917: 165.

#### Type locality.

BRAZIL: Rio de Janeiro:Petrópolis [22.5°S, 43.18°W].

#### Type material.

**Lectotype**, here designated: “*Phelister distractus* typ. Petrop.[olis]” / “Type” / “coll. J. Schmidt” / “*Phelister distractus* Schmidt, 1896 ex. Coll. Schmidt-Bickhardt” / “LECTOTYPE *Phelister distractus* Schmidt, 1896 M.S.Caterino & A.K.Tishechkin des. 2010” (ZMHB). This species was described from an unspecified number of specimens, and the lectotype designation fixes primary type status on the only known original specimen.

#### Other material.

**BRAZIL: Paraná:** 1: Guartelá, Campo São Paolo, 24°32'S, 50°17'W, 900m, xi.2007, pitfall, E. Grossi (CHND); 1: Piraquara, Mananciais da Serra, 25°29.77'S, 48°58.90'W, 1000m, 17–31.x.2007, FIT, P. Grossi & D. Parizotto (UFPR).

#### Diagnostic description.

Length: 2.00–2.18 mm, width: 1.53–1.68 mm; body rufopiceous, elytra and pygidia appearing faintly darker than rest of body, elongate, subdepressed; frons flat, sides of frontal stria weakly rounded inward, central part of frontal stria fine, evenly arcuate, narrowly detached from sides; supraorbital stria absent; labrum distinctly asymmetrical with median apical depression and blunt process left of center; left mandible untoothed, right mandible with small subacute basal tooth; pronotal disk weakly, indistinctly impressed in prescutellar region, with fine ground punctation and ~15 coarser punctures toward sides; marginal pronotal stria interrupted behind head, submarginal stria continuous from sides across front; median pronotal gland openings situated behind eye, anterolateral opening midway between that and pronotal corner; elytra with striae finely, not too deeply impressed, outer subhumeral stria present in apical half, inner subhumeral stria absent, striae 1-3 complete, 4^th^ stria present in apical half, 5^th^ stria present in apical third, sutural stria present in apical three-fourths; elytral disk with few apical marginal punctures; prosternal keel truncate at base, carinal striae complete, free basally, united in narrow anterior arch, middle part of keel distinctly microsculptured; prosternal lobe with marginal stria interrupted or absent at middle; mesoventrite broadly emarginate, marginal stria interrupted; mesometaventral stria narrowly arcuate, reaching middle of mesoventral disk, angulate at mesocoxa, lateral metaventral stria extended toward outer third of metacoxa; 1^st^ abdominal ventrite with two complete lateral striae, central part of disk with numerous small, shallow punctures in anterior half, between metacoxae; propygidium with moderately large, shallow, irregularly round punctures separated by slightly less than their diameters; pygidium with ground punctation fine and quite sparse, with coarser small punctures mainly in basal half; marginal pygidial sulcus complete, deeply crenulate, slightly removed from margin at middle, with fairly wide, flat marginal bead. Male not known.

#### Remarks.

*Operclipygus distractus* and *Operclipygus punctifrons* are unusual among members of the *Operclipygus farctus* group in a few characters. In particular they have the prosternal keel not at all emarginate at the base(Fig. 50E), and two complete lateral striae on the 1^st^ abdominal ventrite. However, due to the marginal position of their median pronotal gland openings we retain them here. *Operclipygus distractus* can be recognized by these characters in combination with its lack of dense frontal ground punctation.

### Operclipygus punctifrons sp. n.

urn:lsid:zoobank.org:act:5F5C582B-BB91-4F1E-91AE-0F21FDE84551

http://species-id.net/wiki/Operclipygus_punctifrons

Figs 51D–H 53A–B Map 18

#### Type locality.

BRASIL: Distrito Federal: Brasilia, IBGE Ecological Reserve [15°5.5'S, 47°53'W].

#### Type material.

**Holotype male**: “**BRASIL: Dist. Federal**, Brasilia, Res. Ecol. de IBGE, 15°5.5'S, 47°53'W. Lin. 1, Pto. 1. Armad. janela, area não queimada. 6.1.1998”/ “Caterino/Tishechkin Exosternini Voucher EXO-00550” (UFPR). **Paratypes** (2): 1: same data as type (CHND); 1: same locality, Malaise trap, 22.xii.1997 (UFPR)

#### Other material.

**ARGENTINA: Jujuy:** Parque Nac. Calilegua, Aguas Negras, 550m, 18–28.xii.1987, FIT, S. & J. Peck (CMNC).

#### Diagnostic description.

Length: 2.25–2.56 mm, width: 1.72–2.00 mm; body rufobrunneus, elongate, sides subparallel for most of length, subdepressed, pronotum and head with very conspicuous ground punctation; frons weakly depressed at middle, frontal stria divergent between eyes, more or less straight at middle; epistoma short; labrum about half as long as wide, shallowly emarginate; both mandibles with prominent subacute tooth at base; pronotal disk lacking prescutellar impression, ground punctation becoming more conspicuous to sides, with ~10 coarser punctures laterally; marginal stria interrupted behind head; submarginal stria continuous from sides across front, markedly crenulate; median pronotal gland opening behind eye, anterolateral opening midway between median and anterior pronotal corner; elytron with one complete epipleural stria, outer subhumeral stria nearly complete, barely abbreviated basally, inner subhumeral stria absent, striae 1-3 complete, 4^th^ stria nearly complete, abbreviated or interrupted near base, 5^th^ stria present in apical half, sutural stria present in apical two-thirds; elytral disk with distinct, irregular series of punctures along apical margin vaguely connecting apices of striae; prosternal keel very weakly produced at base, carinal striae connected at base and just short of presternal suture, enclosed area microsculptured, secondary striae faintly impressed behind prosternal gland openings; prosternal lobe lacking marginal stria, densely punctate at middle; mesoventrite weakly emarginate, marginal stria interrupted; mesometaventral stria weakly angulate at middle, reaching beyond middle of mesoventral disk, sinuate near mesocoxa, lateral metaventral striae extending to outer third of metacoxa; 1^st^ abdominal ventrite with two complete lateral striae, inner pair extended weakly mediad along basal margin, not meeting; propygidium with moderately large, shallow round punctures more or less uniformly separated by half their diameters; pygidium with ground punctation fine and sparse, with small round punctures quite uniformly separated by their diameters; marginal pygidial sulcus fine, complete. Male genitalia (Figs 51D–H): accessory sclerites present; T8 with sides evenly convergent from base to apex, without apicolateral desclerotized areas, basal emargination broadly arcuate, deep, nearly reaching basal membrane attachment line, apical emargination wide at apex, narrowing, ventrolateral apodemes most strongly developed near base, narrowing to near apex; S8 with sides subparallel, weakly divergent apically, apices of guides slightly produced on inner edges, 3 or 4 prominent setae apically, ventromedial edges approximate throughout length, possibly weakly fused; T9 with sides rather evenly, arcuately convergent to apex, apices narrow, subacute, not opposing; T10 large, completely divided; S9 with stem narrow, with strengthening ridge along midline, base truncate, apex narrowly inwardly arcuate, apical flange uninterrupted; tegmen long, narrow, subparallel in basal two-thirds narrowed to subacute apex, dorsal edge rather evenly curved from base to apex, membraneous beneath, appearing bulged, medioventral process long, acute at apex, very weakly sclerotized, contained within ventral membrane, not projecting beneath; basal piece about one-fourth tegmen length; median lobe narrow, over half tegmen length, with proximal apodemes weak, narrow, not obviously differentiated.

#### Remarks.

While apparently closely related to *Operclipygus distractus*, with a produced prosternal keel base, a few punctures along the apical elytral margin, and lacking a marginal stria on the prosternal lobe, this species is readily distinguished by its conspicuous pronotal and head ground punctation(Figs 53A–B), its complete frontal stria, and its nearly complete outer subhumeral stria. We exclude an Argentinian singleton from the type series due to slight aedeagal differences, though other characters indicate an unambiguously close relationship.

#### Etymology.

The name of this species refers to the conspicuous ground punctation of the frons, as well as on other parts of the body.

### Operclipygus latemarginatus

(Bickhardt, 1920) comb. n.

http://species-id.net/wiki/Operclipygus_latemarginatus

**Figure 52.**
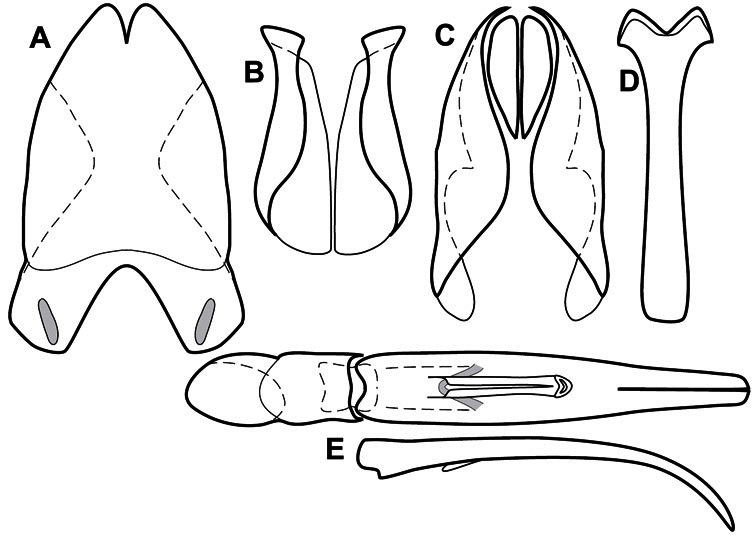
Male genitalia of *Operclipygus latemarginatus*. **A** T8 **B** S8 **C** T9 & T10 **D** S9 **E** Aedeagus, dorsal and lateral views.

Figs 52A–E 53C–D Map 18

Pseudister latemarginatus Bickhardt, 1920: 237.
Pseudister laterimarginatus : Mazur (1997, misspelling).

#### Type locality.

Not specified beyondBuenos Aires Province, Argentina.

#### Type material.

**Lectotype**, here designated (ZMHB): “Rep. Argentina. Prov. Buenos Aires, 17.X.1909, C. Bruch” / “LECTOTYPE *Pseudister latemarginatus* Bickhardt, 1920 M.S.Caterino & A.K.Tishechkin des. 2010”. There should also be a syntype in Argentina, which we were not able to borrow but should nonetheless henceforth be considered a paralectotype. This species was described from an unspecified number of specimens, and the lectotype designation fixes primary type status on one of the known syntypes.

#### Other material.

**BRAZIL: Minas Gerais**: 1: Ingaí, Univ. Fed. Lavras, Lavras, 21°14'S, 45°00'W, ix.2001, FIT, dry forest, F.Z. Vaz-de-Mello (CEMT); **Paraná:** 1: Tibagi, Parque Estad. Guartelá, 24.5663°S, 50.2570°W, 12–15.xii.2011, FIT, forest, M.S. Caterino & A.K. Tishechkin, DNA Extract MSC-2276 (SBMNH); 1: Campina Grande do Sul, Estrada de Mandacaia, 26.xii.2008, FIT, F.W.T. Leivas (UFPR); **Rio de Janeiro:** 1: Nova Friburgo, 22°16'S, 42°32'W, 26–31.x.2009, FIT (CHND).

#### Diagnostic description.

Length: 2.62–2.96 mm, width: 2.25–2.37 mm; body rufopiceous, subquadrate, depressed; frons prominent, transversely elevated between antennal bases but depressed at middle, frontal stria varied, broadly interrupted at middle in lectotype, fine fragments visible nearer the sides, complete, sinuate in all other specimens; labrum wide, one-third as long as wide, slightly emarginate anteriorly; left mandible with weak, blunt basal tooth, right mandible with small subacute tooth; antennal club with basal and middle annuli complete, transverse; pronotal disk with prescutellar impression narrow, distinct, as long or slightly longer than scutellum, with few larger punctures at sides; marginal pronotal stria fine, complete or interrupted behind head; submarginal stria continuous along lateral and anterior margins, may be sinuous at sides, with bead wider anteriorly (as in lectotype); anterior pronotal gland openings close together, somewhat posterolaterad eye; elytron with outer subhumeral stria briefly interrupted at middle but complete to apex, variably abbreviated at base, inner subhumeral stria absent, striae 1-3 complete, 4^th^ stria present in apical two-thirds or less, 5^th^ stria present in apical third or less, sutural stria present in apical two-thirds; prosternal keel weakly produced at base, carinal striae straight, convergent and connected anteriorly and posteriorly; prosternal lobe with complete marginal stria; anterior margin of mesoventrite broadly emarginate; marginal mesoventral stria very fine and barely interrupted at middle; displaced by anteriorly arcuate mesometaventral stria; lateral metaventral stria extending toward middle of metacoxa; 1^st^ abdominal ventrite with single lateral stria abbreviated apically, disk with few fine punctures along posterior margin; propygidium covered with small, shallow punctures separated by a little less than their diameters, slightly smaller and sparser posteriorly; pygidial punctures finer and sparser, with inconspicuous ground punctation; both propygidium and pygidium with transverse waves of microsculpture; marginal pygidial sulcus present around apical third of margin, fine but distinct, obsolete basally. Male genitalia (Fig. 52): accessory sclerites present; T8 with sides subparallel in basal two-thirds, faintly desclerotized at apicolateral angle, convergent to apex, with rather deep, narrow basal emargination, basal membrane attachment line distad basal emargination by about one-third its depth, apical emargination very narrow, ventrolateral apodemes symmetrical, widely separated basally; S8 rather short, sides subparallel, narrowed at middle, apices bluntly rounded, ventral halves approximate just at base, slightly diverging to apex, apical guides evenly developed from base to apex; T9 with sides subparallel in basal third, convergent to narrow, weakly opposing apices; T10 completely divided; S9 narrowest near apex, with sclerotized, elevated midline, weakly widened to subtruncate base, apex minutely emarginate, apical flanges separate, extending up onto lateral flanges; tegmen widest near base, narrowed fairly evenly to apex, apex evenly curved ventrad in apical half, medioventral process narrowly ‘U’-shaped, projecting beneath about one-fourth from base; basal piece about one-third tegmen length; median lobe about one-third tegmen length, with proximal apodemes thin, undifferntiated.

#### Remarks.

**Figure 53.**
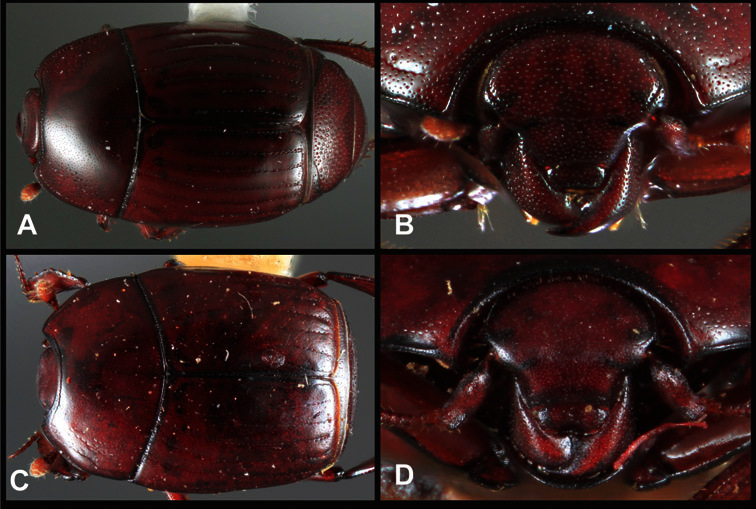
*Operclipygus farctus* group. **A** Dorsal habitus of *Operclipygus punctifrons* **B** Frons of *Operclipygus punctifrons* **C **Dorsal habitus of *Operclipygus latemarginatus* **D** Frons of *Operclipygus latemarginatus*.

This species is placed in the *Operclipygus farctus* group mainly on the basis of the position of the pronotal gland openings, close together along the anterior margin, and it is in some respects similar to the preceding two species, mostly in body shape, projecting prosternal keel, and pygidial punctation. However, it differs in a number of other characters, including frontal shape and striae(Fig. 53D), pronotal striae(Fig. 53C), presence of prescutellar impression, and most significantly, in having the annuli of the antennal club complete and straight/transverse. The apicolaterally desclerotized T8(Fig. 52A) on the other hand, is quite similar to that of several members of this group. Its position will require careful evaluation in future analyses. Specimens more or less corresponding to this species are known from a few localities in Minas Gerais, Paraná and Rio de Janeiro, Brazil. However, these lack the typically (or aberrantly?) sinuate submarginal pronotal stria. We consider them provisionally conspecific, but more material is needed. Bickhardt (1920) noted that the type was collected from the nest of *Ctenomys* Blainville (Rodentia: Ctenomyidae), or tuco-tuco, and Bruch (1937) provided a few additional details on the type collection.

### *Operclipygus hirsutipes* group

**Figure 54.**
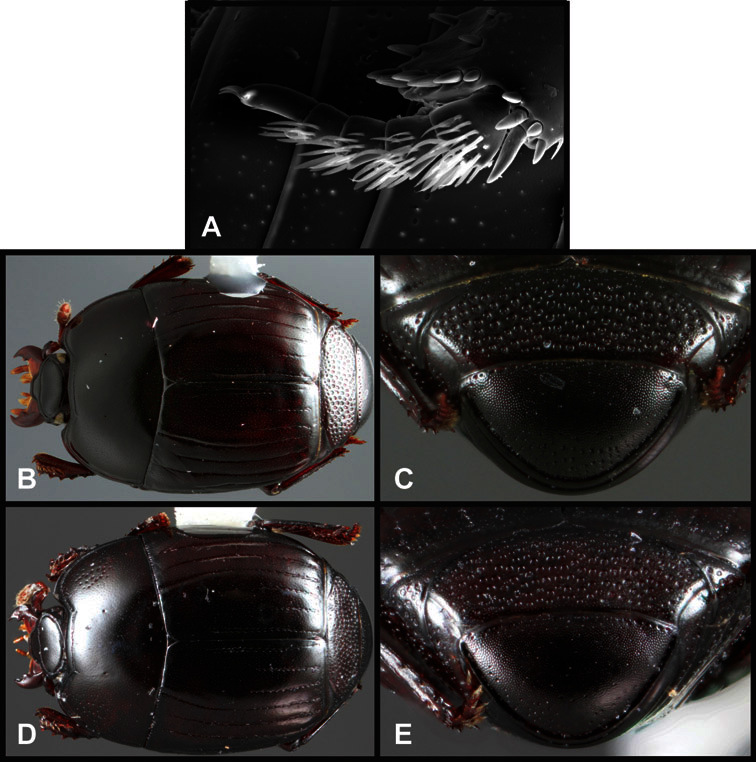
*Operclipygus hirsutipes* group. **A** Tarsus of *Operclipygus hirsutipes* **B** Dorsal habitus of *Operclipygus hirsutipes* **C **Pygidia of *Operclipygus hirsutipes* **D** Dorsal habitus of *Operclipygus guianensis* **E** Pygidia of *Operclipygus guianensis*.

**Figure 55.**
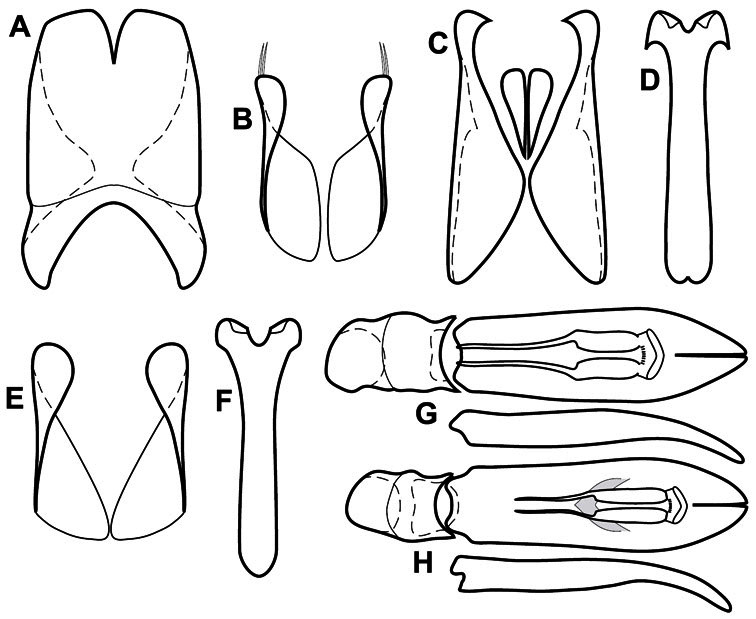
Male genitalia of *Operclipygus hirsutipes* group. **A** T8 of *Operclipygus hirsutipes* **B** S8 of *Operclipygus hirsutipes* **C** T9 & T10 of *Operclipygus hirsutipes* **D** S9 of *Operclipygus hirsutipes* **E** S8 of *Operclipygus guianensis* **F** S9 of *Operclipygus guianensis* **G** Aedeagus, dorsal and lateral views, of *Operclipygus hirsutipes* **H** Aedeagus, dorsal and lateral views, of *Operclipygus guianensis*.

The two species in this group are closely related and difficult to separate from each other. However, they are relatively easy to distinguish from other *Operclipygus* by the dense brush of setae on the tarsomeres(Fig. 54A). Most *Operclipygus* have only two short rows of setae lining the venter of each tarsomere, not a dense brush. Externally there is little else by which to recognize them. However, their genitalia are also highly distinctive, in particular the 9^th^ tergite(Fig. 55C), with its apices dorsolaterally flattened, and weakly divergent at the apex.

### Key to the species of the *Operclipygus hirsutipes* group

**Table:** 

1	Secondary punctures of pygidium finer, relatively inconspicuous(Fig. 54E); outer subhumeral stria usually complete; Guianas	*Operclipygus guianensis* sp. n.
–	Secondary punctures of pygidium coarser, conspicuous(Fig. 54C); outer subhumeral stria usually interrupted; western Amazonia	*Operclipygus hirsutipes* sp. n.

### Operclipygus hirsutipes sp. n.

urn:lsid:zoobank.org:act:4725B183-B10E-44C4-A3E9-C8651D576EE5

http://species-id.net/wiki/Operclipygus_hirsutipes

**Map 19.**
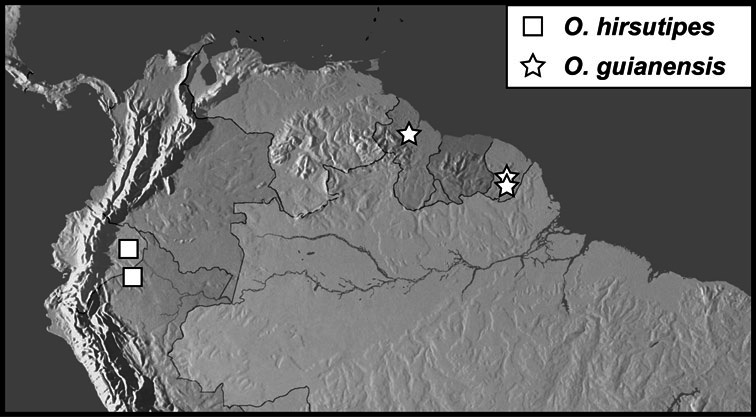
Records of the *Operclipygus hirsutipes* group.

Figs 54A–C 55A–D, G Map 19

#### Type locality.

ECUADOR: Orellana:Yasuní Research Station [0°40.5'S, 76°24'W].

#### Type material.

**Holotype male**: “**Ecuador:** Napo, mid.Rio Tiputini, Yasuní Res. Stn. 0°40.5'S, 76°24'W, FIT #M1, 5–11 Jul 1999. AKT#084, C.Carlton & A.Tishechkin”/ “LSAM0013250” (FMNH). **Paratypes** (5): 1: same data as type (LSAM); **ECUADOR: Orellana:** 1:Yasuní Res. Stn., mid.Rio Tiputini, 0°40.5'S, 76°24'W, 28.vi–5.vii.1999, FIT, C.E. Carlton & A.K. Tishechkin (LSAM), 1: 26.vii-4.viii.1999, FIT, A.K. Tishechkin (USFQ); 1: Tiputini Biodiversity Station, 0.6376°S, 76.1499°W, 4–9.vi.2011, FIT, M.S. Caterino & A.K. Tishechkin, DNA Extract MSC-2180 (SBMNH); 1: Parque Nac. Yasuní, Via Maxus at Puente Piraña, 0°39.5'S, 76°26'W, 14–20.vii.2008, FIT, A.K. Tishechkin (AKTC).

#### Other material.

**PERU: Loreto:** 1: 1.5km N Teniente Lopez, 2°35.66'S, 76°06.92'W, 210–240m, 20.vii.1993, FIT, R. Leschen (SEMC).

#### Diagnostic description.

Length: 2.25–2.53 mm, width: 2.03–2.18 mm; body rufopiceous, elongate, sides subparallel to weakly rounded, moderately strongly depressed; frons broadly depressed at middle, sides of frontal stria divergent, sinuate over antennal bases, complete across front; supraorbital stria complete, strongly arched at middle, connected to sides of frontal stria; labrum short, apically emarginate; left mandible with broad, subacute tooth, right mandible with smaller acute tooth; pronotal disk with vague, linear prescutellar depression, ground punctation fine, with few (~6) coarse punctures in a cluster near sides; anterior pronotal margin weakly, bluntly projecting over head; marginal stria interrupted at middle; lateral submarginal stria complete, curved inward at front nearly to anterior stria; anterior submarginal stria transverse across middle of front, just barely recurved posterad at ends; median pronotal gland openings about halfway back on disk on each side; elytron with two complete epipleural striae, with outer subhumeral stria interrupted at middle, inner subhumeral stria present at middle, apically and especially basally abbreviated, striae 1-3 complete to apex, but distinctly abbreviated from base, 4^th^ stria present in apical half, 5^th^ stria present in apical third, sutural stria present in apical two-thirds, widening and diverging from suture toward front; prosternal keel rounded, projecting at base, carinal striae complete, sinuate at middle, connected in narrow anterior arch; prosternal lobe with marginal stria barely abbreviated at sides; anterior mesoventral margin shallowly emarginate, with complete marginal stria; mesometaventral stria weakly and broadly arched forward to basal third of mesoventral disk, continued at sides by lateral metaventral stria extending toward middle of metacoxa; 1^st^ abdominal ventrite with complete, arcuate inner lateral stria and weak, abbreviated outer; meso- and metatarsomeres with dense brushes of ventral setae, not just rows along ventrolateral margins; propygidium densely and uniformly covered with ovoid, ocellate punctures, separated by less than one-third their diameters; most of pygidial disk with fine, dense ground punctation only, with single row of coarse punctures along basal margin, and with sparsely scattered, inconspicuous, coarser punctures toward apex; marginal pygidial sulcus complete, very deep, crenulate mainly on inner edge.Male genitalia (Figs 55A–D, G): accessory sclerites absent; T8 with sides narrowed near base, weakly rounded to near apex, apical emargination narrow, basal emargination broadly subangulate, nearly reaching basal membrane attachment line, ventrolateral apodemes most strongly developed near base, narrowed toward apex; S8 short, with sides parallel, apical guides gradually more strongly developed to rounded apices, ventrally halves approximate at base, divergent in apical half; apices of T9 not convergent to apex, apices dorsolaterally flattened, widened and rounded on distal margin, with acute teeth on inner edges; T10 much smaller than opening in T9, with halves separate; S9 with sides more or less parallel, barely narrowed near apex, with apical emargination small, rounded, apical flanges narrow, separate; tegmen short, broad, widest just beyond midpoint, lacking medioventral process; proximal apodemes of median lobe long, three-fourths tegmen length, abruptly narrowed one-third proximad from gonopore; basal piece about one-third tegmen length.

#### Remarks.

The two species in this group can be separated easily by geography. This species from western Amazonia may be recognized by the generally interrupted outer subhumeral stria, the depressed frons, more conspicuous coarse pygidial punctures(Fig. 54C), and the smaller number of lateral pronotal punctures(Fig. 54B), as well as by genitalic characters, in particular by the apparently absent medioventral process of the tegmen(Fig. 55G). One specimen from Peru differs in a few minor external characters, having complete outer subhumeral striae, and having the lateral and anterior submarginal pronotal striae joined in front, and we exclude it from the type series.

#### Etymology. 

This species’ name refers to its dense tarsal setae.

### Operclipygus guianensis sp. n.

urn:lsid:zoobank.org:act:91893068-07DF-4566-8E18-0BF0DB6DDB48

http://species-id.net/wiki/Operclipygus_guianensis

Figs 54D–E 55E–F, H Map 19

#### Type locality.

FRENCH GUIANA: Saül [3°1.37'N, 53°12.57'W].

#### Type material.

**Holotype male**: “**GUYANE FRANÇAISE**: Bélvédère de Saül, point de vue. 3°1'22"N, 53°12'34"W. Piège vitre, 20.xii.2010. SEAG leg.”/ “Caterino/Tishechkin Exosternini Voucher EXO-001766” (MNHN). **Paratypes** (10): **FRENCH GUIANA:** 1: Belvèdére de Saül, point de vue, 3°1'22"N, 53°12'34"W, 10.xii.2010, Window trap, SEAG (MNHN); 1: Régina, Réserve des Nouragues, 4°2.27'N, 52°40.35'W, 19.ii.2010, FIT, SEAG (CHND), 1: 3.xi.2009, FIT, SEAG (CHND); 2: Mont. Tabulaire Itoupé, 3°1.38'N, 53°5.73'W, 570m, 17.iii.2010, FIT, SEAG (FMNH, MSCC), 2: 24.iii.2010, FIT, SEAG (AKTC, MNHN), 2: 31.iii.2010, FIT, SEAG (CHND). **GUYANA: Mazaruni Potaro**: 1: Takutu Mountains, 6°15'N, 59°5'W, 11.xii.1983, Window trap, montane rainforest near logging area, P.J. Spangler, R.A. Faitoute & W.E. Steiner (USNM).

#### Diagnostic description.

In external characters, the differences between this species and the preceding are few and minor, as follows: length: 2.56–2.65 mm, width: 2.34–2.43 mm; frons not markedly depressed at middle; pronotal disk with larger number of coarser punctures near sides, ~10-15 in dense cluster just anterad middle; median pronotal gland openings near one-third from anterior margin of pronotum; elytra with outer subhumeral stria usually complete, striae 1-3 less distinctly abbrevated at base; 1^st^ abdominal ventrite with outer lateral stria curved behind metacoxa, ending in a distinctly enlarged gland opening. Male genitalia: T8 identical to that of *Operclipygus hirsutipes* (Fig. 55A); S8 with apices more broadly rounded(Fig. 55E), and inner edges more strongly divergent from near base; T9 identical to above; T10 small as above, but with halves fused for short distance at base; S9(Fig. 55F) narrower overall, with deeper, broader apical emargination; tegmen(Fig. 55H) similar in shape to above, with sides more rounded toward apex, and with medioventral process present, broadly ‘V’-shaped, weakly sclerotized but with acute apex, not projecting beneath tegmen; median lobe long, with wide gonopore, proximal apodemes about three-fourths tegmen length, abruptly narrowed midway to base of tegmen.

#### Remarks.

Within the group the Guianan species is best recognized by the more numerous and more strongly impressed lateral pronotal punctures(Fig. 54D), finer pygidial punctures(Fig. 54E), the generally complete outer subhumeral elytral stria, the flat frons, and by the distinctly enlarged gland opening on the 1^st^ abdominal ventrite in which the outer lateral stria of the disk terminates, as well as by male genitalic characters.

#### Etymology.

 This species is named for the region from which it is exclusively known.

### *Operclipygus hamistrius* group

**Figure 56:**
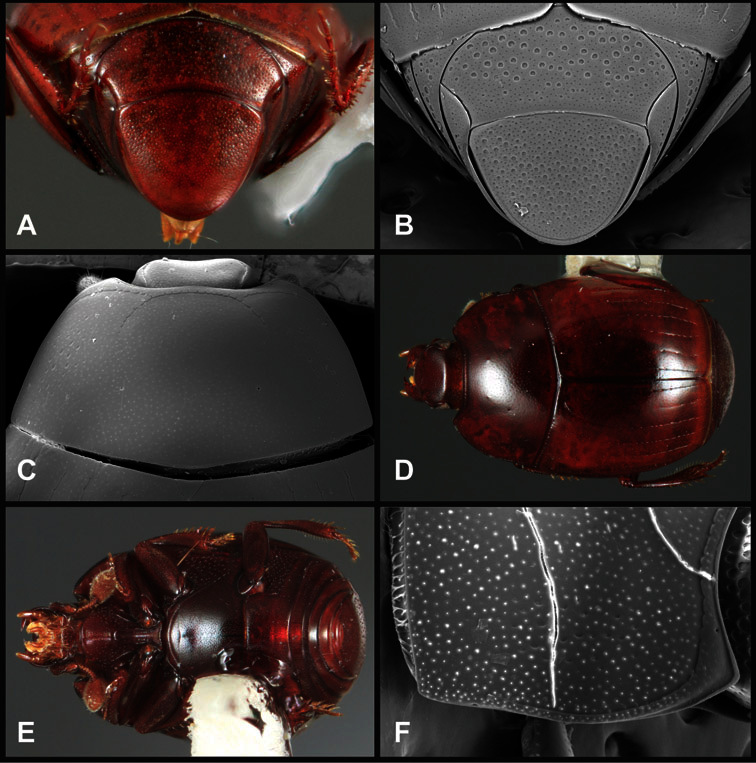
**.** *Operclipygus hamistrius* group. **A** Pygidial microsculpture of *Operclipygus arquus* **B** Pygidial microsculpture of *Operclipygus hamistrius* **C** Pronotum of *Operclipygus arquus* **D** Dorsal habitus of *Operclipygus hamistrius* **E** Ventral habitus of *Operclipygus hamistrius* **F** Lateral view of pronotum of *Operclipygus hamistrius*.

The *Operclipygus hamistrius* group is one of the most taxonomically challenging groups in the genus. Not only are the species very similar to each other, but they are also highly variable in many characters typically used for species diagnosis. Furthermore, the male genitalia show relatively little variation across most of the group, although a few species and subgroups of species can be recognized using genitalia. There is no single character by which to recognize members of the *Operclipygus hamistrius* group. Perhaps the most useful feature is the fact that nearly all species show conspicuous, generally transverse waves of microsculpture on some part(s) of the body, especially on the pygidium and propygidium (Figs 56A–B, 60B). The particular distribution of microsculpture can be useful in separating the species. Similar microsculpture can be found in a few other *Operclipygus*, but it is rare. The most typical form of the submarginal pronotal striae, with the anterior, central portion detached and recurved posterad for some distance onto the pronotal disk(e.g., Figs 56C, 58A), when observed in combination with a position of the median pronotal gland openings well behind the pronotal midline, is largely diagnostic, although a couple species we place in the *Operclipygus impunctipennis* group (which lack any microsculpture) show this as well. Similar pronotal striae can be found in assorted other *Operclipygus*, but essentially never in combination with such posteriorly displaced gland openings. However, there are some *hamistrius* group species that have the submarginal stria unbroken, and it may even be variable within a few of the species. Most of the species are relatively strongly depigmented, appearing rufescent, with only a few approaching piceous or even brunneus. The pattern of elytral striae, so variable among other species in the genus, is almost invariant in the *hamistrius* group, with an abbreviated outer subhumeral, absent inner subhumeral, complete 1^st^-4^th^ dorsals, and similarly shortened 5^th^ and sutural striae. The rare exceptions diagnose species in the group easily.

**Figure 57.**
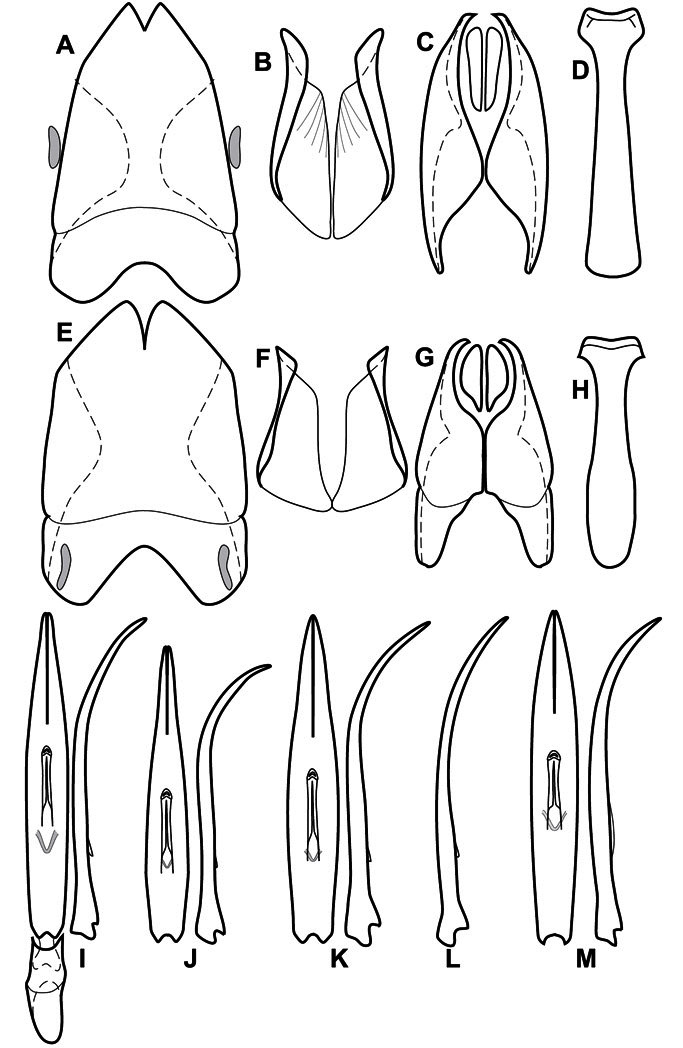
Male genitalia of *Operclipygus hamistrius* group. **A** T8 of *Operclipygus hamistrius* **B** S8 of *Operclipygus hamistrius* **C** T9 & T10 of *Operclipygus hamistrius* **D** S9 of *Operclipygus hamistrius* **E** T8 of *Operclipygus quinquestriatus* **F** S8 of *Operclipygus quinquestriatus* **G **T9 & T10 of *Operclipygus quinquestriatus* **H** S9 of *Operclipygus quinquestriatus* **I** Aedeagus, dorsal and lateral views, of *Operclipygus hamistrius* **J** Aedeagus, dorsal and lateral views, of *Operclipygus geometricus* **K** Aedeagus, dorsal and lateral views, of *Operclipygus arquus* **L **Aedeagus, lateral view, of *Operclipygus rufescens* **M** Aedeagus, dorsal and lateral views, of *Operclipygus chiapensis*.

The male genitalia in the group follow a very consistent pattern(Figs 57A–D, I–M), with accessory sclerites well developed; T8 with lateral margins angulate subapically, ventrolateral apodemes symmetrical along underside nearly meeting at midline; S8 variable in overall shape, but generally with narrow apical guides and distinct obliquely longitudinal wrinkles ventrally; T9 generally with apices convergent, finely subtruncate to acute; T10 with halves separate; apex of S9 shallowly arcuate but without fine median emargination, the apical flange entire; aedeagus generally narrow, elongate, apex subacute, with medioventral process thin but well sclerotized, narrowly ‘U’-shaped, weakly projecting beneath; median lobe narrow and short, no more than one-third tegmen length. Species level characters are found in variation in shape of T8, S8, S9, and the aedeagus.

The geographic distribution of the species is relatively restricted, and can assist in identification. They occur almost exclusively in Central America (including southern Mexico), and particularly in the highlands. A couple of species are found in extreme northwestern South America (Venezuela and Ecuador). But the *Operclipygus hamistrius* group is primarily a Central American radiation.

### Key to the species of the *Operclipygus hamistrius* group

**Table:** 

1	Outer subhumeral elytral stria more or less complete, may be interrupted or slightly fragmented toward base	2
–	Outer subhumeral stria shorter, absent from basal half	3
2	5^th^ dorsal elytral stria complete(Fig. 58D)	*Operclipygus quinquestriatus* sp. n.
–	5^th^ dorsal elytral stria present only in apical half (Fig. 58E)	*Operclipygus campbelli* sp. n.
3	4^th^ dorsal elytral stria arched mediad at base toward, sometimes reaching, elytral suture(Fig. 58A)	4
–	4^th^ dorsal stria not arched mediad at base, more or less parallel to 3^rd^ stria	5
4	Anterior and lateral submarginal pronotal striae continuous across front of pronotum(Fig. 59F); Central America	*Operclipygus propinquus* sp. n.
–	Anterior submarginal pronotal stria detached from lateral, briefly recurved posterad behind eyes; southeastern U.S.A., northern Mexico(Fig. 58A)	*Operclipygus geometricus* (Casey)
5	Lateral submarginal pronotal stria obsolete in basal half or more	6
–	Lateral submarginal pronotal stria reaching base, may be fine and close to margin, rarely interrupted at middle	9
6	Lateral submarginal pronotal stria meeting pronotal margin near midpoint(Fig. 59G), diverging strongly toward front, nearly meeting anterior submarginal stria, which is strongly arcuate, ends oblique, not recurved directly posterad; propygidium and pygidium lacking microsculpture	*Operclipygus intersectus* sp. n.
–	Lateral submarginal pronotal stria not meeting pronotal margin at midpoint, nor diverging so strongly from margin toward front, may be continuous with anterior submarginal; at least propygidium with conspicuous microsculpture	7
7	Body distinctly bicolored(Fig. 58F)	*Operclipygus dybasi* sp. n.
–	Body unicolored	8
8	Anterior and lateral submarginal pronotal striae continuous across front; males with narrow depression along anterior half of the lateral submarginal stria, this depression delimited posteriorly by a fine, parallel secondary stria which abruptly bends to margin at middle(Fig. 59B); Central America	*Operclipygus impressicollis* sp. n.
–	Anterior submarginal stria detached from lateral, ends recurved posterad(Fig. 59H); males and females both with weak, indistinct depression behind submarginal stria, not delimited by secondary stria; Venezuela	*Operclipygus troglodytes* sp. n.
9	Pronotum lacking coarse lateral punctures(Fig. 60A); marginal pygidial sulcus weak, fragmented apically, may be absent(Fig. 60B)	*Operclipygus montanus* sp. n.
–	Pronotum with conspicuous lateral punctures; pygidial stria varied	10
10	Anterior submarginal pronotal stria detached from lateral, forming an even arch across anterior one-third of pronotum(Fig. 56C)	*Operclipygus arquus* sp. n.
–	Anterior submarginal pronotal stria either continuous with lateral, or if detached, recurved perpendicularly posterad, not forming a smoothly curved arch	11
11	Marginal pronotal stria descending to hypomeron about one-fourth behind anterior pronotal corner, conspicuously disrupting margin(Fig. 56F); propygidium and pygidium with conspicuous microsculpture(Fig. 56B); frontal stria usually complete; northern Central America, southern Mexico	*Operclipygus hamistrius* (Schmidt)
–	Marginal pronotal stria not disrupting margin, rarely descending to hypomeron closer to anterior corner; other characters varied	12
12	Outer subhumeral stria absent; marginal mesoventral stria(Fig. 58C) complete along anterior margin; not displaced by mesometaventral stria; pygidium lacking microsculpture	*Operclipygus rubidus* (Hinton)
–	Outer subhumeral stria present in apical half of elytron; marginal mesoventral stria interrupted at middle; pygidium with microsculpture or not	13
13	Propygidium and entire pygidium with conspicuous microsculpture; anterior submarginal pronotal stria detached from lateral stria, ends recurved posterad (Fig. 59C); pronotum with conspicuous ground punctation, especially toward sides; head broad; labrum wide, emarginate(Fig. 4D)	*Operclipygus nubosus* sp. n.
–	Pygidium with microsculpture at most along basal margin, frequently entirely lacking; anterior and lateral submarginal pronotal striae continuous along anterior margin	14
14	Marginal pygidial sulcus well-impressed around apical half; lateral submarginal pronotal stria very close to margin, complete(Fig. 49A)	*Operclipygus rufescens* sp. n.
–	Marginal pygidial sulcus weak, fragmented near apex; lateral submarginal pronotal stria more distant from margin, may be interrupted at middle	15
15	Ground punctation of pronotum conspicuous(Fig. 59D); frontal stria complete; northern Central America	*Operclipygus chiapensis* sp. n.
–	Ground punctation of pronotum fine, sparse(Fig. 58G); frontal stria interrupted at sides; Ecuador	*Operclipygus pichinchensis* sp. n.

### Operclipygus hamistrius

(Schmidt, 1893) comb. n.

http://species-id.net/wiki/Operclipygus_hamistrius

**Map 20.**
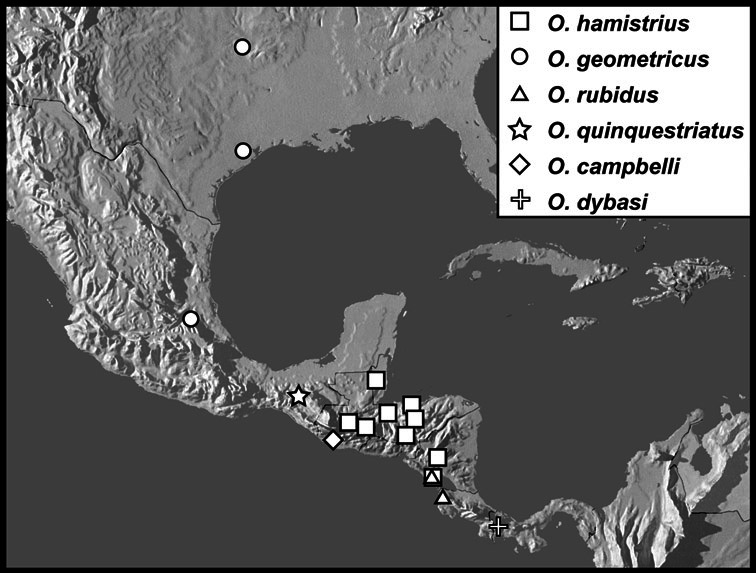
Records of the *Operclipygus hamistrius* group.

Figs 4B , 6F 56B, D–F 57A–D, I Map 20

Phelister hamistrius Schmidt, 1893: 13.

#### Type locality.

Not specified beyond Mexico.

#### Type material.

**Lectotype**, here designated (ZMHB): “Mexico.”/”*hamistrius*” / “LECTOTYPE *Phelister hamistrius* Schmidt M.S.Caterino & A.K.Tishechkin des. 2010”. This species was described from an unspecified number of specimens, and the lectotype designation fixes primary type status on the only known original specimen.

**Other material. BELIZE: Cayo:** 1: Las Cuevas Research Station, 16°44'N, 88°59'W, vi.2006, J. Kitson (BMNH), 1: 550m, v.1997, D. Inward (BMNH); 1: Chiquibul Forest Reserve, San Pastor, 13.xi.1994, FIT (BMNH); 1: Gran de Oro, 6.i.1995, FIT (BMNH). **GUATEMALA**: **Baja Verapaz**: 1: 7km E Purulha, 1600m, 23.v.1991, cloud forest litter, R. Anderson (SEMC); 1: 17km N Salama Hy 5, 1700m, 29.vi–3.vii.1993, FIT, J. Ashe & R. Brooks (SEMC); **Zacapa**: 2: 3.5km SE La Union, 1500m, 25–27.vi.1993, FIT, J. Ashe & R. Brooks (SEMC, FMNH); 3: 23–25.vi.1993 (SEMC, MSCC, AKTC). **HONDURAS: Atlantida**: 2: 15km W La Ceiba, 15–19.vi.1996, FIT, tropical rainforest, R. Lehman (TAMU), 1: 9–30.vii.1996, Malaise trap, tropical rainforest, R. Lehman (TAMU); **Cortés**: 2: Parque Nac. Cusuco, El Cortecito, 361198, 1717050, 1250m, 4–7.vi.2006, FIT, mature broadleaf forest, J. Nunez-Mino (OUMNH); 1: Parque Nac. Cusuco, 15°29'N, 88°13'W, 30.viii.1995, Malaise trap, oak/pine cloud forest, R. Cave, (AKTC); 1: 15.vii.1995, FIT, R. Cordero (AKTC); 1: Parque Nac. Cerro Azul-Meambar, Los Pinos, 14°52.4'N, 87°54.7'W, 800m, 10–16.v.2002, FIT, secondary forest, S. Peck (CHND); **Francisco Morazán**: 1: Yuscaran Cerro Montserrat, 28.i.1994, W. Morjan (AKTC); **Olancho:** 1: Parque Nac. La Muralla, 15km N La Union, 15°07'N, 86°45'W, ii.1995, Malaise trap, high elevation rainforest, R. Cave (AKTC); 1: La Union, Parque Nac. La Muralla, x.1994, R. Cordero (AKTC), 2: xi.1994, I. Maradiaga (AKTC), 2: 20–31.viii.1994, R. Cordero (AKTC). **NICARAGUA, Granada**: 1: Volcan Mombacho, San Joaquin #3, 21.vi.1998, malaise trap, J.M. Maes (MEL); 1: Volcan Mombacho, El Progreso #3, 15.iv.1998, malaise trap, J.M. Maes (MEL); **Matagalpa**: 1: 6 km N Matagalpa, Selva Negra Hotel, 12°59.99'N, 85°54.53'W, 1530m, 20–22.v.2002, FIT, R. Brooks, Z. Falin, S. Chatzimanolis (SEMC); 6: Selva Negra Reserve, 13°0.3'N, 85°54.2'W, 1395m, FIT, 19–27.v.2012, A. Derunkov, DNA Extracts MSC-2334, MSC-2349, MSC-2350, MSC-2351 (SBMNH, MSCC, AKTC).

#### Diagnostic description.

Length: 1.78–2.25 mm, width: 1.44–1.84 mm; body rufescent, elongate oval, outline slightly interrupted at humeri, pronotum more nearly parallel-sided, moderately convex; frons shallowly depressed at middle, with conspicuous, fine ground punctation; frontal stria weakly divergent between eyes, anterior portion varied from complete to absent between antennal bases, occasionally represented only by isolated fragments; apical margin of labrum distinctly emarginate; left mandible untoothed, right mandible with small, acute basal tooth; pronotum lacking prescutellar impression, disk with ~15 small coarse punctures at sides; marginal stria of pronotum descending from dorsal margin anteriorly to hypomeron about one-fourth behind anterior corners, markedly disrupting margin; lateral submarginal stria very close to sides, frequently weak, fragmented toward base, either continuous across front or interrupted, the central portion barely detached with its ends faintly recurved posterad; median pronotal gland openings about two-thirds pronotal length from anterior margin; elytra with two complete epipleural striae, outer subhumeral stria present in apical half, inner subhumeral stria absent, striae 1-4 complete, 5^th^ stria present in apical third, sutural stria present in apical half; nearly entire venter bearing waves of microsculpture; prosternal keel weakly emarginate at base, carinal striae complete, narrowed between coxae, with faint microsculpture between, meeting in bulbous anterior arch, weak secondary striae present behind prosternal gland openings; prosternal lobe with marginal stria abbreviated at sides; anterior margin of mesoventrite weakly projecting, marginal stria complete or interrupted; mesometaventral stria strongly arched forward very close to anterior mesoventral margin, continued by lateral metaventral stria toward metacoxa, frequently interrupted in posterior half of metaventral disk; central portion of metaventral disk with conspicuous ground punctation, particularly toward posterior margin; 1^st^ abdominal ventrite with single, incomplete lateral stria, punctures of sides of disk simple, separate, not coalescing or forming strigosity; propygidium and pygidium with very conspicuous transversely reticulate microscultpure; propygidium with sparse small, shallow punctures, separated 2–3× their diameters; pygidium with smaller punctures separated by only about 1.5× their diameters; marginal pygidial stria fine, present only around apical half of margin, occasionally interrupted near apex. Male genitalia (Figs 57A–D, I): accessory sclerites well developed; T8 with sides straight, weakly convergent to near apex, with faint desclerotization and abrupt step to apex (especially evident in lateral view), basal emargination narrow, rather shallow, reaching only halfway to basal membrane attachment line, ventrolateral apodemes symmetrical along underside nearly meeting at midline; S8 rather short, with sides weakly convergent toward apex, apical guides gradually more strongly developed toward apex, apices obliquely subacute, ventral surface distinctly wrinkled, halves approximate near base, weakly diverging toward apex; T9 with sides moderately rounded, apices convergent, subacute; T10 with halves separate; S9 with broad, weakly arcuate base, narrowed apically, apex shallowly arcuate but without fine median emargination, apical flange entire; tegmen narrow, elongate, widest just beyond midpoint, apex subacute, with medioventral process thin but well sclerotized, narrowly ‘U’-shaped, projecting beneath, about one-fourth from tegmen base; basal piece about one-third tegmen length; median lobe thin, no more than one-third tegmen length.

#### Remarks.

The nominate species in this group can be recognized by the combination of: simple punctures on sides of the 1^st^ abdominal ventrite (Fig. 56E), rather than elongate gouges; the descent of the marginal pronotal stria to the hypomeron about one-fourth behind the anterior corner, usually conspicuously disrupting the margin (Fig. 56F); the frequently complete frontal stria; and the usually continuous lateral and anterior submarginal striae (Fig. 56D). The unique type specimen, from an unspecified locality in Mexico, does not correspond extremely well with specimens from parts of northern Central America, having the pronotal striae broken, in particular, but they are certainly close. More material from Mexico would help substantiate the differences, and perhaps justify the separation of populations from most of these other areas as a distinct species.

### Operclipygus geometricus

(Casey, 1893) comb. n.

http://species-id.net/wiki/Operclipygus_geometricus

Figs 57J 58A Map 20

Phelister geometricus Casey, 1893: 550.

#### Type locality.

Not specified beyond Texas State, USA.

#### Type material.

**Holotype**: “Tex” / “*geometricus* Csy” / “CASEY bequest 1925” / “TYPE USNM 38456” (USNM), examined 2011.

#### Other material.

**MEXICO: Hidalgo**: 1: 5.v.1939, HSB (TAMU); **USA: Oklahoma:** 1: Latimer Co., iv.1994, K. Stephan (TAMU), 1: ix.1990, K. Stephan (TAMU), 1: v.1988, K. Stephan (TAMU), 1: vi.1995, K. Stephan (TAMU), 1: vii.1995, K. Stephan (TAMU); **USA**: **Texas**: 17: Fort Bend Co., Brazos Bend St. Pk., 15.vi–23.ix.2000, FIT, buckeye-sycamore forest, B. Raber & E. Riley (TAMU, FMNH, MSCC, AKTC), 24: 4.vi–25.ix.2000 (TAMU).

#### Diagnostic description.

Length: 1.90–2.15 mm, width: 1.56–1.84 mm; body rufobrunneus, slightly elongate oval, widest behind humeri, moderately convex; frons depressed at middle, sides of frontal stria weakly divergent, frequently interrupted over antennal bases, central part sinuate, weakly crenulate; supraorbital stria weak, fragmented to complete; labrum slightly asymmetrically emarginate apically; left mandible without tooth, right mandible with small acute basal tooth; prescutellar impression absent; pronotal disk wth fine sparse ground punctation and numerous (~20) coarse elongate punctures laterally; marginal pronotal stria fine, complete around sides and front; lateral submarginal stria close to margin at sides, frequently fragmented toward base, curving inward at front nearly to anterior submarginal stria, which is barely recurved posterad at sides; median pronotal gland openings far behind ends of anterior stria, three-fourths of the distance from the anterior to the posterior pronotal margin; elytra with two complete epipleural striae, the uppermost broadly sulciform, outer subhumeral stria represented by only short apical fragment, rarely with short basal fragment also, inner subhumeral stria absent, striae 1-4 complete, the 4^th^ stria arched inward basally, ending freely, 5^th^ stria present in apical half to two-thirds, sutural stria slightly shorter; elytral disk with few small punctures subserially arranged along apical margin; prosternal keel weakly emarginate at base, carinal striae complete, rather widely separated to front, united in broad anterior arch; prosternal lobe with weak, frequently fragmented marginal stria; anterior mesoventral margin weakly projecting, marginal stria weak, generally interrupted at middle; mesometaventral stria arched strongly forward to near anterior mesoventral margin, forming a continuous arc with lateral metaventral stria which extends posterad to inner third of metacoxa; 1^st^ abdominal ventrite with single, incomplete lateral stria, punctures of lateral part of disk coalescing into elongate strigosity posterolaterad coxa; propygidium uniformly covered with shallow, round punctures separated by about one-half their diameters, lacking microsculpture; pygidium with ground punctation fine, sparse, with small punctures throughout, separated by about twice their diameters; marginal pygidial stria finely impressed along apical half of margin, weak and rarely fragmented. Male genitalia with segments 8–10 identical to those of *Operclipygus hamistrius* (see Figs 57A–D); tegmen (Fig. 57J) shorter, narrower, especially toward apex, widest in basal half, strongly bent ventrally in apical third, medioventral process small, projecting beneath about, one-fourth from base.

**Figure 58.**
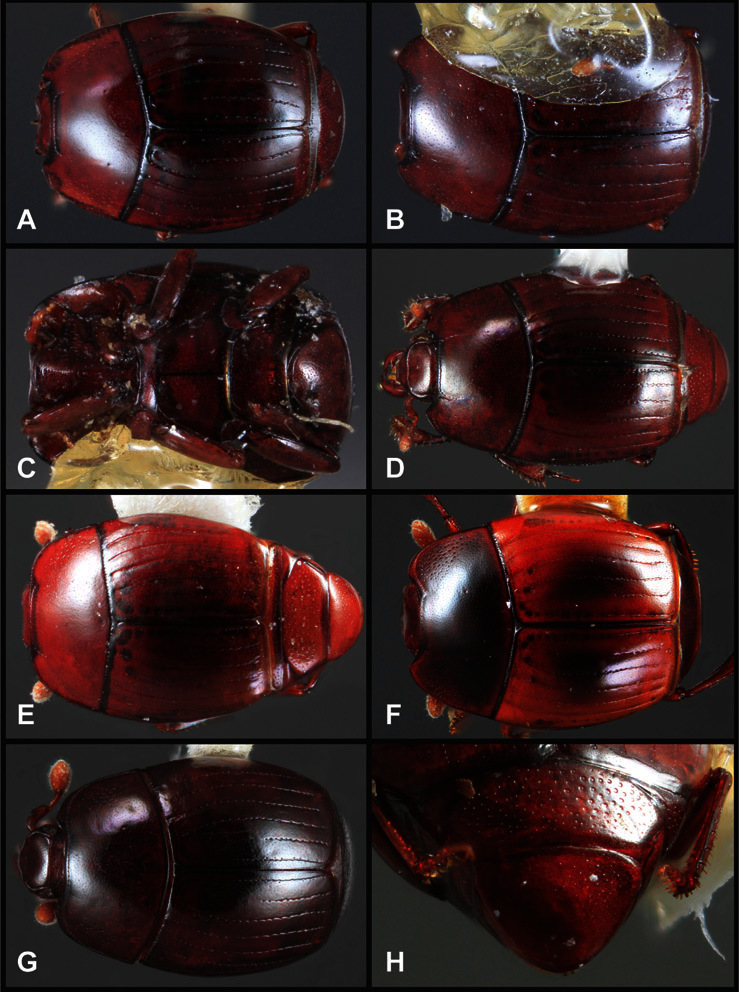
*Operclipygus hamistrius* group. **A** Dorsal habitus of *Operclipygus geometricus* **B** Dorsal habitus of *Operclipygus rubidus* **C** Ventral habitus of *Operclipygus rubidus* **D** Dorsal habitus of *Operclipygus quinquestriatus* **E** Dorsal habitus of *Operclipygus campbelli* **F** Dorsal habitus of *Operclipygus dybasi* **G** Dorsal habitus of *Operclipygus pichinchensis* **H** Pygidia of *Operclipygus pichinchensis*.

**Remarks.** While the distribution of this species is practically diagnostic, it differs substantially from other members of the group in several characters. The frontal stria is frequently complete, or narrowly interrupted over the antennae; the elytral striae are strongly and crenulately impressed (Fig. 58A), with the outer subhumeral stria very short and apical; it is essentially devoid of microsculpture, except rarely on the sides of the sterna; and the propygidial punctures are unusually dense and numerous. With its broken submarginal pronotal striae and displaced median pronotal gland openings it cannot be confused with any other Exosternini in or near the Nearctic.

### Operclipygus rubidus

(Hinton, 1935) comb. n.

http://species-id.net/wiki/Operclipygus_rubidus

Figs 58B–C Map 20

Phelister rubidus Hinton, 1935c: 59.

#### Type locality.

Not specified beyond Mexico.

#### Type material.

**Holotype**: “Mexico” / “Type” / “G.Lewis Coll. B.M.926-369” / “ *Phelister rubidus* Hntn Type” (BMNH), examined 2010.

#### Other material.

**COSTA RICA: Guanacaste:** 1: Parque Nac. Sta. Rosa, Est. Sta. Rosa, 3–12.vi.1992, III curso Parataxon. (INBIO). **NICARAGUA: Granada**: 1: Reserva Domitila, 11°42.50'N, 85°57.20'W, 100m, 6–9.vi.2002, FIT, R. Brooks, Z. Falin, S. Chatzimanolis (SEMC); 1: Volcan Mombacho, Santa Ana #3, 2.vi.1998, malaise trap, J.M. Maes (MEL), 2: 30.vi.1998, malaise trap, J.M. Maes (FMNH), 2: 15.vii.1998, malaise trap (MEL), J.M. Maes; 2: Volcan Mombacho, San Joaquin #3, 30.vi.1998, malaise trap, J.M. Maes (MSCC, AKTC); **Masaya**: 1: Las Flores, vi.1994, malaise trap, J.M. Maes (FMNH).

#### Diagnostic description.

Length: 1.81–1.97 mm, width: 1.47–1.62 mm; body oval, subquadrate, rufescent; frons flat, frontal stria varied across frons, from nearly complete to fragmented to broadly interrupted; epistoma and labrum narrowed, labrum 1.5× as wide as long, truncate; pronotal disk finely punctate, with ~20 coarser lateral punctures; marginal pronotal stria not descending onto hypomeron; lateral submarginal pronotal stria complete, very close to marginal at sides, not meeting slightly recurved anterior marginal in front; elytra with subhumeral striae absent, striae 1-4 complete, 5^th^ and sutural striae present in apical two-thirds; venter almost entirely lacking microsculpture, with only vestigial fragments at sides of sterna; prosternal keel truncate at base, with carinal striae strongly converging, parallel in apical third, joined by narrow anterior arch; mesoventrite not projecting; propygidium broad with scattered small punctures, with vestigial microsculpture; pygidium lacking microsculpture, also with sparse small punctures, fine marginal striae nearly complete, usually obsolete only in basal fourth. Male: segments 8–10 indistinguishable from those of *Operclipygus hamistrius* (see Figs 57A–D), and tegmen indistinguishable from that of *Operclipygus geometricus* (see Fig. 57J).

#### Remarks.

This distinctive species may be recognized by the lack of an outer subhumeral stria, the relatively flat frons, the numerous lateral pronotal punctures, and the complete marginal mesoventral stria (Fig. 58C), which is not reached by the subangulate mesometaventral stria.

### Operclipygus quinquestriatus sp. n.

urn:lsid:zoobank.org:act:1B70F1F0-4819-4B60-A814-C607FD472C92

http://species-id.net/wiki/Operclipygus_quinquestriatus

Figs 57E–H 58D Map 20

#### Type locality.

MEXICO: Chiapas: Sumidero National Park [16°50'N, 93°5.5'W].

#### Type material.

**Holotype male**: “**MEXICO:** Chiapas, Pq. Nac. Sumidero, 1000m. F.I.T. 25 May 1990. H.&A.Howden”/ “Caterino/Tishechkin Exosternini Voucher EXO-00333” (CNCI). **Paratype**: 1: same data as type (BDGC).

#### Diagnostic description.

Length: 1.75–2.06 mm, width: 1.50–1.65 mm; body rufescent, elongate ovoid, widest well behind humeri, moderately, evenly convex; microsculpture almost completely absent, vague rudiments present at sides of abdominal sterna; frons flat, not markedly depressed at middle, frontal stria complete; supraorbital stria absent; labrum about twice as wide as long, weakly emarginate apically, with weak apical process below margin; left mandible untoothed, right with small, subacute basal tooth; antennal club only slightly elongate; pronotal disk lacking prescutellar impression, ground punctation sparse, but conspicuous, with few coarser punctures sparsely scattered along sides; marginal stria barely descending to hypomeron at sides, only narrowly interrupted behind head; lateral submarginal pronotal stria complete, close to side, curving inward toward front, nearly meeting anterior submarginal stria, which is recurved posterad about one-third the length of the pronotal disk; median pronotal gland openings about two-thirds pronotal length from anterior margin; elytra with two complete epipleural striae, outer subhumeral stria nearly complete, but briefly interrupted at middle, inner subhumeral stria absent, striae 1-5 complete, sutural stria barely abbreviated basally; prosternal keel weakly emarginate at base, with complete carinal striae barely converging anteriorly, united in anterior arch; prosternal lobe with nearly complete marginal stria very close to margin; mesoventral margin with narrow, but distinct median projection, marginal stria complete; mesometaventral stria narrowly angulate anteriorly, reaching just forward of mesoventral midpoint; lateral metaventral stria extending to middle of metacoxa; 1^st^ abdominal ventrite with complete inner lateral stria, outer slightly abbreviated, sides of disk with punctures coalesced into elongate strigosity; propygidium with ground punctation fine, sparse, with small round punctures evenly separated by about their diameters; pygidium with fine, sparse ground punctation and very few coarser punctures interspersed, mainly in basal corners; marginal pygidial stria fine, nearly complete, though obsolete at base and narrowly interrupted at apex. Male genitalia (Figs 57E–H) extremely similar to those of *Operclipygus geometricus*, differing by T8 with sides slightly narrowing; S8 shorter, sides more strongly convergent toward apex, apical guides narrower; T9 halves approximate for about middle third of their length, apices narrow, weakly convergent; S9 very narrow for most of apical half, abruptly expanded to basal half, basal margins subparallel, rounded apically; tegmen indistinguishable from that of *Operclipygus geometricus* (see Fig. 57J), widest in basal half, narrow in most of apical half, with very narrow ‘U’-shaped medioventral process barely projecting beneath.

#### Remarks.

While distinctly a member of the *Operclipygus hamistrius* group, this species is unusual in having a complete 5^th^ dorsal elytral stria (Fig. 58D). This alone will distinguish it from other members of the group.

#### Etymology.

This species’ name refers to its five complete dorsal elytral striae.

### Operclipygus campbelli sp. n.

urn:lsid:zoobank.org:act:A1857408-5AA8-493E-8A81-9361E8105E4E

http://species-id.net/wiki/Operclipygus_campbelli

Fig. 58E Map 20

#### Type locality.

GUATEMALA: Suchitepéquez: Finca San Rafael Olimpo [14°32'N, 91°34'W].

#### Type material.

**Holotype male**: “GUAT. Finca San Rafael Olimpo, Cuyotenango Such. I.21.1966, J.M.Campbell, 1700’“ / “collected from large detritus cavity of *Atta sp*., 6-8’ deep”/ “FMNH-INS 0000069340” (FMNH). **Paratypes** (186): **GUATEMALA**: **Suchitepéquez**: 116: Cuyotenango, Finca San Rafael Olimpo, 1700ft, 21.i.1966, large detritus cavity of *Atta* sp., 6-8’ deep, J.M. Campbell (FMNH, MSCC, AKTC, CHND), 70: 26.ii.1966 (FMNH).

#### Diagnostic description.

This species is extremely similar to the preceding, differing in only a few characters as follows: length: 1.65–1.93 mm, width: 1.34–1.59 mm; body completely lacking microsculpture; marginal pronotal stria not descending to hypomeron, generally complete along upper edge of lateral pronotal margin as well as behind head; anterior submarginal pronotal stria strongly recurved posterad on about anterior one-third of pronotal disk; elytra with outer subhumeral stria complete, interrupted at middle, or with apical half obsolete, 5^th^ stria present in apical half, sutural stria present in apical two-thirds; prosternal keel truncate at base, not emarginate; mesoventral margin weakly projecting at middle, mesoventral marginal stria complete; mesometaventral stria bluntly angulate forward, only reaching middle of mestosternal disk; propygidium with coarser punctures slightly larger and more closely set, separated by slightly less than their diameters; marginal pygidial stria obsolete in basal half or more. Male genitalia indistinguishable from those of *Operclipygus geometricus* (see Figs 57A–D, J).

#### Remarks.

This species is very similar to *Operclipygus quinquestriatus*. Both have a nearly complete, but interrupted outer subhumeral elytral stria, as well as a complete frontal stria, strongly recurved anterior submarginal pronotal stria, and a lateral submarginal pronotal stria that is complete and close to the margin. *Operclipygus campbelli* has the 5^th^ dorsal elytral stria short (Fig. 58E), nowhere near complete as in *Operclipygus quinquestriatus*.

#### Etymology.

We name this species to honor J. Milton Campbell, recognizing his efforts in collecting an exceptional series of this otherwise uncollected species.

### Operclipygus dybasi sp. n.

urn:lsid:zoobank.org:act:362C1FE7-0D4B-4E70-8AB4-8D89A97DDCE3

http://species-id.net/wiki/Operclipygus_ dybasi

Fig. 58F Map 20

#### Type locality.

PANAMA: Chiriquí: Finca Lerida near Boquete [8°47'N, 82°26'W].

#### Type material.

**Holotype female**: “Finca Lerida, near Boquete, Chiriquí Prov., PANAMA, March 14 1959” / “Barca area alt. 5650 ft.” / “CNHM Panama Zool. Exped. (1959) H. S. Dybas leg.”/ “Berlese: floor litter” / “*Phelister* II det. R. Wenzel 19 “ / FMNH-INS 0000069314” / “♀” (FMNH).

#### Diagnostic description.

Length: 2.25 mm, width: 2.00 mm; body rufobrunneus, faintly bicolored, elytra mostly lighter rufescent, with diffuse darker spots at middle on each side of the median suture; broadly rounded, widest at humeri, relatively flattened above, though convex beneath; frons strongly depressed at middle, with conspicuous ground punctation, lacking microsculpture; frontal stria rounded at sides, absent from middle; labrum about 3× as wide as long, strongly emarginate at middle; left mandible with large blunt basal tooth, right mandible with small acute basal tooth; pronotal disk with fine, narrow prescutellar impression, fine ground punctation becoming coarser and denser toward sides, with numerous coarse punctures intermingled at sides; marginal pronotal stria not descending onto hypomeron; lateral submarginal stria obsolete in basal two-thirds, present only in anterior corner, quite close to anterolateral part of marginal stria; anterior submarginal stria present across middle, barely recurved posterad at sides; median pronotal gland openings just over halfway back from anterior pronotal margin; elytra with two complete epipleural striae, outer subhumeral stria present in posterior half, inner subhumeral stria absent, striae 1-4 complete, 5^th^ stria present in apical third, sutural stria present slightly longer; venter with weak microsculpture on prosternum and fairly conspicuously at sides of posterior sterna; prosternal keel shallowly emarginate at base, depressed between coxae, with carinal striae complete, united in narrow anterior arch, with fragmented secondary striae between coxae; prosternal lobe appearing deflexed, marginal stria nearly complete; mesoventrite weakly projecting at front, marginal mesoventral stria broadly interrupted by strongly arched meso-metaventral stria; lateral metaventral stria gently curved laterad posteriorly, extending toward posterior corner of metepisternum; propygidium and pygidium with conspicuous transverse microsculpture; propygidium with sparse small, round punctures separated by about 3× their diameters; pygidium with rather dense ground punctation at sides and apically, with very slightly coarser punctures sparsely intermingled throughout; pygidial sulcus fine, weak, present around apical half of margin only. Male: not known.

#### Remarks.

This species is only known from a single female, but exhibits several distinctive characters, including the bicolored pattern (Fig. 58F), the general breadth of the body and head, and the relatively high density of the ground punctation toward the sides of the pronotum.

#### Etymology.

We name this species for Henry Dybas (1915–1981), formerly of the Field Museum, collector of the unique specimen, and among the published few who have contributed to current understanding of *Operclipygus* diversity.

### Operclipygus pichinchensis sp. n.

urn:lsid:zoobank.org:act:7F98F864-4486-4E79-9F87-2F2E271D2487

http://species-id.net/wiki/Operclipygus_pichinchensis

Figs 58G–H Map 21

#### Type locality.

ECUADOR: Pichincha: Rio Palenque Research Center [0°35'S, 79°22'W].

#### Type material.

**Holotype male**: “ECUADOR LOS RÍOS CCRP [Centro Cientifico Rio Palenque] 7Jan1981 SSandoval” / “Caterino/Tishechkin Exosternini Voucher EXO-02337” (FMNH). **Paratypes** (11): same data as type, except as noted: 1: 27.xii.1980 (CHSM), 2: 1.i.1981 (CHSM, USFQ), 1: 4.i.1981 (CHSM), 4: 23.xii.1980 (FMNH, MSCC, AKTC), 1: 18.vi.1980 (CHSM), 1: 24.ii.1977, T. DeVries (CHSM); 1: Tinalandia, Santo Domingo 16km E, 0°16'53"S, 79°3'39"W, 750m, 26–27.iii.1999, FIT, R. Brooks & D. Brzoska (SEMC).

#### Other material.

**PANAMA: Darién:** 1: Cana Biological Station, 7°45'18"N, 77°41'6"W, 7–9.vi.1996, FIT, J. Ashe & R. Brooks (SEMC).

#### Diagnostic description.

Length: 1.75–2.03 mm, width: 1.47–1.62 mm; body rufescent, elongate oval, widest behind humeri, rather strongly convex, particularly near anterior third of elytra; frons and upper third of epistoma depressed at middle; sides of frontal stria nearly parallel between eyes, sinuate over antennal bases, fragmented but subcontiguous across front; labrum about 2.5× as wide as long, only very weakly emarginate apically; left mandible with weak, blunt basal tooth, right with small acute basal tooth; pronotal disk with very small punctiform prescutellar impression, ground punctation of disk very fine and sparse, with ~12 coarser punctures toward sides; marginal pronotal stria continuous behind head and along lateral margins, not descending onto hypomeron; lateral submarginal pronotal stria continuous with anterior portion across front, arching slightly anterad in pronotal corners, converging to middle of lateral margin, interrupted subbasally, with short basal fragment, pronotum weakly, narrowly depressed behind; elytra with two complete epipleural striae, outer subhumeral stria present in apical half, inner subhumeral stria absent, striae 1-4 complete, 5^th^ stria present in apical half, sutural stria present in apical two-thirds; venter with microsculpture on most of prosternum, mesoventrite, along anterior one-fourth and at sides of metaventrite, and on 1^st^ abdominal ventrite; prosternal keel very weakly emarginate at base, carinal striae complete, narrowing between coxae, united in slightly bulbous anterior arch, secondary carinal striae present between procoxae and prosternal gland openings; mesoventrite with anterior margin straight, marginal stria interrupted at middle; mesometaventral stria strongly arched forward, nearly to mesoventral margin, continued at sides by lateral metaventral striae to outer third of metacoxa; 1^st^ abdominal ventrite with inner lateral stria abbreviated, outer lateral stria present only as sparse basal fragments; propygidium and basal fourth of pygidium with weakly impressed transverse microsculpture, most of pygidium lacking microsculpture; propygidial punctures small, round, separated by about twice their diameters; pygidial punctures smaller, sparser, against sparse, fine ground punctation; marginal pygidial sulcus weak, present on apical third at most, may be fragmented to absent. Male genitalia very similar to those of *Operclipygus hamistrius* (see Figs 57A–D, I), differing as follows: T8 with sides subparallel; S8 shorter, with apices bluntly subacuminate, ventral creases conspicuous; tegmen like that of *Operclipygus arquus* (see Fig. 57K) shorter, widest distinctly basad middle, with sides rounded, not as strongly narrowed to apex.

#### Remarks.

This species appears closely related to a species from Central American highlands, *Operclipygus rufescens*, which also generally has the anterior and lateral portions of the submarginal pronotal striae connected and continuous across front (Fig. 58G), but differs in having this stria more distantly spaced from the margin, as well as having it interrupted at the sides. It also has the marginal pygidial stria very short, fine, and frequently fragmented (likely variably) toward the apex, and the pygidial punctation slightly coarser (Fig. 58H). A single female from Panama: Darién shares all these characters and is tentatively assigned to this species.

#### Etymology.

This species’ name refers to the region of western Ecuador it is known from.

### Operclipygus rufescens sp. n.

urn:lsid:zoobank.org:act:3028663D-08C5-4FEF-9A29-95BA3C3CFDB6

http://species-id.net/wiki/Operclipygus_rufescens

Fig. 59A Map 21

#### Type locality.

COSTA RICA: Puntarenas: Monteverde Reserve [10°13.5'N, 84°49.5'W].

#### Type material.

**Holotype male**: “COSTA RICA: Puntarenas-Guanacaste border, Monte Verde, 1760m” / “10 May 1989, J. Ashe, R. Brooks, R. Leschen, ex. flight intercept trap”/ “Snow Entomol. Mus., Costa Rica Exped #092” / “SEMC0903631 KUNHM-ENT” (SEMC). **Paratypes** (10): **COSTA RICA: Puntarenas:** 1: Monteverde, 1500m, 21.ii–1.iii.1983, FIT, cloud forest, D. Lindemann (SEMC), 1: 1520m, 9.v.1989, FIT, J. Ashe, R. Brooks, R. Leschen (INBIO), 1: 1520m, 11–18.vi.1983, FIT (CHSM), 1: 1520m, 2–9.vii.1983, FIT, D.H. Lindeman (CHSM), 1: 1520m, 21.v.1989, FIT, J. Ashe, R. Brooks, R. Leschen (MSCC), 1: 1610m, 7.vii.1990, FIT, S.E. Roberts (AKTC), 1: 1400m, 9.v.1989, FIT, J. Ashe, R. Brooks, R. Leschen (FMNH), 1: 1400m, 21.v.1989, FIT, J. Ashe, R. Brooks, R. Leschen (SEMC), 1: 1570m, 21.v.1989, FIT, J. Ashe, R. Brooks, R. Leschen (SEMC); 1: Cerras Amigos, 1780m, 21.v.1989, FIT, J. Ashe, R. Brooks, R. Leschen (SEMC).

#### Other material.

**COSTA RICA: Alajuela**: 1: Peñas Blancas, 800m, 19.v.1989, FIT, J. Ashe, R. Brooks, R. Leschen (SEMC); 1: Peñas Blancas, 1420m, 20.v.1989, FIT, J. Ashe, R. Brooks, R. Leschen (SEMC); **Guanacaste:** 2: Parque Nac. Guanacaste, Est. Pitilla [misspelled Patilla], 10°59'22"N, 85°25'33"W, 13–15.vii.2000, FIT, J. Ashe, R. Brooks, Z. Falin (SEMC); **Heredia:** 5: La Selva Biol. Stn., 10°26'N, 84°01'W, 22.vi.1998, FIT, C.E. Carlton & A.K. Tishechkin (LSAM), 6: 23.vi.1998 (LSAM), 2: 24.vi.1998 (LSAM), 3: 25.vi.1998 (LSAM), 1: 26.vi.1998 (LSAM), 7: 21.vi.1998 (LSAM), 2: 28.vi.1998 (LSAM), 4: 29.vi.1998 (LSAM), 2: 24.ii.1992, FIT, W. Bell (SEMC), 1: 3.iii.1992 (SEMC), 1: 22.i.1992 (SEMC), 1: 9.ii.1992 (SEMC), 2: 17.ii.1992 (SEMC), 1: 24.iii.1992 (SEMC), 1: 14.ii.1992 (SEMC), 1: 27.iii.1992 (SEMC), 4: 5–8.iii.2001, FIT, E.G. Riley (TAMU), 1: 8.vii.2001, pitfall, A. Cline (AKTC), 3: 10.vi.2012, OTS Beetle Course, DNA Extracts MSC-2304, MSC-2335, MSC-2336 (SBMNH, MSCC, LSAM), 1: 15.vi.2012, DNA Extract MSC-2323 (LSAM); **Limón:** 1: Area Cons. Tortuguero, Sector Cerro Cocori, Fca. de E. Rojas, 150m, i.1993, E. Rojas, (INBIO), 2: iii.1993 (INBIO), 2: iv.1992 (INBIO), 2: ix.1991 (INBIO), 2: vi.1993 (INBIO), 1: vii.1993 (INBIO), 1: 9–30.xi.1992 (INBIO); 1: Sardinas, Barra del Colorado, 15m, 26.iv–3.v.1995, F. Araya, (INBIO); **Puntarenas:** 8: Las Alturas Biol. Sta., 08°56.17'N, 82°50.01'W, 1660m, 31.v–3.vi.2004, FIT, J.S. Ashe, Z. Falin, I. Hinojosa (SEMC); 3: Est. Biol. Las Alturas, Coto Brus, 8°57'N, 82°52'W, 1600m, 30.iii-3.iv.2002, FIT, A.K. Tishechkin (AKTC, LSAM); 2: Est. Biol. Las Cruces, San Vito, 1200m, 1–30.vii.1982, FIT, B.D. Gill (BDGC), 6: 17.viii-12.ix.1982, FIT, B.D. Gill; 2: Est. Biol. Las Cruces, Coto Brus, 8°47'N, 82°57'W, 1100m, 25–30.iii.2002, FIT, A.K. Tishechkin (AKTC, LSAM), 1: 31.iii–1.iv.2002, FIT, A.K. Tishechkin (LSAM); 4: Altamira Biol. Sta., 09°01.76'N, 83°00.49'W, 1510–1600m, 4–7.vi.2004, FIT, J.S. Ashe, Z. Falin, I. Hinojosa (SEMC); 2: Est. La Casona, 1520m, 15.viii.1994, 23.viii.1994, K.L. Martinez, (INBIO). **PANAMA: Chiriquí:** 6: Hartmann's Finca, 4km N Sta. Clara, 1500m, 30.vi–13.vii.1982, FIT, B.D. Gill (AKTC, BDGC); 1: La Fortuna Dam, 1200m, 14.vi–16.vii.1982, FIT, B.D. Gill (BDGC).

#### Diagnostic description.

Length: 1.87–1.90 mm, width: 1.44–1.47 mm; body rufescent, elongate, ovoid, generally widest behind humeri; frons moderately depressed at middle, lacking microsculpture; central portion of frontal stria varied from complete to fragmented to absent; labrum broad, almost 3× as wide as long, emarginate apically; sides of pronotum usually sinuate in basal third, rounded to subangulate to anterior corner; pronotal disk with fine, sparse ground punctation, lacking coarser lateral punctures, with shallow, poorly defined depression along and behind anterolateral portion of submarginal stria; marginal pronotal stria generally descending to hypomeron at anterior corner, delimiting antennal cavity beneath, often effaced entirely; anterior and lateral portions of submarginal stria generally connected, continuous along anterior and lateral margins, arched forward to near anterior pronotal corner, outward to nearly intersect lateral margin, then diverging from margin slightly, running parallel to margin in basal half; elytra with two complete epipleural striae, with outer subhumeral stria fine, present in apical third, inner subhumeral stria absent, striae 1-4 complete, 5^th^ stria present in apical half, sutural slightly longer; venter with microsculpture on prosternum, mesoventrite, and anterior fourth of metaventral disk, with rudimentary sculpturing on the 1^st^ abdominal ventrite; prosternal keel, mesoventrite, and anterior half of metaventrite markedly depressed, especially in males, posterior half of metaventrite convex and prosternal lobe deflexed; prosternal keel weakly emarginate at base, carinal striae complete, with weak secondary striae; some populations with distinct presternal swelling at base of prosternal lobe; mesoventral margin weakly projecting, with marginal stria interrupted; mesometaventral stria arched forward to near anterior mesoventral margin; lateral metaventral stria extended toward middle of metacoxa, slightly abbreviated apically; 1^st^ abdominal ventrite with single, abbreviated lateral stria; propygidium and basal half of pygidium with conspicuous microscuplture; propygidial punctures very small, irregularly separated by about 5× their diameters; pygidium elongate, about 1.3 times as long as maximum width, with fine ground punctures, few coarser punctures in basal corners; marginal pygidial stria fine, obsolete along basal half or more of margin, often secondarily interrupted. Male gentialia extremely similar to those of *Operclipygus hamistrius* (see Figs 57A–D), differing as follows: T8 broader, with deeper basal emargination; S8 slightly more elongate and with narrower, more attenuate apical guides; S9 with apex slightly narrower and base slightly broader; tegmen (Fig. 57L) very slightly shorter, broader, dorsoventral curvature more even, less abruptly curved ventrad.

#### Remarks.

**Figure 59.**
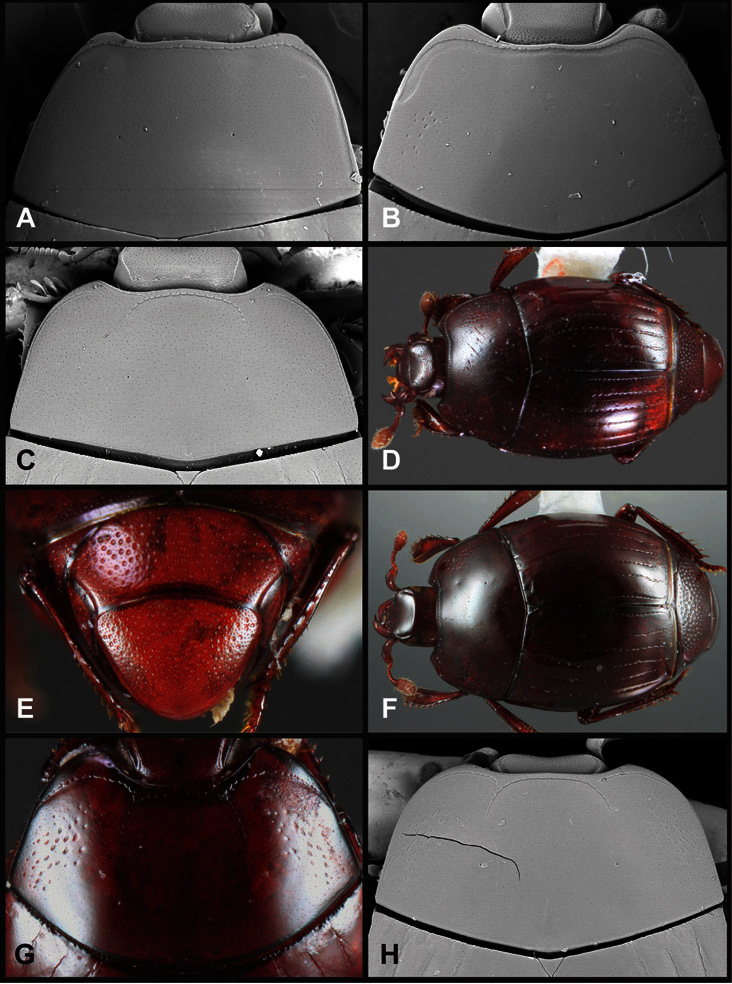
*Operclipygus hamistrius* group. **A** Pronotum of *Operclipygus rufescens* **B** Pronotum of male of *Operclipygus impressicollis* **C** Pronotum *Operclipygus nubosus* **D** Dorsal habitus of *Operclipygus chiapensis* **E** Pygidia of *Operclipygus chiapensis* **F** Dorsal habitus of *Operclipygus propinquus* **G** Pronotum of *Operclipygus intersectus* **H** Pronotum of *Operclipygus troglodytes*.

This species is quite variable in many external characters, and hard to characterize. But most individuals can be recognized by the shallow depression behind the anterior portion of the submarginal pronotal stria (Fig. 59A), in addition to the marginal stria of the pronotum descending to the hypomeron very close to the anterior corners, much further forward than in *Operclipygus hamistrius*. The lateral submarginal pronotal stria also runs very close to the margin in the anterior half, and it is generally continuous with the anterior submarginal stria (not broken and recurved posterad). The type series is limited to those specimens from Monte Verde, Costa Rica, although specimens from other localities agree well in most diagnosticcharacters.

**Map 21.**
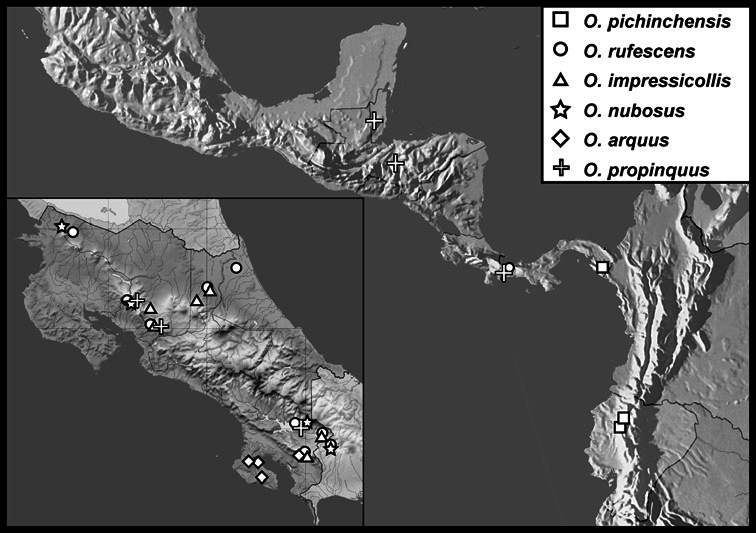
Records of the *Operclipygus hamistrius* group; records for Costa Rica detailed in inset.

#### Etymology.

This species is named for its rufescent coloration, which it shares with many species in the *Operclipygus hamistrius* group.

### Operclipygus impressicollis sp. n.

urn:lsid:zoobank.org:act:BC5F9AF6-1B48-4530-AEB6-96AF414D465E

http://species-id.net/wiki/Operclipygus_impressicollis

Fig. 59A Map 21

#### Type locality.

COSTA RICA: Puntarenas: Las Cruces Biological Station [8°47'N, 82°57'W].

#### Type material.

**Holotype male**: “**COSTA RICA:** Puntarenas, San Vito, Las Cruces, F.I.T. 1,200m. 1–30 July 1982 B.D.Gill leg.”/ “Caterino/Tishechkin Exosternini Voucher EXO-00549” (CNCI). **Paratypes** (10): same data as type, except as noted: 5: 17.vii–12.ix.1982 (BDGC, FMNH, MSCC, AKTC), 1: 7–14.viii.1982 (CHSM); **COSTA RICA: Puntarenas:** 1: Las Alturas Biol. Sta., 08°56.17'N, 82°50.01'W, 1660m, 31.v–3.vi.2004, FIT, J.S. Ashe, Z. Falin, I. Hinojosa (SEMC); **Alajuela**, 2: Peñas Blancas, 800m, 19.v.1989, FIT, J. Ashe, R. Brooks, R. Leschen (SEMC); 1: La Selva Biol. Stn., 10°26'N, 84°01'W, 21.vi.1998, FIT, C.E. Carlton & A.K. Tishechkin (LSAM).

#### Other material.

**COSTA RICA: Puntarenas:** 3: Est. Biol. Las Cruces, San Vito, 1200m, 1–30.vii.1982, FIT, B.D. Gill; 1: Las Alturas Biol. Sta., 08°56.17'N, 82°50.01'W, 1660m, 31.v–3.vi.2004, FIT, J.S. Ashe, Z. Falin, I. Hinojosa (SEMC); **Alajuela**: 1: Peñas Blancas, 800m, 19.v.1989, FIT, J. Ashe, R. Brooks, R. Leschen (SEMC); 1: Reserva Biol. San Ramon, 27km. N. & 8km. W. San Ramon, 10°13'4"N, 84°35'46"W, 810m, 8.vii.2000, FIT, J. Ashe, R. Brooks, Z. Falin (SEMC); **Heredia:** 1: 16km SSE La Virgen, 10°16'N, 84°05'W, 1050–1150m, 9–14.iii.2001, FIT, primary forest, E.G. Riley (TAMU); 1: La Selva Biol. Stn., 10°26'N, 84°01'W, 21.vi.1998, FIT, C.E. Carlton & A.K. Tishechkin (LSAM), 1: 27.vi.1998 (LSAM), 1: 29.vi.1998 (LSAM), 1: 5.ii.1992, FIT, W. Bell (SEMC), 1: 11.iii.1992 (SEMC).

#### Diagnostic description.

Length: 2.00–2.03 mm, width: 1.62–1.72 mm; body rufescent, elongate oval, not strongly convex; frons and epistoma strongly depressed at middle, antennal bases prominent, frontal disk with moderately dense, conspicuous ground punctation; frontal stria rounded at sides, sinuate over antennae, weakened and variably interrupted at front; antennal club elongate, twice as long as wide, and twice as long as funicle; labrum about twice as wide as long, weakly emarginate apically; pronotal disk with very fine punctiform or narrowly linear prescutellar impression, fine, sparse ground punctation, and ~10 coarser lateral punctures; marginal pronotal stria not descending to hypomeron, often effaced in anterior half; submarginal pronotal stria continuous along anterior margin, reaching middle of pronotal side, usually obsolete in basal half; male pronotum with shallow, well-defined depression along and behind anterolateral portion of submarginal pronotal stria, the depression delimited posteriorly by a very finely crenulate stria parallel to submarginal stria which converges to submarginal stria at middle of pronotal side; female pronotum faintly or not at all depressed; elytra with two complete epipleural striae, outer subhumeral stria present in apical third, inner subhumeral stria absent, striae 1-4 complete, 5^th^ stria present in posterior on-third or slightly more, sutural slightly longer; most of venter with microsculpture, only faint to lacking along metaventral midline; prosternal keel weakly emarginate at base, broad, with carinal striae well separated, depressed, prosternal lobe apex appearing slightly reflexed; mesoventrite very weakly projecting at middle, with marginal stria broadly interrupted; mesometaventral stria strongly arched forward to near mesoventral margin; metaventrite markedly convex in middle of basal half (in both sexes); lateral metaventral stria extending toward outer corner of metacoxa, but generally abbreviated just behind middle; lateral striae of 1^st^ abdominal ventrite variable, never complete; propygidium and pygidium both with dense microsculpture; propygidium with moderately large punctures mainly in basal half; pygidium with fine sparse ground punctation, and few slightly larger punctures inconspicuously intermingled; marginal pygidial stria fine, obsolete in basal half or more. Male genitalia essentially indistinguishable from those of *Operclipygus rufescens* (see Figs 57A–D, L).

#### Remarks.

This species is difficult to characterize due to a sexual dimorphism. The males are easy to recognize by a narrow, discrete depression along the anterior half of the lateral submarginal stria, where the depression is delimited by a secondary stria, which abruptly converges to the margin at the middle (Fig. 59B). The depression continues behind the anterior portion of the submarginal stria, but is not as clearly delimited. In the females there is a narrower and vague impression, but this is not very distinct from one present in a couple related species (e.g. Fig. 58A). In both sexes the anterior and lateral portions of the submarginal pronotal stria are continuous, the lateral part ending near the pronotal midpoint. They are also among the most microsculptured species, with strong microsculpture on the pygidium and propygidium, as well as most of the venter, just effaced close to the metaventral midline. Due to slight uncertainty in association, we limit the type series to male specimens.

#### Etymology.

The name of this species refers to the depression behind the submarginal pronotal stria, which is especially noteworthy in males.

### Operclipygus nubosus sp. n.

urn:lsid:zoobank.org:act:662E9676-C894-4BEA-B9B1-EC5E412F9324

http://species-id.net/wiki/Operclipygus_nubosus

Fig. 59A Map 21

#### Type locality.

COSTA RICA: Puntarenas: Monteverde Reserve [10°13.5'N, 84°49.5'W].

#### Type material.

**Holotype male**: “COSTA RICA: Puntarenas Monte Verde, 1520m, 21 May 1989, J. Ashe, R. Brooks, R. Leschen, ex., flight intercept trap” /“Snow Entomol. Mus. Costa Rica Exped. #316” / “SEMC0903669 KUNHM-ENT” (SEMC). **Paratypes** (82): **COSTA RICA: Puntarenas:** 30: Monte Verde, 1520m, 21.v.1989, FIT, J. Ashe, R. Brooks, R. Leschen (SEMC, FMNH, MSCC, AKTC, CHND, INBIO), 2: 1520m, 9.v.1989 (SEMC), 2: 1400m, 21.v.1989 (CHSM), 5: 1520m, 11.v.1989 (SEMC), 1: 1400m, 13.v.1989 (SEMC), 9: 1520m, 14.v.1989 (SEMC), 1: 1400m, 14.v.1989 (SEMC), 4: 1570m, 14.v.1989 (SEMC), 1: 1400m, 24.v.1989 (SEMC), 3: 1520m, 24.v.1989 (SEMC), 1: 1400–1540m, 10.v.1989, pitfall (SEMC), 1: 1520–1570m, 11.v.1989, pitfall (SEMC), 2: 1500m, 21.ii-1.iii.1983, FIT, D.H. Lindeman (CHSM), 1: 1520m, 11–18.vi.1983 (CHSM), 3: 1520m, 2–9.vii.1983 (CHSM), 2: 1520m, 15–23.vii.1983 (CHSM), 1: 1630m, 8.vii.1989, FIT, S.E. Roberts (SEMC), 3: 1630m, 6.vii.1990, FIT, S.E. Roberts (SEMC), 1: 1030m, 27.vi.1990, FIT, S.E. Roberts (SEMC), 3: 1610m, 28.vi.1990, FIT, S.E. Roberts (SEMC), 2: 1610m, 29.vi.1990, FIT, S.E. Roberts (SEMC), 1: 1–2.vi.1993, FIT, C. Michalski (SEMC); 1: Monteverde, Campbell's Woods, 1570m, 11.v.1989, FIT, J. Ashe, R. Leschen, R. Brooks (SEMC); 1: Monteverde, Campbells Bull Pen, 19–29.v.1993, FIT, S. Lingafelter (SEMC); 1: San Luis, Monteverde, 1040m, xi.1993, Z. Fuentes, (INBIO); 1: Res. Biol. Monteverde, San Luis, 1040m, 24.viii–15.ix.1992, F.A. Quesada, (INBIO); 1: Res. Biol. Monteverde, Estacion La Casona, A.C. Arenal, 1520m, vi.1993, N.G. Obando, (INBIO); 18: Est. La Casona, 1520m, 15–23.viii.1994, K.L. Martinez, (INBIO), 6: 3–17.ix.1994, K.L. Martinez, (INBIO); 2: Res. Biol. Monteverde, Est. La Casona, 1520m, 6–25.vi.1994, K.L. Martinez, (INBIO), 1: 5–10.x.1994, K. Martinez, (INBIO).

#### Other material.

**COSTA RICA: Guanacaste:** 3: Parque Nac. Guanacaste, Lado SO Vol. Cacao, 1000–1400m, 21–29.v.1992, M. Ortiz, (INBIO); **Puntarenas:** 4: Est. Pittier, 4.2km SW del Gemelo, 1660m, 8.ix.1999, FIT, R. Gutierrez (INBIO); 1: Est. Pittier, Coto Brus, Send Cerro Pittier, 1670m, 9.ix.1999, FIT, R. Gonzales (INBIO), 1: 30.x–3.xi.1999, FIT, R. Gonzales (INBIO); 2: Est. Biol. Las Cruces, San Vito, 1–30.vii.1982, FIT, B.D. Gill, 1: 17.viii–12.ix.1982, FIT, B.D. Gill (CHSM); 1: Est. Biol. Las Cruces, Wilson Botanical Garden, 1200m, 27.v.1993, FIT, J.S. & A.K. Ashe (SEMC); 2: Est. Biol. Las Cruces, Coto Brus, 8°47'N, 82°57'W, 1100m, 25–30.iii.2002, FIT, A.K. Tishechkin (LSAM); 2: Est. Biol. Las Alturas, Coto Brus, 8°57'N, 82°52'W, 1550m, 3–4.iv.2002, FIT, A. Cline & A. Tishechkin (LSAM); 1: 1600m, 5–7.iv.2002, FIT, A.K. Tishechkin (LSAM); 11: Las Alturas Biol. Sta., 08°56.17'N, 82°50.01'W, 1660m, 31.v–3.vi.2004, FIT, J.S. Ashe, Z. Falin, I. Hinojosa (SEMC); **PANAMA: Chiriquí:** 2: 27.7km W. Volcan, Hartmann's Finca, 8°51'42"N, 82°44'48"W, 1650m, 17–18.vi.1996, FIT, J. Ashe, R. Brooks (SEMC); 6: 27.7km W. Volcan, Hartmann's Finca, 08°45'N, 82°48'W, 1450m, 14–17.vi.1982, FIT, J. Ashe & R. Brooks (SEMC); 19: 4km N Sta. Clara Hartmann's Finca, 1500m, 30.vi–13.vii.1982, FIT, B.D. Gill (AKTC, BDGC).

#### Diagnostic description.

Length: 1.87–2.22 mm, width: 1.56–1.84 mm; body rufescent, ovoid, with outline interrupted at elytral-pronotal corner, prothorax distinctly narrower and more parallel-sided than posterior portion of body; head large, with labrum especially broad, emarginate at apex; frons depressed at middle, frequently with rudimentary fine microsculpture in depression, with sides of frontal stria rounded, variably interrupted at middle; pronotum with lateral margins sinuate near base, subparallel to apex, curving inward near apex, sometimes abruptly so, with discal ground punctation rather conspicuous, especially toward sides, with ~12 coarser punctures at sides; lateral marginal pronotal stria not descending to hypomeron, but frequently effaced for short distance at middle; lateral submarginal pronotal stria complete, close to side, barely curved inward at front; anterior submarginal pronotal stria recurved almost directly posterad at sides, for about one-fifth pronotal length; elytra with outer subhumeral stria present in apical half or less, inner subhumeral stria absent, striae 1-4 complete, 5^th^ stria present in apical third, sutural stria present in apical half; venter with faint microsculpture on prosternum, mesoventrite, and anterolateral corners of metaventrite; prosternum, mesoventrite, and anterior half of metaventrite depressed, posterior half of metaventrite weakly convex; prosternal keel weakly emarginate at base, carinal striae complete, rather widely separated to near apex; prosternal lobe with weak swelling at center of base; mesoventrite very weakly projecting at middle, marginal stria complete to narrowly interrupted; mesometaventral stria strongly arched forward to near mesoventral margin; lateral metaventral stria extending toward middle of metacoxa; 1^st^ abdominal ventrite with two strongly abbreviated lateral striae; propygidium and pygidium both with conspicuous transverse microsculpture; propygidium with small punctures separated by 3–4× their diameters; pygidium with fine ground punctation conspicuous, but few coarser punctures; fine apical marginal stria present only in apical half. Male genitalia essentially indistinguishable from those of *Operclipygus rufescens* (see Figs 57A–D, L).

#### Remarks.

This species is very similar to both *Operclipygus rufescens* and *Operclipygus arquus*, but can be distinguished by the combination of a relatively parallel-sided, basally sinuate pronotum, presence of relatively coarser pronotal ground punctation toward the sides (Fig. 59C), in addition to ~12 distinct punctures, a detached and directly posteriorly recurved anterior submarginal pronotal stria, only briefly anteriorly inturned lateral submarginal pronotal stria, and broad head with large, distinctly emarginate labrum (Fig. 4D). Populations that we assign to this species from Panama are distinctly larger in size, especially head and labrum breadth, have the pronotal ground punctation more conspicuous, and have the aedeagus a little larger, not as distinctly widest near base, more nearly parallel-sided in basal two-thirds. However, all populations show considerable variability in most of these characters, and we chose to unite them under one name for the present.

#### Etymology.

This species’ name refers to the fact that most specimens have been found in cloud-forested areas.

### Operclipygus arquus sp. n.

urn:lsid:zoobank.org:act:DCDF486F-9574-43D1-B391-B1EDFAE5D241

http://species-id.net/wiki/Operclipygus_arquus

Figs 56A, C 57K Map 21

#### Type locality.

COSTA RICA: Puntarenas: Rincon de Osa [8°41.141'N, 83°31.117'W].

#### Type material.

**Holotype male**: “**COSTA RICA**: Punta. Prov, Rincon de Osa, 50m 8°41.141'N, 83°31.117'W, 23–26-VI-2001,S.&J.Peck, 01-13, ex FIT, CR1P01 005” / SM0520653 KUNHM-ENT” (INBIO). **Paratypes** (16): **COSTA RICA: Puntarenas:** 1: Rincon de Osa, 8°41.141'N, 83°31.117'W, 40m, 23–26.vi.2001, FIT, S. & J. Peck (SEMC), 2: 150m, 23–26.vi.2001, FIT, S. & J. Peck (CMNC); 4: Rancho Quemado, Peninsula de Osa, 200m, ix.1992, F. Quesada, (INBIO, FMNH, MSCC, AKTC), 1: 292500, 511000, 200m, v.1991, F. Quesada, (INBIO), 2: v.1992, F. Quesada y G. Varela, (INBIO), 1: x.1992, F. Quesada, (INBIO), 1: xi.1992, M. Segura, (INBIO); 2: Reserva Forestal Golfo Dulce, Est. Agujas, Golfito, Sendero Zamia, 250–350m, 9–12.viii.1998, FIT, A. Azofeifa (INBIO), 2: 250–350m, 11–15.xi.1999, FIT, A. Azofeifa (INBIO).

#### Other material.

**COSTA RICA: Puntarenas:** 6: Est. Biol. Las Cruces, San Vito, 17.viii–12.ix.1982, FIT, B.D. Gill (BDGC, MSCC, AKTC), 3: 1200m, 1–30.vii.1982, FIT, B.D. Gill (BDGC).

#### Diagnostic description.

Length: 2.03–2.12 mm, width: 1.62–1.75 mm; body rufescent, ovoid, sides rounded; frons depressed at middle, prominent over antennae; frontal stria rounded at sides, broadly interrupted in most, with occasional median fragments present; labrum about 2.5× as wide as long, very weakly emarginate apically; pronotum with ground punctation fine and sparse with few, ~6-8 coarse lateral punctures, prescutellar region slightly depressed, but lacking distinct fovea; marginal pronotal stria not descending to hypomeron; pronotum with complete lateral submarginal stria close to margin, lateral margin slightly sinuate toward base; anterior submarginal stria detached, forming more or less continuous arc across front of pronotum, reaching back one-fourth to one-third pronotal length; elytra with outer subhumeral stria present in apical half or less, inner subhumeral stria absent, striae 1-4 very fine but complete, 4^th^ often fragmented toward base, 5^th^ stria present in apical third, sutural stria present in apical half; venter with faint microsculpture almost throughout, effaced on swelling of metaventrite; prosternum, mesoventrite, and anterior half of metaventrite depressed (female with depression of metaventrite less distinct); prosternal keel weakly emarginate at base, with complete carinal striae rather widely separated to near apex; prosternal lobe with weak swelling at center of base; mesoventrite very weakly projecting at middle, with marginal stria broadly interrupted; mesometaventral stria strongly arched forward to near mesoventral margin; metaventrite markedly convex in middle of basal half (in both sexes); lateral metaventral stria extending toward outer corner of metacoxa, but generally abbreviated just behind middle; 1^st^ abdominal ventrite with two strongly abbreviated lateral striae; propygidium and pygidium both with conspicuous transverse microsculpture; propygidium with sparse, moderately large punctures mainly in basal half; male especially with anterior half of pygidium simply, finely sparsely punctate, females with coarser pygidial punctures; fine apical marginal stria present only in apical half. Male genitalia very similar to those of *Operclipygus hamistrius* (see Figs 57A–D), differing as follows: T8 with sides subparallel; S8 shorter, with apices bluntly subacuminate, ventral creases conspicuous; tegmen (Fig. 57K) shorter, widest distinctly basad middle, with sides rounded, not as strongly narrowed to apex; indistinguishable from that of *Operclipygus pichinchensis*.

#### Remarks.

This species is fairly easily recognized in this complex by its broadly arcuate anterior submarginal pronotal stria (Fig. 56C).

#### Etymology.

The name of this species means arc, referring to its curved pronotal stria. It is a noun in apposition.

### Operclipygus propinquus sp. n.

urn:lsid:zoobank.org:act:56101DA5-F124-4536-BD15-18D70E54C977

http://species-id.net/wiki/Operclipygus_propinquus

Fig. 59F Map 21

#### Type locality.

COSTA RICA: Puntarenas: Las Alturas Biological Station [8°57'N, 82°52'W].

#### Type material.

**Holotype male**: “**COSTA RICA**: Puntarenas Prov., Coto Brus, Est. Biol. Las Alturas. 1600m. 8°57'N, 82°52'W. F.I.T. #3. 30.III–4.IV.2002 A.Tishechkin”/ “LSAM 0111357” (FMNH). **Paratypes** (3): **COSTA RICA: Puntarenas:** 1: Est. Biol. Las Cruces, Coto Brus, 8°47'N, 82°57'W, 1000m, 22–30.iii.2003, FIT, A. Cline & A. Tishechkin (LSAM); 1: Monteverde, 1240m, 21.v.1989, FIT, J. Ashe, R. Brooks, R. Leschen (SEMC); 1: **Alajuela**, Peñas Blancas, 875m, 19.v.1989, FIT, J. Ashe, R. Brooks, R. Leschen (SEMC).

#### Other material.

**BELIZE: Cayo:** 1: Gran de Ora, 5.vi.1995, 8.vi.1995, King/Howe/Rosado (BMNH). **HONDURAS: Cortés:** 2: Parque Nac. Cerro Azul-Meambar, Los Pinos, 14°52.4'N, 87°54.7'W, 800m, 10–16.v.2001, FIT, S. Peck (SEMC, CHSM). **PANAMA: Chiriquí:** 1: 4km N Sta. Clara Hartmann's Finca, 1500m, 30.vi–13.vii.1982, FIT, B.D. Gill (BDGC).

#### Diagnostic description.

Length: 2.28–2.53 mm, width: 1.90–2.22 mm; body rufobrunneus, elongate oval, ventrally planar, moderately convex dorsally, almost completely lacking microsculpture, only faint traces at sides of sterna, occasionally faint vestiges on propygidium; frons depressed at middle, with rather sparse ground punctation; frontal stria rounded at sides, variably incomplete across front; labrum about twice as wide as long, emarginate apically, with small off-center process beneath margin; pronotal disk lacking prescutellar impression, with very fine, inconpicuous ground punctation, ~20 coarse punctures at sides; marginal pronotal stria generally descending to hypomeron for short distance, just behind anterior corners; submarginal pronotal stria continuous across front, interrupted or completely obsolete at sides; elytra with two complete epipleural striae, outer subhumeral stria short, present in only apical fourth, inner subhumeral stria absent, striae 1-4 complete (1^st^ may be weak to obsolete at apex), 4^th^ stria arched to near suture at base, 5^th^ stria present in apical fourth, sutural stria present in apical half or slightly more; elytral disk with few very small subserial punctures along the apical margin; prosternal keel weakly emarginate at base, carinal striae weakly converging anteriorly; mesoventral margin weakly projecting at middle, marginal mesoventral stria fine, continuous across anterior margin; mesometaventral stria broadly arched forward to anterior third of mesoventral disk; lateral metaventral stria extending to middle of metacoxa; 1^st^ abdominal ventrite with two abbreviated lateral striae, outer occasionally bent laterad behind metacoxa; punctures at sides of 1^st^ abdominal ventrite slightly coalesced, longitudinally strigose; propygidium with moderately large punctures separated by about their diameters, denser toward base, impunctate in apical fourth; pygidium with fine, sparse ground punctation, with few coarser punctures intermingled at middle; marginal pygidial stria very fine, impressed only along extreme apex. Male: aside from its larger size, there is little to distinguish the male genitalia from those of *Operclipygus hamistrius* (see Figs 56A–D); the sides of the tegmen are very slightly more rounded, and broader toward the base, very much as in *Operclipygus arquus* (see Fig. 57K).

#### Remarks.

This species, while known from few specimens, has a surprisingly broad distribution, from Chiriquí, Panama to western Belize. But the species is quite distinctive within the complex, and all essential characters agree among disparate localities. It can be easily recognized by its general lack of microsculpture, its continuous submarginal pronotal stria (Fig. 59F), the 4^th^ dorsal elytra stria arching to near the elytral suture, and its generally larger and darker body.

#### Etymology.

The name of this species, meaning ‘neighboring’, refers to the distribution across several neighboring countries of Central America.

### Operclipygus intersectus sp. n.

urn:lsid:zoobank.org:act:7E169C87-3126-4122-B246-0FFAC8F3101B

http://species-id.net/wiki/Operclipygus_intersectus

Fig. 59A Map 21

#### Type locality.

PANAMA: Panamá: Chepo-Carti Road [9.28°N, 79.1°W].

#### Type material.

**Holotype male**: “**PANAMA**: Panama, Chepo-Carti Rd. 400m. June 1982 FIT. B.D.Gill.”/ “Caterino/Tishechkin Exosternini Voucher EXO-00331” (CNCI). **Paratypes** (3): **PANAMA: Panamá**: 1: Chepo-Carti Rd., 400m, vi.1982, FIT, B.D. Gill (BDGC); 2: Nusagandi, Ina Igar, 18–21.v.1993, FIT/pitfall, primary forest, E.G. Riley (TAMU).

#### Other material.

**PANAMA: Colón:** 2: Parque Nac. San Lorenzo, Achiote, Cafetal C Dist., 09°11'N, 79°58'W, 250m, 27.vi–11.vii.2007, FIT, A. Mercado (GBFM, AKTC); 1: San Lorenzo Forest, 9°17'N, 79°58'W, 6–8.x.2003, FIT, A.K. Tishechkin (GBFM). **Darién:** 2: Cana Biological Station, Serrania de Pirre, 7°45'18"N, 77°41'6"W, 1200m, 7–9.vi.1996, FIT, J. Ashe, R. Brooks (SEMC, FMNH), 1: 1380m, 4–7.vi.1996, FIT, J. Ashe, R. Brooks (SEMC).

#### Diagnostic description.

Length: 1.90–2.22 mm, width: 1.53–1.87 mm; body rufescent, ovoid, sides more or less evenly rounded; frons depressed at middle, with conspicuous ground punctation; frontal stria rounded at sides, variably interrupted at middle; labrum about twice as wide as long, shallowly emarginate apically; pronotum with sides weakly sinuate near base, disk with fine, sparse ground punctation, with ~15 coarser punctures at sides; marginal pronotal stria broadly interrupted behind head, complete, not descending onto hypomeron at sides; lateral submarginal pronotal stria forming an anterolateral arch, obsolete in basal half, diverging strongly from margin to front, nearly reaching anterior submarginal stria, which forms a long, continuous arch across front, variable in length; venter with pro-, meso- and anterior half of metaventrite depressed, with microsculpture throughout except on posterior metaventral swelling; prosternal keel very weakly emarginate at base, carinal striae convergent to middle, parallel anteriorly, united in narrow arch; mesoventrite faintly projecting at middle, marginal stria fine, complete to narrowly interrupted; mesometaventral stria arched forward nearly to mesoventral margin; lateral metaventral stria extending toward middle of metacoxa, barely abbreviated at apex; 1^st^ abdominal ventrite with two abbreviated lateral striae; propygidium with only faint vestigial microsculpture, pygidium lacking microsculpture; propygidial punctures moderately large and dense in basal half, smaller and sparser to apex; pygidium with small punctures numerous, uniformly scattered to near apex; marginal pygidial stria fine, present along apical half of margin, may be fragmented apically. Male genitalia indistinguishable from those of *Operclipygus arquus* (see Figs 57A–D, K).

#### Remarks.

The distinctive pattern of pronotal submarginal striae (Fig. 59G) distinguishes this species from close relatives in the *Operclipygus hamistrius* group. One of the specimens from Panama: Colón is assigned here which has the anterior portion of the submarginal stria continuous with the lateral, but the distinctive ends of the lateral striae, meeting the margin at an acute angle, associate it with this species with some confidence.

**Map 22.**
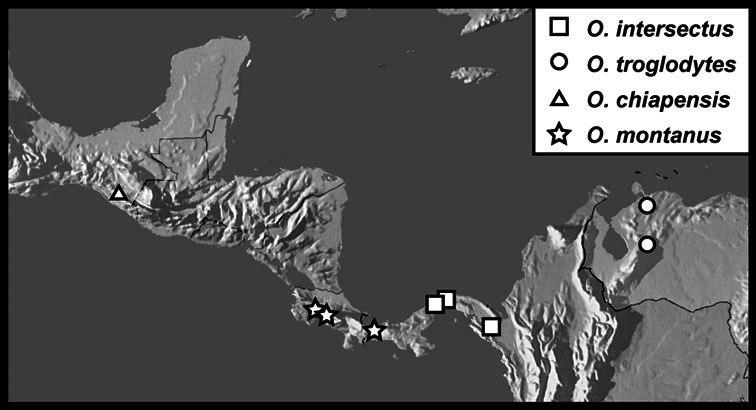
Records of the *Operclipygus hamistrius* group.

#### Etymology.

This species’ name refers to the distinctive intersection of the lateral submarginal pronotal stria with the lateral pronotal margin.

### Operclipygus troglodytes sp. n.

urn:lsid:zoobank.org:act:EC113EAB-17EE-4FD4-97D8-48E7D3DD41C2

http://species-id.net/wiki/Operclipygus_troglodytes

Fig. 59A Map 21

#### Type locality.

VENEZUELA: Falcón: San Luis Mountains [11°13'N, 69°38.5'W].

#### Type material.

**Holotype male**: “VENEZUELA Falcon State, San Luis Mts. in cave, 1973, P.R.F.Chapman, C.I.E. A7179 CC27” (BMNH). **Paratypes** (22): 19: same data as type (BMNH, FMNH, AKTC, MSCC); **VENEZUELA: Lara**, 1: Yacumbu Nat. Park, Sanare, 17.4km SE Yacambu N.P., 9°42'26"N, 69°34'34"W, 1510m, 16–18.v.1998, FIT, J. Ashe, R. Brooks, R. Hanley (SEMC); 2: 18.v–1.vi.1998, FIT, J. Ashe, R. Brooks, R. Hanley (SEMC).

#### Diagnostic description.

This species is extremely similar to the preceding, differing significantly only in the following characters: length: 1.78–2.22 mm, width: 1.47–1.87 mm; frontal stria usually present at middle, interrupted only over antennal bases; labrum broader and more distinctly emarginate; marginal pronotal stria not descending onto hypomeron; lateral submarginal pronotal stria obsolete along most of lateral margin, present only in anterior corners; anterior submarginal stria broadly arched across anterior margin, recurved posterad one-fifth to one-fourth pronotal length; prosternum and mesoventrite not as distinctly depressed, posterior half of metaventrite only weakly convex; propygidium with vestigial fragments of microsculpture; pygidium lacking microsculpture, with relatively conspiuous fine ground punctation and coarser punctures rather dense in basal corners, smaller and sparser toward apex; marginal pygidial stria fine, present on apical half to two-thirds of margin. Male genitalia very similar to those of *Operclipygus hamistrius* (see Figs 57A–D), differing as follows: T8 with sides subparallel; S8 shorter, with apices bluntly subacuminate, ventral creases conspicuous; tegmen shorter, widest distinctly basad middle, with sides rounded, not as strongly narrowed to apex; tegmen like that of *Operclipygus arquus* (see Fig. 57K).

#### Remarks.

This species represents one of the most geographically distant extensions of the *Operclipygus hamistrius* group. However, it is very similar to the preceding species, most readily distinguished by the more strongly abbreviated lateral submarginal pronotal stria (Fig. 59H), which is shorter than the recurved arms of the anterior submarginal stria. Its frontal stria is also more strongly and completely impressed.

#### Etymology.

This species’ name refers to the fact that most specimens were collected in a cave.

### Operclipygus chiapensis sp. n.

urn:lsid:zoobank.org:act:C97943E8-6FDC-4501-8199-8FAA3E145F24

http://species-id.net/wiki/Operclipygus_chiapensis

Figs 57M 59D–E Map 22

#### Type locality.

MEXICO: Chiapas: Cerro El Triunfo Reserve [15°39'N, 92°50'W].

#### Type material.

**Holotype male**: “México: “Reserva Cerro El Triunfo”, Mpio. Ángel Albino Corzo, Chiapas. 07/XI/2008. Bosque Mesófilo. 1500msnm, UTM 0522185 N 1734333W. Pitfall NTP80-1 Calamar. Caballero, U. Col. Ecosur-SCLC”/ “Histeridae sp. 2” / “6935 ECO-SC-E” (ECOSUR). **Paratypes** (2): 1: same data as type (ECOSUR); 1: **MEXICO, Chiapas**, 3km SE Custepec, 15.71582°N, 92.93846°W, 1700m, 17.v.2008, sifted leaf litter, secondary mesophyll forest, DNA Extract MSC-2154 (SEMC).

#### Diagnostic description.

Length: 1.90–2.12 mm, width: 1.68–1.87 mm; body rufobrunneus, ground punctation rather conspicuous throughout, with microsculpture on propygidium, prosternum, mesoventrite, sides of metaventrite; elongate oval, widest at humeri; frons weakly depressed at middle; frontal stria slightly divergent at sides, complete across front; labrum rather broad, convex, emarginate at apex; pronotal disk with numerous (~20) coarse punctures at sides in addition to conspicuous ground punctation; marginal pronotal stria complete across front and along sides; submarginal pronotal stria complete and continuous along front and sides; median pronotal gland openings just over two-thirds pronotal length from anterior margin; elytra with outer subhumeral stria present in apical half, inner subhumeral stria absent, striae 1-4 complete, 5^th^ stria present in apical half, sutural stria slightly longer than 5^th^; elytra with few subserial punctures along apical margin; prosternal keel very weakly emarginate at base, carinal striae complete, convergent to front; prosternal lobe with dense punctures at middle, marginal stria interrupted; mesoventrite barely projecting at front, marginal stria broadly interrupted; mesometaventral stria broadly and strongly arched forward to mesoventral margin; lateral metaventral stria extending to middle of metacoxa; 1^st^ abdominal ventrite with inner lateral stria present in basal two-thirds, outer present along inner edge of metacoxa, weakly bent laterad, strongly abbreviated apically; postcoxal fovea inconspicuous; propygidium with conspicuous microsculpture, fine sparse ground punctation, and uniform small, round punctures, separated by about 1.5× their diameters throughout; pygidium lacking microsculpture, with ground punctures slightly denser than on propygidium, and with coarser punctures smaller than those of propygidium, more densely spaced. Male genitalic segments 8-10 indistinguishable from those of *Operclipygus hamistrius* (see Figs 57A–D); tegmen (Fig. 57M) narrow, elongate, with sides subparallel in basal three-fourths, curved ventrad in apical third; medioventral process weakly sclerotized, forming a rather wide ‘U’, not projecting beneath; basal piece about one-third tegmen length; median lobe about one-third tegmen length, proximal apodemes differentiated, filamentous in proximal third.

#### Remarks.

Within the *Operclipygus hamistrius* group, this species can be recognized by its conspicuousdorsal ground punctation (Fig. 59D), complete elytral striae 1-4, presence of microsculpture on the propygidium, and the coarse punctation of propygidium and pygidium (Fig. 59E).

#### Etymology.

This species’ name refers to its only known state of occurrence in southern Mexico.

### Operclipygus montanus sp. n.

urn:lsid:zoobank.org:act:FA50BE39-2006-4997-BC72-D629A5BCE5F7

http://species-id.net/wiki/Operclipygus_montanus

**Figure 60.**
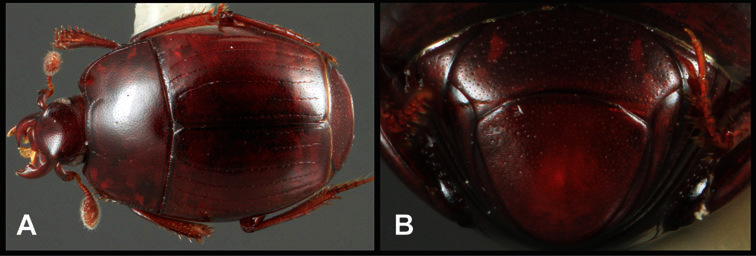
*Operclipygus montanus* (*Operclipygus hamistrius* group). **A** Dorsal habitus **B** Pygidia.

Figs 60A–B 61A–F Map 22

#### Type locality.

COSTA RICA: Puntarenas: Monteverde Reserve [10°13.5'N, 84°49.5'W].

#### Type material.

**Holotype male**: “COSTA RICA: Puntarenas, Monte Verde, Cerro Amigos, 1780m” / “21 May 1989, J. Ashe, R. Brooks, R. Leschen, ex. flight intercept trap”/ “Snow Entomol. Mus., Costa Rica Exped #314” / “SEMC0903662 KUNHM-ENT” (SEMC). **Paratypes** (4): **COSTA RICA: San Jose**: 1: Genesis II Reserve, 09°42.57'N, 83°54.64'W, 2360m, 13–16.2004, FIT, J. Ashe, Z. Falin, I. Hinojosa (SEMC). **PANAMA: Chiriquí**: 3: 6.0 km NE Boquete, 8°48'0"N, 82°26'0"W, 1650m, 14–19.vi1996, FIT, J. Ashe, R. Brooks (SEMC, FMNH).

#### Other material.

**COSTA RICA: Puntarenas:** 1: Monteverde, 1520m, 14.v.1989, FIT, J. Ashe, R. Brooks, R. Leschen (SEMC); 4: Monteverde, Cerro Amigos, 1780m, 21.v.1989, FIT, J. Ashe, R. Brooks, R. Leschen (SEMC, MSCC, AKTC, INBIO); 1: Monteverde Reserve, 1.vi.1993, FIT, C. Michalski (SEMC); **Alajuela**: 3: Peñas Blancas, 1420m, 20.v.1989, FIT, J. Ashe, R. Brooks, R. Leschen (SEMC, INBIO); **San Jose**: 1: Genesis II Reserve, 09°42.57'N, 83°54.64'W, 2360m, 13–16.2004, FIT, J. Ashe, Z. Falin, I. Hinojosa (SEMC). **PANAMA: Chiriquí**: 1: 5.6 km NE Boquete, La Culebra Tr., 8°48'23"N, 82°25'18"W, 1650m, 15–19.vi.1996, FIT, J. Ashe, R. Brooks (SEMC); 1: 5.9 km NE Cerro Punta, 8°22'N, 82°34'W, 2100m, 14–19.vi.1996, FIT, J. Ashe, R. Brooks (SEMC).

#### Diagnostic description.

Length: 2.03–2.22 mm, width: 1.65–1.75 mm; body rufescent, elongate ovoid, strongly convex, especially in anterior third of elytra; frons with conspicuous, fine ground punctation, moderately depressed at middle, frontal stria rounded at sides, absent or fragmented across front; labrum about 3× as wide as long, emarginate apically; pronotal disk with small, vague prescutellar impression, fine sparse ground punctation, few or no coarse lateral punctures, faintly depressed behind middle portion of anterior submarginal stria; marginal pronotal stria not descending to hypomeron, but often obsolete for short distance just in front of lateral midpoint, broadly interrupted behind head; anterior and lateral submarginal striae most often connected and continuous along lateral and anterior margins, may be interrupted behind eye, with anterior part briefly recurved posterad, or may be interrupted for some distance along lateral margin; elytron with dorsal striae rather broadly impressed, especially 4^th^ and 5^th^, 1^st^ and 2^nd^ striae frequently weakened apically, two epipleural striae complete, outer subhumeral stria present in apical half, inner subhumeral stria absent, striae 1-4 complete, 5^th^ stria present in apical half, sutural stria just slightly longer; venter with microsculpture on prosternal keel, in anterior corners of meso- and metaventrites; prosternal keel weakly emarginate at base, carinal striae widely divergent basally, narrowed between coxae, divergent anteriorly, microsculptured within, with secondary strioles alongside; prosternal lobe with weak swelling at middle in front of presternal suture; mesoventral margin very weakly projecting, marginal mesoventral stria weakly interrupted at middle; mesometaventral stria arched strongly forward to near mesoventral margin; lateral metaventral stria extending toward middle of metacoxa; propygidium rather narrow, only about twice as wide as median length, with fine ground punctation and rudimentary transverse microsculpture, with coarse punctures irregularly separated by about twice their diameters; pygidium with similar fine ground punctation, with few very slightly larger punctures sparsely interspersed; marginal pygidial sulcus generally absent, or represented by few weak apical fragments. Male genitalia (Figs 60A–F): accessory sclerites present; T8 elongate, with sides slightly convergent toward apex, basal emargination shallow, basal membrane attachment line distant from it, ventrolateral apodemes well developed, nearly meeting at midline; S8 short, sides divergent and curving strongly ventrad, apices widely divergent and acute, ventrally halves strongly divergent from midline, with weakly sclerotized median vela; T9 with apices relatively broad, subtruncate; T10 small, halves separate; S9 rather short, broad, with broadly triangular, translucent apex, apex arcuate, apical flange narrowed, but not completely interrupted at middle; tegmen short and broad, sides subparallel in basal two-thirds, narrowed to apex, medioventral process thin, widely ‘U’-shaped, forming a strong ventral process; basal piece nearly as long as tegmen, with thick distal apodemes articulating with tegmen; median lobe about half tegmen length, proximal apodemes thick.

#### Remarks.

This species is highly distinctive based on male genitalia (Figs 60A–F), with the short, deflexed S8 unique (and often visible in dried material), and short, broad tegmen. Externally, many more or less distinctive characters vary, but the submarginal pronotal stria is often continuous across the anterior and lateral margins, the marginal pygidial sulcus is generally lacking (Fig. 60B), the pronotum lacks coarse lateral punctures (Fig. 60A), the elytral striae are relatively strongly impressed, and the frontal stria is generally absent between the antennal bases. In fact, there is some variation in tegmen shape among available populations, as well. However, there is no question that they are closely related. The species is sympatric with multiple other highland *hamistrius* group species, so long series must be examined carefully to pick out members of this one. We restrict the type series to males due to their unambiguously unique genitalia.

**Figure 61.**
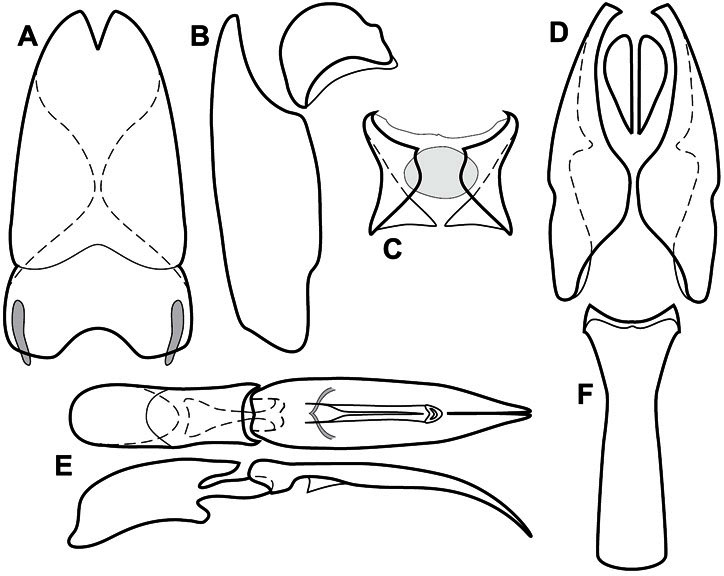
Male genitalia of *Operclipygus montanus* (*Operclipygus hamistrius* group). **A** T8 **B** Lateral view of T8 and S8 **C **S8 (dorsal view) **D** T9 & T10 **E** Aedeagus, dorsal and lateral views **F** S9.

#### Etymology.

This species’ name refers to its occurrence in several widely separated upland localities.

### *Operclipygus plicicollis* group

**Figure 62.**
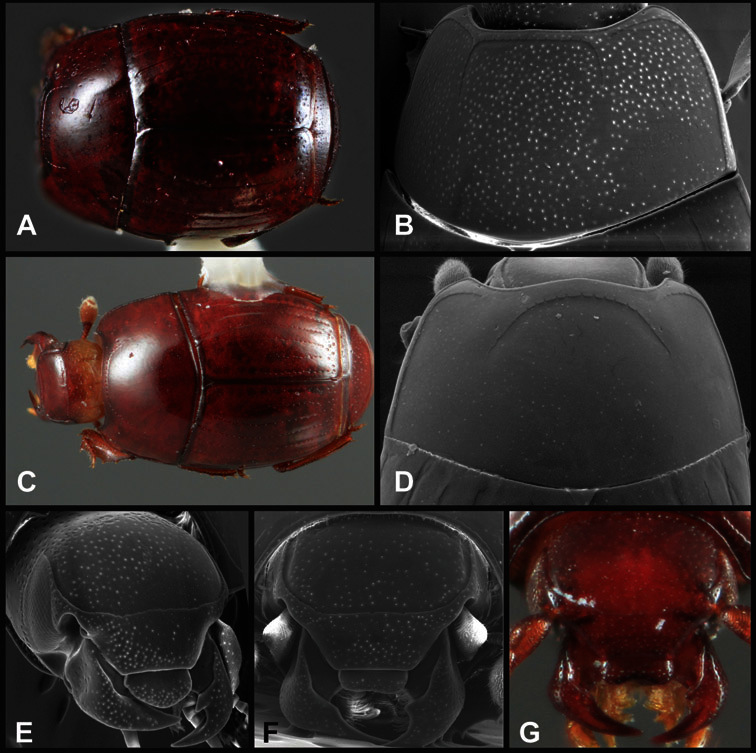
*Operclipygus plicicollis* group. **A** Dorsal habitus of *Operclipygus plicicollis* (lectotype) **B** Pronotum of *Operclipygus plicicollis* **C** Dorsal habitus of *Operclipygus cephalicus* **D** Pronotum of *Operclipygus cephalicus* **E** Frons/mandibles of *Operclipygus plicicollis* **F** Frons/mandibles of *Operclipygus cephalicus* **G** Frons/mandibles of *Operclipygus longidens*.

The *Operclipygus plicicollis* group is easy to recognize on the basis of the small convex body with a disproportionately large head (Figs 62C, D). The species all have mandibles with prolonged apices (Figs 62E–G), as well as a strongly flattened or plicate pronotal base. None has the characteristic pygidial sulcus of *Operclipygus*, but several other characters support the group's inclusion here. Male genitalia do not offer many distinctive characters for the group, but species level characters are found in the shape of the tegmen.

### Key to the species of the *Operclipygus plicicollis* group

**Table:** 

1	Left mandible with strong tooth (Fig. 62E); labrum outwardly arcuate; ends of anterior submarginal pronotal stria recurved straight back about one-third pronotal length (Fig. 62B)	*Operclipygus plicicollis* (Schmidt)
–	Left mandible with small or no tooth; labrum truncate to emarginate; ends of anterior submarginal pronotal stria shorter or recurved obliquely posterolaterad, or both	2
2	Anterior submarginal pronotal stria with ends curving posterolaterad from pronotal margin (Fig. 62D); left mandible with small basal tooth (Fig. 62F); labrum very short, transverse, not emarginate apically	*Operclipygus cephalicus* sp. n.
–	Anterior submarginal pronotal stria with ends bent straight posterad, very short, extending less than one-sixth pronotal length; left mandible completely untoothed (Fig. 62G); labrum short but distinctly emarginate apically (Fig. 62G)	*Operclipygus longidens* sp. n.

### Operclipygus plicicollis

(Schmidt, 1893)

urn:lsid:zoobank.org:act:67800132-5312-47C4-BA6E-395D8A0E1613

http://species-id.net/wiki/Operclipygus_plicicollis

**Map 23.**
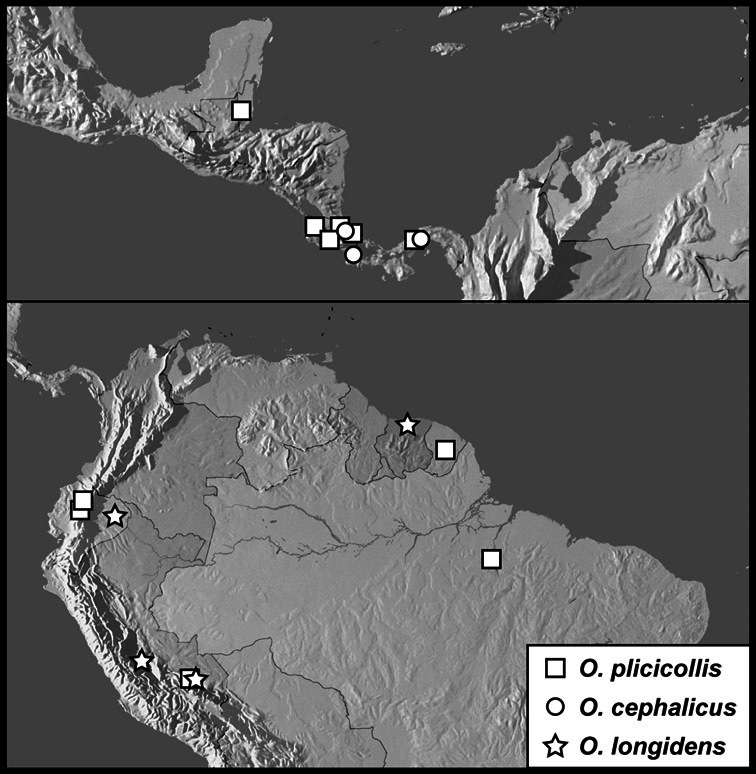
Records of the *Operclipygus plicicollis* group.

Figs 62A–B, E 63A–E Map 23

Phelister plicicollis Schmidt, 1893b: 87; *Operclipygus plicicollis*: Dégallier et al. (2012: 37).

#### Type locality.

Not specified beyond Mexico.

#### Type material.

**Lectotype**, here designated (ZMHB): “Mex.” / “**LECTOTYPE** *Phelister plicicollis* Schmidt, 1893 M.S.Caterino & A.K.Tishechkin des. 2010”. This species was described from an unspecified number of specimens, and the lectotype designation fixes primary type status on the only known original specimen.

#### Other material.

**BELIZE: Cayo:** 1: Las Cuevas Research Station, 550m, v.1997, D. Inward (BMNH), 1: 16.ix.1994, FIT (BMNH). **BRAZIL: Pará:** 1:Tucuruí, 3°45'S, 49°40'W, 7.xii.1983, termitière + fourmis (CHND). **COSTA RICA: Guanacaste:** 1: Parque Nac. Guanacaste, 9km S Santa Cecilia, Est. Pitilla, 700m, 3–18.x.1991, P. Rios, (INBIO); **Heredia:** 2: La Selva Biol. Stn., 10°26'N, 84°01'W, 27.vi.1998, arboreal nest of *Nasutitermes* sp., C.E. Carlton & A.K. Tishechkin (LSAM), 10.vi.2012, FIT, OTS Beetle Course, DNA Extract MSC-2320 (SBMNH); **Limón:** 1: Res. Biol. Hitoy Cerere, Valle de la Estrella, Send. Miramar, 400m, 18–27.ix.2000, FIT, W. Arana (INBIO); 1: Area Cons. Tortuguero, Rio Sardinas, R.N.F.S. Barra del Colorado, 10m, 1–14.ii.1994, F.V. Araya, (INBIO); 2: Santa Clara, Lasmercedes, 200–300m, 12.vi.1928, guest of *Nasutitermes ephratae* Holmgren, F. Nevermann (FMNH); 1: Santa Clara, Hamburg Farm, Parismina Branch, 18.x.1926, *Nasutitermes corniger* (Motschulsky), F. Nevermann (USNM); **Puntarenas:** 1: Res. Biol. Carara, Est. Quebrada Bonita, 50m, i.1994, J.C. Saborio, (INBIO). **ECUADOR: Pichincha:** 2: Rio Palenque Sta., 47km S Santo Domingo, 700ft, 23.v.1975, Broken termite nest, S. Peck (FMNH); 1: Tinalandia, 2.ii.1983, L. Huggert (LUND); 1: Santo Domingo, verst. W. d.Andes, 600m, iv.1965, arboreal termitarium, N. et J. Leleup (FMNH). **FRENCH GUIANA:** 1: Belvèdére de Saül, point de vue, 3°1'22"N, 53°12'34"W, 10.xii.2010, Window trap, SEAG (MNHN). **PANAMA: Colón:** 1: San Lorenzo Forest, STRI crane site, 9°17'N, 79°58'W, 12–13.v.2004, FIT, A.K. Tishechkin, DNA Extract MSC-2225 (AKTC), 1: 14–15.v.2004 (GBFM), 1: 21–24.v.2004 (AKTC), 1: 25–26.v.2004 (AKTC), 1: 26–29.v.2004 (GBFM), 2: 29.ix.2003, sifting arboreal carton nest of *Nasutitermes*, A.K. Tishechkin (AKTC), 1: 6–8.x.2003, FIT, A.K. Tishechkin (GBFM), 1: 30.x–2.xi.2003, FIT, A.K. Tishechkin (AKTC), 1: 11–12.v.2004, FIT, A.K. Tishechkin (AKTC). **PERU: Madre de Dios:** 2: Manu National Park, Rio Alto Madre de Dios, Pantiacolla Lodge, 12°39.3'S, 71°13.9'W, 14–19.xi.2007, FIT, D. Brzoska (SEMC).

#### Diagnostic description.

**Figure 63.**
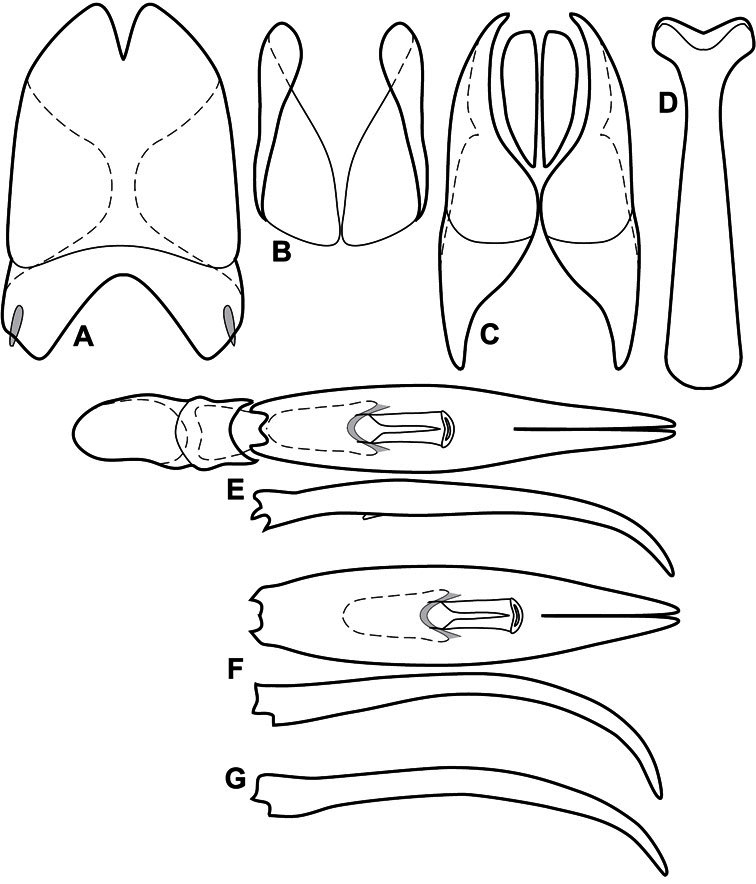
Male genitalia of *Operclipygus plicicollis* group. **A** T8 of *Operclipygus plicicollis* **B** S8 of *Operclipygus plicicollis* **C** T9 & T10 of *Operclipygus plicicollis* **D** S9 of *Operclipygus plicicollis* **E** Aedeagus, dorsal and lateral views, of *Operclipygus plicicollis* **F** Aedeagus, dorsal and lateral views, of *Operclipygus cephalicus* **G** Aedeagus, lateral view, of *Operclipygus longidens*.

Length: 1.44–1.75 mm, width: 1.15–1.47 mm; body rufescent, small, elongate oval, convex, with a disproportionately large head; frons broad, shallowly depressed at middle, antennal bossae moderately prominent, frontal stria with sides more or less parallel between eyes, anterior portion complete, transverse to weakly dorsally arcuate at middle; supraorbital stria present, fragmented, but more or less complete; epistoma convex along apical margin; labrum short, wide, bulging, distal margin rounded; both mandibles with apices prolonged, left mandible with strong, acute basal tooth, right without tooth; pronotal disk without prescutellar impression, but with plicae extending forward about half pronotal length on each side, with fine ground punctation, variably coarsely punctate at sides, with few to many punctures; marginal stria complete around sides and front, though weak behind head; lateral submarginal stria close to edge, curving inward at front but ending freely; anterior submarginal stria recurved to perpendicular at sides, extending about one-fourth pronotal length; median pronotal glands beyond ends of anterior stria, half or more pronotal length from the anterior margin; elytra with two complete epipleural striae, outer subhumeral stria complete, inner subhumeral stria absent, striae 1-4 complete, 5^th^ stria present in apical half to three-fourths, sutural stria slightly shorter; elytral disk with few small punctures near apical margin; prosternal keel emarginate at base, carinal striae fine, complete, joined anteriorly but not posteriorly; prosternal lobe rather short and wide, marginal stria complete; mesoventral margin acutely projecting, marginal stria weak, complete to narrowly interrupted; mesometaventral stria weakly arched forward onto basal half of mesoventral disk, sinuate near mesocoxa, extending posterolaterad toward middle of metacoxa; 1^st^ abdominal ventrite with single lateral stria, impunctate at middle; propygidium with small punctures separated by about twice their diameters in basal half, smaller and very sparse posteriorly; pygidium with only fine sparse ground punctation; pygidial sulcus absent. Male genitalia (Figs 63A–E): accessory sclerites present; T8 with sides subparallel in basal two-thirds, angulate at side, convergent to apex, basal membrane attachment line distinctly distad basal emargination, which is broadly arcuate, ventrolateral apodemes of T8 nearly meeting at middle; S8 short with lateral flanges better developed toward apex, inner edges strongly divergent from base to apex; T9 with sides weakly rounded to apex, apices evenly acuminate, slightly opposing; T10 comprising separate halves; S9 narrowest just basad apex, with broadly rounded base, apical emargination shallow, with single, uninterrupted apical flange; tegmen strongly narrowed in apical third, with broadly rounded ‘U’-shaped medioventral process at basal fourth; median lobe short, proximal apodemes undifferentiated.

**Remarks.** The nominate species in the group is also the most widespread. Its perpendicularly recurved anterior submarginal stria (Fig. 62B), outwardly arcuate labrum (Fig. 62E), and strongly toothed left mandible readily distinguish it from the following two.

### Operclipygus cephalicus sp. n.

urn:lsid:zoobank.org:act:BBB66578-2556-4686-A045-B6C8EF203AFA

http://species-id.net/wiki/Operclipygus_cephalicus

Figs 62C–D, F 63F Map 23

#### Type locality.

COSTA RICA: Puntarenas: Las Cruces Biological Station [8°47'N, 82°57'W].

#### Type material.

**Holotype male**: “**COSTA RICA**: Puntarenas Prov., Coto Brus, Est. Biol Las Cruces. 8°47'N, 82°57'W 1,100m. F.I.T. # 2. 10–11 Apr 2002, A. Tishechkin”/ “Caterino/Tishechkin Exosternini Voucher EXO-00308” (FMNH). **Paratypes** (17): **COSTA RICA: Puntarenas:** 1: Est. Biol. Las Cruces, Coto Brus, 8°47'N, 82°57'W, 1000m, 22–23.iii.2002, FIT, A. Cline & A. Tishechkin (LSAM); 1: P.N. Manuel Antonio, Quepos, 80m, vii.1991, R. Zuniga (INBIO); 1: **Limón:** Area Cons. Tortuguero, Sector Cerro Cocori, Fca. De E. Rojas, 150m, i.1992, E. Rojas, (INBIO). **PANAMA: Colón:** 1: San Lorenzo Forest, 9°17'N, 79°58'W, 27–28.ix.2003, FIT, A.K. Tishechkin (GBFM), 1: 6–8.x.2003 (GBFM), 1: 8–9.x.2003 (AKTC), 1: 19–21.x.2003 (AKTC), 2: 12–13.v.2004, DNA Extract MSC-2225 (MSCC), 1: 15–17.v.2004 (GBFM), 2: 17–18.v.2004 (AKTC), 1: 20–21.v.2004 (AKTC), 1: 21–24.v.2004 (AKTC), 3: 24.v.2004, 25.v.2004 (AKTC, MSCC, FMNH).

#### Diagnostic description.

This species is very similar to *Operclipygus plicicollis*, differing mainly in the following characters: length: 1.22–1.53 mm, width: 0.97–1.25 mm; epistoma not strongly convex; labrum short, transverse, truncate to slightly emarginate apically; left mandible with only weakly developed basal tooth; pronotal disk basally flattened, but without well-developed plicae, disk lacking coarse lateral punctures; anterior submarginal pronotal stria recurved, but broadly arcuate, with ends oblique, to nearly pronotal midpoint; median pronotal gland openings alongside recurved arms of anterior stria, about one-fourth pronotal length from anterior margin; elytra with 5^th^ stria present only in apical third, generally fragmented, sutural stria present in just less than apical half; marginal mesoventral stria interrupted at middle; mesometaventral stria narrowly arched forward, angulate near mesocoxa; 1^st^ abdominal ventrite with only very short inner lateral stria or lacking lateral striae entirely. Male: segments 8–10 identical to those of *Operclipygus plicicollis* (see Figs 63A–D); tegmen (Fig. 63F) broader, with very weak medioventral process, strongly and evenly dorsoventrally curved to apex.

#### Remarks.

This species is easy to distinguish from *Operclipygus plicicollis* by its broadly arcuate anterior submarginal pronotal stria (Fig. 62D), as well as the shape of the labrum (Fig. 62F), and other characters as described.

#### Etymology.

The name of this species refers to the disproportionately large head, common to this small species group.

### Operclipygus longidens sp. n.

urn:lsid:zoobank.org:act:F56A49B6-AF2C-46FC-ADAB-8416EE16307C

http://species-id.net/wiki/Operclipygus_longidens

Figs 62G 63G Map 23

#### Type locality.

ECUADOR: Orellana:Yasuní Research Station [0°40.5'S, 76°24'W].

#### Type material.

**Holotype male**: “**Ecuador:** Napo, mid.Rio Tiputini, Yasuní Res. Stn. 0°40.5'S, 76°24'W, FIT #M1, 28 Jun–5 Jul 1999. AKT#046, C.Carlton & A.Tishechkin”/ “LSAM0045741” / “Yasuní Inventory Exos Genus 5 sp.1 A.K.Tishechkin det. 2007” / “Operclipygus sp #24, Hist 143, Yasuní NP Inventory A.K.Tishechkin det 2010” (FMNH). **Paratypes** (5): 1: same data as type (LSAM); 1: same data as type, except 23–28.vi.1999, FIT, A.K. Tishechkin (AKTC); **ECUADOR: Orellana:** 2:Tiputini Biodiversity Station, 0.6376°S, 76.1499°W, 4–9.vi.2011, FIT, M.S. Caterino & A.K. Tishechkin, DNA Extracts MSC-2281 & MSC-2179 (MSCC, USFQ), 1: 1.viii.2008, FIT, A.K. Tishechkin, DNA Extract MSC-1893 (AKTC).

#### Other material.

**PERU: Madre de Dios:** 1: Manu National Park, Pantiacolla Lodge, Alto Madre de Dios River, 12°39.3'S, 71°13.9'W, 420m, 14–19.xi.2007, FIT, D. Brzoska (SEMC); **Junín**: 1: ~16 k NW Satipo, Rio Venado at 11°11.677'S, 74°46.137'W, 1150m. 3–8.iii.2010, A.V.Petrov (AKTC). **SURINAME: Para**: 1: near Overbridge, 05°31'10"N, 55°04'10"W, 10–14.ii.2010, FIT, W.B. Warner (WBWC).

#### Diagnostic description.

This species is very similar to *Operclipygus plicicollis*, differing mainly in the following characters: length: 1.15–1.28 mm, width: 0.94–1.03 mm; central part of frontal stria arching forward onto epistoma; labrum transverse, distinctly emarginate at apex; left mandible with basal tooth rather small; pronotal disk with basal plicae present, short, with few coarse lateral punctures; anterior submarginal pronotal stria recurved briefly and obliquely at sides, reaching only about one-sixth pronotal length posterad; median pronotal gland openings slightly varied in position, from very close to the anterior margin, in front of curve of anterior submarginal stria, to close to its apex, about 8 puncture widths from the margin; prosternal keel strongly narrowed to front, densely punctate between carinal striae; prosternal lobe convex, also with fine, dense punctures; marginal mesoventral stria interrupted; mesometaventral stria arched strongly forward; propygidium with fine ground punctation rather dense and conspicuous; pygidium with very dense, fine ground punctation. Male genitalia: segments 8–10 identical to those of *Operclipygus plicicollis* (see Figs 62A–D); tegmen (Fig. 63G) broader, with very weak medioventral process, dorsoventral curvature moderate, with abrupt bends near middle and one-fifth from apex.

#### Remarks.

Among the species in this group, this is most easily identified by its very short anterior submarginal pronotal stria, its emarginate labrum (Fig. 62G), and by the dense ground punctation on the pygidium. We limit the type series to those specimens from the vicinity of Rio Tiputini in eastern Ecuador. The specimens from Junín, Peru and Suriname both have much denser punctation on a more quadrate pronotum, and may together be distinct. The specimen from Madre de Dios, Peru, on the other hand, shows little to distinguish it from the types.

#### Etymology.

The name of this species refers to the elongated apices of its mandibles.

### *Operclipygus fossipygus* group

**Figure 64.**
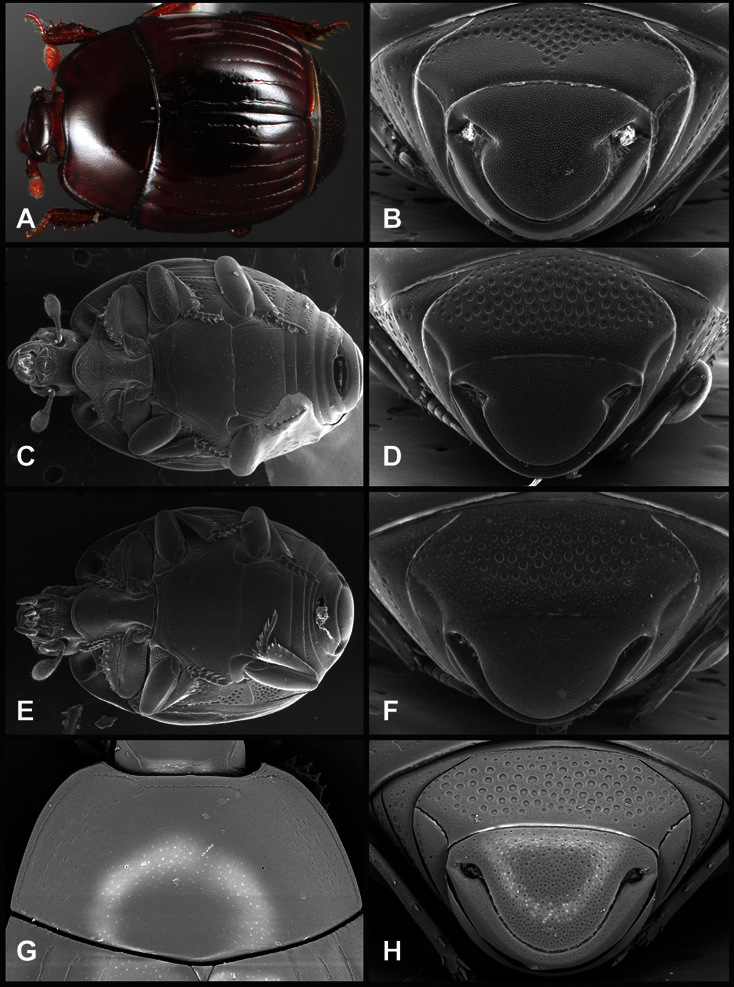
*Operclipygus fossipygus* group. **A** Dorsal habitus of *Operclipygus fossipygus* **B** Pygidia of *Operclipygus fossipygus* **C **Ventral habitus of *Operclipygus foveipygus* **D** Pygidia of *Operclipygus foveipygus* **E** Ventral habitus of *Operclipygus gibbulus* **F** Pygidia of *Operclipygus gibbulus* **G** Pronotum of *Operclipygus fungicolus* **H** Pygidia of *Operclipygus fungicolus*.

**Figure 65.**
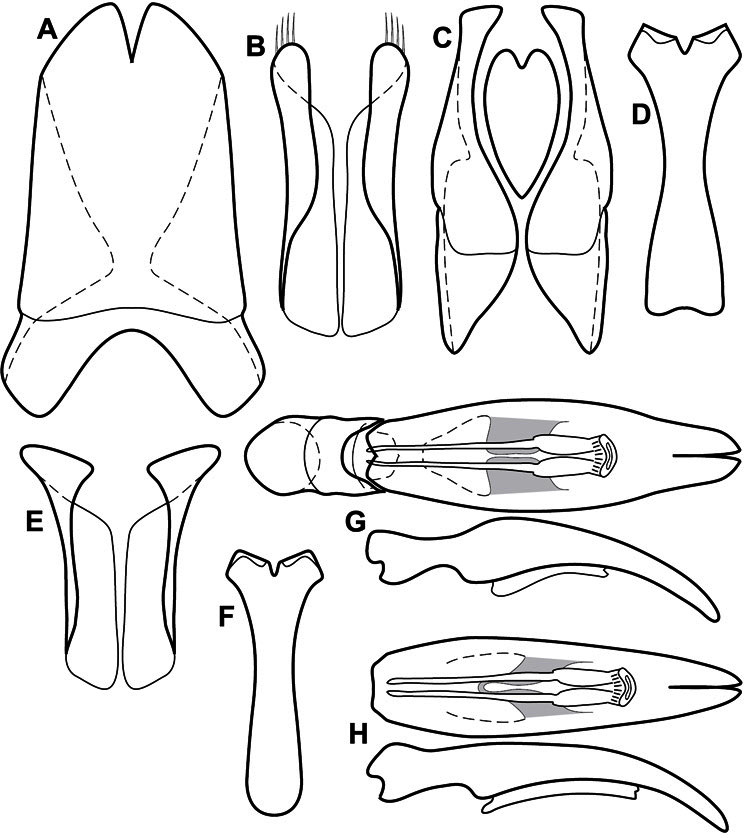
Male genitalia of *Operclipygus fossipygus* group. **A** T8 of *Operclipygus fossipygus* **B** S8 of *Operclipygus fossipygus* **C** T9 & T10 of *Operclipygus fossipygus* **D** S9 of *Operclipygus fossipygus* **E** S8 of *Operclipygus foveipygus* **F** S9 of *Operclipygus foveipygus* **G** Aedeagus, dorsal and lateral views, of *Operclipygus fossipygus* **H** Aedeagus, dorsal and lateral views, of *Operclipygus foveipygus*.

This group of nine species contains the most widely recognized species of the genus. The most commonly collected species in this group have the apical margin of the pygidium deeply sulcate, this sulcus ending in deep foveae on each side (Figs 64B, D, F, H). However, the distinctive male genitalia of such species clearly unite a larger group, some of which have a weak or even no marginal pygidial sulcus (*Operclipygus subdepressus* Schmidt). The aedeagus of species in this group (e.g., Figs 65G–H) is relatively short and broad, with a strong medioventral process. The median lobe has a large gonopore (which appears generally to be exposed beneath, in lateral view) and long, thick proximal apodemes. The halves of the 10^th^ tergite are generally fused, at least over most of their lengths. Members of the *Operclipygus hirsutipes* group are somewhat similar in tegmen and median lobe shapes, and may be related, but they lack a medioventral process and have the halves of the 10^th^ tergite separated. In general, members of the *Operclipygus fossipygus* group are larger than average, weakly depressed, and broadly rounded in overall body form.

### Key to the species of the *Operclipygus fossipygus* group

**Table:** 

1	Pygidium with deep foveae in basal corners (connected by sulcus or not)…2
–	Pygidium lacking deep basal foveae, pygidial sulcus uniform in width or lacking entirely	7
2	Pygidial foveae not connected by marginal sulcus (Fig. 67F)	*Operclipygus disconnectus* sp. n.
–	Pygidial foveae connected by deep marginal sulcus	3
3	Anterior portion of submarginal pronotal stria detached from lateral stria, recurved slightly posterad (Fig. 64G)	*Operclipygus fungicolus* (Wenzel & Dybas)
–	Anterior portion of submarginal pronotal stria connected to lateral stria	4
4	Propygidium with large lateral areas of dense ground punctation only, secondary punctures restricted to basal margin and narrow median band (Fig. 64B); outer subhumeral stria generally interrupted; inner subhumeral stria present for short distance behind middle	*Operclipygus fossipygus* (Wenzel)
–	Propygidium with only small posterolateral areas devoid of secondary punctures; other characters varied	5
5	Inner subhumeral stria absent; Central America	*Operclipygus gibbulus* (Schmidt)
–	Inner subhumeral stria impressed; South America	6
6	Basal pygidial fovea small (Fig. 67E); known from western slope of Ecuadorian Andes	*Operclipygus therondi* Wenzel
–	Basal pygidial fovea large (Fig. 64D); widespread in low and middle-elevations of Amazonia and eastern Andean foothills	*Operclipygus foveipygus* (Bickhardt)
7	Pygidium lacking marginal sulcus (Fig. 67H)	*Operclipygus olivensis* sp. n.
–	Pygidium with marginal sulcus	8
8	Body subdepressed, elongate, parallel-sided, rufescent (Fig. 67A); anterior pronotal margin not at all produced	*Operclipygus subdepressus* (Schmidt)
–	Body rounded, rufo-piceous (Fig. 67C); anterior pronotal margin weakly produced	*Operclipygus simplicipygus* sp. n.

### Operclipygus fossipygus

(Wenzel, 1944)

http://species-id.net/wiki/Operclipygus_fossipygus

**Map 24.**
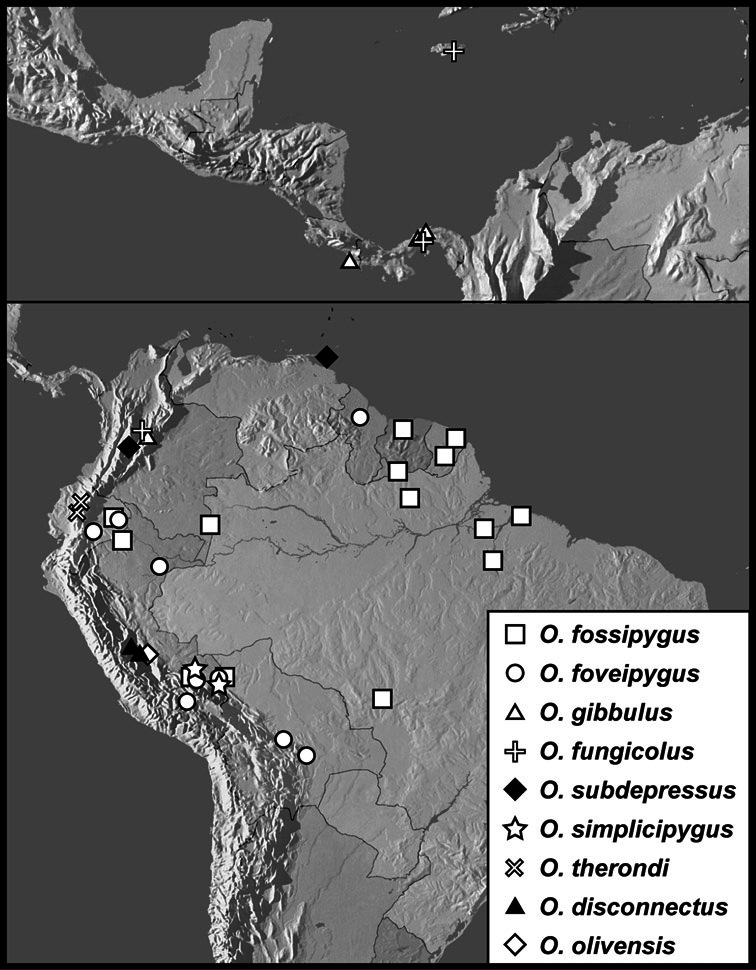
Records of the *Operclipygus fossipygus* group.

Figs 6E 64A–B 65A–D, G Map 24

Phelisteroides fossipygus Wenzel, 1944: 144; *Operclipygus fossipygus*: Wenzel (1976: 258).

#### Type locality.

BRAZIL: Pará: Santarém [2°26'S, 54°42'W].

#### Type material.

**Holotype**: “Santarem Brazil Acc. No. 2966” / “Type Phelisteroides fossipygus Wenzel” (CMNH), examined, 2012.

#### Other material.

**BRAZIL: Amapá:** 1: Serra do Navio, 0°59'N, 52°00'W, 1–14.v. 1991, FIT (CHND); **Mato Grosso:** 1: Tangara da Serra, 14°19.05'S, 57°43.9'W, 640m, 22–29.i.2009, FIT, R.S. Silva (CEMT); **Pará:** 2: Tucuruí, 3°45'S, 49°40'W, iv.1986, FIT (UFPR), 4: vi.1985, FIT (CHND), 1: 16–29.vii.1985, FIT, human dung (CHND); 1: IPEAN, Utinga, Belém, 1°27'S, 48°26'W, v.1985, FIT (CHND), 2: viii.1985, FIT (CHND); 1: Melgaço, Rio Marinaú, 1°51'S, 51°20'W, 31.x–13.xi.1993, FIT (CHND). **COLOMBIA: Vaupés:** 1: Est. Biol. Caparú, Rio Apoporis, 1.1°S, 69.5°W, 27.ix-1.xii.1995, FIT, Black-water terrace forest on sandy soils, B.D. Gill (BDGC); 2: Parque Nac. Mosiro-Itajura (Caparú), Centro Ambiental, 1°04'S, 69°31'W, 60m, 20–30.i.2003, FIT, D. Arias & M. Sharkey (IAVH). **ECUADOR: Orellana:** 3:Yasuní Res. Stn., mid.Rio Tiputini, 0°40.5'S, 76°24'W, 20–29.vi.1999, FIT, C.E. Carlton & V. Moseley (LSAM), 2: 17–23.vi.1999 (LSAM), 1: 22–28.vi.1999 (LSAM), 3: 5–12.vii.1999 (LSAM, FMNH), 1: 7–13.vii.1999 (LSAM), 3: 23–30.vi.1999 (LSAM), 5: 28.vi-5.vii.1999 (LSAM), 3: 4–17.vii.1999 (LSAM), 2: 17–26.vii.1999 (LSAM); 1: 5–10.ix.1999, FIT, primary forest, E.G. Riley (TAMU); 3: Parque Nac. Yasuní, Via Maxus at Puente Piraña, 0°39.5'S, 76°26'W, 14–20.vii.2008, FIT, A.K. Tishechkin (AKTC), 1: 20–24.vii.2008, FIT, A.K. Tishechkin (AKTC); 1: Tiputini Biodiversity Station, 30.vii.2008, Day FIT, A.K. Tishechkin, DNA Extract MSC-1896 (AKTC); 3: Tiputini Biodiversity Station, 0.6376°S, 76.1499°W, 4–9.vi.2011, FIT, M.S. Caterino & A.K. Tishechkin, DNA Extracts MSC-2289, MSC-2175, MSC-2291 (SBMNH, MSCC, USFQ); 2: Tiputini Biodiversity Station, 0°38'0"S, 76°9'0"W, 220m, 5–25.ix.2000, D.J. Inward & K.A. Jackson (BMNH). **FRENCH GUIANA:** 1: Régina, Réserve des Nouragues, 4°2.27'N, 52°40.35'W, 28.i.2010, FIT, SEAG (CHND); 1: Régina, Réserve des Nouragues, 4°2.27'N, 52°40.35'W, 19.ii.2010, FIT, SEAG (CHND), 2: 3.xi.2009, FIT, SEAG (CHND); 1: Roura, 27.4km SSE, 4°44'20"N, 52°13'25"W, 280m, 10.vi.1997, FIT, J. Ashe, R. Brooks (SEMC); 1: Belvèdére de Saül, 3°1'22"N, 53°12'34"W, 30.xi.2010, Window trap, SEAG (MNHN). **PERU: Loreto:** 1: Campamento San Jacinto, 2°18.75'S, 75°51.77'W, 175–215m, 12.vii.1993, FIT, R. Leschen (SEMC); 1: 1.5km N Teniente Lopez, 2°35.66'S, 76°06.92'W, 210–240m, 22.vii.1993, FIT, R. Leschen (SEMC); **Madre de Dios:** 1: Manu National Park, Zona res., Rio Manu, Cocha Juarez, trail nr. Manu Lodge, 18–24.ix.1991, FIT, A. Hartman (FMNH); 4: Tambopata, Reserva Cuzco Amazonico, 15km NE Pto. Maldonado, 12°33'S, 69°03'W, 200m, 22.vi.1989, FIT, J. Ashe & R. Leschen (SEMC), 1: 28.vi.1989, FIT, D. Silva, R.A. Leschen (SEMC), 1: 24.vi.1989, FIT, J. Ashe & R. Leschen (SEMC). **SURINAME**: **Saramacca**: 1: West Suriname Road, 108km WSW Zanderij Airport, 5°13'37"N, 55°52'54"W, 30m, 10–14.vi.1999, FIT, Z. Falin, B. DeDijn (SEMC); **Sipaliwini**: 5: CI-RAP Surv. Camp 2: Sipaliwini River, 2°10.521'N, 56°47.244'W, 210m, 27.viii-1.ix.2010, FIT, T. Larsen & A.E.Z. Short (SEMC); 1: CI-RAP Surv. Camp 3: Wehepai SE Kwamala, 2°21.776'N, 56°41.861'W, 237m, 3–7.ix.2010, FIT, T. Larsen & A.E.Z. Short (SEMC).

#### Diagnostic description.

Length: 1.87–2.25 mm, width: 1.68–2.00 mm; body rufopiceous, ovoid; frons depressed at middle; frontal stria rounded, weakly divergent at sides, generally interrupted over antennal bases, strongly rounded anterad at middle; supraorbital stria weak, fragmented, detached from sides of frontal stria; epistoma convex; labrum about twice as wide as long, asymmetrically emarginate apically; left mandible untoothed, right mandible with small acute basal tooth; pronotum broadly depressed at base, but lacking distinct prescutellar impression, with fine, sparse ground punctation, with few or no larger lateral punctures; anterior pronotal margin distinctly projecting at middle; marginal pronotal stria broadly interrupted behind head; submarginal pronotal stria continuous, complete along lateral and anterior margins; median pronotal gland openings simple, about two-thirds pronotal length from anterior margin; elytra with two complete epipleural striae, outer subhumeral stria complete or interrupted at middle, inner subhumeral stria present in apical half, often shortened at apex, dorsal striae 1-3 complete, 4^th^ and 5^th^ striae similar in length, present in about apical half, sutural stria present in apical three-fourths; prosternal keel weakly projecting at base, carinal striae generally complete, free or united anteriorly; anterior mesoventral margin shallowly emarginate, marginal stria complete; mesometaventral stria weakly arched forward at middle one-third, sinuate near mesocoxa, continued by lateral metaventral stria which curves laterad toward outer corner of metacoxa; 1^st^ abdominal ventrite with two abbreviated lateral striae; postmetacoxal fovea not evident; propygidium with large punctures confined to middle and along basal margin, with fine, dense ground punctation elsewhere; pygidium with fine, dense ground punctation, lacking coarser punctures except along extreme basal margin; marginal pygidial sulcus deep, ending in very large, transversely elongate basal foveae. Male genitalia (Figs 65A–D, G): accessory sclerites absent; T8 more or less parallel-sided, slightly convergent apically, with narrow apical emargination, basal emargination evenly arcuate, basal membrane attachment line distad basal emargination by about one-third its depth, ventrolateral apodemes most strongly developed at their bases, narrowed apically; S8 parallel-sided, with apical guides narrow, evenly developed over most of length, with halves fused; T9 parallel sided in basal two-thirds, convergent to broad, subtruncate apices; T10 with halves finely divided; S9 rather short and broad, sclerotized along midline and along distal edges, with base widened, truncate to weakly emarginate, apical emargination narrow, apical flanges separate; tegmen widest near middle, narrowed to base and apex, strongly curved dorsoventrally, with medioventral process composed of inwardly projecting lateral flanges that are not fused along midline, together forming strong ventral projection; median lobe about three-fourths tegmen length, with proximal apodemes strongly differentiated into thick and thinner proximal portions; basal piece about one-third tegmen length.

#### Remarks.

This is a highly distinctive species, easily recognized by the deep, basal pygidial foveae (Fig. 64B), the dense ground punctation on the sides of the propygidium, and the projecting anterior pronotal margin (Fig. 64A). There is some variation in genitalic structure, exhibited in an individual from Amazonian Peru, especially, in which the spiculum gastrale (S9) is sclerotized along the midline rather than along the margins. However, the unusual form of the aedeagus and its medioventral process is identical with typical *Operclipygus fossipygus*.

### Operclipygus foveipygus

(Bickhardt, 1918)

http://species-id.net/wiki/Operclipygus_foveipygus

Figs 64C–D 65E–F, H Map 24

Phelister foveipygus Bickhardt, 1918c: 243; *Operclipygus foveipygus*: Wenzel (1976: 258).

#### Type locality.

Not specified beyond Bolivia.

#### Type material.

**Holotype** (ZMHB): “Boliv.”/”Type”/”*Phelister pulvillus*” [sic] /”*Phelister foveipygus* Bickhardt n. sp.” (ZMHB), examined 2010.

#### Other material.

**BOLIVIA: Cochabamba:** 4: Est. Biol. Valle Sajta, Univ. San Simon, 67.5km E Villa Tunari, 17°06'19"S, 64°46'57"W, 300m, 9–13.ii.1999, FIT, lowland rain forest, F. Genier (CMNC); 1: 117km E Cochabamba, at Lagunitas, 17°06'22"S, 65°40'57"W, 1000m, 8–12.ii.1999, FIT, montane evergreen forest, F. Genier (CMNC); 1: 117km E Cochabamba, Yungas, Cochabamba-Villa Tunari Rd., 17°6'32"S, 65°41'12"W, 1040m, 1–6.ii.1999, FIT, yungas forest, R. Hanley (SEMC), 1: 6–8.ii.1999, FIT, R. Hanley (CMNC); **Santa Cruz:** 8: 5km SSE Buena Vista, Flora y Fauna Hotel, 17°29.925'S, 63°39.128'W, 440m, 15–24.xii.2003, FIT, S. & J. Peck (CMNC); 1: 5km SSE Buena Vista, Flora y Fauna Hotel, 17°29.92'N, 63°39.13'W, 440m, 24–31.xii.2003, FIT, S. & J. Peck (CMNC) 2: 7–12.v.2004, FIT, A.R. Cline (AKTC), 1: 25.iv.2004, 6.v.2004, FIT, A.R. Cline (AKTC); 2: Amboro National Park, Los Volcanes, 18°06'S, 63°36'W, 1000m, 20.xi-12.xii.2004, FIT, H. Mendel & M.V.L. Barclay (BMNH). **ECUADOR: Napo**: 4: 20km S. Tena, 600m, 11.vii.1976, forest litter, S. Peck (FMNH); **Orellana**: 12: Yasuní Res. Stn., mid.Rio Tiputini, 0°40.5'S, 76°24'W, 17–23.vi.1999, FIT, A.K. Tishechkin (LSAM), 3: 18–23.vi.1999 (LSAM), 10: 20–29.vi.1999 (LSAM), 17: 23–30.vi.1999 (LSAM), 25: 28.vi-5.vii.1999 (LSAM), 2: 2.vii.1999, rotten sticks (LSAM), , 2: 4–17.vii.1999 (LSAM), 12: 5–12.vii.1999 (LSAM), 1: 7–13.vii.1999 (LSAM), 13: 11–18.vii.1999 (LSAM), 2: 12–20.vii.1999 (LSAM), 5: 18–25.vii.1999 (LSAM), 19: 23.vii-4.viii.1999 (LSAM), 1: 26–30.vii.1999 (LSAM), 4: 5–10.ix.1999, FIT, primary forest, E.G. Riley (TAMU); 3: Parque Nac. Yasuní, Via Maxus at Puente Piraña, 0°39.5'S, 76°26'W, 20–24.vii.2008, FIT, A.K. Tishechkin (AKTC), 3: 14–20.vii.2008, FIT, A.K. Tishechkin (AKTC); 25: Tiputini Biodiversity Station, 0.6376°S, 76.1499°W, 2–9.vi.2011, FIT, M.S. Caterino & A.K. Tishechkin, DNA Extract MSC-2172 (SBMNH); 1: Tiputini Biodiversity Station, 0°38.2'S, 76°8.9'W, 27–31.vii.2007, FIT, A.K. Tishechkin (AKTC), 2: 5–25.ix.2000, D.J. Inward & K.A. Jackson (BMNH); 1: Yuturi Lodge, 21.iii.1999, FIT, R. Brooks (CMNC); 5: Payamino Research Station, 0°29'36.01"S, 77°17'29.15"W, 300m, 30.vii-12.viii.2007, FIT, CPDT Gillett (BMNH); **Sucumbíos:** 6: Sacha Lodge, 0°28'14"S, 76°27'35"W, 270m, 21–24.iii.1999, FIT, R. Brooks (SEMC), 1: 24.iii.1999, FIT, R. Brooks (CMNC). **GUYANA: Mazaruni Potaro**: 1: Takutu Mountains, 6°15'N, 59°5'W, 11.xii.1983, Window trap, montane rainforest near logging area, P.J. Spangler, R.A. Faitoute & W.E. Steiner (USNM). **PERU: Cusco:** 1: Consuelo, Manu rd. km 165, 8.x.1982, leaf litter, L. Watrous & G. Mazurek (FMNH), 1: 1.x.1982, litter at rotten logs, L. Watrous & G. Mazurek (FMNH); **Loreto:** 1: km 63, rd. Iquitos - Nauta, Rio Itaya, 4°15.205'S, 73°26.074'W, 140m, 9–13.i.2011, A.V. Petrov (AKTC); **Madre de Dios:** 1: Los Amigos Field Station/CICRA, 12.5624°S, 70.0930°W, 288m, 2–11.i.2007, pitfall, bamboo forest, J. Jacobs (CASC), 23: 20–29.vii.2006, pitfall, bamboo forest, J. Jacobs (CASC), 4: 31.vii-9.viii.2006, pitfall, terra firma forest, J. Jacobs (CASC), 3: 4–15.iii.2007, pitfall, bamboo forest, J. Jacobs (CASC), 1: 272m, 20–29.vii.2006, pitfall, bamboo forest, J. Jacobs (CASC), 2: 284m, 20–29.vii.2006, pitfall, bamboo forest, J. Jacobs (CASC); 2: Los Amigos Field Station, 12.5428°S, 70.1422°W, 281m, 20–29.vii.2006, pitfall, bamboo forest, J. Jacobs (CASC), 1: 31.vii–9.viii.2006, pitfall, bamboo forest, J. Jacobs (CASC); 1: Los Amigos Field Station, 12.5421°S, 70.1435°W, 292m, 20–29.vii.2006, pitfall, bamboo forest, J. Jacobs (CASC), 1: 20–29.vii.2006, pitfall, terra firma forest, J. Jacobs (CASC); 1: Los Amigos Field Station, 12.5657°S, 70.0988°W, 272m, 2–11.i.2007, pitfall, bamboo forest, J. Jacobs (CASC), 2: 31.vii–9.viii.2006, pitfall, bamboo forest, J. Jacobs (CASC), 1: 4–15.iii.2007, pitfall, bamboo forest, J. Jacobs (CASC), 1: 29.vii.2006, pitfall, bamboo forest, J. Jacobs (CASC), 8: 20–29.vii.2006, pitfall, bamboo forest, J. Jacobs (CASC), 1: 2–11.i.2007, pitfall, terra firma forest, J. Jacobs (CASC); 1: Los Amigos Field Station, 12.5458°S, 70.1149°W, 283m, 31.vii-9.viii.2006, pitfall, bamboo forest, J. Jacobs (CASC); 5: Manu National Park, Amazonas Lodge, N. Atalaya, 12°52.2'S, 71°22.6'W, 480m, 10–13.xi.2007, FIT, D. Brzoska (SEMC); 2: Manu National Park, Pantiacolla Lodge, Alto Madre de Dios River, 12°39.3'S, 71°13.9'W, 420m, 14–19.xi.2007, FIT, D. Brzoska (SEMC); 6: Manu National Park, Cocha Cashu Bio. Sta., 11°53'45"S, 71°24'24"W, 350m, 17–19.x.2000, FIT, R. Brooks (SEMC); 2: Manu National Park, Pakitza Bio. Stn., Reserved Zone, Castanal Trail, 11°56'41"S, 71°17'0"W, 317m, 15–16.x.2000, FIT, R. Brooks (SEMC); 2: Manu National Park, Cocha Salvador, Reserved Zone, 12°0'13"S, 71°31'36"W, 310m, 20–21.x.2000, FIT, R. Brooks (SEMC); 3: Tambopata, Reserva Cuzco Amazonico, 15km NE Pto. Maldonado, 12°33'S, 69°03'W, 200m, 20.vi.1989, FIT, J. Ashe & R. Leschen (SEMC), 4: 15.vi.1989, FIT, J. Ashe & R. Leschen (SEMC), 1: 17.vi.1989, Agaricales, J. Ashe, R. Leschen (SEMC), 7: 17.vi.1989, FIT, J. Ashe & R. Leschen (SEMC), 2: 24.vi.1989, Bolete, J. Ashe, R. Leschen (SEMC), 3: 22.vi.1989, FIT, J. Ashe & R. Leschen (SEMC), 7: 24.vi.1989, FIT, J. Ashe & R. Leschen (SEMC), 1: 28.vi.1989, FIT, J. Ashe & R. Leschen (SEMC), 1: 30.vi.1989, FIT, J. Ashe & R. Leschen (SEMC), 1: 2.vii.1989, FIT, J. Ashe & R. Leschen (SEMC), 1: 4.vii.1989, FIT, J.S. Ashe. R. Leschen, D. Silva (SEMC), 1: 13.vii.1989, FIT, J. Ashe & R. Leschen (SEMC), 2: 16.vii.1989, FIT, J. Ashe & R. Leschen (SEMC), 1: 18.vii.1989, Ganoderma, J. Ashe, R. Leschen (SEMC), 1: 16.vii.1989, *Buchenavia* fruit fall, J. Ashe & R. Leschen (SEMC).

#### Diagnostic description.

Length: 1.75–2.37 mm, width: 1.56–2.09 mm; body piceous, broadly rounded; frons shallowly depressed at middle, with fine, sparse ground punctation; frontal stria with sides divergent, rounded, sinuate over antennal bases, arcuate across front; supraorbital stria fine, rounded dorsad, detached from sides of frontal stria; epistoma moderately convex; labrum about twice as wide as long, asymmetrically emarginate apically, projecting beneath; left mandible untoothed, right mandible with small, subacute basal tooth; pronotum lacking prescutellar impression, with fine, sparse ground punctation, lacking lateral pronotal punctures; anterior margin of pronotum very weakly projecting at middle; marginal pronotal stria generally broadly interrupted behind head, may be weak and fragmented; submarginal pronotal stria continuous, complete along lateral and anterior margins, marginal bead convex; median pronotal gland openings about three-fourths pronotal length behind anterior margin; elytra with two complete epipleural striae, outer subhumeral stria complete, inner subhumeral stria present in apical half, fine, dorsal striae 1-3 complete, 4^th^ stria present in apical third or slightly more, 5^th^ stria present in apical half, sutural stria present in apical three-fourths; prosternal keel very weakly projecting at base, carinal striae complete, broadly separated at base, meeting in rather narrow anterior arch; anterior metaventral margin broadly, very shallowly emarginate, marginal stria complete; mesometaventral stria arched forward at middle short of mesoventral midpoint, sinuate laterally, continued obliquely posterolaterad toward outer corner of metacoxa, abbreviated at apex; 1^st^ abdominal ventrite with two lateral striae, both abbreviated posteriorly, with small fovea near inner corner of metacoxa; propygidium with fine, moderately sparse ground punctation and irregularly oval, shallow punctures over most of disk separated by one-fourth their diameters or slightly more; pygidium with fine, dense ground punctation, lacking coarser punctures except along basal margin; marginal pygidial sulcus deep, but rather fine along most of margin, ending in large, transversely oval basal foveae. Male genitalia (Figs 65E–F, H): accessory sclerites absent; T8 about twice as long as broad, basal membrane attachment line slightly distad basal emargination, apical emargination narrow, shallow, ventrolateral apodemes most strongly developed near base, narrowed to apex, not meeting at midline; S8 with apical guides divergent, strongly and somewhat abruptly expanded near apex, bearing a couple setae; T9 with apices weakly convergent, slightly enlarged and apically truncate, ventrolateral apodemes well developed; T10 with halves fused along basal part of midline; S9 broad, evenly widened to base and apex, with distinct apical emargination, apical flanges separate, more strongly sclerotized along midline and lateral margins; tegmen with sides evenly converging from near base to apex, with strong, narrow medioventral process projecting strongly beneath; median lobe large, nearly as long as tegmen, with wide gonopore, proximal apodemes differentiated into short, thick portion, with proximal arms prominent, sinuate; basal piece short, about one-third tegmen length.

#### Remarks.

This species and *Operclipygus fossipygus* are the most commonly collected species that have large pygidial foveae, and they are sympatric over much of Amazonia. *Operclipygus foveipygus* lacks dense punctation on the sides of the propygidium (Fig. 64D), and has coarse secondary punctures more or less uniformly distributed across the propygidium, whereas the coarse punctures are very narrowly limited to the middle of the disk in *Operclipygus fossipygus* (see Fig. 64B).

### Operclipygus gibbulus

(Schmidt, 1889) comb. n.

http://species-id.net/wiki/Operclipygus_gibbulus

Figs 64E–F 66A–C, H Map 24

Phelister gibbulus Schmidt, 1889c: 339.

#### Type locality.

COLOMBIA: Bogotá [uncertain, question-marked on the type label].

#### Type material.

**Lectotype**, here designated: “Columbia, Bogota?”[the question mark is on the label]/ ”*gibbulus* Schm.” / “LECTOTYPE *Phelister gibbulus* Schmidt, 1889 M.S.Caterino & A.K.Tishechkin des. 2010” (ZMHB). This species was described from an unspecified number of specimens, and the lectotype designation fixes primary type status on the only known original specimen.

#### Other material.

**COSTA RICA: Puntarenas:** 1: Parque Nac. Corcovado, Est. Sirena, 0, 100m, ii.1990, G. Fonseca, (INBIO), 1: iv.1990, G. Fonseca, (INBIO), 1: vi.1990, F. Quesada, (INBIO); 1: Parque Nac. Corcovado, Est. Sirena, Send. Espaveles, 0–100m, 10–20.vii.2001, FIT, A. Azofeifa (INBIO); 1: Parque Nac. Corcovado, Est. Sirena, Corcovado Trail, 8°29'7"N, 83°34'39"W, 150m, 28.vi-1.vii.2000, FIT, Z. Falin (SEMC); 1: Rancho Quemado, Peninsula de Osa, 200m, 12–24.v.1993, A. Gutierrez, (INBIO). **PANAMA: Colón:** 1: San Lorenzo Forest, 9°17'N, 79°58'W, 27–28.ix.2003 (LSAM), 1: 12–13.v.2004 (LSAM), 1: 14–15.v.2004 (LSAM), 1: 19–20.v.2004 (LSAM), 4: 21–24.v.2004 (GBFM), 1: 24–25.v.2004 (LSAM), 1: 25–26.v.2004 (LSAM), 1: 9–11.x.2003 (LSAM); 10: Parque Nac. San Lorenzo, Achiote, Cafetal A Dist., 9°12'N, 79°58'W, 250m, 7–21.v.2007, FIT, A. Mercado (AKTC, GBFM); 1: Parque Nac. San Lorenzo, Achiote, Pastizal A Dist., 09°13'N, 80°01'W, 100m, 12–27.i.2008, FIT, A. Mercado (GBFM); **Panamá**: 1: Barro Colorado Isl., iv-x.1947, Berlese funnel, Zetek (USNM), 1: 19.ii-9.iii.1975, Berl. Rotten flowers of *Pseudobombax*, Lawrence, Erwin (FMNH), 1: 4.viii.2000, FIT, S. Chatzimanolis (SEMC), 1: 27–31.vii.2000, FIT, S. Chatzimanolis (SEMC), 1: 40m, 18–22.vi.2000, FIT, S. Chatzimanolis (SEMC), 1: 16.vii.1994, FIT, D. Banks, 1: 7.vii.1994, FIT, D. Banks, 1: 4.viii.1994, FIT, D. Banks; 1: Cerro Azul, ca., 2000ft, 21.ii.1976, flood debris, A.F. Newton (FMNH); 1: Old Plantation Rd. 6.9km S Gamboa, 09°05'N, 79°40'W, 80m, 7–22.vi.1995, FIT, J. Ashe, R. Brooks (SEMC); 1: Nusagandi Reserve, 09°21'N, 78°59'W, 350m, 16–17.v.1995, FIT, J. & A. Ashe (SEMC).

#### Diagnostic description.

**Figure 66.**
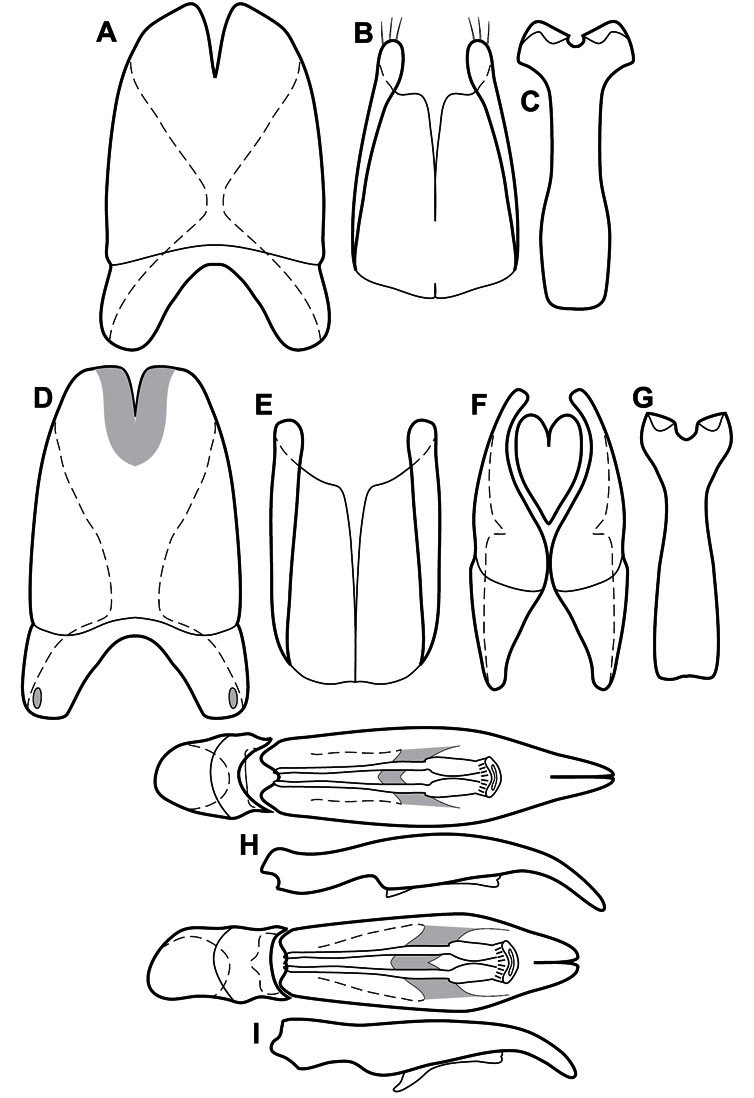
Male genitalia of *Operclipygus fossipygus* group. **A** T8 of *Operclipygus gibbulus* **B** S8 of *Operclipygus gibbulus* **C** S9 of *Operclipygus gibbulus* **D** T8 of *Operclipygus fungicola* **E** S8 of *Operclipygus fungicola* **F** T9 & T10 of *Operclipygus fungicola* **G** S9 of *Operclipygus fungicola* **H **Aedeagus, dorsal and lateral views, of *Operclipygus gibbulus* **I** Aedeagus, dorsal and lateral views, of *Operclipygus fungicola*.

Length: 1.90–2.25 mm, width: 1.68–1.97 mm; body rufopiceous, broadly rounded; frons shallowly depressed at middle, with fine, sparse ground punctation; sides of frontal stria divergent, rounded, sinuate over antennal bases, arcuate across front; supraorbital stria fine, rounded dorsad, detached from sides of frontal stria; epistoma moderately convex; labrum about twice as wide as long, asymmetrically emarginate apically, projecting beneath; left mandible untoothed, right mandible with small, subacute basal tooth; pronotum lacking prescutellar impression, with fine, sparse ground punctation, lacking lateral pronotal punctures; anterior margin of pronotum very weakly projecting at middle; marginal pronotal stria generally broadly interrupted behind head; submarginal pronotal stria continuous, complete along lateral and anterior margins, marginal bead convex; median pronotal gland openings about three-fourths pronotal length behind anterior margin; elytra with single complete epipleural striae, outer subhumeral stria complete, sinuate at middle, inner subhumeral stria absent, subhumeral region strongly, narrowly convex, dorsal striae 1-3 complete, 4^th^ and 5^th^ striae similar in length, present in about apical third, sutural present in apical two-thirds; prosternal keel broad, very weakly projecting at base, carinal striae complete, broadly separated at base, meeting in broad anterior arch; anterior metaventral margin broadly, very shallowly emarginate, marginal stria complete or narrowly interrupted; mesometaventral stria broadly arched forward at middle to near marginal mesoventral stria, continued obliquely posterolaterad, abbreviated apically; 1^st^ abdominal ventrite with two lateral striae, inner usually complete, curved mediad posteriorly, outer stria abbreviated, a small fovea present near inner corner of metacoxa; propygidium with fine, moderately sparse ground punctation and irregularly oval, shallow punctures over most of disk except posterolateral corners, separated by one-fourth their diameters or slightly more; pygidium with fine, dense ground punctation, lacking coarser punctures except for few small punctures along basal margin; marginal pygidial sulcus deep, but rather narrow along most of margin, ending in large, more or less round basal foveae. Male genitalia (Figs 66A–C, H): accessory sclerites absent; T8 with sides weakly convergent to subtruncate apex, apical emargination narrow, deep, basal emargination narrowly rounded, nearly reaching basal membrane attachment line, ventrolateral apodemes most strongly developed basally, narrowed to apex; S8 with sides weakly convergent to apex, apical guides narrow, widening only slightly to apex, halves weakly fused along most of ventral midline; T9 with sides subparallel in basal half, convergent to broad, subtruncate, weakly convergent apices; halves of T10 almost completely fused, with only very narrow apical emargination between; S9 rather short, narrowed at middle, widening slightly to subtruncate base, apical emargination shallow, apical flanges separate; tegmen short, broad, widest just distad midpoint, narrowing weakly to base and more strongly to apex, with strong dorsoventral curvature, medioventral process projecting beneath, about one-third from tegmen base; median lobe prominent, about three-fourths tegmen length, proximal apodemes strongly differentiated proximad gonopore; basal piece short, about one-fourth tegmen length.

#### Remarks.

This species is closely related, and very similar to, *Operclipygus foveipygus*. Both have large basolateral pygidial foveae and lack dense ground punctation on the sides of the propygidium (as found in *Operclipygus fossipygus*). *Operclipygus gibbulus*, however, lacks any indication of the inner subhumeral elytral stria, and has the mesometaventral stria (Fig. 64E) arched more broadly and further anterad than in *Operclipygus foveipygus* (Fig. 64C).

### Operclipygus fungicolus

(Wenzel & Dybas, 1941)

http://species-id.net/wiki/Operclipygus_fungicolus

Figs 64G–H 66D–G, I Map 24

Phelisteroides fungicolus Wenzel & Dybas, 1941: 452; *Operclipygus fungicolus*: Wenzel (1976: 258).

#### Type locality.

COLOMBIA: Cundinamarca: Puerto Salgar [5°28'N, 74°39'W].

#### Type material.

**Holotype female**: “P’to. Salgar, Cund. Colomb., VII-31-38” (FMNH), examined 2006.

#### Other material.

**JAMAICA**: 1: Newport, 16.ii.1969, in rotten orange, J.H. Frank (BMNH). **PANAMA: Colón:** 1: San Lorenzo Forest, 9°17'N, 79°58'W, 22.ix.2003, Winkler extraction, forest litter, A. Dejean, G. Orivel, B. Cobrara, H.-P. Aberlenc & M. Leponce (GBFM), 1: 8–9.x.2003, FIT, A.K. Tishechkin (LSAM), 1: 26–27.x.2003, FIT, A.K. Tishechkin (LSAM), 2: 22–24.x.2003, FIT, A.K. Tishechkin (GBFM), 1: 30.x-2.xi.2003, FIT, A.K. Tishechkin (LSAM); 7: Parque Nac. San Lorenzo, Achiote, Cafetal A Dist., 09°12'N, 79°58'W, 0m, 26.vi-10.vii.2007, FIT, A. Mercado (AKTC, GBFM), 2: 50m, 7–21.v.2007, FIT, A. Mercado (AKTC, LSAM); 1: Parque Nac. San Lorenzo, Achiote, Pastizal B Dist., 09°12'N, 79°59'W, 10m, 12–27.v.2008, FIT, A. Mercado (GBFM); 1: Parque Nac. San Lorenzo, Achiote, Cafetal C Dist., 09°11'N, 79°58'W, 100m, 27.vi-11.vii.2007, FIT, A. Mercado (LSAM); **Panamá**: 1: Canal Zone, Gamboa, 1 1/2 km N, 9°07'N, 79°42'W, 17.vii.1972, cut *Luehea* on ground in forest, G. Otis (USNM); 1: Howard Air Force Base, 08°20'08"N, 79°50'52"W, 10m, 15–20.vii.1999, FIT, A. Gillogly & J. Woolley (TAMU).

#### Diagnostic description.

Length: 1.84–2.00 mm, width: 1.59–1.68 mm; body rufopiceous, elongate oval; frons weakly depressed; frontal stria with sides divergent, weakly sinuate over antennal bases, arcuate, complete across front; supraorbital stria weak, usually present only at middle, disconnected from sides of frontal stria; epistoma weakly emarginate apically; labrum about twice as wide as long, very weakly asymmetrically emarginate; pronotal sides convergent weakly rounded in basal two-thirds; prescutellar impression absent; ground punctation fine, sparse, with ~20 coarse lateral punctures; marginal pronotal stria complete along sides, very weak across front, usually fragmented or interrupted; lateral submarginal pronotal stria complete along side, curved inward anteriorly, nearly meeting anterior submarginal stria, which is weakly arcuate, barely recurved posterad at sides; median pronotal gland openings simple, situated behind ends of recurved anterior stria, about two-thirds pronotal length from anterior margin; elytra with three epipleural striae, lateral-most weakly abbreviated at base, outer subhumeral stria usually complete, fragmented to abbreviated basally, inner subhumeral stria present in apical half, dorsal striae 1-3 complete, 4^th^ stria present in apical two-thirds, 5^th^ stria present in apical half, sutural stria present in apical three-fourths; prosternal keel weakly projecting at base, carinal striae complete, evenly convergent to anterior arch, microsculptured within; mesoventral margin broadly emarginate, marginal stria complete; mesometaventral stria strongly, narrowly arched forward, nearly meeting marginal mesoventral stria, sinuate at sides, continued by lateral metaventral stria toward outer third of metacoxa; 1^st^ abdominal ventrite with two lateral striae, inner stria complete, outer stria curving laterad behind metacoxa, ending in or barely passing through small lateral postmetacoxal fovea; propygidium with sparse ground punctation and moderately large, round punctures separated by slightly less than their diameters; pygidium with fine, dense but rather shallow ground punctation, with slightly coarser punctures interspersed; marginal pygidial sulcus deep, narrow along most of margin, divergent from margin slightly toward base, ending in large, deep basal foveae. Male genitalia (Figs 66D–G, I): accessory sclerites present, small; T8 with sides evenly convergent to subtruncate apex, with deep, narrow apical emargination, basal emargination evenly arcuate, nearly meeting basal membrane attachment line, ventrolateral apodemes most strongly developed basally, evenly narrowed to apex; S9 more or less parallel-sided, with apical guides narrow, evenly developed in apical two-thirds, halves approximate along most of ventral midline; T9 parallel-sided in basal half, convergent to rather broad, blunt apices; T10 cordate, halves fused in basal two-thirds, with narrow apical emargination; S9 rather short and broad, sclerotized along midline, narrowed at middle, base broad, rounded, with weak median emargination, apex with broad emargination, apical flanges separate; tegmen short, broad, widest about one-third from apex, evenly narrowed to base, with strong mediobasal process projecting beneath about one-third from base; median lobe large, nearly as long as tegmen, with wide gonopore, proximal apodemes differentiated into short thick portion, with proximal arms prominent, sinuate; basal piece short, about one-third tegmen length.

#### Remarks.

This is the only species that has basolateral pygidial foveae and has the submarginal pronotal stria broken behind the eye (Fig. 64G). The pygidial foveae (Fig. 64H) are also relatively small compared to the other common foveate species (*Operclipygus fossipygus*, *Operclipygus gibbulus*, and *Operclipygus foveipygus*.)

### Operclipygus subdepressus

(Schmidt, 1889)

http://species-id.net/wiki/Operclipygus_subdepressus

**Figure 67.**
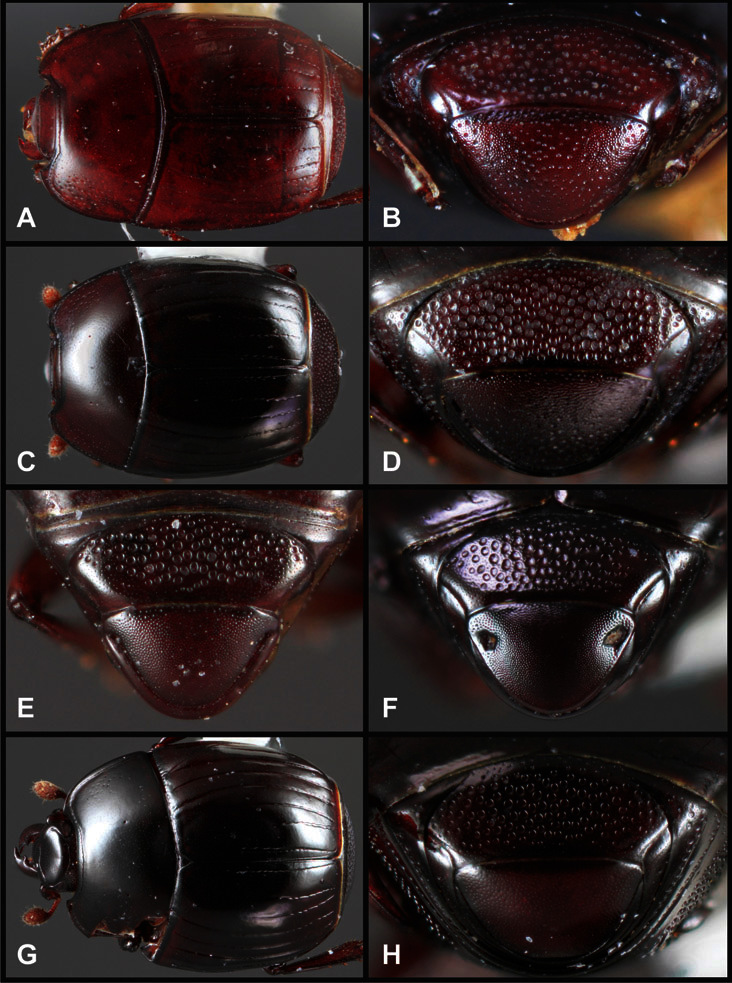
*Operclipygus fossipygus* group. **A** Dorsal habitus of *Operclipygus subdepressus* **B** Pygidia of *Operclipygus subdepressus* **C** Dorsal habitus of *Operclipygus simplicipygus* **D** Pygidia of *Operclipygus simplicipygus* **E** Pygidia of *Operclipygus therondi* **F** Pygidia of *Operclipygus disconnectus* **G** Dorsal habitus of *Operclipygus olivensis* **H** Pygidia of *Operclipygus olivensis*.

Figs 67A–B 68A–E Map 24

Phelister subdepressus Schmidt, 1889c: 337; *Pseudister subdepressus*: Bickhardt (1917: 165); *Operclipygus subdepressus*: Wenzel (1976: 260).

#### Type locality.

Not specified beyond Colombia.

#### Type material.

**Lectotype**, here designated: “Columb” / “67882” / “Type” / “*Phelister subdepressus* Schm. typ.” / “LECTOTYPE *Phelister subdepressus* Schmidt, 1889 M.S. Caterino & A.K. Tishechkin des. 2010” (ZMHB). This species was described from an unspecified number of specimens, and the lectotype designation fixes primary type status on the only known original specimen.

#### Other material.

**TRINIDAD**: 1: xi.1903, B.W. Indies, G.E. Bryant (BMNH)

#### Diagnostic description.

**Figure 68.**
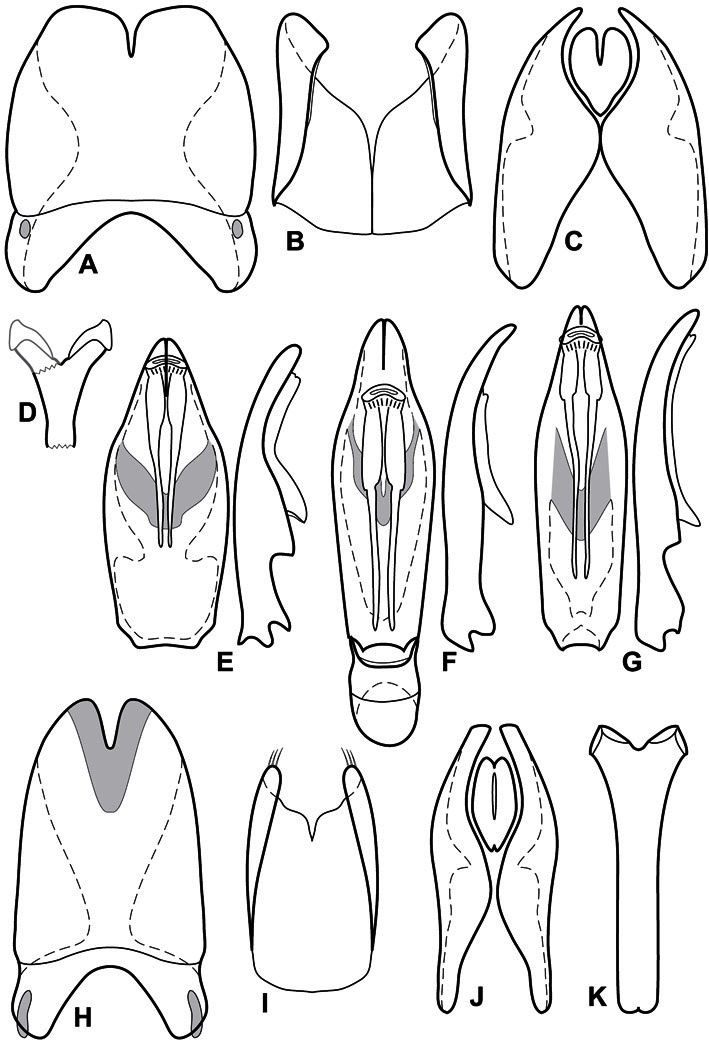
Male genitalia of *Operclipygus fossipygus* group. **A** T8 of *Operclipygus subdepressus* **B** S8 of *Operclipygus subdepressus* **C** T9 & T10 of *Operclipygus subdepressus* **D** S9 of *Operclipygus subdepressus*, (stem and head both broken in only available male; presumed reconstruction shown in gray) **E** Aedeagus, dorsal and lateral views, of *Operclipygus subdepressus* **F** Aedeagus, dorsal and lateral views, of *Operclipygus simplicipygus* **G** Aedeagus, dorsal and lateral views, of *Operclipygus disconnectus* **H** T8 of *Operclipygus disconnectus* **I** S8 of *Operclipygus disconnectus* **J** T9 & T10 of *Operclipygus disconnectus* **K** S9 of *Operclipygus disconnectus*.

Length (nontype only measured): 2.12 mm, width: 1.68 mm; body rufescent, elongate, subdepressed, approximately parallel-sided; frons weakly depressed, frontal stria divergent at sides, almost evenly arcuate across front, weakly sinuate above antennal bases; supraorbital stria absent; epistoma flat; labrum about twice as wide as midline length, apex asymmetrical, shorter on right side than left; left mandible slightly produced at base, right with acute basal tooth; pronotum lacking prescutellar impression, disk with fine, inconspicuous ground punctation, with numerous (>20) coarser punctures along sides; marginal pronotal stria broadly interrupted behind head; submarginal pronotal stria continuous along lateral and anterior margins, but weakly angulate behind eyes; median pronotal gland openings simple, slightly less than one-half pronotal length behind anterior pronotal margin; elytron with two complete epipleural striae, outer subhumeral stria interrupted at middle, inner subhumeral stria present only in apical half, striae 1-3 complete, 4^th^ stria present in just over apical half, 5^th^ stria present in apical half, sutural stria present in apical two-thirds; elytral disk with irregular series of apical marginal punctures; pronotal keel projecting at base, carinal striae complete, sinuate, meeting in narrow anterior arch; anterior mesoventral margin distinctly emarginate, marginal stria complete or interrupted at middle; mesometaventral stria displaced anterad, transverse across middle of mesoventrite, bend posterolaterad, continued by lateral metaventral stria toward middle of metacoxa; 1^st^ abdominal ventrite with inner lateral stria complete, outer abbreviated, without postmetacoxal fovea; propygidium with ground punctation fine, sparse, densely covered with small coarse punctures, separated by much less than their diameters; pygidium with dense, fine ground punctation, with numerous coarser punctures interspersed, more densely toward apex; marginal pygidial sulcus complete, moderately deep, weakly crenulate. Male genitalia (Figs 68A–E): accessory sclerites present, very small, not basal; T8 broad and short, sides curving to subtruncate apex; apical emargination narrow, moderately deep, basal emargination broadly arcuate, basal membrane attachment line distad basal emargination by about one-fourth its depth, ventrolateral apodemes most strongly developed basally, narrowed to apex; S8 more or less parallel-sided, apical guides narrow at base, broadening to rounded apices, with distinctly membraneous inner edges, halves narrowly fused at extreme ventral base, divergent to apex; T9 with sides rounded, convergent to subacute, weakly convergent apices; T10 cordate, fused along midline in basal half; S9 [of only available male broken] with stem narrowed near apex, with narrow apical emargination, apical flanges separate; tegmen short and broad, widest about one-third from apex, with extremely large and projecting medioventral tooth, in addition to distinct ventrolateral cusp on body of tegmen; median lobe with wide gonopore, long proximal apodemes, showing weak differentiation; basal piece about one-third tegmen length.

#### Remarks.

The aedeagus (Fig. 68E) of this species unequivocally indicates relationships with the *Operclipygus fossipygus* group. However, it has a number of unusual characters for that group, including the elongate, subdepressed body form (Fig. 67A), the submarginal pronotal stria showing a postocular angulation, and the lack of basal pygidial foveae (Fig. 67B).

### Operclipygus simplicipygus sp. n.

urn:lsid:zoobank.org:act:190D77AC-0695-4B3E-B72E-89B2712F75C9

http://species-id.net/wiki/Operclipygus_simplicipygus

Figs 67C–D 68F Map 24

#### Type locality.

PERU: Madre de Dios: 15 km NE Puerto Maldonado, Cuzco Amazónico Reserve [12°33'S, 69°03'W].

#### Type material.

**Holotype male**: “PERU: Tambopata Prov. Madre de Dios Dpto. 15km NE Puerto”/ “Maldonado, Reserva Cuzco Amazónico 12°33'S, 69°03'W 200m, #Z2U16”/ “28 June 1989, J. S. Ashe, R. A. Leschen #316 ex. flight intercept trap” / “Caterino/Tishechkin Exosternini Voucher EXO-02212” (SEMC). **Paratype** (1): **PERU: Madre de Dios:** Manu National Park, Cocha Cashu Bio. Stn., 11°53'45"S, 71°24'24"W, 350m, 19.x.2000, fungus covered log, R. Brooks (SEMC).

#### Diagnostic description.

Length: 1.97–2.00 mm, width: 1.68–1.78 mm; body rufopiceous, elongate oval; subdepressed; frons weakly depressed at middle, with fine, sparse ground punctation; frontal stria divergent between eyes, curving mediad, weakly sinuate over antennal bases, fine, complete, and arcuate across front; epistoma shallowly emarginate apically; labrum about twice as wide as long, truncate apically; pronotum lacking prescutellar impression, with fine, rather conspicuous ground punctation, with ~15 coarse lateral punctures; marginal stria broadly interrupted behind head; lateral submarginal stria complete along side, curving inward, meeting or nearly meeting anterior marginal stria at weak postocular angle, anterior marginal stria may be very weakly recurved posterad if free; median pronotal gland openings about half pronotal length behind anterior margin; elytron with two complete epipleural striae, outer subhumeral stria complete, sinuate at middle, inner subhumeral stria present in apical half, dorsal striae 1-3 complete, striae 4-5 present in apical half, sutural stria present in apical three-fourths; elytra with weak strioles between apices of striae 1 and 2; prosternal keel outwardly produced at base, carinal striae complete, rather narrowly separated, united anteriorly and posteriorly; prosternal lobe narrowly rounded, marginal stria complete, well impressed; anterior margin of mesoventrite shallowly emarginate, with complete marginal stria; mesometaventral stria weakly arched forward at middle, reaching basal third of mesoventrite, continued by lateral metaventral stria posterolaterad toward middle of metacoxa; 1^st^ abdominal ventrite with two complete lateral striae; propygidium with moderately dense ground punctation mostly obscured by uniformly dense secondary punctures; pygidium with dense ground punctation with numerous small secondary punctures interspersed; marginal pygidial sulcus deep, complete, not ending in basal foveae. Male genitalia: segments 8-10 very similar to those of *Operclipygus subdepressus* (see Figs 68A–C), except S8 with sides weakly convergent to apex, apical guides undeveloped at base, only slightly widened in apical half to rounded apices; S9 [of only available male broken across stem near apex] with stem broader, with rounded apical emargination; tegmen (Fig. 68F) short and broad, widest about one-third from apex, with extremely large and projecting medioventral tooth; median lobe with wide gonopore, long proximal apodemes, showing weak differentiation; basal piece about one-third tegmen length.

#### Remarks.

Aside from marked differences in body shape (Fig. 67C), this species appears to be closely related to *Operclipygus subdepressus*, sharing a deep but basically simple pygidial sulcus (Fig. 67D), presence of part of the inner subhumeral stria and dense propygidial punctation, as well as some characters of male genitalia, such as the short, broad 8^th^ tergite, possibly correlated with a short, wide aedeagus. In any case, the relative roundness and convexity of the body of *Operclipygus simplicipygus* will separate it easily.

#### Etymology.

This species’ name refers to the lack of basal pygidial foveae, otherwise common in most members of the *Operclipygus fossipygus* group.

### Operclipygus therondi

Wenzel, 1976

http://species-id.net/wiki/Operclipygus_therondi

Fig. 67E Map 24

Operclipygus therondi Wenzel, 1976: 250.

#### Type locality.

ECUADOR: Pichincha:Santo Domingo [0°15'S, 79°10'W].

#### Type material.

**Holotype male**: published type locality: “Ecuador, versant O. des Andes, Santo Domingo, 500m” (IRNSB), not examined. **Paratype** **female**, genitalia missing: “Ecuador:verst.O.desAndes, Santo Domingo 600m., J. et. N. Leleup”/”Paratype *Operclipygus therondi* Wenzel” (FMNH), examined 2006.

#### Other material.

**ECUADOR: Pichincha:** 1: Santo Domingo, Tinalandia, 680m, 4.v–25.vii.1985, malaise/FIT, S. & J. Peck (CHSM); 1: 16km E Santo Domingo, Tinalandia, 750m, 27.iii.1999, R. Brooks (CMNC).

#### Diagnostic description.

Length: 2.15–2.37 mm, width: 1.93–2.06 mm; body rufopiceous, broadly rounded; frons weakly depressed at middle; frontal stria rounded at sides, rather narrowly arcuate across front; supraorbital stria weak, present at middle, detached from frontal stria; epistoma flat to weakly convex; labrum about twice as wide as long, weakly, asymmetrically emarginate apically; pronotal sides rather strongly convergent to front, disk lacking prescutellar impression, with fine, sparse ground punctation, lacking coarser lateral punctures; marginal pronotal stria interrupted behind head; submarginal pronotal stria continuous along lateral and anterior margins; median pronotal gland openings simple, about three-fourths pronotal length behind anterior margin; elytron with two complete epipleural striae, outer subhumeral stria complete, inner subhumeral stria faintly impressed in apical half or absent, dorsal striae 1-3 complete, 4^th^ stria present in apical two-thirds, 5^th^ stria present in apical half, sutural stria present in apical two-thirds; prosternal lobe truncate to very weakly emarginate at base, carinal striae complete, widely separated at base, united in anterior arch; anterior mesoventral margin truncate, marginal stria fine, fragmented at middle; mesometaventral stria very broadly arched forward to near marginal mesoventral stria, which is continued posterolaterally by lateral metaventral stria toward outer third of metacoxa; 1^st^ abdominal ventrite with inner lateral stria complete, outer stria present in basal half only; lacking conspicuous postmetacoxal fovea; propygidium with fine, sparse ground punctation, especially conspicuous in posterolateral corners, with larger, round punctures separated by about one-third their diameters; pygidium with fine, dense ground punctation, without coarser punctures except along basal margin; marginal pygidial sulcus complete, fine, complete, weakly widened toward vague basal foveae. Male not available for study.

#### Remarks.

The unique form of the marginal pygidial sulcus, with a deep marginal groove ending in weakly enlarged basal foveae (Fig. 67E), easily distinguishes this species.

### Operclipygus disconnectus sp. n.

urn:lsid:zoobank.org:act:8DF29A91-B484-4F25-86AB-2D3D2DB299CF

http://species-id.net/wiki/Operclipygus_disconnectus

Figs 67F 68G–K Map 24

#### Type locality.

PERU: Junín:11km NE Puerto Ocopa, Los Olivos [11°7.0'S, 74°15.52'W].

#### Type material.

**Holotype male**: “**PERU: Depto. Junín,** 11km NE Puerto Ocopa, Los Olivos. 11°7.00'S, 74°15.52'W, 1200m. Window traps 31.iii.–1.iv.2009. A.V. Petrov”/ “Caterino/Tishechkin Exosternini Voucher EXO-00401” (FMNH). **Paratypes** (2): **PERU: Junín:** 1: Pampa Hermosa Lodge, 22km N San Ramon, 10°59.3'S, 75°25.5'W, 1220m, 24–27.xi.2007, FIT, D. Brzoska (SEMC); 1: Villa Rica Rd., 10°47'6"S, 75°18'54"W, 1475m, 15–18.x.1999, FIT, R. Brooks & D. Brzoska (SEMC).

#### Diagnostic description.

Length: 2.12–2.31 mm, width: 1.90–2.12 mm; body piceous, broadly rounded; frons weakly depressed; frontal stria with sides rounded, interrupted over antennal bases, arcuate across front; supraorbital stria fine, rounded dorsad, detached from sides of frontal stria; epistoma moderately convex; labrum about twice as wide as long, asymmetrically emarginate apically, projecting beneath; left mandible untoothed, right mandible with small, subacute basal tooth; pronotum lacking prescutellar impression, with fine, sparse ground punctation, with 2-5 coarse lateral punctures; anterior margin of pronotum not projecting at middle; marginal pronotal stria generally broadly interrupted behind head; submarginal pronotal stria continuous, complete along lateral and anterior margins, marginal bead weakly convex; median pronotal gland openings about two-thirds pronotal length behind anterior margin; elytra with two complete epipleural striae, outer subhumeral stria complete, inner subhumeral stria fine, present in apical half, dorsal striae 1-3 complete, 4^th^ stria present in apical half, 5^th^ stria present in apical third, sutural stria present in apical three-fourths; prosternal keel truncate to weakly rounded at base, carinal striae complete, convergent to anterior arch, microsculptured between; anterior metaventral margin straight, marginal stria complete; mesometaventral stria rather narrowly arched forward at middle, reaching to mesoventral midpoint, sinuate laterally, continued obliquely posterolaterad toward outer corner of metacoxa, slightly abbreviated at apex; 1^st^ abdominal ventrite with complete inner lateral stria, abbreviated outer stria; inconspicuous fovea present near inner corner of metacoxa; propygidium with fine, moderately sparse ground punctation and irregularly oval, shallow punctures over most of disk, separated by about one-third their diameters; pygidium with fine, dense ground punctation, lacking coarser punctures; marginal pygidial sulcus absent, but basolateral corners of pygidium with rather small, round foveae. Male genitalia (Figs 68G–K): accessory sclerites present, large; T8 rather narrow, slightly convergent apically, apical emargination rather narrow, but with larger, secondary, parallel desclerotized area surrounding it, basal emargination evenly arcuate, basal membrane attachment line distad basal emargination by about one-fourth its depth, ventrolateral apodemes most strongly developed near base, narrowed to apex, not meeting at midline; S8 with sides weakly rounded, subparallel, with apical guides narrow, widening weakly to apex; T9 with apices weakly convergent, slightly enlarged and apically truncate; T10 with halves fused along basal third of midline; S9 broad apically, sclerotized along edges, evenly narrowed to narrowly rounded base, with shallow apical emargination, apical flanges separate; tegmen widest one-third from apex, narrowed to base and apex, with strong, narrow medioventral process projecting beneath; median lobe large, three-fourths as long as tegmen, with wide gonopore, proximal apodemes differentiated into short thick portion, with proximal arms prominent, sinuate; basal piece short, about one-third tegmen length.

#### Remarks.

This species is unique in the presence of basolateral pygidial foveae that are not connected by any indication of a marginal sulcus (Fig. 67F).

#### Etymology.

This species’ name refers to the lack of marginal connection between the pygidial foveae.

### Operclipygus olivensis sp. n.

urn:lsid:zoobank.org:act:4B45FBA9-BA50-495D-BCB8-2EF103C0D918

http://species-id.net/wiki/Operclipygus_olivensis

Figs 67G–H Map 24

#### Type locality.

PERU: Junín:11km NE Puerto Ocopa, Los Olivos [11°7.0'S, 74°15.52'W].

#### Type material.

**Holotype female**: “**PERU: Junín,** 11km NE Puerto Ocopa, Los Olivos. 1,200m 11°3.00'S, 74°15.52'W, Flight intercepts 26–27.iii.2009. A.K. Tishechkin. AT 1081”/ “Caterino DNA Voucher, Extraction: MSC-2151, Species: *Opercl*. ~KU2010.5.3, Extraction Date: viii.10.2011” / “Caterino/Tishechkin Exosternini Voucher EXO-00677” (FMNH).

#### Diagnostic description.

Length: 2.40 mm, width: 2.09 mm; body piceous, broadly rounded; frons shallowly depressed at middle, with fine, sparse ground punctation; frontal stria with sides divergent, rounded, narrowly interrupted over antennal bases, arcuate across front; supraorbital stria fine, rounded dorsad, detached from sides of frontal stria; epistoma moderately convex; labrum about twice as wide as long, asymmetrically emarginate apically; left mandible untoothed, right with small, subacute basal tooth; pronotum lacking prescutellar impression, with fine, sparse ground punctation, with a few faint, shallow lateral pronotal punctures; anterior margin of pronotum not projecting at middle; marginal pronotal stria broadly interrupted behind head; submarginal pronotal stria continuous, complete along lateral and anterior margins, marginal bead convex; median pronotal gland openings just over half pronotal length behind anterior margin; elytra with two complete epipleural striae, outer subhumeral stria complete, inner subhumeral stria fine, present in apical two-thirds, fragmented basally, striae 1-3 complete, 4^th^ and 5^th^ striae similar in length, present in about apical third, sutural stria present in apical two-thirds; prosternal keel very weakly projecting at base, carinal striae complete, broadly separated at base, meeting in anterior arch; anterior metaventral margin very weakly emarginate, marginal stria narrowly interrupted; mesometaventral stria broadly arched forward nearly to marginal mesoventral stria, slightly sinuate laterally, continued obliquely posterolaterad toward middle of metacoxa, more or less complete; 1^st^ abdominal ventrite with two abbreviated lateral striae; inconspicuous fovea present near inner corner of metacoxa; propygidium with fine, inconspicuous ground punctation and irregularly round, shallow punctures over most of disk, separated by about one-third their diameters; pygidium with very fine but dense ground punctation, with few slightly coarser punctures in basal third; completely lacking marginal pygidial sulcus or foveae. Male not known.

#### Remarks.

While numerous external characters suggest that this species belongs in the *Operclipygus fossipygus* group, it lacks any indication of a marginal pygidial sulcus (Fig. 67H). It is unfortunate that no male is known, as the distinctive aedeagus of the group would help to solidify its placement here. However, phylogenetic analyses underway, including DNA sequences for this species, do support a close relationships to other members of the *fossipygus* group.

#### Etymology.

This species is named for the village of Los Olivos, its type locality.

### *Operclipygus impunctipennis* group

**Figure 69.**
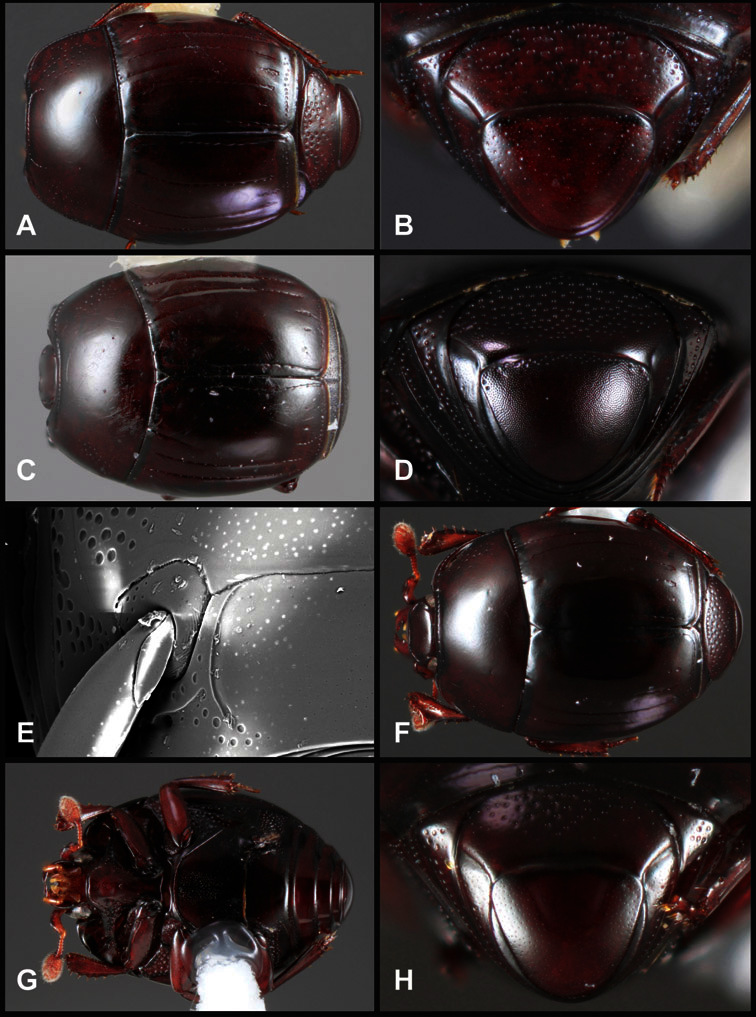
*Operclipygus impunctipennis* group. **A** Dorsal habitus of *Operclipygus impunctipennis* **B** Pygidia of *Operclipygus impunctipennis* **C** Dorsal habitus of *Operclipygus punctissipygus* **D** Pygidia of *Operclipygus punctissipygus* **E** 1^st^ abdominal ventrite of *Operclipygus punctissipygus* **F** Dorsal habitus of *Operclipygus granulipectus* **G** Ventral habitus of *Operclipygus granulipectus* **H** Pygidia of *Operclipygus granulipectus*.

This large group of species is difficult to characterize, and is probably closely related to the *Operclipygus hamistrius* group, having generally quite similar male genitalia. Externally the species of the *Operclipygus impunctipennis* group generally lack the microsculpture of the *Operclipygus hamistrius* group, have very reduced, fine, abbreviated, or absent marginal pygidial sulcus, frequently have dense pygidial ground punctation, and usually have the anterior submarginal pronotal stria continuous with the lateral stria, not broken as is generally the case in the *Operclipygus hamistrius* group. Many of the species have a series of apical marginal elytral punctures (e.g., Figs 69A, C), and in some these are coalesced into a distinct marginal stria (e.g., Fig. 5H). The elytral striation is often strongly reduced, with many species having no more than two or three complete striae.

**Figure 70.**
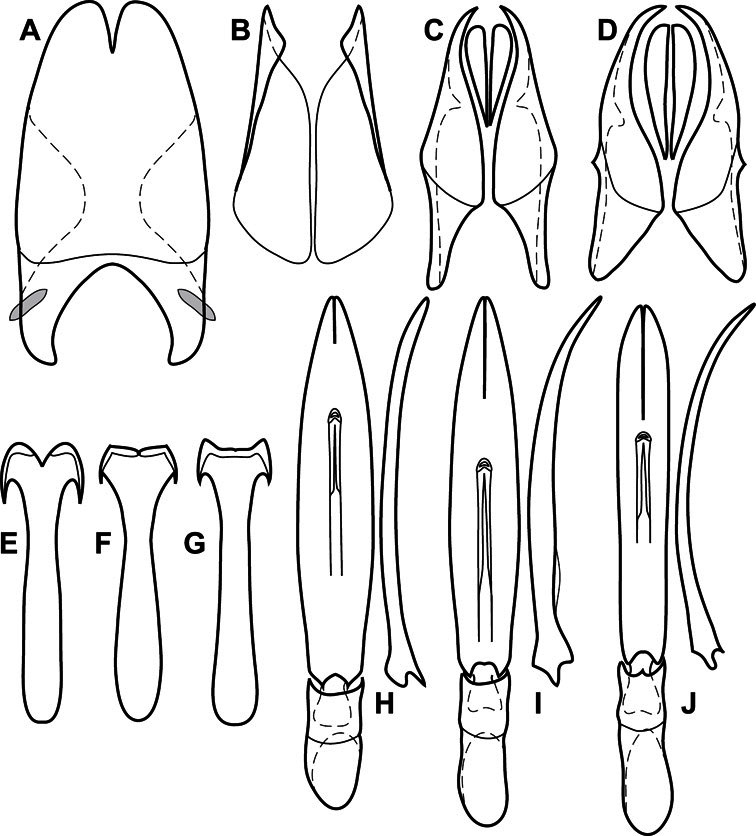
Male genitalia of *Operclipygus impunctipennis* group. **A** T8 of *Operclipygus impunctipennis* **B** S8 of *Operclipygus impunctipennis* **C** T9 & T10 of *Operclipygus impunctipennis* **D** T9 & T10 of *Operclipygus punctissipygus* **E** S9 of *Operclipygus impunctipennis* **F** S9 of *Operclipygus punctissipygus* **G** S9 of *Operclipygus granulipectus* **H** Aedeagus, dorsal and lateral views, of *Operclipygus impunctipennis* **I** Aedeagus, dorsal and lateral views, of *Operclipygus punctissipygus* **J** Aedeagus, dorsal and lateral views, of *Operclipygus granulipectus*.

Male genitalia in the group are somewhat varied, with two moderately well-defined subgroups. One of these (e.g., Figs 70A–J; species most closely related to *Operclipygus impunctipennis*) possesses accessory sclerites, has the medioventral process of the aedeagus weak or absent, and has the basolateral corners of the head of the 9^th^ sternite recurved, or ‘hooked’. The other subgroup (those below starting with *Operclipygus foveiventris*; see Figs 75A–H) lacks accessory sclerites, has a strong medioventral process close to the base of the aedeagus, has the tegmen evenly tapered over much of its length, and has a very thin, elongate median lobe. However, not all of the species can easily be assigned to one of these groups.

### Key to the species of the *Operclipygus impunctipennis* group

**Table:** 

1	Elytron with four complete elytral striae (stria 1 may be abbreviated apically, but striae 2-4 are complete)	2
–	Elytron with fewer than four complete dorsal striae	4
2	Pygidium with dense ground punctation and coarse secondary punctation (Fig. 76B); outer subhumeral stria present in apical half	*Operclipygus maesi* sp. n.
–	Ground punctation of pygidium fine and sparse; secondary punctation varied; outer subhumeral stria absent	3
3	1^st^ dorsal elytral stria at least weakened, usually obsolete in apical half; lateral submarginal pronotal stria obsolete basally	*Operclipygus nicodemus* sp. n.
–	1^st^ dorsal stria complete to apex; lateral submarginal pronotal stria usually complete, may be interrupted at middle	*Operclipygus chamelensis* sp. n.
4	Pygidium with dense ground punctation, secondary punctation varied; marginal pygidial sulcus absent	5
–	Ground punctation of pygidium sparse; secondary punctation varied; marginal pygidial sulcus fine, often fragmented, usually present	11
5	Pygidium completely lacking secondary punctures (except possibly along extreme basal margin), with fine ground punctation only	6
–	Pygidium with secondary punctures, though they may be small and sparse	8
6	Central portion of frontal stria absent; lateral submarginal pronotal stria generally more or less complete along side (Fig. 76C); lateral pronotal punctures few, faint; 3^rd^ dorsal stria interrupted	*Operclipygus pacificus* sp. n.
–	Central portion of frontal stria present as a detached fragment; lateral submarginal pronotal stria at least slightly abbreviated at base; other characters varied	7
7	Outer lateral stria of 1^st^ abdominal ventrite extending behind metacoxa, passing through a distinct fovea behind the posterolateral coxal corner (Fig. 69E); apical margin of elytron with series of disconnected punctures (Fig. 69C); 3^rd^ dorsal stria interrupted	*Operclipygus punctissipygus* sp. n.
–	Outer lateral stria of 1^st^ abdominal ventrite not extending laterad behind metacoxa; postmetacoxal fovea situated behind inner corner of coxa, very small and inconspicuous; apical margin of elytron lacking punctures or marginal stria (Fig. 78C); 3^rd^ dorsal stria usually complete	*Operclipygus vorax* sp. n.
8	1^st^ abdominal ventrite with large fovea behind inner corner of metacoxa, with the lateral stria passing through it, bending behind coxa (Fig. 73F); basal fragment of 4^th^ dorsal elytral stria generally present (Fig. 73E); 3^rd^, 4^th^ and 5^th^ stria represented by very short apical fragments; apical margin of elytron with crenulate stria (Fig. 73E)	*Operclipygus foveiventris* sp. n.
–	1^st^ abdominal ventrite with small to inconspicuous fovea behind inner corner of metacoxa; other characters varied, but basal fragment of 4^th^ stria and apical fragment of 5^th^ stria often absent	9
9	Coarse punctures of propygidium dense, covering entire disk (Fig. 73G); secondary punctures of pygidium rather coarse; lateral pronotal punctures numerous, coarse (Fig. 73G); 3^rd^ dorsal elytral stria frequently complete; basal fragment of 4^th^ dorsal stria present	*Operclipygus marginipennis* sp. n.
–	Coarse punctures of propygidium markedly diminished in posterolateral corners, where dense ground punctation becomes conspicuous; secondary punctures of pygidium rather fine; 3^rd^ dorsal elytral stria interrupted; basal fragment of 4^th^ stria present or absent	10
10	Outer subhumeral stria generally represented by apical fragment; body often with faint metallic green tinge (Fig. 78A); apical margin of elytron with well impressed stria; basal fragment of 4^th^ dorsal elytral stria usually absent	*Operclipygus subviridis* sp. n.
–	Outer subhumeral stria absent; body lacking any metallic tinge, piceous; other characters varied, but 4^th^ stria usually represented by basal fragment	*Operclipygus nitidus* sp. n.
11	Anterior submarginal pronotal stria detached from lateral stria, recurved posterad	12
–	Submarginal pronotal stria continuous across anterior margin	13
12	Elytron with 4^th^ dorsal stria more or less complete (may be weakened or narrowly interrupted at middle) and 3^rd^ dorsal stria broadly interrupted (Fig. 69A)	*Operclipygus impunctipennis* (Hinton)
–	Elytron with 3^rd^ dorsal stria complete, 4^th^ variably complete to interrupted (Fig. 71E)	*Operclipygus tripartitus* sp. n.
13	4^th^ dorsal elytral stria well impressed in about basal fourth	14
–	4^th^ stria absent from basal half of elytron or at most represented by very small basal puncture (may be small apical fragment regardless)	15
14	Basal fragment of 4^th^ dorsal stria more strongly impressed than fine, scratch-like 3^rd^, both interrupted at middle (Fig. 71F); 1^st^ abdominal ventrite with large fovea posterolaterad metacoxa (see Fig. 73A)	*Operclipygus latifoveatus* sp. n.
–	3^rd^ dorsal stria usually complete and always as or more strongly impressed than 4^th^ (Fig. 73D); 1^st^ abdominal ventrite with very small fovea behind inner corner of metacoxa	*Operclipygus mangiferus* sp. n.
15	Elytron with distinct apical marginal stria	16
–	Elytron lacking a distinct apical marginal stria; 1^st^ abdominal ventrite with large fovea behind outer corner of metacoxa (Fig. 73A)	*Operclipygus lissipygus* sp. n.
16	Male metaventrite shallowly depressed and setose (Fig. 69G); pygidium lacking secondary punctures, with fine sparse ground punctation only (Fig. 69H); 1^st^ abdominal ventrite with inconspicuous fovea behind inner corner of metacoxa	*Operclipygus granulipectus* sp. n.
–	Male metaventrite not depressed or setose, identical to that of female; pygidium with few secondary punctures; 1^st^ abdominal ventrite with large fovea behind outer corner of metacoxa (Fig. 71B)	*Operclipygus pauperculus* sp. n.

### Operclipygus impunctipennis

(Hinton, 1935) comb. n.

http://species-id.net/wiki/Operclipygus_impunctipennis

**Map 25.**
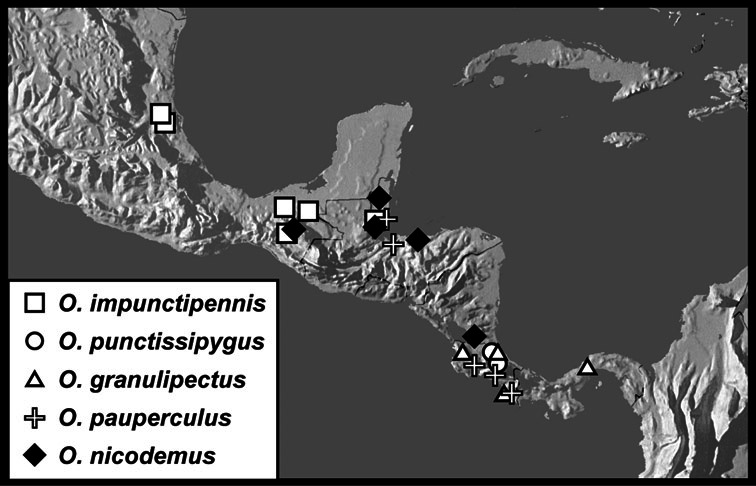
Records of the *Operclipygus impunctipennis* group.

Figs 69A–B 70A–C, E, H Map 25

Phelister impunctipennis Hinton, 1935b: 58.

#### Type locality.

MEXICO: Tabasco: Teapa [17°33'N, 92°57'W].

#### Type material.

**Holotype**: “Teapa, Tabasco, Feb. H.H. Smith” / “*Phelister impunctipennis* Hntn.” (BMNH), examined 2010.

#### Other material.

**BELIZE: Cayo:** 1: San Pastor, 27.x.1994, FIT (BMNH). **MEXICO: Chiapas**: 1: “Encinar 16 de septiembre” Carretera Tuxtla Gtz.-Chicoasen Loc. Cn., 16°48'60"N, 93°10'43"W, 851m, 22.iii.2005, cow dung (ECOSUR), 1: 17.iii.2005, cow dung (ECOSUR), 1: 26.iii.2005, cow dung (ECOSUR), 2: 25.v.2005, dead rat bait (ECOSUR); 1: Palenque, 100m, 2–30.vii.1983, FIT, S. & J. Peck & R. Anderson (CHSM); 1: 8km S Chicoasen, 690m, 1.vi.1991, sifted from leaf litter, J. Ashe (CHSM);' 1: Salto de Agua, 8km SE, 17.51592°N, 92.30231°W, 100m, 14.vi.2008, sifted leaf litter, secondary wet forest (SEMC); 4: Parque Nac. Sumidero, 1000m, 25.v.1990, FIT, H. & A. Howden (AKTC, BDGC), 1: 13.vi.1990, FIT, H. & A. Howden (BDGC), 1: 1–14.vi.1990, FIT, H. & A. Howden (BDGC); 3: **San Luis Potosí**, 13km E Xilitla, 335m, 4.vi.1987, berl. trop. for litter, R. Anderson (SEMC); 1: 50mi. NNW Cd. Valles El Salto, 6.vii.1969, tropical decid. forest leaf litter, S. & J. Peck (FMNH).

#### Diagnostic description.

Length: 1.72–1.87 mm, width: 1.47–1.59 mm; body rufopiceous, elongate oval, smooth, shining; frons weakly depressed at middle, with sparse but fairly conspicuous ground punctation; frontal stria weakly rounded between eyes, complete across front; supraorbital stria absent; epistoma mostly convex; labrum about twice as wide as long, apically emarginate on dorsal part of edge, but with apical process beneath, just left of midline; left mandible lacking basal tooth, right mandible with small subacute tooth; pronotal disk with prescutellar impression lacking or extremely fine, punctiform, ground punctation very fine and inconspicuous, with ~10 coarse lateral punctures; marginal pronotal stria complete behind head and along lateral margins; lateral submarginal stria complete to variably basally abbreviated along side, arched inward anteriorly, nearly meeting anterior portion, which is recurved posterad about one-fifth pronotal length; median pronotal gland openings situated behind recurved ends of anterior stria, about two-thirds pronotal length from anterior margin; elytron with two complete epipleural striae, outer subhumeral stria very short and apical, inner subhumeral absent, striae 1-2 complete, 3^rd^ stria very finely impressed in basal third (rarely reaching the anterior elytral margin), and as a very short apical stria, 4^th^ stria complete, 5^th^ stria present as basal rudiment or absent, sutural stria present in apical two-thirds; elytral disk with apical row of conspicuous punctures; prosternal lobe weakly emarginate basally, carinal striae complete, widely separate at base, convergent at middle, weakly divergent to apical arch; prosternal lobe with complete marginal stria; mesoventrite weakly projecting at middle, marginal stria barely interrupted; mesometaventral stria evenly and strongly arched forward to near anterior mesoventral margin; lateral metaventral stria extending to inner third of metacoxa; metaventral disk convex; 1^st^ abdominal ventrite with single lateral stria present, longitudinal in basal half, interrupted near inner corner of metacoxa, continuing directly laterad behind coxa, passing through a small but distinct postcoxal fovea; propygidium with ground punctation fine, with moderately large punctures separated by slightly less than their diameters in basal half, frequently coalesced into short strioles in anterolateral corners, becoming smaller and sparser toward apex, more or less absent from apical third; pygidium with ground punctation fine, sparse, slightly coarser punctures often interspersed, separated by 4–5× their diameters; marginal pygidial stria very fine, present only apically, occasionally absent. Male genitalia (Figs 70A–C, E, H): accessory sclerites present; T8 elongate, sides subparallel in basal half, angulate to narrow apex, basal emargination broad and deep, nearly reaching basal membrane attachment line; S8 with sides convergent to apex, with apical guides narrow along most of margin, expanded near acuminate apex, inner edges approximate near base, weakly divergent to near apex; T9 with sides subparallel in basal half, convergent toward narrow, subacute apex; T10 with halves separate; S9 narrowest near apex, with base of stem slightly wider, subparallel to rounded base, apex narrowly emarginate, rounded, with strongly produced basolateral corners, apical flange divided; tegmen rather flat, with sides evenly rounded from base to apex, curving ventrad, medioventral process not at all evident; basal piece about one-third tegmen length; median lobe just over half tegmen length, proximal apodemes differentiated into similar-length thick and filamentous portions.

#### Remarks.

The pronotal striae (Fig. 69A) would tend to place this species in the *Operclipygus hamistrius* group. However, the darker coloration, lack of microsculpture on the integument, pattern of elytral striation, and the very distinct male genitalia exclude it from that group. Among related species, *Operclipygus impunctipennis* may be distinguished by the pronotal striae, in part. Most other species in this group have the submarginal stria unbroken across the front. This, together with the unusual pattern of elytral striae, the third being broadly interrupted but with the fourth complete, will distinguish it from nearly any other *Operclipygus*.

### Operclipygus punctissipygus sp. n.

urn:lsid:zoobank.org:act:A367CFBF-6014-470D-B780-42D21A8886D8

http://species-id.net/wiki/Operclipygus_punctissipygus

Figs 69C–E 70D, F, I Map 25

#### Type locality.

COSTA RICA: Heredia: La Selva Biological Station [10°26'N, 84°1'W].

#### Type material.

**Holotype male**: “COSTA RICA: Heredia, La Selva, 3.2 km SE Puerto Viejo, 100m, 17 Feb. 1992, W.Bell, ex: flight intercept trap”/ “SEMC0903633 KUNHM-ENT” (INBI). **Paratypes** (10): 6: same data as type, except as noted: 1: 9.ii.1992, FIT, W. Bell (SEMC), 2: 21.ii.1992 (SEMC, FMNH), 2: 1.iii.1992 (SEMC, MSCC), 1: 17.iii.1992 (SEMC), 1: 28.vi.1998, FIT, C.E. Carlton & A.K. Tishechkin (LSAM); **Cartago:** 1: Parque Nac. Barbilla, R.F. Rio Pacuare, Turrialba, Send. Rio Danto, 500–600m, 28.viii–5.ix.2001, FIT, W. Arana (INBIO); **Heredia:** 1: Parque Nac. Braulio Carrillo, Est. Magsasay, 200m, i.1991, M. Barrelier, (INBIO); **Limón:** 1: Sector Cedrales de la Rita, 3km N. del Puente Rio Suerte, Ruta Puerto Lindo, 10m, viii.1996, FIT, E. Rojas, (INBIO).

#### Diagnostic description.

Length: 1.93–2.22 mm, width: 1.65–1.93 mm; body rufopiceous, slightly elongate oval, sides evenly rounded; frons depressed at middle, with fine, sparse ground punctation; sides of frontal stria divergent between eyes, interrupted over antennal bases, present as isolated arc in front; supraorbital stria absent; epistoma strongly convex; labrum narrow, about twice as wide as long, weakly emarginate with small process beneath, left of midline; pronotum lacking prescutellar impression, with ground punctation fine and inconspicuous, with ~10 coarser punctures at sides; marginal pronotal stria absent behind head, complete at sides; submarginal pronotal stria complete across front, extending along anterior half of lateral margin, obsolete in basal half; median pronotal gland openings about two-thirds pronotal length from anterior margin; elytron with two complete epipleural striae, outer subhumeral stria very short and apical, inner subhumeral absent, striae 1-2 complete, 3^rd^ stria very finely impressed in basal third, reaching the anterior elytral margin, and as a very short apical stria, 4^th^ stria represented by very short apical fragment, 5^th^ stria absent, sutural stria present in apical two-thirds; elytral disk with apical row of conspicuous punctures; prosternal keel very weakly emarginate at base, carinal striae converging, united anteriorly about one-fourth before presternal suture, weak secondary striae present between procoxa and primary carinal stria; prosternal lobe rather short, marginal stria abbreviated at sides; mesoventrite not projecting at middle, marginal stria broadly interrupted by mesometaventral stria which is arched forward along most of the mesoventral margin; lateral metaventral stria strongly abbreviated posteriorly, reaching only about middle of metaventral disk; 1^st^ abdominal ventrite with single lateral stria present, longitudinal in basal half, interrupted near inner corner of metacoxa, continuing directly laterad behind coxa, passing through a small but distinct postcoxal fovea behind outer corner of coxa; propygidium and especially pygidium with ground punctation dense; propygidium with small, round punctures separated by about their diameters, becoming sparser apicolaterally; pygidium with only a few coarser punctures along basal margin; marginal pygidial stria absent. Male genitalia (Figs 70D, F, I): very similar to those of *Operclipygus impunctipennis*, differing as follows: T8 with basal emargination shallower, only reaching halfway to basal membrane attachment line; S8 with sides subparallel in basal half, weakly divergent to apices; T9 with acute lateral denticles near middle; S9 with sides more rounded to base, apex subtruncate, with very small median emargination, basolateral corners not as prominently produced; tegmen very similar to that of *Operclipygus impunctipennis*, but widest point slightly basad middle.

#### Remarks.

This species can be distinguished by its broad body outline (Fig. 69C), depressed frons with stria interrupted at the sides, submarginal pronotal stria which is continuous across the front but ending midway along the lateral margins, presence of only two complete elytral striae, transverse postmetacoxal stria with small fovea (Fig. 69E), and presence of dense ground punctation on the propygidium and pygidium (Fig. 69D).

#### Etymology.

This species’ name refers to the fine, dense punctures of the pygidium.

### Operclipygus granulipectus sp. n.

urn:lsid:zoobank.org:act:401118C8-A041-4B39-B6D7-A9DC93C3CA49

http://species-id.net/wiki/Operclipygus_granulipectus

Figs 69F–H 70G, J Map 25

#### Type locality.

COSTA RICA: Heredia: La Selva Biological Station [10°26'N, 84°1'W].

#### Type material.

**Holotype male**: “COSTA RICA: Heredia, La Selva, 3.2 km SE Puerto Viejo, 100m, 28 Feb. 1992, W.Bell, ex: flight intercept trap”/ “SEMC0903634 KUNHM-ENT” (INBIO). **Paratypes** (28): 26: same data as type, except as noted: 2: same data as type (INBIO), 1: 14.ii.1992 (SEMC), 2: 17.ii.1992 (SEMC, FMNH), 1: 19.ii.1992 (SEMC), 2: 1.iii.1992 (SEMC, MSCC), 3: 3.iii.1992 (SEMC, AKTC), 2: 6.iii.1992 (SEMC), 1: 19.vi.1998 (LSAM), 1: 22.vi.1998, FIT, C.E. Carlton & A.K. Tishechkin (LSAM), 1: 24.vi.1998 (LSAM), 1: 25.vi.1998 (LSAM), 1: 26.vi.1998 (LSAM), 2: 27.vi.1998 (LSAM), 2: 28.vi.1998 (LSAM), 1: 29.vi.1998 (LSAM), 2: 170m, 2–5.iii.1991, FIT, H. & A. Howden (CMNC), 2: 21–28.iii.1988, W.E. Steiner, J.M. Hill, J.M. Swearingen, J.M. Mitchell (USNM).

#### Other material

(13). **COSTA RICA: Guanacaste:** 1: Parque Nac. Guanacaste, 9km S Santa Cecilia, Est. Pitilla, 700m, vii.1995, FIT, C. Moraga, P. Rios, (INBIO); **Limón:** 2: Area Cons. Tortuguero, Sector Cerro Cocori, Fca. De E. Rojas, 150m, v.1993, E. Rojas, (INBIO), 2: 28.v–17.vi.1992, E. Rojas, (INBIO); 1: Oeste de R.B. Hitoy Cerere, Finca el Tucan, 100-200m, 14–21.viii.2001, FIT, W. Arana (INBIO); 1: R.B. Hitoy Cerere, Send. Espavel, 140m, 18.iii–1.iv.2002, FIT, W. Arana (INBIO); 2: Estrella Valley, Pandora, 1–16.iii.1984, FIT, H. Howden & G. Manley (CHSM); **Puntarenas:** 3: Est. Esquinas, Peninsula de Osa, 200m, iv.1993, J.F. Quesada, (INBIO); 2: Bosque Esquinas, Peninsula de Osa, 200m, iv.1993, J.F. Quesada, (INBIO); 2: Alajuela, San Jose, *Eciton burchelli*, H. Schmidt (BMNH). **PANAMA: Panamá**: 1: Barro Colorado Isl., 9°11'N, 79°51'W, 1.viii.1994, FIT, D. Banks (SEMC).

#### Diagnostic description.

This species is very similar to the preceding, *Operclipygus punctissipygus*, differing only in the following characters: length: 1.62–1.93 mm, width: 1.37–1.68 mm; frontal stria complete over antennae; supraorbital stria frequently represented by a few fragments; elytron with outer subhumeral stria absent, stria 1 variably abbreviated from apex, sutural stria longer, present in apical three-fourths, apically continuous with apical marginal elytral stria which extends laterad to near apex of 2^nd^ stria, sometimes connected with it; prosternal keel with carinal striae complete, not shortened anteriorly; not only mesometaventral stria, but actual mesometaventral suture arched strongly forward; anteromedial portion of male metaventral disk shallowly depressed, densely punctate, with extremely fine, scale-like setae in punctures; lateral stria of 1^st^ abdominal ventrite bending laterad behind coxa, but generally fragmented; lacking distinct postcoxal fovea; propygidium and pygidium with ground punctation fine and rather sparse; propygidium with coarser punctures small, sparse, restricted to basal half; pygidium lacking coarse punctures; marginal pygidial stria fine, present only at extreme apex. Male genitalia (Fig. 70G, J): T9 lacking distinct lateral denticle; S9 with apical margin lacking median emargination, apical flange continuous, with basolateral corners strongly prolonged proximad; tegmen narrow, elongate, lacking medioventral process; basal piece nearly one-half tegmen length; else as in *Operclipygus impunctipennis*.

#### Remarks.

This species’ most distinctive character is the depressed and setose male meso- and metaventrites (Fig. 69G). It is otherwise rather variable, and hard to characterize. The well-impressed apical marginal elytral stria (Fig. 69F), usual presence of only a single complete dorsal elytral stria (the 2^nd^, with the 1^st^ abbreviated from the apex and the 3^rd^ interrupted), and the lack of coarse punctures on the pygidium (Fig. 69H) should distinguish most specimens. Due to considerable variation, we limit the type series to those specimens from the vicinity of La Selva, in Heredia, Costa Rica.

#### Etymology.

This species’ name refers to the very finely setose meso- and metaventrites of the male.

### Operclipygus pauperculus sp. n.

urn:lsid:zoobank.org:act:1E545708-BD4E-4A64-9718-29508181DD6F

http://species-id.net/wiki/Operclipygus_pauperculus

**Figure 71.**
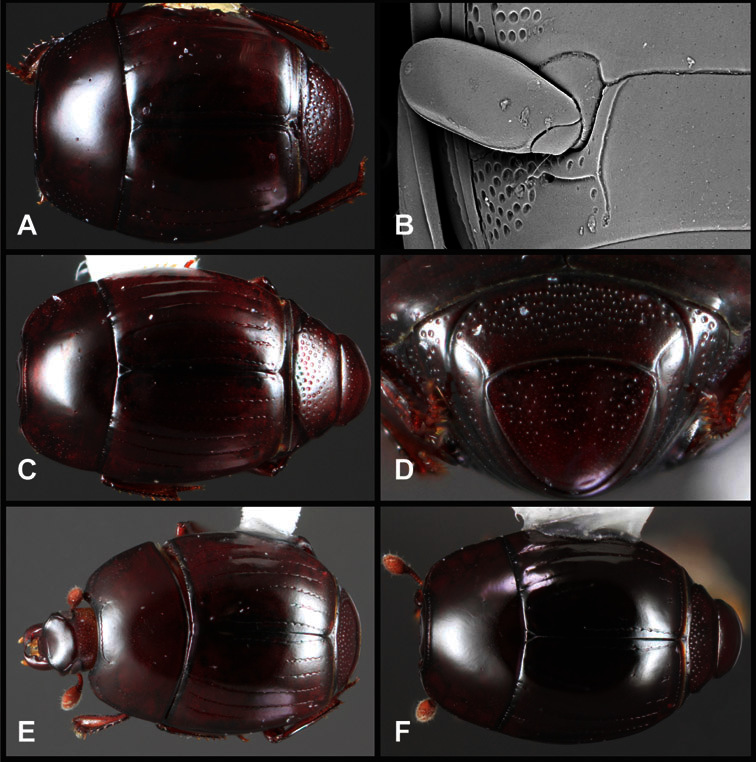
*Operclipygus impunctipennis* group. **A** Dorsal habitus of *Operclipygus pauperculus* **B** 1^st^ abdominal ventrite of *Operclipygus pauperculus* **C** Dorsal habitus of *Operclipygus nicodemus* **D** Pygidia of *Operclipygus nicodemus* **E** Dorsal habitus of *Operclipygus tripartitus* **F** Dorsal habitus of *Operclipygus latifoveatus*.

Figs 71A–B 72A–C, H Map 25

#### Type locality.

COSTA RICA: Puntarenas: Quebrada Bonita Station [9°50'N, 85°0'W].

#### Type material.

**Holotype male**: “Est. Quebrada Bonita, R.B. Carara, Prov. Punta., COSTA RICA, 50m. Ago 1994, R.M. Guzmán, L N 194500_469850 #3163”/ “INBIO CRI002037795” (INBIO). **Paratypes** (7): 1: same data as type; 5: same data as type, except as noted: 2: 200m, iv.1990, R. Zuniga, (INBIO, AKTC), 1: iv.1994, R. Guzman (INBIO), 2: 50m, ix.1994, J.C. Saborio, (INBIO, FMNH), 1: 50m, 1–29.vii.1992, R. Guzman, (MSCC).

#### Other material.

**COSTA RICA: Puntarenas:** 1: Rincon, 7.viii.1967, dead Smooth-billed Ani, J.R. Holman (FMNH); 1: Parque Nac. Manuel Antonio, Quepos, 80m, vii.1991, R. Zuniga, (INBIO); **Alajuela**: 1: San Jose, F Nevermann, La Caja B., Schmidt (FMNH); **Heredia:** 1: La Selva Biol Sta., 10.4306°N, 84.0064°W, 10.vi.2012, FIT, OTS Beetle Course, DNA Extract MSC-2308 (LSAM). **BELIZE: Stann Creek**: 1: Cockscomb Basin Wildlife Sanctuary, 16°46.830'N, 88°27.543'W, 80m, 30–31.v.2008, B. Ratcliffe, R. Cave, M. Jameson, J. Orozco (UNL). **HONDURAS: Cortés:** 1: Lago Yojoa, 650m, 23–28.viii.1994, FIT, tropical, evergreen forest, S. & J. Peck (CMNC); 2: Yojoa Lake, Deer Island, 14°55'N, 87°58'W, 670m, 19–21.vi.1994, FIT, J. Ashe, R. Brooks (SEMC), 4: 22–26.vi.1994, FIT, J. Ashe, R. Brooks (SEMC).

#### Diagnostic description.

**Figure 72.**
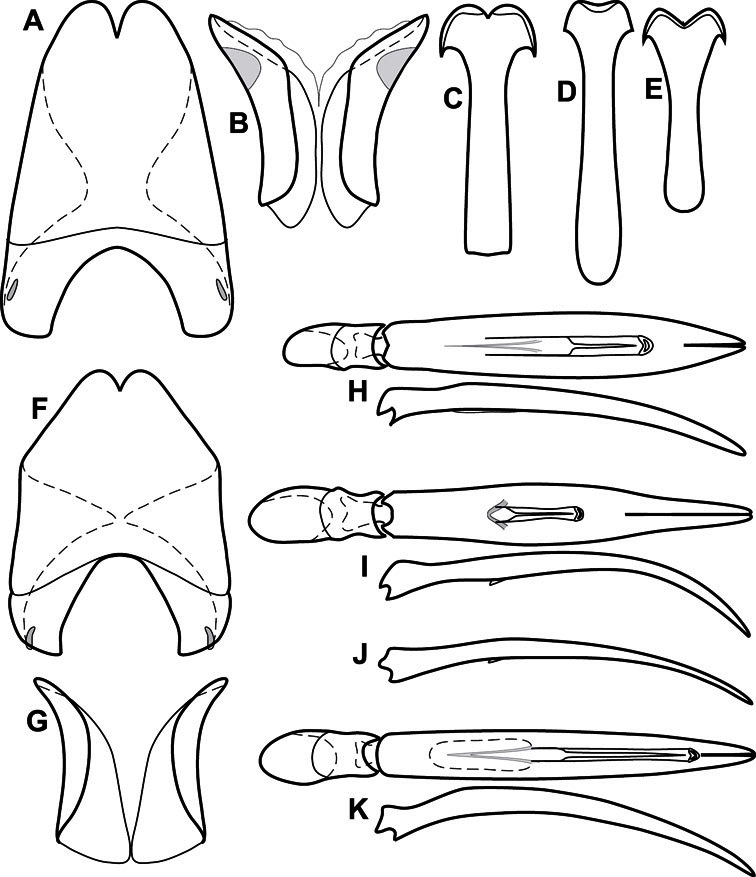
Male genitalia of *Operclipygus impunctipennis* group. **A** T8 of *Operclipygus pauperculus* **B** S8 of *Operclipygus pauperculus* **C **S9 of *Operclipygus pauperculus* **D** S9 of *Operclipygus nicodemus* **E** S9 of *Operclipygus latifoveatus* **F** T8 of *Operclipygus latifoveatus* **G** S8 of *Operclipygus latifoveatus* **H** Aedeagus, dorsal and lateral views, of *Operclipygus pauperculus* **I** Aedeagus, dorsal and lateral views, of *Operclipygus nicodemus* **J** Aedeagus, lateral view, of *Operclipygus tripartitus* **K** Aedeagus, dorsal and lateral views, of *Operclipygus latifoveatus*.

This species is extremely similar to both the preceding two, differing principally in the following characters: length: 1.65–1.67 mm, width: 1.40–1.44 mm; frontal stria usually complete, but may be interrupted over antennal bases; submarginal pronotal stria continuous across front, complete along lateral margin; pronotal disk with few, ~8 coarse lateral punctures; elytron with outer subhumeral stria absent, 1^st^ dorsal stria slightly abbreviated from base, 2^nd^ stria complete, 3^rd^ stria with fine basal scratch and apical appendix, 4^th^ stria usually absent, may be represented by short apical fragment, 5^th^ stria absent, sutural stria present in apical three-fourths, sinuate, connected to crenulate stria along apical elytral margin; prosternal striae complete, close, parallel in anterior half, united by narrow arch; male metaventrite not impressed or densely punctate; sterna with faint microsculpture at sides; 1^st^ abdominal ventrite with near complete longitudinal lateral stria in addition to transverse postcoxal stria, postcoxal stria ending in distinct fovea behind outer corner of metacoxa; propygidium with faint microsculpture, pygidium without; propygidial punctures small, separated by about their diameters, present only in basal half to two-thirds; pygidium with fine ground punctures only; marginal pygidial stria finely impressed along extreme apex or absent. Male genitalia (Figs 72A–C, H): accessory sclerites strongly reduced or absent; T8 with sides more or less evenly convergent from base to apex, apical emargination narrow, basal emargination deep, nearly reaching basal membrane attachment line, ventrolateral apodemes not meeting along midline, extending only along middle third of segment; S8 strongly divergent to apex, apical guides well developed, bearing distinctly darkened maculae near apex; T9 lacking lateral denticles; T10 long, halves separate; S9 short, slightly widened toward truncate base, apex with narrow median emargination, rounded on each side, basolateral corners strongly produced proximally; tegmen widest near middle, sides gently curved to base and subacute apex, medioventral process not evident; basal piece about one-third tegmen length; median lobe about half tegmen length, proximal apodemes differentiated medially into thick and filamentous portions.

#### Remarks.

The presence of propygidial microsculpture in this species is unusual for the *impunctipennis* group, and this, in combination with the uninterrupted submarginal pronotal stria that excludes it from the frequently microsculptured *Operclipygus hamistrius* group, is almost sufficient to recognize it. It is also characterized by a small but distinct abdominal fovea behind the posterolateral margin of the metacoxa (Fig. 71B), a complete stria along the posterior margin of the elytra (Fig. 71A), and the presence of only two complete elytral striae (1^st^ and 2^nd^, the 1^st^ rarely abbreviated slightly from apex). Given some variation in propygidial punctation, density of microsculpture, and completeness of various striae, we restrict this type series to specimens from a small area of Puntarenas, Costa Rica.

#### Etymology.

This species’ name is based on its small size and secondarily its reduced (depauperate) elytral striation.

### Operclipygus nicodemus sp. n.

urn:lsid:zoobank.org:act:76762EAC-7E56-4545-B8C7-AE2B23E0A1C0

http://species-id.net/wiki/Operclipygus_nicodemus

Figs 71C–D 72D, I Map 25

#### Type locality.

BELIZE: Cayo:Las Cuevas Research Station [16°44'N, 88°60'W].

#### Type material.

**Holotype male**: “Las Cuevas Belize”/ “FIT 12 5.08.94” / “Caterino/Tishechkin Exosternini Voucher EXO-00317” (BMNH). **Paratypes** (15): 3: same data as type (BMNH, FMNH); 7: same data as type, except as noted: 6: Las Cuevas Research Station, 16°44'N, 88°60'W, 550m, v.1997, FIT, D. Inward (BMNH, MSCC, AKTC), 1: 500–700m, i-ii.1998, D. Inward (BMNH); 1: Gran de Oro, 5–8.vi.1995, FIT, King/Howe/Rosado (BMNH); 1: Chiquibul Forest Reserve, San Pastor, 27.x.1994, FIT (BMNH), 1: 16.ix.1994, FIT (BMNH); **Orange Walk**: 1: Rio Bravo Cons. Area, trail to well, 17°50'38"N, 89°01'0.4"W, 25.iv–5.v.1996, FIT, C.E. Carlton (SEMC); 1: Rio Bravo Cons. Area, rd. to Archaelogical site, 17°50'56"N, 89°02'34"W, 25.iv–5.v.1996, FIT, C.E. Carlton (LSAM).

#### Other material.

**HONDURAS: Atlantida**: 1: 13km. E La Ceiba, 150m, vii.1996, FIT, cocoa plantation, R. Lehman (TAMU), 8: 15–19.vi.1996, FIT, tropical rainforest, R. Lehman (TAMU), 2: 20.vi–20.vii.1996, Malaise trap, tropical rainforest, R. Lehman (TAMU), 6: 175m, 9–30.vii.1996, Malaise trap, tropical rainforest, R. Lehman (TAMU). **MEXICO: Chiapas**: 1: El Aguacero, 16km W Ocozocoautla, 680m, 5–13.vi.1990, FIT, H. & A. Howden (CMNC); 1: Leguna Belgica 16km NW Ocozocoautla, 970m, 31.v.1990, H. & A. Howden (CHSM). **NICARAGUA: Rio San Juan:** 1: El Castillo loc. Bartola, iii.2000, I.G. Trezzi (CHFP).

#### Diagnostic description.

Length: 1.56–1.72 mm, width: 1.28–1.44 mm; body rufobrunneus, elongate oval; frons shallowly depressed at middle, with fine, sparse ground punctation; frontal stria rounded at sides, generally complete across middle; pronotal disk lacking prescutellar impression, with fine, sparse ground punctation, ~16 moderate punctures sparsely scattered at sides; marginal pronotal stria complete behind head and along sides; submarginal stria continuous across front, ending at or before middle of lateral margin; elytra with outer and inner subhumeral striae absent, stria 1 well impressed in basal half only, obsolete or only very weakly impressed in apical half, striae 2-4 complete, 5^th^ stria present in apical half, sutural stria present in apical two-thirds; elytral disk with very few, small apical punctures; prosternal keel weakly impressed at base, carinal striae complete, parallel in apical half, united in narrow anterior arch; mesoventral margin weakly projecting, marginal stria complete or narrowly interrupted; mesometaventral stria broadly arched forward to near anterior mesoventral margin; lateral metaventral stria extending to middle of metacoxa; 1^st^ abdominal ventrite with two abbreviated lateral striae, outer bent abruptly behind metacoxa, fragmented; small postcoxal fovea present near inner corner of metacoxa; propygidium and pygidium with fine, sparse ground punctures; propygidium with medium, ocellate punctures separated by about their diameters in basal third, distinctly smaller and sparser in apical half; pygidium with small punctures uniformly interspersed with ground punctation, separated by 3–4× their diameters; marginal pygidial stria fine, present along about apical third of pygidial margin. Male genitalia (Figs 72D, I): accessory sclerites present; T8 parallel-sided in basal two-thirds, angulate to apex, apical emargination narrow, acute, basal emargination rather narrow, rounded, basal membrane attachment line distad apex of emargination by about one-third its depth; S8 with sides weakly convergent to near apex, then slightly divergent, apical guides narrow, widened weakly at apex; T9 parallel-sided in basal half, convergent to apex; T10 with halves separate; S9 narrow near apex, weakly widened to rounded base, apex without median emargination, apical flange entire, basolateral corners not prolonged proximad; tegmen widest basad middle, apical half narrow, curving evenly ventrad, medioventral process weak, ‘U’-shaped, projecting beneath about one-fourth from base; basal piece just over one-third tegmen length; median lobe about one-half tegmen length, proximal apodemes not strongly differentiated.

#### Remarks.

The pattern of elytral striae (Fig. 71C), with the 1^st^ stria strongly abbreviated from the base and striae 2-4 complete is almost unique. In combination with the basally obsolete lateral submarginal pronotal stria and the basally densely punctate propygidium, this is sufficient to distinguish it from all others in the group. We limit the type series to specimens from Belize, as specimens from other areas show some variation in the density of propygidial punctation.

#### Etymology.

This species is named in memory of Mr. Nicodemus ‘Chapal’ Bol, former caretaker of the Las Cuevas Field Station, the type locality of this species, in recognition for his assistance during a 2000 visit by the first author.

### Operclipygus tripartitus sp. n.

urn:lsid:zoobank.org:act:D4787E14-D54E-4919-8BAC-03F8C531503E

http://species-id.net/wiki/Operclipygus_tripartitus

Figs 71E 72J Map 26

#### Type locality.

COSTA RICA: Heredia: La Selva Biological Station [10°26'N, 84°01'W].

#### Type material.

**Holotype male**: “**COSTA RICA:** Heredia, Est.Biol. LaSelva. 10.26°N, 84.01°W. F.I.T. 22 June 1998 C.Carlton & A.Tishechkin”/ “LSAM0046194” (FMNH). **Paratypes** (77): 42: same data as type, except as noted: 2: 19.vi.1998, FIT, C.E. Carlton & A.K. Tishechkin (LSAM), 1: 20.vi.1998 (LSAM), 10: 21.vi.1998 (LSAM, FMNH, MSCC, AKTC), 1: 22.vi.1998 (LSAM), 3: 23.vi.1998 (LSAM), 7: 24.vi.1998 (LSAM), 5: 25.vi.1998 (LSAM), 4: 26.vi.1998 (LSAM), 4: 28.vi.1998 (LSAM), 1: 29.vi.1998 (LSAM), 1: 5.ii.1992, FIT, W. Bell (SEMC), 1: 24.ii.1992 (SEMC), 1: 21.iii.1992 (SEMC), 1: 24.iii.1992 (SEMC), 1: 28.ii.1992 (SEMC); **Heredia:** 1: Los Arbolitos, Sarapiqui, 30–10m, 20–27.iii.1993, F. Araya (INBIO); **Limón:** 2: Area Cons. Tortuguero, Sector Cerro Cocori, Fca. de E. Rojas, 150m, i.1993, E. Rojas (INBIO), 2: vi.1991 (INBIO), 3: iii.1992, E. Rojas (INBIO), 3: iv.1992, E. Rojas (INBIO), 1: 26.vii–2.viii.1992 (INBIO), 2: 28.v–17.vi.1992 (INBIO), 1: 9–30.xi.1992 (INBIO), 2: xii.1992 (INBIO), 3: ii.1993, E. Rojas (INBIO), 4: iii.1993, E. Rojas (INBIO), 3: iv.1993, E. Rojas (INBIO), 2: ix.1991 (INBIO), 3: v.1993 (INBIO), 1: x.1993 (INBIO), 1: xi.1991 (INBIO).

#### Other material.

**COSTA RICA: Alajuela:** 1: Cano Negro, RNVS Cano Negro, 20m, 5–26.i.1993, K. Flores, (INBIO), 1: 1–23.xi.1994, K.F. Flores, (INBIO); **Cartago:** 1: Parque Nac. Barbilla, Est. Barbilla, Turrialba, Send. a Rio Barbilla, 250m, 14–26.i.2001, FIT, W. Arana (INBIO); **Guanacaste:** 1: Parque Nac. Guanacaste, Est. Pitilla [misspelled Patilla], 10°59'22"N, 85°25'33"W, 610m, 13–15.vii.2000, FIT, J. Ashe, R. Brooks, Z. Falin (SEMC); **Puntarenas:** 3: Rancho Quemado, Peninsula de Osa, 200m, ix.1992, F. Quesada (INBIO), 1: v.1992, F. Quesada y G. Varela (INBIO), 1: vii.1992, F. Quesada (INBIO), 1: x.1992, F. Quesada (INBIO), 2: xii.1991, F. Quesada, (INBIO), 1: 21.iii–7.iv.1992, F. Quesada, (INBIO); 2: Est. Sirena, Playa Sirena, 1–100m, viii.1995, G. Fonseca (INBIO); 1: Parque Nac. Corcovado, Sirena Stn. Rio Pavo Trail, 8°29'5"N, 83°35'33"W, 5m, 25–28.vi.2000, FIT, Z. Falin (SEMC); 1: Est. Esquinas, Peninsula de Osa, x.1993, M. Segura (INBIO); 1: Rincon de Osa, 8°41.141'N, 83°31.117'W, 50m, 23–26.vi.2001, FIT, S. & J. Peck (SEMC). **PANAMA: Colón:** 1: San Lorenzo Forest, 9°17'N, 79°58'W, 15–17.v.2004, FIT, A.K. Tishechkin (AKTC), 1: 17–18.v.2004, FIT, A.K. Tishechkin (AKTC), 1: 18–19.v.2004, FIT, A.K. Tishechkin (AKTC), 2: 24–25.v.2004, FIT, A.K. Tishechkin (GBFM), 1: 25–26.v.2004, FIT, A.K. Tishechkin (GBFM); 1: Parque Nac. San Lorenzo, Achiote, Cafetal A Dist., 09°12'N, 79°58'W, 100m, 26.vi–10.vii.2007, FIT, A. Mercado (GBFM); **Panamá**: 1: Pueblo Nuevo Cave, 9°4'N, 78°45'W, 16.vi.1999, on bat guano, W. Reeves (AKTC); 1: Barro Colorado Isl., 9°11'N, 79°51'W, 1–5.vii.2000, FIT, S. Chatzimanolis (SEMC).

#### Diagnostic description.

Length: 1.72–1.90 mm, width: 1.47–1.62 mm; body rufobrunneus, shining, elongate oval with sides rather broadly rounded; frons depressed at middle, disk very finely and sparsely punctate; frontal stria divergent between eyes, sinuate over antennae, continuous across front; supraorbital stria very weakly impressed, frequently fragmented and/or disconnected from frontal stria; labrum narrow, about 1.8× as wide as long, asymmetrically emarginate apically, with left side slightly more prominent; left mandible untoothed, right with very small basal tooth; pronotal disk faintly impressed in prescutellar area, disk with very fine, sparse ground punctation, with generally few, but up to ~20 lateral punctures; lateral submarginal pronotal stria present in anterior half, curving inward but not continuous with anterior portion in most individuals; anterior submarginal pronotal stria very weakly recurved posterad at sides; median pronotal gland openings about three-fourths pronotal length behind anterior margin; elytra with outer and inner subhumeral striae absent, 1^st^ dorsal stria usually obsolete in apical half, striae 2-3 complete, 4^th^ stria broadly interrupted at middle, present at base and apex, 5^th^ stria represented by short apical fragment, sutural stria present in apical three-fourths; elytral disk with few disorganized apical punctures; prosternal keel weakly emarginate at base, with carinal striae complete, fine, widely separated basally, converging anteriorly; mesoventral margin weakly projecting, marginal stria interrupted for width of prosternum; mesometaventral stria arched forward to near mesoventral margin; lateral metaventral stria extending toward outer corner of metacoxa, abbreviated apically; 1^st^ abdominal ventrite with two abbreviated lateral striae, the outer bent behind metacoxa, short, frequently fragmented; postcoxal fovea inconspicuous; propygidium and pygidium lacking microsculpture; propygidium with small round punctures separated by about their diameters along basal margin, becoming smaller and sparser to apex; pygidium with fine, sparse ground punctation, few or no coarser punctures; marginal pygidial stria very finely impressed at extreme apex or absent. Male genitalia: segments 8-10 indistinguishable from those of *Operclipygus nicodemus* (see Fig. 72I and description above), tegmen (Fig. 72J) differing from that species only slightly in having the medioventral process strongly reduced, barely projecting beneath, and having the dorsoventral curvature of tegmen very weak.

#### Remarks.

This species is distinguished by the interrupted submarginal pronotal stria (Fig. 71E), with the ends of the anterior portion weakly recurved behind the eyes, in combination with the apically abbreviated 1^st^ dorsal stria, the complete 2^nd^-3^rd^ striae, and interrupted 4^th^ stria. We restrict the type series to a relatively small area in northwestern Atlantic lowland Costa Rica, as there is some variation in body shape and striation. Specimens from Panamá, in particular, were initially considered to represent a distinct species. However, in most characters, including male genitalia, the various included populations are indistinguishable.

**Map 26.**
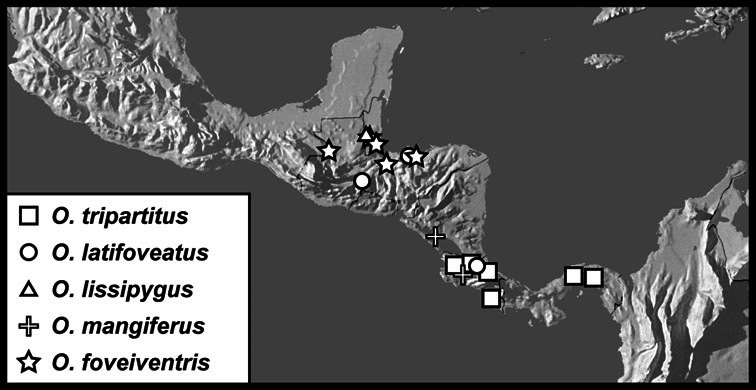
Records of the *Operclipygus impunctipennis* group.

#### Etymology.

This species’ name refers to its three-part submarginal pronotal stria.

### Operclipygus latifoveatus sp. n.

urn:lsid:zoobank.org:act:664DD897-AC23-4916-919F-180CFDFC84E7

http://species-id.net/wiki/Operclipygus_latifoveatus

Figs 71F 72E–G, K Map 26

#### Type locality.

HONDURAS: Atlántida: 15 km W La Ceiba [15°47'N, 86°56.5'W].

#### Type material.

**Holotype male**: “HONDURAS: Atlantida, 15 km. W. La Ceiba, VII-9-30-1996, 175m. Coll. R. Lehman” **/** “Malaise trap, tropical rainforest” / “SEMC0903639 KUNHM-ENT” (SEMC). **Paratypes** (6): **HONDURAS, Atlantida**: 2: 15km W La Ceiba, 15–19.vi.1996, FIT, tropical rainforest, R. Lehman (TAMU); 1: 13 km E La Ceiba, vii.1995, 150m, R. Lehman (TAMU). **GUATEMALA: Zacapa:** 1: 3.5km SE La Union, 1500m, 4.vi.1991, cloud forest litter, R. Anderson (SEMC), 2: 25–27.vi.1993, FIT, J. Ashe, R. Brooks (SEMC).

#### Other material.

**COSTA RICA: Heredia:** 1: La Selva Biol. Stn., 3km S Puerto Viejo, 50m, 19.ii.1980, H. & A. Howden (CMNC).

#### Diagnostic description.

Length: 1.50–2.03 mm, width: 1.31–1.78 mm; body rufopiceous, ovoid, widest near middle of elytra; frons depressed at middle; frontal stria rounded at sides, complete or interrupted over antennal bases, arcuate across middle; labrum narrow, less than twice as wide as long, apex asymmetrically emarginate, left side protruding, with small apical process beneath margin; left mandible untoothed, right with small acute basal tooth; pronotal disk without prescutellar impression, disk with very fine, inconspicuous ground punctation, with ~16-20 coarse punctures near sides; marginal pronotal stria complete to narrowly interrupted behind head; submarginal pronotal stria continuous across front, fine, close to margin, and frequently interrupted at sides; elytron with single complete epipleural stria, subhumeral striae absent, stria 1-2 complete, striae 3-4 present basally and apically, but broadly interrupted at middle, 5^th^ stria absent, sutural present in apical two-thirds, continuous apically with strong apical marginal stria; prosternum depressed, with weak basal emargination, carinal striae complete, narrowed between coxae, slightly bulbous anteriorly, with secondary carinal striae in basal half; mesoventral margin very weakly projecting, marginal stria interrupted; mesometaventral stria broadly, subangulately arched forward to near mesoventral margin; lateral metaventral stria extending posterad to middle of metacoxa; 1^st^ abdominal ventrite with single arcuate lateral stria, present in basal two-thirds of ventrite, with detached, transverse postmetacoxal stria which passes through a deep fovea behind posterolateral corner of coxa; ground punctation of pygidia fine, inconspicuous, microsculpture absent; propygidium with moderately large, shallow punctures separated by less than their diameters in basal half, apical half with only few small punctures; pygidium with small punctures sparsely scattered by about 5× their diameters; marginal pygidial stria fine, present along apical half of margin only. Male genitalia (Figs 72E–G, K): accessory sclerites present, very small; T8 short, sides parallel in basal half, angulate and convergent from near midpoint to apex, basal emargination very deep, reaching basal membrane attachment line, apical emargination almost absent, ventrolateral apodemes subacute ventrally, nearly meeting at midline beneath; S8 with sides parallel in basal half, divergent and downturned to subacute apices, apical guides most strongly developed at middle, narrowed to base and apex, ventral halves fused at base, evenly divergent to apices; T9 with sides weakly convergent in basal two-thirds, more strongly convergent to narrow, subacute apices; T10 weakly sclerotized, halves separate; S9 short, wide, with sides mostly parallel, weakly narrowed near apex, apex broadly, angulately emarginate, with apical flange interrupted at middle, basolateral corners prolonged as fine filaments; tegmen more or less parallel-sided in basal two-thirds, narrowed to apex, medioventral process vestigial, barely visible as a narrowly ‘V’-shaped, weakly sclerotized ventral thickening, not at all projecting beneath; tegmen in lateral view constricted at base, evenly and weakly curving to apex; median lobe about two-thirds tegmen length; basal piece about one-fifth tegmen length.

#### Remarks.

**Figure 73.**
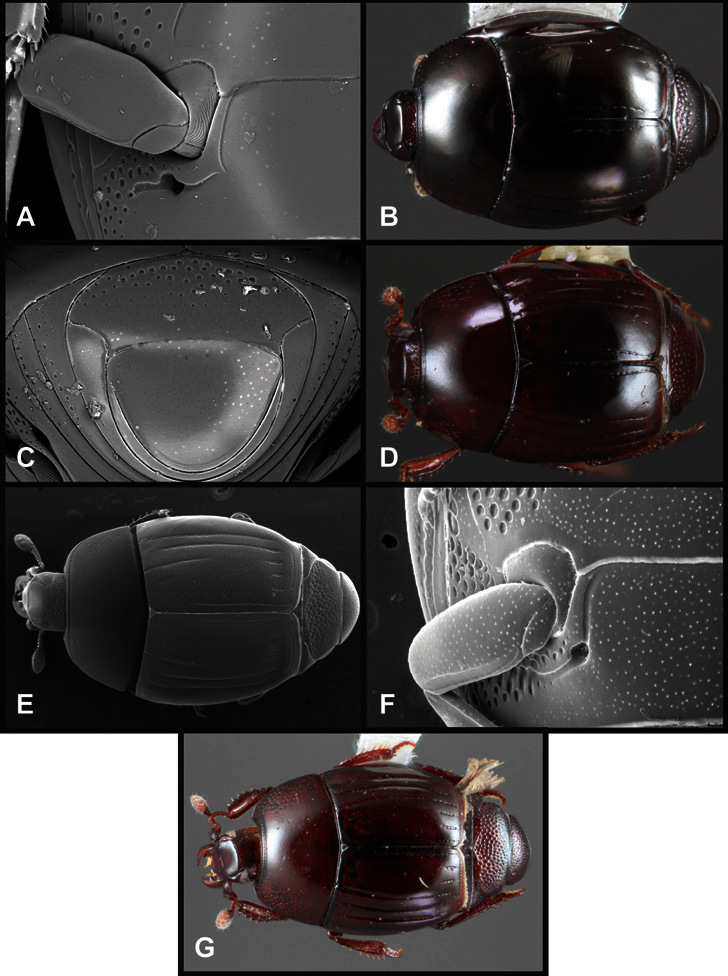
*Operclipygus impunctipennis* group. **A** 1^st^ abdominal ventrite of *Operclipygus lissipygus* **B** Dorsal habitus of *Operclipygus lissipygus* **C** Pygidia of *Operclipygus lissipygus* **D** Dorsal habitus of *Operclipygus mangiferus* **E** Dorsal habitus of *Operclipygus foveiventris* **F** 1^st^ abdominal ventrite of *Operclipygus foveiventris* **G** Dorsal habitus of *Operclipygus marginipennis*.

Among members of the *Operclipygus impunctipennis* group, this species can be recognized by the interrupted 3^rd^ and 4^th^ dorsal elytral striae (Fig. 71F), the strong, complete apical marginal elytral stria, and the large fovea behind the outer corner of the postcoxa (shared with the following; see Fig. 73A). We exclude one, disjunct specimen from Costa Rica from the type series.

#### Etymology.

The name of this species refers to the conspicuous foveae on the sides of the first abdominal ventrite.

### Operclipygus lissipygus sp. n.

urn:lsid:zoobank.org:act:D12A8338-77DD-4CFC-9628-17DFC750818E

http://species-id.net/wiki/Operclipygus_lissipygus

Figs 73A–C Map 26

#### Type locality.

BELIZE: Cayo:Las Cuevas Research Station [16°44'N, 88°60'W].

#### Type material.

**Holotype male**: “Las Cuevas Belize” / “FIT 11, 5.08.94”/ “Caterino/Tishechkin Exosternini Voucher EXO-00324” (BMNH). **Paratypes** (14): 12: same data as type, except as noted: **BELIZE: Cayo:** 3: Las Cuevas Research Station, 16°43.99'N, 88°59.20'W, 550m, 22.v.2000, sifted under rotting fruit, M.S. Caterino, DNA Extract MSC-0066 (MSCC, AKTC), 1: 30.v.2000, FIT (FMNH), 1: v.1996 (BMNH), 2: 550m, v.1997, D. Inward (BMNH), 2: 11.viii.1994, FIT (BMNH), 1: 5.viii.1994, FIT (BMNH), 2: 16.ix.1994, FIT (BMNH); 1: New Maria, 20.i.1995, FIT (BMNH).

#### Diagnostic description.

This species is extremely similar to the preceding differing only as follows length: 1.81–1.87 mm, width: 1.56–1.62 mm; frontal stria complete over antennal bases; lateral submarginal pronotal stria continuous along front and sides, not interrupted; pronotal disk with <10 lateral punctures; elytra with 2 epipleural striae, striae 1-2 complete, 3^rd^ stria present in basal third as very fine stria, and a short apical fragment, striae 4-5 absent, sutural stria present in apical two-thirds; apical margin of elytra lacking stria or punctures; prosternum not strongly depressed; marginal stria of mesoventrite broadly interrupted; inner stria of 1^st^ abdominal ventrite not extending beyond basal half, ending very close to or meeting inner end of transverse postmetacoxal stria; deep fovea present behind posterolateral corner of metacoxa; propygidium with rather small punctures concentrated along basal margin, sparser to absent in apical half; pygidium with few or no punctures aside from very fine, inconspicuous ground punctation; marginal pygidial stria very fine, present only on extreme pygidial apex. Male genitalia indistinguishable from those of *Operclipygus latifoveatus* (see Figs 72E–G, K).

#### Remarks.

The presence of a deep fovea behind the metacoxa (Fig. 73A) in combination with the fine, abbreviated 3^rd^ dorsal stria, absent 4^th^ dorsal stria (Fig. 73B), lack of marginal elytral stria, smooth pygidium and few propygidial punctures (Fig. 73C) complete frontal stria, and small number of lateral pronotal punctures will distinguish this species from others in this group.

#### Etymology.

This species’ name refers to the very smooth, almost impunctate pygidium.

### Operclipygus mangiferus sp. n.

urn:lsid:zoobank.org:act:65741C0D-6FE3-4ED6-AFB5-6A6A0768572A

http://species-id.net/wiki/Operclipygus_mangiferus

Figs 73D 74A–D Map 26

#### Type locality.

COSTA RICA: Guanacaste: Liberia [10°38'N, 85°26'W].

#### Type material.

**Holotype male**: “COSTA RICA: Guanacaste, Liberia, 20 May 1993, 50m, J. & A. Ashe #047, ex: rotting mangos”/ “SEMC0903635 KUNHM-ENT” (INBIO). **Paratypes** (8): 1: same data as type; 1: Parque Nac. Barra Honda, 100m, iv.1995, M. Reves, (INBIO); 1: Est. Palo Verde, Ref. Nac. Fauna Silv., R.L. Rodriguez, 10m, vi.1991, U. Chavarria, (INBIO); 1: Parque Nac. Guanacaste, 9km S Santa Cecilia, Est. Pitilla, 13.viii.1966, carrion trap, S. Peck (FMNH). **NICARAGUA: Granada**: 1: Volcan Mombacho, Santa Ana #1, 2.vi.1998, malaise trap, J.M. Maes (MEL); **Masaya**: 3: Las Flores, vi.1994, malaise trap, J.M. Maes (MEL).

#### Diagnostic description.

**Figure 74.**
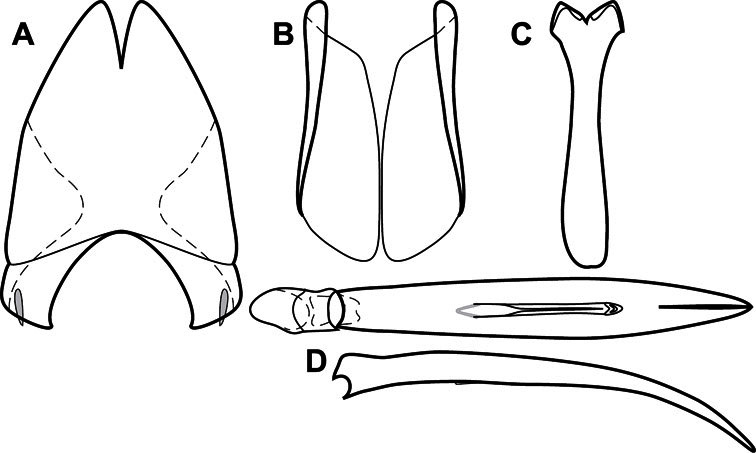
Male genitalia of *Operclipygus mangiferus* (*Operclipygus impunctipennis* group). **A** T8 **B** S8 **C** S9 **D** Aedeagus, dorsal and lateral views.

Length: 1.75–1.81 mm, width: 1.47–1.59 mm; body rufobrunneus, elongate oval, sides weakly rounded; frons weakly depressed, with fine, sparse ground punctation; sides of frontal stria short, impressed near upper margin of eye, broadly interrupted over antennal bases, short, transverse across middle; labrum narrow, less than twice as wide as long, apex asymmetrically emarginate, left side protruding, with small apical process beneath margin; left mandible untoothed, right with small acute basal tooth; pronotal disk lacking prescutellar impression, with fine, inconspicuous ground punctation and numerous coarse punctures at sides; marginal pronotal stria complete or narrowly interrupted behind head; submarginal pronotal stria continuous across anterior margin, extending along only anterior third to half of lateral margin, obsolete basally; elytron with two complete epipleural striae, inner and outer subhumeral striae absent, striae 1-3 complete, well impressed, 4^th^ stria present in basal third and as apical fragment, 5^th^ stria absent, sutural stria present in apical two-thirds, very briefly curving laterad at apex, continued along apex by sparse series of punctures; prosternal keel truncate to very weakly emarginate at base, with complete carinal striae which are very narrowly separated anteriorly in most individuals; anterior mesoventral margin very weakly projecting, marginal stria very fine but usually complete between broadly arched mesometaventral stria and mesoventral margin; lateral metaventral stria extending to middle of metacoxa; 1^st^ abdominal ventrite with two lateral striae, both obsolete in posterior half; lacking postmetacoxal stria and fovea; propygidium and pygidium lacking microsculpture; propygidium with medium-sized, round, shallow punctures irregularly separated by about their diameters in basal half, punctures smaller and sparser in apical half; pygidium wide, short, about equal in midline length to propygidium, with fine, sparse ground punctures and slightly coarser punctures evenly scattered throughout; marginal pygidial stria absent. Male genitalia (Fig. 74A–D) very similar to those of *Operclipygus latifoveatus*, differing as follows: T8 with apical emargination distinct, narrow, ventrolateral apodemes well separated along midline; S8 with sides subparallel, apical guides narrow throughout, halves not fused ventrobasally; S9 with base more narrowly rounded, apex with very small median emargination, basolateral corners not prolonged proximad; tegmen widening slightly from base, widest one-third from apex, narrowed to subacute apex, medioventral process very weak, not or barely projecting beneath; median lobe about one-half tegmen length.

#### Remarks.

This species is rather isolated in the group, completely lacking any marginal pygidial stria, and having strongly impressed elytral striae 1-3 (Fig. 73D). These characters, in addition to the anteriorly continuous, but posteriorly obsolete submarginal pronotal striae are sufficient to distinguish it.

#### Etymology.

The name of this species refers to the discovery of the types of this species in association with rotting mangoes, though this is probably not an exclusive habit.

### Operclipygus foveiventris sp. n.

urn:lsid:zoobank.org:act:94D071DF-125A-439D-8AED-77479A8C28E0

http://species-id.net/wiki/Operclipygus_foveiventris

Figs 73E–F 75A–C, G Map 26

#### Type locality.

BELIZE: Cayo: Las Cuevas Research Station [16°44'N, 88°60'W].

#### Type material.

**Holotype male**: “BELIZE: Cayo, Las Cuevas Res. Sta. 550m, v.1997, D. Inward”/ “Caterino/Tishechkin Exosternini Voucher EXO-00323” (BMNH). **Paratypes** (31): 29: same data as type, except as noted: 13: same data as type (BMNH, FMNH, MSCC, AKTC, CHND),2: 16°44'N, 88°59'W, 500–700m, i-ii.1998 (BMNH), 1: iv.1996 (BMNH), 2: v.1996 (BMNH), 2: vi.2006, J. Kitson (BMNH), 1: 5.viii.1994, FIT (BMNH), 1: 22–23.v.2000, FIT, M.S. Caterino (MSCC), 1: 23.v.2000 (MSCC), 1: 11.viii.1994, FIT (BMNH), 2: 5.viii.1994, FIT (BMNH), 1: 16.ix.1994, FIT (BMNH); 2: 16°43.971'N, 88°59.196'W, 580m, 1–4.vi.2008, B. Ratcliffe, R. Cave, M. Jameson, J. Orozco (UNL); 1: New Maria, 20.i.1995, FIT (BMNH); **Stann Creek**: 1: Cockscomb Basin Wildlife Sanctuary, 16°46.830'N, 88°27.543'W, 80m, 30–31.v.2008, B. Ratcliffe, R. Cave, M. Jameson, J. Orozco (UNL).

#### Other material.

**HONDURAS: Atlantida**: 3: 13km E La Ceiba, 150m, vii.1996, FIT, cocoa plantation, R. Lehman (TAMU); 3: 15km W La Ceiba, 15–19.vi.1996, FIT, tropical rainforest, R. Lehman (TAMU), 1: 20.vi-–20.vii.1996, Malaise trap, tropical rainforest, R. Lehman (TAMU), 1: 15–19.vi.1996, R. Lehman (TAMU); **Cortés**, 1: Parque Nac. Cusuco, 5km N Buenos Aires, 15°29'N, 88°13'W, 15.viii.1995, Malaise trap, oak/pine cloud forest, R. Cave (AKTC), 1: 30.viii.1995, Malaise trap, oak/pine cloud forest, R. Cave (AKTC); 7: Lago Yojoa, 650m, 23–28.viii.1994, FIT, tropical, evergreen forest, S. & J. Peck (CMNC); 1: Yojoa Lake, Deer Island, 14°55'N, 87°58'W, 670m, 22–26.vi.1994, FIT, J. Ashe, R. Brooks (SEMC). **MEXICO: Chiapas**: 1: Playon de la Gloria, 16.16036°N, 90.90148°W, 160m, 24.vi.2008, sifted leaf litter, mature wet forest (SEMC).

#### Diagnostic description.

**Figure 75.**
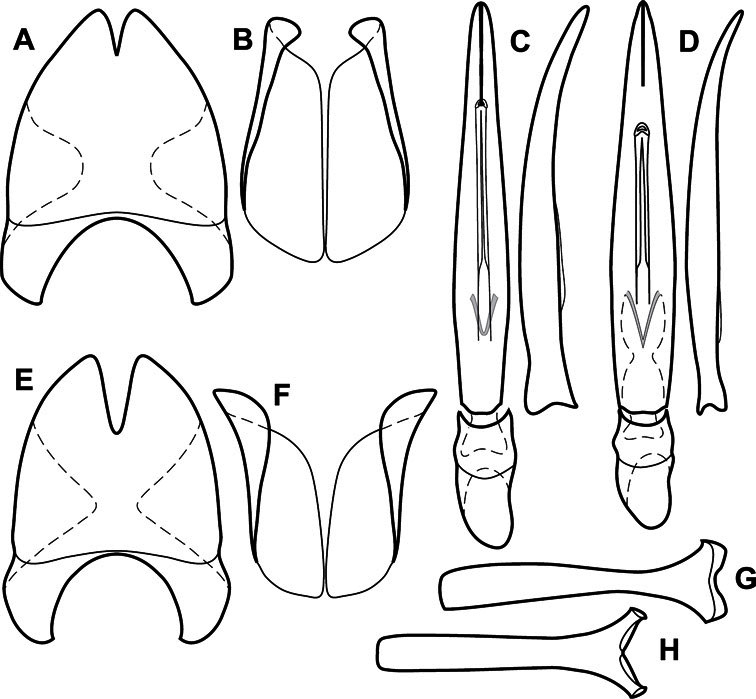
Male genitalia of *Operclipygus impunctipennis* group. **A** T8 of *Operclipygus foveiventris* **B** S8 of *Operclipygus foveiventris* **C **Aedeagus, dorsal and lateral views, of *Operclipygus foveiventris* **D** Aedeagus, dorsal and lateral views, of *Operclipygus maesi* **E** T8 of *Operclipygus maesi* **F** S8 of *Operclipygus maesi* **G** S9 of *Operclipygus foveiventris* **H** S9 of *Operclipygus maesi*.

Length: 2.31–2.71 mm, width: 2.06–2.25 mm; body rufopiceous, oval, with sides distinctly rounded, strongly convex; frons and upper part of epistoma strongly impressed; frontal stria divergent between eyes, interrupted over antennal bases, arcuate across middle; labrum about twice as wide as long, weakly emarginate apically; pronotal disk weakly impressed in prescutellar region, but lacking discrete impression, with very fine, inconspicuous ground punctation and ~20 coarse lateral punctures; marginal pronotal stria complete along anterior and lateral margins; submarginal pronotal stria continuous across front, extending along anterior half of lateral margin; median pronotal gland openings about two-thirds pronotal length from anterior margin; elytron with two complete epipleural striae, inner and outer subhumeral striae generally absent, rarely with small fragments of outer present, striae 1-2 complete, 3^rd^ stria present in basal third and as short apical fragment, 4^th^ stria present in basal sixth and as short apical fragment, 5^th^ stria absent, sutural stria present in apical three-fourths, curving laterad apically but usually not connected to apical marginal stria, which is present between striae 2 and 5; prosternum with microsculpture on keel and on prosternal lobe; microsculpture lacking on posterior ventrites; prosternal keel truncate at base, carinal striae complete, converging from base to middle, subparallel anteriorly, connected in narrow anterior arch; weak secondary carinal striae present mediad procoxa; mesoventrite very wide and short, mesoventral margin weakly sinuate, not projecting, marginal stria broadly interrupted; mesometaventral stria strongly and broadly arched forward to near mesoventral margin; lateral metaventral stria extending posterad toward inner third of metacoxa, abbreviated behind middle; 1^st^ abdominal ventrite with single lateral stria present in basal half, passing through deep fovea behind inner corner of metacoxa, continuing laterad behind coxa; propygidium and pygidium with dense, shallow ground punctation; propygidium with slightly elongate punctures separated by about half their diameters basomedially, smaller and sparser laterally and apically; pygidium with small coarse punctures rather uniformly scattered by about 4× their diameters; marginal pygidial stria absent. Male genitalia (Figs 75A–C, G): accessory sclerites absent; T8 short, with sides weakly rounded, subparallel in basal half, convergent to apex, apical emargination narrow, shallow, basal emargination broadly rounded, deep, reaching to basal membrane attachment line, ventrolateral apodemes small, distant ventrally; S8 with sides converging to apex, apical guides very narrow, truncate apically, ventral halves approximate at base, diverging slightly to apex; T9 with sides subparallel in basal half, convergent to apex, apices narrow, acute on inner corner; T10 with halves separate; S9 narrow, elongate, narrowest proximad apex, sides in basal half subparallel, asymmetrical, apex shallowly inwardly arcuate, without median emargination, apical flange narrowed at middle but not interrupted; basolateral corners not prolonged proximad; tegmen widest near base, weakly sinuately narrowed to apex, medioventral process present as a narrow ‘V’-shaped sclerite about one-fifth from tegmen base, entirely contained in ventral membrane, not projecting beneath; median lobe narrow, about four-fifths tegmen length, proximal apodemes not strongly differentiated; basal piece about one-third tegmen length.

#### Remarks.

This species can be easily recognized by the pattern of elytral striation (Fig. 73E), with the 3^rd^ and 4^th^ striae broadly interrupted and the fifth represented by only a very short apical fragment, the presence of an apical elytral stria, the presence of a large postmetacoxal fovea behind the inner corner of the coxa (Fig. 73F; not displaced laterad, like in a few species above), the dense ground punctation of the pygidia, and the complete absence of a marginal pygidial stria. We restrict the type series to those specimens from Belize.

#### Etymology.

This species is named for the distinct, large fovea behind the metacoxa.

### Operclipygus marginipennis sp. n.

urn:lsid:zoobank.org:act:461FE05C-0E10-4806-A0DA-ADA5E67939D5

http://species-id.net/wiki/Operclipygus_marginipennis

Figs 73G Map 27

#### Type locality.

BELIZE: Orange Walk: Rio Bravo Conservation Area [17°50.93'N, 89°2.57'W].

#### Type material.

**Holotype male**: “BELISE: Orange Walk Dist. Rio Bravo Conserv. Area (rd. to Archaeological site) 17°50'56"N, 89°02'34"W, 25-IV to 5-V-1996, C. Carlton #101, flt.intcpt. trap #1”/ “SM0051140 KUNHM-ENT” (SEMC). **Paratypes** (3): 3: same data as type, except as noted: 1: same data as type, 1: 18–25.iv.1996, FIT, C.E. Carlton & V. Moseley (SEMC), 2: La Milpa Field Stn., 17°50'N, 89°01'W, 15–25.v.1997, FIT, C.E. Carlton (LSAM).

#### Other material.

**MEXICO: Chiapas**: 1: “Encinar 16 de septiembre” Carretera Tuxtla Gtz.-Chicoasen Loc. Cn., 16°48'60"N, 93°10'43"W, 851m, 22.iv.2005, dead rat bait (ECOSUR), 1: 25.iv.2005, dead rat bait (ECOSUR), 2: 24.iv.2005, dead rat bait (ECOSUR, MSCC), 1: 2.v.2005, dead rat bait (FMNH); **Tabasco**: 3: Reserva Balancan - Tenosique, 28.iii.1976, Cebo Pescado Noche-Dia, disturbed deciduous forest, P. Reyes C. (ZISP).

#### Diagnostic description.

This species is extremely similar to the preceding, differing mainly in the following characters: length: 2.03–2.37 mm, width: 1.72–2.00 mm; body slightly narrower, less broadly rounded; pronotum with more numerous, ~40, lateral discal punctures; elytron with subhumeral striae absent, striae 1-2 complete, 3^rd^ stria complete or rarely narrowly interrupted at middle, 4^th^ stria present in basal fourth and as short apical fragment, 5^th^ stria represented by short apical fragment, sutural stria present in apical two-thirds, more frequently connected to apical marginal elytral stria (about half of available specimens); 1^st^ abdominal ventrite with a small fovea behind the inner corner of metacoxa, lateral stria present, ending in fovea, not continuing laterad behind metacoxa; propygidium and pygidium with similar dense ground punctation, the coarse punctures of propygidium much more dense and numerous, coarse punctures of pygidium also more numerous; apical marginal stria absent. Male genitalia indistinguishable from those of *Operclipygus foveiventris* (see Figs 75A–C, G).

#### Remarks.

*Operclipygus marginipennis* is very similar to the preceding species but differs by its small postmetacoxal fovea, usually complete 3^rd^ dorsal elytral stria, and more numerous lateral pronotal punctures (Fig. 73G). We restrict the type series to those specimens from Belize, due to evident variation in completeness of the 3^rd^ elytral stria, pronotal punctation, and ventral striae among localities.

#### Etymology.

This species’ name refers to the apical stria along the posterior elytral margin.

### Operclipygus maesi sp. n.

urn:lsid:zoobank.org:act:88CB2444-E88E-44AB-8B83-3343DEE3392D

http://species-id.net/wiki/Operclipygus_maesi

**Map 27.**
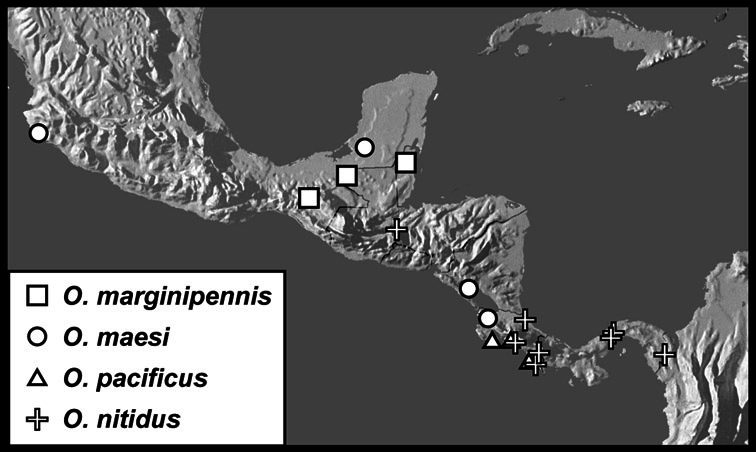
Records of the *Operclipygus impunctipennis* group.

Figs 75D–F, H 76A–B Map 27

#### Type locality.

NICARAGUA: Granada: Mombacho Volcano [11°50'N, 85°59'W].

#### Type material.

**Holotype male**: “NICARAGUA – Granada, Volcan Mombacho, San Joaquin #3, malaise trap, 15.XII.1998 J.M. Maes legit”/ “Caterino/Tishechkin Exosternini Voucher EXO-00325” (FMNH). **Paratypes** (8): 7: same data as type, except as noted: 1: 15.v.1998 (MEL), 2: 15.xi.1998 (MEL, FMNH), 1: 30.vi.1998 (AKTC), 1: Santa Ana #2, 15.v.1998 (MSCC), 1: Santa Ana #3, 2.vi.1998 (FMNH), 1: Bosque seco, 28.ii.1999 (FMNH); 1: Reserva Domitila, 11°42.50'N, 85°57.20'W, 100m, –9.vi.2002, FIT, R. Brooks, Z. Falin, S. Chatzimanolis (SEMC).

#### Other material.

**COSTA RICA: Guanacaste:** 1: Parque Nac. Guanacaste, Playa Naranjo, Sta Rosa, i.1991, E. Alcazar, (INBIO); 1: Parque Nac. Barra Honda, 3km NO de Nacaome, 100m, x.1993, M. Reyes, (INBIO). **MEXICO: Campeche**: 1: Escarcega, 6km W, El Tormento, 110m, 12–23.viii.1983, FIT, evergreen trop. For., S. & J. Peck (CMNC); **Jalisco**: 6: Chamela Biol. Stn., 14.vii.1989, FIT, R. Brooks (SEMC, CHSM); 3:16–20.vii.1989, FIT, R. Brooks (SEMC).

#### Diagnostic description.

Length: 2.18–2.25 mm, width: 1.87–1.90 mm; body rufopiceus, elongate oval, widest at humeri, subdepressed; frons and epistoma depressed at middle; frontal stria rounded at sides, interrupted over antennal bases, arcuate at middle; labrum about 2.5× as wide as long, emarginate apically; pronotum lacking prescutellar impression, with fine, sparse ground punctation and ~24 coarse lateral punctures; marginal pronotal stria complete along anterior and lateral margins; submarginal pronotal stria continuous behind anterior margin, extending posterad along anterior half to two-thirds of lateral margin, obsolete at base; median pronotal gland openings about two-thirds pronotal length behind anterior margin; elytra with two complete epipleural striae, outer subhumeral stria present in apical third to one-half, inner subhumeral stria absent, striae 1-4 complete, 5^th^ stria present in apical third to one-half, sutural stria present in apical two-thirds; elytron with apical marginal series of small punctures; pronotal keel truncate at base, carinal striae complete, keel microsculptured between; prosternal lobe rather short; anterior mesoventral margin straight, marginal stria interrupted at middle; mesometaventral stria broadly arched forward about two-thirds mesoventral length toward anterior margin; lateral metaventral stria extending toward middle of metacoxa; 1^st^ abdominal ventrite with two lateral striae, inner stria complete, the outer stria apically abbreviated; postmetacoxal fovea inconspicuous; propygidium with dense ground punctation visible at sides and along apical margin, with coarser round punctures fairly uniformly and densely separated by about one-half their diameters; pygidium with fine, dense ground punctation throughout, with small secondary punctures uniformly separated by about twice their diameters; marginal pygidial stria absent. Male genitalia (Figs 75D–F, H): accessory sclerites absent; T8 sides arcuately convergent to near apex, then angulate to apex, apical emargination rather deep, basal emargination broad, deep, reaching basal membrane attachment line, ventrolateral apodemes subacute at ventral corners, not meeting beneath; S8 with sides divergent to apex, with apical guides widening markedly from base, ventral halves approximate in basal half, divergent to apex; T9 sides subparallel in basal half, convergent to narrow apices, T10 halves separate; S9 sides subparallel in basal two-thirds, base narrowly truncate; apex with narrow median emargination, apical flange interrupted, lateral flanges narrow; tegmen widest just distad base, narrowed in apical two-thirds, with slight ventral curvature near apex, medioventral process evident, narrowly ‘V’-shaped, mostly contained in ventral membrane, only weakly projecting beneath; median lobe about two-thirds tegmen length, proximal apodemes differentiated, with proximal filamentous ends short; basal piece about one-third tegmen length.

#### Remarks.

**Figure 76.**
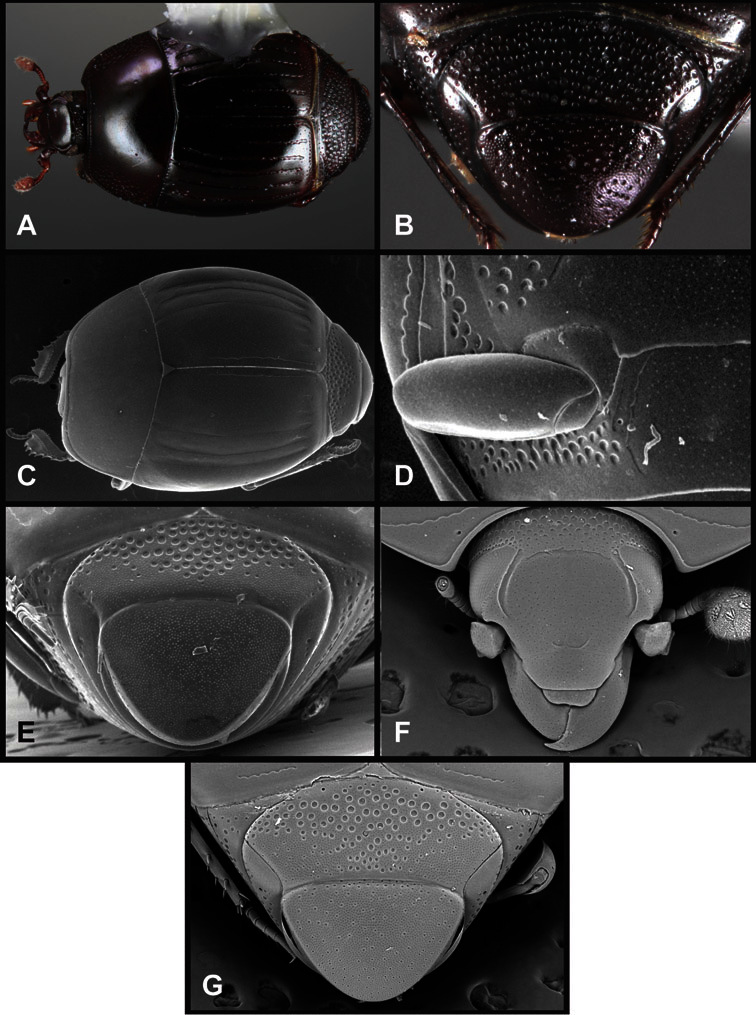
*Operclipygus impunctipennis* group. **A** Dorsal habitus of *Operclipygus maesi* **B** Pygidia of *Operclipygus maesi* **C **Dorsal habitus of *Operclipygus pacificus* **D** 1^st^ abdominal ventrite of *Operclipygus pacificus* **E** Pygidia of *Operclipygus pacificus* **F** Frons of *Operclipygus nitidus* **G** Pygidia of *Operclipygus nitidus*.

Among species in the *Operclipygus impunctipennis* group, this species can be distinguished by the well impressed outer subhumeral stria in the apical half, basally obsolete submarginal pronotal stria, 4 complete dorsal elytral striae (Fig. 76A), and combination of dense ground punctation as well as high density of larger punctures on both the propygidium and pygidium (Fig. 76B). Due to variability in pronotal punctation and completeness of the lateral stria we restrict this type series to those specimens from central Nicaragua.

#### Etymology.

We name this species for Jean-Michel Maes, director of the Entomological Museum of Leon, Nicaragua, who has tirelessly promoted appreciation of insect biodiversity in Central America.

### Operclipygus pacificus sp. n.

urn:lsid:zoobank.org:act:F35CDB10-1DF4-44B7-9445-C2284D907764

http://species-id.net/wiki/Operclipygus_pacificus

Figs 76C–E 77A, C, E, H Map 27

#### Type locality.

COSTA RICA: Puntarenas: Quebrada Bonita Station [9°50'N, 85°0'W].

#### Type material.

**Holotype male**: “Est. Q. Bonita, Prov. Punta, COSTA RICA. 50m. Set 1993. R. Guzmán, L N 194500_469850 #2349”/ “INBIO CRI001659037” (INBIO). **Paratypes** (21): 17: same data as type, except as noted: 1: ix.1994, R.M. Guzman, (INBIO), 2: ix.1994, R.M. Guzman, (INBIO, FMNH), 2: ix.1994, J.C. Saborio, (INBIO), 1: v.1994, J. Saborio, (INBIO), 2: v.1994, R.M. Guzman, (INBIO), 1: vii.1992, J.C. Saborio, (INBIO), 1: x.1994, J.C. Saborio (INBIO), 3: xi.1994, R. Guzman (INBIO, FMNH), 1: 10–28.viii.1992, R. Guzman (INBIO), 1: 6–27.xi.1992, R. Guzman (INBIO), 1: 2–23.ix.1992, R. Guzman (INBIO), 1: 80m, xi.1994, J.C. Saborio (INBIO); 4: Res. Biol. Carara, Sector Laguna Meandrica, LN1979000,472800, 100m, R. Zuninga, vi.1990 (INBIO, MSCC, AKTC).

#### Other material.

**COSTA RICA: Puntarenas:** 1: Rancho Quemado, Peninsula de Osa, 200m, v.1991, J.C. Saborio, (INBIO), 1: ix.1992, M. Segura (INBIO), 1: 12–24.v.1993, A. Gutierrez (INBIO); 1: Parque Nac. Corcovado, Est. Sirena, 0–100m, xi.1990, C. Saborio (INBIO).

#### Diagnostic description.

**Figure 77.**
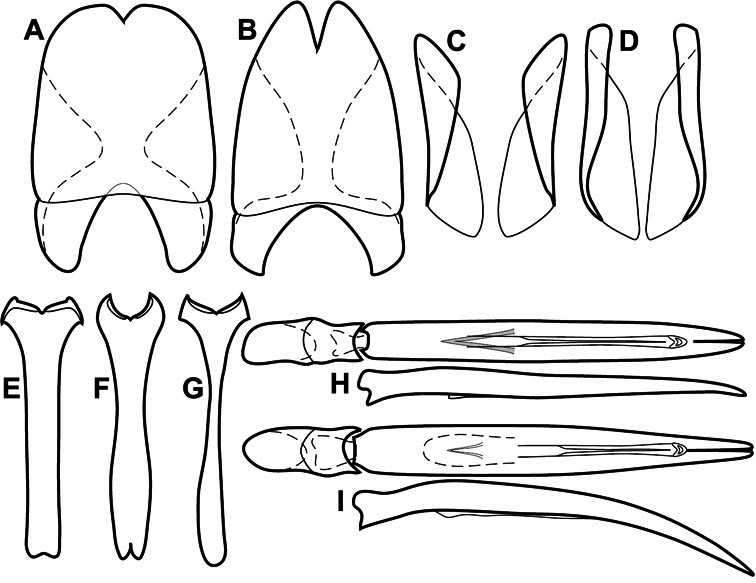
Male genitalia of *Operclipygus impunctipennis* group. **A** T8 of *Operclipygus pacificus* **B** T8 of *Operclipygus subviridis* **C** S8 of *Operclipygus pacificus* **D** S8 of *Operclipygus subviridis* **E** S9 of *Operclipygus pacificus* **F** S9 of *Operclipygus nitidus* **G** S9 of *Operclipygus subviridis* **H** Aedeagus, dorsal and lateral views, of *Operclipygus pacificus* **I** Aedeagus, dorsal and lateral views, of *Operclipygus subviridis*.

Length: 1.75–2.37 mm, width: 1.68–2.12 mm; body rufopiceous, strongly rounded, convex; frons broad, slightly depressed in middle; frontal stria just turning inward between eyes, absent from middle; labrum less than twice as wide as long, weakly emarginate apically; left mandible untoothed, right with small, subacute basal tooth; pronotal disk with prescutellar impression weak, with very fine prescutellar fovea in some individuals, ground punctation fine and inconspicuous, with ~10 coarser lateral punctures; marginal pronotal stria broadly interrupted behind head; submarginal pronotal stria continuous across front and sides, rarely abbreviated or fragmented at sides; median pronotal gland openings about two-thirds pronotal length behind anterior margin; elytra with two complete epipleural striae, outer and inner subhumeral striae absent, striae 1-2 complete, 3^rd^ stria present in basal third and apical one-sixth, rarely complete, 4^th^ and 5^th^ striae present as apical rudiments, sutural stria present in apical four-fifths; apical elytral stria absent; prosternal keel broad, flat, weakly emarginate at base, carinal striae well-separated at base, sinuate, joined anteriorly in broad arch; prosternal lobe short; mesoventrite short, wide, weakly projecting anteriorly, marginal stria narrowly interrupted at middle; mesometaventral stria arched forward to middle of mesoventrite, sinuate at sides; lateral metaventral stria extending toward outer third of metacoxa, slightly abbreviated at apex; 1^st^ abdominal ventrite with complete inner lateral stria, abbreviated outer lateral stria, with fine postmetacoxal fovea between them; propygidium and pygidium with dense, fine, shallow ground punctation; propygidium with coarse basomedial punctures, sparser laterally and posteriorly; pygidium lacking any interspersed coarse punctures; apical marginal stria absent. Male genitalia (Figs 77A, C, E, H): accessory sclerites absent; T8 short, sides subparallel in basal two-thirds, arcuately convergent to apex, apical emargination shallow, basal emargination deep, rather narrow, intersecting basal membrane attachment line, ventrolateral apodemes subacute medially, not meeting at midline; S8 with halves well separated, sides weakly divergent to blunt apices, apical guides widened from base to apex; T9 with sides parallel in basal two-thirds, convergent to narrow apices; T10 with halves separate; S9 narrow, sides subparallel, base narrowly emarginate, apex with very fine median emargination, apical flange interrupted; tegmen very narrow, somewhat sinuate in lateral view, medioventral process narrowly ‘V’-shaped, weakly projecting beneath about one-fourth from base; median lobe thin, about two-thirds tegmen length, with proximal apodemes differentiated, proximal one-fourth filamentous; basal piece about one-third tegmen length.

#### Remarks.

This larger, convex species can be distinguished by the absence of the central part of the frontal stria, generally complete lateral submarginal pronotal stria (Fig. 76C), presence of apical fragments of 3^rd^, 4^th^ and 5^th^ elytral striae, absence of apical marginal elytral stria, fine postmetacoxal fovea (Fig. 76D), and pygidium lacking any interspersed coarse punctures (Fig. 76E). We restrict the type series to specimens from a relatively small part of northwestern Puntarenas, Costa Rica.

#### Etymology.

This species occurs along the Pacific coastal areas of Costa Rica, and is named accordingly.

### Operclipygus nitidus sp. n.

urn:lsid:zoobank.org:act:585171C0-A06D-4194-A2C6-6AE191C77596

http://species-id.net/wiki/Operclipygus_nitidus

Figs 76F–G 77F Map 27

#### Type locality.

PANAMA: Colón:San Lorenzo Forest [9°17'N, 79°58'W].

**Type material. Holotype male**: “**PANAMA: Colón Pr.,** San Lorenzo Forest. 9°17'N, 79°58'W. Flight intercept FIT-C1-13. 13–14 May 2004, A.Tishechkin. IBISCA’04”/ “LSAM0111145” (FMNH). **Paratypes** (16): 7: same data as type, except as noted: 1: 12–13.v.2004 (GBFM), 1: 14–15.v.2004 (GBFM), 1: 15–17.v.2004 (LSAM), 1: 19–20.v.1994 (AKTC), 1: 6–8.x.2003 (MSCC), 2: 24–25.x.2003 (LSAM, FMNH); **Panamá**: 1: Chepo-Carti Rd., 400m, vi.1982, FIT, B.D. Gill (BDGC); 1: Barro Colorado Isl., 9°11'N, 79°51'W, 10–17.vii.2000, FIT, S. Chatzimanolis (SEMC), 1: 3.vii.1994, FIT, D. Banks, 3: 8.vii.1994, FIT, D. Banks (SEMC), 1: 15.vii.1994, FIT, D. Banks; 1: Cerro Campana, Capira, 8°44'N, 79°57'W, 790m, 5.vi.1995, FIT, J. Ashe, R. Brooks (SEMC).

#### Other material.

**COSTA RICA: Limón:** 1: Area Cons. Tortuguero, Sector Cerro Cocori, Fca. De E. Rojas, 150m, x.1993, E. Rojas (INBIO); **Puntarenas:** 1: Res. Biol. Carara, Est. Quebrada Bonita, 100m, i.1995, R. Guzman (INBIO), 2: 50m, ix.1994, J.C. Saborio (INBIO), 1: vii.1994, R. Guzman (INBIO), 1: x.1994, J.C. Saborio, (INBIO), 1: xi.1994, R. Guzman, (INBIO), 1: 4–26.i.1993, R. Guzman, (INBIO); 4: Rancho Quemado, Peninsula de Osa, 200m, v.1991, J.C. Saborio (INBIO), 1: vi.1992, F. Quesada y M. Segura (INBIO), 1: viii.1991, F. Quesada (INBIO), 1: xii.1991, F. Quesada, (INBIO); 1: Est. Esquinas, Peninsula de Osa, 301400, 542200, x.1993, M. Segura, (INBIO); 1: Peninsula de Osa, 12–15.vii.1966, S. Peck (FMNH); 1: Parque Nac. Corcovado, Est Agujas, Golfito, C. Rincon, 745m, 13.ii.2000, FIT, A. Azofeifa (INBIO); 1: Reserva Forestal Golfo Dulce, Est. Agujas, Golfito, 250–350m, 16.v.2000, FIT, A. Azofeifa (INBIO); 1: Est. Biol. Las Cruces, Coto Brus, 8°47'N, 82°57'W, 100m, 8–10.iv.2002, FIT, A.K. Tishechkin (LSAM), 1: 2–8.iii.2002, FIT, A. Cline & A. Tishechkin (LSAM); 2: Est. Biol. Las Cruces, San Vito, 17.viii-12.ix.1982, FIT, B.D. Gill (BDGC); 1: Est. Biol. Las Alturas, Coto Brus, 8°57'N, 82°52'W, 1550m, 30.iii-3.iv.2002, FIT, A. Cline & A. Tishechkin (LSAM). **GUATEMALA: Zacapa**: 1: 3.5km SE La Union, 1500m, 25–27.vi.1993, FIT, J. Ashe & R. Brooks (SEMC). **PANAMA: Darién:** 1: Cana Biological Station, Serrania de Pirre, 7°45'18"N, 77°41'6"W, 1200m, 7–9.vi.1996, FIT, J. Ashe, R. Brooks (SEMC).

#### Diagnostic description.

This species is extremely similar to the preceding, *Operclipygus pacificus*, differing only in the following characters: length: 2.12–2.56 mm, width: 1.87–2.28 mm; frons more strongly depressed, with central portion of frontal stria present as small median arc; submarginal pronotal stria obsolete in basal half of lateral margin; elytra nearly always with short apical marginal stria or apical series of punctures; prosternum narrower, with carinal striae more clearly convergent anteriorly; 1^st^ abdominal ventrite with single lateral stria ending in small but distinct fovea behind inner corner of metacoxa, rarely continuing laterad behind coxa for short distance; pygidium with small punctures uniformly interspersed with fine, dense ground punctation. Male genitalia more or less indistinguishable from that of *Operclipygus pacificus* (see Figs 77A, C, H), except with S9 (Fig. 77F) more clearly desclerotized along midline, widened basally, with distinct, narrow basal emargination; tegmen widest just basad middle, weakly narrowing to apex, slightly thicker dorsoventrally; basal piece short, about one-fourth tegmen length.

#### Remarks.

This species and *Operclipygus pacificus* are very closely related, but differ consistently in several characters. This species always has the frons more deeply impressed (Fig. 76F) and has a small median piece of the frontal stria present. It also has very small punctures on the pygidium interspersed with the dense ground punctation (Fig. 76G), and has the prosternum less broad, with the carinal striae more distinctly converging to the front. The two species co-occur at Rancho Quemado (Osa Peninsula, Costa Rica), where they remain diagnosable. We restrict the type series to those specimens from Central Panama, due to evident variation in some superficial characters among localities.

#### Etymology.

This species’ name refers to its shining integument.

### Operclipygus subviridis sp. n.

urn:lsid:zoobank.org:act:079F7050-05B2-430F-BB3F-293B4177AB41

http://species-id.net/wiki/Operclipygus_subviridis

**Map 28.**
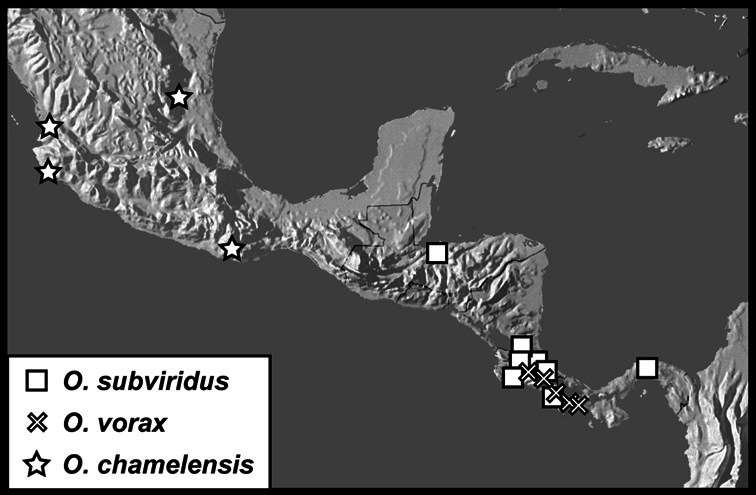
Records of the *Operclipygus impunctipennis* group.

Figs 77B, D, G, I 78A–B Map 28

#### Type locality.

COSTA RICA: Heredia: La Selva Biological Station [10°26'N, 84°01'W].

#### Type material.

**Holotype male**: “**Costa Rica:** Heredia, Est. Biol. La Selva. 10.26’[°]N 84.01’[°]W. F.I.T. 24 June 1998, C.Carlton & A.Tishechkin leg.” / “LSAM0046225” (FMNH). **Paratypes** (71): 54: same data as type, except as noted: 2: 19.vi.1998 (LSAM), 4: 21.vi.1998 (LSAM, MSCC, AKTC), 1: 22.vi.1998 (LSAM), 4: 23.vi.1998 (LSAM), 1: 24.vi.1998 (LSAM), 4: 25.vi.1998 (LSAM, FMNH), 3: 26.vi.1998 (LSAM), 1: 27.vi.1998 (LSAM), 3: 28.vi.1998 (LSAM), 1: 29.vi.1998 (LSAM), 1: i.1992, FIT, W. Bell (SEMC), 1: 25.i.1992 (SEMC), 2: 3.ii.1992 (SEMC), 2: 9.ii.1992 (SEMC), 3: 17.ii.1992 (SEMC), 9: 19.ii.1992 (SEMC), 1: 24.ii.1992 (SEMC), 2: 28.ii.1992 (SEMC), 2: 6.iii.1992 (SEMC), 2: 11.iii.1992 (SEMC), 1: 17.iii.1992 (SEMC), 2: 21.iii.1992 (SEMC), 1: 27.iii.1992 (SEMC), 1: 8.vi.2012, OTS Beetle Course, DNA Extract MSC-2303 (LSAM), 1: 21–28.iii.1988, W.E. Steiner, J.M. Hill, J.M. Swearingen, J.M. Mitchell (USNM); 3: Parque Nac. Braulio Carrillo, Est. El Ceibo, 400–600m, xi.1989, R. Aguilar & M. Zumbado, (INBIO); **Limón:** 2: Area Cons. Tortuguero, Sector Cerro Cocori, Fca. de E. Rojas, 150m, iii.1992, E. Rojas, (INBIO), 1: iii.1993 (INBIO), 1: ix.1991 (INBIO), 1: v.1993 (INBIO), 3: vi.1991 (INBIO), 1: xii.1992 (INBIO), 1: 10–30.ix.1992 (INBIO). **NICARAGUA: Rio San Juan**: 3: El Castillo loc. Bartola, iii.2000, I.G. Trezzi (CHFP, MEL); 1: Refugio Bartola, 60km SE San Carlos, 10°58.40'N, 84°20.30'W, 100m, 25–28.v.2002, FIT, R. Brooks, Z. Falin, S. Chatzimanolis (SEMC).

#### Other material.

**COSTA RICA: Guanacaste:** 1: Rio San Lorenzo, Tierras Morenas, Z.P. Tenorio, 1050m, ii.1993, C. Rodriguez, (INBIO), 1: ix.1995, G. Rodriguez, (INBIO); 1: Parque Nac. Guanacaste, 9km S Santa Cecilia, Est. Pitilla, 700m, viii.1994, C. Moraga, (INBIO), 1: x.1994, C. Moraga, (INBIO); **Cartago:** 1: Parque Nac. Barbilla, Turrialba, 2km despues del Rio Dantas, 400m, 17.xi.2000, 28.xi.2000, FIT, W. Arana (INBIO); 1: Parque Nac. Barbilla, Sendero El Felino, 1.5km SO de estqcion, 690m, 20.v.2001, 24.v.2001, W. Arana (INBIO); **Alajuela**, 3: Peñas Blancas, 800m, 19.v.1989, FIT, J. Ashe, R. Brooks, R. Leschen (SEMC); **Limón:** 1: Res Biol Hitoy Cerere, Est. Hitoy Cerere, Valle de la Estrella, 160m, 28.ix-11.x.1999, FIT, W. Arana (INBIO), 1: 100m, v–vi.1992, malaise trap, (INBIO); 1: R.B. Hitoy Cerere, Send. a Tepezcuintle, 100–200m, 30.iv-2.v.2002, pitfall, W. Arana (INBIO); 1: Amubri, 70m, 1–22.x.1994, G. Gallardo, (INBIO); **Puntarenas:** 1: Res. Biol. Carara, Est. Quebrada Bonita, 50m, xi.1994, R. Guzman, (INBIO); 1: Est. Biol. Las Cruces, Coto Brus, 8°47'N, 82°57'W, 1000m, 1–2.iv.2002, FIT, A. Cline & A. Tishechkin (LSAM); 1: Monteverde, 1240m, 21.v.1989, FIT, J. Ashe, R. Brooks, R. Leschen (CHSM). **HONDURAS: Cortés:** 1: Parque Nac. Cusuco, El Cortecito, 361198, 1717050, 1250m, 4–7.vi.2006, FIT, mature broadleaf forest, J. Nunez-Mino (OUMNH). **PANAMA: Panamá**: 2: Nusagandi, Ina Igar trail, 18–21.v.1993, pitfall, primary forest, E.G. Riley (TAMU); 1: Barro Colorado Isl., 9°11'N, 79°51'W, 40m, 25–30.vi.2000, FIT, S. Chatzimanolis (SEMC), 1: 14–18.vi.2000, FIT, S. Chatzimanolis (SEMC).

#### Diagnostic description.

Length: 2.06–2.56 mm, width: 1.81–2.31 mm; body rufobrunneus, generally with a very faint, dull greenish tinge, elongate oval, strongly convex; frons depressed at middle; frontal stria rounded at sides, broadly interrupted above antennal bases, with a short sinuate fragment at middle; supraorbital stria weak, detached from sides of frontal stria; labrum narrow, less than twice as wide as long, weakly emarginate at apex; left mandible untoothed, right with small, subacute basal tooth; pronotal disk weakly depressed basomedially, but lacking distinct prescutellar impression, with very fine, inconspicuous ground punctation and ~10 larger, shallow punctures at sides; marginal pronotal stria interrupted behind head; submarginal pronotal stria continuous across front and along anterior half of lateral margin, obsolete basally; elytron with two complete epipleural striae, outer subhumeral stria absent or represented as very short fragment in apical half, inner subhumeral stria absent, striae 1-2 complete, 3^rd^ stria present in basal third, and as short apical fragment, striae 4-5 represented by short apical fragments only, sutural stria present in apical three-fourths; elytral disk with crenulate, frequently fragmented apical marginal stria; prosternal keel broad, truncate at base, carinal striae converging almost straight to narrow anterior arch; meso- and metaventrite together convex; mesoventrite short, wide, not projecting anteriorly, marginal stria broadly interrupted; mesometaventral stria broadly arched forward to near mesoventral margin; lateral metaventral stria short, not extending past middle of ventrite; 1^st^ abdominal ventrite with single, abbreviated lateral stria, with small fovea behind inner corner of metacoxa; propygidium and pygidium with dense, fine, shallow ground punctation; propygidium with coarse basomedial punctures, sparser laterally and posteriorly; pygidium with few very small, interspersed coarse punctures; apical marginal stria absent. Male genitalia (Figs 77B, D, G, I): accessory sclerites absent; T8 short, sides subparallel in basal two-thirds, convergent to apex, apical emargination narrow, basal emargination deep, broad, nearly reaching basal membrane attachment line, ventrolateral apodemes separated beneath; S9 with sides subparallel to weakly convergent with narrowly rounded apices, ventral halves approximate at base, diverging weakly to apex; T9 subparallel in basal two-thirds, apices narrow; T10 with halves separate; S9 strongly narrowed at middle, with sides subparallel in basal fourth, base narrowly subtruncate, apex with very narrow median emargination, apical flanges interrupted; tegmen widest near base, very gradually narrowed to apex, with little dorsoventral curvature, medioventral process visible from above as a narrow ‘V’-shaped sclerotization, mostly contained in ventral membrane, very weakly projecting beneath; median lobe about half tegmen length, very thin; basal piece about one-third tegmen length.

#### Remarks.

**Figure 78.**
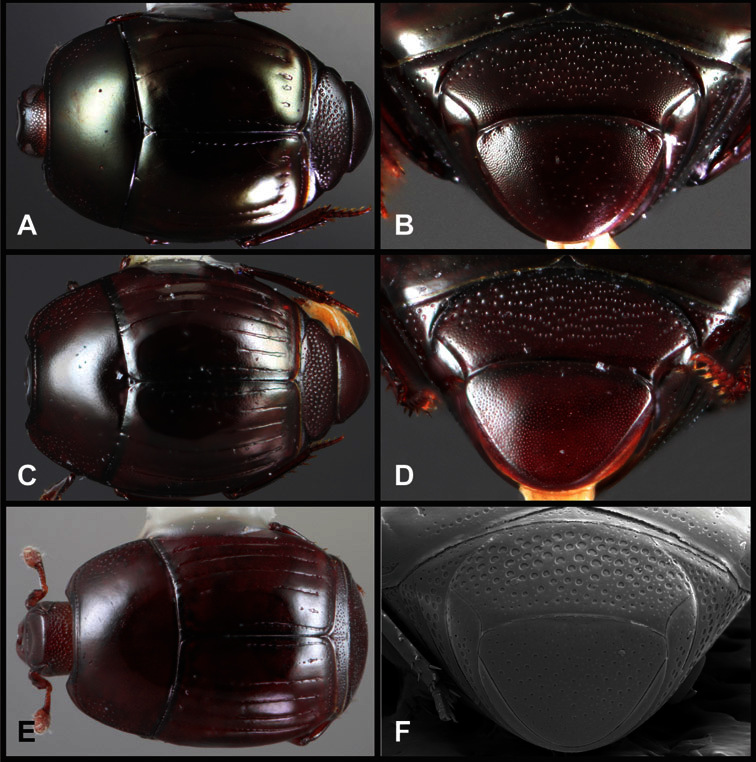
*Operclipygus impunctipennis* group. **A** Dorsal habitus of *Operclipygus subviridis* **B** Pygidia of *Operclipygus subviridis* **C** Dorsal habitus of *Operclipygus vorax* **D** Pygidia of *Operclipygus vorax* **E** Dorsal habitus of *Operclipygus chamelensis* **F** Pygidia of *Operclipygus chamelensis*.

This species is very similar to several in this group, having only two complete dorsal elytral striae, with the 3^rd^ stria present basally and apically, and the 4^th^ and 5^th^ present only as apical fragments (Fig. 78A). Although faint, the greenish metallic tinge of most individuals of this species will separate it from all others in this group. This species also frequently has a short outer subhumeral stria in the apical half, a strong apical marginal elytral stria, median part of the frontal stria present, a small (not minute) fovea behind the inner corner of the metacoxa, and small punctures sparsely interspersed with the dense ground punctation of the pygidium (Fig. 78B).

We limit the type series to specimens from northeastern Costa Rica and adjacent areas in Nicaragua.

#### Etymology.

The name of this species refers to its faint metallic greenish tinge.

### Operclipygus vorax sp. n.

urn:lsid:zoobank.org:act:550A79D8-36B0-4A07-88F6-CF8F30B824DA

http://species-id.net/wiki/Operclipygus_vorax

Figs 78C–D 79A, C, E, G Map 28

#### Type locality.

COSTA RICA: Puntarenas: Las Alturas Biological Station [8°57'N, 82°52'W].

#### Type material.

**Holotype male**: “**COSTA RICA**: Puntarenas, Coto Brus, Est. Biol. Las Alturas. 8°57'N, 82°52'W. 1,600m. F.I.T. #4. 5–8 Apr 2002, A.Tishechkin”/ “Caterino/Tishechkin Exosternini Voucher EXO-00316” (FMNH). **Paratypes** (53): 32: same data as type, except as noted: 5: 1550m, 25–30.iii.2002, FIT, A. Cline & A. Tishechkin (LSAM), 4: 1600m, 25–30.iii.2002 (LSAM), 9: 1600m, 30.iii-4.iv.2002 (LSAM, FMNH, INBIO, MSCC, AKTC), 1: 1550m, 30.iii-3.iv.2002, FIT, A.K. Tishechkin (LSAM), 1: 1600m, 3–4.iv.2002 (LSAM), 3: 1550m, 3–4.iv.2002 (LSAM), 1: 1550m, 4–5.iv.2002 (LSAM), 2: 1600m, 4–5.iv.2002 (LSAM), 6: 1600m, 5–8.iv.2002 (LSAM), 1: 08°56.17'N, 82°50.01'W, 1660m, 31.v-3.vi.2004, FIT, J.S. Ashe, Z. Falin, I. Hinojosa (SEMC); 2: Est. Biol. Las Cruces, Coto Brus, 8°47'N, 82°57'W, 100m, 8–10.iv.2002, FIT, A.K. Tishechkin (LSAM), 1: 22–31.iii.2002 (LSAM), 3: 25–30.iii.2002 (LSAM), 3: 2–8.iv.2002 (LSAM), 1: 8–10.iv.2002 (LSAM); 1: Parque Nac. Amistad, Est. Las Mellizas, Fca. Cafrosa, 1300m, iii.1990, M. Ramirez & G. Mora, (INBIO); 2: Altamira Biol. Sta., 09°01.76'N, 83°00.49'W, 1510–1600m, 4–7.vi.2004, FIT, J.S. Ashe, Z. Falin, I. Hinojosa (SEMC); 1: Monteverde, 1520m, 25.vi-2.vii.1983, FIT, D.H. Lindeman (CHSM); 1: 24.v.1989, FIT, J. Ashe, R. Brooks, R. Leschen (SEMC), 1: 21.ii-1.iii.1983, FIT, D. Lindemann (AKTC); 1: Est. La Casona, 1520m, 15–23.viii.1994, K.L. Martinez, (INBIO); 1: Res. Biol. Monteverde, Est. La Casona, 1520m, 5–10.x.1994, K. Martinez, (INBIO); 1: Est. Pittier, Send. Altamira, 1700–1760m, 10–20.v.1996, FIT con Carrona, A.M. Maroto, (INBIO); **San Jose**: 1: Cloudbridge Reserve, 2.4km ENE Sn Gerardo de Rivas, House Environs, 09°28.36'N, 83°34.51'W, 1700m, 8–11.vi.2004, FIT, J.S. Ashe, Z. Falin, I. Hinojosa (SEMC).

#### Other material.

**PANAMA: Chiriquí:** 3: 4km N Sta. Clara, Hartmann's Finca, 1500m, 13.vii.1982, FIT, B.D. Gill (BDGC); 2: 27.7km W. Volcan, Hartmann's Finca, 08°45'N, 82°48'W, 1450m, 14–17.vi.1995, FIT, J. Ashe & R. Brooks (SEMC); 1: 20km N. Gualaca, Finca La Suiza, 08°39'N, 82°12'W, 1350m, 24.v–9.vi.1995, FIT, J. Ashe & R. Brooks (SEMC); 1: Hornito, Finca La Suiza, 1220m, 6.vi.2000, FIT, H. & A. Howden (CMNC).

#### Diagnostic description.

**Figure 79.**
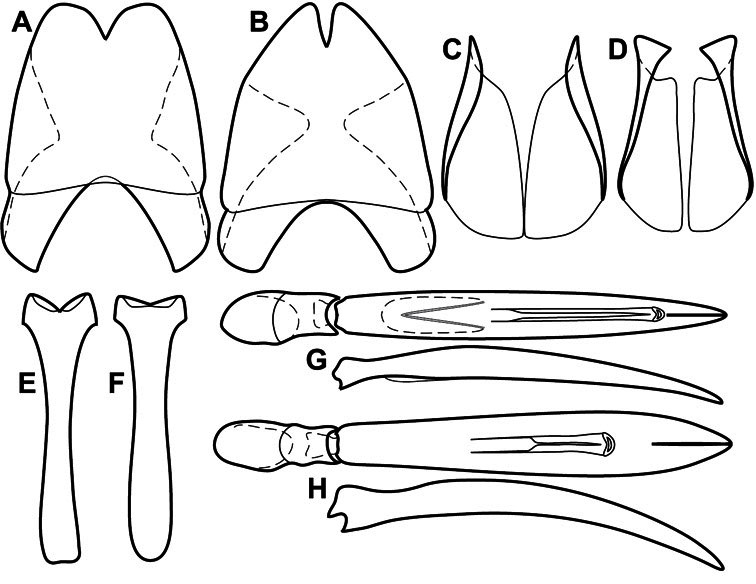
Male genitalia of *Operclipygus impunctipennis* group. **A** T8 of *Operclipygus vorax* **B** T8 of *Operclipygus chamelensis* **C** S8 of *Operclipygus vorax* **D** S8 of *Operclipygus chamelensis* **E** S9 of *Operclipygus vorax* **F** S9 of *Operclipygus chamelensis* **G** Aedeagus, dorsal and lateral views, of *Operclipygus vorax* **H** Aedeagus, dorsal and lateral views, of *Operclipygus chamelensis*.

Length: 2.12–2.53 mm, width: 1.84–2.18 mm; body rufobrunneus, elongate oval, sides distinctly rounded; frons and epistoma depressed at middle; frontal stria with sides rounded, interrupted over antennal bases, forming a detached arc at middle; labrum less than twice as wide as long, weakly emarginate at apex; left mandible untoothed, right with small, acute basal tooth; pronotal disk lacking prescutellar impression, with very fine, sparse ground punctation and ~18 coarse lateral punctures; marginal pronotal stria interrupted behind head; submarginal pronotal stria continuous across front of pronotum, extending along anterior two-thirds of lateral margin, obsolete at base; median pronotal gland openings situated about two-thirds pronotal length from anterior margin; elytron with two complete epipleural striae, inner and outer subhumeral striae absent, striae 1-3 complete (3^rd^ rarely interrupted), 4^th^ and 5^th^ striae present only in apical third, sutural present in apical three-fourths; elytral disk lacking apical marginal striae or punctures; prosternal keel and prosternal lobe with faint microsculpture; prosternal keel broad, weakly emarginate at base, carinal striae complete, convergent and nearly united along base, united anteriorly in broad arc; prosternal lobe with marginal stria close to margin, abbreviated at sides; mesoventral margin broadly projecting, marginal stria weak and variably fragmented at middle; mesometaventral stria strongly arched forward at middle to near anterior mesoventral margin; lateral metaventral stria extending toward inner third of metacoxa; 1^st^ abdominal ventrite with complete inner lateral stria and abbreviated outer lateral stria; postmetacoxal fovea absent; propygidium and pygidium with moderately dense, fine ground punctation; propygidium with coarser elongate punctures dense in basal two-thirds, obsolete apically; pygidium lacking coarser punctures; marginal pygidial stria absent. Male genitalia (Figs 79A, C, E, G): accessory sclerites absent; T8 short, sides subparallel in basal two-thirds, convergent to apex, apical emargination narrow, basal emargination deep, subangulate, intersecting basal membrane attachment line, ventrolateral apodemes widely separated beneath; S9 with sides convergent to narrow, subacute apices, ventral halves approximate at base, diverging weakly to apex; T9 with sides subparallel in basal two-thirds, apices narrow; T10 with halves separate; S9 narrowest at midpoint, slightly widened to rounded base, apex angulately emarginate, but without distinct median emargination, apical flange entire or narrowed medially; tegmen widest just basad middle, weakly narrowing to apex, rather thick dorsoventrally near base, narrowed to apex but not curved ventrad; medioventral process thin, contained in ventral membrane, not projecting beneath; basal piece about one-fourth tegmen length; median lobe about half tegmen length.

#### Remarks.

The basally abbreviated submarginal pronotal stria, usually complete elytral striae 1-3 (Fig. 78C), inconspicuous abdominal foveae, presence of two lateral striae on abdominal ventrite 1 (the outer abbreviated), and lack of coarse pygidial punctures or marginal pygidial stria (Fig. 78D) will distinguish this species. Specimens from Chiriquí, Panama generally have the 3^rd^ elytral stria interrupted, indicating some geographic variation, so they are excluded from the type series.

#### Etymology.

The name of this species means ‘voracious’.

### Operclipygus chamelensis sp. n.

urn:lsid:zoobank.org:act:545389AB-AECB-4281-AAC9-4F01B0431D11

http://species-id.net/wiki/Operclipygus_chamelensis

Figs 78E–F 79B, D, F, H Map 28

#### Type locality.

MEXICO: Jalisco: Chamela Biological Station [19°32'N, 105°02'W].

#### Type material.

**Holotype male**: “MEXICO:Jalisco, Chamela Biol. Stn., 16–20 July 1989, Robert W. Brooks #054, ex., flight interept trap”/ “Caterino/Tishechkin Exosternini Voucher EXO-00326” / “SEMC0903664 KUNHM-ENT” (SEMC). **Paratypes** (18): 11: same data as type (SEMC, FMNH, MSCC, AKTC); 7: same data but 14.vii.1989 (SEMC).

#### Other material.

**MEXICO: Nayarit**: 1: 1.viii.1953, under bark large woodchips, Bark tight-fitting chips; fairly fresh, mixed palm-hardwood grove, C.H. Seevers (FMNH); **Oaxaca**: 1: 4.7mi south San Gabriel Mixtepec, 16.vii.1985, J. Woolley & G. Zolnerowich (TAMU); **Tamaulipas**: 1: nr. Gomez Farias, Rancho del Cielo, 1000m, 6.vi-7.viii.1983, FIT, cloud forest, S. & J. Peck (CMNC).

#### Diagnostic description.

Length: 1.75–2.06 mm, width: 1.44–1.78 mm; body rufobrunneus, elongate oval; frons weakly impressed, with fine, inconspicuous ground punctation; sides of frontal stria rounded, interrupted over antennal bases, rarely complete, central portion sinuate; supraorbital stria weak, fragmented, detached from frontal stria; labrum about twice as wide as long, weakly asymmetrically emarginate; left mandible untoothed, right with small, subacute basal tooth; pronotal disk lacking prescutellar impression, ground punctation fine, sparse, with ~16 coarse lateral punctures; marginal and submarginal pronotal striae complete across front and sides; median pronotal gland openings about two-thirds pronotal length behind anterior margin; elytron with two complete epipleural striae, inner and outer subhumeral striae absent, striae 1-4 complete, 5^th^ stria present as short apical fragment, sutural present in posterior two-thirds; elytra with apical series of small punctures; prosternal keel weakly emarginate at base, carinal striae complete, united in narrow anterior arch; prosternal keel with marginal stria abbreviated at sides, narrowly interrupted at middle; mesoventral margin weakly projecting at middle, marginal stria broadly interrupted; mesometaventral stria broadly and strongly arched forward to mesoventral margin; lateral metaventral stria extending toward middle of metacoxa; 1^st^ abdominal ventrite with two lateral striae, both abbreviated, inner slightly longer than outer; postmetacoxal fovea absent; propygidium and pygidium lacking microsculpture, with fine, very sparse ground punctation; propygidium with medium, ocellate punctures separated by one-third their diameters in basal half, punctures smaller and sparser toward apex; pygidium with conspicuous coarse punctures uniformly separated by about 3× their diameters; marginal pygidial stria absent. Male genitalia (Figs 79B, D, F, H): accessory sclerites absent; T8 short, with sides weakly rounded, subparallel in basal half, convergent to apex, apical emargination narrow, shallow, basal emargination broadly rounded, deep, reaching to basal membrane attachment line, ventrolateral apodemes small, distant ventrally; S8 with sides narrowed to middle, divergent near apex, apical guides narrow throughout most of their length but widened abruptly near apex, ventral halves approximate at base, diverging slightly to apex; T9 with sides subparallel in basal half, convergent to apex, apices narrow, acute on inner corner; T10 with halves separate; S9 narrowest just distad middle, basal sides weakly rounded to narrow base, apex lacking median emargination, apical flange narrowed at middle but not interrupted; basolateral corners not prolonged proximad; tegmen narrow in basal fourth, widened to apical third, sides rounded, medioventral process very weakly sclerotized, indistinct; median lobe narrow, about one-half tegmen length, proximal apodemes differentiated at midpoint into thick and filamentous sections; basal piece about one-third tegmen length.

**Remarks.** This species is distinguished by its complete submarginal pronotal striae (Fig. 78E), complete elytral striae 1-4, row of apical elytral punctures, and absence of marginal pygidial stria (Fig. 78F). We assign a few specimens from other parts of Mexico to this species, although they show variation in a few characters, and should be reassessed in light of additional material.

#### Etymology.

This species’ name recognizes Estación de Biología Chamela, where most of the specimens of this species were collected.

### *Operclipygus marginellus* group

The name *Tribalister* Horn has been applied to two distinctive Nearctic species, *Tribalister marginellus* LeConte and *Tribalister striatellus* Fall. Although the two remain quite distinctive, combined data phylogenetic analyses clearly show both that they are deeply nested within *Operclipygus*, and that they have several Neotropical relatives that although superficially distinct, do share a few significant characters. Here we have synonymized *Tribalister* and recognize these species as a group within *Operclipygus*.

**Figure 80.**
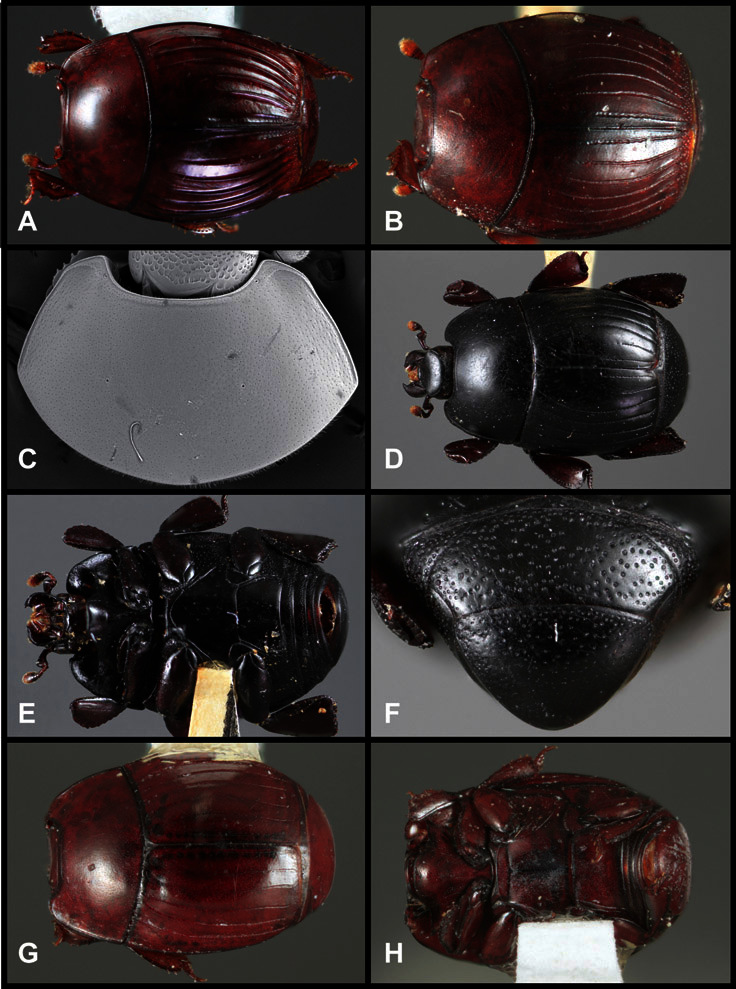
Various *Operclipygus marginellus* group species. **A** Dorsal habitus of *Operclipygus marginellus* **B** Dorsal habitus of *Operclipygus striatellus* **C** Pronotum of *Operclipygus striatellus* **D** Dorsal habitus of *Operclipygus formicatus* **E** Ventral habitus of *Operclipygus formicatus* **F** Pygidia of *Operclipygus formicatus* **G** Dorsal habitus of *Operclipygus hintoni* **H** Ventral habitus of *Operclipygus hintoni*.

The group is highly diverse in external morphology ranging from 2.5–8mm in size, with a great variety of external sculpturing. They all have the outer subhumeral elytral stria complete, and in most it forms a distinct lateral marginal carina of the elytron (Figs 80A–B, D, G). In addition the uppermost epipleural stria is frequently subcariniform as well. However, in a couple species the outer subhumeral stria is weakly or not at all cariniform. All the species have the lateral submarginal pronotal stria complete, very close to the margin, and sub- or fully carinate, such that the pronotal disk is depressed along its inner edge (Fig. 80B). Most retain the marginal pygidial sulcus, though it may be weak or absent. Some of these characters, particularly the exaggerated pronotal and elytral striae, are shared with members of the *Operclipygus dytiscoides* group, but species of the *marginellus* group never have the longitudinally ridged prosternal lobe that unites that group. In male genitalia they show few distinctive characters, though in those species where males are known, the apex of S9 is more or less entire, with the apical flange continuous (if narrowed) along the distal edge.

### Key to the species of the *Operclipygus marginellus* group

**Table:** 

1	Elytral striae deeply impressed to carinate, strongly convergent apically toward suture (Figs 80A–B); USA	2
–	Elytral striae distinct or not, never deeply impressed or carinate, at most weakly convergent apically; Mexico to South America	3
2	Elytral striae strongly carinate (Fig. 80A)	*Operclipygus marginellus* (J.E. LeConte)
–	Elytral striae deeply impressed but not carinate (Fig. 80B)	*Operclipygus striatellus* (Fall)
3	Elytral striae very weakly impressed; lateral marginal elytral carina strong; pronotum depressed near posterolateral corner	4
–	Elytral striae varied, impressed or not; lateral marginal elytral carina weaker; pronotum not depressed in posterolateral corners	6
4	Epistoma lacking oblique lateral carinae; left mandible with strong basal tooth (Fig. 84F)	*Operclipygus dentatus* sp. n.
–	Epistoma with oblique lateral carinae (Fig. 84G); left mandible not strongly toothed	5
5	Elytral striae very faintly impressed, 4^th^ and 5^th^ absent, 2^nd^ and 3^rd^ represented by scratch-like basal rudiments (Fig. 84A)	*Operclipygus selvorum* sp. n.
–	Elytral striae finely impressed, but distinct, 4^th^ and 5^th^ present in apical half, 2^nd^ and 3^rd^ complete	*Operclipygus ashei* sp. n.
6	Pygidium lacking marginal sulcus	*Operclipygus hintoni* sp. n.
–	Pygidium with marginal sulcus	7
7	Elytra coarsely punctate, elytral striae largely obliterated by punctures (Fig. 82A)	*Operclipygus orchidophilus* sp. n.
–	Elytra with only very fine, sparse ground punctation; elytral striae distinct and complete	8
8	Body very large (>7mm); legs broadly expanded, meso- and metatibiae subtriangular (Fig. 80E)	*Operclipygus formicatus* sp. n.
–	Body much smaller (<3mm) (Fig. 82E); tibiae narrow	*Operclipygus baylessae* sp. n.

### Operclipygus marginellus

(LeConte, 1860) comb. n.

http://species-id.net/wiki/Operclipygus_marginellus

**Map 29.**
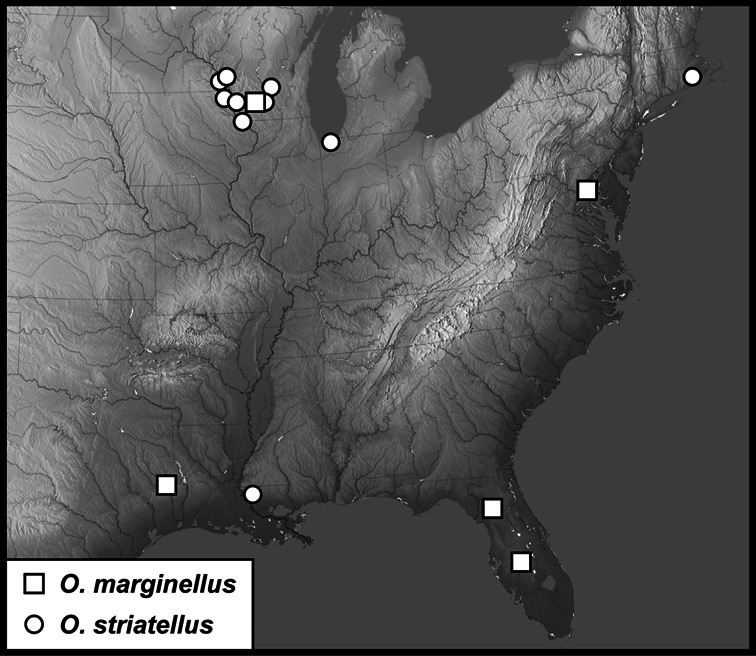
Records of the *Operclipygus marginellus* group.

Figs 80A 81A–D, G Map 29

Phelister marginellus J.E. LeConte 1860: 311; *Tribalister marginellus*: Horn 1873: 299.

#### Type locality.

Not specified beyond Maryland State, USA.

#### Type material.

**Lectotype**, herein designated: “Maryland”/ “*Phelister marginata* [sic]” / “*Tribalister marginellus* Horn (Lec.)” / “MCZ Type 35024” / “Aug.-Dec.2004 MCZ Image Database” / “LECTOTYPE *Phelister marginellus* LeC. M.S. Caterino & A.K. Tishechkin des. 2010” (MCZC). This species was described from an unspecified number of specimens, and the lectotype designation fixes primary type status on the only known original specimen.

#### Other material.

**USA: Florida**: 1: Levy Co., 4.0 mi. SW Archer, 29°30'10"N, 82°24'23"W, 19–26.iv.2000, P. Skelley, barrier pitfall trap (FSCA), 1: 13–25.vii.2000, 1: 18–21.v.1988, P. Skelley, window trap in rosemary, turkey oak sandhill (FSCA); 1: Alachua Co., 5.v.1993, R. Lundgren, flight barrier trap in hardwood hammock (CHPWK), 1: 18.vii.1994 (AKTC), 2: 18.vi.1995 (CHPWK); 1: Polk Co., Lake Marion Creek Estates, FIT, 26.x-9.xi.1999, R. Morris, FIT (AKTC). **Texas**: 1: Angelina Co., Angelina NF, ~3mi. NE Rockland, 31°3'19"N, 94°22'06"W, 19.ix-2.x.1996, Clarke, Menard, Riley, pitfall, unmanaged longleaf pine (TAMU). **Wisconsin**: 1: Sauk Co., Spring Green Preserve SNA, 43°11'58"N, 90°03'32"W, 12.vii.2003, J.P. Gruber, in nest *Aphaenogaster* sp., under rock on bluff prarie (CHJG), 1: 17.vi.2000, attached to leg of live *Aphaenogaster treatae* Forel, under board in sandy oak barren (CHJG), 1: 19–30.v.2001, J.P. Gruber, FIT, 1: 30.v-16.vi.2001, J.P. Gruber, FIT (CHJG).

#### Diagnostic description.

**Figure 81.**
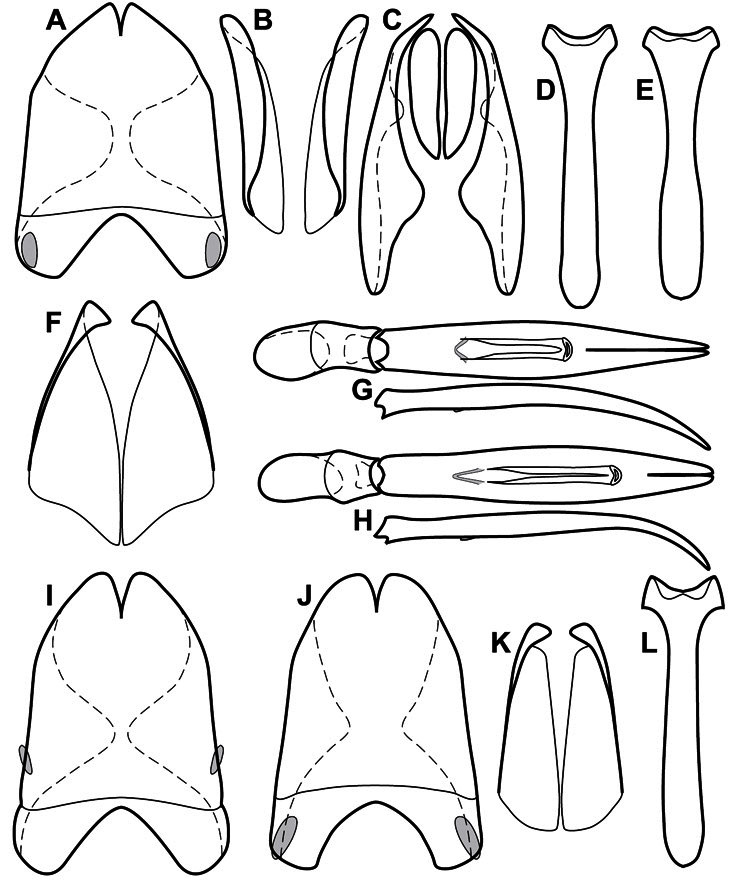
Male genitalia of *Operclipygus marginellus* group species. **A** T8 of *Operclipygus marginellus* **B** S8 of *Operclipygus marginellus* **C** T9 & T10 of *Operclipygus marginellus* **D** S9 of *Operclipygus marginellus* **E** S9 of *Operclipygus striatellus* **F** S8 of *Operclipygus striatellus* **G** Aedeagus, dorsal and lateral views, of *Operclipygus marginellus* **H** Aedeagus, dorsal and lateral views, of *Operclipygus hintoni* **I** T8 of *Operclipygus striatellus* **J** T8 of *Operclipygus hintoni* **K** S8 of *Operclipygus hintoni* **L** S9 of *Operclipygus hintoni*.

Length: 1.68–1.75 mm, width: 1.50–1.55 mm; body rufescent, broadly rounded, strongly convex, with very fine granulate microsculpture on most surfaces; frons broad, weakly depressed at middle, sides of frontal stria rounded, curving evenly, transverse across front; supraorbital stria complete, connected to sides of frontal stria; epistoma weakly depressed, slightly elevated at sides, weakly emarginate apically; labrum about 2.5× as wide as long, flat, weakly emarginate apically; left mandible with broad, blunt basal tooth, right with slightly smaller, subacute tooth; pronotum lacking prescutellar impression, with only very faint, shallow lateral punctures; marginal pronotal stria continuous along all edges; lateral submarginal pronotal stria complete, close to margin, subcarinate, continuous with anterior submarginal stria, pronotal disk depressed along their inner edges; median pronotal gland openings faintly annulate, located about two-thirds pronotal distance behind anterior margin; elytron with two complete, crenulate epipleural striae, outer subhumeral stria carinate, forming lateral elytral margin continuous with lateral pronotal margin, inner subhumeral stria absent, dorsal striae 1-5 complete, rather broadly depressed, with outer edge of stria strongly carinate, sutural stria obsolete in basal third, similarly deeply impressed, all striae converging toward midline apically; elytral disk with numerous coarse punctures near apical margin; prosternal keel truncate at base, with carinal striae complete, divergent and free basally and apically; prosternal lobe very short, with marginal stria weak to obsolete; mesoventrite broadly and shallowly emarginate along anterior margin, with marginal stria very close to margin, weak at middle; mesometaventral stria broadly arched forward to anterior mesoventral margin, meeting inner corner of mesocoxa at side, continued by lateral metaventral stria which extends laterally, parallel to postmesocoxal stria, strongly abbreviated; metaventrite moderately convex; 1^st^ abdominal ventrite with two lateral striae, depressed along complete inner lateral stria, outer stria obsolete basally; propygidium uniformly and densely covered with coarse punctures; pygidium with fewer and sparser punctures, mainly at sides, impunctate toward apex; marginal pygidial sulcus well impressed along most of margin, obsolete toward base. Male genitalia (Figs 81A–D, G): accessory sclerites present; T8 short, with sides subparallel in basal two-thirds, abruptly narrowed to apex, apical emargination narrow, basal emargination nearly reaching basal membrane attachment line, ventrolateral apodemes evenly developed to base and apex, nearly meeting along midline; S8 elongate, subparallel, with apical guides well developed throughout most of length, ventral edges divergent to apex; T9 with sides subparallel in basal third, convergent to apex, apices narrow, opposing; T10 with halves fully separate; S9 broadened in basal half, weakly rounded at base, lacking apical emargination, apical flange entire; tegmen narrow, with sides rounded, widest just basad midpoint, ventromedial projection weak, ‘U’-shaped, barely projecting beneath about one-fourth from base; median lobe about one-third tegmen length, proximal apodemes not obviously differentiated; basal piece almost one-half tegmen length.

#### Remarks.

The two Nearctic species of the *Operclipygus marginellus* group are easily distinguished. *Operclipygus marginellus* has distinctly carinate elytral striae (Fig. 80A), whereas those of *Operclipygus striatellus* are merely deeply impressed (Fig. 80B).

### Operclipygus striatellus

(Fall, 1917) comb. n.

http://species-id.net/wiki/Operclipygus_striatellus

Figs 80B–C 81E–F, I Map 29

Tribalister striatellus Fall, 1917: 165.

#### Type locality.

Not specified beyond Rhode Island State, USA.

#### Type material.

**Holotype**: “R.I.”/ “N.B.X. p.31” / “TYPE *striatellus*” / “H. C. FALL COLLECTION” / “M.C.Z. Type 24127” / “Aug.-Dec.2004 MCZ Image Database” (MCZC), examined 2012.

#### Other material.

**USA: Iowa**: 1: Allamakee Co., Fish Farm Mounds Wildlife Area, 43°27'23"N, 91°16'43"W, 11.vi.2005, J.P. Gruber, in nest *Aphaenogaster* sp., under rock on bluff prarie (CHJG); 1: Dubuque Co., Kaufmann Avenue Prarie, 42°30'54"N, 90°40'40"W, 16.vi.2003, J.P. Gruber, in nest *Aphaenogaster* sp., under rock on bluff prarie (CHJG). **Indiana**: 1: Porter Co., Indiana Dunes N. L., Inland Marsh, 15.ix-18.x.1995, R. Grundel, unbaited pitfall trap (CHPWK). **Minnesota**: 1: Houston Co., Mound Prarie SNA, 43°45'54"N, 91°25'51"W, 11.vi.2003, J.P. Gruber, in nest of *Aphaenogaster* sp., under rock on bluff prarie; (CHJG) 2: Mound Prarie SNA, 43°46'00"N, 91°25'27"W, 11.vi.2003, J.P. Gruber, in nest *Aphaenogaster* sp., under rock on bluff prarie (CHJG). **Wisconsin**: 1: Columbia Co., Rocky Run Ck. SFA, 43°27'38"N, 89°19'31"W, 21.vi.2009, J.P. Gruber, beaten from *Quercus* sp. (CHJG), 1: 21.vi.2009, J.P. Gruber, in nest *Aphaenogaster* sp., under wood on ground, hardwood forest/savanna (CHJG), 1: 23.vii.2008, J.P. Gruber, in nest *Aphaenogaster* sp., hardwood forest/savanna (CHJG); 1: Crawford Co., Hogback Prarie SNA, 43°13'03"N, 90°52'36"W, 16.vi.2001, J.P. Gruber, in nest *Aphaenogaster* sp., under rock on bluff prarie (CHJG), 1: 23.vi.2002, J.P. Gruber, being carried by worker *Aphaenogaster* sp.; 43°12'50"N, 90°52'19"W (CHJG), 1: 17.VI.2005, J. Gruber & P. Kovarik, *Aphenogaster* sp. nest (CHPWK); Crawford Co., Rush Creek SNA, 43°22'04"N, 91°08'15"W, 11.vi.2005, J.P. Gruber, in nest *Aphaenogaster* sp., under rock on bluff prarie (CHJG); 1: Monroe Co., Ft. McCoy Sand Quarry, T17N R3W sec2, 18.vi.1999, J.P. Gruber, in sand in mammal burrow opening, prarie/oak savanna (CHJG); 1: Trempealeau Co., Brady's Bluff Prarie SNA, 44°00'57"N, 91°28'34"W, 11.vi.2003, J.P. Gruber, in nest *Aphaenogaster* sp., under rock on bluff prarie (CHJG); 3: Grant Co., Dewey Heights Prarie SNA, 42°44'02"N, 91°01'02"W, 9.vi.2003, J.P. Gruber, in nest *Aphaenogaster* sp., under rock on bluff prarie (CHJG, MSCC: DNA Extract MSC-0132); 1: Sauk Co., Spring Green Preserve SNA, 43°12'22"N, 90°03'32"W, 22.vii.2012, J.P. Gruber, in nest *Aphaenogaster* sp., under rock on bluff prarie (CHJG). **Louisiana**: 1: West Feliciana Parish, Feliciana Preserve, 30°47'N, 91°15'W, 18.v.1999, J. Fassbender, Berlese, forest litter, 1: 1–29.iv.2001, A. Cline, FIT (AKTC), 3: 29.iv–24.v.2001, A.R. Cline, FIT (AKTC, LSAM), 1: 24.v.-2.vi.2001, A.R. Cline, FIT (LSAM), 2: 2–15.vi.2001, A.R. Cline & A.K. Tishechkin, FIT (AKTC, LSAM), 1: 7.vii.–22.ix.2001, A.R. Cline & A.K. Tishechkin, FIT (LSAM), 1: 7.iii.–23.v.2004, A.K. Tishechkin, FIT (LSAM); 1: West Feliciana Parish, Tunica Hills WMA West of Weyanoke, 3.v.1985, C. Barr (LSAM). **CANADA: 1: Ontario** Mississauga, 22.vi.1979, A. Francoeur (Bousquet and Laplante 2005).

#### Diagnostic description.

Length: 1.56–1.93 mm, width: 1.34–1.62 mm; body rufescent, elongate oval, with rather conspicuous ground punctation and sparse patches of granulate microsculpture; frons broad, depressed at middle, sides of frontal stria rounded, slightly sinuate above antennal bases, complete, weakly arcuate dorsad across front; apical margin of epistoma weakly emarginate; labrum about 2.5× as wide as long, weakly emarginate apically; left mandible bluntly produced along inner edge, right mandible with more discrete, acute tooth; pronotum lacking prescutellar impression or lateral punctures; marginal pronotal stria continuous along lateral and anterior edges; lateral submarginal pronotal stria complete, close to margin, subcarinate, continuous with anterior submarginal stria, pronotal disk depressed along their inner edges; median pronotal gland openings distinctly annulate, located about two-thirds pronotal distance behind anterior margin; elytron with two complete epipleural striae, outer subhumeral stria weakly carinate, forming weak lateral elytral margin continuous with lateral pronotal margin, inner subhumeral stria absent, dorsal striae 1-5 complete, faintly carinate along their outer edges, sutural stria obsolete in basal fourth, all striae converging toward midline apically; elytral disk with numerous coarse punctures near apical margin; prosternal keel truncate at base, carinal striae complete, divergent basally, free basally and apically; prosternal lobe very short, marginal stria weak; mesoventrite truncate along anterior margin, with marginal stria very close to margin; mesometaventral stria broadly arched forward to anterior mesoventral margin, continued by lateral metaventral stria which extends laterally, close and parallel to postmesocoxal stria, strongly abbreviated at midpoint; metaventrite moderately convex; 1^st^ abdominal ventrite depressed, especially at sides along complete inner lateral stria; propygidium uniformly and densely covered with coarse punctures; pygidium with fewer and sparser punctures, mainly at sides, impunctate toward apex; marginal pygidial sulcus well impressed along most of margin, obsolete toward base. Male genitalia extremely similar to those of *Operclipygus marginellus* (see Fig. 81), differing as follows: T8 (Fig. 81I) slightly more elongate, evenly narrowed to apex; S8 (Fig. 81F) shorter, sides convergent to apex, with narrow apical guides developed only near apex; S9 (Fig. 81E) with base more broadly expanded; aedeagus indistinguishable.

#### Remarks.

See diagnosis under *Operclipygus marginellus*, above.

### Operclipygus formicatus sp. n.

urn:lsid:zoobank.org:act:3AD7D621-1684-4143-9A30-C1D81A02EBDB

http://species-id.net/wiki/Operclipygus_formicatus

Figs 80D–F Map 30

#### Type locality.

BRAZIL: Minas Gerais:Mar de Hespanha [21°52'S, 43°0.5'W].

#### Type material.

**Holotype female**: “Mar de Hespanha, Min. Ger., Brasil, A. Heyne, BerlinW.”/ “*Discoscelis formicata* Lewis Type” [nomen nudum] / “Caterino/Tishechkin Exosternini Voucher EXO-00006” (BMNH).

#### Diagnostic description.

Length: 5.67 mm, width: 4.66 mm; body piceous, elongate oval, strongly convex; frons broad, sides of frontal stria divergent between eyes, angulate mediad above antennal bases, arcuate dorsad at middle; supraorbital stria fine, narrowly detached from sides of frontal stria; epistoma depressed at middle, slightly elevated at sides, truncate apically; labrum rather narrow, apically arcuate; antennal club small, barely twice as long as antennomere 8, only sparsely tomentose on dorsal and ventral bases; mandibles broad, left mandible lacking basal tooth, right mandible with small, blunt (worn?) basal tooth; pronotum with faint, punctiform prescutellar impression, disk impunctate, with only inconspicuous ground punctation; marginal pronotal stria complete along lateral and anterior edges; lateral submarginal stria complete along side, bent inward at front, narrowly detached from anterior submarginal stria which is very finely impressed, faintly recurved posterad at sides; median pronotal gland openings located behind ends of anterior submarginal stria, about one-fourth pronotal length from anterior margin; elytron with two complete epipleural striae, inner and outer subhumeral striae complete, simple, dorsal striae 1-4 complete, all extended mediad along basal margin as fine striae, 5^th^ stria present only in apical fourth, sutural stria present in apical half; elytral disk lacking apical punctures; prosternal keel weakly emarginate at base, narrow, with carinal striae complete, weakly divergent, free basally and apically; secondary carinal striae present, parallel to basal half of carinal striae; prosternal lobe truncate apically, with complete marginal stria diverging straight posterad at sides, nearly meeting lateral prosternal striae; mesoventrite narrow, anterior margin very weakly produced, with complete marginal stria; metaventrite narrowly depressed along midline, impunctate; mesometaventral stria subangulately arched forward, extending to about one-fourth behind anterior mesoventral margin, continued by lateral metaventral stria posterolaterad to outer third of metacoxa; 1^st^ abdominal ventrite with one complete lateral stria; all tibiae broadly expanded, prothoracic tibiae with outer edge evenly arcuate, with fine marginal teeth, meso- and metatibiae approximately triangular, with apical width equal to about three-fourths tibial length, each bearing fine marginal spines; meso- and metatarsi short, retractable into poorly developed apical tibial grooves; propygidium and pygidium with ground punctation very fine, each bearing irregularly sparse, small round punctures; marginal sulcus of pygidium fine, complete. Male: not known.

#### Remarks.

This species is unmistakeable in its large size (Fig. 80D) and broadly expanded tibiae (Fig. 80E). If its position within *Operclipygus* was not so well supported, it might easily justify its own genus. Analyses indicate that it is probably the sister group to the remaining members of the *Operclipygus marginellus* group. Lewis's unpublished intention to place this species in *Discoscelis* Schmidt, an Haeteriine genus, is based purely on some superficial similarities, primarily in gross body form and the greatly expanded legs. Perhaps the reason it was never published is that he recognized the error.

**Map 30.**
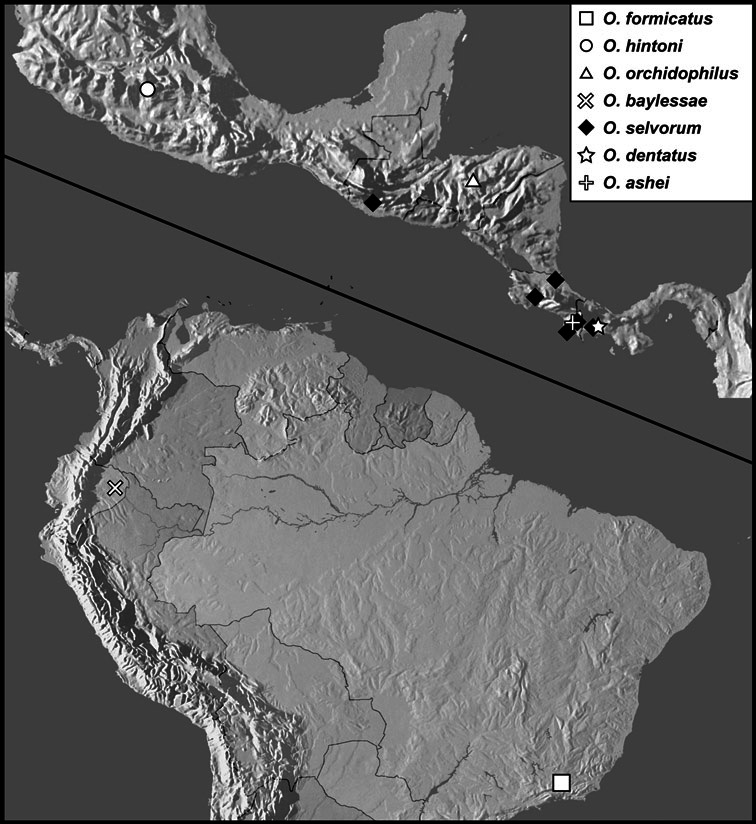
Records of the *Operclipygus marginellus* group.

#### Etymology.

We honor Lewis's suggested specific name for this species, which might indicate some knowledge of its myrmecophily that is not reflected on any of the specimen's labels. Its true habits remain unknown.

### Operclipygus hintoni sp. n.

urn:lsid:zoobank.org:act:570D475F-8F23-44C9-A45F-AEB596BBD272

http://species-id.net/wiki/Operclipygus_hintoni

Figs 80G–H 81H, J–L Map 30

#### Type locality.

MEXICO: Mexico: Real de Arriba [21°2.5'N, 100°0.2'W].

#### Type material.

**Holotype male**: “Real de Arriba, Temescaltepec, Mex.”/ “1934 B.M. 1959-100” / “H.E. Hinton, R.L. Usinger, Collectors” / “Caterino/Tishechkin Exosternini Voucher EXO-00177” (BMNH).

#### Diagnostic description.

Length: 2.40 mm, width: 2.00 mm; body rufobrunneus, elongate oval; frons broad, depressed at middle, with sides of frontal stria weakly divergent between eyes, sinuate over antennal bases, weakly arcuate dorsad at middle; supraorbital stria concealed in type; epistoma wide, depressed, with fine lateral marginal striae, shallowly emarginate apically; labrum about twice as wide as long, weakly emarginate apically; left mandible with blunt basal tooth, right with smaller, subacute basal tooth; pronotum lacking prescutellar impression, disk with fine but conspicuous ground punctation, lacking coarse lateral punctures; marginal pronotal stria complete along lateral and anterior edges; lateral submarginal stria complete along side, close to margin, with marginal bead narrowly elevated, pronotal disk depressed along inner edge, especially toward front, curved posteromediad at anterior pronotal angle, not meeting anterior submarginal stria, which is fine, faintly recurved posterad at sides; median pronotal gland openings located behind ends of anterior submarginal stria, about one-third pronotal length behind anterior margin; elytron with two complete epipleural striae, outer subhumeral stria complete, forming weak but distinct lateral elytral margin, inner subhumeral stria absent, dorsal striae 1-5 complete, carinate along their outer edges, 2-5 with basal ends curving inward along basal margin, sutural stria obsolete in basal fourth; elytral disk with series of apical marginal punctures; prosternal keel weakly produced at base, carinal striae complete, divergent, separate at base, close and parallel in anterior two-thirds, connected apically; prosternal lobe bluntly rounded apically, marginal stria very fine, obsolete at sides; mesoventrite wide, apical margin shallowly emarginate, marginal stria fine, narrowly interrupted at middle; mesometaventral stria present as a broad, more or less transverse arch between mesocoxae, mesometaventral suture depressed near coxa; metaventral stria present from mesocoxal depression posterolaterad toward metepisternum, continuous with short recurrent stria along metaventral-metepisternal suture; 1^st^ abdominal ventrite with two complete lateral striae; all tibiae weakly expanded, submarginal ridge of the meso- and metatibiae forming a nearly continuous carina parallel to margin; ground punctation of propygidium and pygidium very fine, inconspicuous, propygidium with very faint, shallow, though moderately wide, secondary punctures, pygidium lacking secondary punctures; marginal pygidial sulcus absent. Male genitalia (Fig. 81H, J–L) very similar to that of *Operclipygus marginellus*, differing mainly as follows: T8 broader and longer, with sides weakly convergent in basal two-thirds; S8 with sides weakly convergent to apex, apical guides narrower, abruptly widened near apex; S9 broader in basal two-thirds; tegmen widest near midpoint, not as strongly narrowed to apex, rather strongly and abruptly curved ventrad in apical third.

#### Remarks.

This species is quite isolated, and lacking a pygidial sulcus, was not immediately recognized as an *Operclipygus*. However, several other external and genitalic characters support its position here. Among Mexican Exosternini it is unmistakeable in its combination of subcarinate outer subhumeral and dorsal elytral striae (Fig. 80G), its fine lateral epistomal striae, and the unusual form of the mesometaventral stria (Fig. 80H) and associated lateral mesoventral depressions.

#### Etymology.

This species’ name honors Howard E. Hinton (1912–1977), recognizing his collection of the type specimen, as well as his many contributions to our knowledge of Neotropical Exosternini.

### Operclipygus orchidophilus sp. n.

urn:lsid:zoobank.org:act:E24F73F3-103F-41DF-B3C9-892AC8B9D443

http://species-id.net/wiki/Operclipygus_orchidophilus

Figs 82A–D Map 30

#### Type locality.

Honduras, but not known for certain (see below).

#### Type material.

**Holotype female**: “with Orchid plants, Honduras, 1.30 ‘[19]40, EQ A 68278”/ “Caterino/Tishechkin Exosternini Voucher EXO-00178” (USNM)**.**

#### Diagnostic description.

Length: 2.62 mm, width: 2.18 mm; body rufobrunneus, elongate oval, with very conspicuous ground punctation, with scattered coarser punctures, particularly on elytra; frons broad, shallowly depressed at middle, with strong ground punctation and granulate microsculpture; sides of frontal stria subparallel between eyes, curving anteromediad onto sides of epistoma, absent from central part of frons; supraorbital stria absent; anterior margin of epistoma weakly emarginate; labrum about 2.5× as wide as long, narrowed to more or less truncate apex; left mandible untoothed, right with small, acute basal tooth; pronotum with fine, sublinear prescutellar impression, disk with strong ground punctation at middle, finer along sides where granulate microsculpture is more evident; marginal pronotal stria complete along lateral and anterior margins; lateral submarginal pronotal stria subcarinate, more or less complete along side, very close to marginal stria, merging with it in anterior one-fifth, pronotal disk narrowly depressed along inner edge of stria; anterior submarginal stria weak, present for short distance behind middle third of head; median pronotal gland openings located laterad ends of anterior submarginal stria, about 8 puncture widths behind anterior margin; anterolateral pronotal gland openings not evident; elytron with two complete epipleural striae, uppermost distinctly carinate; outer subhumeral stria complete, strongly carinate, forming lateral elytral marginal carina; inner subhumeral stria carinate, converging to apex of inner subhumeral stria; 1^st^ dorsal stria carinate, impressed only in apical third, also converging to apex of subhumeral striae, basally barely detectable, scratchlike; dorsal striae 2-3 faintly visible as fine basal rudiments, 4^th^ stria represented only by very short basal arch connected to base of sutural stria, 5^th^ stria as series of punctures extending most of elytral length, sutural more or less complete, strongly fragmented toward apex; elytral disk with strong ground punctation and numerous coarser punctures intermingled, more densely toward apex; prosternal keel truncate at base, carinal striae complete, divergent basally, subparallel in anterior two-thirds, not joined apically; prosternal lobe moderately short, subtruncate, lacking marginal stria; meso- and metaventrite with fine but conspicuous ground punctation and distinct granulate microsculpture; mesoventrite with anterior margin emarginate, marginal stria fine, complete; mesometaventral stria forming broad arch, extending to near anterior mesoventral margin, meeting mesocoxa at sides; lateral metaventral stria originating at mesocoxa, extending posterolaterad to metepisternum; 1^st^ abdominal ventrite with single complete lateral stria; ventrites 2-4 with irregular transverse stria along posterior margin; all legs, especially tibiae, moderately broadly expanded, the apical width of the meso- and metatibiae slightly more than one-half their lengths, their edges with spines reduced to obsolete; propygidial and pygidial disks with fine ground punctation, rather coarse secondary punctation, and conspicuous, granulate microsculpture; propygidium with lateral marginal striae; marginal sulcus of pygidium complete and well-impressed. Male not known.

#### Remarks.

**Figure 82.**
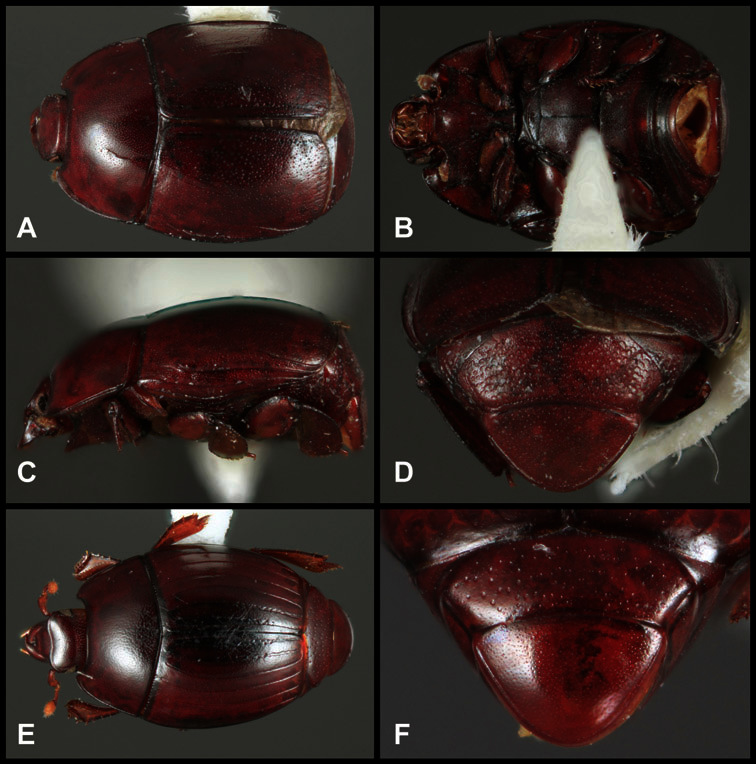
Various *Operclipygus marginellus* group species. **A** Dorsal habitus of *Operclipygus orchidophilus* **B** Ventral habitus of *Operclipygus orchidophilus* **C** Lateral habitus of *Operclipygus orchidophilus* **D** Pygidia of *Operclipygus orchidophilus* **E **Dorsal habitus of *Operclipygus baylessae* **F** Pygidia of *Operclipygus baylessae*.

This species is unique in many characters, particularly the pattern of elytral striation (Fig. 82A), with most dorsal striae reduced to short, scratch-like rudiments, and the moderately expanded tibiae (Fig. 82C). The lateral epistomal striae and lateral propygidial striae (Fig. 82D) are also highly distinctive. Its expanded tibiae may reflect a relationship with *Operclipygus formicatus*. The type locality, as vague as it is, must also be regarded as tentative (D. Furth, personal communication).

#### Etymology.

This species is named for its apparent association with orchids, as it has only been collected during an inspection of plant material being imported into the U.S. from Honduras.

### Operclipygus baylessae sp. n.

urn:lsid:zoobank.org:act:87F9D860-19CF-4AD4-8444-C68119B31662

http://species-id.net/wiki/Operclipygus_baylessae

Figs 82E–F 83A–E Map 30

#### Type locality.

ECUADOR: Orellana: Yasuní Research Station [0°40.5'S, 76°24'W].

#### Type material.

**Holotype male**: “**Ecuador:** Napo, mid.Rio Tiputini, Yasuní Res. Stn. 0°40.5'S, 76°24'W, FIT#M2 20–29 Jun 1999. CEC#030 C.Carlton & V.Moseley” / “LSAM 0013234” / “LSAM0045734” (FMNH).

#### Diagnostic description.

**Figure 83.**
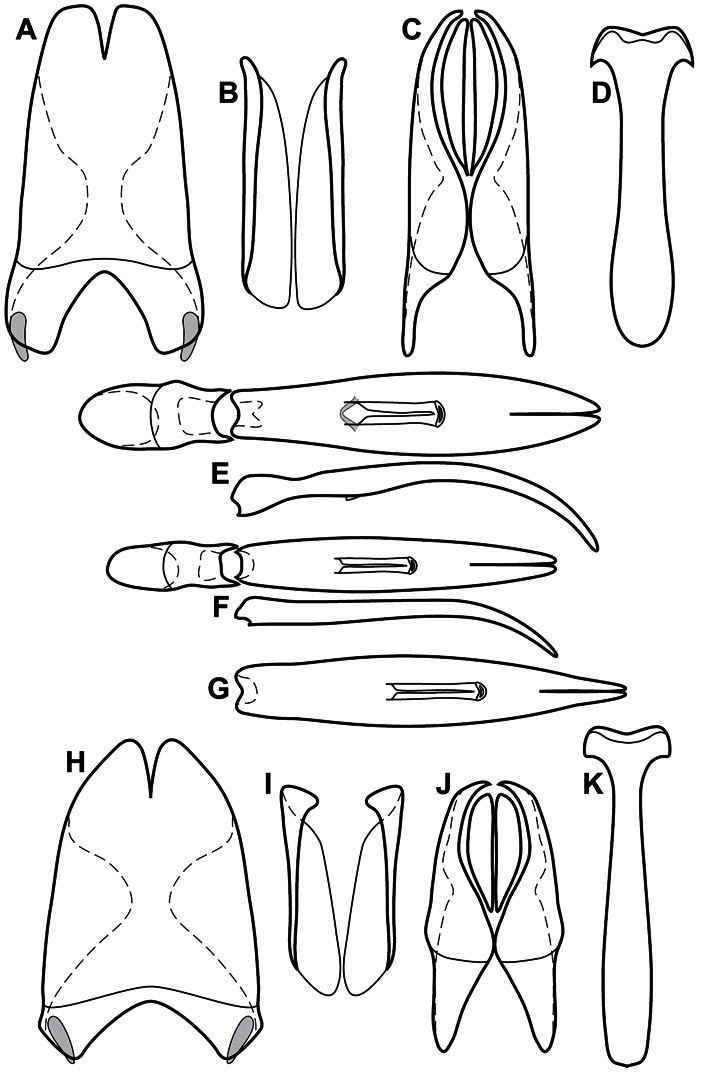
Male genitalia of *Operclipygus marginellus* group species. **A** T8 of *Operclipygus baylessae* **B** S8 of *Operclipygus baylessae* **D** S9 of *Operclipygus baylessae* **C** T9 & T10 of *Operclipygus baylessae* **E** Aedeagus, dorsal and lateral views, of *Operclipygus baylessae* **F** Aedeagus, dorsal and lateral views, of *Operclipygus selvorum* **G** Aedeagus, dorsal view, of *Operclipygus ashei* **H** T8 of *Operclipygus selvorum* **I** S8 of *Operclipygus selvorum* **J** T9 & T10 of *Operclipygus selvorum* **K** S9 of *Operclipygus selvorum*.

Length: 1.93 mm, width: 1.62 mm; body rufobrunneus, elongate oval, subdepressed, generally smooth, but with conspicuous pronotal ground punctation; frons weakly depressed at middle, with conspicuous ground punctation and very faint granulate microsculpture; frontal stria rounded at sides, slightly sinuate across front; epistoma mostly flat, weakly emarginate at apex; labrum about twice as wide as long, rounded at sides, weakly emarginate apically; left mandible with weak, blunt basal tooth, right mandible concealed in type; pronotum lacking discrete prescutellar impression, but with ground punctation coarser and denser toward prescutellar region; pronotal disk lacking coarse lateral punctures, but with faint granulate microsculpture throughout; marginal pronotal stria broadly interrupted behind head; lateral submarginal pronotal stria complete along side, close to margin, weakly carinate, barely curved inward in front; anterior submarginal pronotal stria absent; median pronotal gland openings behind eye on each side, about 8 puncture widths behind anterior pronotal margin; elytron with single epipleural stria, outer subhumeral stria complete, subcarinate, forming weak lateral elytral margin, inner subhumeral stria absent, dorsal striae 1-5 complete, sutural stria complete and distinctly widened toward base; elytral interstriae with very faint granulate microsculpture; prosternal keel weakly produced at base, carinal striae divergent posteriorly, slightly shortened in front, meeting in narrow anterior arch; prosternal lobe rounded, marginal stria complete; anterior margin of mesoventrite broadly emarginate, marginal stria complete; mesometaventral stria narrowly arched forward to about mesoventral midpoint, angulate near mesocoxa, continued by lateral metaventral stria to near metacoxal midpoint; 1^st^ abdominal ventrite with two complete lateral striae; propygidium and pygidium with fine ground punctation, propygidium with very sparse, small secondary punctures, pygidium lacking secondary punctures; marginal pygidial sulcus fine, complete. Male genitalia (Fig. 83A–E): accessory sclerites present; T8 with sides evenly convergent to apex, apical emargination narrow, basal emargination subtriangular, not reaching basal membrane attachment line, ventrolateral apodemes most strongly developed basally, narrowed to apex, nearly meeting at midline; S8 elongate, sides subparallel, apical guides evenly developed throughout length, ventral halves separate, weakly divergent to apex; T9 with sides subparallel in basal half, weakly convergent to apex, apices narrow, abruptly inturned; T10 with halves separate; S9 broad, sides subparallel in basal half, base rounded, apex lacking median emargination, apical flange continuous though weakly narrowed medially, basolateral corners weakly prolonged; tegmen narrow, with sides rounded, widest near midpoint, ventromedial projection weak, ‘U’-shaped, barely projecting beneath about one-third from base; median lobe about one-third tegmen length, proximal apodemes not obviously differentiated; basal piece almost one-half tegmen length.

#### Remarks.

The placement of this species in the *Operclipygus marginellus* group follows from the phylogenetic results, and several characters support it. However, it is unusual in numerous characters too, and deserves further investigation. It may be easily recognized by the presence of a complete, fine pygidial sulcus (Fig. 82H), the complete elytral striae (Fig. 82E; minus inner subhumeral), especially the characteristically basally widened sutural stria, and by the increased density of pronotal punctures in the prescutellar region.

#### Etymology.

We name this species in honor of Victoria Moseley Bayless, curator of the Lousiana State Arthropod Museum and co-collector of the unique type of this species.

### Operclipygus selvorum

sp. n.

urn:lsid:zoobank.org:act:8634281C-0E23-487C-B5EF-D395AE311262

http://species-id.net/wiki/Operclipygus_selvorum

Figs 83F, H–K 84A–D Map 30

#### Type locality.

COSTA RICA: Heredia: La SelvaBiological Station **[**10°26'N, 84°01'W].

#### Type material.

**Holotype male**: “**COSTA RICA**: Heredia, Est. Biol. LaSelva. 10.26’[sic]N 84.01'W. F.I.T.22June 1998 C.Carlton & A. Tishechkin” / “LSAM 0046258” (FMNH). **Paratypes** (17): 4: same data as type, except as noted: 1: 19.vi.1998 (LSAM), 1: 26.vi.1998 (LSAM), 2: vii-viii.1998, N. Franz (AKTC, MSCC); **Puntarenas:** 1: Las Cruces Biol. Sta. 1330m, 08°47.14'N, 82°57.58'W, 28–31.v.2004, FIT, J.S. Ashe, Z.Falin, I.Hinojosa. (SEMC); 1: Rancho Quemado, Peninsula de Osa, 200m, v.1991, F. Quesada, (INBIO), 1: v.1992 (INBIO), 1: vii.1992 (INBIO); 1: Res. Biol. Carara, Est. Quebrada Bonita, 50m, iv.1994, J. Saborio, (INBIO), 1: v.1994, J. Saborio (INBIO); **Limón:** 1: Area Cons. Tortuguero, Sector Cerro Cocori, Fca. de E. Rojas, 150m, vi.1993, E. Rojas, (INBIO), 1: 12–31.viii.1994 (INBIO), 1: iii.1993 (INBIO), 1: ix.1991 (INBIO); **Alajuela**: 1: Peñas Blancas, 880m, 19.v.1989, FIT, J. Ashe, R. Brooks, R. Leschen. **PANAMA: Chiriquí**: 1: Cerro Hornio, 15 km NE Gualaca, viii.1982, 1200m, FIT, B.Gill (BDGC). **GUATEMALA: Suchitepéquez**: 1: 4km S Volcan Atitlan, 14.55103°N, 91.19350°W, 1750m, 15.vi.2009, LLAMA, DNA Extract MSC-2156 (SBMNH).

#### Diagnostic description.

Length: 1.62–2.00 mm, width: 1.40–1.75 mm; body rufobrunneus, smooth, dully shining, elongate oval, moderately depressed; frons and epistoma broad, depressed between strongly carinate frontal striae which descend onto the epistoma, not crossing the anterior frontal margin; labrum about three times as wide as long, weakly emarginate apically; left mandible with large, blunt basal tooth, right with much smaller, subacute tooth; pronotum lacking prescutellar impression, disk broadly depressed in posterolateral corners, with very fine ground punctation, lacking coarse lateral punctures; marginal pronotal stria complete along lateral and anterior margins; lateral submarginal pronotal stria subcarinate, more or less complete along side, very close to marginal stria, merging with it in anterior one-fifth, pronotal disk narrowly depressed along inner edge of stria; anterior submarginal stria weak, present for short distance behind middle third of head, parallel and/or connected to anterior marginal stria at ends; median pronotal gland openings faintly annulate, located about two-thirds pronotal length from anterior pronotal margin; elytron with two complete epipleural striae, the uppermost distinctly carinate; outer subhumeral stria complete, strongly carinate, forming lateral elytral marginal carina; inner subhumeral stria absent, dorsal stria 1 very fine, scratch-like, present in apical two-thirds, dorsal striae 2-3 very fine, scratch-like, variably present in basal half, striae 4-5 absent, sutural stria fine, present in apical half; elytral disk depressed in anterolateral corner, in common with pronotal depression; elytron lacking apical marginal punctures; prosternal keel truncate to weakly emarginate at base, carinal striae complete, divergent and separate basally and apically; most of venter with conspicuous waves of microsculpture; prosternal lobe short, wide, subtruncate apically, lacking marginal stria; anterior margin of mesoventrite weakly outwardly produced, marginal stria complete; mesometaventral stria present as a broad, more or less transverse arch between mesocoxae, mesometaventral suture depressed near coxa; metaventral stria extending posterad from mesocoxal depression curving laterad toward metepisternum, continuous with short recurrent stria along metaventral-metepisternal suture; 1^st^ abdominal ventrite with inner lateral stria more or less complete, outer stria rudimentary; all tibiae weakly expanded, submarginal ridge of the meso- and metatibiae forming a weak, continuous carina parallel to margin; ground punctation of propygidium and pygidium very fine, inconspicuous, both lacking secondary punctures; propygidium with lateral striae; marginal pygidial sulcus fine, complete. Male genitalia (Figs 83F, H–K): accessory sclerites present; T8 with sides weakly convergent in basal two-thirds, strongly convergent in apical third, apical emargination narrow, basal emargination broad, deep, basal membrane attachment line distad emargination by about one-third its depth, ventrolateral apodemes most strongly developed near middle, present only along middle of segment; S8 with sides subparallel, apical guides well developed throughout length, gradually wider toward apex, ventral edges subparallel, but well separated; T9, S9 and T10 indistinguishable from those of *Operclipygus marginellus*; tegmen with shape as in *Operclipygus marginellus*, but with medioventral process extremely weak, hardly evident, not projecting beneath.

#### Remarks.

**Figure 84.**
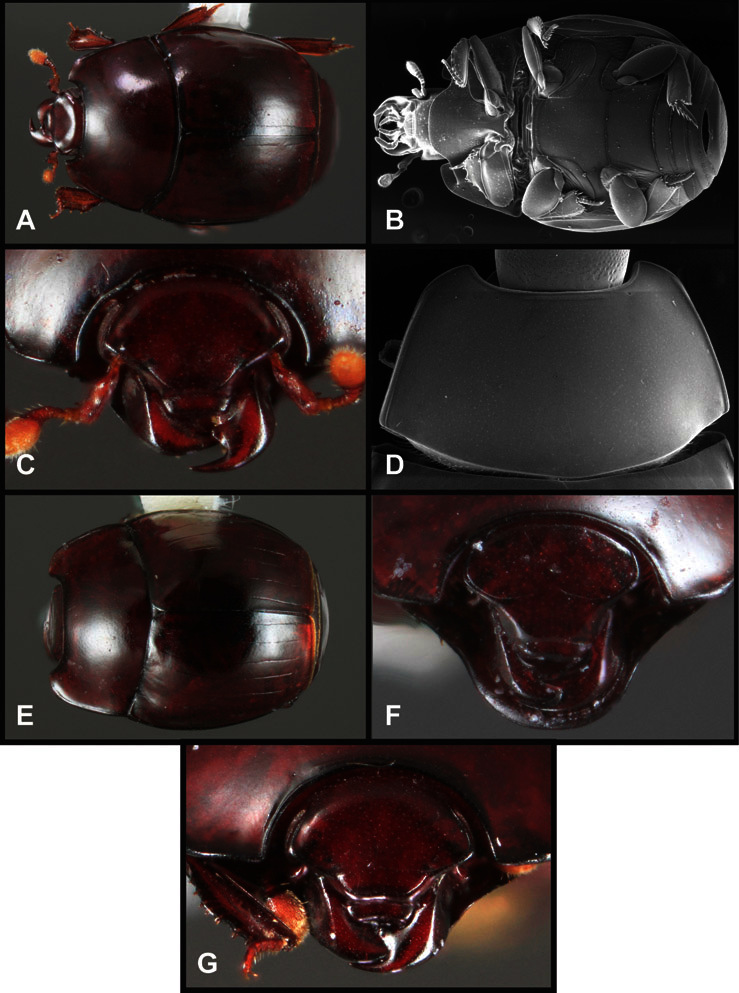
Various *Operclipygus marginellus* group species. **A** Dorsal habitus of *Operclipygus selvorum* **B** Ventral habitus of *Operclipygus selvorum* **C** Frons of *Operclipygus selvorum* **D** Pronotum of *Operclipygus selvorum* **E** Dorsal habitus of *Operclipygus dentatus* **F** Frons of *Operclipygus dentatus* **G** Frons of *Operclipygus ashei*.

This species and the following two represent a very closely related trio, united by their unique posterolateral pronotal depressions (Fig. 84D). Among the three, *Operclipygus selvorum* is unique in its almost completely obsolete elytral striae (Fig. 84A).

#### Etymology.

This species is named for its type locality, the popular and incredibly diverse Estación Biológica La Selva, in Herédia, Costa Rica.

### Operclipygus dentatus sp. n.

urn:lsid:zoobank.org:act:31425622-2C05-4E10-A112-2B29D6C6E874

http://species-id.net/wiki/Operclipygus_dentatus

Figs 84E–F Map 30

#### Type locality.

PANAMA: Chiriquí: La Fortuna Station [08°46'N, 82°12'W].

#### Type material.

**Holotype male**: “PANAMA: Chiriquí Prov. La Fortuna, “Cont. Divide Trail”, 08°46'N, 82°12'W, 1100m, 21–23 May 1995 J. & A. Ashe #044, ex: flight intercept trap” / “SEMC0903608” (SEMC).

#### Other material.

**COSTA RICA: Guanacaste:** 1: Parque. Nac. Guanacaste, Sector Las Pailas. 6–26.vi.1994 (INBIO).

#### Diagnostic description.

This species is very similar to the preceding, differing mainly in the following characters: length: 1.68–1.72 mm, width: 1.53–1.56 mm; body form broader, sides not as strongly rounded; pronotal sides almost subparallel, elytra slightly rounded at sides; frons flat, not at all depressed; frontal stria not carinate, finely impressed, evenly rounded at sides onto front, recurved dorsad along anterior margin of frons, interrupted at middle, not extending onto epistoma; both mandibles very strongly and acutely dentate; anterior submarginal pronotal stria absent; pronotal disk more strongly depressed in posterolateral corners; all dorsal elytral striae fine but distinct, striae 1-4 complete, 5^th^ stria present in apical three-fourths, sutural present in apical two-thirds; prosternal lobe larger, more broadly rounded apically; venter with vestigial or no microsculpture; marginal mesoventral stria absent; meso- and metatibiae with submarginal spines, not forming a submarginal ridge; propygidium and pygidium completely lacking marginal striae. Male genitalia are extremely similar to those of the preceding species (see Figs 83F, H–K), differing principally in the more strongly narrowed S8, with apical guides that are distinctly wider near the apices.

#### Remarks.

This species is very closely related to both the preceding and following species, but differs radically in the simple form of the frontal striae, being not at all carinate (Fig. 84F). The specimen from Guanacaste, Costa Rica is similar in most respects, but differs slightly in elytral striation, and has a complete frontal stria, not interrupted medially. We consider it conspecific, but exclude it from the type series.

#### Etymology.

This species is named for the very strong teeth on the mandibles.

### Operclipygus ashei sp. n.

urn:lsid:zoobank.org:act:77891E8F-F464-495F-AFB1-9510FBCA8E93

http://species-id.net/wiki/Operclipygus_ashei

Figs 83G 84G Map 30

#### Type locality.

COSTA RICA: Puntarenas: Las Cruces Biological Station [8°47'N, 82°57'W].

#### Type material.

**Holotype male**: “**COSTA RICA**: Puntarenas Prov. Las Cruces Biol. Sta. 1330m, 08°47.14'N, 82°57.58'W, 28–31-V-2004. J.S. Ashe, Z.Falin, I.Hinojosa. Ex: flight intercept trap. CR1AFH04 060”/ “SM0621562 KUNHM-ENT” (INBIO). **Paratype** (1): **COSTA RICA: Puntarenas:** Coto Brus, Est. Biol. Las Cruces, 8°17'N, 82°57'W, 1100m, 8–20.vi.2005, FIT, M. Ferro (LSAM).

#### Diagnostic description.

This species is very similar to *Operclipygus selvorum*, differing mainly in the following characters: length: 2.31–2.50 mm, width: 1.97–2.06 mm; body slightly broader, subquadrate; frons and epistoma depressed at middle; frontal stria carinate at sides, continued onto epistoma as lateral carinae, but also complete across the anterior margin of the frons as a simple stria; all dorsal elytral striae fine but distinct, striae 1-3 complete, 4^th^ barely abbreviated at base, 5^th^ present in apical half, sutural present in apical two-thirds; prosternal lobe larger, more broadly rounded, with fine marginal stria which is obsolete at sides; propygidium with a few secondary punctures along basal margin. Male genitalia: segments 8–10 very similar to those of *Operclipygus selvorum*, and indistinguishable from that of *Operclipygus dentatus* (see Figs 83F, H–K and preceding description); tegmen (Fig. 83G) longer, with slightly more arcuate sides.

#### Remarks.

This species falls right between the preceding two morphologically, having the strong frontal carina of *Operclipygus selvorum* (Fig. 84G), but the fine, more or less complete elytral striae of *Operclipygus dentatus* (see Fig. 84E). The combination of frontal carinae that descend onto the epistoma along with a fine frontal stria on the frons is unique in this species.

#### Etymology.

We name this species in honor of the late James ʽsteve’ Ashe (1947–2005), formerly of the University of Kansas, in recognition of his exceptionally productive fieldwork in Central America.

##### *Operclipygus*, incertae sedis

The following 27 species are not assigned to any species group. Most are rather generalized, but lack obvious similarities to species in other recognized groups. The majority of these have a detached anterior submarginal pronotal stria, and median pronotal gland openings only slightly displaced posterad the anterior pronotal margin. These characters might appear to ally them with species in the *Operclipygus panamensis*, *Operclipygus kerga*, *Operclipygus sejunctus*, *Operclipygus impuncticollis*, *Operclipygus hospes*, or *Operclipygus dubius* groups. But this condition is likely to be a symplesiomorphy shared by all of these, and it seems more likely that these *incertae sedis* species form a grade out of which some of the more distinctive groups have arisen. More detailed phylogenetic analyses focusing on the species of *Operclipygus* should be able to resolve some of these relationships more satisfactorily.

The last two species treated in this section, *Operclipygus angustisternus* and *Operclipygus shorti*, do not fit this picture. In fact, based on external characters they might have been assigned to the *Operclipygus fossipygus* group, as they are both relatively large, convex species with well developed pygidial sulci and strongly displaced median pronotal gland openings. However, both lack the highly specialized and distinctive male genitalia of members of the *Operclipygus fossipygus* group. It is possible that one or both are its sister group, but that requires much further exploration.

### Operclipygus teapensis

(Marseul, 1853) comb. n.

http://species-id.net/wiki/Operclipygus_teapensis

**Figure 85.**
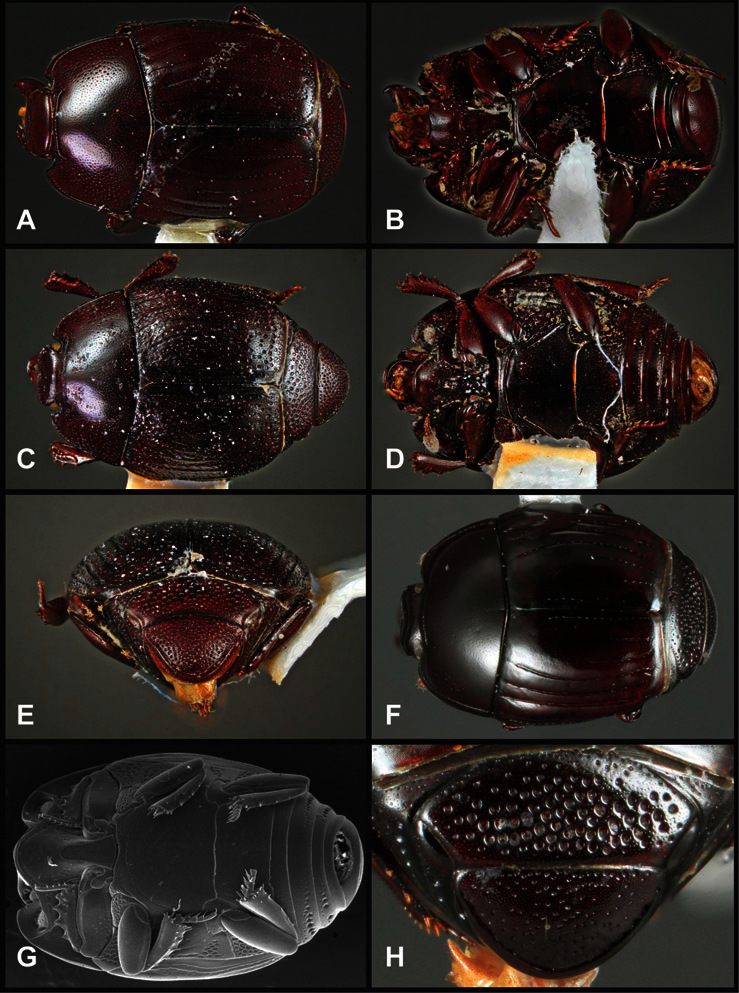
Various *Operclipygus* species. **A** Dorsal habitus of *Operclipygus teapensis* **B** Ventral habitus of *Operclipygus teapensis* **C** Dorsal habitus of *Operclipygus punctulatus* **D** Ventral habitus of *Operclipygus punctulatus* **E** Pygidia of *Operclipygus punctulatus* **F **Dorsal habitus of *Operclipygus lama* **G** Ventral habitus of *Operclipygus lama* **H** Pygidia of *Operclipygus lama*.

**Map 31.**
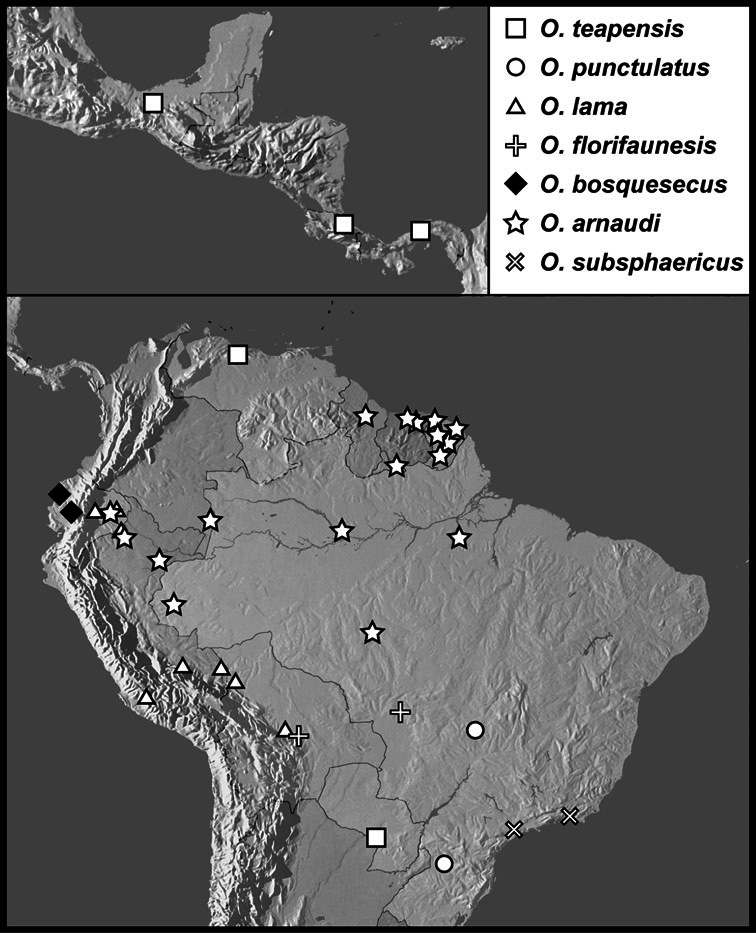
Records of the *Operclipygus* incertae sedis spp.

Figs 85A–B 86A–E Map 31

Phelister teapensis Marseul, 1853: 482.

#### Type locality.

MEXICO: Tabasco: Teapa [17°33'N, 92°57'W].

#### Type material.

**Lectotype male**, hereby designated: “14 *Phelister teapensis* M. Teapa Pilat [illegible: ?Bournt?]” / “Museum Paris, Coll. de Marseul 2842-90” / “TYPE” / [handwritten label on which nothing is legible] / “LECTOTYPE *Phelister teapensis* Marseul, 1853 M.S.Caterino & A.K.Tishechkin des. 2010” (MNHN). This species was described from an unspecified number of specimens, and the lectotype designation fixes primary type status on the only known original specimen.

#### Other material.

**COSTA RICA: Limón:** 1: Hamburg farm, Reventazon, Ebene, Dung of bat, F. Nevermann, 25.iii.1928 (FMNH), 2: 26.vii.1931 (FMNH), 7: 28.ix.1928 (USNM); 1: Hamburg Farm, Siquirres, E. Reimoser (FMNH). **PANAMA: Panamá**: 1: Rio Chilibrillo, Bat Caves, 29.ix.1923, bat cave, Zetek, Molino & Shannon (USNM); 1:Summit, CZ, 9.i.1941, Dung of *Dirias* & *Molossus*, K.W. Cooper (FMNH). **PARAGUAY**: **Chaco**: 3: Fiebrig (FMNH). **VENEZUELA: Yaracuy**: 4: Minas de Aroa Tunel Polvorin, 20.xii.1998, on guano, H. Escalona (MSCC).

#### Diagnostic description.

**Figure 86.**
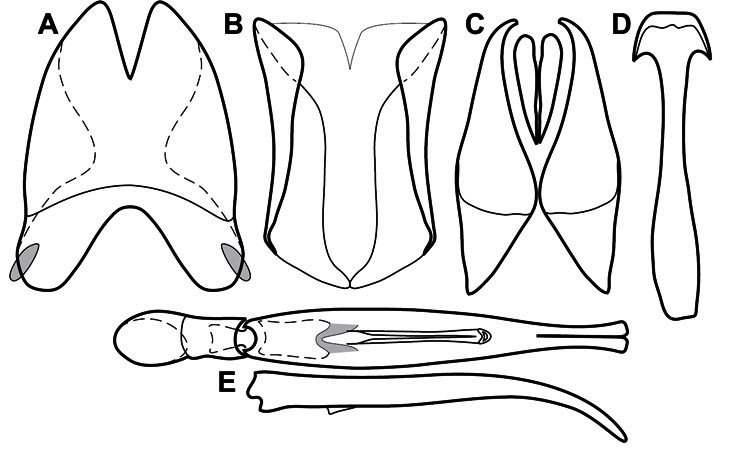
Male genitalia of *Operclipygus teapensis*. **A** T8 **B** S8 **C** T9 & T10 **D** S9 **E** Aedeagus, dorsal and lateral views.

Length: 1.90–2.68 mm, width: 1.47–2.06 mm; body rufo-brunneus, elongate oval, widest at humeri, prothorax markedly narrower, sides nearly parallel; head with frons broad, flat to weakly depressed; frontal striae parallel between eyes, finely impressed, sinuate in front, complete or very narrowly interrupted at sides, continuous with complete supraorbital stria; labrum about half as long as wide, shallowly emarginate; pronotum with linear prescutellar impression, about equal in length to scutellum; lateral marginal pronotal stria continuous along sides and front, narrowly interrupted behind head in some individuals; lateral submarginal stria complete, turning inward, ending freely at front; anterior submarginal stria close to margin, crenulate, diverging from margin at sides; median pronotal gland openings inconspicuous; pronotal disk with some coarser punctures at sides, varying in number and density; elytron with two complete epipleural striae; outer subhumeral stria complete, inner weak, present in apical half in some individuals, varying to absent; dorsal striae 1-4 complete, 5^th^ and sutural striae subequal, present in apical two-thirds; prosternal keel truncate at base, carinal striae complete, sinuate, meeting in arch near presternum; secondary carinal striae variably present between procoxae and carinal striae; prosternal lobe generally strongly reflexed, somewhat long and narrow, its marginal stria curving away from margin toward presternal suture at sides; mesoventrite truncate in front, with complete marginal stria; mesometaventral stria bent forward in a blunt angle, nearly to marginal stria, meeting lateral metaventral stria at a small angulation near mesocoxa, extending toward middle of metacoxa; 1^st^ abdominal ventrite with two lateral striae close together, outer stria curving behind metacoxa; propygidium short, with small punctures separated by about twice their diameters intermixed with fine ground punctures; pygidium with much smaller coarse punctures amid ground punctation; pygidum rarely with fragments of apical marginal stria (mostly in Venezuelan specimens, among material available). Male genitalia (Figs 86A–E; see discussion of variation under ‘Remarks’, below): accessory sclerites present; T8 rather short, with sides rounded, basal emargination narrow, angulate, basal membrane attachment line just distad its apex, apical emargination deep, narrow, ventrolateral apodemes small, widest near base, distant at ventral midline; S8 with apical guides widest just basad apices, narrowing gradually to base, halves fused just at base, diverging then parallel to near apex; T9 with apices narrowly rounded, ventrolateral tooth large; T10 with halves small, barely separated along midline; S9 well sclerotized in apical two-thirds, narrow in apical half, asymmetrically expanded to truncate base, apex with lateral flanges strongly elevated, slightly converging to uninterrupted apical flange; tegmen widest near base, sides rounded, narrowed in apical half, widened to apices, apex curved moderately downward; medioventral process rather weakly sclerotized, ‘U’-shaped, projecting beneath tegmen about one-fourth from base; median lobe slender, about half tegmen length; basal piece less than one-third tegmen length.

#### Remarks.

This is a rather distinctive species based on external morphology, with a subquadrate body form (Fig. 85A), relatively strong ground punctation on most surfaces, a complete outer subhumeral stria, a detached but not strongly recurved anterior submarginal pronotal stria, and angulate mesometaventral stria (Fig. 85B). However, at the same time there is significant geographic variation across its range. Material from Venezuela tends to be more densely punctate than most. However, its genitalia are identical to those from Central American specimens. Three specimens from Paraguay cannot be separated from Central American specimens based on external characters, but have moderately distinctive genitalia, with an aedeagus that is shorter and wider apically than the rest. Given the sparse available specimens from South America, we keep all these together under a single name for the present.

Ranging from southern Mexico to Paraguay, this species spans a surprisingly broad geographic range for one with such apparently specialized habits. Unusually, most specimens have ecological data, and it appears that an association with bat guano in caves or other bat roosts is the primary niche. One label, on a Panamanian specimen, is even more specific, indicating dung of *Dirias* Miller and *Molossus* Geoffroy (common Neotropical bat genera).

### Operclipygus punctulatus sp. n.

urn:lsid:zoobank.org:act:43362453-BCE3-4E30-95F9-17968B3F2FEF

http://species-id.net/wiki/Operclipygus_punctulatus

Figs 85C–E Map 31

#### Type locality.

BRAZIL: Santa Catarina: Nova Teutonia [27°11'S, 52°23'W].

#### Type material.

**Holotype female**: “Nova Teutonia, Sta. Catharina, BRAZ. II:--:79, Fritz Plaumann leg.” / “runs to *dubitabilis*” [R.L. Wenzel's handwriting] / “*Operclipygus* n. sp.!!” / “♀” / “FMNH-INS 0000069128” (FMNH). **Paratypes** (2): 1: same locality as type, xi.1971 (MHNG); **BRAZIL: Goias**, Jatai, x.1972, M. Alvarenga (UFPR).

#### Diagnostic description.

Length: 2.18–2.34 mm, width: 2.00–2.12 mm; body rufo-piceous, strongly rounded, widest behind humeri, sides evenly rounded to front and rear, most surfaces coarsely and conspicuously punctate; frons with sides rounded, shallowly depressed at middle, frontal striae diverging anterad, central portion of frontal stria more or less straight, faintly sinuate at middle, crenulate; supraorbital stria absent; labrum about twice as wide as long, weakly emarginate anteriorly; left mandible untoothed, right with small basal tooth; pronotum with weak, indistinct prescutellar depression, ground punctation very conspicuous, interspersed coarser punctures becoming more numerous to sides; marginal pronotal stria continuous from sides to front, narrowly interrupted at middle; lateral submarginal stria continuous at side, curving inward, ending freely behind eye; anterior submarginal stria present, straight across front (not recurving posterad at ends), ending freely behind eye; median pronotal gland openings present between free ends of submarginal striae, about 4 puncture widths from anterior margin; elytron with two complete epipleural striae; all dorsal striae broadly but shallowly impressed, crenulate, inner and outer subhumeral striae complete in basal two-thirds, obscured by coarse punctures toward apex, striae 1-5 reaching base, progressively more abbreviated apically, 5^th^ stria fragmented over much of its length, sutural stria complete; elytra with very conspicuous ground punctation, as on pronotum, coarser punctures uniformly interspersed in interstriae; prosternal keel truncate at base, carinal striae complete, united in a narrow anterior arch; secondary carinal striae present for short distance behind prosternal gland openings; prosternal lobe short, but evenly rounded, reaching hypomeron at sides, marginal stria complete on anterior portion, obsolete at sides; mesoventrite straight to very weakly projecting at middle, marginal stria complete; mesometaventral stria detached at sides from lateral metaventral stria, displaced anterad and parallel to marginal mesoventral stria; lateral metaventral stria beginning behind mesometaventral suture, extending posterolaterad toward outer third of metacoxa; postmesocoxal stria recurved to mesepimeron; metaventral disk without coarse punctures in middle, but with large punctures at sides; 1^st^ abdominal ventrite with single lateral stria, with increasingly coarse punctures toward posterior margin, especially near ends of lateral striae; abdominal ventrites 2-4 with nearly uniform series of large, shallow punctures along posterior margins; propygidium with uniform coarse punctures separated by one-half their widths; pygidium with coarse punctures smaller, denser; marginal stria complete, constituted by a series of deep confluent punctures; protibia 4-dentate, with small marginal spines. Male not known.

#### Remarks.

This is a readily recognizeable species, distinguished by the coarse punctation on most of the body (Figs 85C–E) in combination with elytral striae which are complete basally but abbreviated toward apex.

#### Etymology.

This is among the most strongly punctate species of *Operclipygus*, and is named for this unusual appearance.

### Operclipygus lama

Mazur, 1988

http://species-id.net/wiki/Operclipygus_lama

Figs 85F–H 87A–E Map 31

Operclipygus lama Mazur, 1988: 295.

#### Type locality.

ECUADOR: Napo: Puerto Misahuallí [1°02'S, 77°40'W].

#### Type material.

**Holotype male**: Ecuador, Napo, Misahualli, II/19/1983; not examined (LUND).

#### Other material.

**BOLIVIA: Cochabamba:** 6: Est. Biol. Valle Sajta, Univ. San Simon, 67.5km E Villa Tunari, 17°06'19"S, 64°46'57"W, 300m, 9–13.ii.1999, FIT, lowland rain forest, F. Genier (CMNC), 2: 7–9.ii.1999, FIT, lowland rain forest, F. Genier (CMNC); **La Paz**: 1: Guanay, Uyapi, 15°25'S, 67°46'W, 20.x.1999, rotting logs, G. Carrasco (CHND). **ECUADOR: Orellana:** 1:Yasuní Res. Stn., 00°40'28"S, 76°38'50"W, 215m, 5–10.ix.1999, FIT, Primary forest, E.G. Riley (TAMU); same data except as noted: 2: 20–29.vi.1999, FIT, C.E. Carlton & A.K. Tishechkin (LSAM), 1: 28–5.vii.1999 (LSAM), 3: 22–28.vi.1999 (LSAM), 2: 12–20.vii.1999 (LSAM), 2: 26.vii-4.viii.1999 (LSAM), 1: 26.vii-4.viii.1999 (LSAM), 3: 5–12.vii.1999 (LSAM), 1: 23–30.vi.1999 (LSAM), 1: 5–11.vii.1999 (LSAM), 2: 28.vi–5.vii.1999 (LSAM), 1: 14–23.vii.1999 (LSAM), 1: 28.vi–5.vii.1999 (LSAM), 1: 11–18.vii.1999 (LSAM); 1: Yuturi Lodge, Rio Napo, 0°35'54"S, 76°2'18"W, 270m, 20–21.iii.1999, FIT, R. Brooks & D. Brzoska (SEMC); 16: Tiputini Biodiversity Station, 0.6376°S, 76.1499°W, 4–9.vi.2011, FIT, M.S. Caterino & A.K. Tishechkin, DNA Extract MSC-2174 (SBMNH, USFQ), 4: 5–25.ix.2000, D.J. Inward & K.A. Jackson (BMNH); 8: Parque Nac. Yasuní, Via Maxus at Puente Piraña, 0°39.5'S, 76°26'W, 14–20.vii.2007, FIT, A.K. Tishechkin (AKTC), 1: 20–24.vii.2008, FIT, A.K. Tishechkin (AKTC); 2: Payamino Research Station, 0°29'36.01"S, 77°17'29.15"W, 300m, 30.vii-12.viii.2007, FIT, tropical rainforest, CPDT Gillett (BMNH); **Sucumbíos:** 1: Sacha Lodge, 0°28'14"S, 76°27'35"W, 270m, 21–24.iii.1999, FIT, R. Brooks (SEMC). **PERU: Ayacucho**: 1: La Mar, Santa Rosa, 640m, 8–15.ix.1976, R. Gordon (USNM); **Loreto:** 2: 1.5km N Teniente Lopez, 2°35.66'S, 76°06.92'W, 210–240m, 20.vii.1993, FIT, R. Leschen (SEMC); 9: 1.5km N Teniente Lopez, 2°35.66'S, 76°06.92'W, 210–240m, 18.vii.1993, FIT, R. Leschen (SEMC); **Madre de Dios:** 6: 24.x.1982, rotten palm flowers, L. Watrous & G. Mazurek (FMNH); 1: Manu National Park, Zona res., Rio Manu, Cocha Juarez, trail nr. Manu Lodge, 18–24.ix.1991, FIT, A. Hartman (FMNH); 6: Manu National Park, Cocha Salvador, 12°0'13"S, 71°31'36"W, 310m, 20–21.x.2000, FIT, R. Brooks (SEMC); 1: Manu National Park, Pantiacolla Lodge, Alto Madre de Dios River, 12°39'22"S, 71°13'55"W, 400m, 23–26.x.2000, FIT, R. Brooks (SEMC); 9: Manu National Park, Cocha Cashu Bio. Sta., 11°53'45"S, 71°24'24"W, 350m, 17–19.x.2000, FIT, R. Brooks (SEMC); 1: Manu National Park, Rio Alto Madre de Dios, Pantiacolla Lodge, 12°39.3'S, 71°13.9'W, 14–19.xi.2007, FIT, D. Brzoska (SEMC); 1: Amazonas Lodge, N Atalaya, 12°52.2'S, 71°22.6'W, 480m, 10–13.xi.2007, FIT, D. Brzoska (SEMC); 1: Tambopata, Reserva Cuzco Amazonico, 15km NE Pto. Maldonado, 12°33'S, 69°03'W, 200m, 16.vii.1989, *Buchenavia* fruit fall, J. Ashe & R. Leschen (SEMC), 8: 24.vi.1989, FIT (SEMC), 22: 22.vi.1989, FIT (SEMC), 3: 20.vi.1989, FIT (SEMC), 6: 16.vii.1989, FIT (SEMC), 2: 19.vii.1989, FIT (SEMC), 5: 13.vii.1989, FIT (SEMC), 5: 13.vi.1989, FIT (SEMC), 1: 30.vi.1989, FIT (CHSM), 3: 28.vi.1989, FIT (SEMC), 4: 26.vi.1989, FIT (SEMC), 7: 17.vi.1989, FIT (SEMC), 5: 15.vi.1989, FIT (SEMC), 1: 16.vi.1989, Agaricales (SEMC), 1: 27.vi.1989, flat ascomycete (SEMC).

#### Diagnostic description.

**Figure 87.**
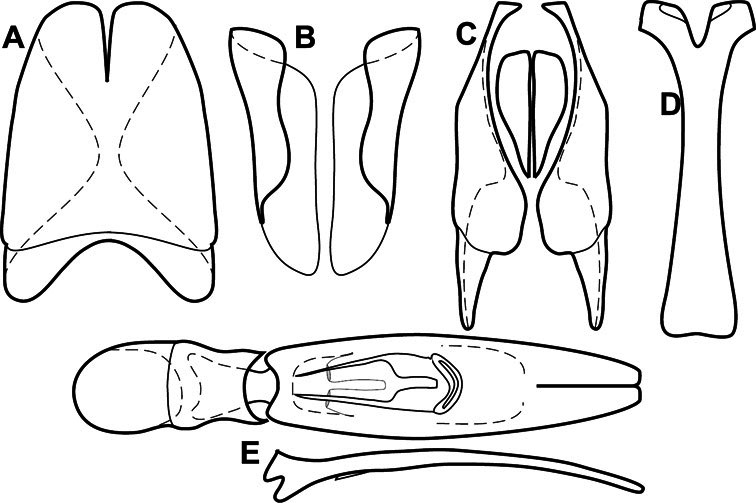
Male genitalia of *Operclipygus lama*. **A** T8 **B** S8 **C** T9 & T10 **D** S9 **E** Aedeagus, dorsal and lateral views.

Length: 1.93–2.18 mm, width: 1.93–2.00 mm; body piceous, rounded, convex; frons weakly depressed, frontal stria complete, continuous with complete supraorbital stria; pronotum with lateral submarginal stria complete, continued anteriorly by anterior marginal stria, which is then interrupted for the distance of the outwardly arcuate anterior pronotal emargination; anterior submarginal pronotal stria detached, barely recurved posterad at apices, median pronotal gland openings just beyond the ends of this stria; pronotal disk with few or no lateral punctures and lacking prescutellar impression; elytron with two complete epipleural striae, outer subhumeral stria entire, sinuate at middle, inner subhumeral stria absent, striae 1-3 complete, 4^th^ stria present in apical half, 5^th^ stria present in apical third, sutural stria present in apical three-fourths; prosternal keel projecting into mesoventral emargination, prosternal carinal striae connected in wide anterior arch, about one-fifth short of presternal suture; prosternal lobe short, marginal stria continued laterally by series of punctures; mesoventral marginal stria usually interrupted; mesometaventral stria arched forward in middle, metaventral disk lacking punctures; 1^st^ abdominal ventrite with two striae on each side, outer stria frequently interrupted; abdominal ventrites 3 and 4 with single series of very large punctures at sides; propygidium with fine, sparse ground punctation and a dense group of deep punctures slightly removed from all margins; pygidium with ground punctation denser than that of propygidium with coarser punctures concentrated along basal margin, more sparsely scattered elsewhere; marginal pygidial stria complete, finely crentulate. Male genitalia (Figs 87A–E): accessory sclerites absent; S8 with apical guides strongly developed from base to apex; S9 desclerotized along midline, with deep apical emargination and small, separate apical flanges; tegmen with basomedial process divided into two lateral processes; median lobe short, broad.

#### Remarks.

This is a rather isolated species, with the divided medioventral processes of the aedeagus (Fig. 87E) found in no other species. Externally, it is most easily recognized by the large deep punctures on ventrites 3 and 4 (Fig. 85G), and the small but deep punctures on the propygidium (Fig. 85H).

Although we did not study the type specimen, we have seen numerous specimens determined by the species’ author, all agreeing in characters with the original description, and to this widespread, more or less invariable species.

### Operclipygus florifaunensis sp. n.

urn:lsid:zoobank.org:act:4276B1D9-8586-41E0-9130-D4091E5D5CB9

http://species-id.net/wiki/Operclipygus_florifaunensis

**Figure 88.**
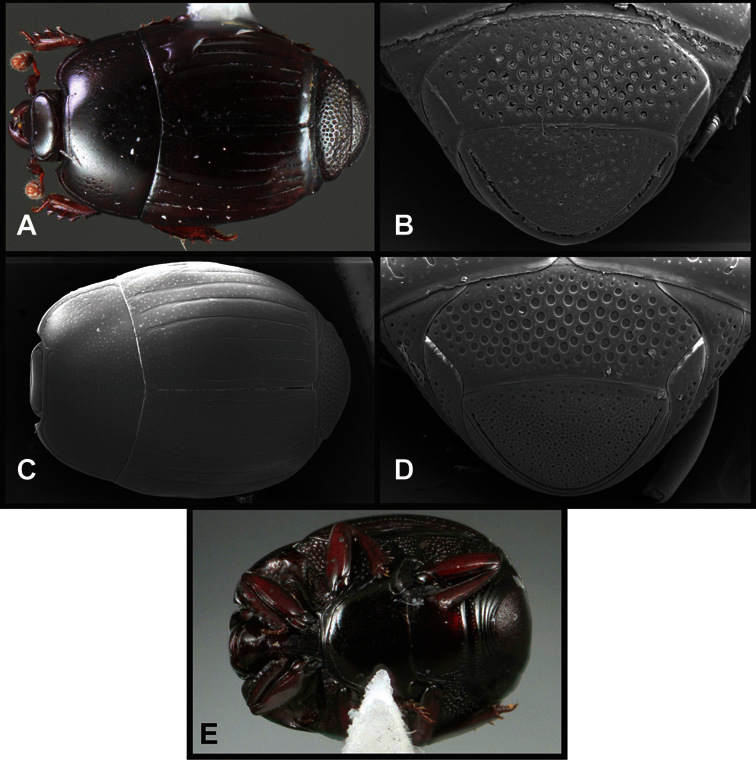
Various *Operclipygus* species. **A** Dorsal habitus of *Operclipygus florifaunensis* **B** Pygidia of *Operclipygus florifaunensis* **C** Dorsal habitus of *Operclipygus bosquesecus* **D** Pygidia of *Operclipygus bosquesecus* **E** Ventral habitus of *Operclipygus bosquesecus*.

Figs 88A–B 89A–E Map 31

#### Type locality.

BOLIVIA: Santa Cruz: 5 km SSE Buena Vista, Flora y Fauna Hotel [17°29.925'S, 63°39.128'W]

#### Type material.

**Holotype male**: “**BOLIVIA: Dpto. Santa Cruz.** 5km SSE Buena Vista, Flora y Fauna Hotel 17°29.925'S, 63°39.128'W. 440m F.I.T. 15–24 Dec 2003. S. & J. Peck” / “Caterino/Tishechkin Exosternini Voucher EXO-00587” (CMNC). **Paratypes** (17): **BOLIVIA: Santa Cruz:** 2: 3.7km SSE Buena Vista, Flora y Fauna Hotel, 17°29.9'S, 63°33.2'W, 400–440m, 2–9.xi.2002, FIT, R. Leschen (AKTC, LSAM), 2: 7–12.v.2004, FIT, A.R. Cline (AKTC, LSAM); 9: 5km SSE Buena Vista, Flora y Fauna Hotel, 17°29.925'S, 63°39.128'W, 440m, 15–24.xii.2003, FIT, S. & J. Peck (CMNC, AKTC, MSCC, FMNH); 1: 4–5km SSE Buena Vista, Hotel Flora y Fauna, 17°29'S, 63°33'W, 29.iv-6.v.2004, FIT, A.R. Cline (AKTC); 1: Potrerillos de Guenda, 17°40'49"S, 63°27'36"W, 47.iv.1998, H. & A. Howden (CMNC). **BRAZIL: Mato Grosso:** 1: Cuiabá, Fazenda Mutuca, 15.3145°S, 55.9703°W, 6–9.xii.2011, FIT, gallery forest, M.S. Caterino & A.K. Tishechkin, DNA Extract MSC-2252 (SBMNH), 1: 24.i.2009, FIT, F.H. Gava & J.R. Rocha (CEMT).

#### Diagnostic description.

**Figure 89.**
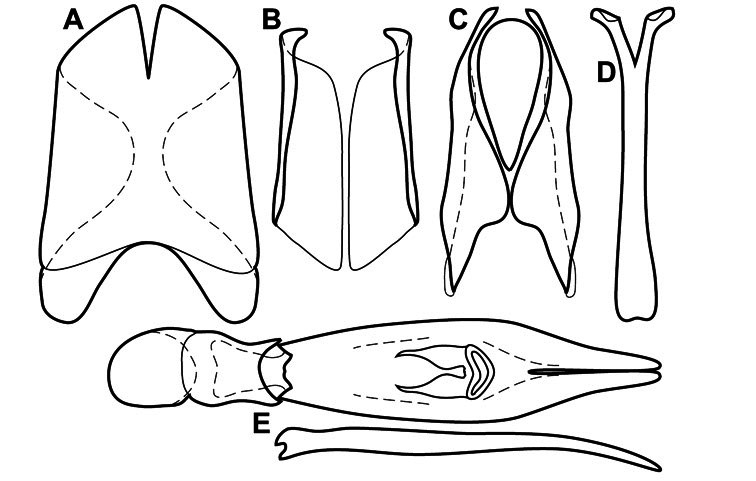
Male genitalia of *Operclipygus florifaunensis*. **A** T8 **B** S8 **C** T9 & T10 **D** S9 **E** Aedeagus, dorsal and lateral views.

Length: 1.84–2.18 mm, width: 1.53–1.81 mm; body rufo-piceous, elongate oval, widest near humeri, generally impunctate except in well-defined areas; head with frons broad, weakly depressed at middle, lateral arms of frontal striae diverging strongly anterad, central portion of frontal stria complete, outwardly arcuate, faintly sinuate at middle; supraorbital stria variably present, usually just at middle, detached from frontal stria; labrum small, about two-thirds as wide as long, rounded apically; pronotum lacking prescutellar impression, disk smooth with ground punctation fine and inconspicuous, with small number (<10) coarser punctures near sides; lateral marginal pronotal stria complete at sides and on to anterior margin but interrupted for width of head; lateral submarginal pronotal stria complete at sides, rather distant from margin, curving inward at front, ending freely behind eye, almost meeting anterior submarginal stria which has its ends curved posterad for a very short distance; median pronotal gland openings beyond ends of recurved stria, about 6 puncture widths from anterior margin; elytron with two complete epipleural striae, outer subhumeral stria complete, inner subhumeral stria absent, striae 1-3 complete, striae 4-5 subequal, present in apical half, sutural stria present in apical two-thirds; prosternum with keel produced posteriorly, with carinal striae running to one-fifth from presternal suture, united in a narrow anterior arch, barely interrupted at base; prosternal lobe rather narrow, marginal stria complete; mesoventrite emarginate, marginal stria interrupted at middle; mesometaventral stria arched forward to near mesoventral margin, sinuate near mesocoxa, continued by lateral metaventral stria posterolaterad toward outer corner of metacoxa, slightly abbreviated at apex; postmesocoxal stria recurved to mesepimeron; 1^st^ abdominal ventrite generally with two complete lateral striae, outer occasionally fragmented; propygidium with conspicuous, fine ground punctation and uniformly covered with large, deep punctures separated by about one-third their widths; pygidium with very dense ground punctation and slightly larger punctures separated irregularly by 1–2× their diameters; marginal pygidial stria deeply and coarsely impressed, complete. Male genitalia (Figs 89A–E): accessory sclerites absent; basal membrane attachment line of T8 tangential to basal emargination; S8 with lateral flanges abruptly developed at apex, with strong inner apical corners; S9 narrow, with deep apical emargination, small apical flanges, and with desclerotized basal and distal ends; T10 undivided; tegmen lacking medioventral process; median lobe wide and very short, with basal apodemes curved and tapering.

#### Remarks.

Although rather unremarkable externally, this species has highly autapomorphic male genitalia (Fig. 89), lacking accessory sclerites, with an undivided T10, and lacking any medioventral process on the tegmen. The deep propygidial punctures (Fig. 88B) and large punctures on the 3^rd^ and 4^th^ abdominal ventrites are reminiscent of *Operclipygus lama*, and preliminary analyses do suggest a sister group relationship between the two species. However, they are easily separated by the more elongate body form of *Operclipygus florifaunensis* (Fig. 88A *vs*. 85F) along with its more deeply impressed pygidial stria (Fig. 88B).

#### Etymology.

This species’ name recognizes the Flora y Fauna Hotel, a popular and productive ecotourism site in the Andean foothills of Santa Cruz, Bolivia, and the type locality of this species.

### Operclipygus bosquesecus sp. n.

urn:lsid:zoobank.org:act:30E6D513-4F57-4784-8B40-F231C17220A9

http://species-id.net/wiki/Operclipygus_bosquesecus

Figs 88C–E 90A–B, E, G Map 31

#### Type locality.

ECUADOR: Manabí: Lalo Loor Reserve [0.0832°S, 80.1520°W].

#### Type material.

**Holotype male**: “**ECUADOR: Manabí** 0.0832°S, 80.1520°W Lalo Loor Bosque Seco Res., 21–27.v.2011, FIT, AT1304, M. Caterino, A. Tishechkin” / “Caterino/Tishechkin Exosternini Voucher EXO-00599” (FMNH). **Paratypes** (9): **ECUADOR: Los Ríos:** 1: CCRP, 8.vi.1980, palma abierta, S. Sandoval (CHSM); **Manabí**: 6: Bosque Seco Lalo Loor, 0.0832°S, 80.1520°W, 21–27.v.2011, FIT, M.S. Caterino & A.K. Tishechkin, DNA Extract MSC-2169 (SBMNH, MSCC, AKTC, USFQ); **Pichincha**: 2: Rio Palenque Sta., 47km S Santo Domingo, 250m, 25.ii.1979, pan trap, S.A. Marshall (CHSM).

#### Diagnostic description.

**Figure 90.**
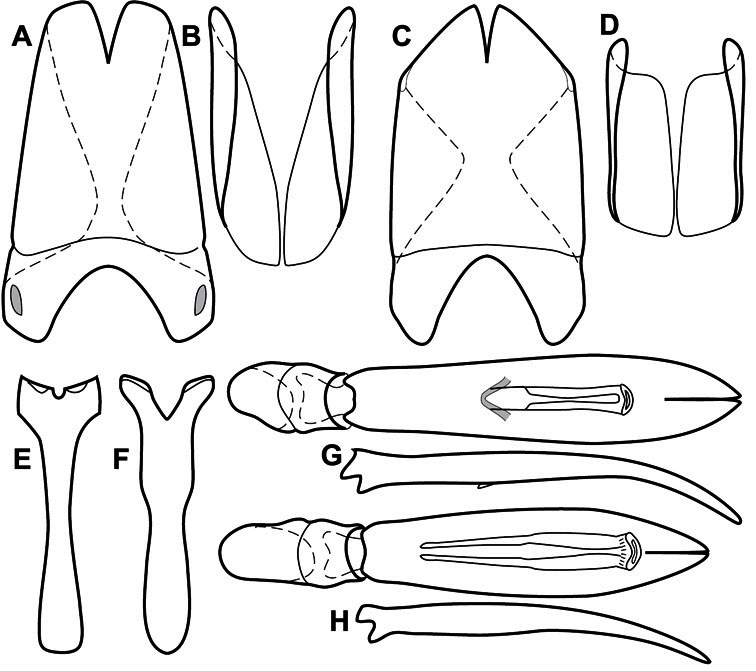
Male genitalia of various *Operclipygus* species. **A** T8 of *Operclipygus bosquesecus* **B** S8 of *Operclipygus bosquesecus* **C** T8 of *Operclipygus arnaudi* **D** S8 of *Operclipygus arnaudi* **E** S9 of *Operclipygus bosquesecus* **F** S9 of *Operclipygus arnaudi* **G** Aedeagus, dorsal and lateral views, of *Operclipygus bosquesecus* **H** Aedeagus, dorsal and lateral views, of *Operclipygus arnaudi*.

Length: 1.81–2.22 mm, width: 1.59–1.93 mm; body piceus, broadly rounded, widest near middle of elytra; frons flat, more or less parallel-sided, with sides of frontal stria weakly rounded; central portion of frontal stria outwardly arcuate, complete or narrowly detached from sides; supraorbital stria present, detached from frontal stria; epistoma convex, labrum short, very weakly asymmetrical, with left side more strongly produced than right; left mandible untoothed, right with small, acute basal tooth; pronotal disk with small, irregular but distinct prescutellar impression, with inconspicuous ground punctation and ~10 coarser, elongate punctures toward sides; marginal stria interrupted for width of head; lateral submarginal stria complete along side, curved inward at front nearly to anterior submarginal stria, the ends of which are perpendicularly recurved, median pronotal gland openings visible laterad its ends, about 8 puncture widths from anterior margin; elytron with three complete epipleural striae, outer subhumeral stria present in apical half only, mostly below a humeral swelling, inner subhumeral stria weakly impressed, present in most of apical half, striae 1-3 complete, 4^th^ stria present in apical two-thirds, 5^th^ stria present in apical half, sutural stria present in apical two-thirds, elytra depressed along suture; prosternal keel truncate at base, carinal striae complete, well separated, converging only slightly to front, connected basally and apically; prosternal lobe with complete marginal stria; mesoventrite with anterior margin straight, marginal stria complete; mesometaventral stria arched forward to near marginal stria, continued by lateral metaventral stria toward outer third of metacoxa; postmesocoxal stria recurved to mesepimeron; 1^st^ abdominal ventrite with single complete (inner) lateral stria and fragments of outer lateral stria, with series of small punctures along posterior margin; propygidium with fine sparse ground punctation, larger shallow punctures uniformly interspersed, separated by about one-half their diameters; pygidium with denser ground punctation and small coarse punctures irregularly but sparsely interspersed; marginal pygidial stria complete, fine, slightly crenulate. Male genitalia (Figs 90A–B, E, G): accessory sclerites present, small; T8 elongate, sides narrowing gradually to apex, basal emargination broadly rounded, basal membrane attachment line distad by about one-half basal emargination depth from its apex, apical emargination narrow; S8 with apical guides moderately well developed, largely constant in width from base to narrowly rounded apices; T9 with apices acute, convergent, opposing; T10 with halves large, separate; S9 narrowest at middle of stem, gradually widened to subtruncate, desclerotized base, with apical emargination distinct, apical flanges small and separate, lateral flanges parallel, shallow; tegmen widest beyond midpoint, with sides rounded to apex, narrowed slightly to base, with narrow, ‘U’-shaped medioventral process projecting beneath about one-third from base, apical third of tegmen moderately curved ventrad; basal piece short, about one-fourth tegmen length; median lobe nearly one-half tegmen length, with gonopore wide, basal apodemes separate throughout their lengths.

#### Remarks.

This species is best recognized by its small size, the presence of a prescutellar impression, the weak vestige of an inner subhumeral stria, and the strongly arched mesometaventral stria (Fig. 88E). There are also very few other *Operclipygus* on the dry western slopes of the Andes where this species occurs.

#### Etymology.

This species is named for the seasonally dry forest environment (in Spanish, bosque seco) where it is found.

### Operclipygus arnaudi

Dégallier, 1982

http://species-id.net/wiki/Operclipygus_arnaudi

Figs 90C–D, F, H 91A–B Map 31

Operclipygus arnaudi Dégallier, 1982: 158.

#### Type locality.

FRENCH GUIANA:Mana [5°39.5'N, 53°46.5'W].

#### Type material.

**Holotype**: **“**27-12-75 piége + cad. Oiseau+lézard, Piste de l’Acarouany, P.K. 5 MANA Guyane Française, N. DEGALLIER” / “*Operclipygus arnaudi* nov. sp. N DEGALLER 1982” / **“**HOLOTYPE” (MNHN); examined 2010.

#### Other material.

**BRAZIL: Acre**: 1: Serra do Divisor, Cruzeiro do Sul, 15–23.ix.2007, D. Passoa Moura (UFPR); **Amazonas**: 1: Reserva Ducke, 26km NE Manaus, ii.1995, FIT, M.G.V. Barbosa (BMNH); **Mato Grosso:** 1: Cotriguaçu, Fazenda São Nicolau, Matinha, 9°50.3'S, 58°15.05'W, x.2009, FIT, M.S. Gigliotti (CEMT), 2: x.2010, FIT, F.Z. Vaz-de-Mello (CEMT); 1: Cotriguaçu, Fazenda São Nicolau, Mata Norte, 9°49.15'S, 58°15.6'W, 8–14.xii.2010, FIT, F.Z. Vaz-de-Mello (CEMT); **Pará:** 1: Monte Alegre, 3°09'S, 52°03'W, 17.vi–3.vii.1992, FIT (CHND). **COLOMBIA: Vaupés:** 1: Parque Nac. Mosiro-Itajura (Caparú), Centro Ambiental, 1°04'S, 69°31'W, 60m, 20–30.i.2003, FIT, D. Arias & M. Sharkey (IAVH). **ECUADOR: Orellana:** 1:Parque Nac. Yasuní, Via Maxus at Puente Piraña, 0°39.5'S, 76°26'W, 20–24.vii.2008, FIT, A.K. Tishechkin (AKTC). **FRENCH GUIANA:** 1: Régina, Réserve des Nouragues, 4°2.27'N, 52°40.35'W, 3.xi.2009, FIT, SEAG (CHND); 1: Roura, 27.4km SSE, 4°44'20"N, 52°13'25"W, 280m, 10.vi.1997, flat ascomycete, J. Ashe & R. Brooks (CMNC); 1: Roura, 39.4km SSE, 4°32'43"N, 52°8'26"W, 270m, 29.v–10.vi.1997, FIT, J. Ashe, R. Brooks (SEMC); 2: Rés. Natur. De la Trinité, 4°4.011'N, 53°16.99'W, 7.ii.2011, Window trap, understory, SEAG (CHND); 1: Montagne des Chevaux, 4°43'N, 52°24'W, 18.iv.2009, FIT, SEAG (CHND), 2: 11.vii.2009, FIT, SEAG (CHND), 1: 22.xii.2008, FIT, SEAG (CHND); 1: Rés. Trésor, Route de Kaw Pk18, 4°36.63'N, 52°16.74'W, 225m, 21.xi.2009, FIT, SEAG (MNHN); 2: Belvèdére de Saül, point de vue, 3°1'22"N, 53°12'34"W, 30.xi.2010, Window trap, SEAG (CHND). **GUYANA:** 1: Essequibo R., Moraballi Creek, 5.ix.1929, Dark forest, Oxf. Univ. Expedn. (OUMNH). **PERU: Junín:** 1: 11km NE Puerto Ocopa, Los Olivos, 11°3.00'S, 74°15.52'W, 1200m, 29–30.iii.2009, FIT, A.K. Tishechkin, DNA Extract MSC-2146 (AKTC); **Loreto:** 1: Campamento San Jacinto, 2°18.75'S, 75°51.77'W, 172–215m, 9.vii.1993, FIT, R. Leschen (SEMC); 2: km 63, rd. Iquitos - Nauta, Rio Itaya, 4°15.205'S, 73°26.074'W, 140m, 9–13.i.2011, A.V. Petrov (AKTC, MUSM). **SURINAME**: **Commewijne**: 2: Akintosoela, CELOS Camp, 39km SE Suriname River bridge road to Redi Doti, 5°16'17"N, 54°55'15"W, 40m, 29.vi–3.vii.1999, FIT, Z. Falin (SEMC); **Para**: 1: nr. Overbridge River Resort, 5°31.8'N, 55°3.5'W, 15–18.ii.2010, FIT, C. Gillet, P. Skelley, W. Warner (FSCA); **Sipaliwini:** 1: CI-RAP Surv. Camp 2: Sipaliwini River, 2°10.521'N, 56°47.244'W, 210m, 27.viii–1.ix.2010, FIT, T. Larsen & A.E.Z. Short (SEMC); 2: CI-RAP Surv. Camp 3: Wehepai SE Kwamala, 2°21.776'N, 56°41.861'W, 237m, 3–7.ix.2010, FIT, T. Larsen & A.E.Z. Short (SEMC).

#### Diagnosis.

This species was thoroughly described by its author, but we take this opportunity to present some useful additional diagnosticcharacters: length: 2.18–2.56 mm, width: 1.87–2.09 mm; body broadly oval, largely impunctate dorsally; frons broad, depressed at middle, lateral arms of frontal stria diverging arcuately; frontal and supraorbital striae complete and continuous; submarginal pronotal striae rather removed from lateral and anterior margins, nearly meeting behind eye, with very narrow interruption; central portion of anterior pronotal margin distinctly projecting at middle; median pronotal gland openings beyond ends of recurved anterior submarginal stria, about 8 puncture widths from anterior margin; elytra with outer subhumeral stria complete, strongly impressed apically, inner subhumeral stria absent, striae 1-3 complete, 4^th^ stria present in apical half, 5^th^ stria present in apical third, sutural stria present in apical two-thirds; prosternal keel produced at base, carinal striae complete, sinuately subparallel, united by anterior arch; mesoventrite shallowly emarginate, marginal stria complete; mesometaventral stria weakly arching forward at middle, continuous with lateral metaventral stria which runs posterad toward inner third of metacoxa; 1^st^ abdominal ventrite with complete inner lateral stria, outer stria variable, complete, fragmented, or absent; propygidium with ground punctation sparse, but with uniformly close, small deep punctures separated by one-third their diameters; pygidium with dense ground punctation, sparse coarser punctures and a complete, deeply crenulate, but rather thin marginal sulcus. Male genitalia (Figs 90C–D, F, H): accessory sclerites absent; T8 parallel-sided for most of length, apices abruptly angled to apex, apical emargination narrow, basal emargination deep, nearly reaching basal membrane attachment line, ventrolateral apodemes most strongly developed at middle, not meeting at midline; S8 parallel-sided, with very narrow apical guides, halves weakly diverging along midline to near apex; T9 with ends only weakly convergent, subacute; T10 with halves separated; S9 narrowed just at middle, with basal and apical stems more or less parallel sided, basal third of stem largely desclerotized, apex with deep, acute median emargination, upturned apical flanges absent; tegmen with sides rounded, widest about one-third from apex, very weakly curved ventrad toward apex, lacking medioventral process; basal piece about one-third tegmen length; median lobe about three-quarters tegmen length, with strong proximal apodemes and broad gonopore.

#### Remarks.

**Figure 91.**
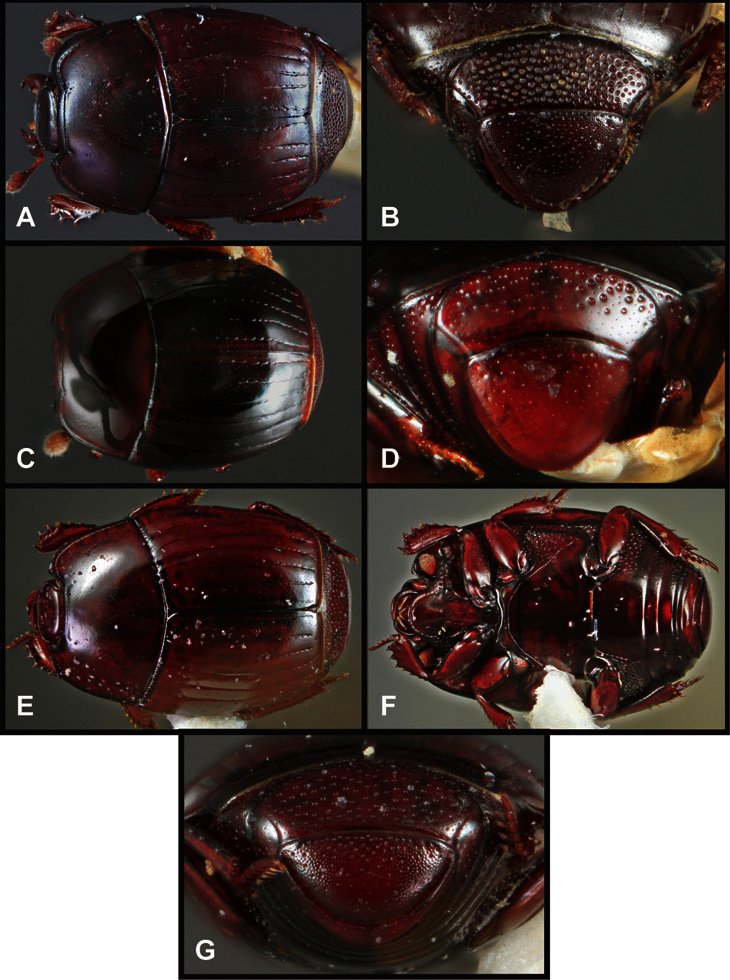
Various *Operclipygus* species. **A** Dorsal habitus of *Operclipygus arnaudi* (holotype) **B** Pygidia of *Operclipygus arnaudi* **C** Dorsal habitus of *Operclipygus subsphaericus* **D** Pygidia of *Operclipygus subsphaericus* **E** Dorsal habitus of *Operclipygus latipygus* **F** Ventral habitus of *Operclipygus latipygus* **G** Pygidia of *Operclipygus latipygus*.

This distinctive species can be recognized by its broad lateral pronotal margin (Fig. 91A), weakly produced anterior pronotal margin, complete outer subhumeral stria, very coarse propygidial punctation (Fig. 91B), and complete marginal pygidial sulcus.

### Operclipygus subsphaericus sp. n.

urn:lsid:zoobank.org:act:26C41B95-3B43-4B97-B4CE-1618E1726696

http://species-id.net/wiki/Operclipygus_subsphaericus

Figs 91C–D Map 31

#### Type locality.

BRAZIL: São Paulo: São Miguel [24°01'S, 48°00'W].

#### Type material.

**Holotype female**: “BRASIL:São Paulo, São Miguel, Elev.800m. Turvo-River.XII-1963, 24°01’[S]-48°00’[W], F. Plaumann leg.”/ “FMNH-INS 0000069135” / “♀” / “Caterino/Tishechkin Exosternini Voucher EXO-01341” (FMNH). **Paratype** (1, probably a male, but genitalia missing): **BRAZIL: Rio de Janeiro:** Represa do Rio Grande, Guanabara, iii.1972, F.M. Oliveira (UFPR).

#### Diagnostic description.

Length: 1.50–1.53 mm, width: 1.31–1.37 mm; body small, rounded, nearly circular, generally impunctate, faintly bicolored, head, pronotum, venter and pygidia rufescent, elytra piceous; head vertical when retracted; frons flat, relatively narrow, sides rounded, central portion of frontal stria outwardly arcuate, sinuate over antennal bases, complete or narrowly interrupted at sides; epistoma flat; labrum about twice as wide as long, apical margin straight; pronotum wide, sides evenly rounded to anterior corners; pronotal disk with basal plicae present in front of 3^rd^ elytral stria, short; small, irregular but distinct prescutellar impression present, disk otherwise with fine, sparse ground punctation, no coarser punctures present; lateral marginal stria interrupted behind head; lateral submarginal pronotal stria complete, close to margin, curved inward anteriorly nearly to anterior submarginal stria, which is crenulate, narrowly recurved posterad at sides; median pronotal gland openings situated between free ends of submarginal striae, about 6 puncture widths from anterior margin; elytron with single complete epipleural stria, outer subhumeral stria present in apical half, laterad humeral swelling, inner subhumeral absent, dorsal striae 1-3 complete, 4^th^ stria present in apical two-thirds, 5^th^ stria present in apical half, sutural stria present in apical two-thirds; prosternal keel weakly emarginate at base, carinal striae converging to front, connected anteriorly but not basally; mesoventrite projecting at middle, marginal stria complete; mesometaventral stria arched forward at middle, sinuate near mesocoxae, continued by lateral metaventral stria, which curves posterolaterad toward middle of metepimeron; metaventral disk impunctate at middle, with few coarse lateral punctures; 1^st^ abdominal ventrite with single arcuate inner lateral stria, and weak series of small punctures along posterior margin; propygidium with ground punctation very sparse, coarser shallow, round punctures in basal two-thirds, denser and larger toward base, apical third impunctate; pygidium with very small, sparse punctures separated by 4´ their diameters or more, diminishing to apex; apical marginal stria fine, present on apical half, obsolete at base. Male: genitalia not known.

#### Remarks.

The small size and strongly convex body of this species (Fig. 91C) is practically diagnostic among *Operclipygus*. In addition the faint bicoloration, propygidium with coarse punctures restricted largely to the basal half (Fig. 91D), and the fine, incomplete marginal pygidial sulcus will help identify it. The paratype specimen is probably a male (genitalia were dissociated while in bulk sample, evidently), exhibiting a depressed metaventral disk, a fairly common sexual dimorphism. This specimen differs in a few other minor characters, most noteably lacking any indication of darker elytra, and also having the marginal mesoventral stria interrupted at the middle. With single specimens from each of two localities, it is impossible to interpret the significance of these characters.

#### Etymology.

This species’ name refers to its unusually rounded and convex body form, quite distinctive within *Operclipygus*.

### Operclipygus latipygus sp. n.

urn:lsid:zoobank.org:act:A97D9239-9A6E-4C9E-81A6-2D86F82CB9B1

http://species-id.net/wiki/Operclipygus_latipygus

Figs 91E–G Map 32

#### Type locality.

PERU: Madre de Dios: Manú National Park, Manú Lodge [12.12°S, 71.09°W].

#### Type material.

**Holotype female**: “PERU: Dept. Madre de Dios, Manu Prov., Parque Nac. Manu, Zona Res., Rio Manu, Cocha Juarez, trail nr. Manu”/ “_Lodge, 18–24.IX.1991, flight intercept trap, A. Hartman, FIELD MUSEUM” / “♀” / “FMNH-INS 0000069208” (FMNH). **Paratypes** (2): **PERU: Madre de Dios:** 1: Tambopata, Reserva Cuzco Amazonico, 15km NE Pto. Maldonado, 12°33'S, 69°03'W, 200m, 17.vi.1989, FIT, J. Ashe & R. Leschen (SEMC); 1: Manu National Park, Pantiacolla Lodge, Alto Madre de Dios R., 12°39.3'S, 71°13.9'W, 420m, 14–19.xi.2007, FIT, D. Brzoska (SEMC).

#### Diagnostic description.

Length: 1.81–.03 mm, width: 1.44–1.68 mm; body rufobrunneus, elongate oval, widest just behind humeri; frons broad, flat, sides rounded, frontal stria outwardly arcuate, fine, complete; supraorbital stria absent; epistoma markedly convex along anterior edge; labrum short, transversely ridged, apical margin weakly emarginate; left mandible with blunt basal swelling, right with small, acute tooth; pronotal disk with sublinear prescutellar impression about equal in length to scutellum, with fine ground punctation and ~8 shallow, larger punctures near sides; marginal pronotal stria interrupted behind width of head; lateral submarginal pronotal stria complete, curved inward anteriorly nearly to anterior submarginal stria, which is weakly crenulate, narrowly recurved posterad at sides; median pronotal gland openings situated beyond free ends of anterior submarginal striae, about 8 puncture widths from anterior margin; elytron with single complete epipleural stria, outer subhumeral stria present in apical half, below humeral swelling, inner subhumeral absent, dorsal striae 1-3 complete, 4^th^ stria present in apical third, 5^th^ stria present in apical third, sutural stria present in apical two-thirds; prosternal keel produced at base, carinal striae extremely fine, connected in narrow anterior arch about one-fourth from presternal suture, posteriorly barely disconnected along basal margin; prosternal lobe narrow, marginal stria complete, slightly diverging from margin toward base; mesoventrite emarginate in front, marginal stria complete; mesometaventral stria arched forward close to marginal stria, continuous with lateral metaventral stria which runs obliquely toward posterior corner of metepisternum, ending about two puncture widths short of it; median part of metaventral disk impunctate; 1^st^ abdominal ventrite with complete inner lateral stria and basal fragments of outer; propygidium with small, shallow, slightly elongated punctures separated by 1.5× their widths; pygidium with ground punctation moderately dense, slightly larger punctures interspersed densely along basal margin, more sparsely toward apex; marginal pygidial sulcus deep, crenulate, unusually distant from margin, especially at middle, with a flat marginal bead about one-fifth pygidial length contrasting with convex basal part. Male: not known.

#### Remarks.

The unusual pygidial sulcus, distant from the margin leaving a broad apical marginal bead (Fig. 91G) is sufficient to distinguish this species. The very fine prosternal striae (Fig. 91F) are also distinctive. It shares some characters with members of the *Operclipygus hospes* group, and may belong there. However, discovery of a male will be necessary to test that idea.

**Map 32.**
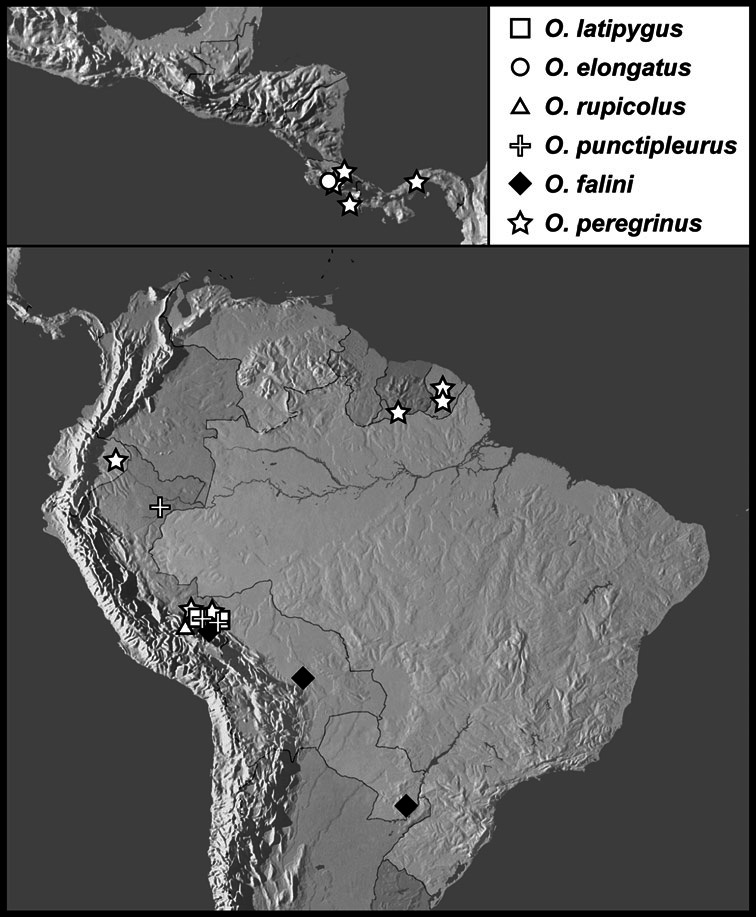
Records of the *Operclipygus* incertae sedis spp.

#### Etymology.

This species’ name is based on the wide pygidial bead beyond its marginal sulcus.

### Operclipygus elongatus sp. n.

urn:lsid:zoobank.org:act:91C3C133-CB90-48AC-BFE5-E3E2B656D356

http://species-id.net/wiki/Operclipygus_elongatus

**Figure 92.**
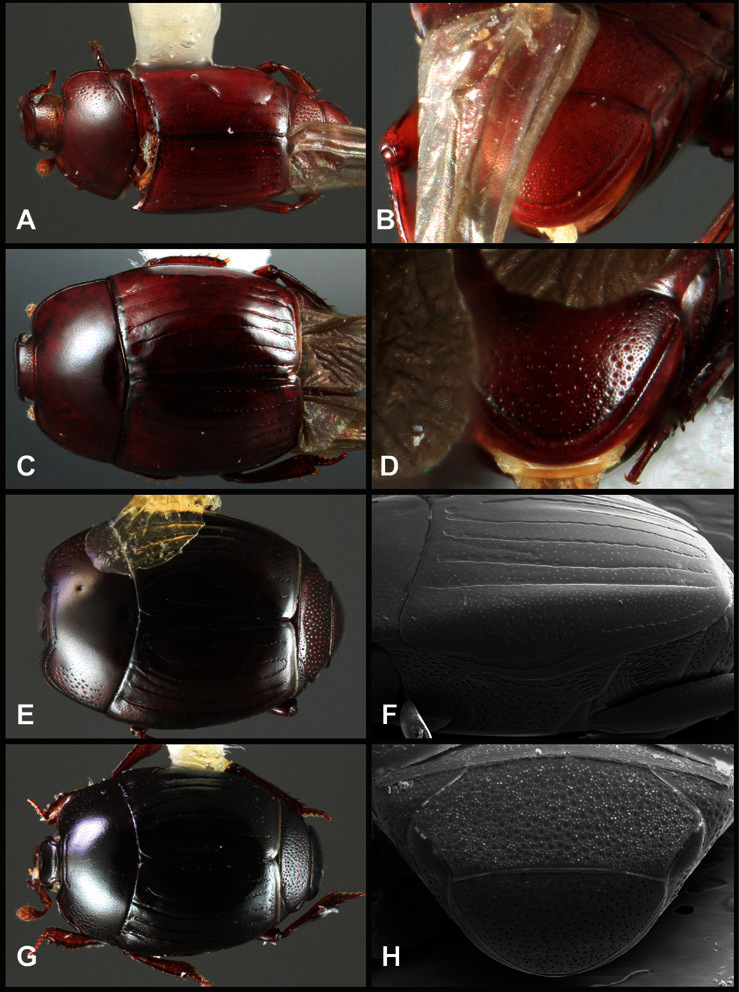
Various *Operclipygus* species. **A** Dorsal habitus of *Operclipygus elongatus* **B** Pygidia of *Operclipygus elongatus* **C **Dorsal habitus of *Operclipygus rupicolus* **D** Pygidia of *Operclipygus rupicolus* **E** Dorsal habitus of *Operclipygus punctipleurus* **F** Lateral habitus of *Operclipygus punctipleurus* **G** Dorsal habitus of *Operclipygus falini* **H** Pygidia of *Operclipygus falini*.

Figs 92A–B 93A–B, E, G Map 32

#### Type locality.

COSTA RICA: Alajuela: Peñas Blancas [10°04'N, 84°37'W].

#### Type material.

**Holotype male**: “COSTA RICA:Alajuela, Peñas Blancas, 1190m 20 May 1989, J. Ashe, R. Leschen, R. Brooks, ex. flight intercept trap” / “Snow Entomol. Mus. Costa Rica Exped. #273” / “Caterino/Tishechkin Exosternini Voucher EXO-00286” (SEMC).

#### Diagnostic description.

**Figure 93.**
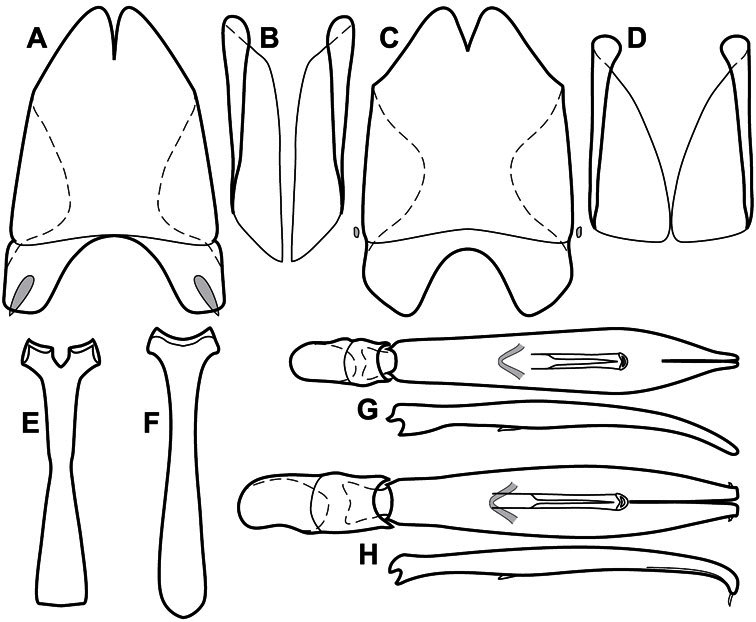
Male genitalia of various *Operclipygus* species. **A** T8 of *Operclipygus elongatus* **B** S8 of *Operclipygus elongatus* **C** T8 of *Operclipygus punctipleurus* **D** S8 of *Operclipygus punctipleurus* **E** S9 of *Operclipygus elongatus* **F** S9 of *Operclipygus punctipleurus* **G** Aedeagus, dorsal and lateral views, of *Operclipygus elongatus* **H** Aedeagus, dorsal and lateral views, of *Operclipygus punctipleurus*.

Length: 1.90 mm, width: 1.31 mm; body rufescent, elongate, nearly parallel-sided; frons weakly depressed in middle, with ground punctation conspicuous; sides of frontal stria divergent between eyes, interrupted over antennal bases, sinuate across front; supraorbital stria connected to frontal stria at sides, but interrupted at middle, ends curved slightly forward; epistoma convex, with slightly denser punctation than frons; labrum about twice as wide as long, outer margin sinuate, weakly projecting at middle; left mandible with incisor bluntly produced, but not toothed, right mandible with acute basal tooth; pronotal disk with prescutellar puncture in middle of weak depression, with fine ground punctation, with numerous (>30) coarser punctures at sides; lateral marginal stria interrupted for width of head, central portion of anterior margin weakly produced; lateral submarginal stria complete, curved inward anteriorly nearly to anterior submarginal stria, which is weakly crenulate, narrowly obliquely recurved at sides; median pronotal gland openings situated between free ends of submarginal striae, about 4 puncture widths from anterior margin; elytron with single epipleural stria, outer subhumeral stria present in apical half, inner subhumeral absent, striae 1-4 complete, 5^th^ stria present in apical two-thirds and with basal point, sutural stria nearly complete, barely abbreviated at base; prosternal keel weakly produced at base, carinal striae complete, subparallel, connected anteriorly and posteriorly; prosternal lobe narrow, marginal stria complete; mesoventrite shallowly emarginate in front, marginal stria complete; mesometaventral stria arched forward to middle of mesoventrite, sinuate near mesocoxa, continued by lateral metaventral stria posterolaterad toward middle of metacoxa; postmesocoxal stria recurved to mesepimeron; short fragment of secondary lateral metaventral stria present behind mesocoxa; 1^st^ abdominal ventrite with inner lateral stria complete, outer obsolete in posterior half; propygidium uniformly covered with medium sized, shallow punctures, separated by about one-half their widths, ground punctation fine and sparse; pygidium with denser ground punctation, with slightly larger punctures sparsely interspersed; marginal pygidial sulcus complete, deep, faintly crenulate, slightly removed from margin, with flat marginal bead about one-eighth total pygidial length. Male genitalia (Figs 93A–B, E, G): accessory sclerites present; T8 with sides evenly convergent, apical emargination narrow, basal emargination deep, broad, its apex tangential to basal membrane attachment line, ventrolateral apodemes most strongly developed basally, not meeting at midline; S8 with sides slightly divergent, with apical guides widening to apex, bluntly rounded apically with inner lobe, ventrally with halves weakly diverging to near apex; T9 with apices weakly convergent, subacute; T10 with halves separated; S9 with stem narrowest at middle, strongly widened to truncate base, apex with triangular median emargination, with apical flanges separate and weak; tegmen with sides straight, slightly widening from base to apex, then narrowed in apical fourth to subacute apex, with narrow ‘U’-shaped medioventral process projecting beneath about one-third from base; tegmen weakly curved ventrad toward apex; basal piece nearly one-half tegmen length; median lobe about one-third tegmen length, with proximal apodemes separate.

#### Remarks.

The elongate body shape (Fig. 92A) and unusually interrupted supraorbital striae will help to identify *Operclipygus elongatus*. The departure of the pygidial sulcus from the margin (Fig. 92B) is somewhat similar to the condition in *Operclipygus latipygus*, and this may be indicative of a relationship between them. However, they are easily distinguished by other characters.

#### Etymology.

This species’ name refers to its body shape, which is more markedly elongate than most *Operclipygus*.

### Operclipygus rupicolus sp. n.

urn:lsid:zoobank.org:act:A8AE163C-6634-4B94-B897-00FD2B58D29D

http://species-id.net/wiki/Operclipygus_rupicolus

Figs 92C–D Map 32

#### Type locality.

PERU: Cusco: Cock of the Rock Lodge NE of Paucartambo [13°03.3'S, 71°32.7'W].

#### Type material.

**Holotype female**: “**PERU**: Dept. Cusco: Cock of the Rock Lodge, NE Paucartambo 13°03.3'S, 71°32.7'W 1120m, 4–9-XI-2007 D.Brzoska, ex. flight intercept trap PER1B07 001” / “SEMC0871399” / “♀” (MUSM).

#### Diagnostic description.

Length: 2.34 mm, width: 1.81 mm; body rufescent, elongate oval, widest at middle of elytra; frons flat with sides rounded, frontal stria continuous, arcuate in front; supraorbital stria absent; epistoma short, flat; labrum short, with apex asymmetrical, left side more strongly produced than right; left mandible untoothed, right with acute basal tooth; pronotal disk depressed along basal margin between the 4^th^ elytral striae, with fine ground punctation only, lacking coarse punctures at sides; marginal stria interrupted for width of head; lateral submarginal stria complete, curved inward anteriorly nearly to anterior submarginal stria, which is weakly crenulate, narrowly recurved posterad at sides; median pronotal gland openings situated laterad end of anterior submarginal stria, about 8 puncture widths from anterior margin; elytron with two complete epipleural striae, outer subhumeral stria complete, inner subhumeral stria absent, striae 1-4 complete, 5^th^ stria present in apical half, sutural present in apical two-thirds; apices of striae 2-5 weakly connected by series of apical punctures; prosternal keel weakly produced at base, carinal striae complete, converging to front, connected by narrow anterior arch, secondary striae present behind prosternal gland openings, weak; prosternal lobe narrow, marginal stria complete; mesoventrite shallowly emarginate in front, marginal stria complete; mesometaventral stria arched forward to near middle of mesoventrite, continued by lateral metaventral stria which runs obliquely toward outer corner of metacoxa; central part of metaventral disk impunctate; 1^st^ abdominal ventrite with two complete lateral striae; propygidium uniformly covered with small punctures separated by about their widths; pygidium with ground punctation dense, but extremely fine, with slightly larger punctures densest toward base, sparser toward apex; marginal pygidial sulcus complete, deep, outer edge crenulate, inner edge smooth. Male: not known.

#### Remarks.

This species is not extremely distinctive, but the lack of lateral pronotal punctures (Fig. 92C), the weak connections among the apices of several elytral striae, the complete mesoventral marginal stria, and the deeply impressed pygidial sulcus (Fig. 92D) together will distinguish it.

#### Etymology.

This species’ name comes from the Latin name of the Andean Cock-of-the-rock, *Rupicola peruviana* (Latham), acknowledging the lodge where the species was collected.

### Operclipygus punctipleurus sp. n.

urn:lsid:zoobank.org:act:1E491274-7976-418B-A8C5-8B3D398552F8

http://species-id.net/wiki/Operclipygus_punctipleurus

Figs 92E–F 93C–D, F, H Map 32

#### Type locality.

PERU: Madre de Dios: 15 km NE Puerto Maldonado, Cuzco Amazónico Reserve [12°33'S, 69°03'W].

#### Type material.

**Holotype male**: “PERU: Tambopata Prov. Madre de Dios Dpto. 15km NE Puerto”/ “Maldonado, Reserva Cuzco Amazónico 12°33'S, 69°03'W 200m, Plot #Z2U16”/ “2 July 1989, J. S. Ashe, R. A. Leschen #366 ex., flight intercept trap” / “Caterino/Tishechkin Exosternini Voucher EXO-00258” (SEMC). **Paratypes** (15): **PERU: Loreto:** 1: Iquitos, 90m, 7.v.1992, FIT, J. Danoff-Berg (SEMC); 4: 15km. From Ucayali on R. Calleria, Colónia Calleria, 13.x.1961, B. Malkin (FMNH, MSCC, AKTC); **Madre de Dios:** 1: 25.x.1982, litter along river, L. Watrous & G. Mazurek (FMNH); 2: Manu National Park, Zona res., Rio Manu, Cocha Juarez trail nr. Manu Lodge, 18–24.ix.1991, FIT, A. Hartman (FMNH); 1: Tambopata, Reserva Cuzco Amazonico, 15km NE Pto. Maldonado, 12°33'S, 69°03'W, 200m, 17.vi.1989, FIT, J. Ashe & R. Leschen (SEMC), 1: 2.vii.1989, FIT, J. Ashe & R. Leschen (SEMC), 2: 30.vi.1989, FIT, J. Ashe & R. Leschen (SEMC), 1: 4.vii.1989, FIT, J. S. Ashe, R. Leschen, D. Silva (CHSM), 1: 13.vii.1989, FIT, J. Ashe & R. Leschen (CHSM), 1: 19.vii.1989, FIT, J. Ashe & R. Leschen (SEMC).

#### Diagnostic description.

Length: 1.84–2.22 mm, width: 1.68–2.03 mm; body rufo-piceous to piceous, sides rounded, subdepressed, dorsum rather flat; frons strongly depressed in middle, frontal stria present only as short median fragment, detached from lateral portions; supraorbital stria present only as isolated median fragment; pronotum with lateral submarginal stria obsolete in basal half, curved inward anteriorly, but not meeting anterior submarginal stria, the ends of which are barely recurved at sides; marginal stria continuous on lateral and anterior margins, rarely interrupted at middle; median pronotal gland openings present between ends of lateral and anterior submarginal striae; prescutellar impression distinct, oval; pronotal disk with numerous coarse punctures at sides; elytron with single complete epipleural stria, with subhumeral interval strongly elevated, enlarging the epipleural region, which bears 15–20 coarse punctures in basal half, outer subhumeral stria present in apical half, inner subhumeral stria absent, dorsal striae broadly, crenulately impressed toward their bases, striae 1-4 complete, 5^th^ stria present in apical half, sutural stria nearly complete, diverging from suture, with basal puncture (which may represent vestige of arch between sutural and 5^th^ striae); prosternum broad, emarginate at base, carinal striae joined anteriorly and posteriorly; mesoventrite projecting anteriorly, marginal stria complete, mesometaventral stria broadly angulate to middle of mesoventrite; central portion of metaventral disk lacking coarse punctures; abdominal ventrite 1 with numerous coarse punctures on each side, with 2 lateral striae, outer stria abbreviated behind metacoxa; propygidial disk with uniform coarse, shallow punctures separated by about one-third their widths; pygidium with dense, fine ground punctation, with barely larger punctures sparsely interspersed; marginal pygidial stria very fine, obsolete in basal corners. Male genitalia (Figs 92C–D, F, H): accessory sclerites present, very small; T8 with sides more or less parallel in basal two-thirds, angled to narrow apex, desclerotized at angle, basal apodemes oblquely truncate, basal emargination rounded, basal membrane attachment line distad emargination by about one-half its depth, ventrolateral apodemes short, distantly separated ventrally; S8 with sides convergent, apical guides gradually increasing in width from base to apex, rounded apically, ventral halves separate at base, gradually divergent to apex; T9 with basal apodemes parallel-sided, sides slightly widened at basal third, convergent in apical two-thirds; apices narrow, subacute, not converging; T10 with halves separate; S9 with stem narrowest near apex, widened to basal fourth, then narrowed to rounded apex, head rather small, apically arcuate, lacking distinct median emargination, apical flange continuous; tegmen with sides rounded, widest at middle, evenly narrowed to base and apex, apex appearing truncate from above, with ventrally directed acute, membraneous processes projecting from outer corners, somewhat walrus tusk-like in appearance; medioventral process narrowly ‘U’-shaped, weakly projecting beneath about one-fourth from base; basal piece nearly one-half tegmen length; median lobe about one-fourth tegmen length, filamentous part of proximal apodemes inconspicuous.

#### Remarks.

This is a very distinct species, most easily recognized by the elevated and punctate epipleuron (Fig. 92F). The presence of punctures on the first ventrite in combination with an impunctate metaventrite is also rather unusual (members of the *Operclipygus sejunctus* group also have punctures in both places), although the following, probably related, species also exhibits them. The tusk-like apical processes of the aedeagus (Fig. 93H) are unusual, exhibited also by *Operclipygus siluriformis*. In most other respects, however, both externally and genitalically, these two species show few significant similarities.

#### Etymology.

This species is named for the punctures of the elytral epipleuron.

### Operclipygus falini sp. n.

urn:lsid:zoobank.org:act:C454AE21-DD78-4EF2-8298-719964FA834D

http://species-id.net/wiki/Operclipygus_falini

Figs 92G–H 94A–B, E, G Map 32

#### Type locality.

PARAGUAY: Cazaapá: San Rafael Reserve [26°17.83'S, 55°43.13'W].

#### Type material.

**Holotype male**: “PARAGUAY: Cazaapá, Hermosa, prop. Lopez family, San Rafael Reserve, bank Rio Tebicuary, 26°17'23"S, 55°43'7"W, 80 m, 1–4 DEC 2000; Z.H.Falin, PAR1F00 107ex. flight intercept trap” / “SM0275398 KUNHM-ENT” (SEMC). **Paratypes** (2): same data as type (SEMC).

#### Other material.

**BOLIVIA**: 1: Las Juntas, xii.1913, Steinbach (CMNH). **PERU: Madre de Dios:** 1: Amazonas Lodge, N Atalaya, 12°52.2'S, 71°22.6'W, 480m, 10–13.xi.2007, FIT, D. Brzoska (SEMC); 1: Tambopata, Reserva Cuzco Amazonico, 15km NE Pto. Maldonado, 12°33'S, 69°03'W, 200m, 20.vi.1989, FIT, J. Ashe & R. Leschen (CHSM).

#### Diagnostic description.

**Figure 94.**
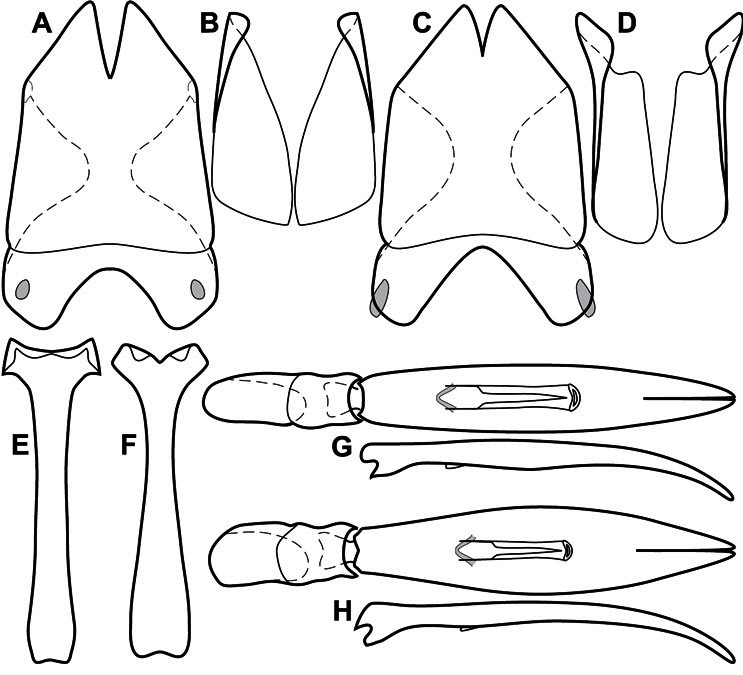
Male genitalia of various *Operclipygus* species. **A** T8 of *Operclipygus falini* **B** S8 of *Operclipygus falini* **C** T8 of *Operclipygus peregrinus* **D** S8 of *Operclipygus peregrinus* **E** S9 of *Operclipygus falini* **F** S9 of *Operclipygus peregrinus* **G** Aedeagus, dorsal and lateral views, of *Operclipygus falini* **H** Aedeagus, dorsal and lateral views, of *Operclipygus peregrinus*.

Length: 1.97–2.15 mm, width: 1.68–1.87 mm; body piceous, with conspicuous fine ground punctation throughout, ovoid with sides weakly rounded; frons strongly depressed at middle, with sides of frontal stria arcuately divergent at sides, interrupted above antennal bases, with a short isolated median arc; supraorbital stria present but disconnected from frontal stria; labrum about half as long as wide, apical margin straight; pronotal disk with distinct, irregularly oval prescutellar impression, with numerous coarse, elongate punctures near sides; marginal pronotal stria nearly complete across front, narrowly interrupted at middle; lateral submarginal pronotal stria present only in anterior half, curving inward in front, ending freely behind eye; anterior submarginal pronotal stria transverse, apices sinuate or barely recurved; median pronotal gland openings just laterad apices of anterior submarginal stria, about 6 puncture widths from anterior margin; elytron with two complete epipleural striae, outer subhumeral stria present in apical half, interrupted at middle, with a short basal fragment, inner subhumeral absent, striae 1-4 complete, 5^th^ stria present in apical half, sutural stria present in apical two-thirds; elytral disk with few punctures arranged subserially along apical margin; prosternal keel broad, flat, shallowly emarginate at base, carinal striae straight, converging to front, connected in a wide anterior arch; mesoventral margin sinuate, projecting at middle, marginal stria complete, additional strial fragments in anterior corners; mesometaventral stria detached from lateral metaventral, arching weakly forward onto posterior third of mesoventrite; anterior end of lateral metaventral stria extending mediad beyond apex of mesometaventral stria, extending posterolaterad toward middle of metacoxa; central part of metaventral disk with conspicuous ground punctation, but without coarser punctures; 1^st^ abdominal ventrite with two complete lateral striae, inner ones convergent anterad (though still widely separate where they meet anterior margin); propygidium and pygidium both with very dense ground punctation, both with slightly larger round punctures interspersed, those of the propygidium separated by about their widths at middle of disk, becoming sparser toward the sides, pygidial punctures smaller, but generally more uniformly distributed across disk; marginal pygidial stria fine, present in apical half to three-fourths, obsolete at base. Male genitalia (Figs 94A–B, E, G): accessory sclerites present, small; T8 parallel-sided for most of length, basal apodemes weakly divergent, apices abruptly angled mediad, with apparent desclerotized area at corner, apical emargination narrow, basal emargination shallow, rounded, basal membrane attachment line distad by about one-third depth of basal emargination, ventrolateral apodemes most strongly developed at middle, not meeting at midline; S8 weakly convergent to apex, with apical guides only developed at apex, with narrowly rounded apices, halves approximate in basal fourth, diverging to apex; T9 with ends only weakly convergent, subacute; T10 with halves separated; S9 with stem nearly parallel-sided, just slightly expanded toward base, with small basal emargination, apex evenly rounded, with continuous, medially narrowed apical flange; tegmen with sides rounded, widest near midpoint, narrowed slightly to base, more strongly narrowed to subacute apex, with rather wide ‘U’-shaped mediovental process projecting beneath about one-fourth from base; basal piece about one-half tegmen length, with long basal foramen; median lobe about half tegmen length.

#### Remarks.

This species can be recognized by the combination of conspicuous pronotal ground punctation (Fig. 92G), abbreviated lateral submarginal pronotal stria, very numerous coarse, lateral pronotal punctures, interrupted outer subhumeral elytral stria, and the very dense ground punctation of both the propygidium and pygidium (Fig. 92H). It appears to be related to *Operclipygus punctipleurus*, but completely lacks the characteristic epipleural punctures of that species. It also shows some similarity to *Operclipygus wenzeli*. We limit the type series to those specimens from Paraguay.

#### Etymology.

This species is named after Zachary Falin of the University of Kansas, in recogniton of his numerous contributions to this study, assisting with loans, as well as having collected the types of this, and several other new species.

### Operclipygus peregrinus sp. n.

urn:lsid:zoobank.org:act:D7F0F7FB-B91D-4C65-A2A2-44BBFCBDF0F0

http://species-id.net/wiki/Operclipygus_peregrinus

Figs 94C–D, F, H 95A–B Map 32

#### Type locality.

PANAMA: Colón: San Lorenzo National Park, Achiote [9°12'N, 79°58'W].

#### Type material.

**Holotype male**: “**PANAMA**: Prov. Colón, Achiote – P. N. San Lorenzo N 09°12', W 79°58' Cafetal A Dist. 100m Tr. Intercepción, A. Mercado 26.VI-10.VII.2007” / “Operclipygus sp. # 9 San Lorenzo Inventory A.K. Tishechkin det. 2010” / “Caterino/Tishechkin Exosternini Voucher EXO-00257” (FMNH). **Paratypes** (11) **PANAMA: Colón:** 1: Parque Nac. San Lorenzo, Achiote, Cafetal B, 9°12'N, 79°59'W, 50m, 8–22.v.2007, FIT, A. Mercado (GBFM); 1: San Lorenzo Forest, STRI crane site, 9°17'N, 79°58'W, 21–24.v.2004, FIT, A.K. Tishechkin (GBFM). **COSTA RICA: Alajuela:** 1: Peñas Blancas, 875m, 19.v.1989, FIT, J. Ashe, R. Brooks, R. Leschen (SEMC), 1: 800m, 19.v.1989, FIT, J. Ashe, R. Brooks, R. Leschen (CHSM); **Limón:** 1: Area Cons. Tortuguero, Sector Cerro Cocori, Fca. de E. Rojas, iv.1992, E. Rojas, (INBIO), 1: vi.1991, E. Rojas, (MSCC); 1: Res. Biol. Hitoy Cerere, A.C.L.A.C. Send. Espavel, 300m, 16.ix-3.x.2000, FIT, W. Arana (AKTC); **Puntarenas:** 1: Rancho Quemado, Peninsula de Osa, 200m, ix.1992, F. Quesada, (INBIO), 1: 12–24.v.1993, A. Gutierrez, (INBIO); 1: Reserva Forestal Golfo Dulce, Est. Agujas, Golfito, Sendero Zamia, 250–250m, 11–15.xi.1999, FIT, A. Azofeifa (INBIO); 1: Est. Agujas, Sendero Zamia, 300m, 20–24.vi.1996, FIT, A. Azofeifa, (INBIO).

#### Other material.

**ECUADOR: Orellana:** 1:Yasuní Res. Stn., mid.Rio Tiputini, 0°40.5'S, 76°24'W, 5–14.vii.1999, FIT, A.K. Tishechkin (LSAM), 1: 17–23.vi.1999, FIT, C.E. Carlton & A.K. Tishechkin (LSAM), 1: 18–25.vii.1999, FIT, A.K. Tishechkin (AKTC), 1: 25–30.vii.1999, FIT, A.K. Tishechkin (LSAM), 1: 26.vii-4.viii.1999, FIT, A.K. Tishechkin (MSCC). **FRENCH GUIANA:** 1: Montagne des Chevaux, 4°43'N, 52°24'W, 22.xii.2008, FIT, SEAG (CHND); 1: Belvèdére de Saül, point de vue, 3°1'22'N, 53°12'34"W, 13.v.2011, polytrap, SEAG (CHND). **PERU: Madre de Dios:** 1: Manu National Park, Cocha Salvador, 12°0'13"S, 71°31'36"W, 310m, 20–21.x.2000, FIT, R. Brooks (CMNC); 1: Manu National Park, Rio Alto Madre de Dios, Pantiacolla Lodge, 12°39.3'S, 71°13.9'W, 14–19.xi.2007, FIT, D. Brzoska (SEMC); 1: Tambopata, Reserva Cuzco Amazonico, 15km NE Pto. Maldonado, 12°33'S, 69°03'W, 200m, 24.vi.1989, FIT, J. Ashe, R. A. Leschen, D. Silva (CHSM), 1: 26.vi.1989, FIT, D. Silva, R.A. Leschen (SEMC), 1: 13.vii.1989, FIT, J. Ashe & R. Leschen (SEMC). **SURINAME: Sipaliwini:** 1: CI-RAP Surv. Camp 1: on Kutari River, 2°10.521'N, 56°47.244'W, 228m, 19–24.viii.2010, FIT, T. Larsen & A.E.Z. Short (SEMC).

#### Diagnostic description.

Length: 2.15–2.40 mm, width: 1.75–1.90 mm; body piceous, elongate oval, widest at humeri; head with frons depressed at middle, sides of frontal stria weakly diverging between eyes, complete across front, subangulate at middle; supraorbital represented by a few fragments, disconnected from frontal stria; labrum short, shallowly emarginate apically; left mandible with very weak tooth, right with more prominent basal tooth; pronotal disk with small, irregular, but distinct prescutellar fovea; ground punctation of pronotal disk fine, inconspicuous, with ~15 coarse punctures toward sides, especially anterolaterally; marginal stria more or less complete across front, tending to be fragmented at middle; central portion of anterior margin projecting weakly; lateral submarginal stria complete, curving inward at front, ending freely behind eye; anterior submarginal stria transverse, with ends barely recurved; median pronotal gland openings laterad ends of anterior submarginal stria, about 8 puncture widths from anterior margin; elytron with one complete epipleural stria, outer subhumeral stria present in apical half and with small isolated fragment in basal half, inner subhumeral weak to absent, striae 1-3 complete, 4^th^ stria present in apical half, 5^th^ stria present in apical third, sutural stria present in apical two-thirds; elytral disk with few small punctures subserially arranged along apical margin; prosternal keel broad, shallowly emarginate at base, carinal striae converging strongly to front, joined in narrow anterior arch, free basally; prosternal lobe rather wide, with marginal stria present only near apex; mesoventrite with anterior margin sinuate, weakly projecting at middle, with marginal stria complete or very narrowly interrupted; mesometaventral stria arched forward to basal third of mesoventral disk, continuous with lateral metaventral stria which extends posterolaterally toward middle of metacoxa; central part of metaventral disk impunctate; 1^st^ abdominal ventrite with complete inner lateral stria, abbreviated outer; propygidium with ground punctation fine and sparse, with slightly elongate shallow punctures interspersed, separated by about their diameters; pygidium with fine, very dense ground punctation only, without any coarser punctures; marginal pygidial stria fine, present in about apical half, obsolete on each side to base. Male genitalia (Figs 94C–D, F, H): accessory sclerites present; T8 with sides weakly convergent in basal two-thirds, angled inward to narrow apex, faintly desclerotized at angle, apical emargination narrow, basal emargination broadly angulate, with basal membrane attachment line distad emargination by about one-sixth its depth, ventrolateral apodemes most strongly developed at middle, not meeting beneath; S8 parallel-sided in basal three-fourths, then somewhat abruptly widened to apex, guides well developed only at apex, ventrally separate from base, diverging gradually to near apex, then truncate; T9 with apices subacute, converging, opposed; T10 with halves separate; S9 with stem narrowest one-third from apex, base broad, truncate to weakly emarginate, apex with very small median emargination, apical flanges small and separate, stem markedly desclerotized at base; tegmen with sides broadly rounded, apex subacute, with narrow ‘U’-shaped medioventral process projecting weakly beneath, about one-fourth from base; basal piece about one-half tegmen length; median lobe about one-third tegmen length, with proximal apodemes separate.

#### Remarks.

**Figure 95.**
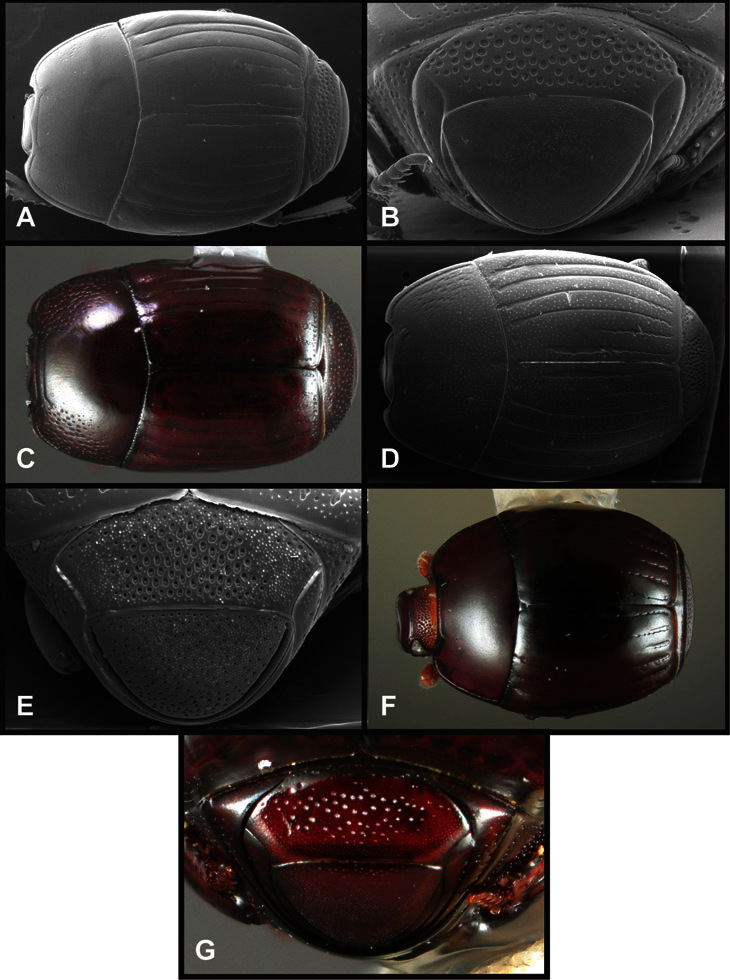
Various *Operclipygus* species. **A** Dorsal habitus of *Operclipygus peregrinus* **B** Pygidia of *Operclipygus peregrinus* **C **Dorsal habitus of *Operclipygus brooksi* **D** Dorsal habitus of *Operclipygus profundipygus* **E** Pygidia of *Operclipygus profundipygus* **F **Dorsal habitus of *Operclipygus punctatissimus* **G** Pygidia of *Operclipygus punctatissimus*.

The finely, evenly punctate pygidium (Fig. 95B) with a fine, abbreviated marginal sulcus, produced anterior pronotal margin, few apical marginal elytral punctures, and slightly shortened carinal prosternal striae together will diagnose this species. Specimens from South American localities generally have the inner subhumeral stria impressed for a short distance behind the middle, but are otherwise consistent in all characters. We restrict the type series to those specimens from Central America.

#### Etymology.

This species’ name hints at its wide distribution, from Costa Rica to French Guiana and Peru.

### Operclipygus brooksi sp. n.

urn:lsid:zoobank.org:act:B318C95F-2941-4CFB-AAF5-C451F0A01970

http://species-id.net/wiki/Operclipygus_brooksi

**Map 33.**
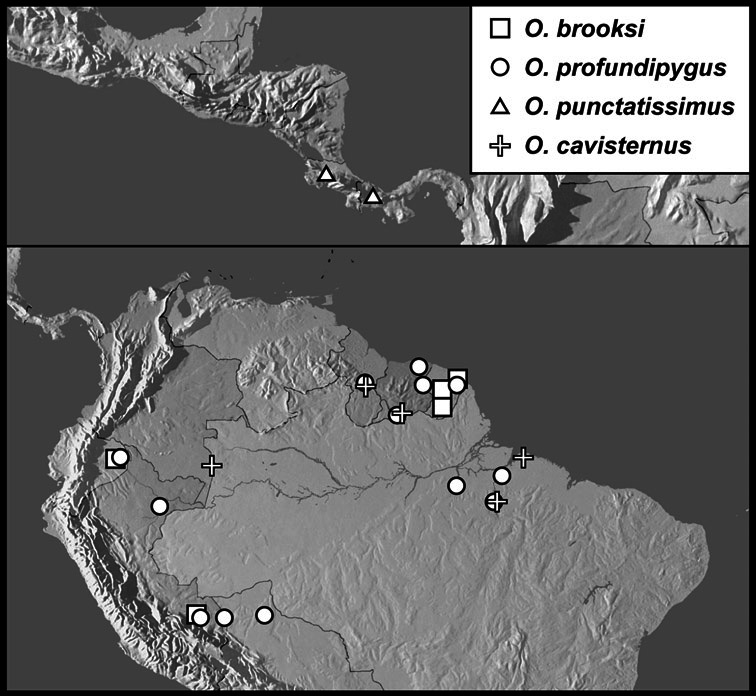
Records of the *Operclipygus* incertae sedis spp.

Figs 95C 96A–B, E, G Map 33

#### Type locality.

PERU: Madre de Dios: Manú National Park, Cocha Panchita [12°1.05'S, 71°13.08'W].

#### Type material.

**Holotype male**: “PERU: Madre de Dios, Cocha Panchita, Reserved Zone, Manu National Park, 300m, 12°1'3"S, 71°13'5"W, 21 OCT 2000; R.Brooks, PERU1B00 067, ex: gilled mushroom”/ “SM0266441 KUNHM-ENT” (SEMC). **Paratypes** (11): **ECUADOR: Orellana:** 1:Parque Nac. Yasuní, Via Maxus, Puente Piraña, 0°39.5'S, 76°"26"W, 20–24.vii.2008, FIT, A.K. Tishechkin (AKTC). **FRENCH GUIANA:** 1: Roura (39.4km SSE), 4°32'43"N, 52°8'26"W, 270m, 25–29.v.1997, FIT, J. Ashe & R. Brooks (SEMC); 1: Mont tabulaire Itoupé, 3°1.82'N, 53°6.40'W, 400m, 17.iii.2010, FIT, SEAG (CHND), 2: 600m, 29.iii.2010, FIT, SEAG (MNHN); 1: Montagne des Chevaux, 4°43'N, 52°24'W, 2.v.2009, FIT, SEAG (CHND); 1: Rés. Natur. des Nouragues, Camp Inselberg, 4°05'N, 52°41'W, 27.x.2010, FIT, SEAG (CHND), 1: 25.i.2011, FIT, SEAG (CHND); 2: Belvèdére de Saül, point de vue, 3°1'22"N, 53°12'34"W, 17.i.2011, FIT, SEAG (CHND, MSCC), 1: 13.v.2011, polytrap, SEAG (AKTC).

#### Diagnostic description.

**Figure 96.**
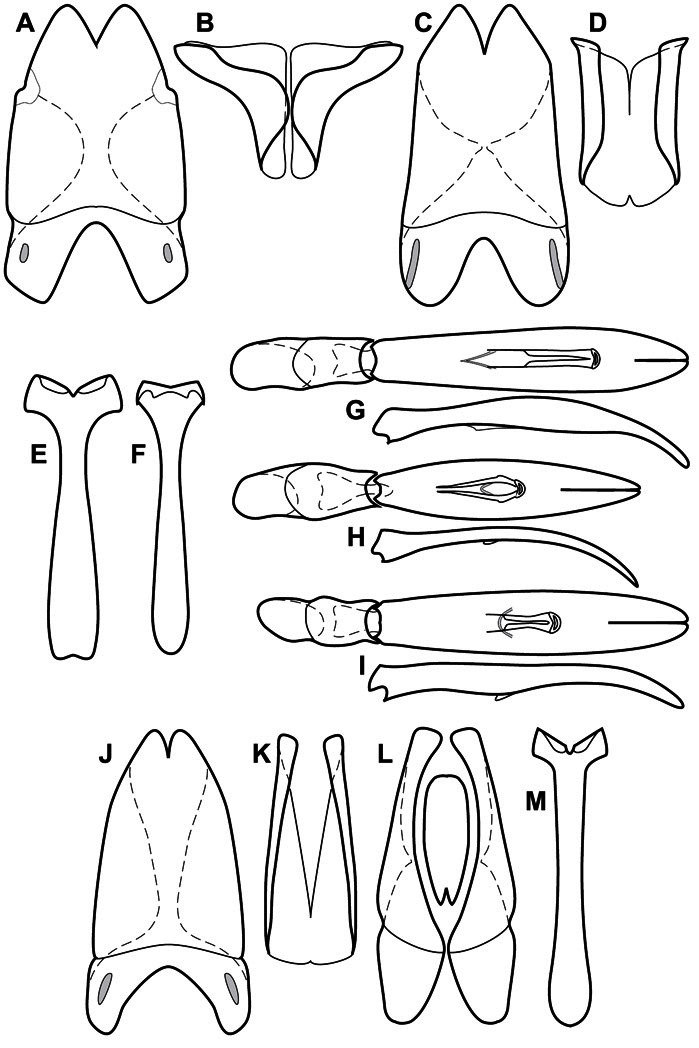
Male genitalia of various *Operclipygus* species. **A** T8 of *Operclipygus brooksi* **B** S8 of *Operclipygus brooksi* **C** T8 of *Operclipygus profundipygus* **D** S8 of *Operclipygus profundipygus* **E** S9 of *Operclipygus brooksi* **F** S9 of *Operclipygus profundipygus* **G** Aedeagus, dorsal and lateral views, of *Operclipygus brooksi* **H** Aedeagus, dorsal and lateral views, of *Operclipygus profundipygus* **I** Aedeagus, dorsal and lateral views, of *Operclipygus punctatissimus* **J** T8 of *Operclipygus punctatissimus* **K** S8 of *Operclipygus punctatissimus* **L** T9 & T10 of *Operclipygus punctatissimus* **M** S9 of *Operclipygus punctatissimus*.

Length: 2.31–2.59 mm, width: 1.65–1.87 mm; body rufo-piceous, elongate, parallel-sided to slightly ovoid; frons depressed at middle, with few small punctures, frontal stria with sides rounded, slightly divergent, sinuate above antennal bases, outwardly arcuate at middle; labrum short, apical margin truncate; left mandible untoothed, right with acute basal tooth; prothorax with sides weakly convergent to front, base of disk with weak, indistinct prescutellar impression, middle one-third of disk with only fine ground punctation, sides with numerous coarse punctures; marginal pronotal stria continuous around front; central part of anterior pronotal margin narrowly and weakly, but subacutely produced over head; lateral submarginal pronotal stria present in anterior half only, curved inward at front, ending behind eye; anterior submarginal pronotal stria recurved posterad about one-sixth pronotal length; median pronotal gland openings situated between anterior end of lateral submarginal and anterior submarginal, about 6 puncture widths from anterior margin; elytron with one complete epipleural stria, outer subhumeral stria present in posterior half only, inner subhumeral stria absent, rarely represented by few median punctures, striae 1-4 complete, finely impressed, 5^th^ stria present in apical half, sutural stria present in apical three-fourths; elytral disk with few scattered punctures near apex; prosternal keel moderately broad, shallowly emarginate at base, with carinal striae converging to front, united short distance behind presternal suture, basally connected or not; weak secondary carinal striae present in basal half; prosternal lobe narrowed evenly to hypomeron, with marginal stria present at middle, becoming fragmented laterally; anterior margin of mesoventrite sinuate, very weakly projecting at middle, marginal stria complete; mesometaventral stria arched forward just anterad mesoventral midpoint, subangulate at middle, continuous with lateral metaventral stria which extends sinuately toward outer corner of metacoxa; 1^st^ abdominal ventrite with inner lateral stria complete, outer obsolete in posterior half, central portion of disk with conspicuous, shallow punctures in anterior two-thirds, slightly denser toward coxae; propygidium nearly as long as pygidium along midline, with sparse fine ground punctation, with larger, shallow, slightly elongate punctures separated by about their diameters throughout; pygidium with very dense, fine ground punctation only, without larger punctures; marginal pygidial stria fine, completely or nearly so, barely abbreviated in anterior corners in some individuals. Male genitalia (Fig. 96A–B, E, G): accessory sclerites present, small; T8 with sides straight in basal three-fourths, abruptly narrowed, convergent to apex, with desclerotized areas at lateral angle, basal emargination deep, bluntly triangular, nearly reaching basal membrane attachment line, apical emargination fairly broad, ventrolateral apodemes most strongly developed at middle, evenly narrowed to base and apex, not meeting at midline; S8 abruptly divergent and downturned in apical half, apical guides enclosing a broad apical velum; T9 with sides subparallel in basal third, convergent to apex, with apices subacute, inturned, nearly meeting; T10 with halves separate; S9 with sides subparallel in basal half, base rounded, finely emarginate at middle; apex with small median emargination, apical flanges separate; tegmen widest one-fourth from apex, sides rounded to subacute apex, apical emargination shallow; medioventral process elongate, ‘V’-shaped, with acute apex weakly projecting beneath about one-third from base; basal piece nearly one-half tegmen length; median lobe about one-half tegmen length, with basal filamentous portion of proximal apodemes about half of overall length.

#### Remarks.

The strong punctation of the lateral third of the pronotum (Fig. 95C) along with the abbreviated lateral submarginal pronotal stria, coarse punctures on the 1^st^ abdominal ventrite, and the weakly projecting anterior pronotal margin will distinguish this species. The species is surprisingly widespread, but conforms in external and in its distinctive genitalic characters well throughout the range.

#### Etymology.

This species is named for Rob Brooks who, during his time at the University of Kansas, helped collected numerous interesting Histeridae. In the case of this species he collected specimens from the extremes of the range, in both Peru and French Guiana.

### Operclipygus profundipygus sp. n.

urn:lsid:zoobank.org:act:E9D13FFA-0FE2-4C58-8A71-32301FC8B8C7

http://species-id.net/wiki/Operclipygus_profundipygus

Figs 95D–E 96C–D, F, H Map 33

#### Type locality.

GUYANA: Region 8: 1 km W Kurupukari, Iwokrama Field Station [4°40.32'N, 58°41.07'W].

#### Type material.

**Holotype male**: “GUYANA: Region 8, Iwokrama Forest, 1 km W Kurupukari, Iwokrama Forest Field Stn., 60 m, 4°40'19"N, 58°41'4"W, 20–25 MAY 2001 R. Brooks,Z.Falin GUY1BF01 034 ex: flight intercept trap”/ “SM0566205 KUNHM-ENT” (SEMC). **Paratypes** (29): **BRAZIL: Pará:** 4: Abaetetuba, 1°42'S, 48°54'W, 26.xi–7.xii.1993, FIT (CEMT, FMNH); 1:Altamira - Marabá: km 18., 3°09'S, 52°03'W, v.1985, FIT (CHND); 1: Tucuruí, 3°45'S, 49°40'W, 6–17.vi.1986, FIT (CHND). **GUYANA: Region 8**: 2: Iwokrama Field Stn., Iwokrama Forest, 1km W Kurupukari, 4°40'19"N, 58°41'4"W, 60m, 21.v.2001, *Acromyrmex* refuse pile, R. Brooks & Z. Falin (SEMC), 3: 26–29.v.2001, FIT, R. Brooks & Z. Falin (SEMC), 1: 20–25.v.2001, FIT, R. Brooks & Z. Falin (SEMC); 5: Kurupukari, 4°40'N, 58°40'W, ix-xi.1992, malaise/FIT (BMNH). **FRENCH GUIANA:** 1: Montjoly, 4°55'N, 52°16'W, xii.1977, banana trap (CHND). **SURINAME: Marowijne**: 1: Perica, 70km E. Paramaribo on East-West Road, 5°40'28"N, 54°36'31"W, 5m, 29–31.v.1999, FIT, Z. Falin, B. DeDijn (SEMC), 3: 31.v–5.vi.1999, FIT, Z. Falin, B. DeDijn (SEMC), 2: 5m, 31.v.1999, FIT, Z. Falin (MSCC, AKTC); **Sipaliwini**: 2: CI-RAP Surv. Camp 1: on Kutari River, 2°10.521'N, 56°47.244'W, 228m, 19–24.viii.2010, FIT, T. Larsen & A.E.Z. Short (SEMC); 2: upper Palumeu, 225m, 2.47700°N, 55.62941°W, CI-RAP Survey camp 1, FIT, 10–16.iii.2012, A.E.Z. Short (MSCC); 1: Wanaboo (near Nason), Marowijne River, 4°43'35"N, 54°26'36"W, 40m, 31.v–5.vi.1999, FIT, Z. Falin, B. DeDijn (SEMC); **Wanica**: 1: Paramaribo, 12km S. on I. Ghandiweg, and 4.5km W on Mijnzorgweg, 5°43'55"N, 55°15'7"W, 10m, 6–7.vi.1999, FIT, Z. Falin, B. DeDijn (SEMC).

**Other material. BOLIVIA: Beni**: 1: Chacobo Indian Village on Rio Benicito, 12°20'S, 66°W, 18–27.vii.1960, under tapir dung, B. Malkin (FMNH), 1: 31.vii-2.viii.1960, B. Malkin (FMNH). **ECUADOR: Orellana:** 1:Yasuní Res. Stn., 00°40'28"S, 76°38'50"W, 215m, 5–10.ix.1999, FIT, Primary forest, E.G. Riley (LSAM); 1: P.N. Yasuní, Via Maxus at Puente Piraña, 0°39.5'S, 76°26'W, 20–24.vii.2008, FIT, A.K. Tishechkin (AKTC). **PERU: Loreto:** 3: Iquitos, 90m, 7.v.1992, FIT, J. Danoff-Berg (SEMC); **Madre de Dios:** 1: Amazonas Lodge, N Atalaya, 12°52.2'S, 71°22.6'W, 480m, 10–13.xi.2007, FIT, D. Brzoska (SEMC); 2: Tambopata, Reserva Cuzco Amazonico, 15km NE Pto. Maldonado, 12°33'S, 69°03'W, 200m, 16.vii.1989, FIT, J. Ashe & R. Leschen (SEMC), 2: 13.vii.1989, FIT, J. Ashe & R. Leschen (SEMC), 1: 20.vi.1989, FIT, J. Ashe & R. Leschen (SEMC), 1: 22.vi.1989, FIT, J. Ashe & R. Leschen (CHSM), 1: 26.vi.1989, FIT, J. Ashe & R. Leschen (CHSM); 1: Manu National Park, Cocha Salvador, 12°0'13"S, 71°31'36"W, 310m, 20–21.x.2000, FIT, R. Brooks (SEMC).

#### Diagnostic description.

Length: 1.75–1.90 mm, width: 1.47–1.59 mm; body rufo-brunneus, elongate oval, widest at humeri, prothorax rather broad, narrowing more conspicuously posterad; frons weakly depressed at middle, often with microsculpture within depression; frontal stria with sides divergent between eyes, broadly outwardly arcuate across front, continuous with complete supraorbital stria; labrum about two-thirds as long as broad, apical margin straight; left mandible untoothed, right with acute basal tooth; pronotum with sides weakly arcuate, convergent; pronotal disk with distinct narrow, sublinear prescutellar impression; pronotal disk with ground punctation fine, with ~15-20 coarser punctures toward sides; lateral marginal pronotal stria complete at side, ending just mesad anterior corner; lateral submarginal stria complete at side, extending forward to anterior margin, replacing marginal for a short distance, ending behind eye, slightly overlapping end of anterior submarginal stria, which is briefly recurved posterad at sides; central portion of anterior pronotal margin weakly projecting; median pronotal gland openings situated laterad ends of anterior submarginal stria; elytron swollen laterad 1^st^ dorsal stria, with one complete epipleural stria, outer subhumeral in posterior half to two-thirds, inner subhumeral stria absent, striae 1-4 complete, 5^th^ stria present in apical half and with a large basal puncture; sutural stria present in apical three-fourths, widened toward base; prosternal keel rounded, produced at base, with carinal striae sinuately convergent to front, united just short of presternal suture; prosternal lobe with marginal stria more or less complete; mesoventrite emarginate anteriorly, with complete marginal stria; mesometaventral stria arched forward just beyond mesoventral midpoint, continued at sides by lateral metaventral stria, which extends toward middle of metacoxa; 1^st^ abdominal ventrite with two complete lateral striae on each side; propygidium with fine, dense ground punctation at sides, with small, deep, coarse punctures densely clustered in middle two-thirds; pygidium with fine, dense ground punctures throughout, with slightly larger punctures generally sparse, but concentrated along basal margin and toward apex of disk; apical marginal sulcus complete, deep and coarse. Male genitalia (Figs 96C–D, F, H): accessory sclerites present; T8 with sides convergent from base to apex, basal emargination rather narrowly angulate, basal membrane attachment line distad by about half basal emargination's depth, apical emargination narrow, ventrolateral apodemes subacute ventrally, nearly meeting at middle; S8 with apical guides well developed, consistent in width throughout length, apices very slightly divergent, ventral halves fused for most of their length; T9 with apices narrow, slightly convergent; T10 with halves separate along midline; S9 rather narrow, weakly widened to rounded base, apex concave, but without median emargination, apical flanges continuous; tegmen short with sides strongly rounded, narrow at base, medioventral process weak, ‘U’-shaped, projecting beneath just basad midpoint; basal piece nearly as long as tegmen; median lobe about half length of tegmen, with basal apodemes widely separate at gonopore, convergent toward tegmen base.

#### Remarks.

The pattern of propygidial punctation (Fig. 95E), with dense ground punctation visible at sides, while larger punctures are concentrated in the middle is unusual, although there are a few distantly related species that share it. Of these, this one alone has a distinct prescutellar impression (Fig. 95D). We restrict the type series to those specimens from the Guianas and northeastern Amazonian region, though there is little evident variation throughout the range.

#### Etymology.

The name of this species refers to both the deep pygidial sulcus and the deeply impressed median propygidial punctures.

### Operclipygus punctatissimus sp. n.

urn:lsid:zoobank.org:act:D9341C4E-3A15-453F-A389-FFC5A383518B

http://species-id.net/wiki/Operclipygus_punctatissimus

Figs 95F–G 96I–M Map 33

#### Type locality.

PANAMA: Chiriquí: La Fortuna Station [08°46'N, 82°12'W].

#### Type material.

**Holotype male**: “PANAMA: Chiriquí Prov. La Fortuna, “Cont. Divide Trail”, 08°46'N, 82°12'W, 1100m, 23 V-9 VI 1995 J. Ashe,R. Brooks #157, ex: flight intercept trap” / “SEMC0903623” (SEMC). **Paratypes** (2): **COSTA RICA: Alajuela:** 1: Est.Biol. San Ramon, Reserva Biol. San Ramon, 27km. N. & 8km. W. San Ramon, 10°13'4"N, 84°35'46"W, 810m, 8.vii.2000, FIT, J. Ashe, R. Brooks, Z. Falin (SEMC); 1: Peñas Blancas, 970m, 20.v.1989, FIT, J. Ashe, R. Leschen, R. Brooks (INBIO).

#### Diagnostic description.

Length: 2.03–2.18 mm, width: 1.81–1.90 mm; body rufo-piceous, sides rounded, widest near middle of elytra, strongly convex; frons rather narrow, weakly depressed at middle, frontal stria with sides rounded, diverging slightly between eyes, anterior portion evenly and strongly outwardly arcuate; supraorbital stria represented by a few detached fragments; epistoma convex; labrum about two-thirds as long as wide, weakly emarginate apically; left mandible untoothed, right with acute basal tooth; pronotal disk with fine, indistinct punctiform prescutellar impression, disk with fine, sparse ground punctures only, lacking coarser punctures to sides; marginal stria interrupted behind head; lateral submarginal stria curved inward along anterior margin, ending very close to recurved end of anterior submarginal stria; median pronotal gland openings posterad recurved ends of anterior stria, about one-fourth pronotal length from anterior margin; elytron with one complete epipleural stria markedly crenulate in basal half, outer subhumeral stria present in apical half only, inner subhumeral stria absent, striae 1-3 complete, 4^th^ and 5^th^ striae subequal, short, present only in apical fourth, sutural stria present in apical three-fourths; prosternum broad, base of keel very faintly emarginate, carinal striae sinuate, barely converging, united in broad anterior arch; prosternal lobe with complete marginal stria; mesoventral margin sinuate but not distinctly projecting at middle, marginal stria interrupted for width of keel; mesometaventral stria strongly, broadly arched forward, displacing marginal mesoventral at middle, continuous at sides with lateral metaventral stria which extends toward outer third of metacoxa; metaventral disk impunctate, convex; 1^st^ abdominal ventrite with single lateral stria, which is complete, arcuate, curving mediad toward posterior margin; propygidium with small but deep punctures, separated by about 1.5× their diameters, concentrated in a central cluster, with narrow impunctate borders along all edges; pygidium rather flat, with fine, moderately dense ground punctures only, no coarser punctures; apical marginal stria completely absent. Male genitalia (Figs 96I–M): accessory sclerites present; T8 elongate, narrow, with sides evenly, arcuately converging to narrow apices, basal apodemes narrowly rounded, basal emargination deep and broad, basal membrane attachment line distad emargination by about one-fourth its depth, apical emargination narrow, ventrolateral apodemes of T8 most strongly developed near base, divergent to apex; S8 narrow, with sides convergent, apical guides narrow throughout, narrowly rounded apically, ventral halves fused in basal third, divergent to apex; T9 parallel-sided in basal half, convergent to relatively thick, rounded apices; T10 halves fused along midline; S9 with stem narrowest at middle, widened to rounded base, apex with small median emargination, with weak, separate apical flanges; tegmen with sides weakly rounded, widest just distad midpoint, apices rounded, medioventral process rather narrowly sclerotized, but strong, with subacute apex projecting prominently beneath near midpoint of tegmen; basal piece just over one-third tegmen length; median lobe about one-half tegmen length, proximal apodemes with filamentous parts conspicuous, nearly as long as thicker parts.

#### Remarks.

This small species is quite distinctive in its combination of fine, dense pygidial punctation (which nonetheless appears relatively smooth) and complete lack of marginal pygidial stria (Fig. 95G). The small propygidal punctures which are removed from its margins are also unusual, and helpful in identification.

#### Etymology.

The name of this species refers to the very fine, dense punctation of the pygidium.

### Operclipygus cavisternus sp. n.

urn:lsid:zoobank.org:act:AF381AF9-0A22-4240-B6E6-B985B86DB451

http://species-id.net/wiki/Operclipygus_cavisternus

**Figure 97.**
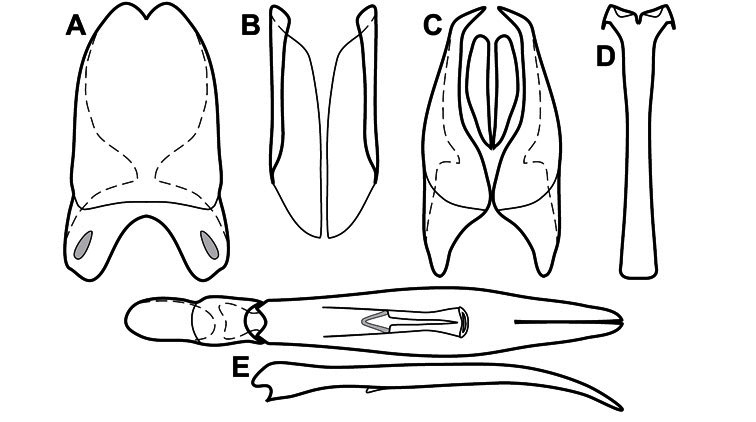
Male genitalia of *Operclipygus cavisternus*. **A** T8 **B** S8 **C** T9 & T10 **D** S9 **E** Aedeagus, dorsal and lateral views.

Figs 97A–E 98A–C Map 33

#### Type locality.

GUYANA: Region 8: 1 km W Kurupukari, Iwokrama Field Station [4°40.32'N, 58°41.07'W].

#### Type material.

**Holotype male**: “GUYANA: Region 8, Iwokrama Forest, 1 km W Kurupukari, Iwokrama Forest Field Stn., 60 m, 4°40'19"N, 58°41'4"W, 20–25 MAY 2001 R. Brooks,Z.Falin GUY1BF01 034 ex: flight intercept trap”/ “SM0565892 KUNHM-ENT” (SEMC). **Paratypes** (3): **GUYANA: Region 8**: 1: Iwokrama Field Stn., Iwokrama Forest, 1km W Kurupukari, 4°40'19"N, 58°41'4"W, 60m, 20–25.v.2001, FIT, R. Brooks & Z. Falin (SEMC). **SURINAME: Sipaliwini:** 1: CI-RAP Surv. Camp 3: Wehepai SE Kwamala, 2°21.776'N, 56°41.861'W, 237m, 3–7.ix.2010, FIT, T. Larsen & A.E.Z. Short (SEMC); 1: upper Palumeu, 225m, 2.47700°N, 55.62941°W, CI-RAP Survey camp 1, FIT, 10–16.iii.2012, A.E.Z. Short (MSCC).

#### Other material.

**BRAZIL: Pará:** 3: IPEAN, Belém, Utinga, 1°27'S, 48°26'W, viii.1985, FIT (CHND, MNHN, AKTC); 1: Tucuruí, 3°45'S, 49°40'W, 24.iii-11.iv.1985, FIT (CHND). **COLOMBIA: Vaupés:** 1: Parque Nac. Mosiro-Itajura (Caparú), Centro Ambiental, 1°04'S, 69°31'W, 60m, 20–30.i.2003, FIT, D. Arias & M. Sharkey (IAVH).

#### Diagnostic description.

Length: 1.78–1.84 mm, width: 1.50–1.53 mm; body rufo-brunneus, broadly oval, widest near elytral midpoint; frons only very weakly depressed at middle, frontal stria rounded at sides, weakly sinuate over antennal bases, weakly arcuate across front; epistoma convex anteriorly; labrum about twice as wide as long, shallowly emarginate along apical margin; pronotal disk with small, shallow, elongate prescutellar impression, fine sparse ground punctation medially, and moderately numerous (~12) coarser, shallow punctures toward sides; marginal pronotal stria interrupted behind head; lateral submarginal stria complete, curved inward at front, nearly reaching weakly recurved ends of anterior submarginal stria; lateral marginal bead of pronotum convex; median pronotal gland openings situated just beyond recurved ends of anterior stria, about 6-8 puncture widths from margin; elytron with one complete epipleural stria, sinuate at middle and crenulate throughout its length, outer subhumeral stria interrupted at middle, rarely with short basal fragment, inner subhumeral stria complete, striae 1-3 complete, 4^th^ stria present in apical half, 5^th^ stria present in apical third, sutural stria present in apical three-fourths; prosternal keel rather broad, flat, very weakly produced at base, carinal striae complete, weakly converging, connected in anterior arch; pronotal lobe with marginal stria complete; anterior mesoventral margin shallowly emarginate at middle, marginal stria complete; mesometaventral stria weakly arched forward, reaching to about basal third of mesoventral disk, continued at sides by lateral metaventral stria posterolaterad toward rear corner of metepisternum; metaventral disk impunctate, with paired, broad, shallow depressions near posterior margin; 1^st^ abdominal ventrite with two complete lateral striae; propygidium with fine, sparse ground punctation, uniformly covered with larger punctures separated by slightly less than their diameters; pygidium with coarse, fine ground punctation and larger punctures fairly coarsely interspersed; marginal pygidial sulcus complete, even, fine but moderately deep. Male genitalia (Figs 97A–E): accessory sclerites present, somewhat reduced in size; T8 with sides evenly convergent to near apex, apices broadly rounded, basal apodemes rounded, basal emargination broad, deep, nearly reaching basal membrane attachment line, ventrolateral apodemes most strongly developed near base, diverging to apex, not meeting at midline; S8 elongate, with sides subparallel, apical guides narrow, even throughout length, apices narrowly rounded, halves separate, weakly divergent to apex; T9 with apices weakly enlarged near tip, inturned, subacute; T10 with halves separate; S9 stem narrow, barely widened to narrowly rounded base, apex with narrow median emargination, apical flanges low, separate; tegmen widest at middle, sides rather straight, converging to base and apex, apices narrowly rounded, weakly curved ventrad in apical half, medioventral process narrow, ‘V’-shaped, projecting beneath about one-fourth from base; basal piece about one-third tegmen length; median lobe nearly one-half tegmen length, proximal apodemes separate.

#### Remarks.

**Figure 98.**
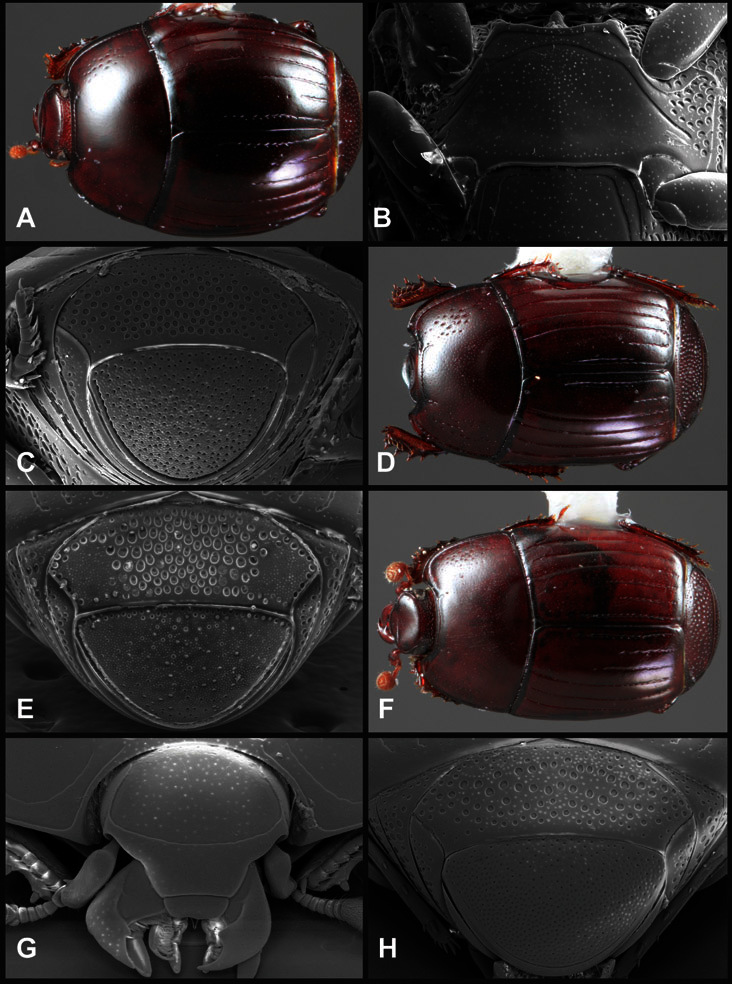
Various *Operclipygus* species. **A** Dorsal habitus of *Operclipygus cavisternus* **B** Ventral habitus of *Operclipygus cavisternus* **C** Pygidia of *Operclipygus cavisternus* **D** Dorsal habitus of *Operclipygus parallelus* **E** Pygidia of *Operclipygus parallelus* **F** Dorsal habitus of *Operclipygus abbreviatus* **G** frons of *Operclipygus abbreviatus* **H** Pygidia of *Operclipygus abbreviatus*.

The shallow depressions of the metaventrite of this species (Fig. 98B), although shallow and not very conspicuous, are unusual and distinctive (and not limited to either sex, as such depressions may sometimes be in Exosternini). This character, in combination with small size, complete, fine, deep pygidial sulcus (Fig. 98C), and complete inner subhumeral stria will distinguish the species. We limit the type series to specimens from the Guianas, which all have a complete frontal stria. Slight variation in specimens from other areas should be investigated more fully.

#### Etymology.

This species’ name refers to the distinctive impressions on either side near the posterior margin of the metaventrite.

### Operclipygus siluriformis sp. n.

urn:lsid:zoobank.org:act:27C7F361-F43A-4D4F-B1EE-20B1E6F050A7

http://species-id.net/wiki/Operclipygus_siluriformis

Figs 99A–E Map 34

#### Type locality.

BRAZIL: Mato Grosso:Fazenda São Nicolau [9°50.3'S, 58°15.05'W].

#### Type material.

**Holotype male**: “**BRASIL: Mato Grosso:** Mpio Cotriguaçu, Fazenda São Nicolau, Matinha. 9°50.3'S, 58°15.05'W. Flight intercept Oct 2009. F.Z.Vaz-de-Mello” / “Caterino/Tishechkin Exosternini Voucher EXO-00985” (CEMT). **Paratypes** (10): 10: same data as type, except as noted: 4: xii.2010 (CEMT, FMNH, UFPR, MSCC, AKTC); 3: Cotriguaçu, Fazenda São Nicolau, Prainha, 9°51.6'S, 58°12.9'W, x.2009 (FMNH); 3: Cotriguaçu, Fazenda São Nicolau, Mata Norte, 9°49.15'S, 58°15.6'W, 8–14.xii.2010, FIT, F.Z. Vaz-de-Mello (MSCC, AKTC).

**Other material. BRAZIL: Pará:** 2: IPEAN, Utinga, Belém, 1°27'S, 48°26'W, viii.1985, FIT (CHND), 5: x.1984, FIT (CHND), 1: xi.1984, FIT (CHND), 1: x.1985, FIT (CHND); 1: Belém, 1°3.1'S, 48°8.8'W, 27.vii.1984 (CHND); 9: Carajás, Serra Norte, 6°04'S, 50°12'W, xi.1984, FIT (CHND); 1: Altamira - Marabá: km 18., 3°09'S, 52°03'W, 10–23.ix.1985, FIT (CHND); 1: Barcarena, 1°30'S, 48°37'W, 19–30.ix.1991, FIT (CHND); 1: Melgaço, Rio Marinaú, 1°51'S, 51°20'W, 31.x–13.xi.1993, FIT (CHND); 7: Tucuruí, 3°45'S, 49°40'W, 6–17.vi.1986, FIT (CHND). **PERU: Junín:** 1: 11km NE Puerto Ocopa, Los Olivos, 11°7.00'S, 74°15.52'W, 1200m, 30–31.iii.2009, Window trap, A.V. Petrov (AKTC).

#### Diagnostic description.

Length: 2.22–2.50 mm, width: 1.87–2.03 mm; body rufo-brunneus, elongate, nearly parallel-sided, sides of elytra weakly arcuate; frons moderately depressed behind frontal stria; frontal stria with sides divergent between eyes, sinuate above antennal bases, continuous, arcuate across front; supraorbital stria complete, meeting sides of frontal stria, area enclosed by frontal and supraorbital striae rather short; labrum about twice as wide as long, rather flat, apical margin shallowly emarginate; left mandible with short, blunt basal tooth, right mandible with more pronounced, subacute tooth; prothorax with disk vaguely and weakly impressed in prescutellar region, very fine, sparse ground punctation, relatively narrow band of ~10 punctures mediad lateral submarginal stria; marginal pronotal stria absent along most of anterior pronotal margin, complete lateral submarginal stria replacing it for a short distance at sides; marginal pronotal bead strongly convex, widening slightly toward the front; anterior submarginal stria weakly recurved posterad at sides; median pronotal gland openings anterolaterad ends of anterior submarginal stria, about three puncture widths from anterior margin; elytra with one complete epipleural stria, outer subhumeral stria complete, inner subhumeral stria absent, striae 1-4 complete, 5^th^ stria present in apical third, sutural stria present in apical three-fourths, the sutural stria broadening toward base; prosternal keel with base rounded, produced strongly into mesoventrite, carinal striae complete, convergent from base, parallel in anterior half, united by narrow arch; prosternal lobe with complete marginal stria; mesoventral margin emarginate, with marginal stria interrupted; mesometaventral stria arched forward to anterior third of mesoventral disk, continued by lateral metaventral stria posterad toward middle of metacoxa; lateral part of metaventral disk with oblique secondary lateral stria; 1^st^ abdominal ventrite with two complete lateral striae, parallel throughout their lengths; propygidium with fine, dense ground punctation at sides, in middle with slightly elongate, rather deep punctures separated by less than half their diameters, larger punctures also present along entire anterior and lateral margins of propygidium; pygidium with ground punctation as on sides of propygidium, with some larger punctures sparsely interspersed; marginal pygidial sulcus deep, complete, crenulate on inner and outer edges. Male: accessory sclerites absent; T8 with sides convergent from base to apex, apices truncate, apical emargination narrow, basal emargination shallow, nearly reaching basal membrane attachment line, ventrolateral apodemes most strongly developed near base, divergent to apex; S8 with sides with sides slightly divergent, apical guides narrow, gradually widened to apex, ventral halves approximate in basal half, divergent apically; T9 parallel-sided in basal two-thirds, convergent to apex, apices widened inward, apically truncate; T10 weakly sclerotized along midline, margins indistinct; S9 stem fairly wide, narrowest near apex, widened to broadly rounded base, with distinct apical emargination, apical flanges small and separate; tegmen stout, widest at middle, evenly narrowed to base and apex, apices broad, appearing truncate from above, with membraneous ventrolateral processes extending from apical corners; medioventral process not at all evident; basal piece almost half tegmen length; median lobe over half tegmen length, with wide gonopore, proximal apodemes strongly differentiated into short, broad, separated bases, and long, well sclerotized extensions reaching to base of tegmen.

#### Remarks.

**Figure 99.**
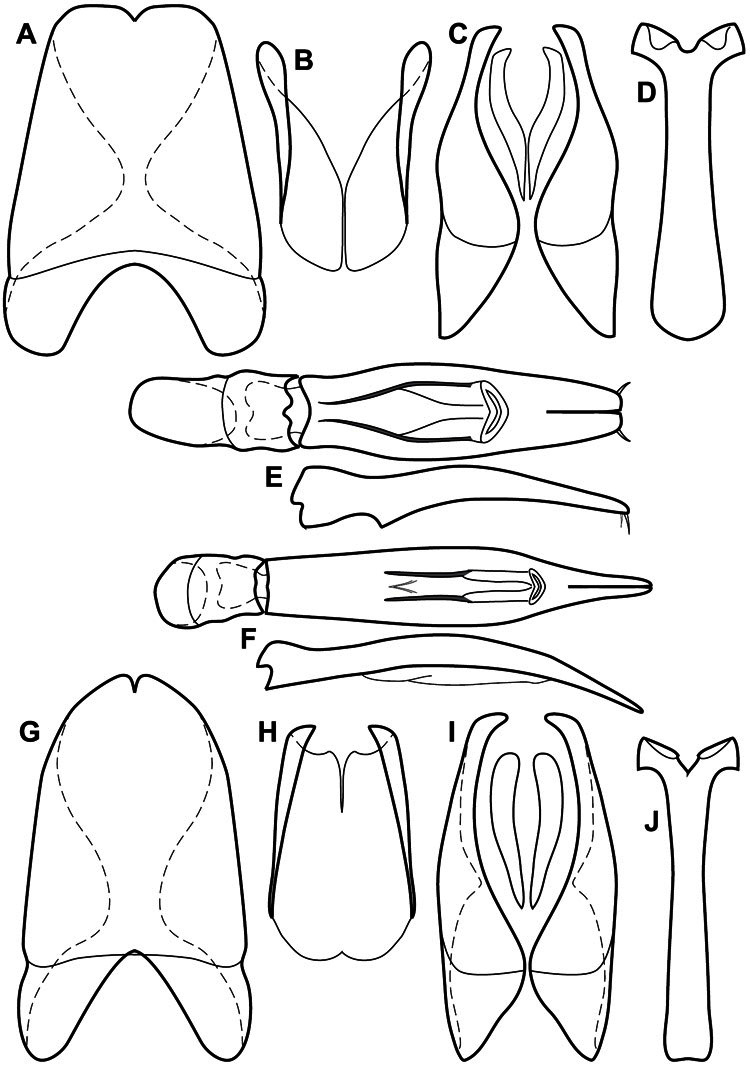
Male genitalia of *Operclipygus* species. **A** T8 of *Operclipygus siluriformis* **B** S8 of *Operclipygus siluriformis* **C** T9 & T10 of *Operclipygus siluriformis* **D** S9 of *Operclipygus siluriformis* **E** Aedeagus, dorsal and lateral views, of *Operclipygus siluriformis* **F** Aedeagus, dorsal and lateral views, of *Operclipygus parallelus* **G** T8 of *Operclipygus parallelus* **H** S8 of *Operclipygus parallelus* **I** T9 & T10 of *Operclipygus parallelus* **J** S9 of *Operclipygus parallelus*.

This species and the following, *Operclipygus parallelus*, are very similar and distinctive externally, both being relatively elongate (Fig. 98D), having a complete outer subhumeral stria, and a propygidium with fine dense punctures on either side of a more coarsely punctate central area (Fig. 98E). This type of propygidum can be found in a few other species, but these species are larger and more distinctly parallel-sided than any others. Their genitalia (Fig. 99) are remarkably different given their external similarity, but they do share a few important characters, and appear to be related: they lack accessory sclerites at the base of T8; apices of T9 are widened and appearing obliquely truncate; the inner edges of T10 are desclerotized and separate; apex of S9 emarginate, with separate apical flanges; median lobe relatively short, but with a large gonopore and elongate basal apodemes with strongly sclerotized basal stems. The tegmen of the *Operclipygus siluriformis* is remarkable (Fig. 99E), with a broad truncate apex bearing whisker-like apical processes, which do not appear to be setae. It lacks an articulated medioventral process, although the tegmen itself has a basoventral cusp. Externally very few characters separate *Operclipygus siluriformis* and *Operclipygus parallelus*. The body of *Operclipygus siluriformis* is slightly more rounded at the sides, the marginal bead of the pronotum is more distinctly and strongly widened to the front, the sides of the metaventral disk are less strongly rugose, it lacks a basal puncture at the 5^th^ dorsal stria, and the labrum is always weakly emarginate, rather than outwardly rounded. Similar apical aedeagal processes are also seen in *Operclipygus punctipleurus*, but that species shares few other characters, and does not appear to be closely related.

We restrict this type series to specimens from the same locality as the holotype. Considerable variation throughout the range, in combination with the existence of an extremely similar, partially sympatric species urges conservatism. At the same time it is worth noting that individuals from Para, Brazil, and even the geographical outlier from Peru, were dissected to confirm the presence of the highly distinctive aedeagus.

#### Etymology.

The name is derived from the catfish suborder Siluriformes, and refers to the whisker-like appendages at the apex of the aedeagus.

### Operclipygus parallelus sp. n.

urn:lsid:zoobank.org:act:90F1E8CF-0D56-49DB-8D46-B89DC74A83FC

http://species-id.net/wiki/Operclipygus_parallelus

**Map 34.**
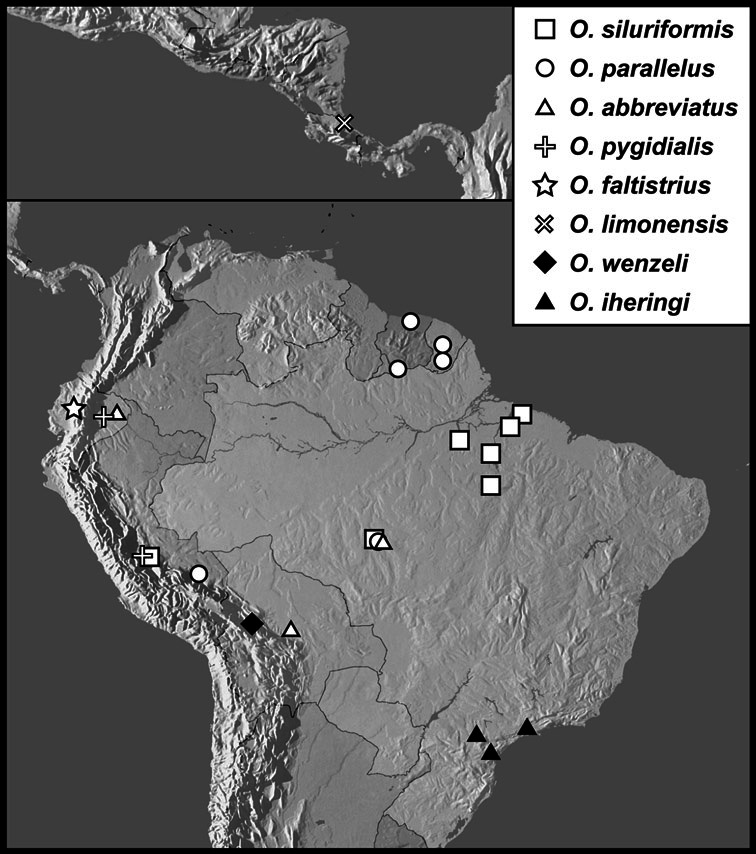
Records of the *Operclipygus* incertae sedis spp.

Figs 98D–E 99F–J Map 34

#### Type locality.

FRENCH GUIANA: Itoupé Table Mountain [3°1.38'N, 53°5.73'W].

#### Type material.

**Holotype male**: “**GUYANE FR.** Mont Tabulaire, Itoupé. 3°1.38'N, 53°5.73'W. 570m. Piège d’interception 24 Mar 2010. SEAG leg.”/ “Caterino/Tishechkin Exosternini Voucher EXO-01311” (MNHN). **Paratypes** (6): 1: same data as type MNHN; 3: same data as type; except as noted: 1: 17.iii.2010, FIT, SEAG (CHND), 2: 800m, 17.iii.2010, FIT, SEAG (CHND); 1: Rés. Natur. des Nouragues, Camp Inselberg, 4°05'N, 52°41'W, 20.viii.2010, FIT, SEAG (AKTC); 1: Belvèdére de Saül, point de vue, 3°1'22"N, 53°12'34"W, 13.v.2011, polytrap, SEAG (MSCC).

#### Other material.

**BRAZIL: Mato Grosso:** 1: Cotriguaçu, Fazenda São Nicolau, Matinha, 9°50.3'S, 58°15.05'W, xii.2009, FIT, F.Z. Vaz-de-Mello (CEMT); 1: xii.2010, FIT, F.Z. Vaz-de-Mello (UFPR); 1: Cotriguaçu, Fazenda São Nicolau, 9°48.9'S, 58°17.15'W, 15–18.xii.2010, FIT, F.Z. Vaz-de-Mello & A.F. Oliveira (FMNH); 1: Cotriguaçu, Fazenda São Nicolau, Mata Norte, 9°49.15'S, 58°15.6'W, 8–14.xii.2010, FIT, F.Z. Vaz-de-Mello (CEMT). **PERU: Madre de Dios:** 1: Amazonas Lodge, N Atalaya, 12°52.2'S, 71°22.6'W, 480m, 10–13.xi.2007, FIT, D. Brzoska (SEMC). **SURINAME: Sipaliwini**: 1: upper Palumeu, 225m, 2.47700°N, 55.62941°W, CI-RAP Survey camp 1, FIT, 10–16.iii.2012, A.E.Z. Short (MSCC); **Para**: 1: nr. Overbridge, 5°31'10"N, 55°4'10"W, 10–14.ii.2010, FIT, W. Warner (WBWC), 2: 14–18.ii.2010 (WBWC).

#### Diagnostic description.

This species is extremely similar to the preceding in external characters, and is only described to the degree to which they differ here: length: 1.78–2.40 mm, width: 1.31–1.62 mm; body strongly elongate-oval; labrum narrowed to front, apical margin carinate and outwardly arcuate; punctiform prescutellar impression fairly evident in most individuals; marginal pronotal bead not strongly widened toward front, submarginal stria more or less parallel to margin; median pronotal gland openings further posterad, somewhat variable, generally 4-6 puncture widths from anterior margin; 5^th^ dorsal elytral stria present in apical third, plus with distinct basal point near scutellum; marginal mesoventral stria very narrowly interrupted or complete at middle; 1^st^ abdominal ventrite with two lateral striae complete, tending to converge toward front. Male genitalia (Figs 99F–J): accessory sclerites absent; T8 with sides convergent from base to apex, apices broadly rounded, apical emargination narrow, basal emargination narrow, its apex tangential to basal membrane attachment line, ventrolateral apodemes most strongly developed near base, divergent to apex; S8 with sides with sides parallel, apical guides narrow, weakly widened to apex, ventral halves fused for most of their length, narrowly then abruptly divergent apically; T9 parallel-sided in basal two-thirds, convergent to apex, apices widened inward, convergent, apically truncate; T10 weakly sclerotized along midline, margins indistinct; S9 stem narrowest near apex, widened to broad subtruncate base, with distinct apical emargination, apical flanges widely separate; tegmen widest two thirds from base, sides arcuate to base, attenuate to subacute apex, medioventral process weak, almost invisible in dorsal view, narrowly ‘V’-shaped, embedded in broad, weakly sclerotized ventral expansion; basal piece one-third tegmen length; median lobe about two-thirds tegmen length, with wide gonopore, proximal apodemes strongly differentiated into short, broad, separated bases, and long, well sclerotized extensions reaching toward base of tegmen.

#### Remarks.

See the preceding species treatment for detailed diagnosis of these two very similar species. We restrict the type series of this species to specimens from French Guiana, though specimens from other localities are consistent in most diagnosticcharacters.

#### Etymology.

This species’ name refers to its strongly parallel-sided body.

### Operclipygus abbreviatus sp. n.

urn:lsid:zoobank.org:act:8C26E8F0-54CA-45AE-9ADA-EB087744BC32

http://species-id.net/wiki/Operclipygus_abbreviatus

Figs 98F–H 100A–E Map 34

#### Type locality.

ECUADOR: Orellana: Yasuní Research Station [0°40.5'S, 76°24'W].

#### Type material.

**Holotype male**: “**Ecuador:** Napo, mid.Rio Tiputini, Yasuní Res. Stn. 0°40.5'S, 76°24'W, FIT #3, 18–23 June 1999. AKT#016, A.Tishechkin”/ “LSAM0013239” / “*Operclipygus* sp #12, Hist 133, Yasuní NP Inventory A.K.Tishechkin det 2010” (FMNH). **Paratypes** (1): same locality as the type, 4–17 July 1999 (LSAM).

#### Other material.

**BOLIVIA: Cochabamba:** 1: Est. Biol. Valle Sajta, Univ. San Simon, 67.5km E Villa Tunari, 17°06'19"S, 64°46'57"W, 300m, 9–13.ii.1999, FIT, lowland rain forest, F. Genier (CMNC); **BRAZIL: Mato Grosso:** 1: Cotriguaçu, Fazenda São Nicolau, 9°50.3'S, 58°15.05'W, xii.2010, FIT, F.Z. Vaz-de-Mello (CEMT).

**Figure 100.**
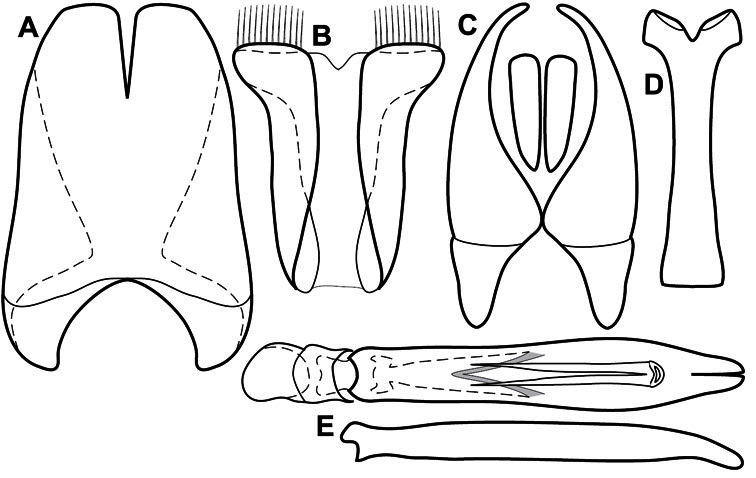
Male genitalia of *Operclipygus abbreviatus*. **A** T8 **B** S8 **C** T9 & T10 **D** S9 **E** Aedeagus, dorsal and lateral views.

**Diagnostic description.** Length: 1.90–2.09 mm, width: 1.50–1.65 mm; body rufobrunneus, elongate oval, widest behind humeri; frons weakly depressed at middle, sides of frontal stria divergent between eyes, rather abruptly bent inward, complete across front; labrum about 2.5× wider than long, emarginate apically; left mandible with broad, blunt basal tooth, right with small, subacute tooth; pronotal disk with distinct, narrow prescutellar impression slightly longer than scutellum; disk with fine, sparse ground punctation, few slightly coarser punctures at sides; marginal stria interrupted behind head; lateral submarginal stria complete along side, curving inward, nearly meeting anterior submarginal, which is barely recurved posterad at sides; median pronotal gland openings just beyond ends of anterior stria, about 6 puncture widths from anterior margin; elytra with outer subhumeral stria present in apical half, inner subhumeral stria absent, striae 1-3 complete, 4^th^ in apical half, 5^th^ stria present in apical third, sutural stria present in apical three-fourths; prosternal keel weakly produced at base, carinal striae sinuate, united anteriorly about one-fifth short of presternal suture, united posteriorly along keel margin; prosternal lobe with marginal stria strongly abbreviated at sides; mesoventrite narrowly emarginate at middle, marginal stria complete; mesometaventral stria broadly arched forward just beyond middle of mesoventral disk, continued by lateral metaventral stria toward outer corner of metacoxa, abbreviated at apex; 1^st^ abdominal ventrite with inner lateral stria complete, outer absent; propygidium with moderately large, shallow punctures sparsely scattered in basal half, smaller and sparser toward apical margin; pygidium with fine, dense ground punctation, with a few coarser punctures along basal margin, otherwise lacking; marginal pygidial sulcus present along apical half of margin, obsolete toward base. Male genitalia (Figs 100A–E): accessory sclerites absent; T8 elongate, with sides slightly convergent, apical emargination deep and narrow, basal emargination broad, tangential to basal membrane attachment line, ventrolateral apodemes most strongly developed near base, narrowed to apex; S8 with sides strongly expanded near apex, apices broad, truncate, with dense comb of setae, halves distant ventrally; T9 with sides rounded, apices subacute, opposing at apex; T10 somewhat reduced, not filling T9 opening, halves separate; S9 with stem narrowest at middle, weakly widened to truncate base, apex with narrow median emargination, apical flanges short, separate; tegmen more or less parallel-sided, weakly expanded near apex, then narrowed to blunt apices, apex only weakly curved ventrad; medioventral process moderately strongly sclerotized, subacute with point about one-fourth from base, but not or very weakly projecting beneath; basal piece about one-fourth tegmen length; median lobe about two-thirds tegmen length, proximal apodemes long, not strongly differentiated, evenly tapered along length, gradually narrowed toward tegmen base.

#### Remarks.

This species can be distinguished by the combination of divergent, subangulate frontal stria (Fig. 98G), short recurved arms of anterior submarginal pronotal stria (Fig. 98F), the minimally distinguishable coarse punctation of the pygidium (amidst the dense ground punctation; Fig. 98H), and the basally obsolete marginal pygidial sulcus. Specimens from localities other than the type locality vary slightly in pygidial punctation and extent of marginal sulcus, and are therefore excluded as types, but they are clearly closely related, and considered part of a broadly defined species.

#### Etymology.

This species’ name refers to its basally abbreviated marginal pygidial sulcus.

### Operclipygus pygidialis

(Lewis, 1908)

http://species-id.net/wiki/Operclipygus_pygidialis

**Figure 101.**
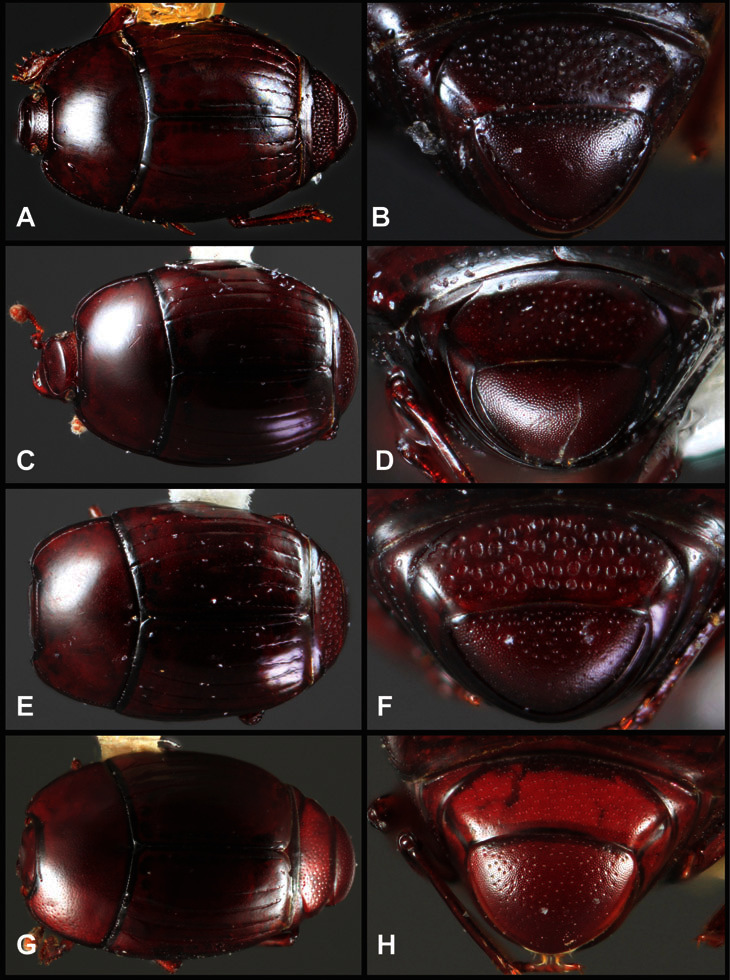
Various *Operclipygus* species. **A** Dorsal habitus of *Operclipygus pygidialis* (lectotype) **B** Pygidia of *Operclipygus pygidialis* **C** Dorsal habitus of *Operclipygus faltistrius* **D** Pygidia of *Operclipygus faltistrius* **E** Dorsal habitus of *Operclipygus limonensis* **F **Pygidia of *Operclipygus limonensis* **G** Dorsal habitus of *Operclipygus wenzeli* **H** Pygidia of *Operclipygus wenzeli*.

Figs 101A–B 102A–C, H Map 34

Phelister pygidialis Lewis, 1908: 157; *Pseudister pygidialis*: Hinton (1935c: 14); *Phelisteroides pygidialis*: Wenzel and Dybas (1941: 454); *Operclipygus pygidialis*: Wenzel (1976: 259).

#### Type locality.

Not specified beyondParaguay.

#### Type material.

**Lectotype**, here designated, probably male: “Paraguay, Dr. Bohls” / “LECTOTYPE *Phelister pygidialis* Lewis M.S.Caterino & A.K.Tishechkin des. 2010” (BMNH). This species was described from an unspecified number of specimens, and the lectotype designation fixes primary type status on the only known original specimen.

#### Other material.

**ECUADOR: Napo**: 1: 20 km S. Tena, 600m, S. Peck, 11.vii. 1976, Berlese forest litter (FMNH). **PERU: Junín:** 1: ~16km NW Satipo, Rio Venado, 11°11.677'S, 74°46.137'W, 1150m, 3–8.iii.2010, A.V. Petrov (AKTC).

#### Diagnostic description.

**Figure 102.**
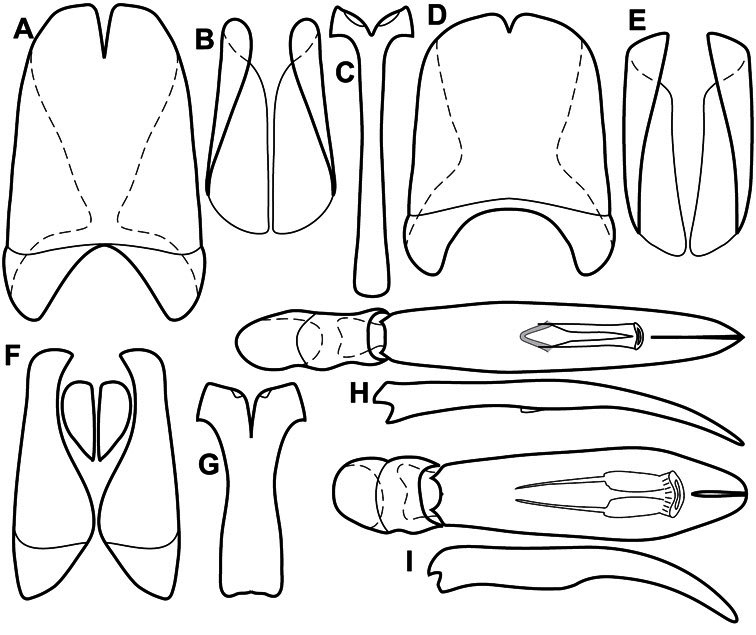
Male genitalia of *Operclipygus* species. **A** T8 of *Operclipygus pygidialis* **B** S8 of *Operclipygus pygidialis* **C** S9 of *Operclipygus pygidialis* **D** T8 of *Operclipygus limonensis* **E** S8 of *Operclipygus limonensis* **F** T9 & T10 of *Operclipygus limonensis* **G** S9 of *Operclipygus limonensis* **H** Aedeagus, dorsal and lateral views, of *Operclipygus pygidialis* **I** Aedeagus, dorsal and lateral views, of *Operclipygus limonensis*.

Length: 1.62–2.06 mm, width: 1.31–1.72 mm; body rufescent, elongate oval, widest behind elytral humeri; frons more or less flat, frontal stria with sides divergent between eyes, weakly sinuate over antennal bases, complete, arcuate anteriorly; epistoma flat at base, weakly convex anteriorly; labrum short, about one-third as long as wide, apex straight; left mandible untoothed, right with acute basal tooth; pronotal disk weakly flattened at base, with small, indistinct prescutellar impression; ground punctation of pronotal disk fine and sparse, but conspicuous, without coarser punctures at sides; marginal pronotal stria complete at side, ending just mesad anterior corner; lateral submarginal stria complete at side, extending forward to anterior margin, replacing marginal for a short distance, ending behind eye; anterior submarginal stria present, briefly recurved posterad at sides; median pronotal gland openings situated laterad ends of anterior submarginal stria; elytron with single complete, sinuate epipleural stria, marginal bead broad and smooth beneath; outer subhumeral stria fine, complete, inner subhumeral absent, striae 1-3 complete, 4^th^ stria present in apical half, 5^th^ stria present in apical third, connected to apex of 4^th^ stria by apical arch, sutural stria present in apical two-thirds, markedly widened anterad; prosternal keel produced, arcuate at base, carinal striae complete, convergent from base to middle, diverging slightly anteriorly, connected by anterior arch; prosternal lobe narrow, long, with complete marginal stria; mesoventral margin shallowly emarginate, with complete, fine marginal stria; mesometaventral stria arched forward at middle, sinuate at side, lateral metaventral stria extending toward outer corner of metacoxa, abbreviated at apex; 1^st^ abdominal ventrite with two complete lateral striae; ventrites 2-4 with single regular series of small punctures at sides, obsolete at middle; propygidium about two-thirds length of pygidium (which appears shorter than normal, rather than propygidium appearing longer than normal), with uniform medium punctures separated by about their diameters, with fine sparse ground punctation interspersed; pygidium with fine, dense ground punctation, with larger punctures sparse, more concentrated along basal margin and onto basomedial part of disk; marginal pygidial sulcus deep, strongly crenulate on inner and outer edges. Male genitalia (Figs 102A–C, H): accessory sclerites absent; T8 with sides evenly convergent from near base to apex, basal emargination deep and broad, meeting basal membrane attachment line at middle, apices obliquely subtruncate, with narrow apical emargination, ventrolateral apodemes broadest near base, almost meeting at midline, narrowed apicad; S8 with sides parallel, apical guides approximately consistent in width from base to apex, ventral halves approximate in basal fourth, divergent to apex; T9 with sides evenly rounded, apices acute, not strongly convergent; halves of T10 approximate along midline; S9 with stem narrow, widened only slightly to narrowly rounded base, with very small apical emargination and separate apical flanges; tegmen widest just beyond midpoint, evenly narrowed to base and apex, apical half curving downward weakly, with medioventral process ‘U’-shaped, projecting beneath near midpoint of tegmen; basal piece about one-third tegmen length; median lobe about one-fourth tegmen length, broad portion of proximal apodemes short, separate.

#### Remarks.

*Operclipygus pygidialis* has some superficial similarity with members of the *Operclipygus impuncticollis* group, especially in the small size, arrangement of pronotal striae (Fig. 101A), and coarse pygidial sulcus (Fig. 101B), although the male genitalia show sufficient differences to suggest that this is convergence. It is also similar to the following species, *Operclipygus faltistrius*, in several characters. *Operclipygus pygidialis* can be separated from the *Operclipygus impuncticollis* group species by the complete outer subhumeral stria and by its overall pygidial shape, which is shorter and broader, and from *Operclipygus faltistrius* by its small, deep propygidial punctures, and deep pygidial sulcus.

### Operclipygus faltistrius sp. n.

urn:lsid:zoobank.org:act:CB0A396C-69F2-4A71-88D5-7D9098C5C49F

http://species-id.net/wiki/Operclipygus_faltistrius

Figs 101C–D Map 34

#### Type locality.

ECUADOR: Pichincha: Rio Palenque Research Center [0°35'S, 79°22'W].

#### Type material.

**Holotype** **of undetermined sex**: “ECUADOR PICHINCHA CCRP [Centro Cientifico Rio Palenque] 17 JUN 1980 SSandoval”/ “Caterino/Tishechkin Exosternini Voucher EXO-02213” (FMNH).

#### Diagnostic description.

Length: 1.78 mm, width: 1.47 mm; body rufescent, elongate oval, sides rounded; frons rather narrow, sides rounded, frontal stria finely impressed, complete across front, weakly sinuate over antennal bases; epistoma with ground punctation slightly denser than that of frons, weakly emarginate apically; labrum narrowed to apex, weakly emarginate; pronotal disk with very faint prescutellar impression, fine, sparse ground punctation, lacking coarse lateral punctures; marginal pronotal stria broadly interrupted behind head; lateral submarginal stria complete along side, nearly meeting anterior submarginal stria, which is very briefly recurved posterad at sides; median pronotal gland openings adjacent to ends of anterior submarginal stria; elytron with single complete epipleural stria, outer subhumeral stria complete, inner subhumeral stria absent, dorsal striae 1-3 complete, 4^th^ stria present in apical half, 5^th^ stria present in apical third, sutural stria present in apical two-thirds; prosternal keel weakly produced at base, with complete carinal striae subparallel throughout their lengths, connected basally and apically; prosternal lobe rather narrowly rounded, with complete marginal stria; anterior margin of mesoventrite shallowly emarginate, with complete marginal stria; mesometaventral stria weakly arched forward to near middle of mesoventrite, sinuate at side, continued posterolaterad by lateral metaventral stria toward outer third of metacoxa, weakly abbreviated apically; 1^st^ abdominal ventrite with two complete lateral striae; propygidium with fine sparse ground punctation which becomes slightly denser toward the sides, with small, deep secondary punctures separated by their diameters or slightly more; pygidium with fine, dense ground punctation, with secondary punctures inconspicuous except along basal margin; marginal pygidial sulcus absent. Male: not known.

#### Remarks.

While this species seems to be closely related to *Operclipygus pygidialis*, which is nearly identical in body shape and striation, the absence of any indication of a marginal pygidial sulcus (Fig. 101D) distinguishes this species immediately. We attempted but were not able to dissect this lone specimen for fear of damaging it. It is hoped more material will become available to allow study of male genitalia.

#### Etymology.

The name of this species is a combination of the Spanish ‘falti-’ or lack, and strius, referring to its lack of a pygidial sulcus.

### Operclipygus limonensis sp. n.

urn:lsid:zoobank.org:act:4DAE6C82-1E8B-4F8F-ACC7-0D9A9C693124

http://species-id.net/wiki/Operclipygus_limonensis

Figs 101E–F 102D–G–I Map 34

#### Type locality. 

COSTA RICA: Limón: Sector Cerro Cocori, E. Rojas Farm [10.60°N, 83.72°W].

#### Type material.

**Holotype male**: “Sector Cerro Cocori, Fca. de E. Rojas, 150 m, Prov. Limón, COSTA RICA. May 1993. E. Rojas, L-N-286000, 567500”/ “InbioCRI001349337” (INBIO). **Paratypes** (2): **COSTA RICA: Limón:** 1: Area Cons. Tortuguero, Sector Cerro Cocori, Fca. De E. Rojas, 150m, v.1993, E. Rojas, (INBIO); 1: Sardinas, Barra del Colorado, 15m, 6–12.xi.1994, F.V. Araya, (INBIO).

#### Diagnostic description.

Length: 1.75–1.87 mm, width: 1.37–1.44 mm; body rufobrunneus, elongate oval, widest at humeri; frons quite convex, frontal stria fine, complete, rounded between eyes, sinuate over antennal bases, arcuate across front; labrum small, weakly emarginate; pronotal disk without coarser lateral punctures; pronotum broadly depressed at base, antescutellar fovea present, but rather ill-defined, slightly longer than scutellum; lateral submarginal pronotal stria complete and rather distant from margin, curving inward along anterior margin and just meeting anterior submarginal stria, which is barely recurved at sides; median gland openings just posterad ends of anterior submarginal stria, about 5 puncture widths from anterior margin; elytron with one complete epipleural stria, outer subhumeral stria nearly complete, but either interrupted at middle, or abbreviated from base in all specimens, inner subhumeral stria absent, striae 1-3 complete, 4^th^ stria present in apical half, 5^th^ stria distinctly shorter than 4^th^, but somewhat varied, sutural stria present in apical two-thirds, striae not widened apically; prosternum with keel weakly produced at base, carinal striae subparallel, weakly convergent to front, meeting in broad arch short of presternal suture; mesoventrite shallowly emarginate at middle, marginal stria complete; mesometaventral stria narrowly arched forward, sinuate near mesocoxae, continued by lateral metaventral stria obliquely toward posterior third of metepisternum, abbreviated apically; 1^st^ abdominal ventrite with single lateral stria; propygidium with ground punctation almost invisible, with large, irregularly oval, shallow punctures separated by about their diameters; pygidium with dense fine ground punctures, coarser punctures concentrated near basal margin, but sparsely scattered throughout; marginal pygidial sulcus complete, deep, with edges weakly crenulate.Male genitalia (Figs 102D–G, I):accessory sclerites absent; T8 rather short, with sides subparallel, basal apodemes narrow, basal emargination broad, shallow, basal membrane attachment line just distad apex of basal emargination, apical emargination narrow, shallow, ventrolateral apodemes narrow, arcuate, distantly separated beneath; S8 with sides weakly divergent, guides markedly widened toward apex, subtruncate, ventral halves separate throughout, divergent to apex; T9 short, subparallel, apices broad, acute at inner margin; T10 with halves separate; S9 short, broad, base emarginate, apex with narrow emargination, apical flanges small, just at inner corners, separate; tegmen stout, widest near apex, bluntly rounded apically, medioventral process absent; basal piece short, about one-fourth tegmen length; median lobe about one-half tegmen length in total, gonopore wide, proximal apodemes strongly differentiated into short thick arms and long filamentous proximal ends.

#### Remarks.

The small size of this species suggests relationships with the *Operclipygus hospes* group, but this is not supported by the male genitalia (Fig. 102D–G, I), which are quite isolated and unusual. It also shares many external characters with *Operclipygus pygidialis*, including the complete outer subhumeral stria, moderately broad lateral pronotal bead, and deep pygidial sulcus, but again differs significantly in male genitalia. Short of dissection, the species can be most readily recognized by the very short posterior arms of the anterior submarginal pronotal stria (Fig. 101E), in combination with a complete lateral submarginal pronotal stria which is distant from the margin, the more or less complete outer subhumeral elytral stria, and the rather large, very shallow propygidial punctures (Fig. 101F).

#### Etymology.

This species’ name refers to the sole Costa Rican province where it has been found.

### Operclipygus wenzeli sp. n.

urn:lsid:zoobank.org:act:AADFB7F4-A71B-4642-B9B5-D6E3DDAC06A1

http://species-id.net/wiki/Operclipygus_wenzeli

Figs 101G–H Map 34

#### Type locality.

BOLIVIA: La Paz:Espía [16°28'S, 67°20'W].

#### Type material.

**Holotype female**: “Espia. RioBopi Boliv.W.M.Mann, July-Aug, 1921” / “Mulford Bio.Expl 1921-22” / “*Phelister* #77 det.R.Wenzel 19”/ “Caterino/Tishechkin Exosternini Voucher EXO-00276” (USNM).

#### Diagnostic description.

Length: 2.15 mm, width: 1.81 mm; body rufescent, broadly oval, widest behind humeri; frons weakly depressed at middle, with conspicuous fine ground punctation; frontal stria rounded between eyes, with central portion detached from sides; pronotal disk with distinct oval prescutellar impression slightly longer than scutellum, ground punctation fine, with numerous coarser punctures along and close to lateral margin; marginal pronotal stria interrupted behind head, lateral submarginal stria obsolete in basal half; anterior submarginal stria divergent from margin at sides, but not recurved; median pronotal gland openings situated just laterad ends of anterior submarginal stria, about 5 puncture widths from anterior margin; elytra with single complete epipleural stria somewhat deeply and crenulately impressed, outer subhumeral stria present in apical half, and as a basal fragment, inner subhumeral stria absent, striae 1-3 complete, 4^th^ stria present in apical half, and as distinct basal puncture, 5^th^ stria absent, sutural stria present in apical three-fourths; prosternal keel weakly emarginate at base, carinal striae divergent, disconnected basally, weakly convergent anteriorly, united in broad arch about one-fourth from presternal suture; mesoventrite weakly projecting at middle, marginal stria complete; mesometaventral stria broadly arched forward beyond middle of mesoventral disk, arc continuous with lateral metaventral stria which extends to middle of metacoxa; 1^st^ abdominal ventrite with single, complete lateral stria; propygidium uniformly sparsely covered with small, very shallow punctures separated by about their diameters; pygidium with fine, shallow but dense ground punctation, with coarser punctures evenly interspersed; marginal pygidial sulcus absent. Male not known.

#### Remarks.

Although known from a single female, this species is more than adequately distinct enough to justify establishment of a species. It may be related to *Operclipygus falini*, with which it shares the abbreviated lateral submarginal pronotal stria (Fig. 101G) and detached central portion of frontal stria. However, these are rather insubstantial similarities, and it differs in its absence of a 5^th^ dorsal elytral stria, the lack of any punctures on the disk of the 1^st^ abdominal ventrite, and the minutely, densely punctate pygidium which lacks any marginal sulcus (Fig. 101H).

#### Etymology.

We name this species in honor of Rupert Wenzel, the 20^th^ century's pre-eminent histerid specialist, in recognition of his contributions to our understanding of *Operclipygus*, and his recognition of this species as undescribed.

### Operclipygus iheringi

(Bickhardt, 1917)

http://species-id.net/wiki/Operclipygus_iheringi

**Figure 103.**
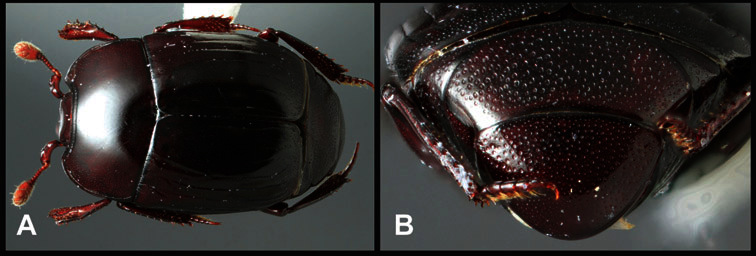
*Operclipygus iheringi*. **A** Dorsal habitus **B** Pygidia.

Figs 103A–B 104A–E Map 34

Phelister iheringi Bickhardt, 1917: 215; *Operclipygus iheringi*: Mazur (1984: 258).

#### Type locality.

BRAZIL: São Paulo: Rair da Serra [exact location unknown].

#### Type material.

**Lectotype,** here designated: “Rair da Serra, São Paulo” / “Type” / “9968” / “LECTOTYPE *Phelister iheringi* Bickhardt, 1917 M.S. Caterino & A.K. Tishechkin des. 2010” (ZMHB). **Paralectotype**: same data as lectotype (ZMHB). This species was described from an unspecified number of specimens, and the lectotype designation fixes primary type status on one of the two known syntypes.

#### Other material.

**BRAZIL: Paraná:** 1: Piraquara, Mananciais da Serra, 1000m, i.2007, P. Grossi (UFPR); 1: Piraquara, Sanepar, 18.vii.2001, P. Grossi (UFPR); 2: Guartelá, Campo São Paolo, 24°32'S, 50°17'W, 900m, xi.2007, pitfall, E. Grossi (UFPR).

#### Diagnostic description.

**Figure 104.**
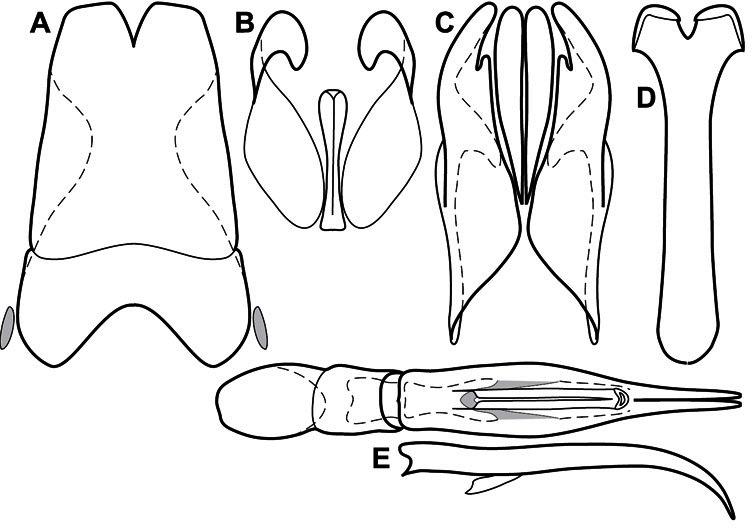
Male genitalia of *Operclipygus iheringi*. **A** T8 **B** S8 **C** T9 & T10 **D** S9 **E** Aedeagus, dorsal and lateral views.

Length: 3.21–3.46 mm, width: 2.56–2.87 mm; body piceous, large, elongate, subdepressed; frons weakly depressed at middle, finely punctate; frontal stria rounded at sides, with a short, detached fragment in front, supraorbital stria complete, connected to sides of frontal; epistoma convex; labrum wide, weakly emarginate; both mandibles with bifid basal teeth; antennal club markedly elongate with basal and middle annuli interrupted; pronotum with lateral margins slightly convergent in basal half, weakly curved inward to apex; pronotal disk with distinct narrow prescutellar impression about as long as scutellum, disk finely punctate throughout, with few barely coarser lateral punctures; marginal pronotal stria complete or narrowly interrupted behind head; submarginal stria continuous from sides across front; central portion of anterior pronotal margin faintly projecting at middle; anterior pronotal margin with a single pair of glands, behind eye on each side; elytron with two complete epipleural punctures, outer subhumeral stria nearly complete, sinuate at middle, frequently abbreviated at base, inner subhumeral stria present in middle, variously abbreviated basally and apically, striae 1-3 complete, 4^th^ stria present in apical third, 5^th^ stria present in apical fourth, sutural stria present in apical half to two-thirds; elytra with broad band of small punctures present along apical margin; prosternal keel broad, flat, weakly emarginate at base, carinal striae convergent to apex, rarely connected by arch; mesoventral margin sinuate, median projection small, acute, marginal stria complete or narrowly interrupted; mesometaventral stria arched to subangulate at middle, reaching anterior half of mesoventral disk; lateral metaventral stria extending posterolaterad to middle of metacoxa; 1^st^ abdominal ventrite with two complete lateral striae very close together, central part of disk with numerous small punctures near these striae; propygidium uniformly covered with small, round punctures separated by slightly less than their diameters; pygidium lacking dense ground punctation, with small punctures diminishing toward apex; marginal pygidial sulcus fine, variably abbreviated at sides, never attaining base. Male genitalia (Figs 104A–E): accessory sclerites present, small; T8 with sides subparallel at middle, slightly divergent basally, desclerotized at apicolateral angles, convergent to apex, basal emargination shallow, reaching less than halfway to basal membrane attachment line, ventrolateral apodemes evenly rounded, distant beneath; S8 with unique median longitudinal sclerite, inner edges of lateral halves strongly divergent, apical guides strongly developed apically, with weakly hooked inner edges; T9 widest near apex, narrowed to basal apodemes, with strongly dentate ventrolateral apodemes, apices narrowed, blunt; T10 completely divided; S9 narrow at middle, weakly widened to narrowly rounded base, apex distinctly emarginate, apical flanges separate; tegmen with sides subparallel in basal half, rather abruptly narrowed to apices, medioventral process narrowly ‘U’-shaped, projecting strongly beneath, about one-fourth from base; basal piece about one-third tegmen length; median lobe about one-half tegmen length, proximal apodemes not obviously differentiated.

#### Remarks.

This is a very distinctive species of rather obscure relationships. It is much larger than average, with the lateral submarginal pronotal stria close to the margin (Fig. 103A), an unusually elongate antennal club, and a broad band of apical marginal elytral punctures. The median ventral sclerite on the 8^th^ sternite of the male genitalia (Fig. 104B) is unique in the genus. A few characters suggest relationships to the *Operclipygus farctus* group, including the desclerotized apicolateral angles of the male 8^th^ tergite. But the many other unique features, particularly the presence of only a single pair of anterior pronotal gland openings, preclude a confident placement at present.

### Operclipygus angustisternus

(Wenzel, 1944)

http://species-id.net/wiki/Operclipygus_angustisternus

**Figure 105.**
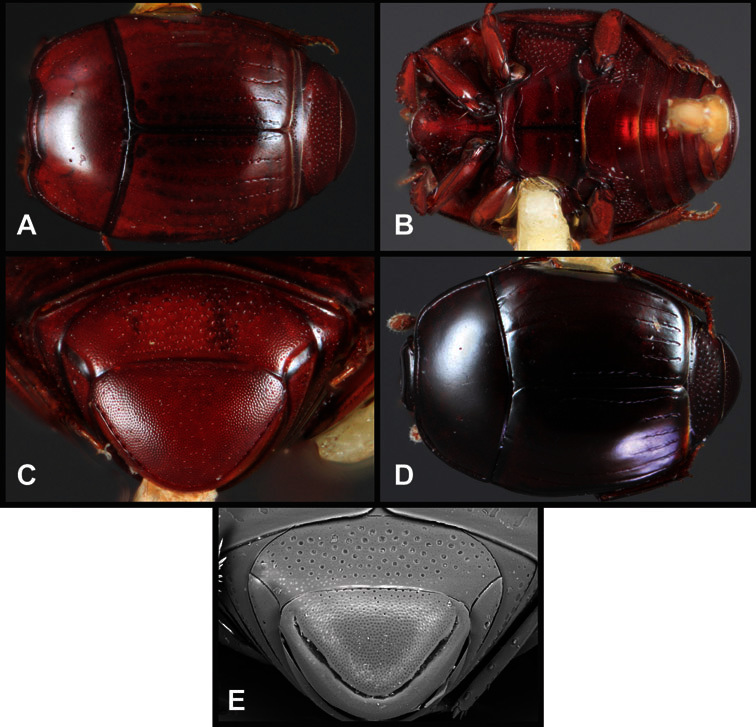
Various *Operclipygus* species. **A** Dorsal habitus of *Operclipygus angustisternus* **B** Ventral habitus of *Operclipygus angustisternus* **C** Pygidia of *Operclipygus angustisternus* **D** Dorsal habitus of *Operclipygus shorti* **E** Pygidia of *Operclipygus shorti*.

**Map 35.**
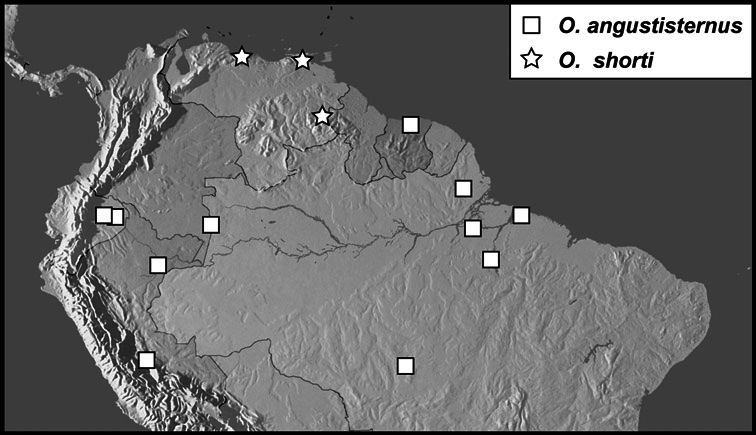
Records of the *Operclipygus* incertae sedis spp.

Figs 105A–C 106A–E Map 35

Phelisteroides angustisternus Wenzel, 1944: 142; *Operclipygus angustisternus*: Wenzel 1976: 257.

#### Type locality.

Not specified beyond Pará State, Brazil.

#### Type material.

**Holotype male**, slightly teneral: “Para” / “Brazil” / “June” / “Type *Phelisteroides angustisternus* Wenzel” (FMNH), examined 2006.

#### Other material.

**BRAZIL: Amapá:** 2: Serra do Navio, 0°59'N, 52°00'W, 1–14.v.1991, FIT (CHND); **Mato Grosso:** 1: Sinop, 11°49'S, 55°29'W, 12–24.vi.1985, FIT (CHND); **Pará:** 1: Tucuruí, 3°45'S, 49°40'W, iv.1986, FIT (CHND); 1: v.1986, FIT (CHND), 1: 20.v-15.vi.1987, FIT (CHND); 2: IPEAN, Belém, Utinga, 1°27'S, 48°26'W, ix.1984, FIT (CHND), 1: ix.1985, FIT (CHND); 1: Melgaço, Rio Marinaú, 1°51.5'S, 51°20'W, 29.x-13.xi.1993, FIT (CHND), 1: 31.x-13.xi.1993, FIT (CHND). **COLOMBIA: Vaupés:** 1: Est. Biol. Caparú, Rio Apoporis, 1.1°S, 69.5°W, 27.ix-1.xii.1995, FIT, Black-water terrace forest on sandy soils, B.D. Gill (BDGC); 1: Parque Nac. Mosiro-Itajura (Caparú), Centro Ambiental, 1°04'S, 69°31'W, 60m, 20–30.i.2003, FIT, D. Arias & M. Sharkey (IAVH). **ECUADOR: Orellana:** 1:Yasuní Res. Stn., mid.Rio Tiputini, 0°40.5'S, 76°24'W, 28.vi-5.vii.1999, FIT, C.E. Carlton & A.K. Tishechkin (LSAM), 1: 12–20.vii.1999, FIT, A.K. Tishechkin (LSAM), 1: 5–11.vii.1999 (LSAM), 1: 20–23.vi.1999 (LSAM), 1: 23–30.vi.1999LSAM); 1: Yasuní Res. Stn., 00°40'28"S, 76°38'50"W, 215m, 5–10.ix.1999, FIT, primary forest, E.G. Riley (TAMU); 1: Parque Nac. Yasuní, Via Maxus at Puente Piraña, 1°39.5'S, 76°26'W, 14–20.vii.2008, FIT, A.K. Tishechkin (AKTC); 1: Payamino Research Station, 0°29'36.01"S, 77°17'29.15"W, 300m, 30.vii-12.viii.2007, FIT, tropical rainforest, CPDT Gillett (BMNH); 1: Tiputini Biodiversity Station, 0°38.2'S, 76°8.9'W, 27–31.vii.2008, FIT, A.K. Tishechkin (AKTC); 1: Tiputini Biodiversity Station, 0°38'0"S, 76°9'0"W, 220m, 5–25.ix.2000, D.J. Inward & K.A. Jackson (BMNH). **PERU: Junín:** 1: 11km NE Puerto Ocopa, Los Olivos, 11°7.00'S, 74°15.52'W, 1200m, 30–31.iii.2009, Window trap, A.V. Petrov (AKTC); 1: 7.5km NE Puerto Ocopa, Cananeden, 11°4.9'S, 74°16.1'W, 1180m, 6.ii.2008, A.V. Petrov (AKTC); 2: ~16km NW Satipo, Rio Venado, 11°11.677'S, 74°46.137'W, 1150m, 3–8.iii.2010, A.V. Petrov (AKTC, MUSM); **Loreto:** 1: 68km SW Iquitos to Nauta, Rio Itaya, 4°11'S, 73°26'W, 110m, 28.ii.2008, A.V. Petrov (AKTC); 1: km 63, rd. Iquitos - Nauta, Rio Itaya, 4°15.205'S, 73°26.074'W, 140m, 8–17.i.2010, A.V. Petrov (MUSM). **SURINAME: Para**: 2: nr. Overbridge River Resort, 5°31.8'N, 55°3.5'W, 13–15.ii.2010, FIT, C. Gillet, P. Skelley, W. Warner (FSCA), 5: 15–18.ii.2010, FIT, C. Gillet, P. Skelley, W. Warner (FSCA); 2: near Overbridge, 05°31'10"N, 55°04'10"W, 10–14.ii.2010, FIT, W.B. Warner (WBWC), 1: 14–18.ii.2010 (WBWC).

#### Diagnostic description.

**Figure 106.**
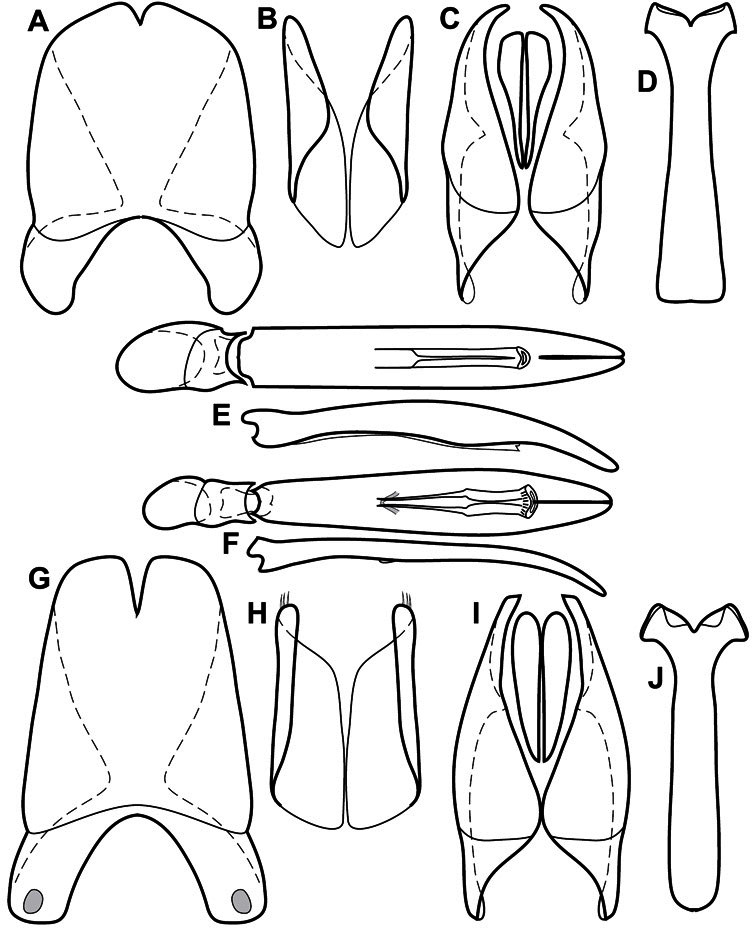
Male genitalia of *Operclipygus* species. **A** T8 of *Operclipygus angustisternus* **B** S8 of *Operclipygus angustisternus* **C** T9 & T10 of *Operclipygus angustisternus* **D** S9 of *Operclipygus angustisternus* **E** Aedeagus, dorsal and lateral views, of *Operclipygus angustisternus* **F** Aedeagus, dorsal and lateral views, of *Operclipygus shorti* **G** T8 of *Operclipygus shorti* **H** S8 of *Operclipygus shorti* **I** T9 & T10 of *Operclipygus shorti* **J** S9 of *Operclipygus shorti*.

Length: 1.68–2.09 mm, width: 1.50–1.87 mm; body rufescent, elongate oval, sides weakly rounded; frons strongly but narrowly depressed at middle; frontal stria with sides divergent, weakly rounded, strongly sinuate over antennal bases, arcuate anterad; supraorbital stria finely impressed, but strongly arcuate dorsad onto vertex, weakly connected with sides of frontal stria; epistoma flat, weakly emarginate at apex; labrum about 3× as wide as long, weakly emarginate, asymmetrical, left side more strongly produced than right, weak projection beneath; left mandible untoothed, right with small, acute basal tooth; pronotum with sides rounded, rather strongly convergent to front, with anterior margin markedly produced over head, lacking prescutellar impression; pronotal disk with fine, sparse ground punctation, with at least a few (up to ~12) coarser punctures at sides; lateral submarginal pronotal stria complete along side, curved inward anteriorly, nearly meeting anterior submarginal stria, which is weakly arcuate, barely turned posterad at sides; median pronotal gland openings annulate, situated behind ends of recurved anterior stria, about two-thirds pronotal length from anterior margin; elytra with two complete epipleural striae, outer subhumeral stria present in posterior one-third to one-half, inner subhumeral stria absent, elytron swollen in subhumeral region, dorsal striae 1-3 complete, 4^th^ stria present in apical two-thirds, 5^th^ stria present in apical third, sutural present in apical three-fourths; prosternal keel rounded, projecting posterad, carinal striae variably shortened, meeting about one-third from presternal suture, anterior one-third of keel narrow; prosternal lobe rather short, marginal stria abbreviated at sides; anterior mesoventral margin emarginate, marginal stria complete; mesometaventral stria weakly arched forward at middle, sinuate at sides, continued by lateral metaventral stria posterad toward inner third of metacoxa; 1^st^ abdominal ventrite with three lateral striae, all three usually abbreviated posteriorly, innermost extending mediad for some distance along anterior margin of ventrite, nearly continuously in some individuals, outermost shortest of the three, ending at an annulate postmetacoxal fovea; sides of propygidium and entire pygidium with dense, fine ground punctation; propygidium with irregular, moderately large punctures at middle, separated by about one-fourth their diameters; pygidium with few barely larger punctures standing out from ground punctation; marginal pygidial stria narrow but deeply impressed apically, becoming shallower, as an interconnected series of punctures toward base. Male genitalia (Figs 106A–E): accessory sclerites absent; T8 with sides subparallel in basal three-fourths, angulate to short, narrowly but shallowly emarginate apex, basal emargination deep, broad, reaching basal membrane attachment line, ventrolateral apodemes most strongly developed at base, narrowing toward apex; S8 with sides subparallel, apical guides narrowing from base to narrowly rounded apex; T9 with sides rounded, with narrow, convergent apices; T10 with halves separate; S9 with stem desclerotized along midline, widest basally, base truncate, with equilateral apical emargination, apical flanges separate; tegmen parallel-sided in basal three-fourths, narrowed to blunt apex, lacking medioventral process; basal piece about one-fourth tegmen length; median lobe narrow, about one-half tegmen length.

#### Remarks.

The presence of dense ground punctation on the sides of the propygidium (Fig. 105C) is restricted to only a few species of *Operclipygus*. *Operclipygus angustisternus* is further distinguished by the strongly sinuate frontal and strongly arched supraorbital striae, the outwardly projecting anterior pronotal margin (Fig. 105A), and by the presence of three lateral striae on the 1^st^ abdominal ventrite (Fig. 105B).

### Operclipygus shorti sp. n.

urn:lsid:zoobank.org:act:2E664762-6E3C-4613-A61A-8866E57C6342

http://species-id.net/wiki/Operclipygus_shorti

Figs 105D–E 106F–J Map 35

#### Type locality.

VENEZUELA: Bolívar: 6 km S San Isidro [6.06°N, 61.40°W].

#### Type material.

**Holotype male**: “VENEZUELA: Bolívar, 6km S San Iisidro[sic], Km 88, 25.VI.-11.VII.1987” / “FMHD#7-1061, lowland rainforest, S.&J. Peck, P#87-45, FIT, FIELD MUSEUM” / “FMNH-INS 0000 069 175” (FMNH). **Paratypes** (5): 2: same data as holotype (FMNH); **VENEZUELA: Aragua**: 1: Henri Pittier NP, Rancho Grande, 1.x.2006, fallen bromeliads, A.E.Z. Short, DNA Extract MSC-1902 (SEMC); 1: Rancho Grande, 1150m, 4.vii–9.viii.1986, B.D. Gill (BDGC); 1: **Miranda**, Capaya, 250m, 24.iii.1967, J.L. Garcia, (MHNLS).

#### Diagnostic description.

Length: 1.97–2.06 mm, width: 1.65–1.84 mm; body rufopiceous, elongate oval; frons very weakly impressed; frontal stria rounded at sides, interrupted over antennal bases, arcuate across front; supraorbital stria fine, more or less complete, but narrowly detached from sides of frontal stria; epistoma weakly convex; labrum about twice as wide as long, weakly, asymmetrically emarginate apically; both mandibles with small, subacute basal tooth; pronotum lacking prescutellar impression, with very fine, inconspicuous ground punctation, lacking coarse lateral punctures; marginal pronotal stria broadly interrupted behind head; submarginal pronotal stria continuous along lateral and anterior margins; median pronotal gland openings simple, about two-thirds pronotal length from anterior margin; elytron with one complete epipleural stria, outer subhumeral stria complete or interrupted at middle, inner subhumeral stria absent, dorsal striae 1-2 complete, 3^rd^ stria present as fine scratch in basal third and as stria in apical third, 4^th^ and 5^th^ striae present in apical fourth, sutural stria present in apical two-thirds; prosternal keel weakly projecting at base, with complete carinal striae connected in rather broad anterior arch; anterior mesoventral margin shallowly emarginate, with complete marginal stria; mesometaventral stria broadly arched forward to near marginal mesoventral, sinuate at sides, continued posterolaterad by lateral metaventral stria, abbreviated apically; 1^st^ abdominal ventrite with single lateral stria abbreviated posteriorly; postmetacoxal fovea small, situated behind inner corner of metacoxa; propygidium with ground punctation fine and sparse, with coarse punctures small, rather deep, and separated by their diameters or slightly more; pygidium with fine, dense ground punctation throughout, lacking coarser punctures except along basal margin; marginal pygidial sulcus very strong and deep, widening slightly to basal corners, but lacking larger basal foveae. Male genitalia (Figs 106F–J): accessory sclerites present, small; T8 with sides evenly convergent to subtruncate apex, apical emargination narrow, basal emargination narrow and deep, nearly meeting basal membrane attachment line, ventrolateral apodemes well developed basally, narrowed to apex; S8 with sides subparallel, apical guides narrow, evenly developed along sides, halves approximate along most of ventral midline; T9 with sides subparallel, weakly arcuate to narrow, weakly convergent apices; T10 with halves separate; S9 with stem narrow, parallel sided, rounded at base, with narrow apical emargination, apical flanges thin, separate; tegmen narrow, with sides weakly rounded, widest in apical half, with bluntly subacute apex, medioventral process thin, narrowly ‘U’-shaped, projecting beneath about one-third from base; median lobe about half tegmen length, with proximal apodemes strongly differentiated into thick portion with finer proximal extensions; basal piece just over one-third tegmen length.

#### Remarks.

The strong pygidial sulcus (Fig. 105E), complete submarginal pronotal stria (Fig. 105D), and position of the median pronotal gland openings would appear to ally this distinctive species with the *Operclipygus fossipygus* group. However, the male genitalia are much more generalized, lacking all the unusual characters of that group. Externally it can be distinguished by the lack of strong foveae at the base of the pygidial marginal sulcus, the single epipleural stria, bilaterally interrupted frontal stria, and single lateral stria on the first abdominal ventrite.

#### Etymology.

We name this species for Andrew Short, collector of the first specimen of the species that we were able to study, in recognition of his efforts cataloging the beetle fauna of Venezuela.

## Discussion

While this study documents a large number of previously unknown species, it is very likely that many more remain to be discovered. Although we had a great wealth of specimen material to work with, there are still many large gaps in sampling, most evidently in much of the central Amazon region. The highest diversity appears to occur on the montane fringes of the Amazon basin, with pockets of diversity and endemism found in some expected places (Guianan Shield, Atlantic Forests) and perhaps some unexpected ones as well, like southeastern Peru. Among poorly sampled areas, the northern Andes of Colombia, and the Guianan Shield parts of Venezeula would appear to be especially promising places to find new species. The Pacific slope of the Andes is generally poorly sampled, but what samples do exist, such as from coastal Ecuador, indicate that there are some localized endemics. Further sampling in forested areas of the Ecuador and northern Peruvian coasts may produce further species. While sampling in southeastern Brazil has been reasonably good, the diversity of unusual and unplaceable species in the region is high, and continued sampling seems likely to yield more diversity.

Central America also hosts a high diversity of species in a couple groups, the *Operclipygus hamistrius* group in the highlands, and the *Operclipygus impunctipennis* group in the lowlands. The bulk of available samples come from Costa Rica and Panama, and the species density in these countries is great. Further sampling in more northern parts of Central America would be very likely to produce additional species.

Conversely, there are some areas within the Neotropics where *Operclipygus* diversity is surprisingly low. In particular, the high Andes host very few species. We have examined many Exosternini from highland Peru and Ecuador, and many species of *Phelister* are found in these areas, but almost no *Operclipygus*. Species diversity also declines abruptly on approaching the temperate zones, both north and south. While three species occur primarily within the Nearctic region, none extend into the subtropical mountains of northwestern Mexico and the southwestern US, and none are known from south temperate areas.

The ecological habits of *Operclipygus* are evidently diverse. Although the majority of specimens have been obtained through passive trapping (flight interception or pitfall), enough ecological data has been recorded on specimen labels to begin to give an idea of the group's habits. It is first of all apparent that the bulk of the species are associated with wet forests. While the drier, more seasonal forests of south-central South America do host a number of unique species, they’re relatively few compared to wet forests, whether lowland or montane. The largest number of at least moderately specific records associate *Operclipygus* species with leaf litter on the forest floor. About 35 species have been recovered through litter sifting. In several cases it appears that the preference is more specific, or at least the species are attracted by fruitfalls and flowerfalls onto the forest floor from above. The lists of species attracted to each of these is largely non-overlapping, but the number of records overall is so few that this probably has little significance. It would be interesting to examine these microhabitats more systematically to see whether preferences might be observed. A relatively small number of species (six) has been collected through arboreal or canopy sampling, and all of these have been more abundantly collected through other means. While some Neotropical Exosternini (especially the genus *Baconia*) do show indications of a distinct canopy fauna, there is no indication of this in *Operclipygus*.

Six of the species have been specifically associated with fungi, including Agaricales, Boletales, and Polyporales, as well as Ascomycota. Interestingly the fungus-associated species include several of the largest and shiniest *Operclipygus*, including two in the *fossipygus* group (*Operclipygus foveipygus*, and *Operclipygus simplicipygus*), suggesting some ecomorphological connection. We would conjecture that several species found in association with dead wood may also be attracted at least in part by fungal (particularly yeast) activity, especially those found in decaying palm and other more recently killed wood.

The ‘typical histerid’ associations with dung and carrion are observed in *Operclipygus*, but not with great frequency. Only a single species has been collected in association with cow dung, two have been attracted to human dung traps, and one was found under tapir dung in a presumably more natural situation. *Operclipygus tripartitus* and *Operclipygus teapensis* have been found in association with bat dung, both in actual caves, as well as in an apparently alternative roosting situation (a specimen of *Operclipygus teapensis* from Hamburg Farm, Costa Rica). *Operclipygus schlingeri*, from Tingo Maria, Peru, was also found in a cave, seemingly likely associated with guano. Carrion records are a little more numerous, with nine species involved. A couple of the most interesting involve *Operclipygus subterraneus*, which was collected in good numbers in buried carrion traps near Curitiba, Paraná, Brazil (under the name *Operclipygus hospes*, by Corréa et al. [2012]). Very little trapping of this type has been carried out anywhere, but the numbers of specimens attracted suggests that this could be an important microhabitat for some otherwise rarely encountered species, and suggests that many *Operclipygus* species may be important tools in forensic investigations.

*Operclipygus* evidently contains several inquilinous species. It appears that most members of the *Operclipygus mirabilis* group are myrmecophilous guests of Attini (leaf-cutting/fungus-growing ants). *Operclipygus mirabilis* itself has been collected multiple times in refuse piles of *Atta colombica*, while *Operclipygus plaumanni* and *Operclipygus mutuca* have both been collected with *Acromyrmex* Mayr. Leaf-cutting ants are also clearly hosts for the *hamistrius* group species *Operclipygus campbelli*, with over 100 individuals found in a single excavation of an *Atta* nest in Guatemala. However, only one of four specimens of *Operclipygus novateutoniae*, and two of ten specimens of *Operclipygus inquilinus*, were found in association with refuse deposits of *Acromyrmex* species. Army ants may also host several species, with one particularly strong case: *Operclipygus ecitonis* has been found in association with *Eciton burchelli* on three separate occasions. Finally, *Aphaenogaster* Mayr (Myrmicinae) are clear hosts of the U.S. *marginellus* group species (*Operclipygus marginellus* and *Operclipygus striatellus*), with nearly all specimens having been collected from their nests. These observations have included both riding on and being carried by the host ants (J. Gruber, pers. comm.). For most other species, the numbers of specimens found in such associations has been so small that the association may simply be accidental or facultative. *Operclipygus juninensis*, *Operclipygus conquisitus*, *Operclipygus sinuatus* and *Operclipygus granulipectus* all have single records mentioning *Eciton* Latreille, while more generic records predominate. Termites are clearly the primary host for *Operclipygus plicicollis*, for which the majority of known specimens have been collected in association with *Nasutitermes* Dudley termite nests.

Finally, a few *Operclipygus* appear to be primarily associated with mammalian burrows. The tuco-tuco genus *Ctenomys* Blainville hosts several Exosternini in subtropical South America, including some *Phelister* and *Pseudister* species, as well as *Operclipygus latemarginatus* (Bickhardt, 1920; Bruch, 1937). We have also seen one collection record of *Operclipygus bidessois* from burrows of pocket gophers in the genus *Orthogeomys* Merriam.

In closing, we will reiterate our hope that this study facilitates the identification of a large component of the neotropical histerid beetle fauna, and that continued collection and documentation of the habits of *Operclipygus* species will help to fill in the numerous gaps in our knowledge of its biology, diversity and relationships.
